# Synthesis of PSI Oligosaccharide for the Development of Total Synthetic Vaccine against *Clostridium difficile*

**DOI:** 10.1021/acs.joc.5c00290

**Published:** 2025-04-11

**Authors:** Hong-Jay Lo, Ravinder Mettu, Chiang-Yun Chen, Shiou-Ting Li, Chung-Yi Wu

**Affiliations:** Genomics Research Center, Academia Sinica, 128 Academia Road, Section 2, Nankang, Taipei 115, Taiwan

## Abstract

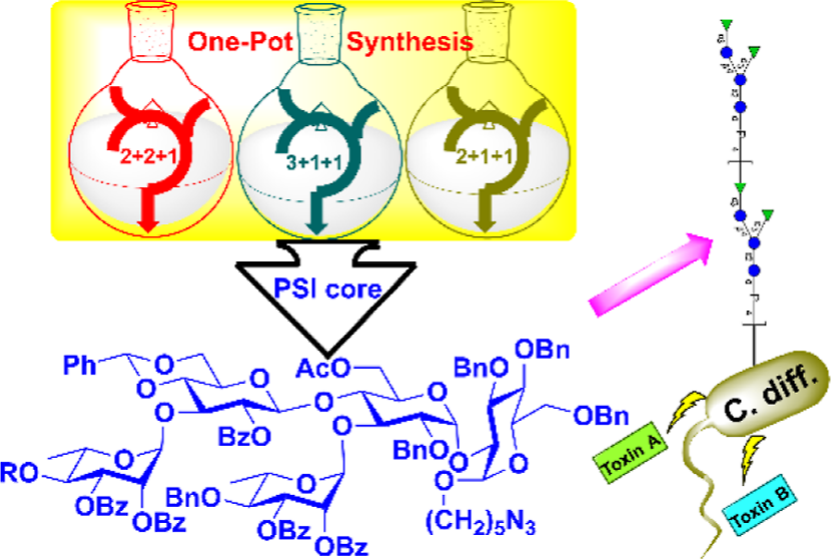

*Clostridium difficile* causes serious nosocomial diarrhea in humans. In particular, *C. difficile* ribotype 027 strain, which is more virulent in nature, is associated with higher mortality rates. To prevent disease caused by *C. difficile*, capsular polysaccharides such as PSI, PSII, and PSIII from the outermost protective layer of *C. difficile* bacterium are good targets in the development of vaccine against *C. difficile*. In this study, we report for the first time an efficient synthesis of phosphorylated PSI oligosaccharide and its derivatives in various lengths. We have also developed a one-pot strategy for the synthesis of a pentasaccharide repeating unit and used this approach to successfully synthesize the protected pentasaccharides in the gram scale.

## Introduction

*Clostridium difficile* is a Gram-positive, spore-forming, gastrointestinal bacterium that generally causes serious nosocomial diarrhea in humans and animals.

The composition of the intestinal bacteria may alter for the patients who take antibiotics.^1^ The dis-regulation of intestinal homeostasis may result in the massive growth of antibiotics-resistant *C. difficile* that may become pathogenic and cause *C. difficile* infection (CDI).^2^ CDI symptoms include mild diarrhea, abdominal pain, fever, sepsis, and shock.^3^ Alarmingly, occurrences of CDI have increased worldwide.^4−7^ From 2002 to 2015, the annual CDI-related medical cost in the US increased from 1.1 billion to about 6.3 billion USD.^8,9^ Also, the average CDI-attributable costs per case is 21,448 USD in 2015 in the United States. Specifically, the higher mortality rates are associated with *C. difficile* ribotype 027 strain, which is reported as hypervirulent.^10^ The hypervirulent *C. difficile* is too dangerous and costly without an anti-*C. difficile* vaccine.

**Figure 1:**
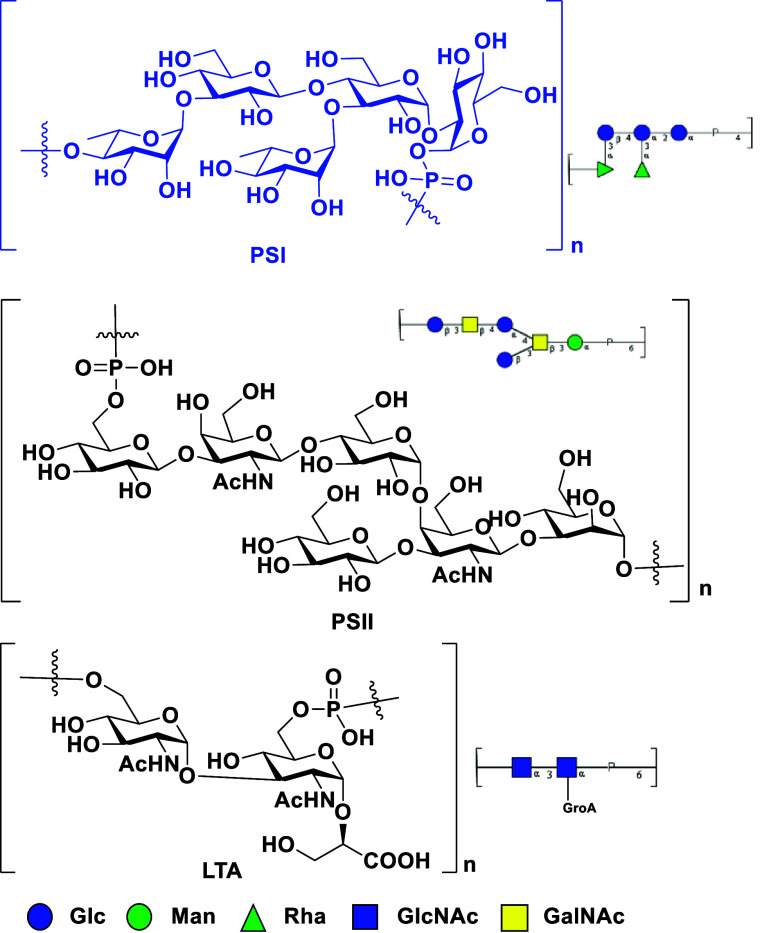
Structure of PSI, PSII, and lipoteichoic acid (LTA).

The virulence factor of *C. difficile* is primarily attributed to toxin A (TcdA) and toxin B (TcdB).^11^ Although a passive immunization of antitoxin neutralizing antibodies reduced the recurrence of *CDI*, the toxin-based vaccine may not inhibit the colonization of *C. difficile*, resulting in the growth of *C. difficile* instead.^12^ Hence, as an alternative to targeting the secreted toxin, the cell surface polysaccharide has arisen as an ideal target for vaccine candidates. Successful examples were demonstrated by conjugating a capsular polysaccharide with carrier protein CRM197 as in *Haemophilus influenzae* type b (Hib), pneumococcal, and meningococcal conjugate vaccines, which were approved and have been widely used.^13,14^ This type of carrier protein-conjugated polysaccharide can induce T cell-dependent immune response and specific antibodies against polysaccharide antigens. Three surface glycans, PSI, PSII, and lipoteichoic acid, were identified on *C. difficile* (Figure 1).^15,16^ Although conjugation of these glycans on a carrier protein as vaccine candidates is immunogenic, isolation of these glycans from *C. difficile* is a challenge due to their low expression level.^15,16^ Chemical synthesis is an alternative strategy to obtain the surface glycans on *C. difficile* for vaccine candidates. PSII has been synthesized and used as a vaccine candidate by Adamo et. al.,^17^ and their results identified the terminal phosphate group as a key epitope to induce specific antibodies. On the other hand, PSI that consists of a pentsaccharide repeating unit including [→4)-α-Rha-(1→3)-β-Glc-(1→4)-[α-Rha-(1→3]-α-Glc-(1→2)-α-Glc-(1→P] has been synthesized for vaccination studies by the Seeberger group and Mario groups independently.^18−21^ The Mario group indicated the native phosphorylated PSI resulted in higher binding titer against the tested horse serum IgG antibodies than the synthetic nonphosphorylated PSI did, suggesting that glycosyl phosphate and polymeric nature of PSI are essential for immunogenicity. However, in their synthesis, the phosphate group was incorporated at the terminal end of glycan, which is different from the internal phosphate group within the native phosphorylated glycan. Since lengths of PSI and locations of phosphate group may change the immunogenicity result.^22^ it is essential to obtain glycans of various structures for a systemic structure–activity relationship study for the development of *C. difficile* vaccine.

Glycosidation of glycosyl donor couples with the OH group of phosphate ester bearing in the glycosyl acceptor is challenging and has not been achieved as far as we know. In this study, we systemically synthesized PSI oligosaccharides ranging from disaccharide to decasaccharide with or without terminal phosphate group and internal phosphate diester for immunogenicity studies. We further developed a one-pot strategy for the synthesis of PSI in gram-scale; the yield and efficiency are far more superior to any existing strategies.

## Results and Discussion

**Scheme 1:**
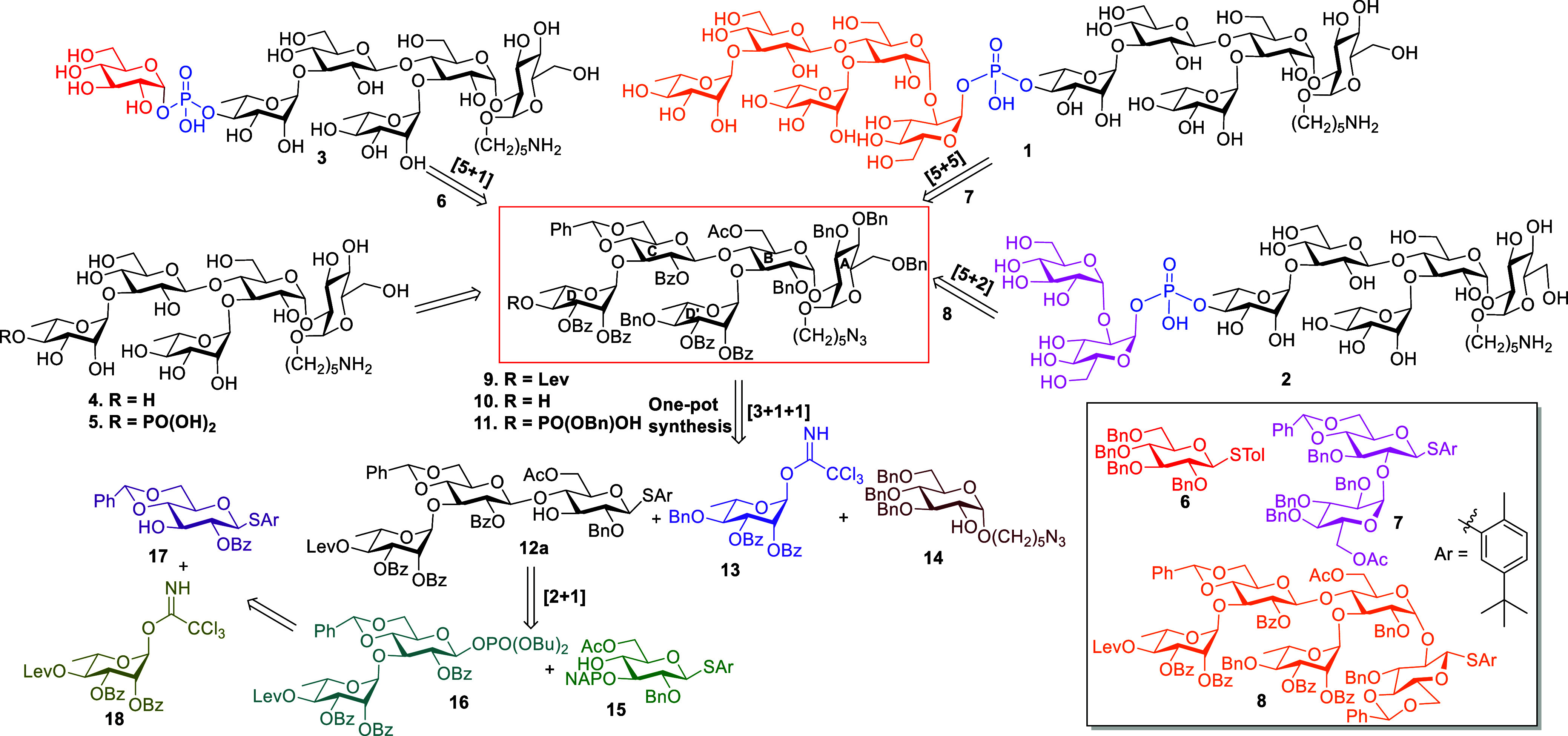
Retrosynthetic Analysis of the Targeted PS-I Oligosaccharides 1-5

The major objective of this study is to synthesize PSI oligosaccharide in various chain lengths with or without a phosphate group for immunogenicity and antigenicity studies. All the target compounds were designed to have an *O*-linked aminopentyl spacer at the reducing end, which is essential for the conjugation of carrier protein to construct the conjugated vaccine against *C. difficile*. Based on our retrosynthetic analysis (Scheme 1), the phosphodiester bridge in target compounds **1–3** can be established through [5 + 5], [5 + 2], and [5 + 1] glycosylation approach by treating the common key phosphorylated acceptor **11** with donor compounds **6**–**8**, respectively. Complete removal of protecting groups of compounds **10** and **11** yields other target compounds **4** and **5**, respectively. The pentasaccharide **9** can be prepared in one pot via [3 + 1 + 1] glycosylation of **12a** with donor **13** followed by the treatment of acceptor **14**. The trisaccharide **12a** can be assembled through glycosylation of corresponding building blocks **15** and **16**.

**Scheme 2:**
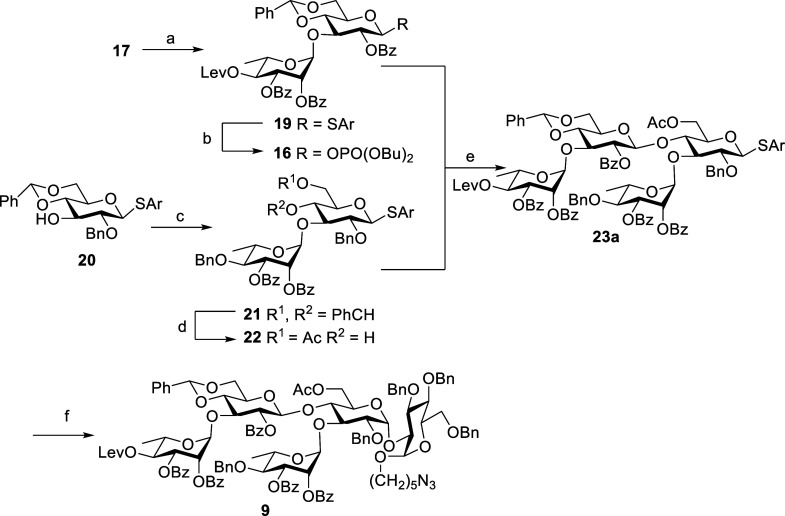
Synthesis of Pentasaccharide 9 by a [2 + 2 + 1] Strategy Reagents and conditions: **18**, TMSOTf, CH_2_Cl_2_, MS 4 Å, −30 °C, 1 h, 87%. HOPO(OBu)_2_, NIS, TfOH, CH_2_Cl_2_, MS 4 Å, 0 °C, 16 h, 92%. **13**, TMSOTf, CH_2_Cl_2_, MS 4 Å, −30 °C, 1 h, 99%. 4% HCl_(aq)_, MeOH, 40 °C 20 h, then Ac_2_O, Et_3_N, CH_2_Cl_2_, 0 °C, 1 h, 73%. **22**, TMSOTf, CH_2_Cl_2_, MS 4 Å, −30 °C, 3 h, 43% (55% b.r.s.m.). **14**, NIS, TfOH, CH_2_Cl_2_, MS 4 Å, −30 °C, 1 h, 69%, Lev = levulinoyl, Ar = 2-methyl-5-*tert*-butylthiophenyl, TMSOTf = trimethylsilyl trifluoromethanesulfonate, b.r.s.m. = based on the recovery of starting materials. NIS = *N*-iodosuccinimide.

Hence, our synthetic strategy began with the preparation of building blocks **6**, **7**, **13–15**, **17**, and **18** in multigram quantities, and their synthesis is discussed in detail in the Supporting Information. A special attention was paid to protecting groups in designing the building blocks. Rhamnose building block **18** was designed to have Lev group at the C-4 position, which could be removed selectively in the presence of other ester groups at the time of phosphorylation. To achieve more alpha selectivity at the stage of glucose linker attachment, we designed the glucose building block **15** to have acetyl groups at C-6^23^ and naphthyl group at C-3 for temporary protection. After obtaining all the building blocks, we focused on accessing PSI repeating oligosaccharide **9** through [2 + 2 + 1] glycosylation strategy. The TMSOTf-mediated glycosidation of rhamnosyl donor **18** with acceptor **17** and the reaction proceeded smoothly to give corresponding α-linked disaccharide **19** in 87% yield (^1^*J*_CH_ = 173.6 Hz); then, **19** was treated with dibutyl phosphate under the activation of the NIS-TfOH system to further convert it to disaccharide phosphate donor **16** (Scheme 2). On the other hand, the disaccharide **21** was assembled by the glycosidation of rhamnosyl donor **13** with acceptor **20** (see the Supporting Information) in the presence of TMSOTf in good yield 99%. The newly formed glycosidic linkage was confirmed to be α-bond based on its anomeric C–H coupling constant value (^1^*J*_CH_ = 176.1 Hz). Thereafter, the benzylidene ring was removed by 4% HCl, and selective acetylation at 6-OH with Ac_2_O/NEt_3_ was carried out in one pot to afford the acceptor **22** in 73% yield (Scheme 2).

Tetrasaccharide **23a** was constructed through [2 + 2] coupling of disaccharide donor **16** with disaccharide acceptor **22** in the presence of TMSOTf at −30 °C, affording compound **23a** in 43% yield. Finally, the fully protected target compound **9** was accomplished in 69% yield by glycosidation of donor **23a** with acceptor **14** in the presence of NIS-TfOH at −30 °C (Scheme 2). The newly formed glycosidic linkage in compound **9** was characterized to be α-bond based on its coupling constant values (^1^*J*_CH_ = 168.7 Hz and ^3^*J*_H–H_ = 3.6 Hz).

**Scheme 3:**
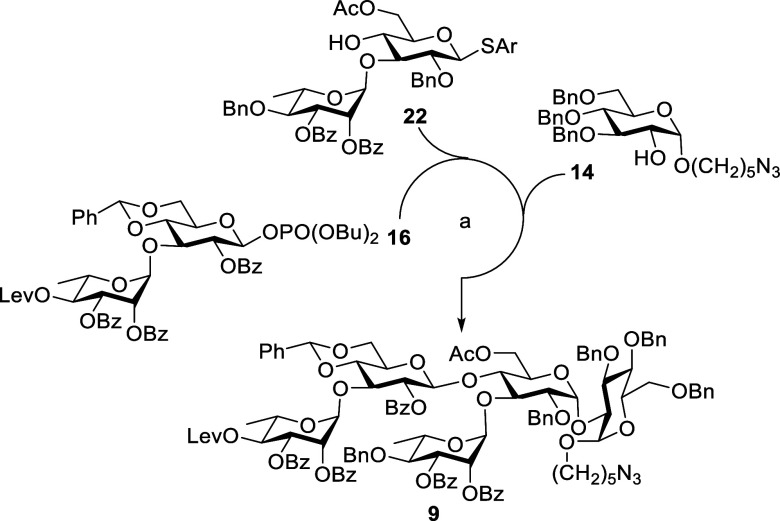
One-Pot Synthesis of Pentasaccharide **9** via the [2 + 2 + 1] Strategy Reagents and conditions: **22**, TMSOTf, CH_2_Cl_2_, MS 4 Å, −30 °C, 3 h, then **14**, NIS, TfOH, CH_2_Cl_2_, MS 4 Å, −30 °C, 1 h, 20%.

After stepwise synthesis of pentasaccharide **9**, we focused on devising a one-pot^24^ synthesis of **9** using same reaction conditions, as shown in the Scheme 3. Glycosidation of phosphate donor **16** with thioglycoside acceptor **22** by activation of TMSOTf in CH_2_Cl_2_ at −30 °C provided tetrasaccharide **23a** after 3 h; then, acceptor **14** and NIS were added sequentially to the reaction flask, and the reaction was finished in 1 h to afford protected pentasaccharide in overall yield of 20% after purification.

**Scheme 4:**
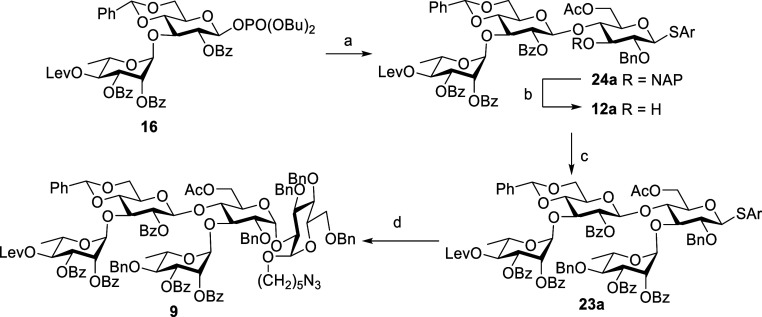
Synthesis of Pentasaccharide **9** by a [3 + 1 + 1] Strategy Reagents and conditions: **15**, TMSOTf, CH_2_Cl_2_, MS 4 Å, −40 °C, 30 min, 98%. DDQ, CH_2_Cl_2_, phosphate buffer pH 7, 0 °C to rt, 3 h, 92%. **13**, TMSOTf, CH_2_Cl_2_, MS 4 Å, −30 °C, 30 min, 91%. **14**, NIS, TfOH, CH_2_Cl_2_, MS 4 Å, −30 °C, 1 h, 69%, NAP = 2-naphthylmethyl, DDQ = 2,3-dichloro-5,6-dicyano-*p*-benzoquinone.

**Scheme 5:**
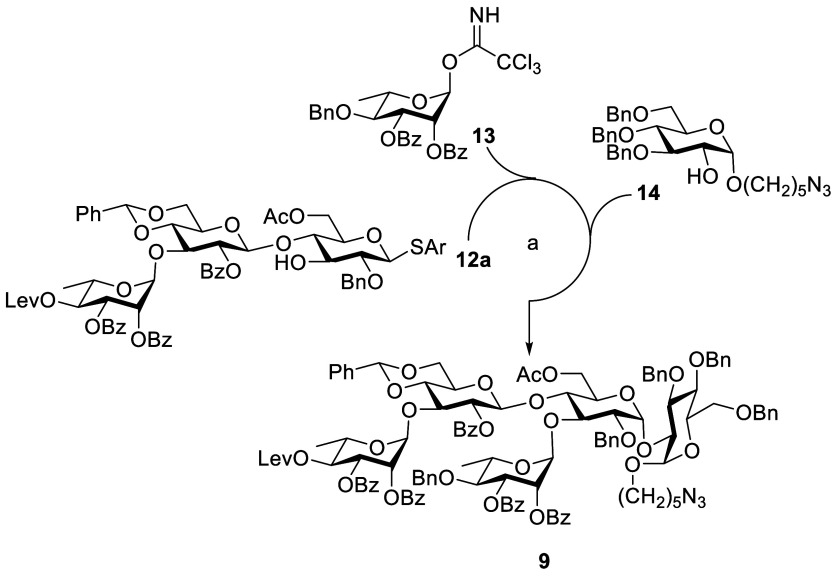
[3 + 1 + 1] One-Pot Synthesis of Pentasaccharide **9** Reagents and conditions: **13**, TMSOTf, CH_2_Cl_2_, MS 4 Å, −30 °C, 30 min, then **14**, NIS, TfOH, CH_2_Cl_2_, MS 4 Å, −30 °C, 1 h, 67%.

Overall, the [2 + 2 + 1] coupling strategy in stepwise or in one-pot synthesis only provided the pentasaccharide **9** in low yield. Moreover, an excess disaccharide donor was required to proceed the reaction and also the purification of tetrasaccharide was difficult. To overcome the process difficulties and to acquire better yield, we decided to examine various alternative synthetic approaches for synthesizing pentasaccharide **9**. The first alternative synthetic approach we used is the [3 + 1 + 1] coupling strategy (Schemes 4 and 5).

The stepwise [3 + 1 + 1] strategy was carried out by TMSOTf-mediated coupling of donor **16** with acceptor **15** in CH_2_Cl_2_ at −40 °C to afford the trisaccharide **24a** (Scheme 4). Fortunately, this reaction smoothly completed in 30 min and afforded the only β-anomer compound in 98% yield. Oxidative cleavage of NAP ether by DDQ gave alcohol **12a** in 92%. Glycosidation of **12a** with donor **13** in the presence of promoter TMSOTf provided tetrasaccharide **23a** in 91% yield. The resulted tetrasaccharide **23a** upon glycosylation with acceptor **14** provided the fully protected pentasaccharide **9** (see Scheme 1). Additionally, pentasaccharide **9** can also be synthesized by sequential one-pot [3 + 1 + 1] glycosylation, in which the trisaccharide acceptor **12a** was treated with donor **13** and, then, acceptor **14** (Scheme 5). After careful tuning of the [3 + 1 + 1] one-pot strategy, we successfully synthesized compound **9** in gram-scale in overall yield of 67%, which is much higher than the previous [2 + 2 + 1] one-pot strategy.

**Scheme 6:**
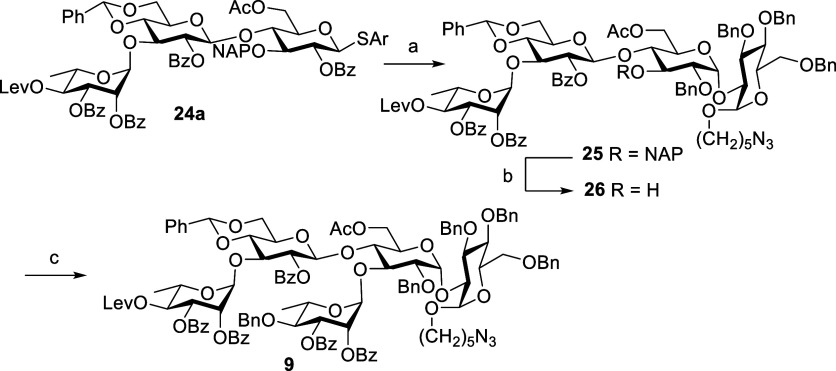
Synthesis of Pentasaccharide **9** by a [3 + 1 + 1] Strategy Reagents and conditions: **14**, NIS, TfOH, CH_2_Cl_2_, Et_2_O, MS 4 Å, −30 °C, 1 h, 25α 73%, 25β 2.5%. DDQ, CH_2_Cl_2_, phosphate buffer pH 7, 0 °C to rt, 3 h, 88%. **13**, TMSOTf, CH_2_Cl_2_, MS 4 Å, −30 °C, 40 min, 96%.

The other alternative synthetic approach we have examined for the synthesis of pentasaccharide **9** is making ABCD tetrasacccharide core unit followed by attachment of rhamnose D′ (Scheme 6).

**Scheme 7:**
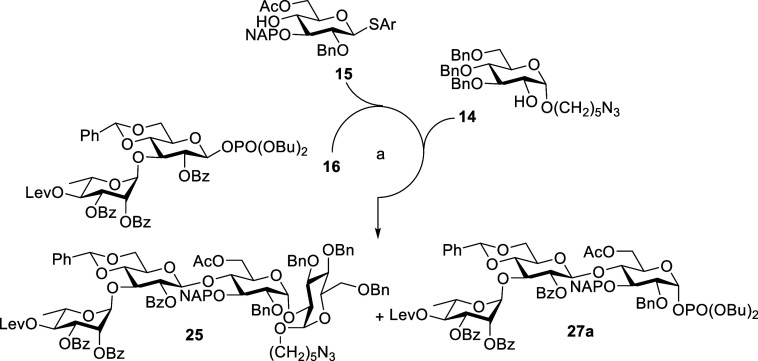
One-Pot Synthesis of Tetrasaccharide **26** via a [2 + 1 + 1] Strategy Reagents and conditions: **15**, TMSOTf, CH_2_Cl_2_, MS 4 Å, −40 °C, 30 min, then **14**, NIS, TfOH, CH_2_Cl_2_, MS 4 Å, −30 °C, 1 h, **25**: 56%, **27a**: 23%.

Glycosidation of compound **24a** with **14** using NIS-TfOH as promoters in a mixture of CH_2_Cl_2_/Et_2_O (1:1) at −30 °C provided **25α** in 73% and 25β in 2.5% yields after the column purification. The NAP ether was removed by oxidative cleavage with DDQ in a mixture of CH_2_Cl_2_ and neutral phosphate buffer solution, and the resulted alcohol compound **26** was subsequently glycosylated with donor **13** in the presence of TMSOTf in CH_2_Cl_2_ at −30 °C to afford the desired pentasaccharide **9** in a 96% yield (Scheme 6). Overall, this strategy had more advantages over the previous described coupling strategies in all aspects. We also examined the synthesis of the ABCD tetrasaccharide core of PSI in a one-pot through [2 + 1 + 1] coupling strategy. Sequential addition of the acceptors **15** (1 equiv) and **14** (1.2 equiv) to the disaccharide donor **16** in CH_2_Cl_2_ at −40 and −30 °C, respectively, afforded tetrasaccharide **25** in overall yield of 56% in one-pot (Scheme 7). We also observed the formation of trisaccharide phosphate **27a** in 23% yield through anomeric phosphorylation of thioglycoside 24a that formed initially in the one-pot synthesis (Scheme 7).^25^

**Scheme 8:**
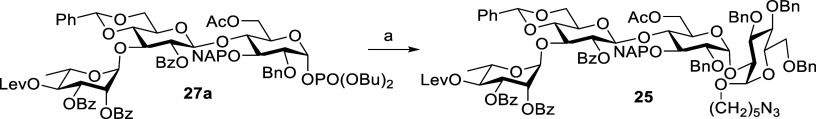
Synthesis of Tetrasaccharide **26** from Compound **27a** Reagents and conditions: **14**, NIS, TMSOTf, CH_2_Cl_2_, MS 4 Å, −30 °C, 1 h, 77%.

Favorably, the aglycon transfer compound **27a** can be used to produce tetrasaccharide linker compound **25** by treating with acceptor **14** (Scheme 8).

**Scheme 9:**
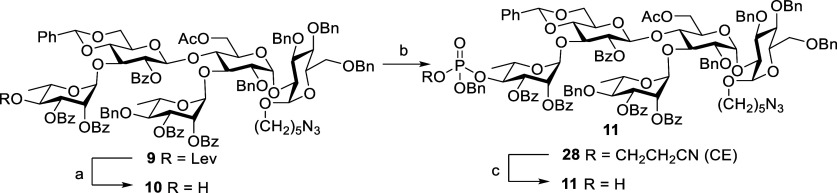
Synthesis of Pentasaccharide **11** Reagents and conditions: hydrazine hydrate, pyridine, AcOH, CH_2_Cl_2_, rt, 1 h, 93%. Benzyl 2-cyanoethyl *N*,*N*-diisopropylphosphoramidite, 1*H*-tetrazole, CH_2_Cl_2_, rt, 40 min, then *m*CPBA, −20 °C, 20 min, 99%. TBAOH, CH_2_Cl_2_, H_2_O, rt, 4 h, 94%.

After the successful synthesis of compound **9**, we focused on phosphorylation at C4–OH of the rhamnose D unit. To introduce the phosphate group, first, hydrazine hydrate in pyridine and AcOH were used to remove the Lev group of compound **9** to give alcohol **10**, which was then phosphorylated with freshly prepared benzyl 2-cyanoethyl *N*,*N*-diisopropyl phosphoramidite^26^ in the presence of 1*H*-tetrazole and oxidized with *m*CPBA to provide phosphotriester **28** as a diasteromeric mixture in 99% yield. Treatment of **28** with tetrabutylammonium hydroxide (TBAOH) in CH_2_Cl_2_/H_2_O system afforded **11** in 94% yield (Scheme 9).

**Scheme 10:**
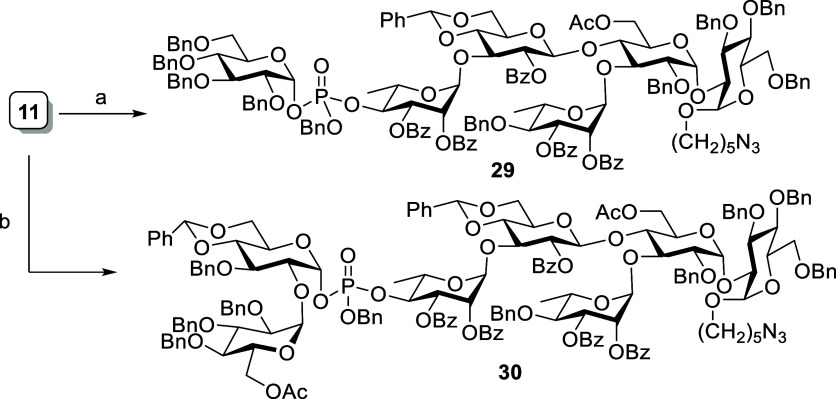
Synthesis of Hexasaccharide **29** and Heptasaccharide **30** Reagents and conditions: **6**, NIS, TfOH, CH_2_Cl_2_, MS 4 Å, 0 °C, 16 h, 70%. **7**, NIS, TfOH, CH_2_Cl_2_, MS 4 Å, rt, 20 h, 75%.

Recent vaccination studies reveal that the length of the oligosaccharide chain also plays an important role in the antigenicity and immunogenicity outcomes.^27^ Therefore, to identify the minimal structural entity for vaccine development against *C. difficile*, it is necessary to synthesize various lengths of PSI oligosaccharides. Hence, we switched our focus to chain elongation of the phosphorylated derivative. The phosphodiester bridge in compound **29** was established upon glycosidation of the donor **6**(28) with acceptor **11** in the presence of a NIS-TfOH system in CH_2_Cl_2_ at 0 °C to afford desired hexasaccharide compound **29** in 70% yield and mostly α-isomer (^1^*J*_CH_ = 170.6 Hz, α/β = 80:1) (Scheme 10). Furthermore, disaccharide donor **7** and acceptor **11** under the above-described reaction conditions gave heptasaccharide **30** in 75% yield and in a single α-isomer (Scheme 10).

**Scheme 11:**
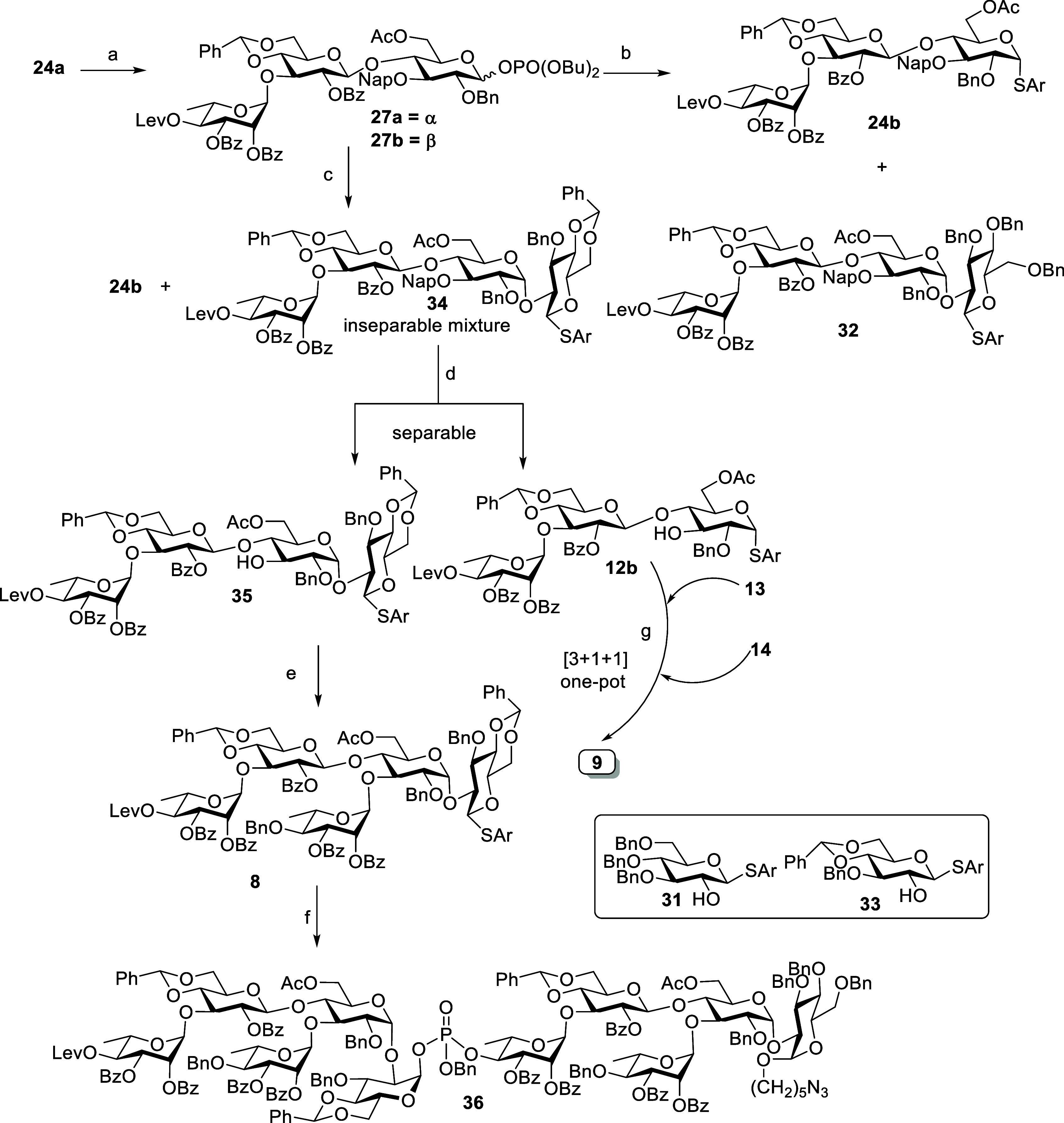
Synthesis of Decasaccharide **36** Reagents and conditions: HOPO(OBu)_2_, NIS, TfOH, CH_2_Cl_2_, MS 4 Å, 0 °C, 16 h, 91% (α/β = 1:3). **31**, TMSOTf, CH_2_Cl_2_, MS 4 Å, −40 to −20 °C, 1 h, **32**: 0%, **24b**: 84%. **33**, TMSOTf, CH_2_Cl_2_, MS 4 Å, −40 to −20 °C, 1 h. DDQ, CH_2_Cl_2_, phosphate buffer pH 7, 0 °C to rt, 3 h, **35**: 36%, **12b**: 34% (two steps). **13**, TMSOTf, CH_2_Cl_2_, MS 4 Å, −30 °C, 40 min, 98%. **11**, NIS, TfOH, CH_2_Cl_2_, MS 4 Å, rt, 40 h, 81%. **13**, TMSOTf, CH_2_Cl_2_, MS 4 Å, −30 °C, 30 min, then **14**, NIS, TfOH, CH_2_Cl_2_, MS 4 Å, −30 °C, 1 h, 40%.

Next, we focused on the synthesis of decasaccharide, which contains two pentasaccharide repeating units that link with phospodiester bonds. The synthesis of pentasaccharide donor building block **8** is depicted in Scheme 11. The reaction between thioglycoside **24a** and dibutyl phosphate in the presence of NIS-TfOH provided α,β-mixture of glycosyl phosphate donor **27a,b** in good yield (91%, α/β = 1:3). Later, it was treated with acceptor **31** using TMSOTf as a promoter to try to synthesize tetrasaccharide **32**. Unfortunately, our attempt failed to produce the desired tetrasaccharide **32** and, instead, only provided aglycon transferred trisaccharide **24b**. Moreover, glycosidation of phosphate donor **27a,b** with acceptor **33** afforded inseparable tetrasaccharide **34** and **24b** in a 1:1 ratio. Similar results were observed when the same reaction was treated with TBSOTf as a promoter (Scheme 11).

Interestingly, these inseparable compounds **34** and **24b** were easily separated after oxidative cleavage of their NAP ether by DDQ treatment (over two steps) to give tetrasaccharide acceptor **35** and trisaccharide acceptor **12b** in 36% and 34% yield, respectively. The tetrasaccharide acceptor **35** upon glycosidation with donor **13** in the presence of TMSOTf provided the pentasaccharide **8** in good yield; then, pentasaccharide **8** was used as a donor in the decasaccharide synthesis. The alpha selective phosphoester bond between the donor **8** and phosphorylated acceptor **11** was established in the presence of NIS-TfOH to give decasaccharide in 81% yield (Scheme 11). The newly formed glycosidic bond in compound **36** was characterized to be α based on its anomeric CH coupling constant value (^1^*J*_CH_ = 180.7 Hz). The trisaccharide acceptor **12b**, which was obtained by aglycon transfer, was successfully utilized in the synthesis of pentasaccahride through a [3 + 1 + 1] glycosylation method (Scheme 11).

**Scheme 12:**
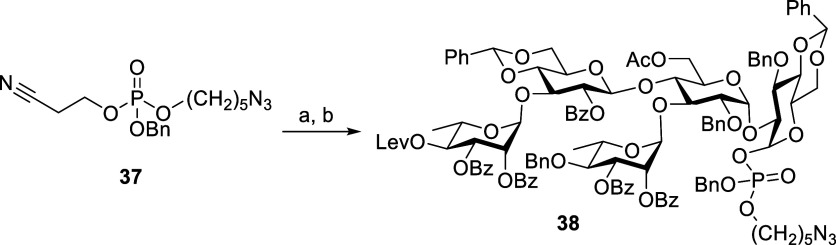
Synthesis of Pentasaccharide **38** Reagents and conditions: TBAOH, CH_2_Cl_2_, H_2_O, 4 h. **8**, NIS, TfOH, CH_2_Cl_2_, MS 4 Å, rt, 20 h, 41% (two steps).

To understand the role and identify the active position of the phosphate group on PSI oligosaccharide in immunogenicity, it is necessary to synthesize the oligosaccharides with the phosphate group at different positions. We have synthesized oligosaccharides with a phosphate group in the middle **29**, **30**, and **36** and at nonreducing end **11**. Next, we switched our focus to synthesize oligosaccharide with a phosphate group at the reducing end. Pentasaccharide **8** was treated with readily prepared phosphate reagent **37** (see the Supporting Information) in the NIS-TfOH system to afford alpha linked phosphate diastereomeric compounds **38** in 41% yield (Scheme 12).

**Scheme 13:**
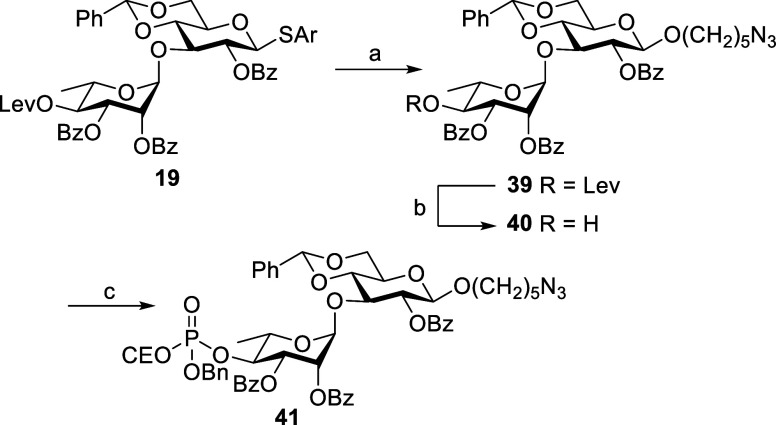
Synthesis of Disaccharide **41** Reagents and conditions: HO(CH_2_)_5_N_3_, NIS, TfOH, CH_2_Cl_2_, MS 4 Å, 0 °C, 16 h, 55% (69% b.r.s.m.). Hydrazine hydrate, pyridine, AcOH, CH_2_Cl_2_, rt, 1 h, 94%. Benzyl 2-cyanoethyl *N*,*N*-diisopropylphosphoramidite, 1*H*-tetrazole, CH_2_Cl_2_, rt, 40 min, then *m*CPBA, −20 °C, 20 min, 93%.

**Scheme 14:**
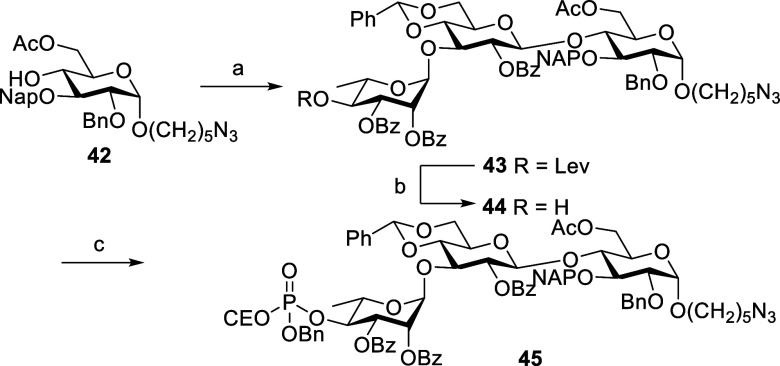
Synthesis of Trisaccharide **45** Reagents and conditions: **16**, TMSOTf, CH_2_Cl_2_, MS 4 Å, −40 °C, 1 h, 99%. Hydrazine hydrate, pyridine, AcOH, CH_2_Cl_2_, rt, 1 h, 95%. Benzyl 2-cyanoethyl *N*,*N*-diisopropylphosphoramidite, 1*H*-tetrazole, CH_2_Cl_2_, rt, 40 min, then *m*CPBA, −20 °C, 20 min, 95%.

**Scheme 15:**
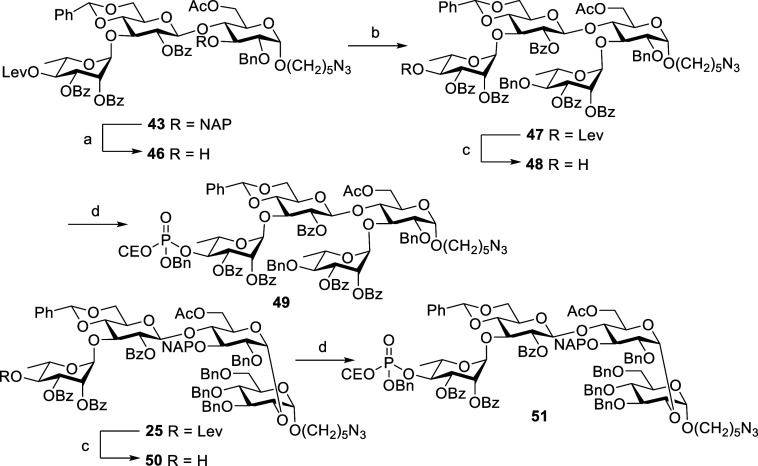
Synthesis of Tetraasaccharid **49** and **51** Reagents and conditions: DDQ, CH_2_Cl_2_, phosphate buffer pH7, 0 °C to rt, 3 h, 95%. **13**, TMSOTf, CH_2_Cl_2_, MS 4Å, −30 °C, 1 h, 93%. Hydrazine hydrate, pyridine, AcOH, CH_2_Cl_2_, rt, 1 h, **48**: 97%, **50**: 93%. Benzyl 2-cyanoethyl *N*,*N*-diisopropylphosphoramidite, 1*H*-tetrazole, CH_2_Cl_2_, rt, 40 min, then *m*-CPBA, −20 °C, 20 min, **49**: 97%, **51**: 93%.

**Scheme 16:**
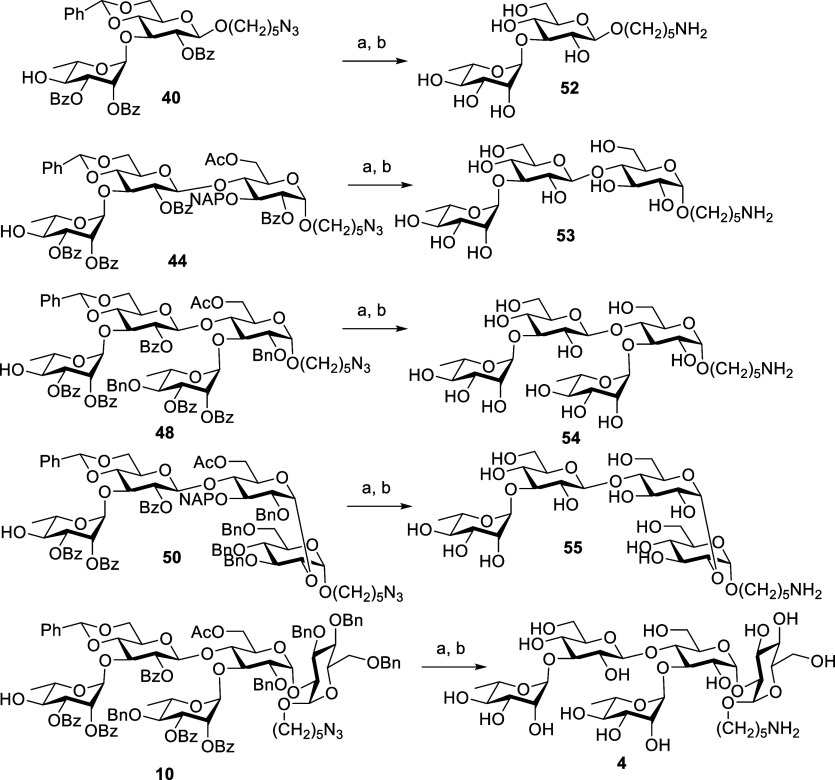
Global Deprotection of Non-phosphorylated Di, Tri, Tetra, and Pentasaccharides Reagents and conditions: NaOMe, MeOH, CH_2_Cl_2_, **40**: rt, 2 h, **44**: rt, 1 h, **48**: rt, 24 h; **50**: rt, 1 h, **10**: 50 °C, 16 h. Pd(OH)_2_, H_2_, MeOH, H_2_O, AcOH, 40 h, **52**: 79%, **53**: 75%, **54**: 82%, **55**: 94%, **4**: 68% (two steps).

To identify the minimal glycan epitope for the development of vaccine and biomarkers against *C. difficile*, it is necessary to synthesize various lengths of PSI glycans for the study. Previous studies showed the lengths of the glycans also play an important role in immune response. In some cases, glycans with shorter lengths could still induce immunity. Therefore, we synthesized shorter PSI glycans ranging from disaccharide to tetrasaccharide with and without phosphate groups in addition to our main target compounds for a complete vaccination study. The syntheses of those compounds are described in Schemes 13–16.

The synthesis of protected disaccharide phosphate **41** was achieved upon glycosidation of donor **19** with 1-azido pentanol acceptor in the presence of NIS-TfOH, followed by the replacement of Lev group on rhamnose with phosphate and then oxidation with *m*CPBA (Scheme 13).

Protected trisaccharide phosphate **45** was synthesized, as shown in Scheme 14. Glycosidation between donor **16** and acceptor **42** (see Supporting Information) in the presence of TMSOTf at −40 °C resulted the trisaccharide **43** in 99% yield. To introduce the phosphate group on rhamnose, hydrazine hydrate in pyridine and AcOH were used to remove the Lev group to give alcohol **44**, which was phosphorylated with benzyl 2-cyanoethyl *N*,*N*-diisopropylphosphoramidite and oxidized with *m*CPBA to give phosphorylated derivative **45** in 95% yield (Scheme 14).

Next, we focused on synthesis of tetrasaccharide phosphate derivatives **49** and **51** (Scheme 15). DDQ-mediated oxidative cleavage of NAP ether of trisaccharide **43** afforded alcohol compound **46** in 95% yield; then, glycosylation of **46** with rhamnosyl donor **13** in the presence of TMSOTf at −30 °C provided **47** in 93% yield. Cleavage of the Lev group of **47** followed by phosphorylation and oxidation provided tetrasaccharide phosphate **49**. Tetrasaccharide phosphate **51** was prepared by removing the Lev group of previously prepared compound **25** followed by employing standard phosphorylation and oxidation protocols, as shown in Scheme 15.

The nonphosphorylated compounds **10**, **40**, **44**, **48**, and **50** were deprotected in a two-step process, in which all the ester groups were removed with NaOMe in methanol. Next, all benzyl and benzylidene groups were removed, and the azide group was reduced to amine in one step by Pd/C catalyzed hydorgenolysis in MeOH/H_2_O/AcOH to give pure final compounds **4** and **52–55** after LH-20 purification (Scheme 16).

**Scheme 17:**
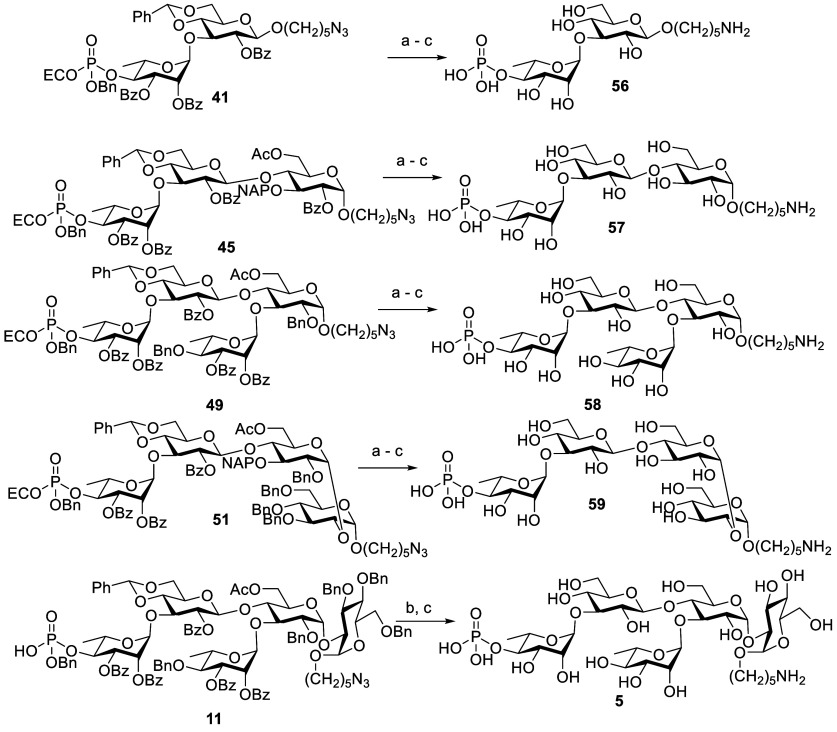
Global Deprotection of Phosphorylated Di, Tri, Tetra, and Pentasaccharides Reagents and conditions: TBAOH, CH_2_Cl_2_, H_2_O, rt, 4 h. NaOMe, MeOH, CH_2_Cl_2_, rt, 1 h. Pd(OH)_2_, H_2_, MeOH, H_2_O, AcOH, 40 h, **56**: 77%, **57**: 72%, **58**: 75%, **59**: 65% (three steps), **5**: 82% (two steps).

Global deprotection of phosphorylated oligosaccharides **28**, **41**, **45**, **49**, and **51** proceeded in a three- or two-steps process, in which the 2-cyanoethyl was cleaved using tetrabutyl ammonium hydroxide. Subsequently, all ester groups were removed with NaOMe in methanol. Next, benzyl and benzylidene groups were removed by Pd/C catalyzed hydrogenolysis in MeOH/H_2_O/AcOH, and the azide group was reduced into amine to provide the pure target compounds **5** and **56**–**59** after LH-20 purification (Scheme 17).

**Scheme 18:**
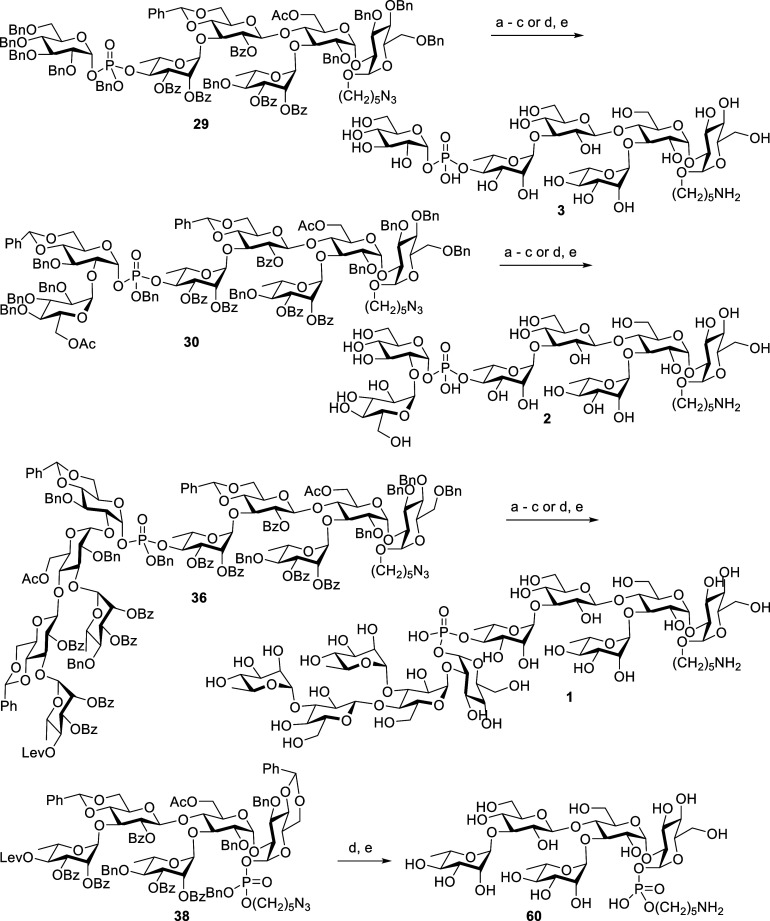
Global Deprotection of Phosphate Bridged Compounds **29**, **30**, **36,** and **38** Reagents and conditions: NaI, acetonitrile, 90 °C, 24 h. NaOMe, MeOH, CH_2_Cl_2_, rt, **29**: 16 h, **30**: 24 h, **36**: 40 h. Pd(OH)_2_, H_2_, MeOH, H_2_O, AcOH, **3**: 40 h, ratio α/β = 6:1 after purify 34% (α/β = 12:1), **2**: 72 h, ratio α/β = 9:1 after purify 28% (α/β = 20:1), **1**: 72 h, ratio α/β = 5:1 after purify 28% (α only) (three steps). Li, NH_3(l)_, THF, −78 °C 1.5 h. NaOMe, MeOH, 16 h, **3**: 49%, **2**: 34%, **1**: 24%, **60**: 46% (two steps).

Global deprotection of phosphate bridged compounds **29**, **30**, **36,** and **38** are challenging as the phosphate is bonded to anomeric position, which is sometimes unstable in reaction conditions. Accordingly, we proceeded in a three-step process in which the benzyl ether group on phosphate was removed with NaI in AcCN. Subsequently, all ester groups were removed by saponification with NaOMe in methanol and CH_2_Cl_2_. Finally, all benzyl ether and benzylidene groups were removed, and the azide group was reduced into amine by Pd/C catalyzed hydrogenolysis in MeOH/H_2_O/AcOH. The obtained oligosaccharides were subjected to Sephadex LH-20 column chromatography using distilled water as an eluent. After careful NMR analysis,^29^ it was found that anomerization was taking place in all the compounds at glucose attached phosphate at the glucose C1 position during the deprotection process, and the final compounds were mixtures of α, β-linkages. However, those compounds were somewhat separable by RP-18 column chromatography (see the Supporting Information). We reasoned that the acidic nature of the reaction medium favored anomerization.^30,31^ Nevertheless, breakthrough results were achieved when applying Birch reduction using Li/liq NH_3_ removed all functional groups except benzoyl group, and reduction of the azide group into amine took place in one pot. The obtained residues were treated with NaOMe in methanol to afford the title compounds in 24–49% yields after performing LH-20 and RP-18 column purifications (Scheme 18).

## Conclusions

In this study, we synthesize various PSI oligosaccharides with various chain lengths with or without the phosphate group for the immunogenicity studies of PSI carbohydrate antigens. The pentasaccharide acceptor was generated in three different optimized processes, including [2 + 2 + 1] one-pot synthesis with 20% yield (Scheme 3), [3 + 1 + 1] one-pot synthesis with 67% yield (Scheme 5), and another [3 + 1 + 1] synthesis with 62% yield (Scheme 6). Then, the phosphorylated acceptor was generated from the pentasaccharide. Following with [5 + 5], [5 + 2], and [5 + 1] glycosylation and global deprotection, the target compound **1–3** were obtained.

## Experimental Section

All reactions were carried out under an inert atmosphere unless mentioned otherwise, and standard syringe–septa techniques were employed. Solvents were purchased from Acros, Echo chemical, Merck, J. T. Baker Sigma-Aldrich, and Fluka and used without further purification. Pulverized Molecule Sieve 4 Å (Acros) was dried with a heater under high vacuum. In our study, silicone oil baths are used as a heating source for chemical reaction. The progress of all reactions were monitored by TLC; TLC glass plates precoated with silica gel 60 F254 (Merck) were used, and TLC was detected by UV light (254 nm), *p*-anisaldehyde, or ceric ammonium molybdate. Column chromatography was performed on silica gel Geduran Si 60 (40–63 μm, Merck). ^1^H, ^13^C, and ^31^P NMR spectra were recorded with Bruker AVANCE 600 (600 MHz) or Bruker AV-III 400 (400 MHz) spectrometer at 25 °C spectrometers, and chemical shifts were measured in δ (ppm) with residual solvent peaks as internal standards (CDCl_3_, δ 7.24 ppm, D_2_O, δ 4.80 ppm in ^1^H NMR and CDCl_3_, δ 77 ppm, in ^13^C NMR). Coupling constants *J* are measured in Hz. Data are represented in chemical shift, multiplicity (s = singlet, d = doublet, t = triplet, q = quartet, m = multiplet, and br = broad). Structural assignments were made with additional information from gCOSY, gHSQC, and gHMBC experiments. HR MALDI-TOF (LR MALDI-TOF) mass spectra were recorded on a Bruker Ultraflex II TOF/TOF200 spectrometer using sinapinic acid as the matrix. HR ESI mass spectra were recorded on an APEX-ultra 9.4 T FTICR-MS (Bruker Daltonics).

### (2-Methyl-5-*tert*-butylphenyl)2,3-*O*-dibenzoyl-4-*O*-oxopentanoate-α-l-rhamnopyranosyl-(1→3)-2-*O*-benzoyl-4,6-*O*-benzylidene-1-thio-β-d-glucopyranoside (**19**)

A mixture of acceptor **17** (3.25 g, 6.08 mmol, 1 equiv), donor **18** (5.61 g, 9.12 mmol, 1.5 equiv), and activated pulverized 4 Å molecular sieves (12.0 g) in anhydrous CH_2_Cl_2_ (180 mL) was stirred under an argon atmosphere for 1 h; then, it was cooled to −30 °C, and TMSOTf (0.28 mL, 1.52 mmol, 0.25 equiv. to acceptor) was added. Stirring was continued until TLC analysis indicated all starting materials had disappeared (1 h). Upon completion, it was quenched by Et_3_N (0.50 mL) and filtered through a pad of Celite. The solvent was removed, and the obtained residue was purified by silica gel column chromatography using EtOAc/*n*-hexane (1:2) as eluents to give compound **19** as a white powder (5.20 g, 87%). *R*_f_ = 0.53 (silica gel, EtOAc/*n*-hexane = 2:3); ^1^H NMR (600 MH_Z_, CDCl_3_): δ 8.03–8.02 (m, 2H, Ar–H), 7.81–7.80 (m, 2H, Ar–H), 7.69–7.67 (m, 2H, Ar–H), 7.54 (d, *J* = 1.8 Hz, 1H, Ar–H), 7.51–7.47 (m, 3H, Ar–H), 7.46–7.43 (m, 1H, Ar–H), 7.40–7.27 (m, 10H, Ar–H), 7.21–7.19 (m, 1H, Ar–H), 7.07 (d, *J* = 7.8 Hz, 1H, Ar–H), 5.65 (s, 1H, Ph–CH), 5.60 (dd, *J* = 10.2, 3.6 Hz, 1H), 5.49 (dd, *J* = 10.2, 9.0 Hz, 1H), 5.35 (dd, *J* = 3.6, 1.8 Hz, 1H), 5.18 (dd, *J* = 10.2, 10.2 Hz, 1H), 5.04 (d, *J* = 1.2 Hz, 1H, C1–H_α_), 4.85 (d, *J* = 10.2 Hz, 1H, C1–H_β_), 4.41 (dd, *J* = 10.2, 5.4 Hz, 1H), 4.30–4.26 (m, 1H), 4.22 (dd, *J* = 9.6, 9.0 Hz, 1H), 3.92 (dd, *J* = 10.8, 10.2 Hz, 1H) 3.87 (dd, *J* = 9.6, 9.0 Hz, 1H), 3.64–3.60 (m, 1H), 2.59–2.53 (m, 1H, −C*H*_a_H_b_), 2.48–2.39 (m, 2H, –CH_2_), 2.31–2.24 (m, 1H, −CH_a_*H*_b_), 2.18 (s, 3H, –CH_3_), 2.00 (s, 3H, –CH_3_), 1.27 (s, 9H, *t*Bu-CH_3_), 0.84 (d, *J* = 6.0 Hz, 3H); ^13^C{^1^H} NMR (150 MHz, CDCl_3_): δ 205.8, 171.8, 165.3, 164.9, 164.5, 149.6, 136.9, 136.9, 133.1, 132.2, 130.0, 129.8, 129.7, 129.6, 129.3, 129.2, 129.2, 128.3, 128.2, 126.2, 125.3, 101.9, 97.9, 88.0, 78.8, 77.5, 73.1, 71.4, 71.0, 70.4, 69.4, 68.6, 66.5, 37.7, 34.4, 31.2, 29.5, 27.8, 20.2, 16.7; HRMS (ESI-TOF) *m*/*z*: [M + Na]^+^ calcd for C_56_H_58_O_14_SNa, 1009.3439; found, 1009.3447.

### Dibutyl 2,3-*O*-dibenzoyl-4-*O*-oxopentanoate-α-l-rhamnopyranosyl-(1→3)-2-*O*-benzoyl-4,6-*O*-benzylidene-β-d-glucopyranosyl Phosphate (**16**)

A mixture of the compound **19** (4.90 g, 4.96 mmol, 1 equiv), dibutyl phosphate (2.95 mL, 14.9 mmol, 3 equiv), and activated pulverized 4 Å molecular sieves (9 g) in anhydrous CH_2_Cl_2_ (180 mL) was stirred under an argon atmosphere for 1 h. The reaction was cooled to 0 °C; then, NIS (2.23 g, 9.93 mmol, 2 equiv) and TfOH (0.5 M in Et_2_O, 2.98 mL, 1.49 mmol, 0.3 equiv) were added. Stirring was continued until TLC analysis indicated all the starting materials had disappeared (16 h). Upon completion, it was quenched by satd. aq. NaHCO_3_ (0.5 mL) and filtered through a pad of Celite. The filtrate was quenched with 20% aq. Na_2_S_2_O_3_ (30 mL) and washed with saturated aq. NaHCO_3_ (10 mL) and brine (10 mL). The separated organic layer was dried over MgSO_4_ and concentrated. The residue was purified by silica gel column chromatography using EtOAc/*n*-hexane (1:1) as eluents to give compound 16 as a white powder (4.57 g, 92%). *R*_f_ = 0.25 (silica gel, EtOAc/*n*-hexane = 2:3); ^1^H NMR (600 MH_Z_, CDCl_3_): δ 8.02–8.00 (m, 2H, Ar–H), 7.81–7.79 (m, 2H, Ar–H), 7.70–7.68 (m, 2H, Ar–H), 7.51–7.27 (m, 14H, Ar–H), 5.63 (s, 1H, Ph–CH), 5.59 (dd, *J* = 10.2, 3.6 Hz, 1H), 5.48–5.47 (m, 2H, –CH, C1–H_β_), 5.34 (dd, *J* = 3.6, 1.8 Hz, 1H), 5.20 (dd, *J* = 10.2, 9.6 Hz, 1H), 5.03 (d, *J* = 1.2 Hz, 1H, C1–H_α_), 4.43 (dd, *J* = 10.2, 4.8 Hz, 1H), 4.29–4.24 (m, 1H), 4.22–4.18 (m, 1H), 4.06–3.98 (m, 2H), 3.90–3.85 (m, 2H), 3.78–3.66 (m, 3H), 2.59–2.53 (m, 1H, −C*H*_a_H_b_), 2.48–2.39 (m, 2H, –CH_2_), 2.33–2.26 (m, 1H, −CH_a_*H*_b_), 2.00 (s, 3H, –CH_3_), 1.65–1.60 (m, 2H, –CH_2_), 1.40–1.34 (m, 2H, –CH_2_), 1.30–1.26 (m, 2H, –CH_2_), 1.05–0.99 (m, 2H, –CH_2_), 0.91 (t, *J* = 7.2 Hz, 3H, –CH_3_), 0.86 (d, *J* = 6.0 Hz, 3H, –CH_3_), 0.67 (t, *J* = 7.2 Hz, 3H, –CH_3_); ^13^C{^1^H} NMR (150 MHz, CDCl_3_): δ 205.7, 171.8, 165.3, 164.8, 164.6, 136.7, 133.3, 133.1, 130.0, 129.7, 129.6, 129.3, 129.3, 129.1, 128.9, 128.3, 128.3, 128.2, 128.2, 126.2, 101.9, 97.7, 96.7, 96.7, 78.5, 75.8, 71.4, 70.4, 69.4, 68.4, 68.1, 68.1, 68.0, 68.0, 67.2, 66.6, 37.7, 32.0, 32.0, 31.8, 31.7, 29.5, 27.8, 18.5, 18.2, 16.7, 13.5, 13.3; ^31^P NMR (162 MH_Z_, CDCl_3_): δ −2.46; HRMS (ESI-TOF) *m*/*z*: [M + Na]^+^ calcd for C_53_H_61_O_18_PNa, 1039.3488; found, 1039.3499.

### (2-Methyl-5-*tert*-butylphenyl) 2,3-di-*O*-benzoyl-4-*O*-benzyl-α-l-rhamnopyranosyl-(1→3)-4,6-*O*-benzylidene-2-*O*-benzyl-1-thio-β-d-glucopyranoside (**21**)

A mixture of acceptor **20** (580 mg, 1.11 mmol, 1 equiv), donor **13** (843 mg, 1.39 mmol, 1.25 equiv), and activated pulverized 4 Å molecular sieves (2 g) in anhydrous CH_2_Cl_2_ (20 mL) was stirred under an argon atmosphere for 1 h; then, it was cooled to −30 °C, and TMSOTf (30.3 μL, 0.17 mmol, 0.15 equiv. with respect to acceptor) was added. Stirring was continued until TLC analysis indicated all starting materials had disappeared (1 h). Upon completion, it was quenched by Et_3_N (0.2 mL) and filtered through a pad of Celite. The solvent was removed, and the obtained residue was purified by silica gel column chromatography using EtOAc/*n*-hexane/CH_2_Cl_2_ (1:6:1) as eluents to give compound **21** as a white powder (1.06 g, 99%). *R*_f_ = 0.59 (silica gel, EtOAc/hexane/CH_2_Cl_2_ = 1:4:1); ^1^H NMR (600 MH_Z_, CDCl_3_): δ 7.98–7.96 (m, 2H, Ar–H), 7.92–7.90 (m, 2H, Ar–H), 7.63 (d, *J* = 1.8 Hz, 1H, Ar–H), 7.63–7.59 (m, 1H, Ar–H), 7.51–7.49 (m, 3H, Ar–H), 7.47–7.45 (m, 2H, Ar–H), 7.38–7.33 (m, 4H, Ar–H), 7.29–7.26 (m, 1H, Ar–H), 7.24–7.20 (m, 6H, Ar–H), 7.14 (d, *J* = 7.8 Hz, 1H, Ar–H), 7.12–7.08 (m, 4H, Ar–H), 7.07–7.04 (m, 1H, Ar–H), 5.79 (dd, *J* = 3.6, 1.8 Hz, 1H), 5.76 (dd, *J* = 9.6, 3.6 Hz, 1H), 5.60 (s, 1H, Ph–CH), 5.34 (d, *J* = 1.2 Hz, 1H, C1–H_α_), 5.09 (d, *J* = 10.2 Hz, 1H), 4.80 (d, *J* = 10.2 Hz, 1H, C1–H_β_), 4.78 (d, *J* = 9.6 Hz, 1H), 4.59 (d, *J* = 10.8 Hz, 1H), 4.53 (d, *J* = 10.8 Hz, 1H), 4.39–4.36 (m, 2H), 4.07 (dd, *J* = 9.6, 8.4 Hz, 1H), 3.86 (dd, *J* = 10.8, 10.2 Hz, 1H), 3.76–3.66 (m, 3H), 3.52 (ddd, *J* = 9.6, 9.6, 4.8 Hz, 1H) 2.40 (s, 3H, –CH_3_) 1.32 (s, 9H, *t*Bu-CH_3_), 0.97 (d, *J* = 6.0 Hz, 3H, –CH_3_); ^13^C{^1^H} NMR (150 MHz, CDCl_3_): δ 165.6, 165.2, 149.6, 137.9, 137.4, 136.9, 135.9, 133.2, 133.0, 132.8, 129.9, 129.8, 129.8, 129.6, 129.6, 128.9, 128.6, 128.4, 128.3, 128.2, 128.2, 128.1, 128.1, 127.7, 127.7, 127.6, 126.3, 124.7, 101.7, 98.0, 88.6, 82.1, 79.0, 78.9, 78.4, 75.9, 74.4, 72.3, 70.9, 70.5, 68.7, 67.5, 34.5, 31.3, 20.3, 17.4; HRMS (ESI-TOF) *m*/*z*: [M + Na]^+^ calcd for C_58_H_60_O_11_SNa, 987.3749; found, 987.3775.

### (2-Methyl-5-*tert*-butylphenyl) 2,3-di-*O*-benzoyl-4-*O*-benzyl-α-l-rhamnopyranosyl-(1→3)-6-*O*-acetyl-2-*O*-benzyl-1-thio-β-d-glucopyranoside (**22**)

To a stirred solution of starting material **21** (1.00 g, 1.04 mmol, 1 equiv) in a mixture of CH_2_Cl_2_/MeOH (10 mL, 1:1 = *v*/*v*), 4% aq. HCl was added until pH was adjusted to 2. The reaction mixture was stirred at 40 °C until TLC analysis indicated all the starting material (20 h) had disappeared. The solvent was removed by rotary evaporation under high vacuum and coevaporated with toluene twice. The obtained residue was dissolved in CH_2_Cl_2_ (10 mL) at 0 °C; then, Ac_2_O (97.7 μL, 1.04 mmol, 1 equiv) and Et_3_N (1.30 mL, 9.32 mmol, 9 equiv) were added. Stirring was continued until TLC analysis indicated the starting material had disappeared (1 h). Upon completion, the reaction mixture was quenched by MeOH (1 mL), and stirring was continued for another 10 min. The solvent was removed by rotary evaporation under high vacuum. The residue was purified by silica gel column chromatography using EtOAc/*n*-hexane (1:2) as eluents to give compound **22** as a white powder (693 mg, 73%). *R*_f_ = 0.19 (silica gel, EtOAc/*n*-hexane = 1:3); ^1^H NMR (600 MH_Z_, CDCl_3_): δ 7.98–7.97 (m, 2H, Ar–H), 7.91–7.90 (m, 2H, Ar–H), 7.61–7.58 (m, 2H, Ar–H), 7.53–7.50 (m, 1H, Ar–H), 7.49–7.44 (m, 4H, Ar–H), 7.37–7.34 (m, 2H, Ar–H), 7.24–7.18 (m, 8H, Ar–H), 7.16–7.14 (m, 1H, Ar–H), 7.11 (d, *J* = 8.4 Hz, 1H, Ar–H), 5.77–5.74 (m, 2H), 5.21 (s, 1H, C1–H_α_), 4.96 (d, *J* = 9.6 Hz, 1H), 4.88 (dd, *J* = 9.6 Hz, 1H), 4.74 (d, *J* = 10.8 Hz, 1H), 4.64 (d, *J* = 10.8 Hz, 1H, C1–H_β_), 4.63 (d, *J* = 10.2 Hz, 1H), 4.39 (dd, *J* = 12.0, 1.8 Hz, 1H), 4.34(dd, *J* = 12.0, 5.4 Hz, 1H), 4.27 (dq, *J* = 9.6, 6.0 Hz, 1H), 3.87 (d, *J* = 2.4 Hz, 1H), 3.81 (dd, *J* = 9.0, 9.0 Hz, 1H), 3.70 (dd, *J* = 8.4, 8.4 Hz, 1H), 3.58–3.51 (m, 2H), 3.48–3.44 (m, 1H), 2.39 (s, 3H, –CH_3_), 2.09 (s, 3H, –CH_3_), 1.42 (d, *J* = 6.0 Hz, 3H, –CH_3_), 1.28 (s, 9H, *t*Bu-CH_3_); ^13^C{^1^H} NMR (150 MHz, CDCl_3_): δ 171.3, 165.5, 165.1, 149.5, 137.6, 137.4, 136.2, 133.4, 133.2, 133.1, 129.9, 129.8, 129.7, 129.6, 129.5, 128.7, 128.6, 128.5, 128.4, 128.3, 128.1, 128.0, 127.8, 124.6, 99.4, 88.2, 88.2, 79.6, 78.5, 77.3, 76.1, 75.2, 72.2, 70.9, 69.3, 69.2, 63.8, 34.5, 31.3, 21.0, 20.3, 18.1; HRMS (ESI-TOF) *m*/*z*: [M + Na]^+^ calcd for C_53_H_58_O_12_SNa, 941.3541; found, 941.3568.

### (2-Methyl-5-*tert*-butylphenyl) 2,3-*O*-dibenzoyl-4-*O*-oxopentanoate-α-l-rhamnopyranosyl-(1→3)-2-*O*-benzoyl-4,6-*O*-benzylidene-β-d-glucopyranosyl-(1→4)-[2,3-di-*O*-benzoyl-4-*O*-benzyl-α-l-rhamnopyranosyl-(1→3)]-6-*O*-acetyl-2-*O*-benzyl-1-thio-β-d-glucopyranoside (**23a**) and [2 + 2] Individual Synthesis of Tetrasaccharide

A mixture of acceptor **22** (150 mg, 0.16 mmol, 1 equiv), donor **16** (490 mg, 0.49 mmol, 3 equiv), and activated pulverized 4 Å molecular sieves (500 mg) in anhydrous CH_2_Cl_2_ (5 mL) was stirred under an argon atmosphere for 1 h; then, it was cooled to −30 °C, and TMSOTf (89 μL, 0.49 mmol, 3 equiv. with respect to acceptor) was added. Stirring was continued for 3 h. The reaction mixture was quenched by Et_3_N (0.1 mL) and filtered through a pad of Celite. The solvent was removed, and the obtained residue was purified by silica gel column chromatography using EtOAc/*n*-hexane (1:2) as eluents to give compound 23a as a white powder (110 mg, 43%) and the recovered starting material (34 mg, b.r.s.m. 55%). [3 + 1] individual synthesis of tetrasaccharide: A mixture of acceptor **12a** (70.0 mg, 0.055 mmol, 1 equiv), donor 13 (49.6 mg, 0.082 mmol, 1.5 equiv), and activated pulverized 4 Å molecular sieves (200 mg) in anhydrous CH_2_Cl_2_ (2 mL) was stirred under an argon atmosphere for 1 h; then, it was cooled to −30 °C, and TMSOTf (2.48 μL, 0.014 mmol, 0.25 equiv. with respect to acceptor) was added. Stirring was continued until TLC analysis indicated all of the starting materials disappeared (30 min). Upon completion, it was quenched by Et_3_N (0.1 mL) and filtered through a pad of Celite. The solvent was removed, and the obtained residue was purified by silica gel column chromatography using EtOAc/*n*-hexane (1:2) as eluents to give compound 23a as a white powder (86 mg, 91%). *R*_f_ = 0.55 (silica gel, EtOAc/*n*-hexane = 1:1); ^1^H NMR (600 MH_Z_, CDCl_3_): δ 8.02–8.00 (m, 4H, Ar–H), 7.96–7.95 (m, 2H, Ar–H), 7.82–7.81 (m, 2H, Ar–H), 7.66–7.65 (m, 2H, Ar–H), 7.62–7.59 (m, 1H, Ar–H), 7.56–7.53 (m, 1H, Ar–H), 7.50–7.43 (m, 5H, Ar–H), 7.41–7.25 (m, 20H, Ar–H), 7.14–7.11 (m, 2H, Ar–H), 7.05 (d, *J* = 7.8 Hz, 1H, Ar–H), 6.99–6.94 (m, 3H, Ar–H), 5.76 (dd, *J* = 3.6, 1.8 Hz, 1H), 5.70 (dd, *J* = 9.6, 3.6 Hz, 1H), 5.65 (dd, *J* = 10.2, 3.6 Hz, 1H), 5.47 (d, *J* = 1.2 Hz, 1H, C1–H_α_), 5.34 (dd, *J* = 9.0, 8.4 Hz, 1H), 5.32 (dd, *J* = 3.6, 1.8 Hz, 1H), 5.17 (dd, *J* = 10.2, 10.2 Hz, 1H), 5.04 (d, *J* = 9.6 Hz, 1H), 4.93 (d, *J* = 1.2 Hz, 1H, C1–H_α_), 4.87 (dq, *J* = 9.6, 6.0 Hz, 1H), 4.82 (d, *J* = 9.6 Hz, 1H), 4.73 (d, *J* = 9.6 Hz, 1H), 4.65 (d, *J* = 10.2 Hz, 1H), 4.57 (d, *J* = 8.4 Hz, 1H, C1–H_β_), 4.49 (d, *J* = 10.2 Hz, 1H, C1–H_β_), 4.41 (dd, *J* = 10.8, 4.8 Hz, 1H), 4.23 (s, 1H, Ph–CH), 4.21–4.14 (m, 3H), 4.01 (dd, *J* = 9.6, 9.0 Hz, 1H), 3.91 (dd, *J* = 9.0, 9.0 Hz, 1H), 3.85 (dd, *J* = 9.6, 9.0 Hz, 1H), 3.81–3.77 (m, 2H), 3.58 (dd, *J* = 9.6, 9.0 Hz, 1H), 3.41 (dd, *J* = 9.6, 9.0 Hz, 1H), 3.35 (ddd, *J* = 9.6, 9.6, 4.8 Hz, 1H), 3.23 (ddd, *J* = 9.6, 4.8, 2.4 Hz, 1H), 2.63–2.58 (m, 1H, −C*H*_a_H_b_), 2.53–2.46 (m, 2H, –CH_2_), 2.38–2.33 (m, 1H, −CH_a_*H*_b_), 2.30 (s, 3H, –CH_3_), 2.04 (s, 3H, –CH_3_), 2.01 (s, 3H, –CH_3_), 1.71 (d, *J* = 6.6 Hz, 3H, –CH_3_), 1.18 (s, 9H, *t*Bu-CH_3_), 0.76 (d, *J* = 6.0 Hz, 3H); ^13^C{^1^H} NMR (150 MHz, CDCl_3_): δ 205.8, 171.9, 170.4, 165.5, 165.4, 165.3, 164.4, 164.3, 149.5, 137.8, 137.3, 137.1, 136.5, 133.2, 133.1, 133.1, 133.1, 132.8, 129.9, 129.9, 129.8, 129.7, 129.7, 129.6, 129.4, 129.2, 129.2, 129.0, 128.8, 128.8, 128.7, 128.4, 128.4, 128.3, 128.3, 128.3, 128.2, 128.1, 128.0, 127.8, 127.5, 126.2, 124.9, 101.1, 100.5, 97.9, 97.4, 88.5, 82.1, 79.7, 77.7, 76.6, 76.4, 76.2, 75.3, 74.5, 74.3, 72.6, 71.7, 70.9, 70.4, 69.4, 68.1, 67.7, 67.6, 66.3, 62.3, 37.8, 34.4, 31.2, 29.6, 27.9, 20.9, 20.3, 18.1, 16.6; HRMS (ESI-TOF) *m*/*z*: [M + H]^+^ calcd for C_98_H_101_O_26_S 1725.6296; found, 1725.6329.

### 5-Azidopentyl 2,3-*O*-dibenzoyl-4-*O*-oxopentanoate-α-l-rhamnopyranosyl-(1→3)-2-*O*-benzoyl-4,6-*O*-benzylidene-β-d-glucopyranosyl-(1→4)-[2,3-di-*O*-benzoyl-4-*O*-benzyl-α-l-rhamnopyranosyl-(1→3)]-6-*O*-acetyl-2-*O*-benzyl-α-d-glucopyranosyl-(1→2)-3,4,6-*tri*-*O*-benzyl-α-d-glucopyranoside (9) and [4 + 1] Individual (Scheme 2)

A mixture of acceptor **14** (39.1 mg, 0.070 mmol, 1.2 equiv), donor **23a** (100 mg, 0.058 mmol, 1 equiv), and activated pulverized 4 Å molecular sieves (1 g) in anhydrous CH_2_Cl_2_ (1 mL) was stirred under an argon atmosphere for 1 h. The reaction was cooled to −30 °C; then, NIS (16.9 mg, 0.075 mmol, 1.3 equiv) and TfOH (0.5 M in Et_2_O, 35 μL, 0.017 μmol, 0.3 equiv) were added. Stirring was continued until TLC analysis indicated all starting materials had disappeared (1 h). Then, the reaction was quenched by Et_3_N (0.1 mL) and filtered through a pad of Celite. The filtrate was quenched with 20% aq. Na_2_S_2_O_3_ (2 mL) and washed with saturated aq. NaHCO_3_ (1 mL) and brine (1 mL). The separated organic layer was dried over MgSO_4_ and concentrated. The obtained residue was purified by silica gel column chromatography using EtOAc/toluene (1:4) as eluents to give compound **9** as a white powder (84 mg, 69%). [**2 + 2 + 1] one-pot** (Scheme 3): A mixture of acceptor **22** (100 mg, 0.11 mmol, 1 equiv), donor **16** (327 mg, 0.33 mmol, 3 equiv), and activated pulverized 4 Å molecular sieves (1 g) in anhydrous CH_2_Cl_2_ (10 mL) was stirred under an argon atmosphere for 1 h; then it was cooled to −30 °C, and TMSOTf (99 μL, 0.54 mmol, 5 equiv. with respect to acceptor) was added. Stirring was continued for 3 h; then, a solution of acceptor **14** (91.7 mg, 0.16 mmol, 1.5 equiv) in CH_2_Cl_2_ (1 mL) was added. The mixture was vigorously stirred for 30 min at −30 °C, and NIS (31.8 mg, 0.14 mmol, 1.3 equiv) was added. Stirring was continued until TLC analysis indicated all the starting materials had disappeared (1 h). Then, then reaction was quenched by Et_3_N (0.1 mL) and filtered through a pad of Celite. The filtrate was quenched with 20% aq. Na_2_S_2_O_3_ (5 mL) and washed with saturated aq. NaHCO_3_ (3 mL) and brine (2 mL). The solvent was removed, and the obtained residue was purified by silica gel column chromatography using EtOAc/toluene (1:4) as eluents. The second column was eluted by acetone/*n*-hexane = 2:3 to give compound **9** as a white powder (45 mg, 20%). [**3 + 1 + 1] one-pot** (Scheme 5): A mixture of acceptor **12a** (1.00 g, 0.78 mmol, 1 equiv), donor **13** (708 mg, 1.17 mmol, 1.5 equiv), and activated pulverized 4 Å molecular sieves (3 g) in anhydrous CH_2_Cl_2_ (20 mL) was stirred under an argon atmosphere for 1 h; then, it was cooled to −30 °C, and TMSOTf (35.4 μL, 0.20 mmol, 0.25 equiv. with respect to acceptor) was added. Stirring was continued until TLC analysis indicated all the starting materials had disappeared (30 min). A solution of acceptor **14** (526 mg, 0.94 mmol, 1.2 equiv) in CH_2_Cl_2_ (10 mL) was added, and the mixture was vigorously stirred for 30 min at −30 °C; then, NIS (228 mg, 1.01 mmol, 1.3 equiv) and TfOH (0.5 M in Et_2_O, 0.47 mL, 0.23 mmol, 0.3 equiv) were added. Stirring was continued until TLC analysis indicated all starting materials had disappeared (1 h). Upon completion, the reaction was quenched by Et_3_N (1 mL) and filtered through a pad of Celite. The filtrate was then quenched with 20% aq. Na_2_S_2_O_3_ (30 mL) and washed with saturated aq. NaHCO_3_ (20 mL) and brine (10 mL). The solvent was removed, and the obtained residue was purified by silica gel column chromatography using EtOAc/toluene (1:4) as eluents to give compound **9** as a white powder (1.10 g, 67%). [**4 + 1**] **individual** (Scheme 6): A mixture of acceptor **26** (2.09 g, 1.26 mmol, 1 equiv), donor **13** (1.14 g, 1.89 mmol, 1.5 equiv), and activated pulverized 4 Å molecular sieves (4 g) in anhydrous CH_2_Cl_2_ (80 mL) was stirred under an argon atmosphere for 1 h. The reaction was cooled to −30 °C, and TMSOTf (57 μL, 0.31 mmol, 0.25 equiv. with respect to acceptor) was added. Stirring was continued until TLC analysis indicated all the starting materials had disappeared (40 min). Then, the reaction was quenched by Et_3_N (0.5 mL) and filtered through a pad of Celite. The solvent was removed, and the obtained residue was purified by silica gel column chromatography using EtOAc/toluene (1:4) as eluents to give compound **9** as a white powder (2.54 g, 96%). [**3 + 1 + 1] one-pot** (Scheme 11): A mixture of acceptor **12b** (100 mg, 0.078 mmol, 1 equiv), donor **13** (70.8 mg, 0.12 mmol, 1.5 equiv), and activated pulverized 4 Å molecular sieves (400 mg) in anhydrous CH_2_Cl_2_ (2 mL) was stirred under an argon atmosphere for 1 h. The reaction was cooled to −30 °C, and TMSOTf (3.54 μL, 0.020 mmol, 0.25 equiv. with respect to acceptor) was added. Stirring was continued until TLC analysis indicated all starting materials had disappeared (30 min). Upon completion, a solution of acceptor **14** (52.6 mg, 0.094 mmol, 1.2 equiv) in CH_2_Cl_2_ (1 mL) was added, and the mixture was vigorously stirred for 30 min at −30 °C; then, NIS (22.8 mg, 0.10 mmol, 1.3 equiv) and TfOH (0.5 M in Et_2_O, 47 μL, 0.023 mmol, 0.3 equiv) were added. Stirring was continued until TLC analysis indicated all starting materials had disappeared (1 h). Upon completion, it was quenched by Et_3_N (0.1 mL) and filtered through a pad of Celite. The filtrate was then quenched with 20% aq. Na_2_S_2_O_3_ (5 mL) and washed with saturated aq. NaHCO_3_ (3 mL) and brine (2 mL). The solvent was removed, and the obtained residue was purified by silica gel column chromatography using EtOAc/toluene (1:4) as eluents to give compound **9** as a white powder (66 mg, 40%). *R*_f_ = 0.58 (silica gel, EtOAc/toluene = 1:3); ^1^H NMR (600 MH_Z_, CDCl_3_): δ 8.02–7.97 (m, 6H, Ar–H), 7.83–7.81 (m, 2H, Ar–H), 7.68–7.66 (m, 2H, Ar–H), 7.60–7.58 (m, 1H, Ar–H), 7.55–7.53 (m, 1H, Ar–H), 7.50–7.44 (m, 4H, Ar–H), 7.41–7.31 (m, 8H, Ar–H), 7.30–7.22 (m, 25H, Ar–H), 7.16–7.13 (m, 3H, Ar–H), 7.10–7.09 (m, 1H, Ar–H), 7.08–7.05 (m, 2H, Ar–H), 5.74 (dd, *J* = 3.0, 1.2 Hz, 1H), 5.70 (dd, *J* = 9.6, 3.6 Hz, 1H), 5.66 (dd, *J* = 10.2, 3.6 Hz, 1H), 5.47 (d, *J* = 1.2 Hz, 1H, C1–H_α_), 5.37 (dd, *J* = 9.0, 8.4 Hz, 1H), 5.35 (dd, *J* = 3.6, 1.2 Hz, 1H), 5.15 (dd, *J* = 10.2, 10.2 Hz, 1H), 5.03 (d, *J* = 3.6 Hz, 1H, C1–H_α_), 4.97 (d, *J* = 3.6 Hz, 1H, C1–H_α_), 4.91 (d, *J* = 1.2 Hz, 1H, C1–H_α_), 4.90–4.86 (m, 1H), 4.84 (d, *J* = 10.2 Hz, 1H), 4.80 (d, *J* = 10.8 Hz, 1H), 4.75 (d, *J* = 9.6 Hz, 1H), 4.68 (d, *J* = 10.2 Hz, 1H), 4.65 (d, *J* = 11.4 Hz, 1H), 4.59–4.52 (m, 4H, C1–H_β_), 4.45–4.40 (m, 2H), 4.36 (d, *J* = 10.8 Hz, 1H), 4.26 (s, 1H, Ph–CH), 4.24–4.17 (m, 3H), 4.05 (d, *J* = 12.0 Hz, 1H), 4.00 (dd, *J* = 9.6, 9.0 Hz, 1H), 3.91 (dd, *J* = 9.6, 9.0 Hz, 1H), 3.83–3.78 (m, 4H), 3.71–3.68 (m, 1H), 3.66–3.55 (m, 5H), 3.46 (dd, *J* = 9.6, 9.0 Hz, 1H), 3.42 (dd, *J* = 9.6, 9.6 Hz, 1H), 3.36–3.32 (m, 2H), 3.09 (t, *J* = 7.2 Hz, 2H, –CH_2linker_), 2.63–2.57 (m, 1H, −C*H*_a_H_b_), 2.53–2.46 (m, 2H, –CH_2_), 2.38–2.33 (m, 1H, −CH_a_*H*_b_), 2.03 (s, 3H, –CH_3_), 2.01 (s, 3H, –CH_3_), 1.67 (d, *J* = 6.0 Hz, 3H, –CH_3_), 1.56–1.50 (m, 2H, –CH_2linker_), 1.47–1.43 (m, 2H, –CH_2linker_), 1.32–1.26 (m, 2H, –CH_2linker_), 0.74 (d, *J* = 6.0 Hz, 3H, –CH_3_); ^13^C{^1^H} NMR (150 MHz, CDCl_3_): δ 205.6, 171.8, 170.4, 165.5, 165.3, 165.3, 164.3, 164.3, 138.3, 138.2, 138.0, 137.9, 137.4, 137.1, 133.2, 133.0, 130.0, 129.8, 129.8, 129.7, 129.7, 129.6, 129.4, 129.2, 129.0, 128.8, 128.8, 128.7, 128.5, 128.4, 128.4, 128.4, 128.3, 128.3, 128.2, 128.1, 127.9, 127.8, 127.7, 127.7, 127.6, 127.6, 126.2, 101.2, 100.6, 98.0, 97.4, 95.9, 94.1, 80.5, 80.3, 79.8, 77.7, 77.6, 76.7, 76.2, 75.7, 75.0, 74.8, 74.3, 73.4, 73.0, 72.7, 72.4, 71.7, 70.9, 70.3, 70.3, 69.5, 69.5, 68.6, 68.2, 67.8, 67.6, 67.4, 66.3, 61.8, 51.1, 37.7, 29.6, 29.0, 28.5, 27.9, 23.4, 20.9, 18.1, 16.6; HRMS (ESI-TOF) *m*/*z*/[M + Na]^+^ calcd for C_119_H_123_N_3_O_32_Na, 2128.7982; found, 2128.8015.

### (2-Methyl-5-*tert*-butylphenyl) 2,3-*O*-dibenzoyl-4-*O*-oxopentanoate-α-l-rhamnopyranosyl-(1→3)-2-*O*-benzoyl-4,6-*O*-benzylidene-β-d-glucopyranosyl-(1→4)-6-*O*-acetyl-2-*O*-benzyl-3-*O*-(2-naphthylmethyl)-1-thio-β-d-glucopyranoside (**24a**)

A mixture of acceptor **15** (2.50 g, 4.07 mmol, 1 equiv), donor **16** (4.97 g, 4.96 mmol, 1.22 equiv), and activated pulverized 4 Å molecular sieves (10 g) in anhydrous CH_2_Cl_2_ (150 mL) was stirred under an argon atmosphere for 1 h. The reaction was cooled to −40 °C, and TMSOTf (0.90 mL, 4.96 mmol, 1.22 equiv) was added. Stirring was continued until TLC analysis indicated all starting materials had disappeared (30 min). Then, the reaction was quenched by Et_3_N (0.5 mL) and filtered through a pad of Celite. The solvent was removed, and the obtained residue was purified by silica gel column chromatography using EtOAc/*n*-hexane (1:2) to give compound **24a** as a white powder (5.66 g, 98%). *R*_f_ = 0.60 (silica gel, EtOAc/*n*-hexane = 1:1); ^1^H NMR (600 MH_Z_, CDCl_3_): δ 8.01–8.00 (m, 2H, Ar–H), 7.88–7.86 (m, 3H, Ar–H), 7.80–7.79 (m, 3H, Ar–H), 7.66–7.47 (m, 5H, Ar–H), 7.44 (t, *J* = 7.2 Hz, 1H, Ar–H), 7.41–7.37 (m, 3H, Ar–H), 7.34–7.24 (m, 14H, Ar–H), 7.15 (dd, *J* = 7.8, 1.8 Hz, 1H, Ar–H), 7.06 (d, *J* = 7.8 Hz, 1H, Ar–H), 5.57 (dd, *J* = 10.2, 3.6 Hz, 1H), 5.42 (dd, *J* = 8.4, 8.4 Hz, 1H), 5.35 (s, 1H, Ph–CH), 5.29 (dd, *J* = 3.6, 1.8 Hz, 1H), 5.18–5.13 (m, 2H), 5.01 (d, *J* = 11.4 Hz, 1H), 4.97 (s, 1H, C1–H_α_), 4.92 (d, *J* = 10.8 Hz, 1H), 4.80 (d, *J* = 10.2 Hz, 1H), 4.70 (d, *J* = 8.4 Hz, 1H, C1–H_β_), 4.53 (d, *J* = 9.6 Hz, 1H, C1–H_β_), 4.23–4.19 (m, 1H), 4.16–4.09 (m, 4H), 3.84 (dd, *J* = 9.6, 9.0 Hz, 1H), 3.70 (dd, *J* = 9.0, 9.0 Hz, 1H), 3.60 (dd, *J* = 9.6, 9.0 Hz, 1H), 3.52 (dd, *J* = 9.6, 9.0 Hz, 1H), 3.41–3.37 (m, 1H), 3.34–3.29 (m, 2H), 2.58–2.52 (m, 1H, −C*H*_a_H_b_), 2.47–2.37 (m, 2H, –CH_2_), 2.33 (s, 3H, –CH_3_), 2.31–2.26 (m, 1H, −CH_a_*H*_b_), 2.00 (s, 3H, –CH_3_), 1.88 (s, 3H, –CH_3_), 1.20 (s, 9H, *t*Bu-CH_3_), 0.80 (d, *J* = 6.0 Hz, 3H, –CH_3_); ^13^C{^1^H} NMR (150 MHz, CDCl_3_): δ 205.7, 171.8, 170.4, 165.3, 164.6, 164.4, 149.4, 138.0, 136.8, 136.6, 136.4, 133.3, 133.2, 133.1, 132.9, 132.7, 130.0, 129.8, 129.7, 129.6, 129.3, 129.2, 129.1, 128.4, 128.3, 128.3, 128.2, 128.2, 128.1, 128.0, 127.9, 127.8, 127.7, 126.2, 126.0, 125.8, 125.8, 101.7, 101.4, 97.9, 88.3, 84.7, 80.7, 78.7, 77.6, 76.4, 76.2, 75.8, 75.6, 74.7, 71.3, 70.4, 69.4, 68.4, 66.7, 66.5, 62.6, 37.7, 34.4, 31.2, 29.5, 27.8, 20.7, 20.3, 16.6; HRMS (ESI-TOF) *m*/*z*: [M + Na]^+^ calcd for C_82_H_84_O_20_SNa, 1443.5169; found, 1443.5192.

### (2-Methyl-5-*tert*-butylphenyl) 2,3-*O*-dibenzoyl-4-*O*-oxopentanoate-α-l-rhamnopyranosyl-(1→3)-2-*O*-benzoyl-4,6-*O*-benzylidene-β-d-glucopyranosyl-(1→4)-6-*O*-acetyl-2-*O*-benzyl-1-thio-β-d-glucopyranoside (**12a**)

To a stirred solution of starting material **24a** (1.21 g, 0.85 mmol, 1 equiv) in a mixture of CH_2_Cl_2_/phosphate buffer, pH 7 (40 mL, 9:1 = *v*/*v*), 2,3-dichloro-5,6-dicyanobenzoquinone (386 mg, 1.70 mmol, 2 equiv) was added at 0 °C. The reaction mixture was vigorously stirred until TLC analysis indicated all the starting material had disappeared (3 h). Upon completion, the reaction mixture was diluted with CH_2_Cl_2_ (30 mL) and washed with saturated aq. NaHCO_3_ (20 mL) and brine (10 mL). The organic phase was washed with water until the solution became colorless; then, the separated organic layer was dried over MgSO_4_, filtered, and concentrated. The obtained residue was purified by silica gel column chromatography using EtOAc/*n*-hexane (1:2) as eluents to give compound 12 as a white powder (1.00 g, 92%). *R*_f_ = 0.49 (silica gel, EtOAc/*n*-hexane = 2:3); ^1^H NMR (600 MH_Z_, CDCl_3_): δ 7.96 (d, *J* = 7.8 Hz, 2H, Ar–H), 7.78 (d, *J* = 7.2 Hz, 2H, Ar–H), 7.64 (d, *J* = 7.8 Hz, 2H, Ar–H), 7.51–7.43 (m, 7H, Ar–H), 7.38–7.26 (m, 13H, Ar–H), 7.14 (dd, *J* = 7.8, 1.8 Hz, 1H, Ar–H), 7.05 (d, *J* = 8.4 Hz, 1H, Ar–H), 5.64 (s, 1H, Ph–CH), 5.56 (dd, *J* = 10.2, 3.6 Hz, 1H), 5.43 (dd, *J* = 8.4, 8.4 Hz, 1H), 5.29–5.28 (m, 1H, C1–H_α_), 5.17 (dd, *J* = 10.2, 10.2 Hz, 1H), 4.98 (s, 1H, C1–H_α_), 4.91 (d, *J* = 10.8 Hz, 1H), 4.85 (d, *J* = 10.8 Hz, 1H), 4.71 (d, *J* = 7.8 Hz, 1H, C1–H_β_), 4.55 (d, *J* = 10.2 Hz, 1H, C1–H_β_), 4.48 (dd, *J* = 10.2, 4.8 Hz, 1H), 4.27–4.19 (m, 2H), 4.01 (d, *J* = 10.8 Hz, 1H), 3.95 (dd, *J* = 12.0, 4.8 Hz, 1H), 3.92–3.85 (m, 2H), 3.79 (dd, *J* = 9.0, 8.4 Hz, 1H), 3.74 (s, 1H), 3.68 (ddd, *J* = 9.6, 9.6, 4.8 Hz, 1H), 3.51 (dd, *J* = 9.6, 8.4 Hz, 1H), 3.39 (t, *J* = 9.0 Hz, 2H), 2.58–2.53 (m, 1H, −C*H*_a_H_b_), 2.48–2.38 (m, 2H, –CH_2_), 2.32 (s, 3H, –CH_3_), 2.30–2.25 (m, 1H, −CH_a_*H*_b_), 2.00 (s, 3H, –CH_3_), 1.81 (s, 3H, –CH_3_), 1.20 (s, 9H, *t*Bu-CH_3_), 0.83 (d, *J* = 6.0 Hz, 3H, –CH_3_); ^13^C{^1^H} NMR (150 MHz, CDCl_3_): δ 205.7, 171.8, 170.1, 165.3, 164.6, 164.4, 149.4, 138.2, 136.6, 133.3, 133.1, 132.6, 130.0, 129.8, 129.7, 129.6, 129.4, 129.2, 129.1, 129.1, 128.4, 128.3, 128.2, 128.1, 127.7, 126.2, 124.7, 102.0, 102.0, 98.0, 87.6, 81.0, 80.1, 78.5, 76.0, 75.3, 75.2, 74.1, 71.3, 70.3, 69.4, 68.3, 67.1, 66.6, 62.6, 37.7, 34.4, 31.3, 29.5, 27.8, 20.6, 20.3, 16.6; HRMS (ESI-TOF) *m*/*z*: [M + Na]^+^ calcd for C_71_H_76_O_20_SNa, 1303.4543; found, 1303.4538.

### 5-Azidopentyl 2,3-*O*-dibenzoyl-4-*O*-oxopentanoate-α-l-rhamnopyranosyl-(1→3)-2-*O*-benzoyl-4,6-*O*-benzylidene-β-d-glucopyranosyl-(1→4)-6-*O*-acetyl-2-*O*-benzyl-*3*-*O*-(2-naphthylmethyl)-α-d-glucopyranosyl-(1→2)-3,4,6-*tri*-*O*-benzyl-α-d-glucopyranoside (**25**)

A mixture of acceptor **14** (190 mg, 0.34 mmol, 0.8 equiv), donor **24a** (600 mg, 0.42 mmol, 1 equiv), and activated pulverized 4 Å molecular sieves (1 g) in anhydrous CH_2_Cl_2_/ether (20 mL, 1:1 = *v*/*v*) was stirred under an argon atmosphere for 1 h. The reaction was cooled to −30 °C; then, NIS (104 mg, 0.46 mmol, 1.1 equiv) and TfOH (0.5 M in Et_2_O, 0.17 mL, 84.4 μmol, 0.2 equiv) were added. Stirring was continued until TLC analysis indicated all starting materials had disappeared (1 h). Then, the reaction was quenched by Et_3_N (0.2 mL), and the solution was filtered through a pad of Celite. The filtrate was quenched with 20% aq. Na_2_S_2_O_3_ (5 mL) and washed with saturated aq. NaHCO_3_ (3 mL) and brine (2 mL). The separated organic layer was dried over MgSO_4_ and concentrated. The obtained residue was purified by silica gel column chromatography using EtOAc/toluene (1:4) as eluents to give (α/β = 29:1) compound **25** as a white powder (α isomer 442 mg, 73% and β isomer 15 mg, 2.5%). **[2 + 1 + 1] one-pot** (Scheme 7): A mixture of acceptor **15** (100 mg, 0.16 mmol, 1 equiv), donor **16** (204 mg, 0.20 mmol, 1.25 equiv), and activated pulverized 4 Å molecular sieves (500 mg) in anhydrous CH_2_Cl_2_ (3 mL) was stirred under an argon atmosphere for 1 h. The reaction was cooled to −40 °C, and TMSOTf (37 μL, 0.20 mmol, 1.25 equiv. with respect to acceptor) was added. Stirring was continued for 30 min; then acceptor **14** (110 mg, 0.20 mmol, 1.2 equiv) in CH_2_Cl_2_ (1 mL) was added. The mixture was vigorously stirred for 30 min at −40 °C, and NIS (47.6 mg, 0.21 mmol, 1.3 equiv) was added. The reaction was warmed to −30 °C, and stirring was continued for 1 h; then, the reaction was quenched by Et_3_N (0.1 mL), and the solution was filtered through a pad of Celite. The filtrate was quenched with 20% aq. Na_2_S_2_O_3_ (3 mL) and washed with saturated aq. NaHCO_3_ (2 mL) and brine (1 mL). The separated organic layer was dried over MgSO_4_ and concentrated, and the obtained residue was purified by silica gel column chromatography using EtOAc/toluene (1:4) as eluents to give compound **25** as a white powder (α/β > 20:1, α isomer 163 mg, 56%) and a side phosphate product **27a** (55 mg, 23%).

### Recovering Tetrasaccharide **25** by Side Phosphate Product **27a**

A mixture of acceptor **14** (32.5 mg, 0.058 mmol, 0.84 equiv), donor **27a** (100 mg, 0.069 mmol, 1 equiv), and activated pulverized 4 Å molecular sieves (200 mg) in anhydrous CH_2_Cl_2_ (2 mL) was stirred under an argon atmosphere for 1 h. The reaction was cooled to −30 °C, and f TMSOTf (19 μL, 0.10 mmol, 1.5 equiv. with respect to acceptor) was added. Stirring was continued until TLC analysis indicated all starting materials had disappeared (1.5 h). Then, the reaction was quenched by Et_3_N (0.1 mL), and the solution was filtered through a pad of Celite. The solvent was removed, and the obtained residue was purified by silica gel column chromatography using EtOAc/*n*-hexane (1:1) as eluents to give compound **25** as a white powder (80 mg, 77%). *R*_f_ = 0.44 (silica gel, EtOAc/toluene = 1:3); ^1^H NMR (600 MH_Z_, CDCl_3_): δ 8.01–8.00 (m, 2H, Ar–H), 7.87–7.84 (m, 3H, Ar–H), 7.82–7.80 (m, 3H, Ar–H), 7.69–7.67 (m, 2H, Ar–H), 7.51–7.43 (m, 5H, Ar–H), 7.38–7.24 (m, 30H, Ar–H), 7.10–7.09 (m, 2H, Ar–H), 5.59 (dd, *J* = 10.2, 3.6 Hz, 1H), 5.39 (dd, *J* = 9.0, 8.4 Hz, 1H), 5.37 (s, 1H, Ph–CH), 5.33 (dd, *J* = 3.6, 1.8 Hz, 1H), 5.15 (dd, *J* = 10.2, 9.6 Hz, 1H), 5.10 (d, *J* = 11.4 Hz, 1H), 5.06 (d, *J* = 3.6 Hz, 1H, C1–H_α_), 5.04 (d, *J* = 11.4 Hz, 1H), 5.00 (d, *J* = 3.6 Hz, 1H, C1–H_α_), 4.96 (d, *J* = 1.2 Hz, 1H, C1–H_α_), 4.91 (d, *J* = 10.8 Hz, 1H), 4.85 (d, *J* = 7.8 Hz, 1H, C1–H_β_), 4.75–4.72 (m, 3H), 4.63 (d, *J* = 11.4 Hz, 1H), 4.57 (d, *J* = 12.0 Hz, 1H), 4.47 (d, *J* = 12.0 Hz, 1H), 4.43 (d, *J* = 10.8 Hz, 1H), 4.24–4.19 (m, 1H), 4.13–4.04 (m, 5H), 4.00 (dd, *J* = 9.6, 9.0 Hz, 1H), 3.98–3.95 (m, 1H), 3.79 (dd, *J* = 9.6, 9.0 Hz, 1H), 3.77–3.75 (m, 1H), 3.70–3.66 (m, 2H), 3.65–3.61 (m, 3H), 3.58–3.54 (m, 2H), 3.40 (dd, *J* = 10.2, 10.2 Hz, 1H), 3.38–3.29 (m, 2H), 3.17 (t, *J* = 7.2 Hz, 2H, –CH_2linker_), 2.57–2.53 (m, 1H, −C*H*_a_H_b_), 2.48–2.39 (m, 2H, –CH_2_), 2.32–2.26 (m, 1H, −CH_a_*H*_b_), 2.00 (s, 3H, –CH_3_), 1.73 (s, 3H, –CH_3_), 1.59–1.49 (m, 4H, 2-CH_2linker_), 1.38–1.32 (m, 2H, –CH_2linker_), 0.78 (d, *J* = 6.0 Hz, 3H, –CH_3_); ^13^C{^1^H} NMR (150 MHz, CDCl_3_): δ 205.7, 171.8170.3, 165.2, 164.5, 164.3, 138.3, 138.1, 137.9, 137.9, 136.8, 136.6, 133.3, 133.1, 133.0, 132.8, 130.0, 129.7, 129.6, 129.3, 129.1, 128.8, 128.5, 128.3, 128.3, 128.2, 128.2, 128.1, 128.1, 128.0, 127.9, 127.8, 127.8, 127.7, 127.6, 127.6, 126.2, 126.1, 125.7, 125.5, 125.4, 101.6, 101.4, 97.9, 95.9, 94.3, 80.6, 79.9, 79.1, 78.6, 77.8, 77.6, 76.4, 76.4, 75.6, 75.1, 75.0, 74.8, 73.4, 72.3, 71.3, 70.4, 70.3, 69.4, 68.8, 68.5, 68.4, 67.9, 66.5, 66.4, 61.9, 51.1, 37.7, 29.5, 29.1, 28.5, 27.8, 23.4, 20.4, 16.5; HRMS (ESI-TOF) *m*/*z*: [M + Na]^+^ calcd for C_103_H_107_N_3_O_26_Na, 1824.7035; found, 1824.7074. β-isomer: *R*_f_ = 0.63 (silica gel, EtOAc/toluene = 1:3); ^1^H NMR (600 MH_Z_, CDCl_3_): δ 8.01 (d, *J* = 7.2 Hz, 2H, Ar–H), 7.87–7.83 (m, 3H, Ar–H), 7.80 (d, *J* = 7.8 Hz, 2H, Ar–H), 7.75 (s, 1H, Ar–H), 7.66 (d, *J* = 7.8 Hz, 2H, Ar–H), 7.52–7.43 (m, 5H, Ar–H), 7.41–7.38 (m, 3H, Ar–H), 7.34–7.18 (m, 25H, Ar–H), 7.15–7.08 (m, 4H, Ar–H), 5.57 (dd, *J* = 10.2, 3.6 Hz, 1H), 5.41 (dd, *J* = 8.4, 8.4 Hz, 1H), 5.37 (s, 1H, Ph–CH), 5.30 (d, *J* = 2.4 Hz, 1H), 5.16 (dd, *J* = 10.2, 9.6 Hz, 1H), 5.06–5.01 (m, 2H), 4.97 (s, 1H, C1–H_α_), 4.93 (d, *J* = 11.4 Hz, 1H), 4.83 (d, *J* = 3.0 Hz, 1H, C1–H_α_), 4.78 (d, *J* = 10.8 Hz, 2H), 4.75 (d, *J* = 10.8 Hz, 1H), 4.71 (d, *J* = 7.8 Hz, 1H, C1–H_β_), 4.67 (d, *J* = 7.8 Hz, 1H, C1–H_β_), 4.65 (d, *J* = 10.8 Hz, 1H), 4.58 (d, *J* = 12.0 Hz, 1H), 4.47–4.44 (m, 2H), 4.21 (dd, *J* = 9.6, 6.0 Hz, 1H), 4.15–4.06 (m, 3H), 3.97 (dd, *J* = 9.6, 9.0 Hz, 1H), 3.79–3.57 (m, 9H), 3.34–3.38 (m, 2H), 3.36–3.32 (m, 2H), 3.25–3.23 (m, 1H), 3.20 (t, *J* = 6.6 Hz, 2H, –CH_2linker_), 2.57–2.52 (m, 1H, −CH_a_*H*_b_), 2.48–2.38 (m, 2H, –CH_2_), 2.31–2.26 (m, 1H, −CH_a_*H*_b_), 2.00 (s, 3H, –CH_3_), 1.85 (s, 3H, –CH_3_), 1.60–1.56 (m, 4H, 2-CH_2linker_), 1.46–1.42 (m, 2H, –CH_2linker_), 0.79 (d, *J* = 6.0 Hz, 3H, –CH_3_); ^13^C{^1^H} NMR (150 MHz, CDCl_3_): δ 205.7, 171.8, 170.3, 165.3, 164.6, 164.4, 138.4, 138.2, 138.1, 138.0, 136.8, 136.4, 133.3, 133.2, 133.1, 132.9, 129.9, 129.7, 129.6, 129.3, 129.2, 129.1, 128.8, 128.5, 128.4, 128.3, 128.3, 128.3, 128.2, 128.1, 127.9, 127.9, 127.8, 127.8, 127.8, 127.7, 127.6, 127.5, 127.4, 126.2, 126.1, 125.9, 125.7, 103.3, 101.6, 101.4, 98.7, 97.8, 82.7, 81.8, 81.4, 78.7, 78.3, 78.2, 77.8, 76.2, 75.6, 75.2, 75.0, 74.7, 73.4, 72.6, 71.3, 70.4, 70.2, 69.4, 68.6, 68.4, 67.8, 66.7, 66.5, 62.1, 51.3, 37.7, 29.5, 29.0, 28.6, 27.8, 23.4, 20.6, 16.6; HRMS (ESI-TOF) *m*/*z*: [M + Na]^+^ calcd for C_103_H_107_N_3_O_26_Na, 1824.7035; found, 1824.7147.

### Dibutyl 2,3-*O*-dibenzoyl-4-*O*-oxopentanoate-α-l-rhamnopyranosyl-(1→3)-2-*O*-benzoyl-4,6-*O*-benzylidene-β-d-glucopyranosyl-(1→4)-6-*O*-acetyl-2-*O*-benzyl-*3*-*O*-(2-naphthylmethyl)-α-d-glucopyranosyl Phosphate (**27a**)

*R*_f_ = 0.16 (silica gel, EtOAc/toluene = 1:3); ^1^H NMR (600 MH_Z_, CDCl_3_): δ 8.03 (d, *J* = 7.2 Hz, 2H, Ar–H), 7.87 (d, *J* = 8.4 Hz, 3H, Ar–H), 7.83 (s, 1H, Ar–H), 7.89 (d, *J* = 7.2 Hz, 2H, Ar–H), 7.65 (d, *J* = 7.2 Hz, 2H, Ar–H), 7.53–7.47 (m, 4H, Ar–H), 7.44 (t, *J* = 7.2 Hz, 1H, Ar–H), 7.39–7.36 (m, 3H, Ar–H), 7.34–7.26 (m, 11H, Ar–H), 7.24–7.23 (m, 3H, Ar–H), 5.79 (dd, *J* = 7.2, 4.2 Hz, 1H, C1–H_α_), 5.57 (dd, *J* = 10.2, 3.6 Hz, 1H), 5.44 (dd, *J* = 9.0, 8.4 Hz, 1H), 5.39 (s, 1H, Ph–CH), 5.30–5.29 (m, 1H), 5.15 (dd, *J* = 10.2, 10.2 Hz, 1H), 5.07 (s, 2H), 4.96 (s, 1H, C1–H_α_), 4.76 (d, *J* = 11.4 Hz, 1H), 4.74 (d, *J* = 8.4 Hz, 1H, C1–H_β_), 4.65 (d, *J* = 11.4 Hz, 1H), 4.23–4.18 (m, 1H), 4.16 (dd, *J* = 12.0, 3.0 Hz, 1H), 4.12–4.08 (m, 3H), 4.00–3.88 (m, 5H), 3.83–3.77 (m, 2H), 3.65 (dd, *J* = 9.6, 9.0 Hz, 1H), 3.57 (ddd, *J* = 9.0, 2.4, 2.4 Hz, 1H), 3.40–3.33 (m, 2H), 2.58–2.52 (m,1H, −CH_a_*H*_b_) 2.47–2.38 (m, 2H, –CH_2_), 2.31–2.25 (m, 1H, −CH_a_*H*_b_), 1.99 (s, 3H, –CH_3_), 1.85 (s, 3H, –CH_3_), 1.56–1.47 (m, 4H, 2-CH_2_), 1.31–1.21 (m, 4H, 2-CH_2_), 0.84 (t, *J* = 7.8 Hz, 3H, –CH_3_), 0.80 (t, *J* = 7.8 Hz, 3H, –CH_3_), 0.78 (d, *J* = 6.0 Hz, 3H, –CH_3_); ^13^C{^1^H} NMR (150 MHz, CDCl_3_): δ 205.7, 171.8, 170.2, 165.2, 164.6, 164.4, 137.5, 136.7, 136.4, 133.3, 133.2, 133.1, 132.9, 130.0, 129.7, 129.6, 129.2, 129.1, 128.7, 128.3, 128.2, 128.2, 128.1, 128.0, 128.0, 127.9, 127.8, 126.2, 125.9, 125.8, 125.7, 101.7, 101.5, 97.9, 94.5, 94.4, 79.1, 78.6, 78.5, 78.5, 76.3, 75.6, 74.7, 73.0, 71.3, 70.3, 70.1, 69.4, 68.4, 67.9, 67.8, 67.4, 67.3, 66.7, 66.5, 61.6, 37.7, 32.0, 32.0, 29.5, 27.8, 20.5, 18.5, 18.5, 16.6, 13.5, 13.4; ^31^P NMR (162 MH_Z_, CDCl_3_): δ −1.61; HRMS (ESI-TOF) *m*/*z*: [M + Na]^+^ calcd for C_79_H_87_O_24_PNa, 1473.5217; found, 1473.5254.

### 5-Azidopentyl 2,3-*O*-dibenzoyl-4-*O*-oxopentanoate-α-l-rhamnopyranosyl-(1→3)-2-*O*-benzoyl-4,6-*O*-benzylidene-β-d-glucopyranosyl-(1→4)-6-*O*-acetyl-2-*O*-benzyl-α-d-glucopyranosyl-(1→2)-3,4,6-*tri*-*O*-benzyl-α-d-glucopyranoside (**26**)

To a stirred solution of starting material **25** (2.60 g, 1.44 mmol, 1 equiv) in CH_2_Cl_2_/phosphate buffer. pH 7, (150 mL, 9:1 = *v*/*v*), 2,3-dichloro-5,6-dicyanobenzoquinone (720 mg, 3.17 mmol, 2.2 equiv) was added at 0 °C. The reaction mixture was vigorously stirred until TLC analysis indicated all the starting material had disappeared (3 h). Then, the reaction mixture was diluted with CH_2_Cl_2_ (50 mL) and washed with saturated aq NaHCO_3_ (40 mL) and brine (20 mL). The organic phase was washed with water until the solution became colorless; then, the separated organic layer was dried over MgSO_4_, filtered, and concentrated. The obtained residue was purified by silica gel column chromatography using EtOAc/toluene (1:4) as eluents to give compound **26** as a white powder (2.10 g, 88%). *R*_f_ = 0.43 (silica gel, EtOAc/toluene = 1:3); ^1^H NMR (600 MH_Z_, CDCl_3_): δ 7.97–7.96 (m, 2H, Ar–H), 7.80–7.79 (m, 2H, Ar–H), 7.67–7.65 (m, 2H, Ar–H), 7.51–7.48 (m, 3H, Ar–H), 7.46–7.43 (m, 1H, Ar–H), 7.38–7.22 (m, 28H, Ar–H), 7.09–7.08 (m, 2H, Ar–H), 5.63 (s, 1H, Ph–CH), 5.58 (dd, *J* = 10.2, 3.6 Hz, 1H), 5.41 (dd, *J* = 9.0, 8.4 Hz, 1H), 5.33 (dd, *J* = 3.6, 1.2 Hz, 1H), 5.16 (dd, *J* = 10.2, 9.6 Hz, 1H), 5.00 (d, *J* = 3.6 Hz, 1H, C1–H_α_), 4.99 (d, *J* = 3.0 Hz, 1H, C1–H_α_), 4.96 (d, *J* = 1.2 Hz, 1H, C1–H_α_), 4.88 (d, *J* = 10.8 Hz, 1H), 4.77 (d, *J* = 7.8 Hz, 1H, C1–H_β_), 4.74–4.69 (m, 3H), 4.56 (d, *J* = 12.0 Hz, 1H), 4.46–4.43 (m, 2H), 4.40 (d, *J* = 10.8 Hz, 1H), 4.28–4.23 (m, 1H), 4.21 (dd, *J* = 9.6, 9.0 Hz, 1H), 4.09 (dd, *J* = 9.6, 9.0 Hz, 1H), 3.99–3.94 (m, 3H), 3.88–3.84 (m, 3H), 3.81 (s, 1H), 3.76–3.73 (m, 1H), 3.69–3.60 (m, 5H), 3.56–3.51 (m, 1H), 3.49–3.46 (m, 1H), 3.40 (dd, *J* = 9.0, 3.0 Hz, 1H), 3.38–3.34 (m, 1H), 3.17 (t, *J* = 7.2 Hz, 2H, –CH_2linker_), 2.58–2.53 (m, 1H, −C*H*_a_H_b_), 2.48–2.39 (m, 2H, –CH_2_), 2.31–2.26 (m, 1H, −CH_a_*H*_b_), 2.00 (s, 3H, –CH_3_), 1.76 (s, 3H, –CH_3_), 1.57–1.47 (m, 4H, 2-CH_2linker_), 1.37–1.31 (m, 2H, –CH_2linker_), 0.82 (d, *J* = 6.0 Hz, 3H, –CH_3_); ^13^C{^1^H} NMR (150 MHz, CDCl_3_): δ 205.7, 171.8, 170.0, 165.3, 164.4, 138.2, 138.1, 138.0, 136.7, 133.2, 133.1, 129.9, 129.7, 129.6, 129.3, 129.2, 129.1, 129.0, 128.5, 128.4, 128.3, 128.3, 128.2, 128.2, 127.8, 127.8, 127.8, 127.6, 127.6, 126.2, 102.1, 101.9, 98.0, 95.9, 94.7, 81.6, 80.8, 78.5, 77.8, 76.5, 76.2, 75.9, 75.0, 74.3, 73.4, 72.2, 71.5, 71.3, 70.3, 70.2, 69.4, 68.5, 68.3, 67.9, 67.8, 67.0, 66.6, 61.8, 51.2, 37.7, 29.5, 29.0, 28.5, 27.8, 23.4, 20.5, 16.6; HRMS (ESI-TOF) *m*/*z*: [M + Na]^+^ calcd for C_92_H_99_N_3_O_26_Na, 1684.6409; found, 1684.6445.

### 5-Azidopentyl 2,3-*O*-dibenzoyl-α-l-rhamnopyranosyl-(1→3)-2-*O*-benzoyl-4,6-*O*-benzylidene-β-d-glucopyranosyl-(1→4)-[2,3-di-*O*-benzoyl-4-*O*-benzyl- α-l-rhamnopyranosyl-(1→3)]-6-*O*-acetyl-2-*O*-benzyl-α-d-glucopyranosyl-(1→2)-3,4,6-*tri*-*O*-benzyl-α-d-glucopyranoside (**10**)

To a stirred solution of starting material 9 (260 mg, 0.12 mmol, 1 equiv) in CH_2_Cl_2_ (3.5 mL), hydrazine hydrate (25 μL, 0.49 mmol, 4 equiv) dissolved in AcOH (0.32 mL) and pyridine (0.48 mL) were added at room temperature. Stirring was continued until TLC analysis indicated all the starting materials had disappeared (1 h). The reaction was quenched by acetone (0.2 mL). The solvent was removed, and the obtained residue was purified by silica gel column chromatography using EtOAc/*n*-hexane (1:2) as eluents to give compound 10 as a white powder (230 mg, 93%). *R*_f_ = 0.49 (silica gel, EtOAc/*n*-hexane = 2:3); ^1^H NMR (600 MH_Z_, CDCl_3_): δ 8.01–7.98 (m, 6H, Ar–H), 7.86–7.84 (m, 2H, Ar–H), 7.70–7.69 (m, 2H, Ar–H), 7.60–7.57 (m, 1H, Ar–H), 7.55–7.44 (m, 5H, Ar–H), 7.40–7.21 (m, 31H, Ar–H), 7.17–7.12 (m, 3H, Ar–H), 7.10–7.05 (m, 3H, Ar–H), 5.73 (dd, *J* = 3.0, 1.8 Hz, 1H), 5.69 (dd, *J* = 9.6, 3.6 Hz, 1H), 5.46 (d, *J* = 1.8 Hz, 1H, C1–H_α_), 5.44 (dd, *J* = 10.2, 3.6 Hz, 1H), 5.36–5.33 (m, 2H), 5.03 (d, *J* = 3.6 Hz, 1H, C1–H_α_), 4.96 (d, *J* = 3.6 Hz, 1H, C1–H_α_), 4.88 (d, *J* = 1.2 Hz, 1H, C1–H_α_), 4.86 (dd, *J* = 9.6, 6.6 Hz, 1H), 4.80 (dd, *J* = 10.2, 9.6 Hz, 2H), 4.73 (d, *J* = 10.2 Hz, 1H), 4.67 (d, *J* = 10.8 Hz, 1H), 4.64 (d, *J* = 10.8 Hz, 1H), 4.59–4.55 (m, 3H, C1–H_β_), 4.53 (d, *J* = 12.0 Hz, 1H), 4.43 (d, *J* = 12.0 Hz, 1H), 4.41 (dd, *J* = 10.8, 4.8 Hz, 1H), 4.36 (d, *J* = 10.8 Hz, 1H), 4.26 (s, 1H, Ph–CH), 4.22–4.18 (m, 2H), 4.05 (d, *J* = 12.0 Hz, 1H), 4.01–3.98 (m, 2H), 3.90 (dd, *J* = 9.6, 9.0 Hz, 1H), 3.82–3.78 (m, 4H), 3.70–3.54 (m, 7H), 3.46 (dd, *J* = 9.6, 9.6 Hz, 1H), 3.40 (dd, *J* = 9.6, 9.0 Hz, 1H), 3.36–3.32 (m, 2H), 3.08 (t, *J* = 7.2 Hz, 2H, –CH_2linker_), 2.41 (d, *J* = 5.4 Hz, 1H, OH), 2.03 (s, 3H, –CH_3_), 1.63 (d, *J* = 6.6 Hz, 3H, –CH_3_), 1.55–1.49 (m, 2H, –CH_2linker_), 1.47–1.42 (m, 2H, –CH_2linker_), 1.31–1.24 (m, 2H, –CH_2linker_), 0.87 (d, *J* = 6.0 Hz, 3H, –CH_3_); ^13^C{^1^H} NMR (150 MHz, CDCl_3_): δ 170.4, 167.4, 165.5, 165.3, 164.4, 164.4, 138.3, 138.2, 138.0, 137.8, 137.4, 137.1, 133.3, 133.2, 133.1, 133.0, 130.0, 130.0, 129.9, 129.8, 129.8, 129.7, 129.6, 129.4, 129.3, 128.8, 128.8, 128.7, 128.6, 128.5, 128.4, 128.4, 128.3, 128.2, 128.1, 127.9, 127.8, 127.7, 127.6, 127.6, 126.2, 101.2, 100.6, 98.2, 97.4, 95.9, 94.1, 80.5, 80.3, 79.8, 77.8, 77.6, 76.7, 76.2, 75.7, 75.0, 74.8, 74.3, 73.4, 73.1, 73.0, 72.7, 72.4, 70.9, 70.5, 70.3, 69.5, 68.9, 68.6, 68.1, 67.8, 67.7, 67.4, 61.8, 51.1, 29.0, 28.5, 23.4, 20.9, 18.1, 16.8; HRMS (ESI-TOF) *m*/*z*: [M + Na]^+^ calcd for C_114_H_117_N_3_O_30_Na, 2030.7614; found, 2030.7634.

### 5-Azidopentyl 2,3-*O*-dibenzoyl-4-O-(benzyloxy-[2-cyanoethoxy]-phosphono)-α-l-rhamnopyranosyl-(1→3)-2-*O*-benzoyl-4,6-*O*-benzylidene-β-d-glucopyranosyl-(1→4)-[2,3-di-*O*-benzoyl-4-*O*-benzyl-α-l-rhamnopyranosyl-(1→3)]-6-*O*-acetyl-2-*O*-benzyl-α-d-glucopyranosyl-(1→2)-3,4,6-*tri*-*O*-benzyl-α-d-glucopyranoside (**28**)

To a stirred solution of starting material **10** (848 mg, 0.42 mmol, 1 equiv) in CH_2_Cl_2_ (20 mL), benzyl 2-cyanoethyl *N*,*N*-diisopropylphosphoramidite^3^ (521 mg, 1.69 mmol, 4 equiv) and 1*H*-tetrazole (0.45 M in acetonitrile, 7.50 mL, 3.38 mmol, 8 equiv) were added at room temperature. Stirring was continued until TLC analysis indicated all the starting materials had disappeared (40 min). Then, the reaction was cooled to −20 °C, and *m*-CPBA (473 mg, 2.11 mmol, 5 equiv) was added. Stirring was resumed until TLC analysis indicated the starting materials had disappeared (20 min). Then, the reaction was quenched by saturated aq. NaHCO_3_ (15 mL), and the solution was extracted by CH_2_Cl_2_ (20 mL × 2). The separated organic layer was dried over MgSO_4_ and concentrated. The obtained residue was purified by silica gel column chromatography using EtOAc/*n*-hexane (1:1) as eluents to give a mixture of two phosphate diastereomers **28** (ratio = 1:1) as a white powder (935 mg, 99%). No attempt was made to separate this mixture of diastereomers, which was directly used for the next step. However, for data analysis, a portion of the mixture was subjected to column chromatography and separated two phosphate diastereomers.

Polar phosphate diastereomer: *R*_f_ = 0.24 (silica gel, EtOAc/hexane = 1:1); ^1^H NMR (600 MH_Z_, CDCl_3_): δ 8.01–7.96 (m, 6H, Ar–H), 7.88–7.87 (m, 2H, Ar–H), 7.66–7.65 (m, 2H, Ar–H), 7.60–7.58 (m, 1H, Ar–H), 7.55–7.50 (m, 2H, Ar–H), 7.47–7.43 (m, 3H, Ar–H), 7.40–7.19 (m, 34H, Ar–H), 7.16–7.13 (m, 4H, Ar–H), 7.11–7.05 (m, 4H, Ar–H), 6.94–6.93 (m, 2H, Ar–H), 5.73–5.68 (m, 3H), 5.47 (d, *J* = 1.2 Hz, 1H, C1–H_α_), 5.37 (dd, *J* = 9.6, 9.0 Hz, 1H), 5.34 (dd, *J* = 3.6, 1.2 Hz, 1H), 5.03 (d, *J* = 3.6 Hz, 1H, C1–H_α_), 4.97 (d, *J* = 3.6 Hz, 1H, C1–H_α_), 4.88–4.83 (m, 3H, C1–H_α_), 4.79 (d, *J* = 10.8 Hz, 1H), 4.77–4.73 (m, 2H, C1–H_β_), 4.67 (d, *J* = 10.8 Hz, 1H), 4.64 (d, *J* = 11.4 Hz, 1H), 4.59–4.56 (m, 3H), 4.53 (d, *J* = 12.0 Hz, 1H), 4.51–4.46 (m, 2H), 4.43 (d, *J* = 12.0 Hz, 1H) 4.41 (dd, *J* = 10.8, 4.2 Hz,1H), 4.36 (d, *J* = 10.8 Hz, 1H), 4.25 (s, 1H, Ph–CH), 4.23–4.17 (m, 3H), 4.05 (d, *J* = 11.4 Hz, 1H), 4.00 (dd, *J* = 9.6, 9.6 Hz, 1H), 3.86–3.75 (m, 6H), 3.69 (ddd, *J* = 9.6, 3.6, 1.8 Hz, 1H), 3.66–3.54 (m, 5H), 3.46 (dd, *J* = 9.6, 9.0 Hz, 1H), 3.42 (dd, *J* = 9.6 9.0 Hz, 1H), 3.36–3.32 (m, 2H), 3.08 (t, *J* = 7.2 Hz, 2H, –CH_2linker_), 2.32 (t, *J* = 6.6 Hz, 2H, –CH_2_), 2.03 (s, 3H, –CH_3_), 1.66 (d, *J* = 6.6 Hz, 3H, –CH_3_), 1.55–1.49 (m, 2H, –CH_2linker_), 1.47–1.42 (m, 2H, –CH_2linker_), 1.31–1.23 (m, 2H, –CH_2linker_), 0.90 (s, 3H, –CH_3_); ^13^C{^1^H} NMR (150 MHz, CDCl_3_): δ 170.4, 165.5, 165.3, 165.2, 164.3, 164.2, 138.2, 138.2, 138.0, 137.4, 137.1, 134.9, 134.9, 133.2, 133.1, 133.1, 130.0, 130.0, 129.8, 129.8, 129.7, 129.6, 129.4, 129.1, 128.9, 128.9, 128.7, 128.6, 128.6, 128.5, 128.5, 128.4, 128.4, 128.3, 128.3, 128.2, 128.1, 127.9, 127.9, 127.8, 127.7, 127.7, 127.6, 127.6, 126.3, 116.0, 101.2, 100.7, 97.8, 97.4, 95.9, 94.1, 80.5, 80.3, 79.9, 77.8, 77.7, 77.7, 77.6, 76.7, 76.6, 76.2, 75.7, 75.0, 74.8, 74.3, 73.4, 72.9, 72.7, 72.4, 70.9, 70.5, 70.3, 69.7, 69.7, 69.7, 69.5, 68.6, 68.2, 67.8, 67.6, 67.4, 66.7, 66.7, 61.8, 61.6, 61.5, 51.1, 29.0, 28.5, 23.4, 20.9, 19.1, 19.1, 18.1, 16.9; ^31^P NMR (162 MH_Z_, CDCl_3_): δ −1.83; HRMS (ESI-TOF) *m*/*z*: [M + Na]^+^ calcd for C_124_H_127_N_4_O_33_PNa, 2253.8012; found, 2253.8109. Nonpolar phosphate diastereomer: *R*_f_ = 0.29 (silica gel, EtOAc/hexane = 1:1); ^1^H NMR (600 MH_Z_, CDCl_3_): δ 8.01–7.96 (m, 6H, Ar–H), 7.94–7.92 (m, 2H, Ar–H), 7.67–7.65 (m, 2H, Ar–H), 7.60–7.58 (m, 1H, Ar–H), 7.55–7.45 (m, 5H, Ar–H), 7.40–7.20 (m, 35H, Ar–H), 7.17–7.05 (m, 9H, Ar–H), 5.75–5.73 (m, 2H), 5.69 (dd, *J* = 9.6, 3.0 Hz, 1H), 5.47 (d, *J* = 1.8 Hz, 1H, C1–H_α_), 5.37 (dd, *J* = 9.6, 8.4 Hz, 1H), 5.33 (dd, *J* = 3.6, 1.2 Hz, 1H), 5.03 (d, *J* = 3.6 Hz, 1H, C1–H_α_), 4.97–4.94 (m, 2H, C1–H_α_), 4.89–4.85 (m, 3H, C1–H_α_), 4.84 (d, *J* = 10.2 Hz, 1H), 4.79 (d, *J* = 10.8 Hz, 1H), 4.74 (d, *J* = 9.6 Hz, 1H), 4.68 (d, *J* = 10.8 Hz, 1H), 4.65 (d, *J* = 11.4 Hz, 1H), 4.59–4.49 (m, 5H, C1–H_β_), 4.45–4.40 (m, 2H), 4.36 (d, *J* = 10.8 Hz, 1H), 4.24 (s, 1H, Ph–CH), 4.23–4.17 (m, 3H), 4.05 (d, *J* = 11.4 Hz, 1H), 3.98 (dd, *J* = 9.6, 9.6 Hz, 1H), 3.91 (dd, *J* = 9.6, 9.6 Hz, 1H), 3.82–3.77 (m, 4H), 3.71–3.54 (m, 8H), 3.46 (dd, *J* = 9.6, 9.0 Hz, 1H), 3.41 (dd, *J* = 9.6, 9.0 Hz, 1H), 3.36–3.31 (m, 2H), 3.08 (t, *J* = 7.2 Hz, 2H, –CH_2linker_), 2.03 (s, 3H, –CH_3_), 2.00–1.91 (m, 2H, –CH_2_), 1.66 (d, *J* = 6.6 Hz, 3H, –CH_3_), 1.54–1.49 (m, 2H, –CH_2linker_), 1.47–1.42 (m, 2H, –CH_2linker_), 1.32–1.25 (m, 2H, –CH_2linker_), 0.86 (d, *J* = 6.6 Hz, 3H, –CH_3_); ^13^C{^1^H} NMR (150 MHz, CDCl_3_): δ 170.4, 165.5, 165.3, 165.2, 164.3, 164.2, 138.2, 138.2, 138.0, 137.9, 137.4, 137.1, 135.2, 135.2, 133.4, 133.2, 133.1, 130.0, 130.0, 129.8, 129.8, 129.7, 129.7, 129.4, 129.0, 128.9, 128.8, 128.7, 128.7, 128.6, 128.5, 128.4, 128.4, 128.4, 128.3, 128.2, 128.1, 127.9, 127.9, 127.8, 127.7, 127.7, 127.6, 127.6, 126.2, 115.8, 101.2, 100.6, 97.8, 97.4, 95.9, 94.1, 80.5, 80.3, 80.3, 79.9, 77.8, 77.8, 77.7, 77.6, 76.7, 76.2, 75.7, 75.0, 74.8, 74.2, 73.4, 72.9, 72.7, 72.4, 70.9, 70.5, 70.3, 69.7, 69.7, 69.5, 68.6, 68.2, 67.8, 67.6, 67.4, 66.7, 66.7, 61.8, 61.5, 61.5, 51.1, 29.0, 28.5, 23.4, 20.9, 18.6, 18.5, 18.1, 16.8; ^31^P NMR (162 MH_Z_, CDCl_3_): δ −1.62; HRMS (ESI-TOF) *m*/*z*: [M + Na]^+^ calcd for C_124_H_127_N_4_O_33_PNa, 2253.8012; found, 2253.8095.

### 5-Azidopentyl 2,3-*O*-dibenzoyl-4-O-(benzyloxy-phosphono)-α-l-rhamnopyranosyl-(1→3)-2-*O*-benzoyl-4,6-*O*-benzylidene-β-d-glucopyranosyl-(1→4)-[2,3-di-*O*-benzoyl-4-*O*-benzyl-α-l-rhamnopyranosyl-(1→3)]-6-*O*-acetyl-2-*O*-benzyl-α-d-glucopyranosyl-(1→2)-3,4,6-*tri*-*O*-benzyl-α-d-glucopyranoside (**11**)

To a stirred solution of starting material **28** (1.84 g, 0.82 mmol, 1 equiv) in CH_2_Cl_2_ (25 mL), TBAOH (40% in water, 1.07 g, 1.65 mmol, 2 equiv) in water (25 mL) was added at room temperature. Stirring was continued until TLC analysis indicated all starting materials had disappeared (4 h). Then, the solution was diluted with CH_2_Cl_2_ (50 mL), and the reaction was extracted by CH_2_Cl_2_ (50 mL × 2). The separated organic layer was dried over MgSO_4_ and concentrated. The obtained residue was purified by silica gel column chromatography using MeOH/CH_2_Cl_2_ (1:10) as eluents to give compound acid with tetrabutylammonium salt **11** as a white powder (1.68 g, 94%) for further step. *R*_f_ = 0.29 (silica gel, MeOH/CH_2_Cl_2_ = 1:10); ^1^H NMR (600 MH_Z_, CDCl_3_): δ 8.00–7.99 (m, 4H, Ar–H), 7.87 (br, 2H, Ar–H), 7.67 (br, 2H, Ar–H), 7.59 (t, *J* = 7.2 Hz, 2H, Ar–H), 7.53 (t, *J* = 7.2 Hz, 1H, Ar–H), 7.48–7.45 (m, 3H, Ar–H), 7.38 (t, *J* = 7.8 Hz, 2H, Ar–H), 7.31–7.07 (m, 35H, Ar–H), 6.94–6.84 (m, 9H, Ar–H), 5.72 (s, 1H), 5.69–5.67 (m, 2H), 5.46 (s, 1H, C1–H_α_), 5.32 (br, 2H), 5.02 (d, *J* = 1.2 Hz, 1H, C1–H_α_), 4.97–4.96 (m, 2H, 2C1–H_α_), 4.83 (br, 1H), 4.79–4.75 (m, 2H), 4.69–4.63 (m, 4H), 4.60–4.52 (m, 6H, C1–H_β_), 4.45 (d, *J* = 12.0 Hz, 1H), 4.38–4.36 (m, 2H), 4.28–4.13 (m, 4H, Ph–CH), 4.02–4.00 (m, 2H), 3.91 (dd, *J* = 9.0, 9.0 Hz, 1H), 3.78–3.76 (m, 4H), 3.71–3.64 (m, 2H) 3.63–3.58 (m, 3H), 3.54–3.53 (m, 2H), 3.46 (dd, *J* = 9.6, 9.0 Hz, 1H), 3.38–3.30 (m, 3H), 3.08 (t, *J* = 7.2 Hz, 2H, –CH_2linker_), 2.01 (s, 3H, –CH_3_), 1.59 (br, 3H, –CH_3_), 1.56–1.50 (m, 2H, –CH_2linker_), 1.47–1.43 (m, 2H, –CH_2linker_), 1.32–1.26 (m, 2H, –CH_2linker_), 0.79 (br, 3H, –CH_3_); ^13^C{^1^H} NMR (150 MHz, CDCl_3_): δ 170.3, 165.5, 165.3, 164.7, 164.0, 138.3, 138.1, 138.0, 137.5, 137.3, 136.9, 133.1, 133.0, 132.9, 132.8, 129.9, 129.8, 129.7, 129.6, 129.5, 129.0, 128.8, 128.7, 128.5, 128.4, 128.4, 128.4, 128.3, 128.1, 128.0, 127.9, 127.8, 127.7, 127.6, 127.5, 127.2, 126.9, 126.9, 125.9, 101.2, 100.5, 98.0, 97.4, 95.8, 94.0, 80.5, 80.2, 79.6, 77.8, 77.5, 76.7, 76.1, 75.6, 74.9, 74.6, 74.4, 73.4, 72.9, 72.6, 72.4, 70.9, 70.8, 70.3, 69.5, 68.5, 68.0, 67.8, 67.6, 67.5, 67.3, 61.8, 51.1, 29.0, 28.4, 23.1, 20.8, 18.0, 17.4; ^31^P NMR (162 MH_Z_, CDCl_3_): δ −2.93; HRMS (ESI-TOF) *m*/*z*: [M + Na]^+^ calcd for C_121_H_124_N_3_O_33_PNa, 2200.7747; found, 2200.7739.

### 5-Azidopentyl 2,3,4,6-tetra-*O*-benzyl-α-d-glucopyranosyl-(1→[phenylmethyl]-phosphate→4)-2,3-*O*-dibenzoyl-α-l-rhamnopyranosyl-(1→3)-2-*O*-benzoyl-4,6-*O*-benzylidene-β-d-glucopyranosyl-(1→4)-[2,3-di-*O*-benzoyl-4-*O*-benzyl-α-l-rhamnopyranosyl-(1→3)]-6-*O*-acetyl-2-*O*-benzyl-α-d-glucopyranosyl-(1→2)-3,4,6-*tri*-*O*-benzyl-α-d-glucopyranoside (**29**)

A mixture of acceptor **11** (300 mg, 0.14 mmol, 1 equiv), donor^28^**6** (490 mg, 0.76 mmol, 5.5 equiv), and activated pulverized 4 Å molecular sieves (800 mg) in anhydrous CH_2_Cl_2_ (8 mL) was stirred under an argon atmosphere for 1 h. The reaction was cooled to 0 °C, and NIS (201 mg, 0.89 mmol, 6.5 equiv) and TfOH (0.5 M in Et_2_O, 0.33 mL, 0.17 mmol, 1.2 equiv) were added. Stirring was continued until TLC analysis indicated all starting materials had disappeared (16 h). Then, the reaction was quenched by Et_3_N (0.2 mL) and filtered through a pad of Celite. The filtrate was quenched with 20% aq. Na_2_S_2_O_3_ (5 mL) and washed with saturated aq. NaHCO_3_ (3 mL) and brine (2 mL). The separated organic layer was dried over MgSO_4_ and concentrated. The obtained residue was purified by silica gel column chromatography using EtOAc/*n*-hexane (2:3) as eluents to give a mixture of phosphate diastereomers **29** as a white powder (260 mg, 70%). No attempt was made to separate this mixture of diastereomers, which was directly used for the next step. However, for data analysis, a portion of the mixture was subjected to column chromatography and separated two phosphate diastereomers.

Polar phospate diastereomer: *R*_f_ = 0.16 (silica gel, EtOAc/Toluene = 1:5); ^1^H NMR (600 MH_Z_, CDCl_3_): δ 8.02–7.98 (m, 4H, Ar–H), 7.95–7.93 (m, 2H, Ar–H), 7.89–7.88 (m, 2H, Ar–H), 7.60–7.57 (m, 3H, Ar–H), 7.54 (dd, *J* = 7.8, 7.2 Hz, 1H, Ar–H), 7.47–7.37 (m, 6H, Ar–H), 7.35–7.34 (m, 2H, Ar–H), 7.31–7.25 (m, 17H, Ar–H), 7.23–7.13 (m, 32H, Ar–H), 7.11–7.01 (m, 12H, Ar–H), 5.73 (dd, *J* = 3.0, 1.8 Hz, 1H), 5.69–5.66 (m, 2H), 5.59 (dd, *J* = 7.2, 3.0 Hz, 1H, C1–H_α_), 5.47 (s, 1H, C1–H_α_), 5.38–5.35 (m, 2H), 5.03 (d, *J* = 3.0 Hz, 1H, C1–H_α_), 4.97 (d, *J* = 3.6 Hz, 1H, C1–H_α_), 4.92–4.76 (m, 6H), 4.71–4.62 (m, 6H), 4.59–4.34 (m, 12H, C1–H_β_), 4.25–4.17 (m, 5H, Ph–CH), 4.04 (d, *J* = 11.4 Hz, 1H), 3.96 (dd, *J* = 9.6, 9.0 Hz, 1H), 3.91 (dd, *J* = 9.6, 9.0 Hz, 1H), 3.82–3.74 (m, 4H) 3.70–3.69 (m, 2H), 3.65–3.54 (m, 7H), 3.47–3.42 (m, 2H), 3.39–3.30 (m, 4H), 3.23–3.21 (m, 1H), 3.08 (t, *J* = 6.6 Hz, 2H, –CH_2linker_), 2.03 (s, 3H, –CH_3_), 1.68 (d, *J* = 6.0 Hz, 3H, –CH_3_), 1.54–1.49 (m, 2H, –CH_2linker_), 1.47–1.42 (m, 2H, –CH_2linker_), 1.32–1.27 (m, 2H, –CH_2linker_), 0.87 (d, *J* = 6.6 Hz, 3H, –CH_3_); ^13^C{^1^H} NMR (150 MHz, CDCl_3_): δ 170.4, 165.6, 165.4, 165.3, 164.4, 164.1, 138.5, 138.2, 138.2, 138.0, 138.0, 137.8, 137.5, 137.4, 137.0, 135.8, 135.7, 133.2, 133.1, 133.0, 132.7, 130.0, 129.9, 129.9, 129.8, 129.8, 129.7, 129.6, 129.1, 129.0, 128.7, 128.5, 128.4, 128.4, 128.3, 128.3, 128.3, 128.3, 128.2, 128.1, 127.9, 127.9, 127.8, 127.8, 127.7, 127.6, 127.6, 127.5, 127.4, 126.0, 101.2, 100.4, 98.0, 97.4, 95.9, 95.4, 95.4, 94.1, 80.8, 80.6, 80.3, 79.9, 79.2, 79.1, 77.6, 77.5, 76.3, 76.2, 75.7, 75.4, 75.0, 74.7, 74.3, 73.4, 73.3, 72.8, 72.8, 72.7, 72.7, 72.4, 70.9, 70.3, 70.2, 69.5, 69.4, 69.3, 68.6, 68.1, 67.8, 67.7, 67.7, 67.4, 66.8, 66.7, 61.9, 51.1, 29.0, 28.5, 23.4, 20.9, 18.2, 17.0; ^31^P NMR (162 MH_Z_, CDCl_3_): δ −2.11; HRMS (ESI-TOF) *m*/*z*: [M+2Na]^2+^ calcd for C_155_H_158_N_3_O_38_PNa_2_, 1373.0023; found, 1373.0071. Nonpolar phosphate diastereomer: *R*_f_ = 0.26 (silica gel, EtOAc/hexane = 5:1); ^1^H NMR (600 MH_Z_, CDCl_3_): δ 8.02–7.95 (m, 8H, Ar–H), 7.63–7.58 (m, 3H, Ar–H), 7.55–7.53 (m, 1H, Ar–H), 7.48–7.44 (m, 3H, Ar–H), 7.41–7.38 (m, 3H, Ar–H), 7.35–7.34 (m, 2H, Ar–H), 7.30–7.04 (m, 58H, Ar–H), 6.95 (d, *J* = 7.2 Hz, 2H, Ar–H), 5.86 (dd, *J* = 6.0, 3.0 Hz, 1H, C1–H_α_), 5.76–5.73 (m, 2H), 5.69 (dd, *J* = 9.6, 3.0 Hz, 1H), 5.48 (d, *J* = 1.2 Hz, 1H, C1–H_α_), 5.40–5.37 (m, 2H), 5.04 (d, *J* = 3.6 Hz, 1H, C1–H_α_), 4.98 (d, *J* = 3.0 Hz, 1H, C1–H_α_), 4.92 (s, 1H, C1–H_α_), 4.90–4.85 (m, 2H), 4.84–4.62 (m, 12H), 4.60–4.53 (m, 5H, C1–H_β_), 4.47–4.40 (m, 3H), 4.41 (dd, *J* = 10.8, 4.8 Hz, 1H), 4.37 (d, *J* = 10.8 Hz, 1H), 4.31 (d, *J* = 12.6 Hz, 1H), 4.28–4.19 (m, 4H, Ph–CH), 4.05 (d, *J* = 12.0 Hz, 1H), 4.00 (dd, *J* = 9.6, 9.0 Hz, 1H), 4.92 (dd, *J* = 9.6, 9.0 Hz, 1H), 3.83–3.78 (m, 6H), 3.70 (ddd, *J* = 10.2, 3.6, 1.8 Hz, 1H), 3.68–3.52 (m, 8H), 3.47 (dd, *J* = 9.6, 9.6 Hz, 1H), 3.40 (dd, *J* = 9.6, 9.0 Hz, 1H), 3.37–3.30 (m, 2H), 3.25 (dd, *J* = 10.8, 1.2 Hz, 1H), 3.09 (t, *J* = 7.2 Hz, 2H, –CH_2linker_), 2.03 (s, 3H, –CH_3_), 1.68 (d, *J* = 6.0 Hz, 3H, –CH_3_), 1.56–1.50 (m, 2H, –CH_2linker_), 1.48–1.43 (m, 2H, –CH_2linker_), 1.33–1.25 (m, 2H, –CH_2linker_), 0.93 (d, *J* = 6.6 Hz, 3H, –CH_3_); ^13^C{^1^H} NMR (150 MHz, CDCl_3_): δ 170.4, 165.6, 165.5, 165.3, 164.4, 164.2, 138.5, 138.2, 138.2, 138.1, 138.0, 137.8, 137.7, 137.4, 137.4, 137.0, 135.6, 135.5, 133.2, 133.0, 133.0, 130.0, 132.9, 130.0, 130.0, 130.0, 129.8, 129.8, 129.7, 129.5, 129.4, 129.2, 128.9, 128.8, 128.6, 128.4, 128.4, 128.4, 128.3, 128.3, 128.2, 128.1, 128.1, 128.0, 127.9, 127.9, 127.8, 127.8, 127.8, 127.7, 127.7, 127.6, 127.1, 126.1, 101.2, 100.4, 97.8, 97.4, 95.8, 95.6, 95.5, 94.1, 81.2, 80.5, 80.3, 79.8, 78.7, 78.7, 77.7, 77.6, 76.7, 76.6, 76.4, 76.2, 75.6, 75.1, 75.0, 74.7, 74.3, 73.4, 73.4, 72.9, 72.7, 72.5, 72.5, 72.4, 70.9, 70.6, 70.3, 70.1, 69.5, 68.7, 68.7, 68.6, 68.1, 67.8, 67.6, 67.6, 67.4, 66.9, 66.9, 61.8, 51.1, 29.0, 28.5, 23.4, 20.8, 18.1, 17.0; ^31^P NMR (162 MH_Z_, CDCl_3_): δ −0.97; HRMS (ESI-TOF) *m*/*z*: [M + Na]^+^ calcd for C_155_H_158_N_3_O_38_PNa, 2723.0153; found, 2723.0214.

### 5-Azidopentyl 6-*O*-acetyl-2,3,4-tri-*O*-benzyl-α-d-glucopyranosyl-(1→2)-4,6-*O*-benzylidene-3-*O*-benzyl-α-d-glucopyranosyl-(1→[phenylmethyl]-phosphate→4)-2,3-*O*-dibenzoyl-α-l-rhamnopyranosyl-(1→3)-2-*O*-benzoyl-4,6-*O*-benzylidene-β-d-glucopyranosyl-(1→4)-[2,3-di-*O*-benzoyl-4-*O*-benzyl-α-l-rhamnopyranosyl-(1→3)]-6-*O*-acetyl-2-*O*-benzyl-α-d-glucopyranosyl-(1→2)-3,4,6-*tri*-*O*-benzyl-α-d-glucopyranoside (**30**)

A mixture of acceptor **11** (223 mg, 0.10 mmol, 1 equiv), donor **7** (356 mg, 0.36 mmol, 3.5 equiv), and activated pulverized 4 Å molecular sieves (600 mg) in anhydrous CH_2_Cl_2_ (6 mL) was stirred under an argon atmosphere for 1 h. The reaction was cooled to 0 °C; then, NIS (104 mg, 0.46 mmol, 4.5 equiv) and TfOH (0.5 M in Et_2_O, 0.20 mL, 0.10 mmol, 1 equiv) were added. Stirring was continued at room temperature until TLC analysis indicated all starting materials had disappeared (20 h). Upon completion, it was quenched by Et_3_N (0.2 mL) and filtered through a pad of Celite. The filtrate was then quenched with 20% aq. Na_2_S_2_O_3_ (5 mL) and washed with saturated aq. NaHCO_3_ (3 mL) and brine (2 mL). The separated organic layer was dried over MgSO_4_ and concentrated. The obtained residue was purified by silica gel column chromatography using EtOAc/*n*-hexane (2:3) as eluents to give a mixture of phosphate diastereomer **30** (230 mg, 75%) as a white powder and also recovered donor **7** (115 mg) as a white powder. α diastereomer: *R*_f_ = 0.69 (silica gel, EtOAc/toluene = 1:3); ^1^H NMR (600 MH_Z_, CDCl_3_): δ 8.00–7.98 (m, 4H, Ar–H), 7.96–7.93 (m, 4H, Ar–H), 7.60–7.58 (m, 3H, Ar–H), 7.55–7.53 (m, 1H, Ar–H), 7.48–7.45 (m, 3H, Ar–H), 7.40–7.37 (m, 3H, Ar–H), 7.36–7.32 (m, 4H, Ar–H), 7.30–7.25 (m, 21H, Ar–H), 7.23–6.98 (m, 42H, Ar–H), 5.72 (dd, *J* = 3.0, 1.8 Hz, 1H), 5.68 (dd, *J* = 9.6, 3.0 Hz, 1H), 5.55 (dd, *J* = 9.6, 3.6 Hz, 1H), 5.49 (dd, *J* = 7.2, 3.0 Hz, 1H, C1–H_α_), 5.47 (d, *J* = 1.2 Hz, 1H), 5.39 (dd, *J* = 3.6, 1.2 Hz, 1H, C1–H_α_), 5.37–5.36 (m, 2H, Ph–CH), 5.11 (dd, *J* = 12.0, 7.2 Hz, 1H), 5.03 (d, *J* = 3.0 Hz, 1H, C1–H_α_), 4.96 (d, *J* = 3.6 Hz, 1H, C1–H_α_), 4.90 (d, *J* = 3.0 Hz, 1H, C1–H_α_), 4.88 (d, *J* = 0.6 Hz, 1H, C1–H_α_), 4.86–4.77 (m, 7H), 4.69–4.63 (m, 6H), 4.60–4.55 (m, 5H, C1–H_β_), 4.53 (d, *J* = 12.0 Hz, 1H), 4.45–4.42 (m, 2H), 4.40 (dd, *J* = 10.8, 4.8 Hz, 1H), 4.35 (d, *J* = 10.8 Hz, 1H), 4.28 (s, 1H, Ph–CH), 4.23–4.19 (m, 4H), 4.05–3.87 (m, 7H), 3.81–3.75 (m, 5H), 3.70–3.54 (m, 7H), 3.47–3.29 (m, 9H), 3.08 (t, *J* = 7.2 Hz, 2H, –CH_2linker_), 2.02 (s, 3H, –CH_3_), 1.91 (s, 3H, –CH_3_), 1.63 (d, *J* = 6.0 Hz, 3H, –CH_3_), 1.55–1.49 (m, 2H, –CH_2linker_), 1.47–1.42 (m, 2H, –CH_2linker_), 1.32–1.26 (m, 2H, –CH_2linker_), 0.92 (d, *J* = 6.0 Hz, 3H, –CH_3_); ^13^C{^1^H} NMR (150 MHz, CDCl_3_): δ 170.5, 170.4, 165.5, 165.3, 164.4, 164.1, 138.5, 138.2, 138.2, 138.1, 138.0, 137.8, 137.6, 137.4, 137.2, 137.1, 136.0, 136.0, 133.2, 133.0, 133.0, 132.8, 130.1, 130.0, 129.9, 129.8, 129.8, 129.7, 129.5, 129.1, 129.0, 128.7, 128.7, 128.6, 128.6, 128.5, 128.5, 128.4, 128.4, 128.3, 128.3, 128.2, 128.1, 128.0, 128.0, 127.9, 127.8, 127.8, 127.7, 127.7, 127.6, 127.5, 126.1, 126.0, 101.4, 101.1, 100.6, 98.1, 97.4, 95.9, 95.1, 94.0, 94.0, 81.9, 81.6, 80.6, 80.3, 79.5, 79.3, 77.6, 77.6, 76.7, 76.6, 76.0, 75.8, 75.7, 75.5, 75.3, 75.0, 74.9, 74.3, 74.2, 74.1, 73.4, 73.2, 72.9, 72.7, 72.4, 70.9, 70.3, 70.3, 70.1, 69.5, 69.5, 69.4, 68.9, 68.6, 68.3, 68.1, 67.8, 67.8, 67.4, 66.7, 64.2, 62.4, 61.9, 51.1, 29.0, 28.5, 23.4, 20.9, 20.8, 18.1, 16.9; ^31^P NMR (162 MH_Z_, CDCl_3_): δ −0.71; HRMS (ESI-TOF) *m*/*z*: [M+2Na]^2+^ calcd for C_170_H_174_N_3_O_44_PNa_2_, 1519.0496; found, 1519.0514.

### Dibutyl 2,3-*O*-dibenzoyl-4-*O*-oxopentanoate-α-l-rhamnopyranosyl-(1→3)-2-*O*-benzoyl-4,6-*O*-benzylidene-β-d-glucopyranosyl-(1→4)-6-*O*-acetyl-2-*O*-benzyl-*3*-*O*-(2-naphthylmethyl)-β-d-glucopyranosyl Phosphate (**27b**)

A mixture of the compound **24a** (1.0 g, 0.70 mmol, 1 equiv), dibutyl phosphate (0.42 mL, 2.11 mmol, 3 equiv), and activated pulverized 4 Å molecular sieves (2 g) in anhydrous CH_2_Cl_2_ (30 mL) was stirred under an argon atmosphere for 1 h. The reaction was cooled to 0 °C; then, NIS (316 mg, 1.41 mmol, 2 equiv) and TfOH (0.5 M in Et_2_O, 0.42 mL, 0.211 mmol, 0.3 equiv) were added. Stirring was continued until TLC analysis indicated all starting materials have disappeared (16 h). Upon completion, it was quenched by saturated aq. NaHCO_3_ (0.2 mL) and filtered through a pad of Celite. The filtrate was quenched with 20% aq. Na_2_S_2_O_3_ (10 mL) and washed with saturated aq. NaHCO_3_ (3 mL) and brine (3 mL). The separated organic layer was dried over MgSO_4_ and concentrated. The obtained residue was purified by silica gel column chromatography using EtOAc/*n*-hexane (1:1) as eluents to give compound **27** as a white powder (α/β = 1:3 mixture 930 mg, 91%). α isomer same as before; β isomer; *R*_f_ = 0.40 (silica gel, EtOAc/*n*-hexane = 1:1); ^1^H NMR (600 MH_Z_, CDCl_3_): δ 8.02–8.00 (m, 2H, Ar–H), 7.87–7.83 (m, 3H, Ar–H), 7.80–7.79 (m, 2H, Ar–H), 7.76 (s, 1H, Ar–H), 7.66–7.65 (m, 2H, Ar–H), 7.53–7.40 (m, 8H, Ar–H), 7.34–7.24 (m, 11H, Ar–H), 7.23–7.21 (m, 3H, Ar–H), 5.57 (dd, *J* = 10.2, 3.6 Hz, 1H), 5.42 (dd, *J* = 9.0, 8.4 Hz, 1H), 5.38 (s, 1H, Ph–CH), 5.30 (dd, *J* = 3.6, 1.8 Hz, 1H), 5.17 (dd, *J* = 10.2, 9.6 Hz, 1H), 5.09–5.07 (m, 2H, C1–H_α_), 5.00–4.98 (m, 2H, C1–H_α_), 4.83 (d, *J* = 11.4 Hz, 1H), 4.75 (d, *J* = 11.4 Hz, 1H), 4.72 (d, *J* = 7.8 Hz, 1H, C1–H_β_), 4.25–4.19 (m, 2H), 4.14–4.07 (m, 3H), 4.02–3.90 (m, 4H), 3.83 (t, *J* = 9.6 Hz, 1H), 3.70 (t, *J* = 9.0 Hz, 1H), 3.63 (t, *J* = 9.0 Hz, 1H), 3.50 (dd, *J* = 8.4, 7.8 Hz, 1H), 3.43 (ddd, *J* = 9.0, 4.2, 1.8 Hz, 1H), 3.39–3.35 (m, 2H), 2.58–2.52 (m, 1H, −C*H*_a_H_b_), 2.47–2.38 (m, 2H, –CH_2_), 2.30–2.25 (m, 1H, −CH_a_*H*_b_), 1.99 (s, 3H, –CH_3_), 1.87 (s, 3H, –CH_3_), 1.59–1.50 (m, 4H, 2-CH_2_), 1.34–1.23 (m, 4H, 2-CH_2_), 0.85 (t, *J* = 7.2 Hz, 3H, –CH_3_), 0.82–0.80 (m, 6H, 2-CH_3_); ^13^C{^1^H} NMR (150 MHz, CDCl_3_): δ 205.7, 171.8, 170.1, 165.2, 164.6, 164.4, 137.9, 136.7, 136.1, 133.3, 133.3, 133.1, 132.9, 129.9, 129.7, 129.6, 129.2, 129.2, 129.1, 128.7, 128.4, 128.2, 128.2, 128.1, 128.0, 127.9, 127.8, 127.6, 126.2, 126.0, 125.8, 125.7, 101.7, 101.4, 98.3, 98.3, 97.8, 92.4, 81.2, 81.2, 78.6, 77.1, 76.1, 75.7, 74.8, 74.6, 73.2, 71.3, 70.4, 49.4, 68.4, 67.7, 67.7, 67.6, 67.6, 66.7, 66.5, 61.6, 37.7, 32.1, 32.1, 32.0, 29.5, 27.8, 20.6, 18.5, 18.5, 16.6, 13.5, 13.5; ^31^P NMR (162 MH_Z_, CDCl_3_): δ −1.70; HRMS (ESI-TOF) *m*/*z*: [M + Na]^+^ calcd for C_79_H_87_O_24_PNa, 1473.5217; found, 1473.5265.

### (2-Methyl-5-*tert*-butylphenyl) 2,3-*O*-dibenzoyl-4-*O*-oxopentanoate-α-l-rhamnopyranosyl-(1→3)-2-*O*-benzoyl-4,6-*O*-benzylidene-β-d-glucopyranosyl-(1→4)-6-*O*-acetyl-2-*O*-benzyl-α-d-glucopyranosyl-(1→2)-3-*O*-benzyl-4,6-*O*-benzylidene-1-thio-β-d-glucopyranoside (**35**)

A mixture of acceptor **33** (120 mg, 0.23 mmol, 1 equiv), donor **27a,b** (435 mg, 0.30 mmol, 1.3 equiv), and activated pulverized 4 Å molecular sieves (500 mg) in anhydrous CH_2_Cl_2_ (5 mL) was stirred under an argon atmosphere for 1 h. The reaction was cooled to −40 °C; then, TMSOTf (54.4 μL, 0.30 mmol, 1.3 equiv. with respect to acceptor) was added, and the reaction was warmed to −20 °C. Stirring was continued until TLC analysis indicated all starting materials had disappeared (1 h). Upon completion, it was quenched by Et_3_N (0.2 mL) and filtered through a pad of Celite. The solvent was removed, and the obtained residue was purified by silica gel column chromatography using EtOAc/*n*-hexane (2:3) as eluents to give a mixture of compounds as a white powder (285 mg) (product/aglycon transfer = 1.2:1) for further step.

To a stirred solution of compound **34** (285 mg) in a mixture of CH_2_Cl_2_/phosphate buffer pH 7 (8.8 mL, 9:1 = *v*/*v*), 2,3-dichloro-5,6-dicyanobenzoquinone (73.4 mg, 0.32 mmol, 1.4 equiv. with respect to first step acceptor) was added at 0 °C. The reaction mixture was vigorously stirred until TLC analysis indicated all starting material had disappeared (3 h). Then, the reaction mixture was diluted with CH_2_Cl_2_ (10 mL) and washed with satuated aq. NaHCO_3_ (10 mL) and brine (5 mL). The organic phase was washed with water until the solution became colorless; then, the separated organic layer was dried over MgSO_4_, filtered, and concentrated. The obtained residue was purified by silica gel column chromatography using EtOAc/toluene (1:6) as eluents to give the targeted compounds (tetrasaccharide **35** 135 mg, 36% and trisaccharide **12b** 100 mg, 34%; 2 steps) as a white powder. **35**: *R*_f_ = 0.51 (silica gel, EtOAc/toluene = 1:4); ^1^H NMR (600 MH_Z_, CDCl_3_): δ 7.87–7.86 (m, 2H, Ar–H), 7.79–7.77 (m, 2H, Ar–H), 7.65–7.63 (m, 2H, Ar–H), 7.51–7.42 (m, 5H, Ar–H), 7.38–7.18 (m, 20H, Ar–H), 7.16–7.14 (m, 4H, Ar–H), 7.11–7.09 (m, 2H, Ar–H), 7.04 (d, *J* = 8.4 Hz, 1H, Ar–H), 5.75 (d, *J* = 3.6 Hz, 1H, C1–H_α_), 5.64 (s, 1H, Ph–CH), 5.56 (dd, *J* = 9.6, 3.6 Hz, 1H), 5.47 (s, 1H, Ph–CH), 5.43 (dd, *J* = 8.4, 7.8 Hz, 1H), 5.30 (dd, *J* = 3.6, 1.2 Hz, 1H), 5.15 (dd, *J* = 10.2, 9.6 Hz, 1H), 4.98–4.95 (m, 2H, C1–H_α_, C1–H_β_), 4.80 (d, *J* = 8.4 Hz, 1H, C1–H_β_), 4.74 (d, *J* = 11.4 Hz, 1H), 4.72–4.66 (m, 3H), 4.47 (dd, *J* = 10.2, 4.8 Hz, 1H, C1–H_β_), 4.30–4.21 (m, 4H), 4.06 (dd, *J* = 9.6, 9.0 Hz, 1H), 3.96–3.79 (m, 6H), 3.75–3.65 (m, 4H), 3.52–3.48 (m, 2H), 3.41 (dd, *J* = 9.6, 3.6 Hz, 1H), 2.58–2.52 (m, 1H, −C*H*_a_H_b_), 2.47–2.38 (m, 2H, –CH_2_), 2.32–2.26 (m, 1H, −CH_a_*H*_b_), 2.25 (s, 3H, –CH_3_), 2.00 (s, 3H, –CH_3_), 1.77 (s, 3H, –CH_3_), 1.27 (s, 9H, *t*Bu-CH_3_), 0.82 (d, *J* = 6.0 Hz, 3H, –CH_3_); ^13^C{^1^H} NMR (150 MHz, CDCl_3_): δ 205.8, 171.8, 170.1, 165.2, 164.4, 164.4, 149.6, 137.8, 137.6, 137.0, 136.7, 134.5, 133.2, 133.1, 132.4, 130.0, 129.9, 129.7, 129.6, 129.3, 129.2, 129.1, 129.0, 128.5, 128.5, 128.4, 128.2, 128.2, 128.2, 127.8, 127.8, 127.5, 126.2, 126.0, 126.0, 125.6, 124.0, 101.9, 101.9, 101.3, 98.0, 95.8, 86.1, 81.9, 81.2, 80.3, 78.6, 78.4, 76.2, 75.7, 74.4, 74.2, 72.9, 71.3, 71.2, 70.3, 69.5, 69.4, 68.4, 68.4, 67.7, 66.9, 66.6, 61.7, 37.7, 34.5, 31.3, 31.2, 29.7, 29.5, 27.8, 20.6, 19.9, 16.6; HRMS (ESI-TOF) *m*/*z*: [M + Na]^+^ calcd for C_91_H_96_O_25_SNa, 1643.5854; found, 1643.5818.

### (2-Methyl-5-*tert*-butylphenyl) 2,3-*O*-dibenzoyl-4-*O*-oxopentanoate-α-l-rhamnopyranosyl-(1→3)-2-*O*-benzoyl-4,6-*O*-benzylidene-β-d-glucopyranosyl-(1→4)-6-*O*-acetyl-2-*O*-benzyl-*3*-*O*-(2-naphthylmethyl)-1-thio-α-d-glucopyranoside (**12b**)

*R*_f_ = 0.41 (silica gel, EtOAc/toluene = 1:4); ^1^H NMR (600 MH_Z_, CDCl_3_): δ 8.00–7.99 (m, 2H, Ar–H), 7.80–7.78 (m, 2H, Ar–H), 7.65–7.64 (m, 2H, Ar–H), 7.51–7.47 (m, 3H, Ar–H), 7.44 (dd, *J* = 7.2, 7.2 Hz, 1H, Ar–H), 7.40–7.23 (m, 16H, Ar–H), 7.12 (dd, *J* = 7.8, 1.8 Hz, 1H, Ar–H), 7.06 (d, *J* = 7.8 Hz, 1H, Ar–H), 5.65 (s, 1H, Ph–CH), 5.58 (dd, *J* = 10.2, 3.6 Hz, 1H), 5.49–5.46 (m, 2H, C1–H_α_), 5.30 (dd, *J* = 3.6, 1.2 Hz, 1H), 5.17 (dd, *J* = 10.2, 10.2 Hz, 1H), 5.00 (s, 1H, C1–H_α_), 4.78–4.71 (m, 3H, C1–H_β_), 4.47 (dd, *J* = 10.2, 4.8 Hz, 1H), 4.28–4.20 (m, 3H), 4.04–3.98 (m, 2H), 3.93–3.87 (m, 3H), 3.75 (s, 1H), 3.70–3.64 (m, 2H), 3.50 (dd, *J* = 3.6, 2.4 Hz, 1H), 2.59–2.53 (m, 1H, −C*H*_a_H_b_), 2.48–2.39 (m, 2H, –CH_2_), 2.34 (s, 3H, –CH_3_), 2.31–2.25 (m, 1H, −CH_a_*H*_b_), 2.00 (s, 3H, –CH_3_), 1.79 (s, 3H, –CH_3_), 1.19 (s, 9H, *t*Bu-CH_3_), 0.84 (d, *J* = 6.0 Hz, 3H, –CH_3_); ^13^C{^1^H} NMR (150 MHz, CDCl_3_): δ 205.8, 171.8, 170.0, 165.2, 164.6, 164.4, 149.6, 137.6, 136.6, 136.4, 133.3, 133.1, 132.8, 130.0, 129.8, 129.7, 129.6, 129.3, 129.2, 129.1, 129.0, 128.6, 128.4, 128.3, 128.3, 128.2, 128.2, 128.0, 127.9, 127.9, 126.2, 124.4, 101.9, 101.9, 98.0, 86.0, 81.1, 78.5, 77.9, 76.0, 74.1, 72.4, 72.1, 71.3, 70.3, 69.3, 68.5, 68.3, 67.1, 66.6, 61.9, 37.7, 34.3, 31.2, 29.5, 27.8, 20.6, 20.2, 16.7; HRMS (ESI-TOF) *m*/*z*: [M + Na]^+^ calcd for C_71_H_76_O_20_SNa, 1303.4543; found, 1303.4540.

### (2-Methyl-5-*tert*-butylphenyl) 2,3-*O*-dibenzoyl-4-*O*-oxopentanoate-α-l-rhamnopyranosyl-(1→3)-2-*O*-benzoyl-4,6-*O*-benzylidene-β-d-glucopyranosyl-(1→4)-[2,3-di-*O*-benzoyl-4-*O*-benzyl-α-l-rhamnopyranosyl-(1→3)]-6-*O*-acetyl-2-*O*-benzyl-α-d-glucopyranosyl-(1→2)-3-*O*-benzyl-4,6-*O*-benzylidene-1-thio-β-d-glucopyranoside (**8**)

A mixture of acceptor **35** (185 mg, 0.11 mmol, 1 equiv), donor **13** (104 mg, 0.17 mmol, 1.5 equiv), and activated pulverized 4 Å molecular sieves (400 mg) in anhydrous CH_2_Cl_2_ (5 mL) was stirred under an argon atmosphere for 1 h. The reaction was cooled to −30 °C, and TMSOTf (5.17 μL, 29.5 μmol, 0.25 equiv. with respect to acceptor) was added. Stirring was continued until TLC analysis indicated all starting materials had disappeared (40 min). Then, the reaction was quenched by Et_3_N (0.5 mL) and filtered through a pad of Celite. The solvent was removed, and the obtained residue was purified by silica gel column chromatography using EtOAc/*n*-hexane (2:3) as eluents to give compound **8** as a white powder (230 mg, 98%). *R*_f_ = 0.57 (silica gel, EtOAc/*n*-hexane = 1:1); ^1^H NMR (600 MH_Z_, CDCl_3_): δ 8.03–8.00 (m, 4H, Ar–H), 7.81–7.79 (m, 4H, Ar–H), 7.62–7.58 (m, 3H, Ar–H), 7.55 (dd, *J* = 7.2, 7.2 Hz, 1H, Ar–H), 7.48–7.21 (m, 29H, Ar–H), 7.14–7.09 (m, 4H, Ar–H), 7.01–6.98 (m, 3H, Ar–H), 6.91–6.89 (m, 3H, Ar–H), 6.84–6.82 (m, 2H, Ar–H), 5.80 (dd, *J* = 3.0, 1.8 Hz, 1H), 5.73–5.71 (m, 2H, C1–H_α_), 5.66 (dd, *J* = 10.2, 3.6 Hz, 1H), 5.42–5.38 (m, 3H, Ph–CH, C1–H_α_), 5.28 (dd, *J* = 3.6, 1.2 Hz, 1H), 5.16 (dd, *J* = 10.2, 9.6 Hz, 1H), 4.94 (dq, *J* = 9.6, 6.0 Hz, 1H), 4.90 (s, 1H, C1–H_α_), 4.88 (d, *J* = 9.6 Hz, 1H), 4.83 (d, *J* = 9.6 Hz, 1H), 4.73 (d, *J* = 9.6 Hz, 1H), 4.64 (d, *J* = 10.8 Hz, 1H), 4.60 (d, *J* = 7.8 Hz, 1H, C1–H_β_), 4.48 (d, *J* = 9.6 Hz, 1H), 4.46–4.42 (m, 2H, C1–H_β_), 4.36–4.35 (m, 1H), 4.29 (dd, *J* = 12.0, 2.4 Hz, 1H), 4.24–4.16 (m, 5H, Ph–CH), 4.02–3.97 (m, 2H), 3.88–3.79 (m, 4H), 3.70–3.61 (m, 4H), 3.45 (dd, *J* = 9.6, 9.0 Hz, 1H), 3.40–3.33 (m, 2H), 2.83–2.57 (m, 1H, −C*H*_a_H_b_), 2.53–2.45 (m, 2H, –CH_2_), 2.38–2.33 (m, 1H, −CH_a_*H*_b_), 2.20 (s, 3H, –CH_3_), 2.14 (s, 3H, –CH_3_), 2.01 (s, 3H, –CH_3_), 1.79 (d, *J* = 6.0 Hz, 3H, –CH_3_), 1.23 (s, 9H, *t*Bu-CH_3_), 0.84 (d, *J* = 6.0 Hz, 3H, –CH_3_); ^13^C{^1^H} NMR (150 MHz, CDCl_3_): δ 205.8, 171.8, 170.5, 165.6, 165.3, 165.2, 164.3, 164.1, 149.5, 137.9, 137.1, 137.0, 136.8, 136.7, 134.1, 133.1, 133.0, 133.0, 132.5, 130.0, 129.9, 129.8, 129.7, 129.6, 129.4, 129.2, 129.1, 129.0, 128.8, 128.6, 128.5, 128.4, 128.2, 128.2, 128.2, 128.1, 127.8, 127.7, 126.2, 126.1, 124.8, 123.8, 101.4, 101.0, 100.5, 97.9, 97.7, 95.7, 85.6, 82.0, 80.8, 79.9, 77.8, 77.8, 76.7, 76.2, 76.0, 74.5, 74.2, 73.3, 73.0, 72.8, 72.5, 71.7, 70.9, 70.4, 69.4, 69.1, 69.0, 68.6, 68.1, 67.6, 67.4, 66.3, 61.9, 37.7, 34.5, 31.3, 31.2, 29.5, 27.9, 21.0, 19.8, 18.2, 16.6; HRMS (ESI-TOF) *m*/*z*: [M + Na]^+^ calcd for C_118_H_120_O_31_SNa, 2087.7426; found, 2087.7429.

### 5-Azidopentyl 2,3-*O*-dibenzoyl-4-*O*-oxopentanoate-α-l-rhamnopyranosyl-(1→3)-2-*O*-benzoyl-4,6-*O*-benzylidene-β-d-glucopyranosyl-(1→4)-[2,3-di-*O*-benzoyl-4-*O*-benzyl-α-l-rhamnopyranosyl-(1→3)]-6-*O*-acetyl-2-*O*-benzyl-α-d-glucopyranosyl-(1→2)-3-*O*-benzyl-4,6-*O*-benzylidene-α-d-glucopyranosyl-(1→[phenylmethyl]-phosphate→4)-2,3-*O*-dibenzoyl-α-l-rhamnopyranosyl-(1→3)-2-*O*-benzoyl-4,6-*O*-benzylidene-β-d-glucopyranosyl-(1→4)-[2,3-di-*O*-benzoyl-4-*O*-benzyl-α-l-rhamnopyranosyl-(1→3)]-6-*O*-acetyl-2-*O*-benzyl-α-d-glucopyranosyl-(1→2)-3,4,6-*tri*-*O*-benzyl-α-d-glucopyranoside (**36**)

A mixture of acceptor **11** (116 mg, 0.053 mmol, 1 equiv), donor **8** (300 mg, 0.16 mmol, 3 equiv), and activated pulverized 4 Å molecular sieves (400 mg) in anhydrous CH_2_Cl_2_ (4 mL) was stirred under an argon atmosphere for 1 h. The reaction was cooled to 0 °C; then, NIS (47.9 mg, 0.21 mmol, 4 equiv) and TfOH (0.5 M in Et_2_O, 95.8 μL, 0.048 mmol, 0.9 equiv) were added. Stirring was continued at room temperature until TLC analysis indicated all starting materials had disappeared (40 h). Then, the reaction was quenched by Et_3_N (0.2 mL) and filtered through a pad of Celite. The filtrate was quenched with 20% aq. Na_2_S_2_O_3_ (5 mL) and washed with saturated aq. NaHCO_3_ (3 mL) and brine (2 mL). The separated organic layer was dried over MgSO_4_ and concentrated. The obtained residue was purified by silica gel column chromatography using EtOAc/*n*-hexane (1:1) as eluents and a second column eluted by EtOAc/toluene = 1:3 to give a mixture of compound **36** (175 mg, 81%) and recovered donor **8** (56 mg) as a white powder. α diastereomer: *R*_f_ = 0.41 (silica gel, EtOAc/toluene = 1:3); ^1^H NMR (600 MH_Z_, CDCl_3_): δ 8.00–7.95 (m, 8H, Ar–H), 7.92–7.90 (m, 3H, Ar–H), 7.80–7.78 (m, 3H, Ar–H), 7.63–7.53 (m, 8H, Ar–H), 7.49–7.41 (m, 7H, Ar–H), 7.40–7.25 (m, 31H, Ar–H), 7.24–6.97 (m, 49H, Ar–H), 6.90 (dd, *J* = 7.2, 7.2 Hz, Ar–H), 5.70–5.61 (m, 5H), 5.47–5.45 (m, 2H, C1–H_α_), 5.36–5.31 (m, 5H, 2C1–H_α_), 5.25 (d, *J* = 3.6 Hz, 1H), 5.21 (s, 1H, Ph–CH), 5.14 (dd, *J* = 10.2, 10.2 Hz, 1H), 5.00 (d, *J* = 3.0 Hz, 1H, C1–H_α_), 4.95 (d, *J* = 3.0 Hz, 1H, C1–H_α_), 4.93–4.87 (m, 2H, C1–H_α_), 4.86–4.75 (m, 5H, C1–H_α_), 4.73–4.62 (m, 8H, C1–H_α_), 4.57–4.51 (m, 6H, 2C1–H_β_), 4.48 (d, *J* = 12.0 Hz, 1H), 4.43–4.33 (m, 5H), 4.23–4.09 (m, 10H, 2Ph–CH), 4.03 (dd, *J* = 10.8, 2.4 Hz, 1H), 3.99–3.94 (m, 3H), 3.89 (dd, *J* = 9.6, 9.0 Hz, 1H), 3.82–3.73 (m, 7H), 3.69–3.67 (m, 1H), 3.65–3.52 (m, 7H), 3.48–3.37 (m, 4H), 3.36–3.25 (m, 4H), 3.15–3.10 (m, 2H), 3.08 (t, *J* = 7.2 Hz, 2H, –CH_2linker_), 2.62–2.56 (m, 1H, −C*H*_a_H_b_), 2.51–2.44 (m, 2H, –CH_2_), 2.37–2.31 (m, 1H, −CH_a_*H*_b_), 2.08 (s, 3H, –CH_3_), 2.00 (s, 3H, –CH_3_), 1.72 (d, *J* = 6.6 Hz, 3H, –CH_3_), 1.59 (d, *J* = 6.0 Hz, 3H, –CH_3_), 1.54–1.48 (m, 2H, –CH_2linker_), 1.46–1.41 (m, 2H, –CH_2linker_), 1.32–1.26 (m, 2H, –CH_2linker_), 0.82 (d, *J* = 6.0 Hz, 3H, –CH_3_), 0.74 (d, *J* = 6.6 Hz, 3H, –CH_3_); ^13^C{^1^H} NMR (150 MHz, CDCl_3_): δ 205.8, 171.8, 170.4, 170.4, 165.5, 165.5, 165.3, 165.3, 165.3, 165.1, 164.4, 164.3, 164.1, 164.0, 138.2, 138.2, 138.0, 137.9, 137.7, 137.4, 137.3, 137.2, 137.1, 137.0, 135.5, 135.5, 133.1, 133.0, 133.0, 132.8, 130.0, 129.9, 129.9, 129.8, 129.8, 129.7, 129.7, 129.6, 129.4, 129.2, 129.1, 129.0, 128.9, 128.8, 128.7, 128.7, 128.6, 128.6, 128.5, 128.5, 128.4, 128.4, 128.4, 128.3, 128.2, 128.2, 128.1, 128.1, 128.1, 128.0, 127.9, 127.8, 127.8, 127.7, 127.7, 127.6, 127.5, 126.2, 126.1, 126.0, 101.3, 101.2, 101.0, 100.6, 100.5, 97.9, 97.4, 97.4, 95.9, 94.4, 94.0, 93.9, 93.8, 82.0, 80.5, 80.3, 79.8, 79.4, 79.2, 77.7, 77.6, 76.7, 76.6, 76.4, 76.3, 75.9, 75.7, 75.0, 74.9, 74.3, 74.2, 73.4, 73.1, 73.0, 72.3, 72.7, 72.6, 72.4, 71.7, 71.7, 70.9, 70.3, 70.2, 70.1, 69.6, 69.6, 69.5, 69.4, 68.6, 68.2, 68.1, 67.8, 67.7, 67.6, 67.5, 67.4, 66.6, 66.3, 64.2, 61.8, 61.6, 51.1, 37.7, 29.5, 29.0, 28.5, 27.9, 23.4, 21.0, 20.9, 18.2, 18.1, 16.9, 16.6; ^31^P NMR (162 MH_Z_, CDCl_3_): δ −1.50; HRMS (ESI-TOF) *m*/*z*: [M + NaH]^2+^ calcd for C_228_H_228_N_3_O_64_PNa_2_, 2043.2191; found, 2043.2038.

### [5-Azidopentyl-(phenylmethyl)-phosphate] 2,3-*O*-dibenzoyl-4-*O*-oxopentanoate-α-l-rhamnopyranosyl-(1→3)-2-*O*-benzoyl-4,6-*O*-benzylidene-β-d-glucopyranosyl-(1→4)-[2,3-di-*O*-benzoyl-4-*O*-benzyl-α-l-rhamnopyranosyl-(1→3)]-6-*O*-acetyl-2-*O*-benzyl-α-d-glucopyranosyl-(1→2)-3-*O*-benzyl-4,6-*O*-benzylidene-α-d-glucopyranoside (**38**)

To a stirred solution of starting material **37** (13.1 mg, 0.039 mmol, 1 equiv) in CH_2_Cl_2_ (0.5 mL), a solution of TBAOH (40%, 50.3 mg, 0.078 mmol, 2 equiv) in water (0.5 mL) was added at room temperature. Stirring was continued until TLC analysis indicated all starting materials had disappeared (4 h). Then, the reaction was diluted with CH_2_Cl_2_ (2 mL) and extracted by CH_2_Cl_2_ (2 mL × 2). The separated organic layer was dried over MgSO_4_ and concentrated. The obtained residue was purified by silica gel column chromatography using MeOH/CH_2_Cl_2_ (1:10) as eluents to give compound acid with tetrabutylammonium salt as a white powder (11.6 mg) for further step. *R*_f_ = 0.17 (silica gel, MeOH/CH_2_Cl_2_ = 1:8); HRMS (ESI-TOF) *m*/*z*: [M + Na]^+^ calcd for C_12_H_18_N_3_O_4_PNa, 322.0927; found, 322.0938. A mixture of acceptor (11.6 mg, 0.039 mmol, 1 equiv), donor **8** (200 mg, 0.097 mmol, 2.5 equiv), and activated pulverized 4 Å molecular sieves (250 mg) in anhydrous CH_2_Cl_2_ (2.5 mL) was stirred under an argon atmosphere for 1 h. The reaction was cooled to 0 °C and NIS (30.5 mg, 0.14 mmol, 3.5 equiv), and TfOH (0.5 M in Et_2_O, 61.9 μL, 0.034 mmol, 0.8 equiv) was added. Stirring was continued at room temperature until TLC analysis indicated all starting materials had disappeared (20 h). Then, the reaction was quenched by Et_3_N (0.1 mL) and filtered through a pad of Celite. The filtrate was quenched with 20% aq. Na_2_S_2_O_3_ (2 mL) and washed with saturated aq. NaHCO_3_ (1.5 mL) and brine (1 mL). The separated organic layer was dried over MgSO_4_ and concentrated. The obtained residue was purified by silica gel column chromatography using EtOAc/*n*-hexane (2:3) as eluents to give compound **38** as a white powder (35 mg, 41%). The α isomer is approximately 1/1 (A and B) mixtures of diastereomers at phosphorus. *R*_f_ = 0.31 (silica gel, EtOAc/hexane = 1:1); ^1^H NMR (600 MH_Z_, CDCl_3_): δ 8.06–7.97 (m, 4H, Ar–H), 7.84–7.79 (m, 4H, Ar–H), 7.65–7.61 (m, 3H, Ar–H), 7.55–7.19 (m, 35H, Ar–H), 7.17–7.06 (m, 9H, Ar–H), 5.75–5.71 (m, 2H, A + BC1-H_α_), 5.70 (s, 1H), 5.64 (dd, *J* = 10.2, 3.6 Hz, 1H), 5.43–5.36 (m, 4H, A + BC1-H_α_, A + BPh–CH), 5.27 (d, *J* = 1.8 Hz, 1H), 5.15 (dd, *J* = 10.2, 9.6 Hz, 1H), 5.02–4.47 (m, 10H, 2A + BC1-H_α_, A + BC1-H_β_), 4.43–4.40 (m, 1H), 4.25–4.09 (m, 8H, A + BPh–CH), 4.00 (dd, *J* = 9.6, 9.0 Hz, 1H), 3.94–3.73 (m, 7H), 3.66–3.40 (m, 5H), 3.35–3.32 (m, 1H), 3.14 (t, *J* = 7.2 Hz, 1H, A-CH_2linker_), 2.98 (td, *J* = 6.6, 4.2 Hz, 1H, B–CH_2linker_), 2.62–2.57 (m, 1H, −C*H*_a_H_b_), 2.52–2.45 (m, 2H, –CH_2_), 2.37–2.32 (m, 1H, −CH_a_*H*_b_), 2.10 (s, 3H, –CH_3_), 2.00 (s, 3H, –CH_3_), 1.79–1.76 (m, 3H, –CH_3_), 1.57–1.53 (m, 1H, A-CH_2linker_), 1.50–1.45 (m, 1H, A-CH_2linker_), 1.36–1.22 (m, 3H, 3B–CH_2linker_), 1.15–1.09 (m, 1H, A-CH_2linker_), 0.76 (d, *J* = 6.0 Hz, 3H, –CH_3_); ^13^C{^1^H} NMR (150 MHz, CDCl_3_): δ 205.8, 171.8, 170.5, 165.5, 165.5, 165.3, 165.2, 164.3, 164.1, 137.9, 137.9, 137.3, 137.3, 137.2, 137.2, 137.1, 137.1, 137.1, 135.7, 135.7, 135.6, 135.5, 133.3, 133.2, 133.1, 133.0, 133.0, 133.0, 133.0, 129.9, 129.9, 129.8, 129.8, 129.7, 129.6, 129.4, 129.2, 129.0, 128.8, 128.7, 128.6, 128.6, 128.5, 128.5, 128.4, 128.3, 128.3, 128.2, 128.2, 128.2, 128.1, 128.0, 128.0, 128.0, 127.8, 127.7, 127.7, 127.6, 126.2, 126.1, 126.1, 126.1, 101.5, 101.4, 101.0, 100.5, 97.9, 97.5, 97.4, 95.6, 95.6, 94.8, 94.7, 82.2, 79.9, 79.6, 77.8, 76.6, 76.3, 76.3, 75.8, 75.8, 75.7, 75.6, 74.3, 74.3, 74.2, 73.4, 73.3, 73.2, 73.2, 72.9, 72.9, 72.8, 72.6, 71.7, 71.1, 71.0, 70.3, 69.8, 69.7, 69.4, 69.2, 69.2, 69.0, 68.6, 68.5, 68.1, 68.1, 67.6, 67.6, 67.5, 67.5, 66.3, 64.2, 64.0, 61.6, 51.1, 51.0, 37.7, 29.7, 29.6, 28.3, 28.1, 27.9, 22.6, 22.4, 20.9, 18.3, 16.6; HRMS (ESI-TOF) *m*/*z*: [M + Na]^+^ calcd for C_119_H_122_N_3_O_35_PNa, 2206.7489; found, 2206.7454.

### 5-Azidopentyl 2,3-*O*-dibenzoyl-4-*O*-oxopentanoate-α-l-rhamnopyranosyl-(1→3)-2-*O*-benzoyl-4,6-*O*-benzylidene-β-d-glucopyranoside (**39**)

A mixture of 5-azidopenan-1-ol (51 mg, 0.40 mmol, 2 equiv), donor **19** (200 mg, 0.20 mmol, 1 equiv), and activated pulverized 4 Å molecular sieves (200 mg) in anhydrous CH_2_Cl_2_ (2 mL) was stirred under an argon atmosphere for 1 h. The reaction was cooled to 0 °C; then, NIS (90 mg, 0.40 mmol, 2 equiv) and TfOH (0.5 M in Et_2_O, 0.12 mL, 0.06 mmol, 0.3 equiv) were added. Stirring was continued until TLC analysis indicated all starting materials had disappeared (16 h). Upon completion, the reaction was quenched by Et_3_N (0.2 mL) and filtered through a pad of Celite. The filtrate was quenched with 20% aq. Na_2_S_2_O_3_ (5 mL) and washed with saturated aq. NaHCO_3_ (3 mL) and brine (2 mL). The separated organic layer was dried over MgSO_4_ and concentrated. The obtained residue was purified by silica gel column chromatography using EtOAc/toluene (1:5) as eluents to give compound **39** as a white powder (105 mg, 55%), and the starting material (40 mg, b.r.s.m. 69%) was recovered. *R*_f_ = 0.39 (silica gel, EtOAc/*n*-hexane = 1:2); ^1^H NMR (600 MH_Z_, CDCl_3_): δ 8.01–8.00 (m, 2H, Ar–H), 7.81–7.79 (m, 2H, Ar–H), 7.70–7.68 (m, 2H, Ar–H), 7.51–7.47 (m, 3H, Ar–H), 7.45–7.42 (m, 2H, Ar–H), 7.38–7.31 (m, 7H, Ar–H), 7.29–7.26 (m, 2H, Ar–H), 5.63 (s, 1H, Ph–CH), 5.60 (dd, *J* = 10.2, 3.6 Hz, 1H), 5.38 (dd, *J* = 9.0, 8.4 Hz, 1H), 5.35 (dd, *J* = 3.6, 1.8 Hz, 1H), 5.20 (dd, *J* = 10.2, 10.2 Hz, 1H), 5.06 (d, *J* = 1.2 Hz, 1H, C1–H_α_), 4.62 (d, *J* = 7.8 Hz, 1H, C1–H_β_), 4.41 (dd, *J* = 10.2, 4.8 Hz, 1H), 4.31–4.27 (m, 1H), 4.19 (dd, *J* = 9.0, 8.4 Hz, 1H), 3.90–3.82 (m, 3H), 3.57 (ddd, *J* = 9.6, 9.6, 4.8 Hz, 1H), 3.45 (ddd, *J* = 9.6, 7.2, 5.4 Hz, 1H), 2.99–2.95 (m, 1H, −C*H*_a_H_blinker_), 2.92–2.88 (m, 1H, −CH_a_*H*_blinker_), 2.59–2.53 (m, 1H, −C*H*_a_H_b_), 2.48–2.39 (m, 2H, –CH_2_), 2.31–2.26 (m, 1H, −CH_a_*H*_b_), 2.00 (s, 3H, –CH_3_), 1.56–1.44 (m, 2H, –CH_2linker_), 1.42–1.35 (m, 2H, –CH_2linker_), 1.28–1.18 (m, 2H, –CH_2linker_), 0.87 (d, *J* = 6.0 Hz, 3H, –CH_3_); ^13^C{^1^H} NMR (150 MHz, CDCl_3_): δ 205.8, 171.9, 165.3, 164.8, 164.5, 137.0, 133.1, 133.0, 129.8, 129.7, 129.6, 129.5, 129.3, 129.2, 128.3, 128.3, 128.2, 128.2, 126.2, 101.8, 101.7, 97.7, 78.9, 76.1, 74.4, 71.4, 70.5, 69.8, 69.4, 68.7, 66.7, 66.5, 51.1, 37.7, 29.5, 28.9, 28.3, 27.9, 23.0, 16.7; HRMS (ESI-TOF) *m*/*z*: [M + Na]^+^ calcd for C_50_H_53_N_3_O_15_Na, 958.3369; found, 958.3396.

### 5-Azidopentyl 2,3-*O*-dibenzoyl-α-l-rhamnopyranosyl-(1→3)-2-*O*-benzoyl-4,6-*O*-benzylidene-β-d-glucopyranoside (**40**)

To a stirred solution of starting material **39** (284 mg, 0.30 mmol, 1 equiv) in CH_2_Cl_2_ (10 mL), hydrazine hydrate (59 μL, 1.21 mmol, 4 equiv) in AcOH (1.0 mL) and pyridine (1.50 mL) were added at room temperature. The reaction mixture was vigorously stirred until TLC analysis indicated all starting material had disappeared (1 h). Upon completion, the reaction was quenched by acetone (0.2 mL). The solvent was removed, and the obtained residue was purified by silica gel column chromatography using EtOAc/*n*-hexane (1:3) as eluents to give compound **40** as a white powder (240 mg, 94%). *R*_f_ = 0.46 (silica gel, EtOAc/*n*-hexane = 1:3); ^1^H NMR (600 MH_Z_, CDCl_3_): δ 8.02–8.00 (m, 2H, Ar–H), 7.83–7.82 (m, 2H, Ar–H), 7.74–7.72 (m, 2H, Ar–H), 7.52–7.50 (m, 3H, Ar–H), 7.48–7.45 (m, 1H, Ar–H), 7.43–7.41(m, 1H, Ar–H), 7.37–7.27 (m, 9H, Ar–H), 5.62 (s, 1H, Ph–CH), 5.44 (dd, *J* = 10.2, 3.6 Hz, 1H), 5.36 (dd, *J* = 9.0, 7.8 Hz, 1H), 5.34 (dd, *J* = 3.6, 1.8 Hz, 1H), 5.03 (d, *J* = 1.8 Hz, 1H, C1–H_α_), 4.62 (d, *J* = 7.8 Hz, 1H, C1–H_β_), 4.40 (dd, *J* = 10.8, 4.8 Hz, 1H), 4.18 (dd, *J* = 9.6, 9.0 Hz, 1H), 4.13–4.09 (m, 1H), 3.90–3.86 (m, 2H), 3.81 (dd, *J* = 9.6, 9.0 Hz, 1H) 3.70 (dd, *J* = 9.6, 9.6 Hz, 1H), 3.57 (ddd, *J* = 9.6, 9.6, 4.8 Hz, 1H), 3.45 (ddd, *J* = 9.6, 7.2, 5.4 Hz, 1H), 2.99–2.95 (m, 1H, −C*H*_a_H_blinker_), 2.92–2.87 (m, 1H, −CH_a_*H*_blinker_), 2.30 (br, 1H, OH), 1.56–1.44 (m, 2H, –CH_2linker_), 1.43–1.35 (m, 2H, –CH_2linker_), 1.29–1.18 (m, 2H, –CH_2linker_), 0.99 (d, *J* = 6.0 Hz, 3H, –CH_3_); ^13^C{^1^H} NMR (150 MHz, CDCl_3_): δ 167.1, 164.8, 164.6, 136.9, 133.3, 133.1, 133.1, 129.8, 129.8, 129.6, 129.4, 129.4, 129.2, 129.2, 128.3, 128.2, 126.2, 101.8, 101.7, 98.0, 79.0, 76.5, 74.5, 72.8, 72.1, 70.8, 69.8, 69.0, 68.7, 66.8, 51.1, 28.9, 28.3, 23.0, 17.0; HRMS (ESI-TOF) *m*/*z*: [M + Na]^+^ calcd for C_45_H_47_N_3_O_13_Na, 860.3001; found, 860.3056.

### 5-Azidopentyl 2,3-*O*-dibenzoyl-4-*O*-[2-cyanoethyl-(phenylmethyl)-phosphate]-α-l-rhamnopyranosyl-(1→3)-2-*O*-benzoyl-4,6-*O*-benzylidene-β-d-glucopyranoside (**41**)

To a stirred solution of starting material **40** (235 mg, 0.28 mmol, 1 equiv) in CH_2_Cl_2_ (10 mL), benzyl 2-cyanoethyl *N*,*N*-diisopropylphosphoramidite^26^ (347 mg, 1.12 mmol, 4 equiv) and 1*H*-tetrazole (0.45 M in acetonitrile, 5.00 mL, 2.28 mmol, 8 equiv) were added at room temperature. Stirring was continued until TLC analysis indicated all starting materials had disappeared (40 min). Then, the reaction was cooled to −20 °C, and *m*-CPBA (243 mg, 1.40 mmol, 5 equiv) was added. Stirring was continued until TLC analysis indicated all starting materials had disappeared (20 min). Upon completion, the reaction was quenched by satutated aq. NaHCO_3_ (10 mL) and extracted by CH_2_Cl_2_ (15 mL × 2). The separated organic layer was dried over MgSO_4_ and concentrated. The obtained residue was purified by silica gel column chromatography using EtOAc/*n*-hexane (1:1) as eluents to give a mixture of two phosphate diastereomers (ratio = 1:1) **41** as a white powder (278 mg, 93%). No attempt was made to separate this diastereomer mixture, which was directly used in the next step. However, for data analysis, a portion of the mixture was subjected to column chromatography and separated two phosphate diastereomers.

Polar phosphate diastereomer: *R*_f_ = 0.36 (silica gel, EtOAc/*n*-hexane = 1:1); ^1^H NMR (600 MH_Z_, CDCl_3_): δ 8.00–7.99 (m, 2H, Ar–H), 7.87–7.85 (m, 2H, Ar–H), 7.69–7.67 (m, 2H, Ar–H), 7.52–7.49 (m, 3H, Ar–H), 7.44–7.40 (m, 2H, Ar–H), 7.37–7.30 (m, 7H, Ar–H), 7.27–7.24 (m, 2H, Ar–H), 7.22–7.20 (m, 1H, Ar–H), 7.17–7.14 (m, 2H), 6.92–6.90 (m, 2H, Ar–H), 5.68 (dd, *J* = 9.6, 3.6 Hz, 1H), 5.62 (s, 1H, Ph–CH), 5.38 (dd, *J* = 9.0, 7.8 Hz, 1H), 5.33 (dd, *J* = 3.6, 1.8 Hz, 1H), 5.03 (d, *J* = 1.2 Hz, 1H, C1–H_α_), 4.71 (dd, *J* = 12.0, 7.8 Hz, 1H), 4.63 (d, *J* = 8.4 Hz, 1H, C1–H_β_), 4.54 (q, *J* = 9.6 Hz, 1H), 4.45 (dd, *J* = 5.4, 5.4 Hz, 1H), 4.40 (dd, *J* = 10.8, 4.8 Hz, 1H), 4.29 (dq, *J* = 9.6, 6.0 Hz, 1H), 4.18 (dd, *J* = 9.6, 9.0 Hz, 1H), 3.90–3.82 (m, 3H), 3.81–3.76 (m, 1H), 3.75–3.69 (m, 1H), 3.58 (ddd, *J* = 9.6, 9.6, 4.8 Hz, 1H), 3.45 (ddd, *J* = 9.6, 7.2, 5.4 Hz, 1H), 3.00–2.95 (m, 1H, −C*H*_a_H_blinker_), 2.92–2.88 (m, 1H, −CH_a_*H*_blinker_), 2.31 (t, *J* = 6.6 Hz, 2H, –CH_2_), 1.55–1.45 (m, 2H, –CH_2linker_), 1.43–1.36 (m, 2H, –CH_2linker_), 1.29–1.18 (m, 2H, –CH_2linker_), 1.03 (d, *J* = 6.6 Hz, 3H, –CH_3_); ^13^C{^1^H} NMR (150 MHz, CDCl_3_): δ 165.2, 164.8, 164.5, 137.0, 134.9, 134.9, 133.2, 133.1, 129.8, 129.7, 129.4, 129.3, 129.3, 129.1, 128.6, 128.5, 128.3, 128.3, 128.2, 127.9, 126.3, 116.0, 101.9, 101.7, 97.5, 78.9, 77.6, 76.2, 74.4, 70.7, 69.9, 69.7, 69.7, 69.6, 69.5, 68.8, 66.8, 66.8, 66.7, 61.6, 61.6, 51.1, 28.9, 28.4, 23.1, 19.1, 19.1, 17.1; ^31^P NMR (162 MH_Z_, CDCl_3_): δ −2.01; HRMS (ESI-TOF) *m*/*z*: [M + Na]^+^ calcd for C_55_H_57_N_4_O_16_PNa, 1083.3399; found, 1083.3408. Nonpolar phosphate diastereomer: *R*_f_ = 0.47 (silica gel, EtOAc/hexane = 1:1); ^1^H NMR (600 MH_Z_, CDCl_3_): δ 8.00–7.99 (m, 2H, Ar–H), 7.93–7.91 (m, 2H, Ar–H), 7.70–7.68 (m, 2H, Ar–H), 7.52–7.45 (m, 4H, Ar–H), 7.43–7.40 (m, 1H, Ar–H), 7.36–7.30 (m, 9H, Ar–H), 7.28–7.27 (m, 2H, Ar–H), 7.29–7.21 (m, 2H, Ar–H), 7.17–7.15 (m, 1H, Ar–H), 5.70 (dd, *J* = 9.6, 3.6 Hz, 1H), 5.61 (s, 1H, Ph–CH), 5.38 (dd, *J* = 9.0, 8.4 Hz, 1H), 5.33 (dd, *J* = 3.6, 1.2 Hz, 1H), 5.03 (d, *J* = 1.2 Hz 1H, C1–H_α_), 4.90 (dd, *J* = 11.4, 7.2 Hz, 1H), 4.83 (dd, *J* = 11.4, 7.8 Hz, 1H), 4.62 (d, *J* = 7.8 Hz 1H, C1–H_β_), 4.56 (q, *J* = 9.6 Hz, 1H), 4.40 (dd, *J* = 10.8, 4.8 Hz, 1H), 4.29 (dq, *J* = 9.6, 6.0 Hz, 1H), 4.17 (dd, *J* = 9.6, 8.4 Hz, 1H), 3.90–3.82 (m, 3H), 3.68–3.63 (m, 1H), 3.60–3.55 (m, 1H), 3.45 (ddd, *J* = 9.6, 7.2, 5.4 Hz, 1H), 2.99–2.95 (m, 1H, −C*H*_a_H_blinker_), 2.92–2.88 (m, 1H, −CH_a_*H*_blinker_), 1.99–1.90 (m, 2H, –CH_2_), 1.56–1.45 (m, 2H, –CH_2linker_), 1.42–1.35 (m, 2H, –CH_2linker_), 1.28–1.19 (m, 2H, –CH_2linker_), 0.98 (d, *J* = 6.6 Hz, 3H, –CH_3_); ^13^C{^1^H} NMR (150 MHz, CDCl_3_): δ 165.1, 164.7, 164.5, 136.9, 135.2, 135.1, 133.4, 133.2, 133.1, 129.8, 129.6, 129.4, 129.3, 129.1, 129.0, 128.8, 128.6, 128.5, 128.3, 128.2, 128.1, 127.9, 126.2, 115.8, 101.8, 101.7, 97.6, 78.9, 77.6, 77.6, 76.3, 74.4, 70.7, 69.8, 69.7, 69.6, 68.7, 66.8, 66.7, 61.5, 61.5, 51.0, 28.9, 28.3, 23.0, 18.5, 18.5, 17.0; ^31^P NMR (162 MH_Z_, CDCl_3_): δ −1.89; HRMS (ESI-TOF) *m*/*z*: [M + Na]^+^ calcd for C_55_H_57_N_4_O_16_PNa, 1083.3399; found, 1083.3408.

### 5-Azidopentyl 2,3-*O*-dibenzoyl-4-*O*-oxopentanoate-α-l-rhamnopyranosyl-(1→3)-2-*O*-benzoyl-4,6-*O*-benzylidene-β-d-glucopyranosyl-(1→4)-6-*O*-acetyl-2-*O*-benzyl-3-*O*-(2-naphthylmethyl)-1-α-d-glucopyranoside (**43**)

A mixture of acceptor **42** (540 mg, 0.96 mmol, 1 equiv), donor **16** (1.20 g, 1.20 mmol, 1.25 equiv), and activated pulverized 4 Å molecular sieves (1.5 g) in anhydrous CH_2_Cl_2_ (15 mL) was stirred under an argon atmosphere for 1 h. The reaction was cooled to −40 °C, and TMSOTf (0.22 mL, 1.20 mmol, 1.25 equiv. with respect to acceptor) was added. Stirring was continued until TLC analysis indicated all starting materials had disappeared (30 min). Then, the reaction was quenched by Et_3_N (0.3 mL) and filtered through a pad of Celite. The solvent was removed, and the obtained residue was purified by silica gel column chromatography using EtOAc/toluene (1:4) as eluents to give compound **43** as a white powder (1.30 g, 99%). *R*_f_ = 0.40 (silica gel, EtOAc/toluene = 3:1); ^1^H NMR (600 MH_Z_, CDCl_3_): δ 8.04–8.03 (m, 2H, Ar–H), 7.89–7.87 (m, 3H, Ar–H), 7.84 (s, 1H, Ar–H), 7.80–7.79 (m, 2H, Ar–H), 7.67–7.66 (m, 2H, Ar–H), 7.55–7.48 (m, 4H, Ar–H), 7.45–7.43 (m, 1H, Ar–H), 7.39–7.25 (m, 17H, Ar–H), 5.57 (dd, *J* = 10.2 3.6 Hz, 1H), 5.43 (dd, *J* = 9.0, 7.8 Hz, 1H), 5.39 (s, 1H, Ph–CH) 5.31 (dd, *J* = 3.6, 1.2 Hz, 1H), 5.15 (dd, *J* = 10.2, 10.2 Hz, 1H), 5.11 (d, *J* = 11.4 Hz, 1H), 5.08 (d, *J* = 11.4 Hz, 1H), 4.96 (s, 1H, C1–H_α_), 4.78 (d, *J* = 7.8 Hz, 1H, C1–H_β_), 4.74 (d, *J* = 12.0 Hz, 1H), 4.66 (d, *J* = 3.6 Hz, 1H, C1–H_α_), 4.62 (d, *J* = 12.0 Hz, 1H), 4.23–4.20 (m, 1H), 4.17–4.08 (m, 4H), 3.97 (dd, *J* = 9.0, 9.0 Hz, 1H), 3.71(dd, *J* = 10.2, 8.4 Hz, 1H), 3.67–3.63 (m, 2H), 3.55–3.49 (m, 2H), 3.40 (dd, *J* = 10.2, 10.2 Hz, 1H), 3.66–3.30 (m, 2H), 3.19 (t, *J* = 7.2 Hz, 1H, –CH_2linke_), 2.58–2.52 (m, 1H, −CH_a_*H*_b_), 2.47–2.38 (m, 2H, –CH_2_), 2.31–2.26 (m, 1H, −CH_a_*H*_b_), 2.00 (s, 3H, –CH_3_), 1.84 (s, 3H, –CH_3_), 1.61–1.54 (m, 4H, 2-CH_2linker_), 1.42–1.35 (m, 2H, –CH_2linker_), 0.78 (d, *J* = 6.0 Hz, 3H, –CH_3_); ^13^C{^1^H} NMR (150 MHz, CDCl_3_): δ 205.7, 171.8, 170.4, 165.2, 164.6, 164.3, 138.2, 136.8, 136.7, 133.3, 133.2, 133.0, 132.9, 129.9, 129.7, 129.6, 129.2, 129.2, 129.1, 128.8, 128.4, 128.3, 128.3, 128.2, 128.2, 128.1, 127.9, 127.8, 127.8, 127.8, 127.7, 126.2, 126.1, 125.7, 125.6, 101.6, 101.5, 97.9, 96.7, 79.9, 79.4, 78.6, 78.0, 76.3, 75.4, 74.8, 73.1, 71.3, 70.3, 69.4, 68.4, 68.2, 68.0, 66.6, 66.5, 62.1, 51.2, 37.7, 29.5, 28.7, 28.5, 27.8, 23.3, 20.6, 16.5; HRMS (ESI-TOF) *m*/*z*: [M + Na]^+^ calcd for C_76_H_79_N_3_O_21_Na, 1392.5098; found, 1392.5117.

### 5-Azidopentyl 2,3-*O*-dibenzoyl-α-l-rhamnopyranosyl-(1→3)-2-*O*-benzoyl-4,6-*O*-benzylidene-β-d-glucopyranosyl-(1→4)-6-*O*-acetyl-2-*O*-benzyl-3-*O*-(2-naphthylmethyl)-1-α-d-glucopyranoside (**44**)

To a stirred solution of starting material **43** (240 mg, 0.18 mmol, 1 equiv) in CH_2_Cl_2_ (5 mL), hydrazine hydrate (34 μL, 0.70 mmol, 4 equiv) dissolved in AcOH (0.4 mL) and pyridine (0.6 mL) were added at room temperature. Stirring was continued until TLC analysis indicated all starting materials had disappeared (1 h). Upon completion, it was quenched by acetone (0.2 mL). The solvent was removed, and the obtained residue was purified by silica gel column chromatography using EtOAc/*n*-hexane (1:2) as eluents to give compound **44** as a white powder (212 mg, 95%). *R*_f_ = 0.69 (silica gel, EtOAc/*n*-hexane = 1:1); ^1^H NMR (600 MH_Z_, CDCl_3_): δ 8.04–8.02 (m, 2H, Ar–H), 7.87–7.86 (m, 3H, Ar–H), 7.83–7.81 (m, 3H, Ar–H), 7.71–7.69 (m, 2H, Ar–H), 7.54–7.45 (m, 5H, Ar–H), 7.40–7.38 (m, 2H, Ar–H), 7.36–7.33 (m, 3H, Ar–H), 7.33–7.24 (m, 12H, Ar–H), 5.41 (dd, *J* = 7.2, 3.6 Hz, 1H), 5.40–5.38 (m, 2H, Ph–CH), 5.28 (dd, *J* = 3.6, 1.2 Hz, 1H), 5.11–5.06 (m, 2H), 4.93 (d, *J* = 1.8 Hz, 1H, C1–H_α_), 4.77 (d, *J* = 7.8 Hz, 1H, C1–H_β_), 4.73 (d, *J* = 12.0 Hz, 1H), 4.65 (d, *J* = 3.6 Hz, 1H, C1–H_α_), 4.61 (d, *J* = 12.0 Hz, 1H), 4.16–4.07 (m, 4H), 4.05–4.01 (m, 1H), 3.96 (dd, *J* = 9.0, 9.0 Hz, 1H), 3.72 (dd, *J* = 9.6, 9.0 Hz, 1H), 3.67–3.62 (m, 3H), 3.54–3.48 (m, 2H), 3.40 (dd, *J* = 10.2, 9.6 Hz, 1H), 3.37–3.29 (m, 2H), 3.18 (t, *J* = 7.2 Hz, 2H, –CH_2linker_), 2.24 (d, *J* = 5.4 Hz, 1H, OH), 1.84 (s, 3H, –CH_3_), 1.63–1.54 (m, 4H, 2-CH_2linker_) 1.41–1.34 (m, 2H, –CH_2linker_), 0.90 (d, *J* = 6.0 Hz, 3H, –CH_3_); ^13^C{^1^H} NMR (150 MHz, CDCl_3_): δ 170.4, 167.0, 164.7, 164.4, 138.2, 136.8, 136.7, 133.3, 133.3, 133.2, 133.1, 132.9, 130.0, 129.8, 129.6, 129.3, 129.2, 129.2, 128.8, 128.4, 128.4, 128.3, 128.2, 128.2, 127.9, 127.8, 127.8, 127.8, 126.2, 126.1, 125.7, 125.6, 101.7, 101.5, 98.2, 96.7, 79.9, 79.4, 78.7, 78.0, 76.7, 75.5, 74.8, 73.1, 72.8, 71.9, 70.5, 69.0, 68.4, 68.2, 68.0, 66.7, 62.1, 51.2, 28.8, 28.5, 23.3, 20.6, 16.8; HRMS (ESI-TOF) *m*/*z*: [M + Na]^+^ calcd for C_71_H_73_N_3_O_19_Na, 1294.4730; found, 1294.4753.

### 5-Azidopentyl 2,3-*O*-dibenzoyl-4-*O*-[2-cyanoethyl-(phenylmethyl)-phosphate]-α-l-rhamnopyranosyl-(1→3)-2-*O*-benzoyl-4,6-*O*-benzylidene-β-d-glucopyranosyl-(1→4)-6-*O*-acetyl-2-*O*-benzyl-3-*O*-(2-naphthylmethyl)-1-α-d-glucopyranoside (**45**)

To a stirred solution of starting material **44** (270 mg, 0.21 mmol, 1 equiv) in CH_2_Cl_2_ (10 mL), benzyl 2-cyanoethyl *N*,*N*-diisopropylphosphoramidite^3^ (262 mg, 0.85 mmol, 4 equiv) and 1*H*-tetrazole (0.45 M in acetonitrile, 3.77 mL, 1.70 mmol, 8 equiv) were added at room temperature. Stirring was continued until TLC analysis indicated all starting materials had disappeared (40 min). Upon completion, the reaction was cooled to −20 °C, and *m*-CPBA (238 mg, 1.06 mmol, 5 equiv) was added. Stirring continued until TLC analysis indicated all starting materials (20 min) had disappeared. Then, the reaction was quenched by saturated aq. NaHCO_3_ (10 mL) and extracted by CH_2_Cl_2_ (15 mL × 2). The separated organic layer was dried over MgSO_4_ and concentrated. The obtained residue was purified by silica gel column chromatography using EtOAc/*n*-hexane (1:1) as eluents to give a mixture of two phosphate diastereomers (ratio = 1:1) **45** as a white powder (302 mg, 95%). No attempt was made to separate the diastereomers, which were directly used in the next step. However, for data analysis, a portion of the mixture was subjected to column chromatography and separated two phosphate diastereomers. Polar phosphate diastereomer: *R*_f_ = 0.39 (silica gel, EtOAc/*n*-hexane = 1:1); ^1^H NMR (600 MH_Z_, CDCl_3_): δ 8.02–8.00 (m, 2H, Ar–H), 7.88–7.82 (m, 6H, Ar–H), 7.65–7.64 (m, 2H, Ar–H), 7.52–7.47 (m, 4H, Ar–H), 7.44–7.41 (m, 1H, Ar–H), 7.37–7.23 (m, 17H, Ar–H), 7.22–7.19 (m, 1H, Ar–H), 7.16–7.13 (m, 2H, Ar–H), 6.91–6.89 (m, 2H, Ar–H), 5.64 (dd, *J* = 9.6, 3.6 Hz, 1H), 5.41 (dd, *J* = 9.0, 8.4 Hz, 1H), 5.37 (s, 1H, Ph–CH), 5.27 (dd, *J* = 3.6, 1.8 Hz, 1H), 5.11–5.05 (m, 2H), 4.92 (d, *J* = 1.2 Hz, 1H, C1–H_α_), 4.77 (d, *J* = 7.8 Hz, 1H, C1–H_β_), 4.74–4.69 (m, 2H), 4.65 (d, *J* = 3.6 Hz, 1H, C1–H_α_), 4.61 (d, *J* = 12.0 Hz, 1H), 4.48 (ddd, *J* = 9.6, 9.6, 9.6 Hz, 1H), 4.43 (dd, *J* = 11.4, 11.4 Hz, 1H), 4.20 (dq, *J* = 9.6, 6.0 Hz, 1H), 4.14 (dd, *J* = 12.0, 3.6 Hz, 1H), 4.11–4.05 (m, 3H), 3.96 (dd, *J* = 9.0, 9.0 Hz, 1H), 3.79–3.74 (m, 1H), 3.73–3.68 (m, 2H), 3.66–3.62 (m, 2H), 3.54–3.48 (m, 2H), 3.38 (dd, *J* = 10.2, 10.2 Hz, 1H), 3.35–3.29 (m, 2H), 3.18 (t, *J* = 7.2 Hz, 2H, –CH_2linker_), 2.30 (t, *J* = 6.6 Hz, 2H, –CH_2_), 1.83 (s, 3H, –CH_3_), 1.58–1.53 (m, 4H, 2-CH_2linker_), 1.40–1.35 (m, 2H, –CH_2linker_), 0.93 (d, *J* = 6.6 Hz, 3H, –CH_3_); ^13^C{^1^H} NMR (150 MHz, CDCl_3_): δ 170.5, 165.6, 165.4, 165.3, 164.4, 164.3, 137.9, 137.8, 137.1, 134.9, 134.9, 133.2, 133.1, 130.0, 129.9, 129.9, 129.8, 129.8, 129.7, 129.6, 129.4, 129.0, 128.9, 128.9, 128.8, 128.6, 128.5, 128.4, 128.3, 128.3, 128.1, 127.9, 127.9, 126.3, 116.0, 101.1, 100.7, 97.7, 97.4, 96.6, 80.9, 79.9, 77.8, 77.7, 76.5, 76.2, 74.9, 74.4, 73.2, 73.0, 72.6, 71.1, 70.5, 69.7, 69.7, 69.6, 68.6, 68.1, 67.6, 67.3, 66.7, 66.7, 62.0, 61.6, 61.6, 51.2, 28.7, 28.5, 23.3, 20.9, 19.2, 19.1, 18.1, 17.0; ^31^P NMR (162 MH_Z_, CDCl_3_): δ −2.01; HRMS (ESI-TOF) *m*/*z*: [M + Na]^+^ calcd for C_81_H_83_N_4_O_22_PNa, 1517.5129; found, 1517.5134. Nonpolor phosphate diastereomer: *R*_f_ = 0.56 (silica gel, EtOAc/hexane = 1:1); ^1^H NMR (600 MH_Z_, CDCl_3_): δ 8.02–8.01 (m, 2H, Ar–H), 7.91–7.90 (m, 2H, Ar–H), 7.88–7.86 (m, 3H, Ar–H), 7.83 (s, 1H, Ar–H), 7.66–7.65 (m, 2H, Ar–H), 7.53–7.46 (m, 5H, Ar–H), 7.36–7.31 (m, 10H, Ar–H), 7.30–7.24 (m, 9H, Ar–H), 7.19–7.16 (m, 2H, Ar–H), 7.14–7.11 (m, 1H, Ar–H), 5.65 (dd, *J* = 9.6, 3.6 Hz, 1H), 5.41 (dd, *J* = 9.0, 7.8 Hz, 1H), 5.37 (s, 1H, Ph–CH), 5.27 (dd, *J* = 3.6, 1.2 Hz, 1H), 5.11–5.06 (m, 2H), 4.91 (s, 1H, C1–H_α_), 4.89 (dd, *J* = 11.4, 7.8 Hz, 1H), 4.81 (dd, *J* = 11.4, 7.8 Hz, 1H), 4.77 (d, *J* = 7.8 Hz, 1H, C1–H_β_), 4.73 (d, *J* = 12.0 Hz, 1H), 4.66 (d, *J* = 3.6 Hz, 1H, C1–H_α_), 4.61 (d, *J* = 12.0 Hz, 1H), 4.51 (ddd, *J* = 9.6, 9.6, 9.6 Hz, 1H), 4.20 (dq, *J* = 9.6, 6.0 Hz, 1H), 4.15 (dd, *J* = 12.0, 3.6 Hz, 1H), 4.09–4.05 (m, 3H), 3.96 (dd, *J* = 9.6, 9.0 Hz, 1H), 3.71 (dd, *J* = 9.6, 8.4 Hz, 1H), 3.67–3.62 (m, 3H), 3.58–3.48 (m, 3H), 3.39 (dd, *J* = 10.2, 10.2 Hz, 1H), 3.35–3.29 (m, 2H), 3.18 (t, *J* = 7.2 Hz, 2H, –CH_2linker_), 1.98–1.88 (m, 2H, –CH_2_), 1.83 (s, 3H, –CH_3_), 1.61–1.53 (m, 4H, 2-CH_2linker_), 1.41–1.34 (m, 2H, –CH_2linker_), 0.88 (d, *J* = 6.0 Hz, 3H, –CH_3_); ^13^C{^1^H} NMR (150 MHz, CDCl_3_): δ 170.4, 165.0, 164.6, 164.4, 138.2, 136.8, 135.2, 135.1, 133.4, 133.3, 133.2, 133.2, 132.9, 129.9, 129.8, 129.6, 129.3, 129.1, 128.9, 128.8, 128.6, 128.5, 128.4, 128.4, 128.3, 128.1, 127.9, 127.8, 127.8, 127.8, 126.2, 126.1, 125.7, 125.7, 125.6, 115.8, 101.7, 101.5, 97.7, 96.7, 79.9, 79.4, 78.6, 78.0, 77.5, 77.5, 76.5, 75.5, 74.7, 73.1, 70.4, 69.6, 69.6, 68.4, 68.2, 68.0, 66.8, 66.8, 66.6, 62.1, 61.5, 61.4, 51.2, 28.8, 28.6, 23.3, 20.6, 18.5, 18.5, 16.8; ^31^P NMR (162 MH_Z_, CDCl_3_): δ −1.87; HRMS (ESI-TOF) *m*/*z*: [M + Na]^+^ calcd for C_81_H_83_N_4_O_22_PNa, 1517.5129; found, 1517.5134.

### 5-Azidopentyl 2,3-*O*-dibenzoyl-α-l-rhamnopyranosyl-(1→3)-2-*O*-benzoyl-4,6-*O*-benzylidene-β-d-glucopyranosyl-(1→4)-6-*O*-acetyl-2-*O*-benzyl-1-α-d-glucopyranoside (**46**)

To a stirred solution of compound **43** (1.30 g, 0.95 mmol, 1 equiv) in CH_2_Cl_2_/phosphate buffer (pH 7, 45 mL, 9:1 = *v*/*v*), 2,3-dichloro-5,6-dicyanobenzoquinone (337 mg, 1.66 mmol, 1.75 equiv) was added at 0 °C. The reaction mixture was vigorously stirred until TLC analysis indicated all starting material had disappeared (3 h). Upon completion, the reaction mixture was diluted with CH_2_Cl_2_ (20 mL) and washed with saturated aq. NaHCO_3_ (20 mL) and brine (10 mL). The organic phase was washed with water until the solution became colorless; then, the separated organic layer was dried over MgSO_4_, filtered, and concentrated. The obtained residue was purified by silica gel column chromatography using EtOAc/*n*-hexane (2:3) as eluents to give compound **46** as a white powder (1.11 g, 95%). *R*_f_ = 0.35 (silica gel, EtOAc/*n*-hexane = 1:1); ^1^H NMR (600 MH_Z_, CDCl_3_): δ 8.00–7.98 (m, 2H, Ar–H), 7.79–7.78 (m, 2H, Ar–H), 7.65–7.64 (m, 2H, Ar–H), 7.51–7.47 (m, 3H, Ar–H), 7.45–7.42 (m, 1H, Ar–H), 7.38–7.28 (m, 15H, Ar–H), 5.63 (s, 1H, Ph–CH), 5.57 (dd, *J* = 10.2, 3.6 Hz, 1H), 5.45 (dd, *J* = 8.4, 8.4 Hz, 1H), 5.30 (dd, *J* = 3.6, 1.2 Hz, 1H), 5.16 (dd, *J* = 10.2, 10.2 Hz, 1H), 4.98 (d, *J* = 1.2 Hz, 1H, C1–H_α_), 4.83 (d, *J* = 12.6 Hz, 1H), 4.72 (d, *J* = 7.8 Hz, 1H, C1–H_β_), 4.62 (d, *J* = 7.8 Hz, 1H), 4.60 (s, 1H, C1–H_α_), 4.45 (dd, *J* = 10.2, 4.8 Hz, 1H), 4.27–4.23 (m, 1H), 4.20 (dd, *J* = 9.6, 9.0 Hz, 1H), 4.03–3.94 (m, 3H), 3.90–3.85 (m, 2H), 3.68–3.64 (m, 3H), 3.53–3.49 (m, 1H), 3.41 (dd, *J* = 9.6, 8.4 Hz, 1H), 3.35 (dd, *J* = 9.6, 3.6 Hz, 1H), 3.25–3.19 (m, 3H, –CH_2linker_), 2.58–2.52 (m, 1H, −CH_a_*H*_b_), 2.47–2.38 (m, 2H, –CH_2_), 2.30–2.25 (m, 1H, −CH_a_*H*_b_), 1.99 (s, 3H, –CH_3_), 1.84 (s, 3H, –CH_3_), 1.59–1.54 (m, 4H, 2-CH_2linker_), 1.40–1.35 (m, 2H, –CH_2linker_), 0.83 (d, *J* = 6.0 Hz, 3H, –CH_3_); ^13^C{^1^H} NMR (150 MHz, CDCl_3_): δ 205.7, 171.8, 170.1, 165.2, 164.6, 164.4, 138.4, 136.6, 133.3, 133.1, 129.9, 129.7, 129.6, 129.3, 129.2, 129.0, 128.5, 128.4, 128.3, 128.2, 128.2, 128.2, 127.8, 127.7, 126.2, 101.9, 101.9, 97.9, 96.8, 81.6, 78.5, 78.4, 76.0, 74.1, 73.0, 71.2, 70.2, 69.3, 68.3, 68.1, 67.2, 67.0, 66.6, 62.0, 51.2, 37.6, 29.5, 28.7, 28.5, 27.8, 23.3, 20.5, 16.6; HRMS (ESI-TOF) *m*/*z*: [M + Na]^+^ calcd for C_65_H_71_N_3_O_21_Na, 1252.4472; found, 1252.4466.

### 5-Azidopentyl 2,3-*O*-dibenzoyl-4-*O*-oxopentanoate-α-l-rhamnopyranosyl-(1→3)-2-*O*-benzoyl-4,6-*O*-benzylidene-β-d-glucopyranosyl-(1→4)-[2,3-di-*O*-benzoyl-4-*O*-benzyl-α-l-rhamnopyranosyl-(1→3)]-6-*O*-acetyl-2-*O*-benzyl-1-α-d-glucopyranoside (**47**)

A mixture of acceptor **46** (1.05 g, 0.85 mmol, 1 equiv), donor **13** (777 mg, 1.28 mmol, 1.5 equiv), and activated pulverized 4 Å molecular sieves (1.5 g) in anhydrous CH_2_Cl_2_ (15 mL) was stirred under an argon atmosphere for 1 h. Then, the reaction was cooled to −30 °C, and TMSOTf (23 μL, 0.13 mmol, 0.15 equiv. with respect to acceptor) was added. Stirring was continued until TLC analysis indicated all starting materials had disappeared (1 h). Upon completion, the reaction was quenched by Et_3_N (0.2 mL) and filtered through a pad of Celite. The solvent was removed, and the obtained residue was purified by silica gel column chromatography using EtOAc/*n*-hexane (2:3) as eluents to give compound **47** as a white powder (1.33 g, 93%). *R*_f_ = 0.44 (silica gel, EtOAc/*n*-hexane = 1:1); ^1^H NMR (600 MH_Z_, CDCl_3_): δ 8.06–8.01 (m, 6H, Ar–H), 7.83–7.82 (m, 2H, Ar–H), 7.68–7.66 (m, 2H, Ar–H), 7.61–7.58 (m, 1H, Ar–H), 7.56–7.53 (m, 1H, Ar–H), 7.50–7.44 (m, 4H, Ar–H), 7.41–7.36 (m, 5H, Ar–H), 7.34–7.25 (m, 15H, Ar–H), 7.19–7.17 (m, 2H, Ar–H), 7.14–7.11 (m, 2H, Ar–H), 5.73–5.71 (m, 2H), 5.67 (dd, *J* = 10.2, 3.6 Hz, 1H), 5.52 (s, 1H, C1–H_α_), 5.36 (dd, *J* = 9.0, 8.4 Hz, 1H), 5.34 (dd, *J* = 3.6, 1.8 Hz, 1H), 5.17 (dd, *J* = 10.2, 10.2 Hz, 1H), 4.92 (d, *J* = 0.6 Hz, 1H, C1–H_α_), 4.89–4.84 (m, 2H), 4.77 (d, *J* = 9.6 Hz, 1H), 4.71 (d, *J* = 12.0 Hz, 1H), 4.55 (d, *J* = 7.8 Hz, 1H, C1–H_β_), 4.55 (d, *J* = 4.2 Hz, 1H, C1–H_α_), 4.51 (d, *J* = 12.0 Hz, 1H), 4.41 (dd, *J* = 10.2, 4.2 Hz, 1H), 4.27 (s, 1H, Ph–CH), 4.22–4.18 (m, 2H), 4.11–4.07 (m, 2H), 4.02 (dd, *J* = 9.6, 9.6 Hz, 1H), 3.86–3.82 (m, 2H), 3.75 (dd, *J* = 9.6, 9.6 Hz, 1H), 3.55–3.52 (m, 2H), 3.47–3.42 (m, 2H), 3.37–3.33 (m, 1H), 3.19–3.16 (m, 1H), 3.15 (t, *J* = 7.2 Hz, 2H, –CH_2linker_), 2.63–2.58 (m, 1H, −C*H*_a_H_b_), 2.53–2.46 (m, 2H, –CH_2_), 2.39–2.33 (m, 1H, −CH_a_*H*_b_), 2.08 (s, 3H, –CH_3_), 2.01 (s, 3H, –CH_3_), 1.74 (d, *J* = 6.6 Hz, 3H, –CH_3_), 1.53–1.48 (m, 4H, 2-CH_2linker_), 1.34–1.29 (m, 2H, –CH_2linker_), 0.76 (d, *J* = 6.0 Hz, 3H, –CH_3_); ^13^C{^1^H} NMR (150 MHz, CDCl_3_): δ 205.7, 171.8, 170.4, 165.5, 165.4, 165.3, 164.3, 137.9, 137.8, 137.1, 133.1, 133.1, 133.0, 133.0, 129.9, 129.9, 129.8, 129.7, 129.7, 129.6, 129.3, 129.1, 128.9, 128.8, 128.8, 128.6, 128.4, 128.3, 128.2, 128.2, 128.0, 127.8, 127.8, 126.2, 101.1, 100.5, 97.9, 97.4, 96.5, 80.9, 79.8, 77.8, 76.5, 76.2, 74.8, 74.4, 73.2, 73.0, 72.6, 71.6, 71.1, 70.3, 69.4, 68.5, 68.1, 67.5, 67.3, 66.3, 61.9, 51.1, 37.7, 29.5, 28.7, 28.5, 27.8, 23.2, 20.9, 18.1, 16.6; HRMS (ESI-TOF) *m*/*z*: [M + Na]^+^ calcd for C_92_H_95_N_3_O_27_Na, 1696.6045; found, 1696.6034.

### 5-Azidopentyl 2,3-*O*-dibenzoyl-α-l-rhamnopyranosyl-(1→3)-2-*O*-benzoyl-4,6-*O*-benzylidene-β-d-glucopyranosyl-(1→4)-[2,3-di-*O*-benzoyl-4-*O*-benzyl-α-l-rhamnopyranosyl-(1→3)]-6-*O*-acetyl-2-*O*-benzyl-1-α-d-glucopyranoside (**48**)

To a stirred solution of starting material **47** (1.29 g, 0.77 mmol, 1 equiv) in CH_2_Cl_2_ (20 mL), hydrazine hydrate (0.15 mL, 3.08 mmol, 4 equiv) dissolved in AcOH (1.6 mL) and pyridine (2.4 mL) were added at room temperature. Stirring was continued until TLC analysis indicated all starting materials had disappeared (1 h). Upon completion, the reaction was quenched by acetone (0.2 mL). The solvent was removed, and the obtained residue was purified by silica gel column chromatography using EtOAc/*n*-hexane (1:2) as eluents to give compound **48** as a white powder (1.18 g, 97%). *R*_f_ = 0.56 (silica gel, EtOAc/*n*-hexane = 1:1); ^1^H NMR (600 MH_Z_, CDCl_3_): δ 8.06 (d, *J* = 7.2 Hz, 2H, Ar–H), 8.04–8.02 (m, 2H, Ar–H), 7.87–7.85 (m, 2H, Ar–H), 7.71 (d, *J* = 7.2 Hz, 2H, Ar–H), 7.60 (t, *J* = 7.2 Hz, 1H, Ar–H), 7.56–7.46 (m, 5H, Ar–H), 7.42–7.39 (m, 2H, Ar–H), 7.37–7.26 (m, 18H, Ar–H), 7.19–7.12 (m, 4H, Ar–H), 5.73–5.71 (m, 2H), 5.52 (s, 1H, C1–H_α_), 5.47 (dd, *J* = 10.2, 3.6 Hz, 1H), 5.36–5.34 (m, 2H), 4.91 (s, 1H, C1–H_α_), 4.89–4.85 (m, 1H), 4.82 (d, *J* = 10.2 Hz, 1H), 4.76 (d, *J* = 10.2 Hz, 1H), 4.72 (d, *J* = 12.0 Hz, 1H), 4.56–4.55 (m, 2H, C1–H_α,_, C1–H_β_), 4.51 (d, *J* = 12.0 Hz, 1H), 4.41 (dd, *J* = 10.8, 4.2 Hz, 1H), 4.28 (s, 1H, Ph–CH), 4.21 (dd, *J* = 12.0, 3.6 Hz, 1H), 4.11–4.07 (m, 2H), 4.06–4.01 (m, 2H), 3.86–3.83 (m, 2H), 3.76 (dd, *J* = 9.6, 9.6 Hz, 1H), 3.68 (dd, *J* = 9.6, 9.6 Hz, 1H), 3.55–3.52 (m, 2H), 3.47–3.41 (m, 2H), 3.37–3.34 (m, 1H), 3.20–3.16 (m, 1H), 3.13 (t, *J* = 7.2 Hz, 2H, –CH_2linker_), 2.45 (br, 1H, OH), 2.08 (s, 3H, –CH_3_), 1.73 (d, *J* = 6.6 Hz, 3H, –CH_3_), 1.53–1.48 (m, 4H, 2-CH_2linker_), 1.34–1.29 (m, 2H, –CH_2linker_), 0.90 (d, *J* = 6.0 Hz, 3H, –CH_3_); ^13^C{^1^H} NMR (150 MHz, CDCl_3_): δ 170.4, 167.2, 165.5, 165.4, 164.4, 137.8, 137.1, 133.3, 133.1, 133.0, 129.9, 129.8, 129.8, 129.7, 129.7, 129.5, 129.3, 129.2, 128.8, 128.6, 128.4, 128.3, 128.3, 128.2, 128.0, 127.8, 126.2, 101.0, 100.6, 98.1, 97.4, 96.5, 80.8, 79.8, 77.8, 76.7, 76.2, 74.7, 74.4, 73.2, 73.0, 72.6, 72.0, 71.1, 70.5, 68.8, 68.5, 68.0, 67.6, 67.3, 61.9, 51.1, 28.7, 28.5, 23.2, 20.9, 18.0, 16.8; HRMS (ESI-TOF) *m*/*z*: [M + Na]^+^ calcd for C_87_H_89_N_3_O_25_Na, 1598.5677; found, 1598.5662.

### 5-Azidopentyl 2,3-*O*-dibenzoyl-4-*O*-[2-cyanoethyl-(phenylmethyl)-phosphate]-α-l-rhamnopyranosyl-(1→3)-2-*O*-benzoyl-4,6-*O*-benzylidene-β-d-glucopyranosyl-(1→4)-[2,3-di-*O*-benzoyl-4-*O*-benzyl-α-l-rhamnopyranosyl-(1→3)]-6-*O*-acetyl-2-*O*-benzyl-1-α-d-glucopyranoside (**49**)

To a stirred solution of starting material **48** (308 mg, 0.20 mmol, 1 equiv) in CH_2_Cl_2_ (10 mL), benzyl 2-cyanoethyl *N*,*N*-diisopropylphosphoramidite^3^ (241 mg, 0.78 mmol, 4 equiv) and 1*H*-tetrazole (0.45 M in acetonitrile, 3.47 mL, 1.56 mmol, 8 equiv) were added at room temperature. Stirring was continued until TLC analysis indicated all starting materials had disappeared (40 min). Upon completion, the reaction was cooled to −20 °C, and *m*-CPBA (219 mg, 0.98 mmol, 5 equiv) was added. Stirring was continued until TLC analysis indicated the starting materials had disappeared (20 min). Upon completion, the reaction was quenched by saturated aq. NaHCO_3_ (10 mL) and extracted by CH_2_Cl_2_ (15 mL × 2). The separated organic layer was dried over MgSO_4_ and concentrated. The obtained residue was purified by silica gel column chromatography using EtOAc/*n*-hexane (1:1) as eluents to give a mixture of two diastereomers (ratio = 1:1), compound **49**, as a white powder (340 mg, 97%). No attempt was made to separate the diastereomers, which were directly used for the next step. However, for data analysis, a portion of the mixture was subjected to column chromatography and separated two phosphate diastereomers. Polar phosphate diastereomer: *R*_f_ = 0.28 (silica gel, EtOAc/*n*-hexane = 1:1); ^1^H NMR (600 MH_Z_, CDCl_3_): δ 8.03–8.00 (m, 6H, Ar–H), 7.88–7.87 (m, 2H, Ar–H), 7.65–7.64 (m, 2H, Ar–H), 7.61–7.58 (m, 1H, Ar–H), 7.55–7.50 (m, 2H, Ar–H), 7.48–7.42 (m, 3H, Ar–H), 7.40–7.38 (m, 2H, Ar–H), 7.36–7.33 (m, 5H, Ar–H), 7.31–7.21 (m, 13H, Ar–H), 7.21–7.10 (m, 6H, Ar–H), 7.08–7.06 (m, 1H, Ar–H), 6.94–6.92 (m, 2H, Ar–H), 5.73–5.68 (m, 3H), 5.50 (s, 1H, C1–H_α_), 5.54 (dd, *J* = 9.0, 8.4 Hz, 1H), 5.31 (dd, *J* = 3.6, 1.2 Hz, 1H), 4.88 (d, *J* = 1.2 Hz, 1H, C1–H_α_), 4.86–4.83 (m, 2H), 4.76–4.73 (m, 2H), 4.70 (d, *J* = 12.0 Hz,1H), 4.54 (d, *J* = 8.4 Hz, 1H, C1–H_β_), 4.53 (d, *J* = 3.6 Hz, 1H, C1–H_α_), 4.52–4.47 (m, 3H), 4.39 (dd, *J* = 10.8, 4.8 Hz, 1H), 4.24 (s, 1H, Ph–CH), 4.20–4.17 (m, 2H), 4.09–4.06 (m, 2H), 4.00 (dd, *J* = 9.6, 9.6 Hz, 1H), 3.84–3.80 (m, 3H), 3.79–3.71 (m, 2H), 3.54–3.50 (m, 2H), 3.46–3.41 (m, 2H), 3.33 (ddd, *J* = 9.6, 9.6, 4.8 Hz, 1H), 3.16 (ddd, *J* = 9.6, 6.6, 6.6 Hz, 1H), 3.12 (t, *J* = 7.2 Hz, 2H, –CH_2linker_), 2.32 (t, *J* = 6.6 Hz, 2H, –CH_2_), 2.07 (s, 3H, –CH_3_), 1.72 (d, *J* = 6.6 Hz, 3H, –CH_3_), 1.52–1.47 (m, 4H, 2-CH_2linker_), 1.32–1.27 (m, 2H, –CH_2linker_), 0.90 (d, *J* = 6.0 Hz, 3H, –CH_3_); ^13^C{^1^H} NMR (150 MHz, CDCl_3_): δ 170.5, 165.6, 165.4, 165.3, 164.4, 164.3, 137.9, 137.8, 137.1, 134.9, 134.9, 133.2, 133.1, 130.0, 129.9, 129.9, 128.8, 129.8, 129.7, 129.6, 129.4, 129.0, 128.9, 128.9, 128.8, 128.6, 128.5, 128.4, 128.4, 128.3, 128.3, 128.1, 127.9, 127.7, 126.3, 116.0, 101.1, 100.7, 97.7, 97.4, 96.6, 80.9, 79.9, 77.8, 77.7, 76.5, 76.2, 74.9, 74.4, 73.2, 73.0, 72.6, 71.1, 70.5, 69.7, 69.7, 69.6, 68.6, 68.1, 67.6, 67.3, 66.7, 66.7, 62.0, 61.6, 61.6, 51.2, 28.7, 28.5, 23.3, 20.9, 19.2, 19.1, 18.1, 17.0; ^31^P NMR (162 MH_Z_, CDCl_3_): δ −1.83; HRMS (ESI-TOF) *m*/*z*: [M + Na]^+^ calcd for C_97_H_99_N_4_O_28_PNa, 1821.6076; found, 1821.6066. Nonpolar phosphate diastereomer: *R*_f_ = 0.42 (silica gel, EtOAc/hexane = 1:1); ^1^H NMR (600 MH_Z_, CDCl_3_): δ 8.04–8.01 (m, 6H, Ar–H), 7.93 (d, *J* = 7.8 Hz, 2H, Ar–H), 7.66 (d, *J* = 7.8 Hz, 2H, Ar–H), 7.61–7.58 (m, 1H, Ar–H), 7.55–7.46 (m, 5H, Ar–H), 7.41–7.38 (m, 2H, Ar–H), 7.36–7.28 (m, 16H, Ar–H), 7.26–7.25 (m, 2H, Ar–H), 7.21–7.06 (m, 9H, Ar–H), 5.76–5.69 (m, 3H), 5.52 (s, 1H, C1–H_α_), 5.35 (dd, *J* = 9.0, 8.4 Hz, 1H), 5.31 (d, *J* = 3.0 Hz, 1H), 4.96 (dd, *J* = 11.4, 7.8 Hz, 1H), 4.89–4.83 (m, 4H, C1–H_α_), 4.76 (d, *J* = 9.6 Hz, 1H), 4.71 (d, *J* = 12.0 Hz, 1H), 4.55–4.49 (m, 4H, C1–H_β_, C1–H_α_), 4.40 (dd, *J* = 10.2, 4.2 Hz, 1H), 4.24 (s, 1H, Ph–CH), 4.22–4.18 (m, 2H) 4.10–4.07 (m, 2H), 4.00 (dd, *J* = 9.6, 9.0 Hz, 1H), 3.86–3.81 (m, 2H), 3.75–3.67 (m, 2H), 3.63–3.59 (m, 1H), 3.54–3.51 (m, 2H), 3.47–3.41 (m, 2H), 3.34 (dd, *J* = 9.6, 9.6, 4.8 Hz, 1H), 3.19–3.15 (m, 1H), 3.13 (t, *J* = 7.2 Hz, 2H, –CH_2linker_), 2.08 (s, 3H, –CH_3_), 2.03–1.92 (m, 2H, –CH_2_), 1.73 (d, *J* = 6.0 Hz, 3H, –CH_3_), 1.53–1.48 (m, 4H, 2-CH_2linker_), 1.33–1.28 (m, 2H, –CH_2linker_), 0.87 (d, *J* = 6.0 Hz, 3H, –CH_3_); ^13^C{^1^H} NMR (150 MHz, CDCl_3_): δ 170.5, 165.5, 165.4, 165.1, 164.4, 164.3, 137.9, 137.8, 137.0, 135.2, 135.1, 133.4, 133.2, 133.1, 129.9, 129.9, 129.8, 129.8, 129.7, 129.6, 129.4, 129.0, 128.9, 128.8, 128.8, 128.7, 128.7, 128.6, 128.5, 128.4, 128.3, 128.2, 128.1, 127.9, 127.8, 127.8, 126.2, 115.8, 101.0, 100.6, 97.7, 97.4, 96.5, 80.8, 79.9, 77.8, 77.7, 77.7, 76.6, 76.2, 74.8, 74.4, 73.2, 73.0, 72.6, 71.1, 70.5, 69.7, 69.6, 68.5, 68.1, 67.5, 67.3, 66.7, 66.7, 62.0, 61.5, 61.5, 51.1, 28.7, 28.5, 23.2, 20.9, 18.6, 18.5, 18.1, 16.8; ^31^P NMR (162 MH_Z_, CDCl_3_): δ −1.61; HRMS (ESI-TOF) *m*/*z*: [M + Na]^+^ calcd for C_97_H_99_N_4_O_28_PNa, 1821.6076; found, 1821.6060.

### 5-Azidopentyl 2,3-*O*-dibenzoyl-α-l-rhamnopyranosyl-(1→3)-2-*O*-benzoyl-4,6-*O*-benzylidene-β-d-glucopyranosyl-(1→4)-6-*O*-acetyl-2-*O*-benzyl-*3-O*-(2-naphthylmethyl)-α-d-glucopyranosyl-(1→2)-3,4,6*-tri-O*-benzyl-α-d-glucopyranoside (**50**)

To a stirred solution of starting material **25** (500 mg, 0.28 mmol, 1 equiv) in CH_2_Cl_2_ (8 mL), hydrazine hydrate (53 μL, 1.11 mmol, 4 equiv) dissolved in AcOH (0.8 mL) and pyridine (1.2 mL), were added at room temperature. Stirring was continued until TLC analysis indicated all starting materials had disappeared (1 h). The reaction was quenched by acetone (0.3 mL). The solvent was removed, and the obtained residue was purified by silica gel column chromatography using EtOAc/*n*-hexane (1:2) as eluents to give compound **50** as a white powder (440 mg, 93%). *R*_f_ = 0.66 (silica gel, EtOAc/*n*-hexane = 1:1); ^1^H NMR (600 MH_Z_, CDCl_3_): δ 7.99 (d, *J* = 7.2 Hz, 2H, Ar–H), 7.85–7.81 (m, 5H, Ar–H), 7.78 (s, 1H, Ar–H), 7.71 (d, *J* = 7.2 Hz, 2H, Ar–H), 7.52–7.45 (m, 5H, Ar–H), 7.37–7.34 (m, 4H, Ar–H), 7.32–7.23 (m, 26H, Ar–H), 7.09–7.07 (m, 2H, Ar–H), 5.41 (dd, *J* = 10.2, 3.6 Hz, 1H), 5.36–5.33 (m, 2H, Ph–CH), 5.29 (d, *J* = 1.8 Hz, 1H), 5.08 (d, *J* = 11.4 Hz, 1H), 5.04 (d, *J* = 3.6 Hz, 1H, C1–H_α_), 5.01 (d, *J* = 11.4 Hz,1H), 4.98 (d, *J* = 3.6 Hz, 1H, C1–H_α_), 4.92 (s, 1H, C1–H_α_), 4.89 (d, *J* = 10.8 Hz, 1H), 4.83 (d, *J* = 7.8 Hz, 1H, C1–H_β_), 4.73–4.69 (m, 3H), 4.61 (d, *J* = 12.0 Hz, 1H), 4.56 (d, *J* = 12.0 Hz, 1H), 4.46 (d, *J* = 12.0 Hz, 1H), 4.41 (d, *J* = 10.8 Hz, 1H), 4.10–3.93 (m, 8H), 3.77 (dd, *J* = 9.6, 9.6 Hz, 1H), 3.74 (dd, *J* = 10.2, 1.8 Hz, 1H), 3.69–3.59 (m, 6H), 3.56–3.52 (m, 2H), 3.40–3.30 (m, 3H), 3.16 (t, *J* = 7.2 Hz, 2H, –CH_2linker_), 2.23 (d, *J* = 5.4 Hz, 1H, OH), 1.71 (s, 3H, –CH_3_), 1.59–1.47 (m, 4H, 2-CH_2linker_), 1.36–1.31 (m, 2H, –CH_2linker_), 0.88 (d, *J* = 6.0 Hz, 3H, –CH_3_); ^13^C{^1^H} NMR (150 MHz, CDCl_3_): δ 170.3, 167.1, 164.6, 164.4, 138.3, 138.1, 138.0, 136.8, 136.6, 133.3, 133.3, 133.1, 133.1, 132.9, 130.0, 129.9, 129.6, 129.3, 129.3, 129.2, 128.8, 128.5, 128.4, 128.3, 128.3, 128.2, 128.2, 128.2, 128.0, 127.9, 127.9, 127.8, 127.8, 127.7, 127.6, 126.2, 126.1, 125.7, 125.5, 125.4, 101.7, 101.4, 98.2, 95.9, 94.4, 80.6, 79.9, 79.1, 78.7, 77.8, 77.6, 76.4, 75.7, 75.2, 75.0, 74.9, 73.4, 72.8, 72.3, 72.0, 70.5, 70.4, 69.0, 68.8, 68.6, 68.5, 68.0, 66.6, 61.9, 51.2, 29.1, 28.6, 23.4, 20.5, 16.8; HRMS (ESI-TOF) *m*/*z*: [M + Na]^+^ calcd for C_98_H_101_N_3_O_24_Na 1726.6667; found, 1726.6704.

### 5-Azidopentyl 2,3-*O*-dibenzoyl-4-*O*-[2-cyanoethyl-(phenylmethyl)-phosphate]-α-l-rhamnopyranosyl-(1→3)-2-*O*-benzoyl-4,6-*O*-benzylidene-β-d-glucopyranosyl-(1→4)-6-*O*-acetyl-2-*O*-benzyl-*3-O*-(2-naphthylmethyl)-α-d-glucopyranosyl-(1→2)-3,4,6*-tri-O*-benzyl-α-d-glucopyranoside (**51**)

To a stirred solution of starting material **50** (275 mg, 0.16 mmol, 1 equiv) in CH_2_Cl_2_ (10 mL), benzyl 2-cyanoethyl *N*,*N*-diisopropylphosphoramidite^26^ (199 mg, 0.65 mmol, 4 equiv) and 1*H*-tetrazole (0.45 M in acetonitrile, 2.87 mL, 1.29 mmol, 8 equiv) were added at room temperature. Stirring was continued until TLC analysis indicated all starting materials had disappeared (40 min). Upon completion, the reaction was cooled to −20 °C, and *m*-CPBA (181 mg, 0.81 mmol, 5 equiv) was added. Stirring was resumed until TLC analysis indicated these starting materials had disappeared (20 min). The reaction was quenched by saturated aq. NaHCO_3_ (10 mL) and extracted by CH_2_Cl_2_ (15 mL × 2). The separated organic layer was dried over MgSO_4_ and concentrated. The obtained residue was purified by silica gel column chromatography using EtOAc/*n*-hexane (1:1) as eluents to give two diastereomers (ratio = 1:1) of compound **51** as a white powder (289 mg, 93%). No attempt was made to separate the diastereomers, which were directly used for the next step. However, for data analysis, a portion of the mixture was subjected to column chromatography and separated two phosphate diastereomers. Polar phosphate diastereomer: *R*_f_ = 0.26 (silica gel, EtOAc/*n*-hexane = 1:1); ^1^H NMR (600 MH_Z_, CDCl_3_): δ 7.99–7.97 (m, 2H, Ar–H), 7.86–7.82 (m, 5H, Ar–H), 7.79 (s, 1H, Ar–H), 7.66–7.65 (m, 2H, Ar–H), 7.53–7.46 (m, 4H, Ar–H), 7.44–7.41 (m, 1H, Ar–H), 7.36–7.19 (m, 31H, Ar–H), 7.16–7.14 (m, 2H, Ar–H), 7.09–7.07 (m, 2H, Ar–H), 6.91–6.89 (m, 2H, Ar–H), 5.65 (dd, *J* = 9.6, 3.6 Hz, 1H), 5.36 (dd, *J* = 9.0, 7.8 Hz, 1H), 5.34 (s, 1H, Ph–CH), 5.30 (dd, *J* = 3.6, 1.8 Hz, 1H), 5.09 (d, *J* = 11.4 Hz, 1H), 5.05 (d, *J* = 3.6 Hz, 1H, C1–H_α_), 5.01 (d, *J* = 11.4 Hz, 1H), 4.99 (d, *J* = 3.6 Hz, 1H, C1–H_α_), 4.91 (d, *J* = 1.2 Hz, 1H, C1–H_α_), 4.89 (d, *J* = 10.8 Hz, 1H), 4.83 (d, *J* = 7.8 Hz, 1H, C1–H_β_), 4.73–4.69 (m, 4H), 4.61 (d, *J* = 11.4 Hz, 1H), 4.56 (d, *J* = 12.0 Hz, 1H), 4.50–4.40 (m, 4H), 4.20 (dq, *J* = 9.6, 6.0 Hz, 1H), 4.10–4.02 (m, 5H), 3.98 (dd, *J* = 9.6, 9.0 Hz, 1H), 3.95 (ddd, *J* = 10.2, 2.4, 2.4 Hz, 1H), 3.79–3.66 (m, 6H), 3.64–3.59 (m, 3H), 3.56–3.52 (m, 2H), 3.39–3.27 (m, 3H), 3.16 (t, *J* = 7.2 Hz, 2H, –CH_2linker_), 2.30 (t, *J* = 6.6 Hz, 2H, –CH_2_), 1.72 (s, 3H, –CH_3_), 1.58–1.53 (m, 4H, 2-CH_2linker_), 1.36–1.30 (m, 2H, –CH_2linker_), 0.92 (d, *J* = 6.0 Hz, 3H, –CH_3_); ^13^C{^1^H} NMR (150 MHz, CDCl_3_): δ 170.3, 165.2, 164.5, 164.3, 138.3, 138.1, 138.0, 136.9, 136.6, 134.9, 134.9, 133.3, 133.2, 132.8, 129.9, 129.8, 129.6, 129.3, 129.2, 129.0, 128.8, 128.6, 128.5, 128.4, 128.4, 128.3, 128.2, 128.2, 128.1, 128.0, 127.9, 127.9, 127.8, 127.8, 127.8, 127.6, 127.6, 126.3, 126.1, 125.7, 125.5, 125.4, 115.9, 101.7, 101.3, 97.7, 95.9, 94.3, 80.6, 79.9, 79.1, 78.6, 77.8, 77.6, 77.5, 77.5, 76.5, 76.4, 75.6, 75.2, 75.0, 74.8, 73.4, 72.3, 70.4, 69.7, 69.6, 69.6, 68.8, 68.6, 68.5, 68.0, 66.8, 66.8, 66.5, 61.9, 61.5, 51.2, 29.1, 28.6, 23.4, 20.5, 19.1, 19.0, 16.9; ^31^P NMR (162 MH_Z_, CDCl_3_): δ −2.01; HRMS (ESI-TOF) *m*/*z*: [M + Na]^+^ calcd for C_108_H_111_N_4_O_27_PNa, 1949.7066; found, 1949.7037. Nonpolar phosphate diastereomer: *R*_f_ = 0.37 (silica gel, EtOAc/hexane = 1:1); ^1^H NMR (600 MH_Z_, CDCl_3_): δ 7.99–7.97 (m, 2H, Ar–H), 7.91–7.90 (m, 2H, Ar–H), 7.85–7.82 (m, 2H, Ar–H), 7.79 (s, 1H, Ar–H), 7.67–7.65 (m, 2H, Ar–H), 7.53–7.46 (m, 5H, Ar–H), 7.36–7.22 (m, 32H, Ar–H), 7.18–7.16 (m, 2H, Ar–H), 7.14–7.11 (m, 1H, Ar–H), 7.09–7.07 (m, 2H, Ar–H), 5.66 (dd, *J* = 9.6, 3.6 Hz, 1H), 5.36 (dd, *J* = 9.0, 8.4 Hz, 1H), 5.34 (s, 1H, Ph–CH), 5.29 (dd, *J* = 3.6, 1.2 Hz, 1H), 5.09 (d, *J* = 11.4 Hz, 1H), 5.04 (d, *J* = 3.6 Hz, 1H, C1–H_α_), 5.02 (d, *J* = 11.4 Hz, 1H), 4.99 (d, *J* = 3.6 Hz, C1–H_α_), 4.91–4.87 (m, 3H, C1–H_α_), 4.84–4.80 (m, 2H, C1–H_β_), 4.74–4.70 (m, 2H), 4.62 (d, *J* = 11.4 Hz, 1H), 4.56 (d, *J* = 12.0 Hz, 1H), 4.50 (q, *J* = 9.6 Hz, 1H), 4.46 (d, *J* = 12.0 Hz, 1H), 4.41 (d, *J* = 10.8 Hz, 1H), 4.20 (dq, *J* = 9.6, 6.0 Hz, 1H), 4.11–4.09 (m, 1H), 4.06–4.02 (m, 4H), 3.98 (dd, *J* = 9.6, 9.0 Hz, 1H), 3.95 (ddd, *J* = 10.2, 3.0, 3.0 Hz, 1H), 3.78–3.73 (m, 2H), 3.69–3.59 (m, 6H), 3.58–3.53 (m, 3H), 3.40–3.33 (m, 2H), 3.29 (ddd, *J* = 9.6, 9.6, 4.8 Hz, 1H), 3.16 (t, *J* = 7.2 Hz, 2H, –CH_2linker_), 1.98–1.88 (m, 2H, –CH_2_), 1.72 (s, 3H, –CH_3_), 1.58–1.47 (m, 4H, 2-CH_2linker_), 1.36–1.30 (m, 2H, –CH_2linker_), 0.87 (d, *J* = 6.6 Hz, 3H, –CH_3_); ^13^C{^1^H} NMR (150 MHz, CDCl_3_): δ 170.3, 165.1, 164.5, 164.3, 138.3, 138.1, 137.9, 136.8, 136.6, 135.2, 135.1, 133.4, 133.3, 133.2, 133.1, 132.8, 129.9, 129.8, 129.6, 129.3, 129.1, 128.9, 128.8, 128.6, 128.5, 128.4, 128.3, 128.2, 128.2, 128.1, 128.0, 127.9, 127.9, 127.9, 127.8, 127.8, 127.6, 127.6, 126.2, 126.1, 125.7, 125.5, 125.4, 115.8, 101.7, 101.4, 97.8, 95.9, 94.3, 80.6, 79.9, 79.1, 78.5, 77.8, 77.6, 77.6, 77.5, 76.6, 76.4, 75.1,. 75.0, 74.8, 73.4, 72.3, 70.4, 69.7, 69.6, 69.6, 68.8, 68.5, 68.5, 66.8, 66.8, 66.5, 61.9, 61.5, 61.4, 51.1, 29.1, 28.6, 23.4, 20.5, 18.5, 18.4, 16.8; ^31^P NMR (162 MH_Z_, CDCl_3_): δ −1.87; HRMS (ESI-TOF) *m*/*z*: [M + Na]^+^ calcd for C_108_H_111_N_4_O_27_PNa, 1949.7066; found, 1949.7042.

### 5-Aminopentyl α-l-Rhamnopyranosyl-(1→3)-β-d-glucopyranoside (**52**)

To a well-stirred solution of disaccharide **40** (140 mg, 0.17 mmol), a mixture of CH_2_Cl_2_ (2 mL) and NaOMe (2 mL, 0.5 M in MeOH) was added at room temperature. The reaction mixture was vigorously stirred until TLC analysis indicated all starting material had disappeared (3 h). The reaction mixture was neutralized with IR-120, filtered, and concentrated. The obtained residue was dissolved in 4.4 mL of MeOH/H_2_O/AcOH mixture (3:1:0.4); then, 20% Pd(OH)_2_/C (140 mg) was added. The reaction mixture was stirred under a H_2_ atmosphere for 40 h at room temperature. The solution was filtered through a pad of Celite, the solvent was removed, and the obtained residue was purified by flash chromatographywith Sephadex LH-20 (eluent by water) to give compound **52** as a white powder (54 mg, 79%). ^1^H NMR (600 MH_Z_, D_2_O): δ 5.15 (d, *J* = 1.2 Hz, 1H, C1–H_α_), 4.49 (d, *J* = 8.4 Hz, 1H, C1–H_β_), 4.03 (dq, *J* = 9.6, 6.6 Hz, 1H), 3.97–3.93 (m, 2H), 3.80 (dd, *J* = 9.6, 3.6 Hz, 1H), 3.75–7.69 (m, 2H), 3.61 (dd, *J* = 9.0, 9.0 Hz, 1H), 3.49–3.44 (m, 3H), 3.39 (dd, *J* = 9.0, 8.4 Hz, 1H), 3.02 (t, *J* = 7.8 Hz, 2H, –CH_2linker_), 1.74–1.66 (m, 4H, 2-CH_2linker_), 1.50–1.45 (m, 2H, –CH_2linker_), 1.27 (d, *J* = 6.6 Hz, 3H, –CH_3_); ^13^C{^1^H} NMR (150 MHz, D_2_O): δ 101.9, 101.1, 82.2, 75.9, 73.7, 71.9, 70.3, 70.2, 70.1, 68.8, 68.1, 60.7, 39.3, 28.1, 26.4, 22.1, 16.4; HRMS (ESI-TOF) *m*/*z*: [M + H]^+^ calcd for C_17_H_34_NO_10_, 412.2177; found, 412.2198.

### 5-Aminopentyl α-l-Rhamnopyranosyl-(1→3)-β-d-glucopyranosyl-(1→4)-α-d-glucopyranoside (**53**)

To a well-stirred solution of trisaccharide **44** (95 mg, 0.075 mmol) in CH_2_Cl_2_ (1.5 mL), NaOMe (1.5 mL, 0.5 M in MeOH) was added at room temperature. Stirring was continued until TLC analysis indicated all the starting materials had disappeared (1 h). The reaction mixture was neutralized with IR-120, filtered, and concentrated. The residue was dissolved in 3.5 mL of MeOH/H_2_O/AcOH mixture (3:1:0.4); then, 20% Pd(OH)_2_/C (95 mg) was added. The reaction mixture was stirred under a H_2_ atmosphere for 40 h at room temperature. The solution was filtered through a pad of Celite, and the obtained residue was purified by flash chromatography with Sephadex LH-20 (eluent by water) to give compound **53** as a white powder (32 mg, 75%). ^1^H NMR (600 MH_Z_, D_2_O): δ 5.15 (s, 1H, C1–H_α_), 4.93 (d, *J* = 3.6 Hz, 1H, C1–H_α_), 4.55 (d, *J* = 7.8 Hz, 1H, C1–H_β_), 4.07 (dd, *J* = 3.0, 1.8 Hz, 1H), 4.06–4.01 (m, 1H), 3.95–3.92 (m, 2H), 3.87–3.80 (m, 4H), 3.78–3.74 (m, 2H), 3.66–3.62 (m, 3H), 3.58–3.53 (m, 1H), 3.51–3.44 (m, 4H), 3.02 (t, *J* = 7.8 Hz, 2H, –CH_2linker_), 1.74–1.67 (m, 4H, 2-CH_2linker_), 1.52–1.45 (m, 2H, –CH_2linker_), 1.27 (d, *J* = 6.0 Hz, 3H, –CH_3_); ^13^C{^1^H} NMR (150 MHz, D_2_O): δ 102.3, 101.1, 97.8, 82.0, 79.0, 75.9, 73.9, 71.9, 71.7, 71.0, 70.4, 70.3, 70.2, 68.8, 67.9, 67.9, 60.6, 59.9, 39.4, 28.0, 26.6, 22.4, 16.4; HRMS (ESI-TOF) *m*/*z*: [M + H]^+^ calcd for C_23_H_43_NO_15_, 574.2705; found, 574.2743.

### 5-Aminopentyl α-l-Rhamnopyranosyl-(1→3)-β-d-glucopyranosyl-(1→4)-[α-l-Rhamnopyranosyl-(1→3)]-α-d-glucopyranoside (**54**)

To a well-stirred solution of tetrasaccharide **48** (190 mg, 0.12 mmol) inCH_2_Cl_2_ (3 mL), NaOMe (3 mL, 0.5 M in MeOH) was added at room temperature. Stirring was continued until TLC analysis indicated all starting materials had disappeared (24 h). The reaction mixture was neutralized with IR-120, filtered, and concentrated. The residue was dissolved in 4.4 mL of MeOH/H_2_O/AcOH mixture (3:1:0.4); then, 20% Pd(OH)_2_/C (190 mg) was added. The reaction mixture was stirred under a H_2_ atmosphere for 40 h at room temperature. The solution was filtered through a pad of Celite and concentrated. The obtained residue was purified by flash chromatography with Sephadex LH-20 (eluent by water) to give compound **54** as a white powder (71 mg, 82%). ^1^H NMR (600 MH_Z_, D_2_O): δ 5.21 (s, 1H, C1–H_α_), 5.14 (s, 1H, C1–H_α_), 4.91 (d, *J* = 3.6 Hz, 1H, C1–H_α_), 4.52 (d, *J* = 7.8 Hz, 1H, C1–H_β_), 4.46–4.41 (m, 1H), 4.07–4.02 (m, 3H), 3.97–3.80 (m, 9H), 3.77–3.72 (m, 2H), 3.63 (t, *J* = 9.0 Hz, 1H), 3.59–3.55 (m, 1H), 3.49–3.46 (m, 4H), 3.39–3.36 (m, 1H), 3.03 (t, *J* = 7.8 Hz, 2H, –CH_2linker_), 1.75–1.68 (m, 4H, 2-CH_2linker_), 1.55–1.44(m, 2H, –CH_2linker_), 1.28–1.27 (m, 6H, 2-CH_3_); ^13^C{^1^H} NMR (150 MHz, D_2_O): δ 101.3, 100.9, 100.8, 98.0, 82.2, 76.4, 76.1, 74.2, 73.0, 72.2, 71.9, 71.9, 71.2, 70.4, 70.2, 70.1, 70.0, 68.8, 68.4, 68.1, 67.8, 61.1, 59.5, 39.3, 28.1, 26.5, 22.4, 16.5, 16.5; HRMS (ESI-TOF) *m*/*z*: [M + H]^+^ calcd for C_29_H_54_NO_19_, 720.3285; found, 720.3314.

### 5-Aminopentyl α-l-Rhamnopyranosyl-(1→3)-β-d-glucopyranosyl-(1→4)-α-d-glucopyranosyl-(1→2)-α-d-glucopyranoside (**55**)

To a well-stirred solution of tetrasaccharide **50** (170 mg, 0.10 mmol) in CH_2_Cl_2_ (3 mL), NaOMe (3 mL, 0.5 M in MeOH) was added at room temperature. Stirring was continued until TLC analysis indicated all starting materials had disappeared (1 h). Upon completion, the reaction mixture was neutralized with IR-120, filtered, and concentrated. The residue was dissolved in 4.4 mL of MeOH/H_2_O/AcOH mixture (3:1:0.4) and 20% Pd(OH)_2_/C (170 mg) were added. The reaction mixture was stirred under a H_2_ atmosphere for 40 h at room temperature. The solution was filtered through a pad of Celite and concentrated. The obtained residue was purified by flash chromatography with Sephadex LH-20 (eluent by water) to give compound **55** as a white powder (72 mg, 94%). ^1^H NMR (600 MH_Z_, D_2_O): δ 5.17–5.16 (m, 2H, 2C1–H_α_), 5.10 (d, *J* = 3.6 Hz, 1H, C1–H_α_), 4.56 (d, *J* = 7.8 Hz, 1H, C1–H_β_), 4.07–4.02 (m, 3H), 3.96–3.88 (m, 5H), 3.83–3.75 (m, 5H), 3.71–3.58 (m, 6H), 3.52–3.44 (m, 5H), 3.03 (t, *J* = 7.2 Hz, 2H, –CH_2linker_), 1.74–1.68 (m, 4H, 2-CH_2linker_), 1.52–1.49 (m, 2H, –CH_2linker_), 1.27 (d, *J* = 6.0 Hz, 3H, –CH_3_); ^13^C{^1^H} NMR (150 MHz, D_2_O): δ 102.3, 101.1, 95.8, 95.2, 81.9, 78.8, 75.9, 75.2, 73.8, 71.9, 71.6, 71.4, 71.3, 71.0, 70.5, 70.3, 70.2, 69.5, 68.8, 67.9, 67.7, 60.6, 59.7, 39.3, 27.9, 26.5, 22.4, 16.4; HRMS (ESI-TOF) *m*/*z*: [M + H]^+^ calcd for C_29_H_54_NO_20_, 736.3234; found, 736.3268.

### 5-Aminopentyl α-l-Rhamnopyranosyl-(1→3)-β-d-glucopyranosyl-(1→4)-[α-l-Rhamnopyranosyl-(1→3)]-α-d-glucopyranosyl-(1→2)-α-d-glucopyranoside (**4**)

To a well-stirred solution of pentasaccharide **10** (140 mg, 0.070 mmol) in CH_2_Cl_2_ (2.5 mL), NaOMe (2.5 mL, 0.5 M in MeOH) was added at room temperature. The reaction was heated at 40 °C, and stirring was continued until TLC analysis indicated all starting materials had disappeared (16 h). Upon completion, the reaction mixture was neutralized with IR-120, filtered, and concentrated. The obtained residue was purified by silica gel column chromatography using CH_2_Cl_2_/MeOH (10:1) as eluents to give intermediate compound as a white powder (85 mg) for the next step. The obtained compound was dissolved in 4.4 mL of a MeOH/H_2_O/AcOH mixture (3:1:0.4); then, 20% Pd(OH)_2_/C (140 mg) was added. The reaction mixture was stirred under a H_2_ atmosphere for 40 h at room temperature. The solution was filtered through a pad of Celite and concentrated. The obtained residue was purified by flash chromatography with Sephadex LH-20 (eluent by water) to give compound **4** as a white powder (42 mg, 68%). ^1^H NMR (600 MH_Z_, D_2_O): δ 5.24 (d, *J* = 1.2 Hz, 1H, C1–H_α_), 5.18 (d, *J* = 3.6 Hz, 1H, C1–H_α_), 5.14 (d, *J* = 1.2 Hz, 1H, C1–H_α_), 5.09 (d, *J* = 3.6 Hz, 1H, C1–H_α_), 4.54 (d, *J* = 7.8 Hz, 1H, C1–H_β_), 4.46–4.41 (m, 1H), 4.06–4.00 (m, 5H), 3.97 (d, *J* = 11.4 Hz, 1H), 3.92–3.68 (m, 10H), 3.74 (dd, *J =* 10.2, 4.2 Hz, 1H), 3.71–3.68 (m, 2H), 3.64–3.57 (m, 2H), 3.50–3.44 (m, 5H), 3.38 (dd, *J* = 9.0, 8.4 Hz, 1H), 3.03 (t, *J* = 7.8 Hz, 2H, –CH_2linker_), 1.74–1.68 (m, 4H, 2-CH_2linker_), 1.52–1.47 (m, 2H, –CH_2linker_), 1.29–1.27 (m, 6H, 2-CH_3_); ^13^C{^1^H} NMR (150 MHz, D_2_O): δ 101.4, 100.9, 100.7, 95.8, 95.1, 82.1, 76.1, 75.9, 75.0, 74.2, 72.7, 72.3, 72.0, 71.6, 71.4, 71.3, 70.4, 70.2, 70.0, 69.5, 68.8, 68.4, 68.1, 67.7, 61.1, 60.5, 59.2, 39.3, 27.9, 26.5, 22.4, 16.5 16.5; HRMS (ESI-TOF) *m*/*z*: [M + H]^+^ calcd for C_35_H_64_NO_24_, 882.3813; found, 882.3851.

### 5-Aminopentyl 4-*O*-(dihydrogen phosporyl)-α-l-rhamnopyranosyl-(1→3)-β-d-glucopyranoside (**56**)

To a stirred solution of starting material **41** (186 mg, 0.18 mmol, 1 equiv) in CH_2_Cl_2_ (2 mL), a solution of TBAOH (40% in water, 229 mg, 0.35 mmol, 2 equiv) in water (2 mL) was added at room temperature. Stirring was continued until TLC analysis indicated all starting materials had disappeared (4 h). The solution was diluted with CH_2_Cl_2_ (5 mL) and extracted by CH_2_Cl_2_ (5 mL × 2). The separated organic layer was dried over MgSO_4_ and concentrated. The obtained residue was purified by silica gel column chromatography using MeOH/CH_2_Cl_2_ (1:10) as eluents to give compound acid with tetrabutylammonium salt as a white powder (152 mg) for the next step. *R*_f_ = 0.26 (silica gel, MeOH/CH_2_Cl_2_ = 1:10); HRMS (ESI-TOF) *m*/*z*: [M + Na]^+^ calcd for C_52_H_54_N_3_O_16_PNa, 1030.3134; found, 1030.3140. To a well-stirred solution of disaccharide (152 mg) in CH_2_Cl_2_ (2 mL), NaOMe (2 mL, 0.5 M in MeOH) was added at room temperature. Stirring was continued until TLC analysis indicated all starting materials had disappeared (2 h). The reaction mixture was neutralized with IR-120, filtered, and concentrated. The residue was dissolved in 4.4 mL of a MeOH/H_2_O/AcOH mixture (3:1:0.4); then, 20% Pd(OH)_2_/C (152 mg) was added. The reaction mixture was stirred under a H_2_ atmosphere for 40 h at room temperature. The solution was filtered through a pad of Celite and concentrated. The obtained residue was purified by flash chromatography with Sephadex LH-20 (eluent by water) to give compound **56** as a white powder (66 mg, 77%) (3 steps). ^1^H NMR (600 MH_Z_, D_2_O): δ 5.16 (s, 1H, C1–H_α_), 4.48 (d, *J* = 8.4 Hz, 1H, C1–H_β_), 4.08 (br, 2H), 3.97–3.92 (m, 4H), 3.75–3.68 (m, 2H), 3.61 (dd, *J* = 9.0, 8.4 Hz, 1H), 3.49–3.44 (m, 2H), 3.39 (dd, *J* = 9.0, 8.4 Hz, 1H), 3.02 (t, *J* = 7.2 Hz, 2H, –CH_2linker_), 1.74–1.66 (m, 4H, 2-CH_2linker_), 1.50–1.45 (m, 2H, –CH_2linker_), 1.30 (d, *J* = 6.0 Hz, 3H, –CH_3_); ^13^C{^1^H} NMR (150 MHz, D_2_O): δ 101.9, 100.6, 82.4, 75.9, 75.8, 75.7, 73.7, 70.6, 70.0, 70.0, 68.0, 67.8, 67.8, 60.8, 39.3, 28.1, 26.4, 22.1, 16.5; ^31^P NMR (162 MH_Z_, D_2_O): δ 2.57; HRMS (ESI-TOF) *m*/*z*: [M + H]^+^ calcd for C_17_H_35_NO_13_P, 492.1841; found, 492.1841.

### 5-Aminopentyl 4-*O*-(dihydrogen phosporyl)-α-l-rhamnopyranosyl-(1→3)-β-d-glucopyranosyl-(1→4)-α-d-glucopyranoside (**57**)

To a stirred solution of starting material **45** (195 mg, 0.13 mmol, 1 equiv) in CH_2_Cl_2_ (2.5 mL), a solution of TBAOH (40% in water, 169 mg, 0.26 mmol, 2 equiv) in water (2.5 mL) was added at room temperature. Stirring was continued until TLC analysis indicated all starting materials had disappeared (4 h). The solution was diluted with CH_2_Cl_2_ (5 mL) and extracted by CH_2_Cl_2_ (5 mL × 2). The separated organic layer was dried over MgSO_4_ and concentrated. The obtained residue was purified by silica gel column chromatography using MeOH/CH_2_Cl_2_ (1:10) as eluents to give compound acid with tetrabutylammonium salt as a white powder (182 mg) for the next step. *R*_f_ = 0.29 (silica gel, MeOH/CH_2_Cl_2_ = 1:8); HRMS (ESI-TOF) *m*/*z*: [M + Na]^+^ calcd for C_78_H_80_N_3_O_22_PNa, 1464.4863; found, 1464.4865. To a well-stirred solution of trisaccharide (182 mg) in CH_2_Cl_2_ (2 mL), NaOMe (2 mL, 0.5 M in MeOH) was added at room temperature. Stirring was continued until TLC analysis indicated all starting materials had disappeared (1 h). Upon completion, the reaction mixture was neutralized with IR-120, filtered, and concentrated. The residue was dissolved in 4.4 mL of MeOH/H_2_O/AcOH mixture (3:1:0.4), and 20% Pd(OH)_2_/C (182 mg) was added. The reaction mixture was stirred under a H_2_ atmosphere for 40 h at room temperature. The solution was filtered through a pad of Celite and concentrated. The obtained residue was purified by flash chromatography with Sephadex LH-20 (eluent by water) to give compound **57** as a white powder (61 mg, 72%) (3 steps). ^1^H NMR (600 MH_Z_, D_2_O): δ 5.17 (s, 1H, C1–H_α_), 4.93 (s, 1H, C1–H_α_), 4.55 (d, *J* = 7.8 Hz, 1H, C1–H_β_), 4.13–4.09 (m, 2H), 4.02–3.94 (m, 4H), 3.87–3.75 (m, 5H), 3.66–3.63 (m, 3H), 3.57 (br, 1H), 3.52–3.49 (m, 2H), 3.46 (t, *J* = 8.4 Hz, 1H), 3.04 (br, 2H, –CH_2linker_), 1.71 (br, 4H, 2-CH_2linker_), 1.50 (br, 2H, –CH_2linker_), 1.31 (d, *J* = 6.0 Hz, 3H, –CH_3_); ^13^C{^1^H} NMR (150 MHz, D_2_O): δ 102.3, 100.6, 97.8, 82.1, 79.0, 76.4, 76.4, 75.9, 73.8, 71.7, 71.0, 70.4, 70.3, 70.0, 67.9, 67.7, 67.7, 60.6, 59.9, 39.4, 28.0, 26.5, 22.4, 16.5; ^31^P NMR (162 MH_Z_, D_2_O): δ 1.35; HRMS (ESI-TOF) *m*/*z*: [M + H]^+^ calcd for C_23_H_45_NO_18_P, 654.2369; found, 654.2392.

### 5-Aminopentyl 4-*O*-(dihydrogen phosporyl)-α-l-rhamnopyranosyl-(1→3)-β-d-glucopyranosyl-(1→4)-[α-l-rhamnopyranosyl-(1→3)]-α-d-glucopyranoside (**58**)

To a stirred solution of starting material **49** (200 mg, 0.111 mmol, 1 equiv) in CH_2_Cl_2_ (2 mL), a solution of TBAOH (40% in water, 144 mg, 0.222 mmol, 2 equiv) in water (2 mL) was added at room temperature. Stirring was continued until TLC analysis indicated all starting materials had disappeared (4 h). The solution was diluted with CH_2_Cl_2_ (5 mL) and extracted by CH_2_Cl_2_ (5 mL × 2). The separated organic layer was dried over MgSO_4_ and concentrated. The obtained residue was purified by silica gel column chromatography using MeOH/CH_2_Cl_2_ (1:10) as eluents to give compound acid with tetrabutylammonium salt as a white powder (184 mg) for the next step. *R*_f_ = 0.31 (silica gel, MeOH/CH_2_Cl_2_ = 1:8); HRMS (ESI-TOF) *m*/*z*: [M + Na]^+^ calcd for C_94_H_96_N_3_O_28_PNa, 1768.5810; found, 1768.5800. To a well-stirred solution of tetrasaccharide (184 mg) in CH_2_Cl_2_ (2 mL), NaOMe (2 mL, 0.5 M in MeOH) was added at room temperature. Stirring was continued until TLC analysis indicated all starting materials had disappeared (16 h). Upon completion, the reaction mixture was neutralized with IR-120, filtered, and concentrated. The residue was dissolved in 4.4 mL of the MeOH/H_2_O/AcOH mixture (3:1:0.4); then, 20% Pd(OH)_2_/C (184 mg) was added. The reaction mixture was stirred under a H_2_ atmosphere for 40 h at room temperature. The solution was filtered through a pad of Celiteand concentrated. The obtained residue was purified by flash chromatography with Sephadex LH-20 (eluent by water) to give compound **58** as a white powder (67 mg, 75%) (3 steps). ^1^H NMR (600 MH_Z_, D_2_O): δ 5.21 (s, 1H, C1–H_α_), 5.16 (d, *J* = 1.2 Hz, 1H, C1–H_α_), 4.91 (d, *J* = 3.6 Hz, 1H, C1–H_α_), 4.53 (d, *J* = 8.4 Hz, 1H, C1–H_β_), 4.46–4.41 (m, 1H), 4.11–4.09 (m, 2H), 4.06 (dd, *J* = 3.0, 1.2 Hz, 1H), 3.99–3.80 (m, 10H), 3.77–3.72 (m, 2H), 3.63 (dd, *J* = 9.0, 9.0 Hz, 1H), 3.59–3.55 (m, 1H), 3.49–3.43 (m, 3H), 3.40–3.37 (m, 1H), 3.03 (t, *J* = 7.2 Hz, 2H, –CH_2linker_), 1.75–1.66 (m, 4H, 2-CH_2linker_), 1.55–1.45 (m, 2H, –CH_2linker_), 1.31 (d, *J* = 6.6 Hz, 3H, –CH_3_), 1.28 (d, *J* = 6.0 Hz, 3H, –CH_3_); ^13^C{^1^H} NMR (150 MHz, D_2_O): δ 101.3, 100.9, 100.4, 98.1, 82.4, 76.4, 76.3, 76.1, 74.2, 73.0, 72.2, 71.9, 71.2, 70.4, 70.1, 70.1, 70.0, 68.4, 68.0, 67.8, 67.7, 61.1, 59.5, 39.3, 28.1, 26.5, 22.4, 16.5, 16.5; ^31^P NMR (162 MH_Z_, D_2_O): δ 1.65; HRMS (ESI-TOF) *m*/*z*: [M + H]^+^ calcd for C_29_H_55_NO_22_P, 800.2948; found, 800.3010.

### 5-Aminopentyl 4-*O*-(dihydrogen phosporyl)-α-l-rhamnopyranosyl-(1→3)-β-d-glucopyranosyl-(1→4)-α-d-glucopyranosyl-(1→2)-α-d-glucopyranoside (**59**)

To a stirred solution of starting material **51** (185 mg, 0.082 mmol, 1 equiv) in CH_2_Cl_2_ (2 mL), a solution of TBAOH (40% in water, 106 mg, 0.16 mmol, 2 equiv) in water (2 mL) was added at room temperature. Stirring was continued until TLC analysis indicated all starting materials had disappeared (4 h). The solution was diluted with CH_2_Cl_2_ (5 mL) and extracted by CH_2_Cl_2_ (5 mL × 2). The separated organic layer was dried over MgSO_4_ and concentrated. The obtained residue was purified by silica gel column chromatography using MeOH/CH_2_Cl_2_ (1:10) as eluents to give compound acid with tetrabutylammonium salt as a white powder (171 mg) for the next step. *R*_f_ = 0.26 (silica gel, MeOH/CH_2_Cl_2_ = 1:10); HRMS (ESI-TOF) *m*/*z*: [M + Na]^+^ calcd for C_105_H_108_N_3_O_27_PNa, 1896.6800; found, 1896.6784. To a well-stirred solution of pentasaccharide (171 mg) in CH_2_Cl_2_ (2 mL), NaOMe (2 mL, 0.5 M in MeOH) was added at room temperature. Stirring was continued until TLC analysis indicated all starting materials had disappeared (1 h). Upon completion, the reaction mixture was neutralized with IR-120, filtered, and concentrated. The residue was dissolved in 4.4 mL of a MeOH/H_2_O/AcOH mixture (3:1:0.4); then, 20% Pd(OH)_2_/C (171 mg) was added. The reaction mixture was stirred under a H_2_ atmosphere for 40 h at room temperature. The solution was filtered through a pad of Celite and concentrated. The obtained residue was purified by flash chromatography with Sephadex LH-20 (eluent by water) to give compound **59** as a white powder (51 mg, 65%) (3 steps). ^1^H NMR (600 MH_Z_, D_2_O): δ 5.17–5.16 (m, 2H, 2C1–H_α_), 5.10 (d, *J* = 3.6 Hz, 1H, C1–H_α_), 4.57 (d, *J* = 8.4 Hz, 1H, C1–H_β_), 4.13–4.05 (m, 3H), 4.01–3.88 (m, 7H), 3.83–3.75 (m, 4H), 3.71–3.57 (m, 6H), 3.55–3.45 (m, 4H), 3.04 (br, 2H, –CH_2linker_), 1.72–1.69 (m, 4H, 2-CH_2linker_), 1.50 (br, 2H, –CH_2linker_), 1.31 (d, *J* = 6.0, 3H, –CH_3_); ^13^C{^1^H} NMR (150 MHz, D_2_O): δ 102.3, 100.6, 95.8, 95.2, 82.1, 78.8, 76.6, 75.9, 75.3, 73.8, 71.6, 71.5, 71.3, 71.0, 70.5, 70.3, 70.1, 69.5, 67.9, 67.7, 67.7, 60.6, 59.6, 39.4, 27.9, 26.5, 22.4, 16.5; ^31^P NMR (162 MH_Z_, D_2_O): δ 1.05; HRMS (ESI-TOF) *m*/*z*: [M – H]^−^ calcd for C_29_H_53_NO_23_P, 814.2740; found, 814.2756.

### 5-Aminopentyl 4-*O*-(dihydrogen phosporyl)-α-l-Rhamnopyranosyl-(1→3)-β-d-glucopyranosyl-(1→4)-[α-l-rhamnopyranosyl-(1→3)]-α-d-glucopyranosyl-(1→2)-α-d-glucopyranoside (**5**)

To a well-stirred solution of pentasaccharide **11** (166 mg) in CH_2_Cl_2_ (2 mL), NaOMe (2 mL, 0.5 M in MeOH) was added at room temperature. Stirring was continued until TLC analysis indicated all starting materials had disappeared (16 h). The reaction mixture was neutralized with IR-120, filtered, and concentrated. The residue was dissolved in 4.4 mL of a MeOH/H_2_O/AcOH mixture (3:1:0.4); then, 20% Pd(OH)_2_/C (166 mg) was added. The reaction mixture was stirred under a H_2_ atmosphere for 40 h at room temperature. The solution was filtered through a pad of Celite and concentrated. The obtained residue was purified by flash chromatography with Sephadex LH-20 (eluent by water) to give compound **5** as a white powder (60 mg, 82%) (2 steps). ^1^H NMR (600 MH_Z_, D_2_O): δ 5.23 (s, 1H, C1–H_α_), 5.17 (d, *J* = 3.0 Hz, 1H, C1–H_α_), 5.15 (s, 1H, C1–H_α_), 5.08 (d, *J* = 3.0 Hz, 1H, C1–H_α_), 4.54 (d, *J* = 7.8 Hz, 1H, C1–H_β_), 4.43 (dq, *J* = 9.6, 6.6 Hz, 1H), 4.10–4.02 (m, 5H), 4.00–3.96 (m, 3H), 3.92–3.77 (m, 9H), 3.73 (dd, *J* = 9.6, 3.6 Hz, 1H), 3.70–3.68 (m, 2H), 3.64–3.57 (m, 2H), 3.49–3.43 (m, 4H), 3.39–3.37 (m, 1H), 3.02 (br, 2H, –CH_2linker_), 1.72–1.68 (m, 4H, 2-CH_2linker_), 1.50–1.49 (m, 2H, –CH_2linker_), 1.30 (d, *J* = 6.0 Hz, 3H, –CH_3_), 1.28 (d, *J* = 6.6 Hz, 3H); ^13^C{^1^H} NMR (150 MHz, D_2_O): δ 101.3, 100.7, 100.5, 95.8, 95.1, 82.3, 76.4, 76.1, 75.9, 75.1, 74.2, 72.7, 72.3, 71.9, 71.6, 71.4, 71.3, 70.4, 70.2, 70.1, 70.0, 69.5, 68.5, 68.0, 67.7, 61.2, 60.6, 59.2, 39.3, 27.9, 26.5, 22.4, 16.5, 16.5; ^31^P NMR (162 MH_Z_, D_2_O): δ 1.55; HRMS (ESI-TOF) *m*/*z*: [M – H]^−^ calcd for C_35_H_63_NO_27_P, 960.3320; found, 960.3304.

### 5-Aminopentyl α-d-glucopyranosyl-(1→dihydrogen phosporyl→4)-α-l-rhamnopyranosyl-(1→3)-β-d-glucopyranosyl-(1→4)-[α-l-Rhamnopyranosyl-(1→3)]-α-d-glucopyranosyl-(1→2)-α-d-glucopyranoside (**3**)

**Method 1:** To a stirred solution of compound **29** (172 mg, 0.064 mmol, 1 equiv) in acetonitrite (2 mL), NaI (19.1 mg, 0.13 mmol, 2eq) was added with a high-pressure tube. The reaction mixture was heated to 90 °C. Stirring was continued until TLC analysis indicated all starting materials had disappeared (24 h). Upon completion, the solvent was removed, and the residue was purified by silica gel column chromatography using MeOH/CH_2_Cl_2_ (1:20) as eluents to give debenzyl hexasaccharide as a white powder (137 mg) for the next step and pentasaccharide **4** as a white powder (22 mg). *R*_f_ = 0.31 (silica gel, MeOH/CH_2_Cl_2_ = 1:8); HRMS (ESI-TOF) *m*/*z*: [M + Na]^+^ calcd for C_148_H_152_N_3_O_38_PNa, 2632.9684; found, 2632.9632. To a well-stirred solution of hexasaccharide (137 mg) in CH_2_Cl_2_ (2 mL), NaOMe (4 mL, 0.2 M in MeOH) was added at room temperature. Stirring was continued until TLC analysis indicated all starting materials had disappeared (16 h). The reaction mixture was neutralized with IR-120, filtered, and concentrated. The residue was dissolved in 4.4 mL of a MeOH/H_2_O/AcOH mixture (3:1:0.4); then, 20% Pd(OH)_2_/C (137 mg) was added. The reaction mixture was stirred under a H_2_ atmosphere for 40 h at room temperature. The solution was filtered through a pad of Celite and concentrated. The obtained residue was purified by flash chromatography with Sephadex LH-20 (eluent by water) (α/β = 6:1) and reverse-phase column chromatography Bond Eluct-C18 (eluent by H_2_O/MeOH = 95:5) to give compound **5** as a white powder (24 mg, 34%) (α/β = 9:1) (3 steps). **Method 2**: Small pieces of lithium (50 mg) were added to a solution of hexamer **29** (30 mg, 0.011 mmol, 1 equiv) in THF (5 mL) and liquid ammonia (20 mL) at −78 °C until the blue color persisted. The mixture was stirred for 1.5 h at −78 °C, and a small amount of NH_4_Cl (600 mg) was carefully added to destroy the excess amount of lithium. The reaction mixture was slowly warmed to room temperature overnight. The residue was dissolved in a mixture of 1-butanol (30 mL) and water (20 mL). The butanol was separated and extracted by water (10 mL × 2). The water layer was removed, and the obtained residue was purified by flash chromatography with Sephadex LH-20 (eluent by water) to give compound acid with some Benzoyl group without deprotection as a white powder (9.8 mg). To a well-stirred solution of hexasaccharide (9.8 mg), NaOMe (2 mL, 0.2 M in MeOH) was added at room temperature. Stirring was continued for 16 h. Upon completion, the reaction mixture was neutralized with IR-120, and pH was adjusted to 7. Then, the mixture was filtered and concentrated. The solution was filtered through a pad of Celite and concentrated. The obtained residue was purified by flash reverse-phase column chromatography Bond Eluct-C18 (eluent by H_2_O/MeOH = 95:5) to give compound **3** as a white powder (α isomer only) (6.1 mg, 49%). α isomer ^1^H NMR (600 MH_Z_, D_2_O): δ 5.56 (dd, *J* = 7.2, 3.6 Hz, 1H, C1–H_α_), 5.24 (d, *J* = 1.2 Hz, 1H, C1–H_α_), 5.18 (d, *J* = 3.6 Hz, 1H, C1–H_α_), 5.16 (d, *J* = 1.8 Hz, 1H, C1–H_α_), 5.09 (d, *J* = 3.6 Hz, 1H, C1–H_α_), 4.54 (d, *J* = 7.8 Hz, 1H, C1–H_β_), 4.44 (dq, *J* = 9.6, 6.6 Hz, 1H), 4.14–4.09 (m, 3H), 4.07–4.03 (m, 3H), 3.98–3.95 (m, 2H), 3.93–3.85 (m, 7H), 3.83–3.73 (m, 7H), 3.72–3.67 (m, 2H), 3.65–3.57 (m, 3H), 3.50–3.46 (m, 5H), 3.40–3.37 (m, 1H), 3.02 (t, *J* = 7.2 Hz, 2H, –CH_2linker_), 1.74–1.68 (m, 4H, 2-CH_2linker_), 1.52–1.48 (m, 2H, –CH_2linker_), 1.33 (d, *J* = 5.4 Hz, 3H, –CH_3_), 1.28 (d, *J* = 6.6 Hz, 3H, –CH_3_); ^13^C{^1^H} NMR (150 MHz, D_2_O): δ 101.3, 100.7, 100.6, 95.8, 95.3, 95.2, 95.1, 82.2, 78.0, 78.0, 76.1, 75.9, 75.1, 74.2, 72.8, 72.7, 72.6, 72.4, 71.9, 71.6, 71.5, 71.3, 70.3, 70.2, 70.0, 69.8, 69.5, 69.2, 68.5, 68.1, 67.7, 67.7, 61.2, 60.6, 60.3, 59.2, 39.4, 27.9, 26.6, 22.4, 16.6, 16.5; ^31^P NMR (162 MH_Z_, D_2_O): δ −1.16; HRMS (ESI-TOF) *m*/*z*: [M – H]^−^ calcd for 1122.3848; found, 1122.3864.

### 5-Aminopentyl α-d-glucopyranosyl-(1→2)-α-d-glucopyranosyl-(1→dihydrogen phosporyl→4)-α-l-rhamnopyranosyl-(1→3)-β-d-glucopyranosyl-(1→4)-[α-l-rhamnopyranosyl-(1→3)]-α-d-glucopyranosyl-(1→2)-α-d-glucopyranoside (**2**)

To a stirred solution of compound **30** (200 mg, 0.067 mmol, 1 equiv) in acetonitrite (2 mL), NaI (20.0 mg, 0.13 mmol, 2eq) was added with a high-pressure tube. The reaction mixture was heated to 90 °C. Stirring was continued until TLC analysis indicated all starting materials had disappeared (24 h). Upon completion, the solvent was removed, and the residue was purified by silica gel column chromatography using MeOH/CH_2_Cl_2_ = 1:20 as eluents to give debenzyl heptasaccharide as a white powder (184 mg) for the next step and pentasaccharide **4** as a white powder (7 mg). *R*_f_ = 0.35 (silica gel, MeOH/CH_2_Cl_2_ = 1:8); HRMS (ESI-TOF) *m*/*z*: [M + Na]^+^ calcd for C_163_H_168_N_3_O_44_PNa 2925.0631; found, 2925.0626. To a well-stirred solution of hexasaccharide (184 mg) in CH_2_Cl_2_ (2.5 mL), NaOMe (5 mL, 0.2 M in MeOH) was added at room temperature. Stirring was continued until TLC analysis indicated all starting materials had diappeared (16 h). The reaction mixture was neutralized with IR-120, filtered, and concentrated. The residue was dissolved in 6.6 mL of a MeOH/H_2_O/AcOH mixture (3:1:0.4); then, 20% Pd(OH)_2_/C (184 mg) was added. The reaction mixture was stirred under a H_2_ atmosphere for 72 h at room temperature. The solution was filtered through a pad of Celite and concentrated. The obtained residue was purified by flash chromatography with Sephadex LH-20 (eluent by water) (α/β = 10:1) and reverse-phase column chromatography Bond Eluct-C18 (eluent by H_2_O/MeOH = 95:5) to give compound **2** as a white powder (24 mg, 28%) (α/β = 20:1) (3 steps). **Method 2:** Small pieces of lithium (50 mg) were added to a solution of hexamer **30** (30 mg, 0.011 mmol, 1 equiv) in THF (5 mL) and liquid ammonia (20 mL) at −78 °C until the blue color persisted. The mixture was stirred for 1.5 h at −78 °C, and a small amount of NH_4_Cl (600 mg) was carefully added to destroy the excess amount of lithium. The reaction mixture was slowly warmed to room temperature overnight. The residue was dissolved in a mixture of 1-butanol (30 mL) and water (20 mL). The butanol was separated and extracted by water (10 mL × 2). The water layer was removed and the obtained residue was purified by flash chromatography with Sephadex LH-20 (eluent by water) to give compound acid with some Benzoyl group without deprotection as a white powder (11.0 mg). To a well-stirred solution of hexasaccharide (11.0 mg), NaOMe (2 mL, 0.2 M in MeOH) was added at room temperature. Stirring was continued for 16 h. Upon completion, the reaction mixture was neutralized with IR-120, and pH was adjusted to 7. Then, the reaction mixture was filtered through a pad of Celite and concentrated. The obtained residue was purified by flash reverse-phase column chromatography Bond Eluct-C18 (eluent by H_2_O/MeOH = 95:5) to give compound **2** as a white powder (α isomer only) (4.4 mg, 34%). α isomer ^1^H NMR (600 MH_Z_, D_2_O): δ 5.77 (dd, *J* = 7.2, 3.6 Hz, 1H, C1–H_α_), 5.25 (s, 1H, C1–H_α_), 5.19 (d, *J* = 3.6 Hz, 1H, C1–H_α_), 5.17 (s, 1H, C1–H_α_), 5.15 (d, *J* = 4.2 Hz, 1H, C1–H_α_), 5.09 (d, *J* = 4.2 Hz, 1H, C1–H_α_), 4.55 (d, *J* = 7.8 Hz, 1H, C1–H_β_), 4.44 (dq, *J* = 9.6, 6.6 Hz, 1H), 4.15–3.77 (m, 25H), 3.74 (dd, *J* = 9.6, 3.6 Hz, 1H), 3.71–3.69 (m, 3H), 3.65–3.53 (m, 4H), 3.50–3.44 (m, 5H), 3.39 (dd, *J* = 9.0, 8.4 Hz, 1H), 3.03 (t, *J* = 7.8 Hz, 2H, –CH_2linker_), 1.75–1.68 (m, 4H, 2-CH_2linker_), 1.52–1.50 (m, 2H, –CH_2linker_), 1.35 (d, *J* = 6.0 Hz, 3H, –CH_3_), 1.29 (d, *J* = 6.0 Hz, 3H, –CH_3_); ^13^C{^1^H} NMR (150 MHz, D_2_O): δ 101.3, 100.7, 100.5, 97.2, 95.9, 95.1, 92.7, 92.7, 82.2, 77.8, 77.8, 76.6, 76.5, 76.1, 75.9, 75.1, 74.2, 72.8, 72.8, 72.7, 72.4, 72.0, 71.8, 71.6, 71.5, 71.5, 71.3, 71.1, 70.2, 70.2, 70.0, 69.9, 69.5, 69.3, 69.0, 68.5, 68.0, 67.7, 67.6, 61.2, 60.6, 60.2, 60.2, 59.2, 39.3, 27.9, 26.5, 22.4, 16.7, 16.5; ^31^P NMR (162 MH_Z_, D_2_O): δ −0.73; HRMS (ESI-TOF) *m*/*z*: [M + H]^+^ calcd for C_47_H_85_NO_37_P, 1286.4553; found, 1286.4542.

### 5-Aminopentyl α-l-rhamnopyranosyl-(1→3)-β-d-glucopyranosyl-(1→4)-[α-l-rhamnopyranosyl-(1→3)]-α-d-glucopyranosyl-(1→2)-α-d-glucopyranosyl-(1→dihydrogen phosporyl→4)-α-l-rhamnopyranosyl-(1→3)-β-d-glucopyranosyl-(1→4)-[α-l-Rhamnopyranosyl-(1→3)]-α-d-glucopyranosyl-(1→2)-α-d-glucopyranoside (**1**)

To a stirred solution of compound **36** (160 mg, 0.039 mmol, 1 equiv) in acetonitrite (1.3 mL), NaI (11.8 mg, 0.079 mmol, 2 eq) was added with a high-pressure tube. The reaction mixture was heated to 90 °C. Stirring was continued until TLC analysis indicated all starting materials had disappeared (24 h). The solvent was removed, and the residue was purified by silica gel column chromatography using MeOH/CH_2_Cl_2_ (1:20) as eluents to give debenzyl hexasaccharide as a white powder (137 mg) for the next step and pentasaccharide **4** as a white powder (22 mg). *R*_f_ = 0.57 (silica gel, MeOH/CH_2_Cl_2_ = 1:8); HRMS (ESI-TOF) *m*/*z*: [M + NaH]^2+^ calcd for C_221_H_223_N_3_O_64_PNa, 1998.1956; found, 1998.1635. To a well-stirred solution of hexasaccharide (151 mg) in CH_2_Cl_2_ (2.5 mL), NaOMe (5 mL, 0.2 M in MeOH) was added at room temperature. Stirring was continued until TLC analysis indicated all starting materials had disappeared (40 h). The reaction mixture was neutralized with IR-120, filtered, and concentrated. The residue was dissolved in 4.4 mL of a MeOH/H_2_O/AcOH mixture (3:1:0.4); then, 20% Pd(OH)_2_/C (151 mg) was added. The reaction mixture was stirred under a H_2_ atmosphere for 40 h at room temperature. The solution was filtered through a pad of Celite and concentrated. The obtained residue was purified by flash chromatography with Sephadex LH-20 (eluent by water) (α/β = 5:1) and reverse-phase column chromatography Bond Eluct-C18 (eluent by H_2_O/MeOH = 93:7) to give compound **1** as a white powder (19 mg, 28%) (α isomer only) (3 steps). **Method 2**: Small pieces of lithium (50 mg) were added to a solution of hexamer **36** (30 mg, 0.011 mmol, 1 equiv) in THF (5 mL) and liquid ammonia (20 mL) at −78 °C until the blue color persisted. The mixture was stirred for 1.5 h at −78 °C, and a small amount of NH_4_Cl (600 mg) was carefully added to destroy the excess amount of lithium. The reaction mixture was slowly warmed to room temperature overnight and concentrated. The residue was dissolved in a mixture of 1-butanol (30 mL) and water (20 mL). The butanol was separated and extracted by water (10 mL × 2). The water layer was removed, and the obtained residue was purified by flash chromatography with Sephadex LH-20 (eluent by water) to give compound acid with Benzoyl group without deprotection as a white powder (9.4 mg). To a well-stirred solution of hexasaccharide (9.4 mg), NaOMe (2 mL, 0.2 M in MeOH) was added at room temperature. Stirring was continued for 16 h. Upon completion, the reaction mixture was neutralized with IR-120, and pH was adjusted to 7. Then, the reaction solution was filtered through a pad of Celite and concentrated. The obtained residue was purified by flash reverse-phase column chromatography Bond Eluct-C18 (eluent by H_2_O/MeOH = 93:7) to give compound **1** as a white powder (α isomer only) (3.1 mg, 24%). α isomer ^1^H NMR (600 MH_Z_, D_2_O): δ 5.76 (dd, *J* = 7.8, 3.0 Hz, 1H, C1–H_α_), 5.26 (br, 2H, 2C1–H_α_), 5.19 (d, *J* = 3.0 Hz, 1H, C1–H_α_), 5.17 (d, *J* = 1.2 Hz, 1H, C1–H_α_), 5.16 (d, *J* = 1.2 Hz, 1H, C1–H_α_), 5.13 (d, *J* = 4.2 Hz, 1H, C1–H_α_), 5.10 (d, *J* = 4.2 Hz, 1H, C1–H_α_), 4.55 (d, *J* = 7.8 Hz, 2H, 2C1–H_β_), 4.49–4.42 (m, 2H), 4.15–4.03 (m, 10H), 4.02–3.86 (m, 16H), 3.84–3.78 (m, 7H), 3.75 (dd, *J* = 9.6, 3.6 Hz, 1H), 3.73–3.69 (m, 4H), 3.66–3.63 (m, 2H), 3.62–3.58 (m, 1H), 3.55 (dd, *J* = 9.6, 9.6 Hz, 1H), 3.51–3.45 (m, 8H), 3.51–3.38 (m, 2H), 3.04 (t, *J* = 7.2 Hz, 2H, –CH_2linker_), 1.76–1.69 (m, 4H, 2-CH_2linker_), 1.53–1.48 (m, 2H, –CH_2linker_), 1.35 (d, *J* = 6.0 Hz, 3H, –CH_3_), 1.30–1.28 (m, 9H, 3-CH_3_); ^13^C{^1^H} NMR (150 MHz, D_2_O): δ 101.4, 101.3, 100.9, 100.9, 100.7, 100.5, 97.5, 95.9, 95.1, 92.7, 92.6, 82.3, 82.1, 77.8, 77.2, 77.1, 76.7, 75.9, 75.1, 74.3, 74.2, 72.9, 72.7, 72.6, 72.4, 72.0, 71.6, 71.5, 71.3, 71.1, 70.4, 70.2, 70.0, 69.5, 69.1, 68.5, 68.4, 68.1, 67.7, 61.1, 60.2, 59.2, 59.2, 39.3, 27.9, 26.5, 22.4, 16.8, 16.5, 16.5, 16.5; ^31^P NMR [(162 MH_Z_, D_2_O): δ −0.47; HRMS (ESI-TOF)] *m*/*z*: [M – H]^−^ calcd for C_65_H_113_NO_50_P, 1738.6062; found, 1738.6054.

### (5-Aminopentyl-phosphate) α-l-rhamnopyranosyl-(1→3)-β-d-glucopyranosyl-(1→4)-[α-l-rhamnopyranosyl-(1→3)]-α-d-glucopyranosyl-(1→2)-α-d-glucopyranoside (**60**)

Small pieces of lithium (50 mg) were added to a solution of hexamer **38** (30 mg, 0.011 mmol, 1 equiv) in THF (5 mL) and liquid ammonia (20 mL) at −78 °C until the blue color persisted. The reaction mixture was stirred for 1.5 h at −78 °C, and a small amount of NH_4_Cl (600 mg) was then carefully added to destroy the excess amount of lithium. The reaction mixture was slowly warmed to room temperature overnight. The residue was dissolved in a mixture of 1-butanol (30 mL) and water (20 mL). The butanol was separated and extracted by water (10 mL × 2). The water layer was removed, and the obtained residue was purified by flash chromatography with Sephadex LH-20 (eluent by water) to give compound acid with the benzoyl group without deprotection as a white powder (9.6 mg). To a well-stirred solution of hexasaccharide (9.6 mg), NaOMe (2 mL, 0.2 M in MeOH) was added at room temperature. Stirring was continued for 16 h. Upon completion, the reaction mixture was neutralized with IR-120, and pH was adjusted to 7. Then, the reaction was filtered through a pad of Celite and concentrated. The obtained residue was purified by flash reverse-phase column chromatography Bond Eluct-C18 (eluent by H_2_O/MeOH = 95:5) to give compound **60** as white powder (α isomer only) (6.1 mg, 46%). ^1^H NMR (600 MH_Z_, D_2_O): δ 5.67 (dd, *J* = 7.8, 3.6 Hz, 1H, C1–H_α_), 5.22 (d, *J* = 1.2 Hz, 1H, C1–H_α_), 5.15 (d, *J* = 1.8 Hz, 1H, C1–H_α_), 5.13 (d, *J* = 3.6 Hz, 1H, C1–H_α_), 4.54 (d, *J* = 7.8 Hz, 1H, C1–H_β_), 4.44 (dq, *J* = 9.6, 6.0 Hz, 1H), 4.09–4.06 (m, 3H), 4.05–4.01 (m, 2H), 3.99–3.86 (m, 10H), 3.83–3.80 (m, 3H), 3.70–3.67 (m, 2H), 3.63 (dd, *J* = 9.0, 9.0 Hz, 1H), 3.52 (dd, *J* = 10.2, 9.6 Hz, 1H), 3.49–3.46 (m, 4H), 3.38 (dd, *J* = 9.0, 8.4 Hz, 1H), 3.04 (t, *J* = 7.8 Hz, 2H, –CH_2linker_), 1.76–1.69 (m, 4H, 2-CH_2linker_), 1.53–1.48 (m, 2H, –CH_2linker_), 1.28–1.27 (m, 6H, 2-CH_3_); ^13^C{^1^H} NMR (150 MHz, D_2_O): δ 101.3, 100.9, 100.9, 97.2, 92.4, 92.4, 82.1, 76.8, 76.7, 76.5, 76.1, 74.2, 72.8, 72.6, 72.6, 72.0, 71.4, 71.1, 70.4, 70.2, 70.1, 70.0, 69.2, 68.8, 68.4, 68.1, 66.1, 66.1, 61.1, 60.3, 59.2, 39.4, 29.1, 29.1, 26.3, 22.0, 16.5, 16.5; ^31^P NMR (162 MH_Z_, D_2_O): δ −0.28; HRMS (ESI-TOF) *m*/*z*: [M + H]^+^ calcd for C_35_H_65_NO_27_P, 962.3476; found, 962.3485.

## Data Availability

The data underlying this study are available in the published article and its Supporting Information.

## Supporting Information Available

Detailed experimental procedures, characterization information, and copies of NMR spectra (PDF)
