# Supplementary material for: Synthesis of PSI Oligosaccharide for the Development of Total Synthetic Vaccine against Clostridium difficile
Source: J Org Chem. 2025 Apr 11;90(16):5586–613. doi: 10.1021/acs.joc.5c00290 (PMC12038850; doi:10.1021/acs.joc.5c00290)
Supplement: Supplementary file 1 — jo5c00290_si_001.pdf [file jo5c00290_si_001.pdf]

Supporting Information

Synthesis of PSI Oligosaccharide for the Development of Total Synthetic Vaccine against Clostridium difficile

Hong-Jay Lo, *,† Ravinder Mettu,† Chiang-Yun Chen, Shiou-Ting Li, Chung-Yi Wu *

†These authors contributed equally.

Genomics Research Center, Academia Sinica, 128 Academia Road, Section 2, Nanakang, Taipei 115, Taiwan.

Table of Contents

Experimental Procedures and Characterization Data S2

Reference S11

^{1}H and ^{13}C NMR Spectra S12

2D NMR HSQC-Coupled spectra S154

Experimental Procedure and Characterization Data

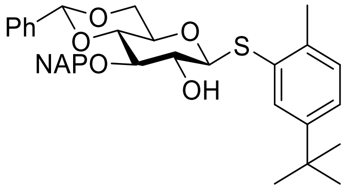

(2-Methyl-5-tert-butylphenyl) 4,6-*O*-benzylidene-3-*O*-(2-naphthylmethyl)-1-thio-β-D-glucopyranoside (61). A mixture of (2-Methyl-5-tert-butylphenyl) 4,6-*O*-benzylidene-3-*O*-1-thio-β-D-glucopyranoside[1] (8.50 g, 19.7 mmol, 1equiv.) and dibutyltin oxide (5.90 g, 23.7 mmol, 1.2 equiv) in toluene (150 mL) was stirred at reflux for 3 h using dean-stark apparatus to remove water with azeotropic, while the mixture became a clear solution. The solution was removed under reduced pressure and coevaporated twice with toluene. The obtained residue was dissolved in *N*,*N*-dimethylformamide (85 mL), 2-(bromomethyl)-naphthalene (6.55 g, 29.6 mmol, 1.5 equiv.) and cesium fluoride (3.60 g, 23.7 mmol, 1.2 equiv.). The reaction was stirred at room temperature for 16 h. The solvent was removed; then, the residue was diluted with EtOAc (200 mL), washed with saturated aq. $NaHCO_3$ (3 mL) and brine (2 mL), dried over $MgSO_4$, filtered, and concentrated *in vacuo*. The residue was purified by silica gel column chromatography using EtOAc : *n*-Hexane (1 : 10) as eluents to give compound **61** as a white powder (8.00 g, 71%) and recover starting material (2.00 g, b.r.s.m. 93%). R_f = 0.23 (silica gel, EtOAc : *n*-Hexane = 1 : 10); ^{1}H NMR (600 MHz, $CDCl_3$): δ 7.80-7.77 (m, 3H, Ar-H), 7.72-7.71 (m, 1H, Ar-H), 7.58 (d, *J* = 1.8 Hz, 1H, Ar-H), 7.48-7.47 (m, 3H, Ar-H), 7.44-7.43 (m, 2H, Ar-H), 7.38-7.36 (m, 3H, Ar-H), 7.22-7.21 (m, 1H, Ar-H), 7.12 (d, *J* = 7.8 Hz, 1H, Ar-H), 5.57 (s, 1H, Ph-CH), 5.09 (d, *J* = 12.0 Hz, 1H), 4.97 (d, *J* = 12.0 Hz, 1H), 4.61 (d, *J* = 7.2 Hz, 1H, C1-$H_β$), 4.35 (dd, *J* = 10.2, 4.8 Hz, 1H), 3.79 (dd, *J* = 10.8, 10.2 Hz, 1H), 3.75-3.68 (m, 2H), 3.61-3.58 (m, 1H), 3.50 (ddd, *J* = 10.8, 10.2, 5.4 Hz, 1H), 2.55 (d, *J* = 2.4 Hz, 1H, OH), 2.39 (s, 3H, $-CH_3$), 1.28 (s, 9H, *t*Bu-CH_3); 13C{1H} NMR (150 MHz, $CDCl_3$): δ 149.5, 137.2, 137.1, 135.6, 133.3, 133.1, 130.7, 130.4, 130.1, 129.1, 128.3, 128.3, 127.9, 127.7, 126.9, 126.1, 126.0, 125.9, 125.4, 101.4, 88.7, 81.5, 81.1, 74.8, 72.8, 70.6, 68.7, 34.5, 31.3, 20.5; HRMS (ESI-TOF) m/z: $[M+Na]^+$ calcd for $C_{35}H_{38}O_5SNa$ 593.2375 found 593.2332.

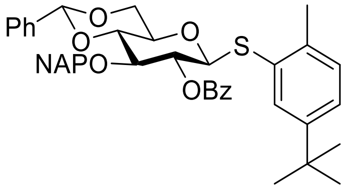

(2-Methyl-5-*tert*-butylphenyl) 2-*O*-benzoyl-4,6-*O*-benzylidene-3-*O*-(2-naphthylmethyl)-1-thio-β-D-glucopyranoside (62). To a stirred solution of starting material **61** (3.10 g, 5.43 mmol, 1 equiv.) in CH_2Cl_2 (20 mL), pyridine (2.2 mL, 27.2 mmol, 5 equiv.), 4-dimethylaminopyridine (66.3 mg, 0.54 mmol, 0.1 eq) and benzoyl chloride (1.26 mL, 10.9 mmol, 2 equiv.) were added. The mixture was stirred at 0 °C until TLC analysis indicated starting materials had disappeared (1 h). Upon completion, the reaction was quenched with methanol and concentrated. The residue was diluted with CH_2Cl_2 (50 mL), extracted with aq. 1M HCl (20 mL), and neutralized with satd. aq. $NaHCO_3$ (20 mL). The solvent was removed, and the crude product was purified by silica gel column chromatography using EtOAc : *n*-Hexane (1 : 4) as eluents to give compound **62** as white powder (3.20 g, 88%). R_f = 0.58 (silica gel, EtOAc : *n*-Hexane = 1 : 3); ^{1}H NMR (600 MHz, $CDCl_3$): δ 8.01-7.99 (m, 2H, Ar-H), 7.70 (d, *J* = 7.8 Hz, 1H, Ar-H), 7.63 (s, 1H, Ar-H), 7.61-7.55 (m, 5H, Ar-H), 7.48 (d, *J* = 8.4 Hz, 1H, Ar-H), 7.46-7.39 (m, 7H, Ar-H), 7.27 (dd, *J* = 8.4, 1.2 Hz, 1H, Ar-H), 7.22 (dd, *J* = 7.8, 2.4 Hz, 1H, Ar-H), 7.09 (d, *J* = 8.4 Hz, 1H, Ar-H), 5.88 (s, 1H, Ph-CH), 5.46 (dd, *J* = 10.2, 8.4 Hz, 1H), 5.02 (d, *J* = 12.6 Hz, 1H), 4.90 (d, *J* = 12.6 Hz, 1H), 4.83 (d, *J* = 10.2 Hz, 1H, C1-$H_β$), 4.43 (dd, *J* = 10.8, 5.4 Hz, 1H), 4.01-3.91 (m, 3H), 3.59 (ddd, *J* = 9.6, 9.0, 4.8 Hz, 1H), 2.22 (s, 3H, $-CH_3$), 1.30 (s, 9H, *t*Bu-CH_3); 13C{1H} NMR (150 MHz, $CDCl_3$): δ 165.0, 149.2, 137.1 136.8, 135.1, 133.1 133.0, 132.8, 132.1, 129.9, 129.8, 129.8, 129.6, 129.0, 128.3, 128.0, 127.8, 127.5, 126.9,

126.1, 126.0, 126.0, 125.8, 125.7, 125.2, 101.3, 87.8, 81.5, 79.0, 74.2, 72.0, 70.4, 68.6, 34.3, 31.2, 20.1; HRMS (ESI-TOF) m/z: $[M+Na]^+$ calcd for $C_{42}H_{42}O_6SNa$ 697.2594 found 697.2638.

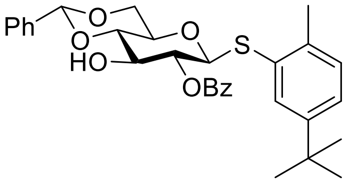

(2-Methyl-5-*tert*-butylphenyl) 2-*O*-benzoyl-4,6-*O*-benzylidene-1-thio-β-D-glucopyranoside (17).[1] To a stirred solution of starting material **62** (10.7 g, 15.9 mmol, 1equiv.) in a mixture of CH_2Cl_2/phosphate buffer (500 mL, 9 : 1 = *v/v*, pH7), 2,3-dichloro-5,6-dicyanobenzoquinone (6.30 g, 27.8 mmol, 1.75 equiv.) was added at 0 °C. The mixture was stirred at 0 °C until TLC analysis indicated all starting materials had disappeared (16 h). Upon completion, the mixture was filtered by a pad of Celite, extracted with satd. aq. $NaHCO_3$ (150 mL), concentrated and purified by silica gel column chromatography using EtOAc : *n*-Hexane (1 : 3) as eluents to give compound **17** as a white powder (7.71 g, 91%). R_f = 0.53 (silica gel, EtOAc : *n*-Hexane = 1 : 3).

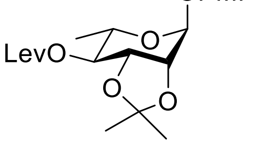

4-Methoxyphenyl 4-*O*-oxopentanoate-2,3-*O*-isopropylidene-α-L-rhamnopyranoside (63). To a stirred solution of 4-Methoxyphenyl 4-*O*-oxopentanoate-2,3-*O*-isopropylidene-α-L-rhamnopyranoside[2] (12.9 g, 41.6 mmol, 1 equiv.) in CH_2Cl_2 (200 mL), pyridine (8.40 mL, 0.10 mol, 2.5 equiv.), 4-dimethylaminopyridine (508 mg, 4.16 mmol, 0.1 equiv.), and levulinic anhydride (19.6 g, 83.1 mmol, 2.2 equiv.) were added at room temperature under argon atmosphere. The reaction mixture was vigorously stirred until TLC analysis indicated all starting materials had disappeared (40 min). Upon completion, the reaction mixture was quenched by satd. aq. $NaHCO_3$ (10 mL). The solvent was removed by rotary evaporation under high vacuum. The obtained residue was diluted with CH_2Cl_2 (200 mL) and washed with satd. aq. $NaHCO_3$ (100 mL) and water (50 mL). The separated organic layer was dried over $MgSO_4$ and concentrated. The obtained residue was purified by silica gel column chromatography using EtOAc : toluene (1 : 5) as eluents to give compound **63** as a white powder (16.2 g, 95%). R_f = 0.44 (silica gel, EtOAc : toluene 1 : 4); ^{1}H NMR (600 MHz, $CDCl_3$): δ 6.96 (d, *J* = 9.0 Hz, 2H, Ar-H), 6.80 (d, *J* = 9.0 Hz, 2H, Ar-H), 5.59 (s, 1H, C1-H_α), 4.88 (dd, *J* = 10.2, 7.8 Hz, 1H), 4.32-4.28 (m, 2H), 3.89-3.84 (m, 1H), 3.73 (s, 3H, O-CH_3), 2.86-2.80 (m, 1H, -CH_aH_b), 2.68-2.60 (m, 2H, -CH_2), 2.56-2.51 (m, 1H, -CH_aH_b), 2.14 (s, 3H, -CH_3), 1.55 (s, 3H, -CH_3), 1.35 (s, 3H, -CH_3), 1.11 (d, *J* = 6.6 Hz, 3H, -CH_3); 13C{1H} NMR (150 MHz, $CDCl_3$): δ 206.2, 171.9, 154.9, 150.0, 117.6, 114.6, 109.9, 96.0, 75.8, 75.6, 74.5, 64.8, 55.5, 37.8, 29.7, 27.9, 27.6, 26.4, 16.9; HRMS (ESI-TOF) m/z: $[M+Na]^+$ calcd for $C_{21}H_{28}O_8Na$ 431.1676 found 431.1674.

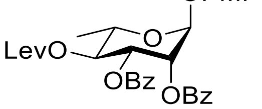

4-Methoxyphenyl 2,3-*O*-dibenzoyl-4-*O*-oxopentanoate-α-L-rhamnopyranoside (64). To a stirred solution of compound **63** (13.2 g, 32.3 mmol, 1 equiv.) in MeOH (150 mL), 4% aq. HCl was added until the pH was adjusted to 2. The reaction mixture was stirred at 40 °C until TLC analysis indicated all starting material had disappeared (20 h). The solvent was removed by rotary evaporation under high vacuum and coevaporated with toluene twice. The obtained residue was dissolved in CH_2Cl_2 (300 mL); then, pyridine (15.7 mL, 0.19 mol, 6 equiv.), 4-dimethylaminopyridine (395 mg, 3.23 mmol, 0.1 equiv.), and benzoyl chloride (11.3 mL, 97.0 mmol, 3 equiv.) were added at 0 °C under argon atmosphere. The reaction was stirred until TLC analysis indicated all starting material had disappeared (1 h). Upon completion, the reaction mixture quenched by MeOH (10 mL). The solvent was removed by rotary

evaporation under high vacuum. The obtained residue was diluted with CH_2Cl_2 (200 mL) and washed with satd. aq. $NaHCO_3$ (100 mL) and water (50 mL). The separated organic layer was dried over $MgSO_4$, concentrated, and the obtained residue was purified by silica gel column chromatography using EtOAc : *n*-Hexane (1 : 2) as eluents to give compound **64** as a white powder (17.3 g, 93%). R_f = 0.44 (silica gel, EtOAc : *n*-Hexane = 2 : 3); ^{1}H NMR (600 MHz, $CDCl_3$): δ 8.07-8.06 (m, 2H, Ar-H), 7.91-7.90 (m, 2H, Ar-H), 7.59-7.56 (m, 1H, Ar-H), 7.50-7.44 (m, 3H, Ar-H), 7.35-7.32 (m, 2H, Ar-H), 7.06 (d, *J* = 9.0 Hz, 2H, Ar-H), 6.83 (d, *J* = 9.0 Hz, 2H, Ar-H), 5.85 (dd, *J* = 10.2, 3.6 Hz, 1H), 5.78 (dd, *J* = 3.6, 1.8 Hz, 1H), 5.56 (d, *J* = 1.8 Hz, 1H, C1-H_α), 5.48 (dd, *J* = 10.2, 9.6 Hz, 1H), 4.22-4.17 (m, 1H), 3.75 (s, 3H, O-CH_3), 2.69-2.63 (m, 1H, -CH_aH_b), 2.59-2.51 (m, 2H, -CH_2), 2.42-2.37 (m, 1H, -CH_aH_b), 2.04 (s, 3H, -CH_3), 1.32 (d, *J* = 6.0 Hz, 3H, -CH_3); 13C{1H} NMR (150 MHz, $CDCl_3$): δ 205.8, 171.9, 165.4, 165.4, 155.2, 149.9, 133.4, 133.2, 129.8, 129.7, 129.2, 128.5, 128.3, 117.6, 114.6, 96.4, 71.3, 70.5, 69.8, 67.1, 55.5, 37.7, 29.4, 27.9, 17.4; HRMS (ESI-TOF) m/z: $[M+Na]^+$ calcd for $C_{32}H_{32}O_{10}Na$ 599.1888 found 599.1888.

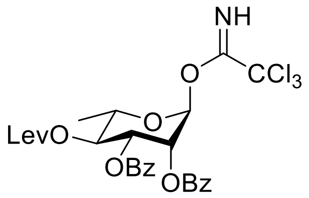

2,3-di-*O*-benzoyl-4-*O*-oxopentanoate-α-L-rhamnopyranosyl trichloroacetimidate (18). To a stirred solution of starting material **64** (18.5 g, 32.1 mmol, 1 equiv.) in a mixture of acetonitrile/water (600 mL, 1 : 1 = *v/v*), ceric ammonium nitrate (52.8 g, 96.3 mmol, 3 equiv.) was added at room temperature. The reaction mixture was vigorously stirred until TLC analysis indicated all starting material had disappeared (2 h). Upon completion, the solvent was removed by rotary evaporation under high vacuum. The obtained residue was diluted with CH_2Cl_2 (400 mL) and washed with water (200 mL). The separated organic layer was dried over $MgSO_4$ and concentrated. The obtained residue was purified by silica gel column chromatography using EtOAc : *n*-hexane (1 : 1) as eluents to give α, β mixture hemiacetals as a yellow powder (15.0 g) for further step.

To a well-stirred solution of the hemiacetal (15.0 g, 31.9 mmol, 1equiv.) obtained above, activated pulverized 4 Å molecular sieves (5 g) in anhydrous CH_2Cl_2 (300 mL), trichloroacetonitrile (32.0 mL, 0.319 mol, 10 equiv.), and DBU (0.95 mL, 6.38 mmol, 0.2 equiv.) were added at 0 °C under argon atmosphere. The reaction was stirred at room temperature for 2 h. Upon completion, the solution was filtered through a pad of Celite and concentrated. The obtained residue was purified by silica gel column chromatography using EtOAc : *n*-Hexane (1 : 5) as eluents to give compound **18** as a white powder (16.8 g, 85%)(over 2 steps). R_f = 0.53 (silica gel, EtOAc : *n*-Hexane = 1 : 2). R_f = 0.53 (silica gel, EtOAc : *n*-Hexane = 1 : 2); ^{1}H NMR (600 MHz, $CDCl_3$): δ 8.78 (s, 1H, NH), 8.03 (d, *J* = 7.8 Hz, 2H, Ar-H), 7.86 (d, *J* = 7.8 Hz, 2H, Ar-H), 7.59-7.57 (m, 1H, Ar-H), 7.49-7.44 (m, 3H, Ar-H), 7.33-7.31 (m, 2H, Ar-H), 6.41 (s, 1H, C1-H_α), 5.81 (dd, *J* = 1.8, 1.2 Hz, 1H), 5.70 (dd, *J* = 10.2, 3.6 Hz, 1H), 5.49 (dd, *J* = 10.2, 9.6 Hz, 1H), 4.27-4.22 (m, 1H), 2.70-2.64 (m, 1H, -CH_aH_b), 2.59-2.52 (m, 2H, -CH_2), 2.41-2.36 (m, 1H, -CH_aH_b), 2.04 (s, 3H, -CH_3), 1.37 (d, *J* = 6.0 Hz, 3H, -CH_3); 13C{1H} NMR (150 MHz, $CDCl_3$): δ 205.9, 171.9, 165.4, 165.2, 159.9, 133.6, 133.3, 129.9, 129.7, 129.0, 128.9, 128.6, 128.4, 94.6, 90.7, 70.6, 69.6, 69.4, 68.9, 37.7, 29.5, 27.8, 17.5; HRMS (ESI-TOF) m/z: $[M+Na]^+$ calcd for $C_{27}H_{26}NO_9Cl_3Na$ 636.0565 found 636.0566.

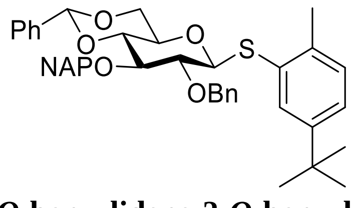

(2-Methyl-5-tert-butylphenyl) 4,6-*O*-benzylidene-2-*O*-benzyl-3-*O*-(2-naphthylmethyl)-1-thio-β-D-glucopyranoside (65). To a suspension of NaH (60%, 511 mg, 12.8 mmol, 2.4 equiv.) in dry DMF (20

mL), (2-Methyl-5-tert-butylphenyl) 4,6-*O*-benzylidene-3-*O*-(2-naphthylmethyl)-1-thio-β-D-glucopyranoside[1] (3.00 g, 5.26 mmol, 1equiv.) dissolved in dry DMF (10 mL) was added at 0 °C under argon atmosphere. The reaction mixture was stirred for 10 min.; then, benzyl bromide (1.96 mL, 15.9 mmol, 3 equiv.) was added dropwise. Stirring was continued for 4 h at room temperature. Upon completion, it was carefully quenched with MeOH at 0 °C and concentrated *in vacuo*. The obtained residue was diluted with CH_2Cl_2 (50 mL) and washed with H_2O. The combined organic layers were dried over $MgSO_4$, filtered, and concentrated *in vacuo*. The resulting residue was purified by silica gel column chromatography using EtOAc : CH_2Cl_2 : *n*-Hexane (1 : 1 : 20) as eluents to give compound **65** as a white solid (3.24 g, 93%). R_f = 0.49 (silica gel, EtOAc : *n*-Hexane = 1 : 10); ^{1}H NMR (600 MHz, $CDCl_3$): δ 7.82-7.80 (m, 1H, Ar-H), 7.78 (br, 1H, Ar-H), 7.75 (d, *J* = 8.4 Hz, 1H, Ar-H), 7.70-7.68 (m, 1H, Ar-H), 7.62 (d, *J* =1.8 Hz, 1H, Ar-H), 7.51-7.43 (m, 5H, Ar-H), 7.41-7.37 (m, 5H, Ar-H), 7.34-7.30 (m, 3H, Ar-H), 7.21 (dd, *J* = 6.6, 1.8 Hz, 1H, Ar-H), 7.13 (d, *J* = 7.8 Hz, 1H, Ar-H), 5.63 (s, 1H, Ph-CH), 5.11 (d, *J* = 11.4 Hz, 1H), 4.99-4.96 (m, 2H), 4.90 (d, *J* = 10.2 Hz, 1H), 4.78 (d, *J* = 9.6 Hz, 1H, C1-H_β), 4.36 (dd, *J* = 10.2, 4.8 Hz, 1H), 3.91 (dd, *J* = 9.6, 8.4 Hz, 1H), 3.85 (dd, *J* = 10.8, 10.2 Hz, 1H), 3.81 (dd, *J* = 9.6, 9.0 Hz, 1H), 3.63 (dd, *J* = 10.2, 8.4 Hz, 1H), 3.49 (ddd, *J* = 9.6, 9.6, 4.8 Hz, 1H), 2.40 (s, 3H, $-CH_3$), 1.30 (s, 9H, *t*Bu-CH_3); 13C{1H} NMR (150 MHz, $CDCl_3$): δ 149.5, 138.0, 137.2, 136.1, 135.8, 133.3, 133.0, 132.7, 129.9, 129.0, 128.9, 128.3, 128.3, 128.2, 128.1, 127.9, 127.8, 127.6, 126.8, 126.2, 126.0, 126.0, 125.8, 124.7, 101.2, 88.5, 82.9, 81.5, 80.9, 76.0, 75.3, 70.1, 68.7, 34.4, 31.3, 20.3; HRMS (ESI-TOF) m/z: $[M+Na]^+$ calcd for $C_{42}H_{45}O_5S$ 661.2982 found 661.3028.

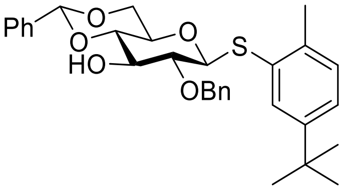

(2-Methyl-5-tert-butylphenyl) 4,6-*O*-benzylidene-2-*O*-benzyl-1-thio-β-D-glucopyranoside (20).[1] To a stirred solution of starting material **65** (1.00 g, 1.51 mmol, 1 equiv.) in a mixture of CH_2Cl_2/phosphate buffer (50 mL, 9 : 1 = *v/v*, pH7), 2,3-dichloro-5,6-dicyanobenzoquinone (601 mg, 2.65 mmol, 1.75 equiv.) was added at 0 °C. The reaction mixture was vigorously stirred until TLC analysis indicated disappearance of starting material (3 h). Upon completion, the reaction mixture was diluted with CH_2Cl_2 (50 mL) and washed with satd aq $NaHCO_3$ (40 mL) and brine (20 mL). The organic phase was washed with water until the solution became colorless. The separated organic layer was dried over $MgSO_4$, filtered, and concentrated. The obtained residue was purified by silica gel column chromatography using EtOAc : *n*-Hexane (1 : 4) as eluents to give compound **20** as a white powder (640 mg, 81%).

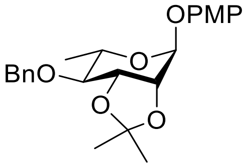

4-Methoxyphenyl 4-*O*-benzyl-2,3-*O*-isopropylidene-α-L-rhamnopyranoside (66).[2] To a suspension of NaH (60%, 2.06 g, 51.4 mmol, 2.1 equiv.) in dry DMF (50 mL), compound 4-Methoxyphenyl 2,3-*O*-Isopropylidene-α-L-rhamnopyranoside[2] (7.6 g, 24.5 mmol, 1equiv.) dissolved in dry DMF (30 mL) was added at 0 °C under argon atmosphere. The reaction mixture was stirred for 10 min.; then,, benzyl bromide (6.12 mL, 51.4 mmol, 2.1 equiv.) was added dropwise. Stirring was continued for 1 h at room temperature. Upon completion, it was carefully quenched with MeOH at 0 °C and concentrated *in vacuo*. The obtained residue was diluted with CH_2Cl_2 (200 mL) and washed with H_2O. The combined organic layers were dried over $MgSO_4$, filtered and concentrated *in vacuo*. The resulting residue was purified by silica gel column chromatography using EtOAc : *n*-Hexane (1 : 10) as eluents to give compound **66** as a white solid (9.55 g, 97%).

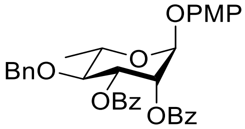

4-Methoxyphenyl 2,3-di-*O*-benzoyl-4-*O*-benzyl-α-L-rhamnopyranoside (67).[1] To a stirred solution of compound **66** (7.25 g, 18.1 mmol, 1 equiv.) in MeOH (85 mL), 4% aq. HCl was added until pH was adjusted to 2. The reaction mixture was stirred at 40 °C until TLC analysis indicated all starting material had disappeared (20 h). The solvent was removed by rotary evaporation under high vacuum and coevaporated with toluene twice. The obtained residue was dissolved by CH_2Cl_2 (200 mL); then, pyridine (8.75 mL, 0.11 mol, 6 equiv.), 4-dimethylaminopyridine (221 mg, 1.81 mmol, 0.1 equiv.), and benzoyl chloride (6.31 mL, 54.3 mmol, 3 equiv.) were added at 0 °C under argon atmosphere. The reaction mixture was stirred until TLC analysis indicated all starting material has disappeared (1 h). Upon completion, the reaction mixture was quenched with MeOH (10 mL), and the solvent was removed by rotary evaporation under high vacuum. The obtained residue was diluted with CH_2Cl_2 (200 mL) and washed with satd. aq. $NaHCO_3$ (100 mL) and water (50 mL). The separated organic layer was dried over $MgSO_4$ and concentrated. The obtained residue was purified by silica gel column chromatography using EtOAc : *n*-Hexane (1 : 4) as eluents to give compound **67** as a white powder (9.10 g, 88%).

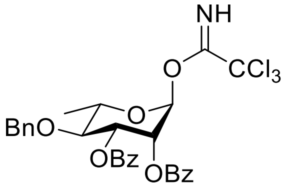

2,3-di-*O*-benzoyl-4-*O*-benzyl-α-L-rhamnopyranosyl trichloroacetimidate (13). To a stirred solution of starting material **67** (9.00 g, 15.8 mmol, 1 equiv.) in a mixture of acetonitrile/water (300 mL, 1 : 1 = *v/v*), ceric ammonium nitrate (26.0 g, 47.5 mmol, 3 equiv.) was added at room temperature. The reaction mixture was vigorously stirred until TLC analysis indicated all starting material has disappeared (2 h). Upon completion, the solvent was removed by rotary evaporation under high vacuum. The obtained residue was diluted with CH_2Cl_2 (300 mL) and washed with water (100 × 2 mL). The separated organic layer was dried over $MgSO_4$ and concentrated. The obtained residue was purified by silica gel column chromatography using EtOAc : *n*-Hexane (1 : 1) as eluents to give α, β mixture hemiacetals as a yellow powder (7.25 g) for the next step.

To a well-stirred solution of hemiacetal (7.25 g, 15.7 mmol, 1 equiv.) prepared above and activated pulverized 4 Å molecular sieves (4 g) in anhydrous CH_2Cl_2 (200 mL), trichloroacetonitrile (15.7 mL, 0.16 mol, 10 equiv.) and DBU (0.47 mL, 3.14 mmol, 0.2 equiv.) were added at 0 °C under argon atmosphere. The reaction mixture was stirred at room temperature for 2 h. Upon completion, the solution was filtered through a pad of Celite and concentrated. The obtained residue was purified by silica gel column chromatography using EtOAc : *n*-Hexane (1 : 5) as eluents to give compound **13** as a white powder (7.30 g, 76%)(2 steps). R_f = 0.44 (silica gel, EtOAc : *n*-Hexane = 1 : 3); ^{1}H NMR (600 MHz, $CDCl_3$): δ 8.73 (s, 1H, NH), 8.05-8.04 (m, 2H, Ar-H), 7.92-7.91 (m, 2H, Ar-H), 7.63 (t, *J* = 7.2 Hz, 1H, Ar-H), 7.53-7.48 (m, 3H, Ar-H), 7.37-7.34 (m, 2H, Ar-H), 7.23-7.19 (m, 5H, Ar-H), 6.39 (d, *J* = 1.8 Hz, 1H, C1-$H_α$), 5.83 (dd, *J* = 3.6, 1.8 Hz, 1H), 5.80 (dd, *J* = 9.6, 3.6 Hz, 1H), 4.75 (d, *J* = 10.8 Hz, 1H), 4.67 (d, *J* = 10.8 Hz, 1H), 4.23-4.18 (m, 1H), 3.88 (dd, *J* = 9.6, 9.6 Hz, 1H), 1.46 (d, *J* = 6.6 Hz, 3H, -CH_3); 13C{1H} NMR (150 MHz, $CDCl_3$): δ 165.3, 165.1, 160.2, 137.3, 133.5, 133.2, 129.8, 129.6, 129.5, 129.3, 128.6, 128.4, 128.4, 128.1, 128.0, 94.9, 90.8, 78.3, 75.3, 72.0, 70.7, 69.5, 18.2; HRMS (MADIL-TOF) m/z: $[M+Na]^+$ calcd for $C_{29}H_{26}NO_7Cl_3Na$ 628.0667 found 628.0709.

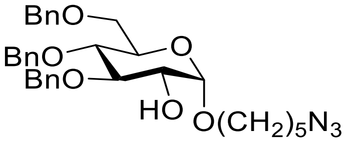

5-Azidopentyl 3,4,6-*tri-O*-benzyl-β-D-glucopyranoside (14). A solution of activated pulverized 4 Å molecular sieves (5.5 g) in anhydrous CH_2Cl_2/DMF (75 mL, 2 : 1 = *v/v*) was stirred under argon atmosphere. The solution was cooled to 0 °C; then, $CuBr_2$ (6.61 g, 29.6 mmol, 3.2 equiv.) and *n*-Bu_4NBr (6.26 g, 19.4 mmol, 2.1 equiv.) were added. To this mixture, 5-azidopenan-1-ol (1.79 g, 13.9 mmol, 1.5 equiv.) and starting material Ethyl 3,4,6-tri-*O*-benzyl-2-O-(2-naphthalenylmethyl)-1-thio-D-glucopyranoside[1] (5.87 g, 9.25 mmol, 1 equiv.) dissolved in CH_2Cl_2 (50 mL) were added dropwise at 0 °C. The reaction was stirred at room temperature until TLC analysis indicated starting materials had disappeared (16 h). Upon completion, it was quenched by saturated aq. $NaHCO_3$ (0.5 mL), diluted with EtOAc (200 mL), and filtered through a pad of Celite. The filtrate was washed with saturated aq. $NaHCO_3$ (100 mL) and brine (50 mL). The separated organic layer was dried over $MgSO_4$ and concentrated. The obtained residue was purified by silica gel column chromatography using EtOAc : *n*-Hexane (1 : 4) as eluents to give inseparable (α : β = 4 : 1) 5-Azidopentyl 3,4,6-*tri-O*-benzyl-(2-naphthalenylmethyl)-β-D-glucopyranoside as a white powder (4.80 g) for the next step.
To a stirred solution of 5-Azidopentyl 3,4,6-*tri-O*-benzyl-(2-naphthalenylmethyl)-β-D-glucopyranoside (4.80 g, 6.84 mmol, 1 equiv.) in CH_2Cl_2/phosphate buffer (200 mL, 9 : 1 = *v/v*, pH7), 2,3-dichloro-5,6-dicyanobenzoquinone (2.72 g, 12.0 mmol, 1.75 equiv.) was added at 0 °C. The reaction mixture was vigorously stirred until TLC analysis indicated all starting material had disappeared (3 h). Upon completion, the reaction mixture was diluted with CH_2Cl_2 (100 mL) and washed with saturated aq. $NaHCO_3$ (100 mL) and brine (50 mL). The organic phase was washed with water until the solution became colorless The separated organic layer was dried over $MgSO_4$, filtered, and concentrated. The obtained residue was purified by silica gel column chromatography using EtOAc : *n*-Hexane (1 : 3) as eluents to give compound **14** as a white powder (α isomer 2.49 g, 48% and β isomer 644 mg, 12%) (2 steps). **14 alpha** R_f = 0.22 (silica gel, EtOAc : *n*-Hexane = 1 : 3); ^{1}H NMR (600 MHz, $CDCl_3$): δ 7.38 (d, *J* = 7.2 Hz, 2H, Ar-H), 7.35-7.26 (m, 11H, Ar-H), 7.16-7.14 (m, 2H, Ar-H), 4.94 (d, *J* = 10.8 Hz, 1H), 4.89 (d, *J* = 3.6 Hz, 1H, C1-$H_α$), 4.85 (d, *J* = 10.8 Hz, 1H), 4.82 (d, *J* = 10.8 Hz, 1H), 4.63 (d, *J* = 12.0 Hz, 1H), 4.53-4.49 (m, 2H), 3.79-3.70 (m, 5H), 3.67 (dd, *J* = 10.8, 1.8 Hz, 1H), 3.62 (dd, *J* = 9.6, 8.4 Hz, 1H), 3.49-3.45 (m, 1H), 3.26 (t, *J* = 7.2 Hz, 2H, -$CH_{2linker}$), 2.00 (br, 1H, OH), 1.67-1.58 (m, 4H, 2-$CH_{2linker}$), 1.48-1.40 (m, 2H, -$CH_{2linker}$); 13C{1H} NMR (150 MHz, $CDCl_3$): δ 138.7, 138.1, 137.9, 128.3, 128.3, 127.9, 127.8, 127.7, 127.7, 127.6, 98.4, 83.4, 77.4, 75.3, 75.0, 73.5, 73.0, 70.6, 68.5, 67.8, 51.2, 28.9, 28.5, 23.3; HRMS (ESI-TOF) m/z: $[M+Na]^+$ calcd for $C_{32}H_{39}N_3O_6Na$ 584.2731 found 584.2733.
14 beta R_f = 0.31 (silica gel, EtOAc : *n*-hexane = 1 : 3); ^{1}H NMR (600 MHz, $CDCl_3$): δ 7.38 (d, *J* = 7.2 Hz, 2H, Ar-H), 7.35-7.27 (m, 11H, Ar-H), 7.19-7.17 (m, 2H, Ar-H), 4.93 (d, *J* = 10.8 Hz, 1H), 4.86-4.83 (m, 2H), 4.61 (d, *J* = 12.6 Hz, 1H), 4.56-4.53 (m, 2H), 4.25 (d, *J* = 7.8 Hz, 1H, C1-$H_β$), 3.95-3.91 (m, 1H), 3.74 (dd, *J* = 10.8, 1.8 Hz, 1H), 3.69 (dd, *J* = 10.8, 4.8 Hz, 1H), 3.62-3.57 (m, 2H), 3.55-3.52 (m, 2H), 3.50-3.47 (m, 1H), 3.26 (t, *J* = 7.2 Hz, 2H, -$CH_{2linker}$), 2.24 (br, 1H, OH), 1.69-1.60 (m, 4H, 2-$CH_{2linker}$), 1.47-1.43 (m, 2H, -$CH_{2linker}$); 13C{1H} NMR (150 MHz, $CDCl_3$): δ 138.6, 138.1, 138.0, 128.4, 128.3, 128.3, 127.9, 127.8, 127.7, 127.7, 127.6, 102.7, 84.5, 77.5, 75.1, 75.0, 74.9, 74.6, 73.4, 69.5, 68.9, 51.2, 29.0, 28.5, 23.2; HRMS (ESI-TOF) m/z: $[M+Na]^+$ calcd for $C_{32}H_{39}N_3O_6Na$ 584.2731 found 584.2727.

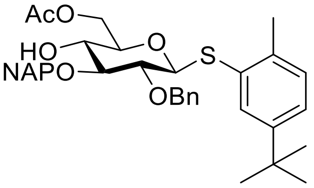

(2-Methyl-5-tert-butylphenyl) 6-*O*-Acetyl-2-*O*-benzyl-3-*O*-(2-naphthylmethyl)-1-thio-β-D-glucopyranoside (15). To a stirred solution of starting material **65** (5.60 g, 8.47 mmol, 1 equiv.) in a CH_2Cl_2/MeOH (180 mL, 2 : 1 = *v/v*), 4% aq. HCl was added until the pH was adjusted to 2. The reaction mixture was stirred at 40 °C until TLC analysis indicated all starting material had disapearred (20 h). The solvent was removed by rotary evaporation under high vacuum and coevaporated with toluene twice. The obtained residue was dissolved in CH_2Cl_2 (150 mL) and cooled to 0 °C; then, Ac_2O (0.80 mL, 8.47 mmol,

1 equiv.) and Et_3N (10.6 mL, 76.3 mmol, 9 equiv.) were added. Stirring was continied until TLC analysis indicated all starting material had disappeared (1 h). Upon completion, the reaction mixture was quenched by MeOH (10 mL). Stirring was continued for 10 min. The solvent was removed by rotary evaporation under high vacuum. The residue was purified by silica gel column chromatography using EtOAc : *n*-Hexane (1 : 3) as eluents to give compound **15** as a white powder (4.58 g, 88%). R_f = 0.29 (silica gel, EtOAc : *n*-Hexane = 1 : 3); ^1H NMR (600 MHz, $CDCl_3$): δ 7.81-7.80 (m, 2H, Ar-H), 7.78-7.76 (m, 1H, Ar-H), 7.73 (s, 1H, Ar-H), 7.59 (d, *J* = 1.8 Hz, 1H, Ar-H), 7.47-7.43 (m, 3H, Ar-H), 7.40-7.39 (m, 2H, Ar-H), 7.33-7.28 (m, 3H, Ar-H), 7.19 (dd, *J* = 7.8, 2.4 Hz, 1H, Ar-H), 7.11 (d, *J* = 8.4 Hz, 1H, Ar-H), 5.07 (d, *J* = 12.0 Hz, 1H), 5.00 (d, *J* = 10.2 Hz, 1H), 4.92 (d, *J* = 12.0 Hz, 1H), 4.80 (d, *J* = 10.2 Hz, 1H), 4.65 (d, *J* = 9.0 Hz, 1H, C1-H_β), 4.39 (dd, *J* = 12.6, 4.8 Hz, 1H), 4.27 (dd, *J* = 12.0, 1.8 Hz, 1H), 3.59-3.53 (m, 3H), 3.45-3.42 (m, 1H), 2.58 (d, *J* = 2.4 Hz, 1H, OH), 2.38 (s, 3H, $-CH_3$), 2.06 (s, 3H, $-CH_3$), 1.28 (s, 9H, *t*Bu-CH_3); 13C{1H} NMR (150 MHz, $CDCl_3$): δ 171.6, 149.5, 137.9, 136.2, 135.6, 133.3, 133.1, 133.1, 129.9, 128.7, 128.5, 128.4, 128.3, 128.2, 127.9, 127.9, 127.7, 126.9, 126.2, 126.1, 125.8, 124.6, 88.3, 85.7, 80.8, 77.5, 75.6, 75.5, 69.9, 63.6, 34.5, 31.3, 31.2, 20.9, 20.3; HRMS (ESI-TOF) m/z: $[M+Na]^+$ calcd for $C_{37}H_{42}O_6SNa$ 637.2594 found 637.2646.

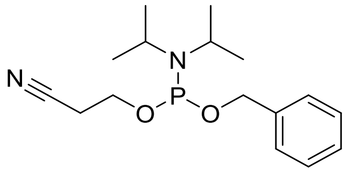

Benzyl 2-cyanoethyl *N,N*-diisopropylphosphoramidite (68).[3] To a stirred solution of 2-cyanoethyl *N,N,N′,N′*-tetraisopropylphosphorodiamidite (0.53 mL, 1.66 mmol, 1.1 equiv.) in anhydrous CH_2Cl_2 (10 mL), diisopropylammonium tetrazolide (129 mg, 0.75 mmol, 0.5 equiv.), activated pulverized 4 Å molecular sieves (200 mg), and benzyl alcohol (0.16 mL, 1.51 mmol, 1 equiv.) were added at room temperature. Stirring was continued until TLC analysis indicated all starting materials had disappeared (2 h). Upon completion, it was filtered through a pad of Celite. The solvent was removed, and the obtained residue was purified by cold silica gel column chromatography using EtOAc : *n*-Hexane : Et_3N (50 : 250 : 1) as eluents to give compound **68** as colorless oil (460 mg, 90%).

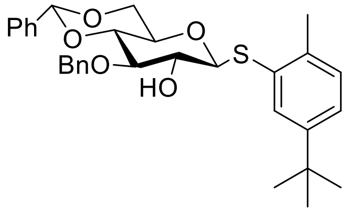

(2-Methyl-5-*tert*-butylphenyl) 4,6-*O*-benzylidene-3-*O*-benzyl-1-thio-β-D-glucopyranoside (69). A mixture of (2-Methyl-5-*tert*-butylphenyl) 4,6-*O*-benzylidene-1-thio-β-D-glucopyranoside[1] (5.00 g, 11.6 mmol, 1equiv.) and dibutyltin oxide (3.76 g, 15.1 mmol, 1.3 equiv) in toluene (125 mL) was stirred at reflux for 3h. Azeotropic removal of water was performed using dean-stark apparatus, during which the mixture became a clear solution. The liquid was removed under reduced pressure and coevaporated twice with toluene. The obtained residue was dissolved in *N,N*-dimethylformamide (70 mL); then, benzyl bromide (1.73 mL, 14.5 mmol, 1.25 equiv.) and cesium fluoride (2.20 g, 14.5 mmol, 1.25 equiv.) were added. The reaction was stirred at room temperature for 16 h. The solvent was removed, and the residue was diluted with EtOAc (200 mL), washed with saturated aq. $NaHCO_3$ (60 mL) and brine (30 mL), dried over $MgSO_4$, filtered, and concentrated *in vacuo*. The residue was purified by silica gel column chromatography using EtOAc : *n*-Hexane (1 : 6) as eluents to give compound **69** as a white powder (4.26 g, 70%) and recovered starting material (200 mg, b.r.s.m. 73%). R_f = 0.54 (silica gel, EtOAc : *n*-Hexane = 1 : 3); ^1H NMR (600 MHz, $CDCl_3$): δ 7.60 (d, *J* = 2.4 Hz, 1H, Ar-H), 7.49-7.47 (m, 2H, Ar-H), 7.38-7.36 (m, 5H, Ar-H), 7.33-7.30 (m, 2H, Ar-H), 7.28 (d, *J* = 7.2 Hz, 1H, Ar-H), 7.23 (dd, *J* = 7.8, 2.4 Hz, 1H, Ar-H), 7.13 (d, *J* = 7.8 Hz, 1H, Ar-H), 5.56 (s, 1H, Ph-CH), 4.95 (d, *J* = 12.0 Hz, 1H), 4.80 (d, *J* = 12.0 Hz, 1H), 4.63 (d, *J* = 9.6 Hz, 1H, C1-H_β), 4.36 (dd, *J* = 10.2, 5.4 Hz, 1H), 3.79 (dd, *J* = 10.2, 10.2 Hz, 1H), 3.71-3.66 (m, 2H), 3.58-3.55 (m, 1H), 3.52-3.48 (m, 1H), 2.57 (d, *J* = 1.8 Hz, 1H, OH), 2.41 (s,

3H, $-CH_3$), 1.30 (s, 9H, *t*Bu-CH_3); 13C{1H} NMR (150 MHz, $CDCl_3$): δ 149.5, 138.2, 137.1, 137.1, 130.7, 130.4, 130.0, 129.0, 128.4, 128.2, 128.1, 127.9, 126.0, 125.3, 101.3, 88.6, 81.7, 81.1, 74.8, 72.6, 70.6, 68.7, 34.4, 31.3, 20.4; HRMS (ESI-TOF) m/z: $[M+Na]^+$ calcd for $C_{31}H_{36}O_5SNa$ 543.2176 found 543.2212.

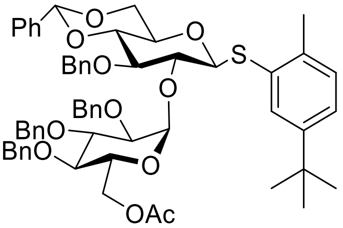

(2-Methyl-5-*tert*-butylphenyl) 6-*O*-acetyl-2,3,4-tri-*O*-benzyl-α-D-glucopyranosyl-(1→2)-4,6-*O*-benzylidene-3-*O*-benzyl-1-thio-β-D-glucopyranoside (7). A mixture of acceptor **69** (651 mg, 1.25 mmol, 1 equiv.), donor **6-*O*-acetyl-2,3,4-tri-*O*-benzyl-α-D-glucopyranosyl *N*-phenyltrifluoroacetimidate**[4] (1.08 g, 1.63 mmol, 1.3 equiv.), and activated pulverized 4 Å molecular sieves (1 g) in anhydrous CH_2Cl_2/ether (28 mL, 1 : 3 = *v/v*) were stirred under argon atmosphere for 1 h. The mixture was cooled to −30 °C; then, TMSOTf (68 μL, 0.38 mmol, 0.3 equiv. with respect to acceptor) was added. Stirring was continued until TLC analysis indicated all starting materials had disappeared (1 h). Upon completion, the reaction was quenched by Et_3N (0.2 mL) and filtered through a pad of Celite. The solvent was removed, and the obtained residue was purified by silica gel column chromatography using EtOAc : *n*-Hexane (1 : 5) as eluents to give compound **7** as a white powder (1.08 g, 87%). R_f = 0.49 (silica gel, EtOAc : *n*-Hexane = 1 : 5); ^{1}H NMR (600 MHz, $CDCl_3$): δ 7.49-7.48 (m, 2H, Ar-H), 7.46 (d, *J* = 1.8 Hz, 1H, Ar-H), 7.38-7.26 (m, 15H, Ar-H), 7.20-7.18 (m, 2H, Ar-H), 7.17-7.14 (m, 1H, Ar-H), 7.12-7.09 (m, 2H, Ar-H), 7.07-7.03 (m, 3H, Ar-H), 7.00-6.99 (m, 2H, Ar-H), 5.98 (d, *J* = 3.6 Hz, 1H, C1-H_α), 5.64 (s, 1H, Ph-CH), 5.18 (d, *J* = 9.6 Hz, 1H, C1-H_β), 5.01-4.97 (m, 2H), 4.88 (d, *J* = 10.8 Hz, 1H), 4.82 (d, *J* = 10.8 Hz, 1H), 4.74 (d, *J* = 10.8 Hz, 1H) 4.69 (d, *J* = 10.2 Hz, 1H), 4.59 (d, *J* = 11.4 Hz, 1H), 4.51 (d, *J* = 11.4 Hz, 1H), 4.38-4.35 (m, 2H), 4.06-3.95 (m, 3H), 3.88-3.80 (m, 4H), 3.65-3.61 (m, 2H), 3.52 (dd, *J* = 9.6, 9.0 Hz, 1H), 2.27 (s, 3H, $-CH_3$), 1.96 (s, 3H, $-CH_3$), 1.32 (s, 9H, *t*Bu-CH_3); 13C{1H} NMR (150 MHz, $CDCl_3$): δ 170.5, 149.8, 138.6, 138.2, 137.3, 137.3, 137.0, 133.6, 132.7, 130.1, 129.0, 128.9, 128.4, 128.3, 128.2, 128.1, 127.9, 127.8, 127.7, 127.6, 125.9, 124.1, 123.7, 101.2, 95.7, 86.0, 82.0, 81.7, 80.8, 80.1, 77.1, 75.7, 75.3, 75.1, 74.9, 73.3, 69.6, 68.8, 68.7, 62.4, 34.6, 31.3, 20.8, 19.8; HRMS (ESI-TOF) m/z: $[M+Na]^+$ calcd for $C_{60}H_{66}O_{11}SNa$ 1017.4218 found 1017.4252.

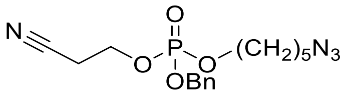

5-Azidopentyl benzyl (2-cyanoethyl) phosphate (37) To a stirred solution of benzyl 2-cyanoethyl *N,N*-diisopropylphosphoramidite (650 mg, 2.11 mmol, 1 equiv.) in CH_2Cl_2 (40 mL), 5-azidopentan-1-ol (545 mg, 4.22 mmol, 2 equiv.) and 1*H*-tetrazole (0.45 M in acetonitrile, 9.37 mL, 4.22 mmol, 2 equiv.) were added at room temperature. Stirring was continied until TLC analysis indicated all starting materials had disappeared (40 min.). Upon completion, the reaction was cooled to −20 °C; then, *m*-CPBA (614 mg, 2.74 mmol, 1.3 equiv.) was added. Stirring was contined until TLC analysis indicated all starting materials had disappeared (20 min). Upon completion, it was quenched by saturated aq. $NaHCO_3$ (15 mL) and extracted by CH_2Cl_2 (20 mL × 2). The separated organic layer was dried over $MgSO_4$ and concentrated, and the obtained residue was purified by silica gel column chromatography using EtOAc : *n*-Hexane (1:1) as eluents to give compound **37** (557 mg, 78%) as a colorless oil. R_f = 0.28 (silica gel, EtOAc : *n*-Hexane = 1:1); ^{1}H NMR (600 MHz, $CDCl_3$): δ 7.39-7.32 (m, 5H, Ar-H), 5.07 (d, *J* = 9.0 Hz, 2H, $-CH_2$), 4.17-4.07 (m, 2H, $-CH_2$), 4.05-4.01 (m, 2H, $-CH_{2linker}$), 3.23 (t, *J* = 6.6 Hz, 2H, $-CH_{2linker}$), 2.68-2.59 (m, 2H, $-CH_2$), 1.67-1.63 (m, 2H, $-CH_{2linker}$), 1.59-1.54 (m, 2H, $-CH_{2linker}$), 1.43-1.38 (m, 2H, $-CH_{2linker}$); 13C{1H} NMR (150 MHz, $CDCl_3$): δ 135.5 (d, J_{cp} = 5.0 Hz, O-Ar), 128.8, 128.6 (2C), 128.1(2C), 116.3, 69.7 (d,

J_{cp} = 5.0 Hz, -CH_2Ph), 68.0 (d, J_{cp} = 5.0 Hz, -$CH_{2linker}$), 61.6 (d, J_{cp} = 5.0 Hz, -CH_2), 51.1, 29.6 (d, J_{cp} = 6.3 Hz, -$CH_{2linker}$), 28.3, 22.6, 19.5 (d, J_{cp} = 6.3 Hz, -CH_2); HRMS (ESI-TOF) m/z: $[M+Na]^+$ calcd for $C_{15}H_{21}N_4O_4PNa$ 375.1193 found 375.1215.

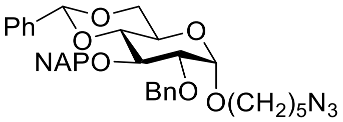

5-azidopentyl 4,6-O-benzylidene-2-*O*-benzyl-3-*O*-(2-naphthylmethyl)-1-α-D-glucopyranoside (70a) A mixture of 5-azidopenan-1-ol (78 mg, 0.60 mmol, 2 equiv.), donor **65** (200 mg, 0.30 mmol, 1 equiv.), and activated pulverized 4 Å molecular sieves (50 mg) in anhydrous CH_2Cl_2 (2 mL) was stirred under argon atmosphere for 1 h. It was cooled to 0 °C, and NIS (156 mg, 0.60 mmol, 2 equiv.) and TfOH (0.5 M in Et_2O, 0.18 mL, 0.09 mmol, 0.3 equiv.) were added; then, the reaction was warmed to room temperature. Stirring was continued until TLC analysis indicated all starting materials had disappeared (16 h). Upon completion, it was quenched by Et_3N (0.2 mL) and filtered through a pad of Celite. The filtrate was then quenched with 20% aq. $Na_2S_2O_3$ (5 mL) and washed with saturated aq. $NaHCO_3$ (3 mL) and brine (2 mL). The separated organic layer was dried over $MgSO_4$ and concentrated, and the obtained residue was purified by silica gel column chromatography using EtOAc : *n*-Hexane (1 : 6) as eluents to give (α : β = 2.7 : 1) compound **70** as a white powder (α isomer 121 mg, 62% and β isomer 44.8 mg, 23.1%). R_f = 0.38 (silica gel, EtOAc : *n*-Hexane = 1 : 6); ^{1}H NMR (600 MHz, $CDCl_3$): δ 7.83 (s, 1H, Ar-H), 7.81 (d, *J* = 7.8 Hz, 1H, Ar-H), 7.77 (d, *J* = 8.4Hz, 1H, Ar-H), 7.69 (d, *J* = 7.8 Hz, 1H, Ar-H), 7.52-7.51 (m, 3H, Ar-H), 7.46-7.42 (m, 2H, Ar-H), 7.38-7.37 (m, 5H, Ar-H), 7.35-7.29 (m, 3H, Ar-H), 5.59 (s, 1H, Ph-CH), 5.09 (d, *J* = 11.4 Hz, 1H), 5.02 (d, *J* = 11.4 Hz, 1H), 4.87 (d, *J* = 12.6 Hz, 1H), 4.76 (d, *J* = 3.0 Hz, 1H, C1-H_α), 4.71 (d, *J* = 12.6 Hz, 1H), 4.27 (dd, *J* = 10.2, 4.2 Hz, 1H), 4.11 (dd, *J* = 9.6, 9.0 Hz, 1H), 3.89-3.85 (m, 1H), 3.74-3.64 (m, 3H), 3.61 (dd, *J* = 9.0, 3.0 Hz, 1H), 3.46-3.42 (m, 1H), 3.25 (t, *J* = 7.2 Hz, 2H, -$CH_{2linker}$), 1.69-1.60 (m, 4H, 2-$CH_{2linker}$), 1.50-1.44 (m, 2H, -$CH_{2linker}$); 13C{1H} NMR (150 MHz, $CDCl_3$): δ 138.3, 137.4, 136.3, 133.3, 132.9, 128.9, 128.4, 128.2, 127.9, 127.9, 127.8, 127.6, 126.5, 126.1, 126.0, 125.8, 125.7, 101.3, 98.0, 82.2, 79.5, 78.5, 75.2, 73.5, 69.1, 68.1, 62.5, 51.2, 28.9, 28.6, 23.4; HRMS (ESI-TOF) m/z: $[M+Na]^+$ calcd for $C_{36}H_{39}N_3O_6Na$ 632.2731 found 632.2776.

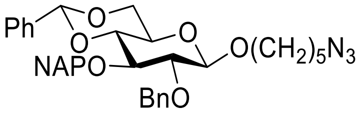

5-azidopentyl 4,6-O-benzylidene-2-*O*-benzyl-3-*O*-(2-naphthylmethyl)-1-β-D-glucopyranoside (70b) R_f = 0.31 (silica gel, EtOAc : *n*-Hexane = 1 : 6); ^{1}H NMR (600 MHz, $CDCl_3$): δ 7.79-7.78 (m, 1H, Ar-H), 7.76 (s, 1H, Ar-H), 7.72 (d, *J* = 8.4 Hz, 1H, Ar-H), 7.66-7.64 (m, 1H, Ar-H), 7.49-7.48 (m, 2H, Ar-H), 7.45-7.40 (m, 3H, Ar-H), 7.37-7.28 (m, 8H, Ar-H), 5.58 (s, 1H, Ph-CH), 5.05 (d, *J* = 11.4 Hz, 1H), 4.95 (d, *J* = 11.4 Hz, 1H), 4.90 (d, *J* = 10.8 Hz, 1H), 4.81 (d, *J* = 10.8 Hz, 1H), 4.49 (d, *J* = 7.2 Hz, 1H, C1-H_β), 4.34 (dd, *J* = 10.2, 4.8 Hz, 1H), 3.92 (dt, *J* = 9.6, 6.6 Hz, 1H), 3.81-3.77 (m, 2H), 3.71 (d, *J* = 9.6, 9.0 Hz, 1H), 3.56 (dt, *J* = 9.6, 6.6 Hz, 1H), 3.48 (dd, *J* = 8.4, 7.8 Hz, 1H), 3.40 (ddd, *J* = 8.4, 8.4, 4.8 Hz, 1H) 3.20 (t, *J* = 7.2 Hz, 2H, -$CH_{2linker}$), 1.69-1.63 (m, 2H, -$CH_{2linker}$), 1.62-1.57 (m, 2H, -$CH_{2linker}$), 1.52-1.41 (m, 2H, -$CH_{2linker}$) ; 13C{1H} NMR (150 MHz, $CDCl_3$): δ 138.4, 137.3, 136.0, 133.3, 133.0, 129.0, 128.3, 128.3, 128.0, 127.9, 127.9, 127.7, 127.6, 126.7, 126.2, 126.0, 125.9, 125.7, 104.1, 101.2, 82.2, 81.5, 80.7, 75.3, 75.1, 70.1, 68.8, 66.0, 51.3, 29.3, 28.6, 23.3; HRMS (ESI-TOF) m/z: $[M+Na]^+$ calcd for $C_{36}H_{39}N_3O_6Na$ 632.2731 found 632.2786.

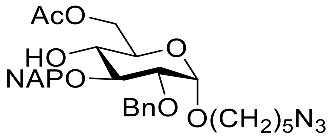

5-azidopentyl 6-O-acetyl-2-*O*-benzyl-3-*O*-(2-naphthylmethyl)-1-α-D-glucopyranoside (42). To a stirred solution of starting material **70a** (800 mg, 1.31 mmol, 1 equiv.) in CH_2Cl_2/MeOH (10 mL, 1 : 1 = *v/v*), 4% aq. HCl was added until the pH was adjusted to 2. The reaction mixture was stirred at 40 °C until

TLC analysis indicated all starting material had disappeared (20 h). The solvent was removed by rotary evaporation under high vacuum and coevaporated with toluene twice. The obtained residue was dissolved in CH_2Cl_2 (10 mL) at 0 °C; then, Ac_2O (0.12 mL, 1.31 mmol, 1 equiv.) and Et_3N (1.65 mL, 11.8 mmol, 9 equiv.) were added. Stirring was continued until TLC analysis indicated all starting material had disappeared (1 h). Upon completion, the reaction mixture was quenched by MeOH (1 mL), and stirring was continued for 10 minutes. Then, the solvent was removed by rotary evaporation under high vacuum. The residue was purified by silica gel column chromatography using EtOAc : *n*-Hexane (1 : 2) as eluents to give compound **42** as a colorless oil (660 mg, 89%). R_f = 0.50 (silica gel, EtOAc : *n*-Hexane = 2 : 3); ^{1}H NMR (600 MHz, $CDCl_3$): δ 7.83-7.79 (m, 4H, Ar-H), 7.48-7.44 (m, 3H, Ar-H), 7.37-7.35 (m, 2H, Ar-H), 7.33-7.27 (m, 3H, Ar-H), 5.14 (d, *J* = 11.4 Hz, 1H), 4.92 (d, *J* = 11.4 Hz, 1H), 4.77 (d, *J* = 12.0 Hz, 1H), 4.76 (d, *J* = 3.6 Hz,1H, C1-H_α), 4.65 (d, *J* = 12.0 Hz, 1H), 4.41 (dd, *J* = 12.0, 4.8 Hz, 1H), 4.20 (dd, *J* = 12.0, 1.8 Hz, 1H), 3.85 (dd, *J* = 9.0, 9.0 Hz, 1H), 3.76 (ddd, *J* = 10.2, 4.8, 2.4 Hz, 1H), 3.64 (dt, *J* = 10.2, 6.6 Hz, 1H), 3.53 (dd, *J* = 9.0, 3.6 Hz, 1H), 3.46 (td, *J* = 9.6, 3.0Hz, 1H), 3.41 (dt, *J* = 10.2, 6.6 Hz, 1H), 3.24 (t, *J* = 7.2 Hz, 2H, -$CH_{2linker}$), 2.60 (d, *J* = 3.0 Hz, 1H, OH), 2.07 (s, 3H, -CH_3), 1.70-1.59 (m, 4H, 2-$CH_{2linker}$), 1.48-1.43 (m, 2H, -$CH_{2linker}$); 13C{1H} NMR (150 MHz, $CDCl_3$): δ 171.3, 138.1, 136.1, 133.3, 133.0, 128.4, 128.3, 127.9, 127.9, 127.7, 126.6, 126.1, 125.9, 125.8, 97.0, 81.0, 79.7, 75.5, 72.9, 70.0, 69.4, 68.0, 63.2, 51.2, 28.9, 28.6, 23.4, 20.8; HRMS (ESI-TOF) m/z: $[M+Na]^+$ calcd for $C_{31}H_{37}N_3O_7Na$ 586.2524 found 586.2557.

References

(1) Martin, C. E.; Weishaupt, M. W.; Seeberger, P. H. Progress toward developing a carbohydrate-conjugate vaccine against *Clostridium difficile* ribotype 027: synthesis of the cell-surface polysaccharide PS-I repeating unit. *Chem. Commun.* **2011**, *47*, 10260-10262.

(2) Werz, D. B.; Seeberger, P. H. Total Synthesis of Antigen *Bacillus Anthracis* Tetrasaccharide—Creation of an Anthrax Vaccine Candidate. *Angew. Chem. Int. Ed.* **2005**, *44*, 6315-6318.

(3) Crich, D.; Dudkin, V. Confirmation of the Connectivity of 4,8,12,16,20-Pentamethylpentacosylphoshoryl *β*-D-Mannopyranoside, an Unusual *β*-Mannosyl Phosphoisoprenoid from *Mycobacterium avium*, through Synthesis*J. Am. Chem. Soc.* **2002**, *124*, 2263-2266.

(4) Li, J.; Li, W.; Yu, B. A divergent approach to the synthesis of simplexides and congeners via a late-stage olefin cross-metathesis reaction. *Org. Biomol. Chem.* **2013**, *11*, 4971-4974.

^{1}H and ^{13}C NMR Spectra

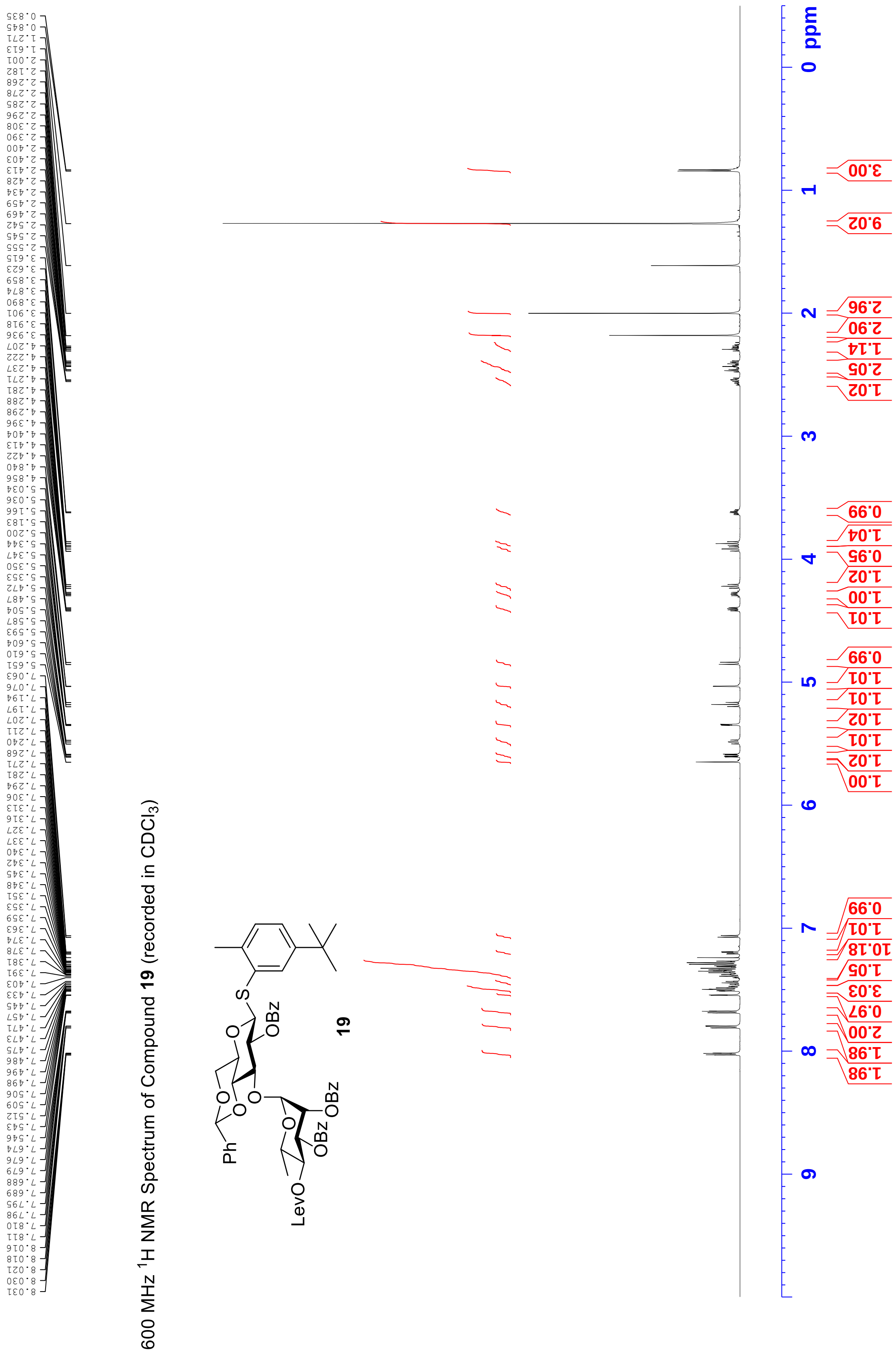

600 MHz ^{1}H NMR Spectrum of Compound **19** (recorded in $CDCl_3$)

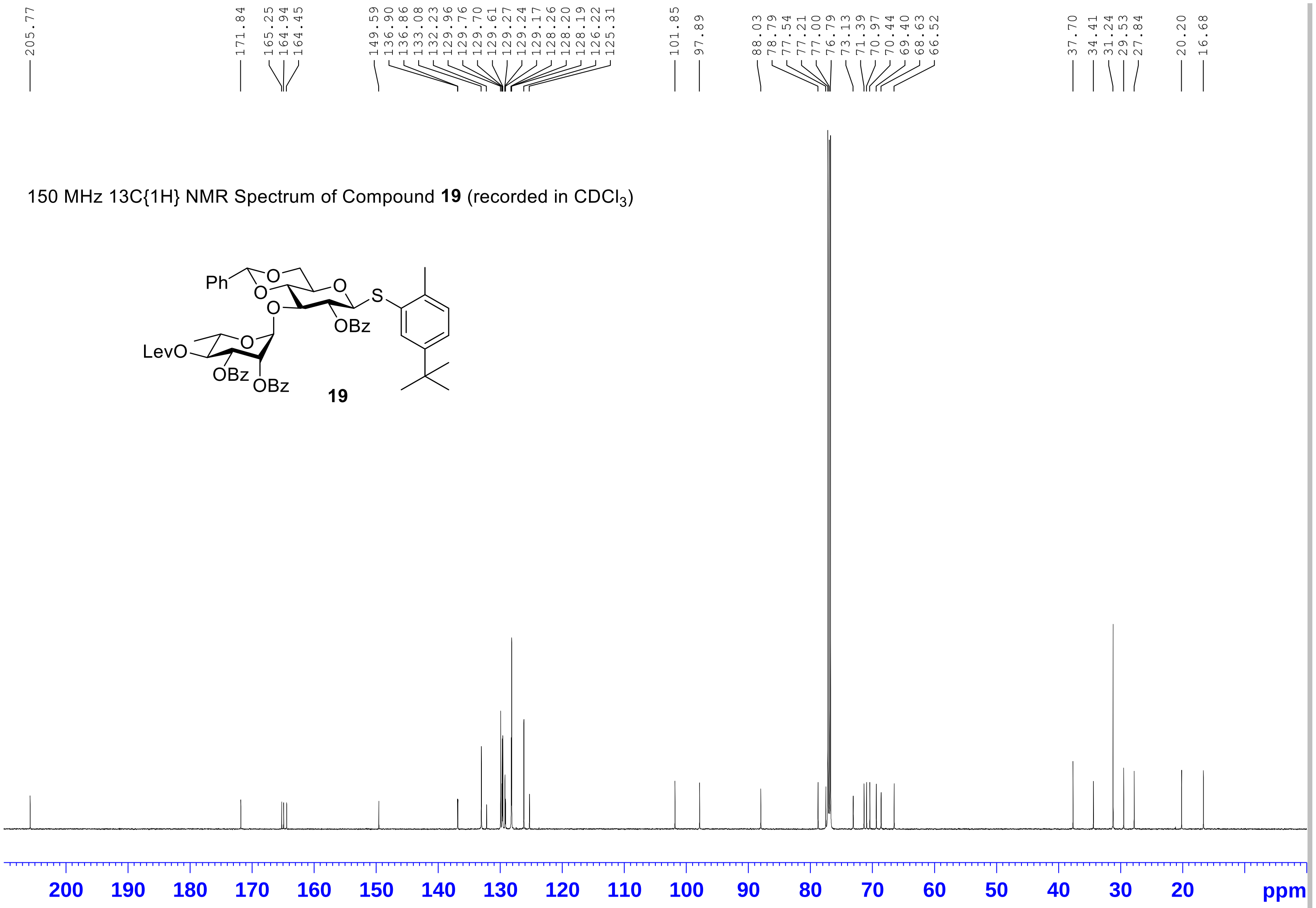

150 MHz 13C{1H} NMR Spectrum of Compound **19** (recorded in $CDCl_3$)

600 MHz ^{1}H NMR Spectrum of Compound **16** (recorded in $CDCl_3$)

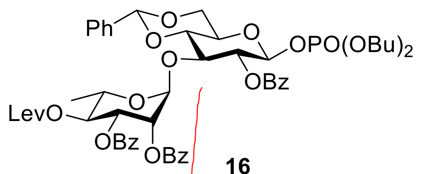

16

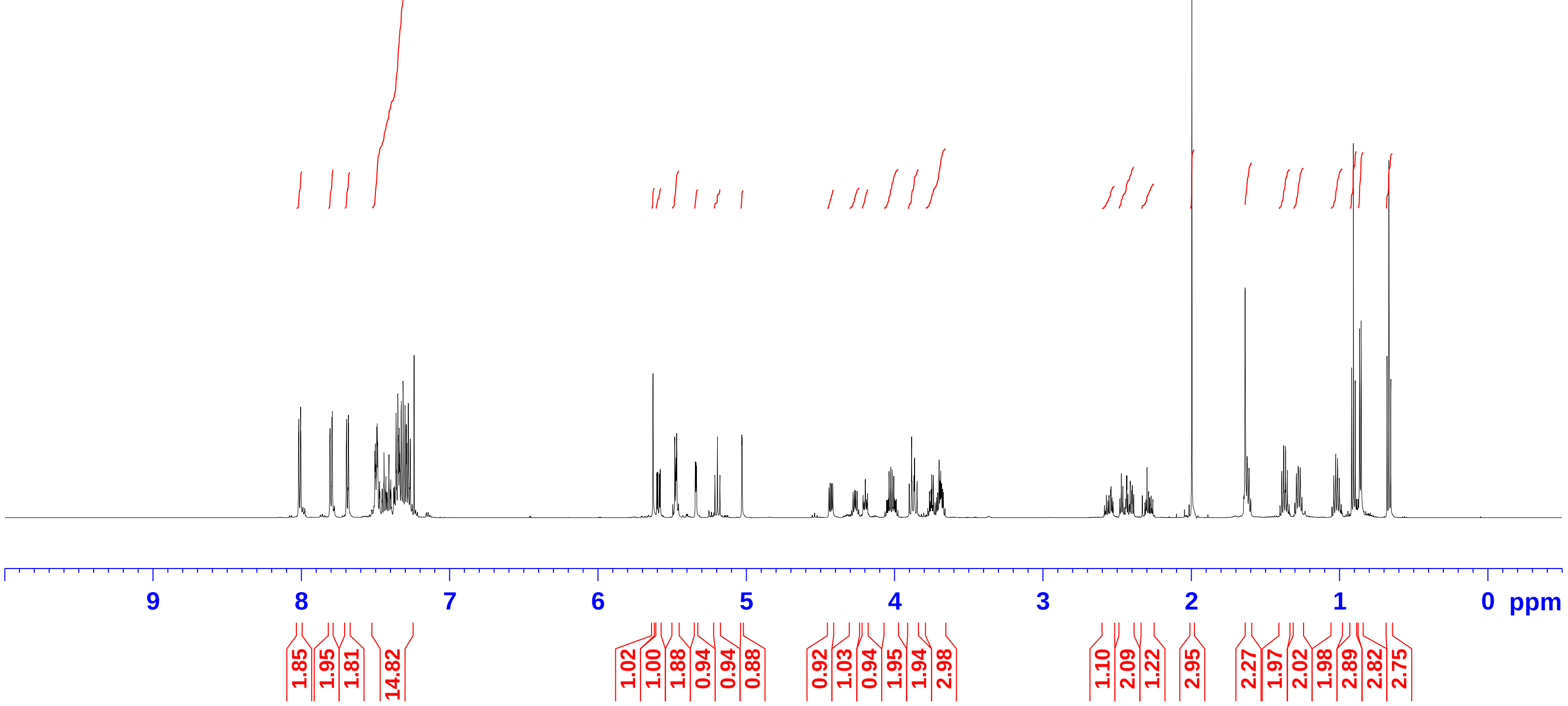

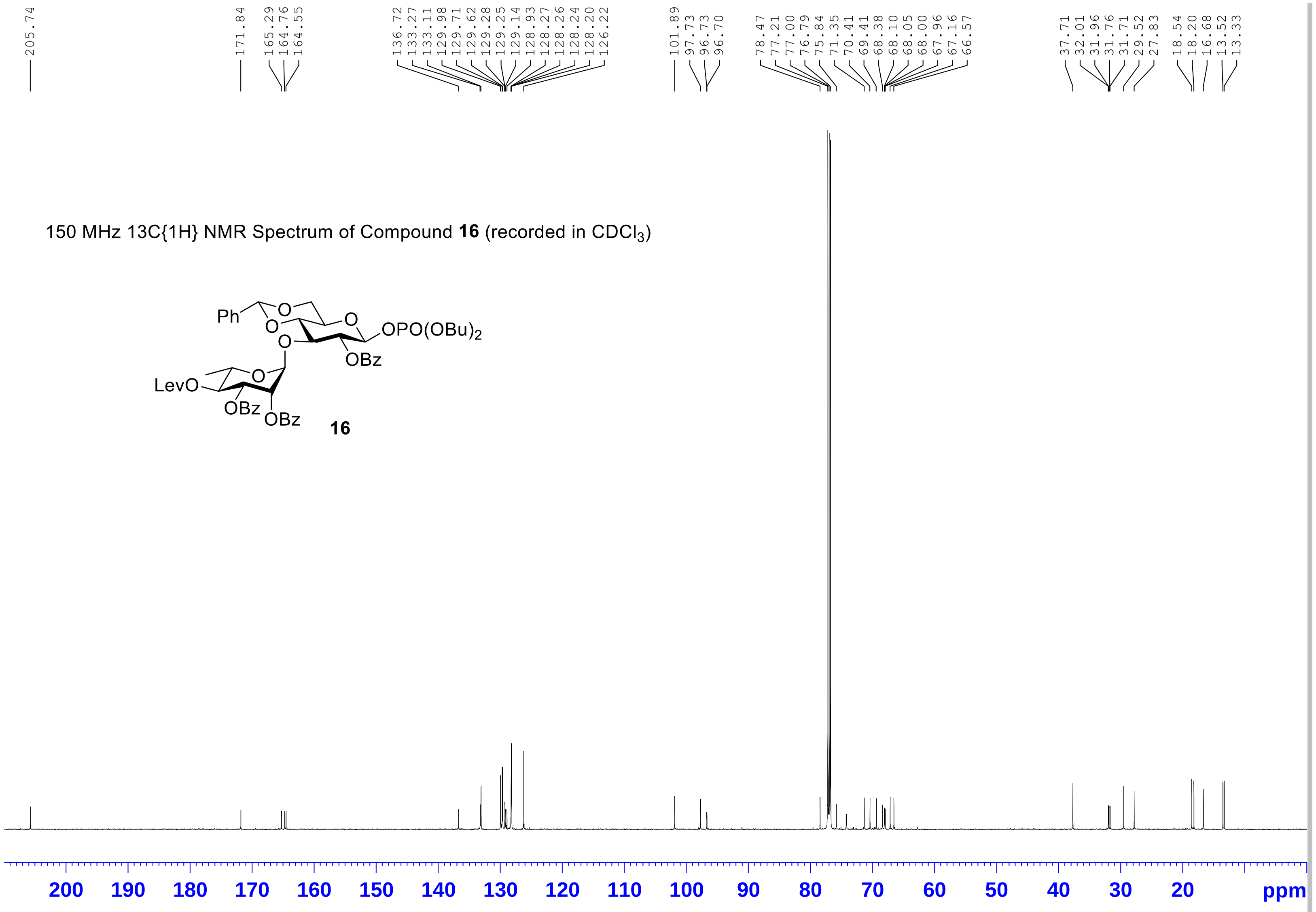

150 MHz 13C{1H} NMR Spectrum of Compound **16** (recorded in $CDCl_3$)

600 MHz ^{1}H NMR Spectrum of Compound **21** (recorded in $CDCl_3$)

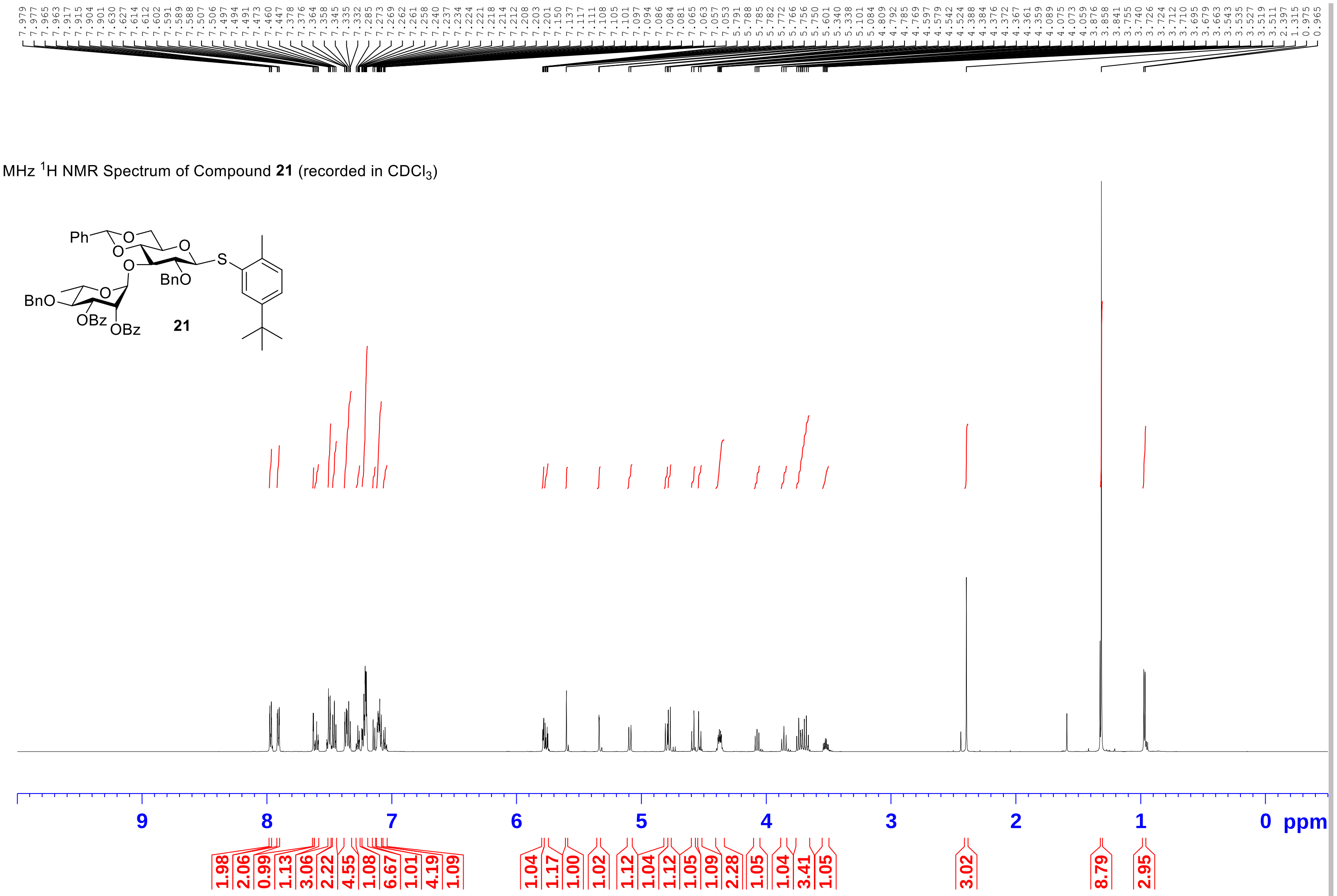

150 MHz 13C{1H} NMR Spectrum of Compound **21** (recorded in $CDCl_3$)

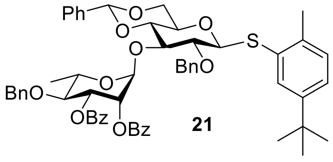

21

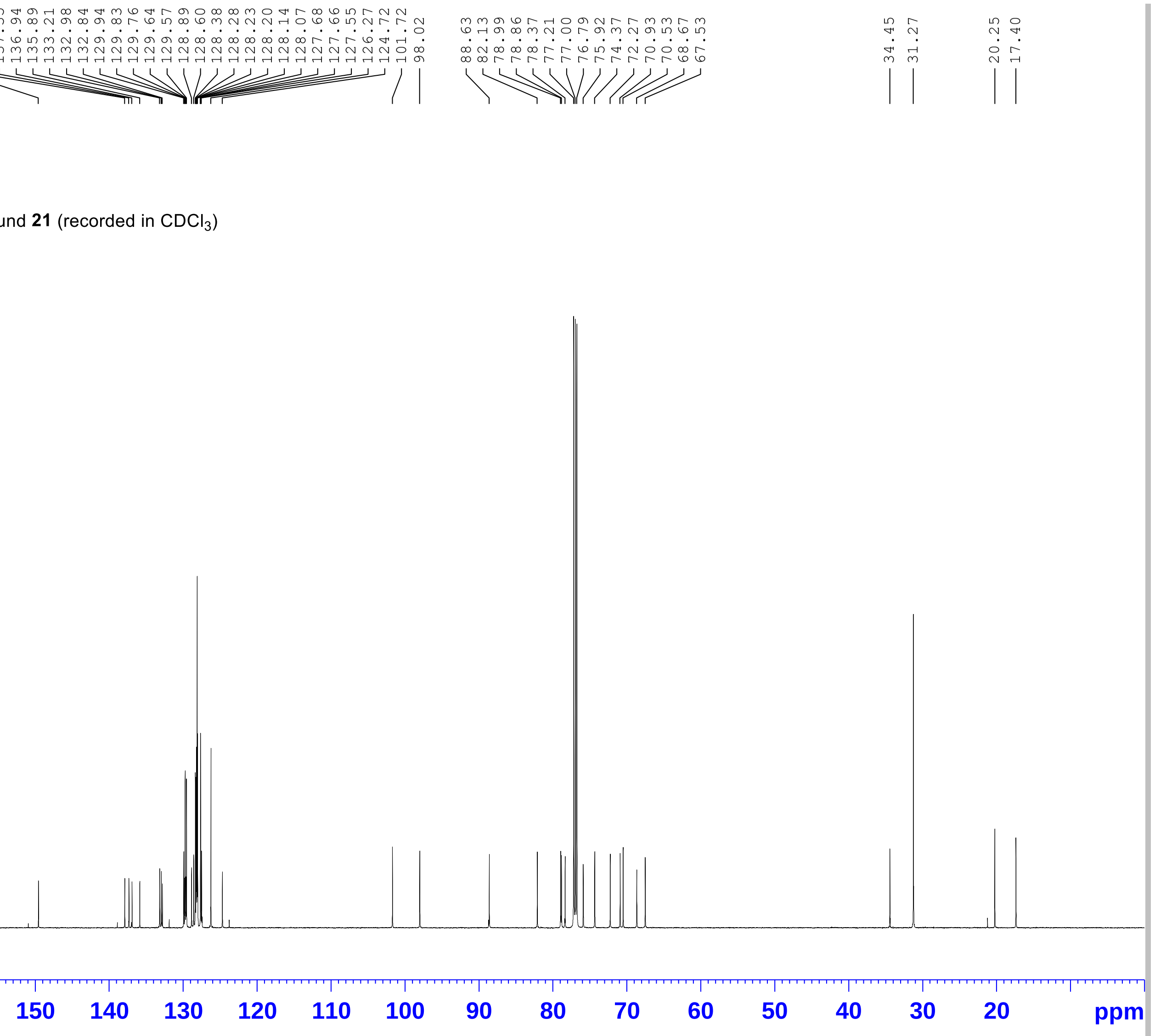

600 MHz ^{1}H NMR Spectrum of Compound **22** (recorded in $CDCl_3$)

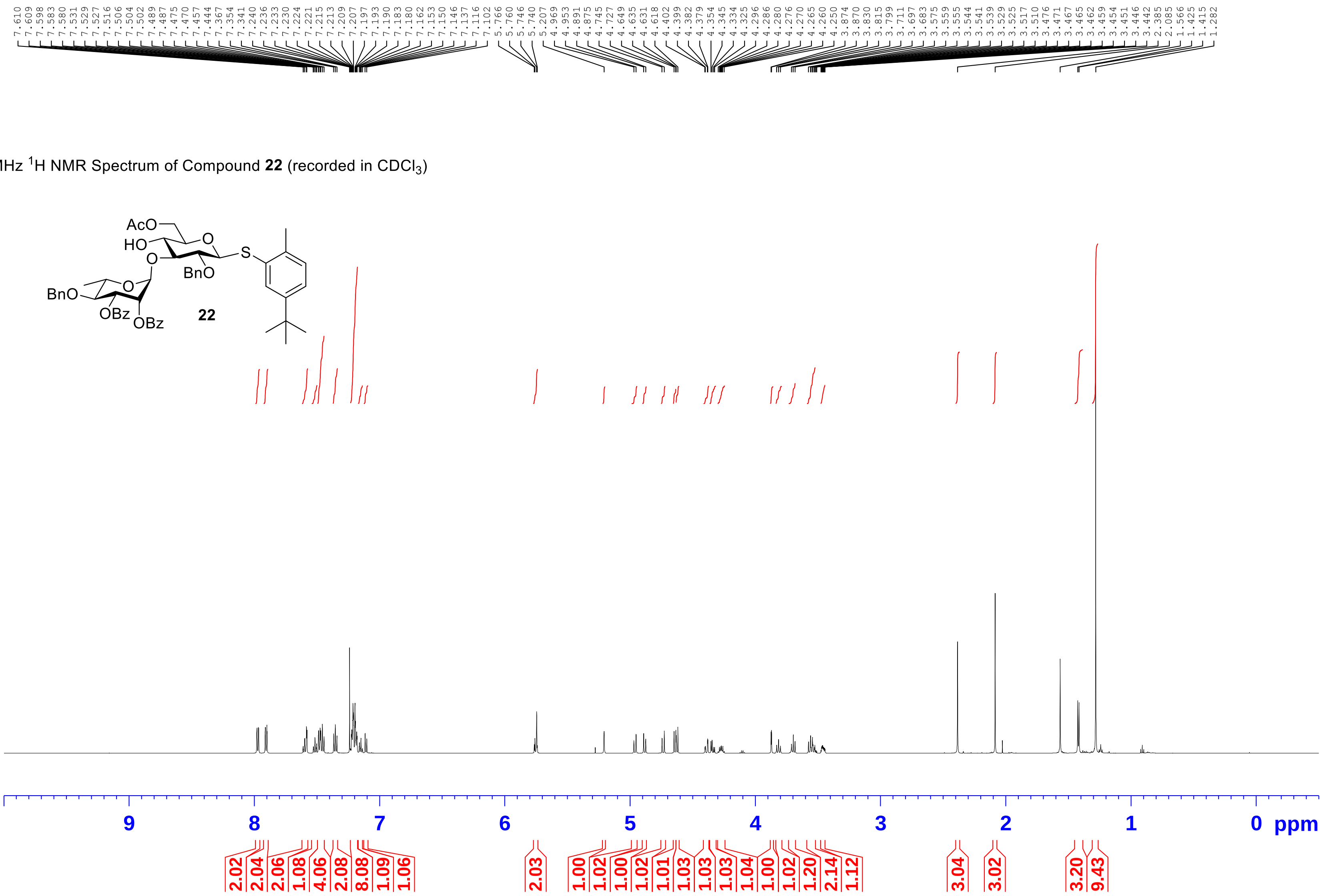

150 MHz 13C{1H} NMR Spectrum of Compound **22** (recorded in $CDCl_3$)

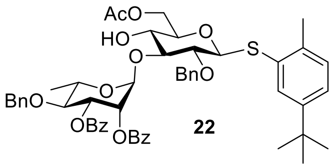

22

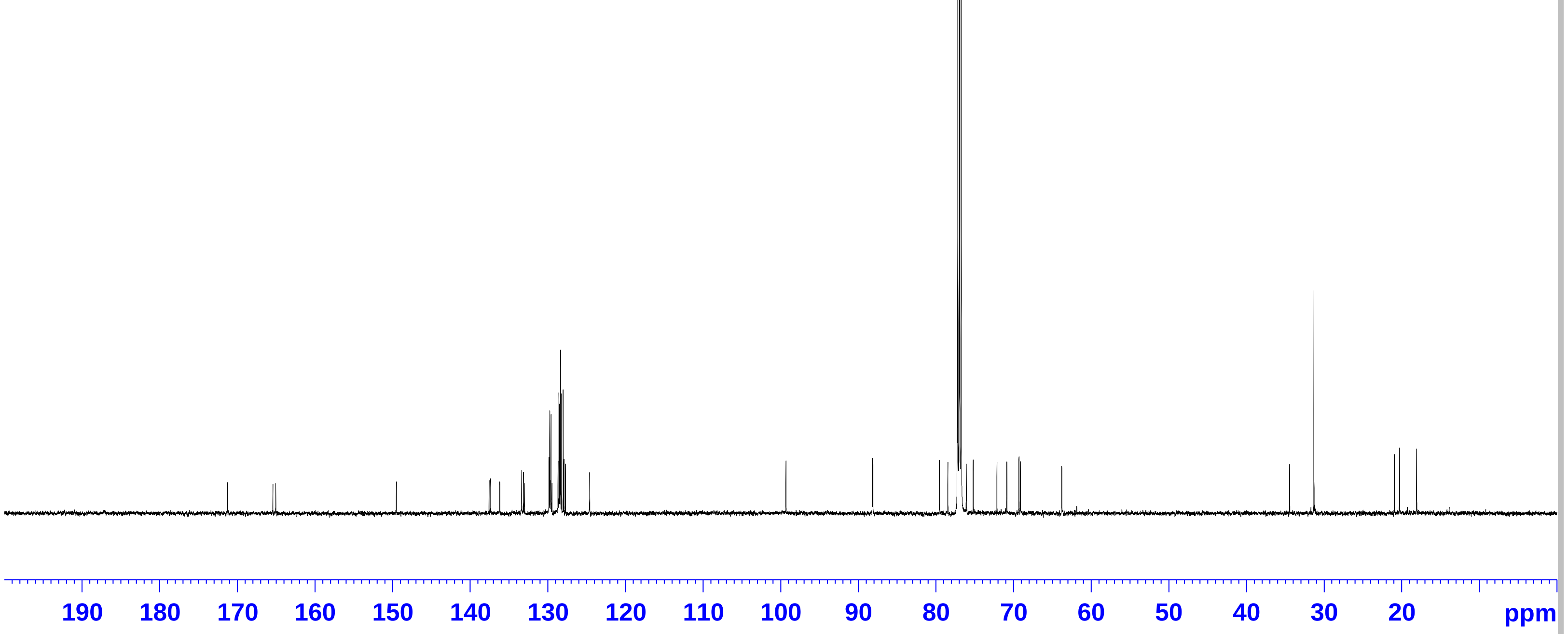

600 MHz ^{1}H NMR Spectrum of Compound **23a** (recorded in $CDCl_3$)

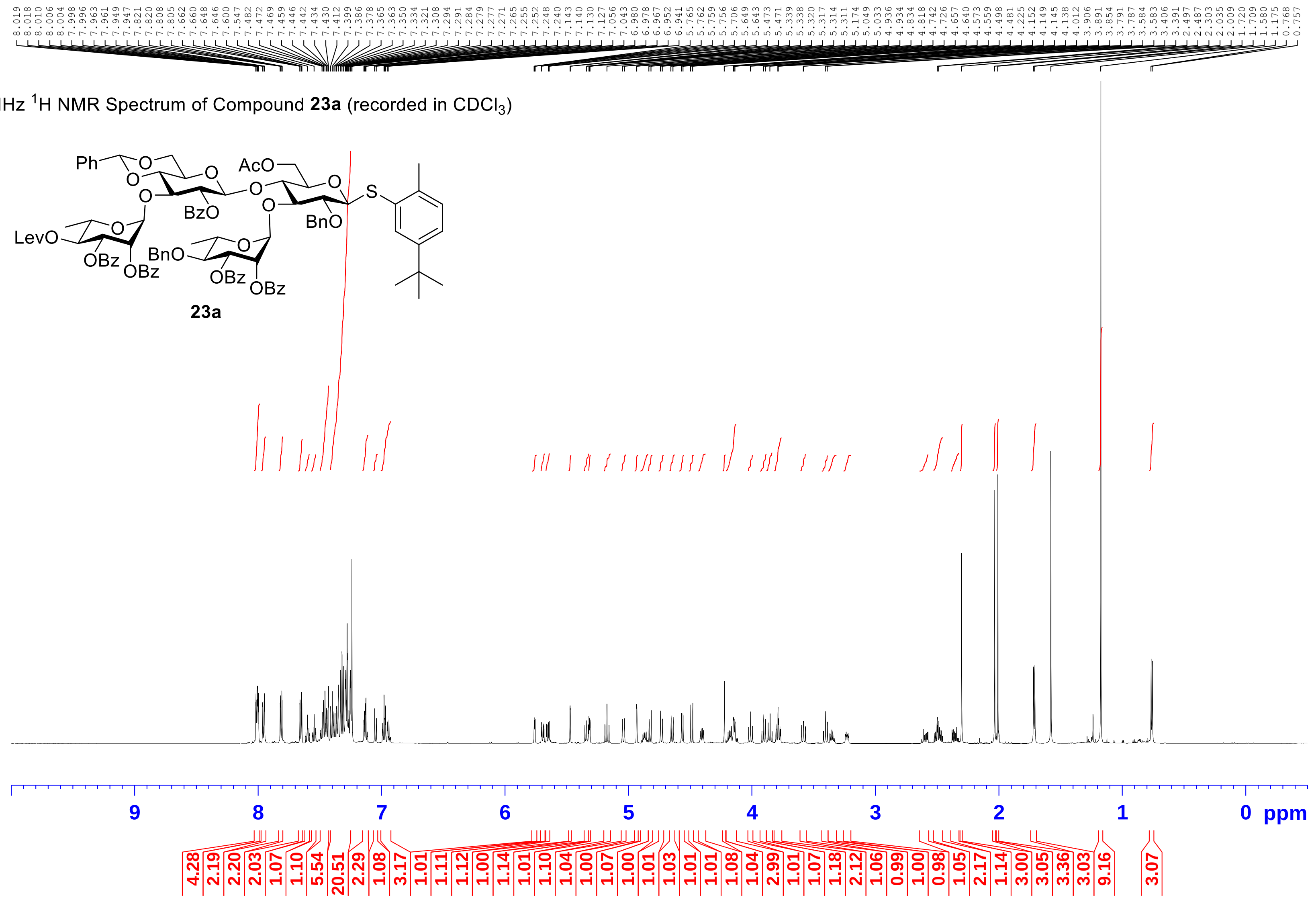

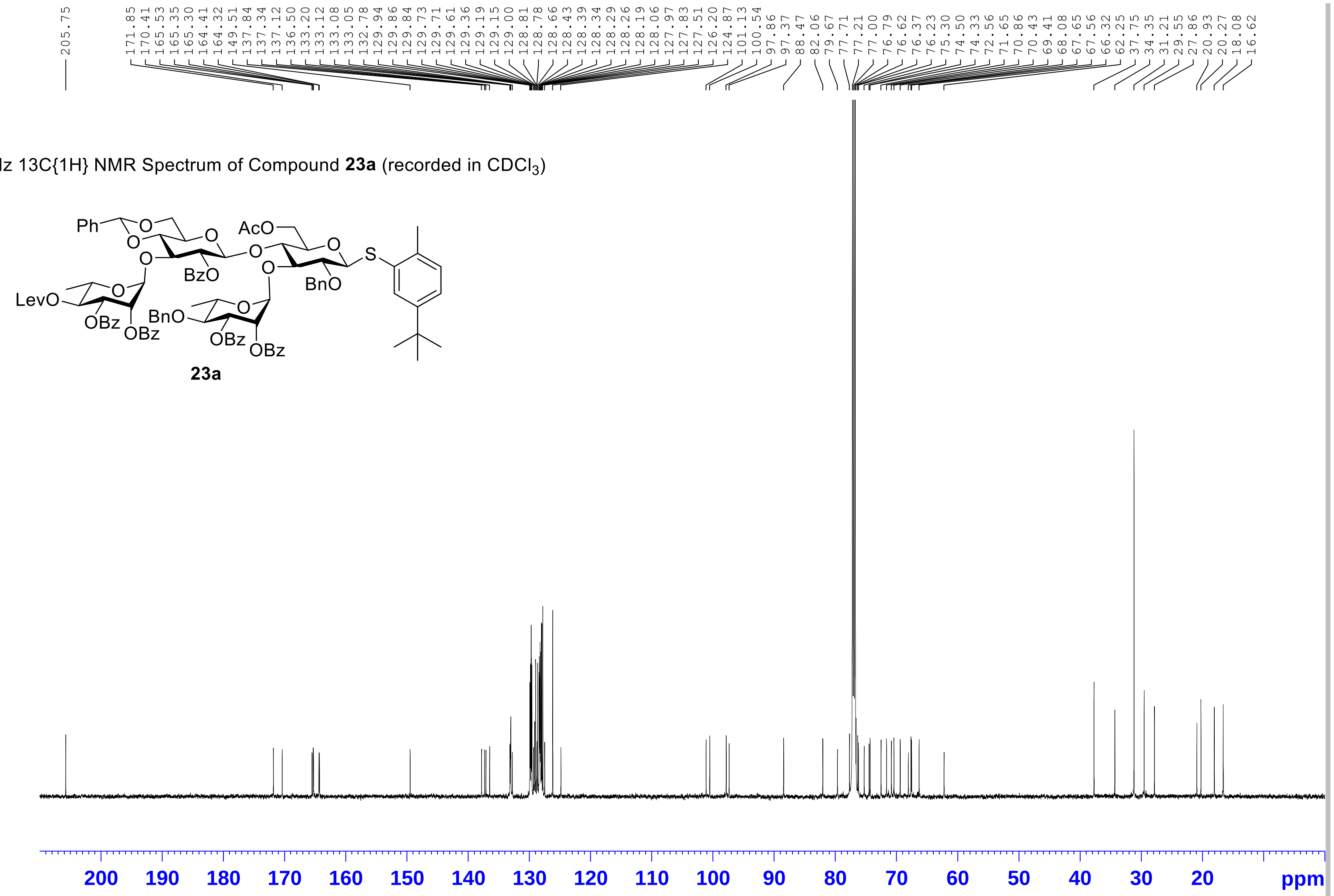

150 MHz 13C{1H} NMR Spectrum of Compound **23a** (recorded in $CDCl_3$)

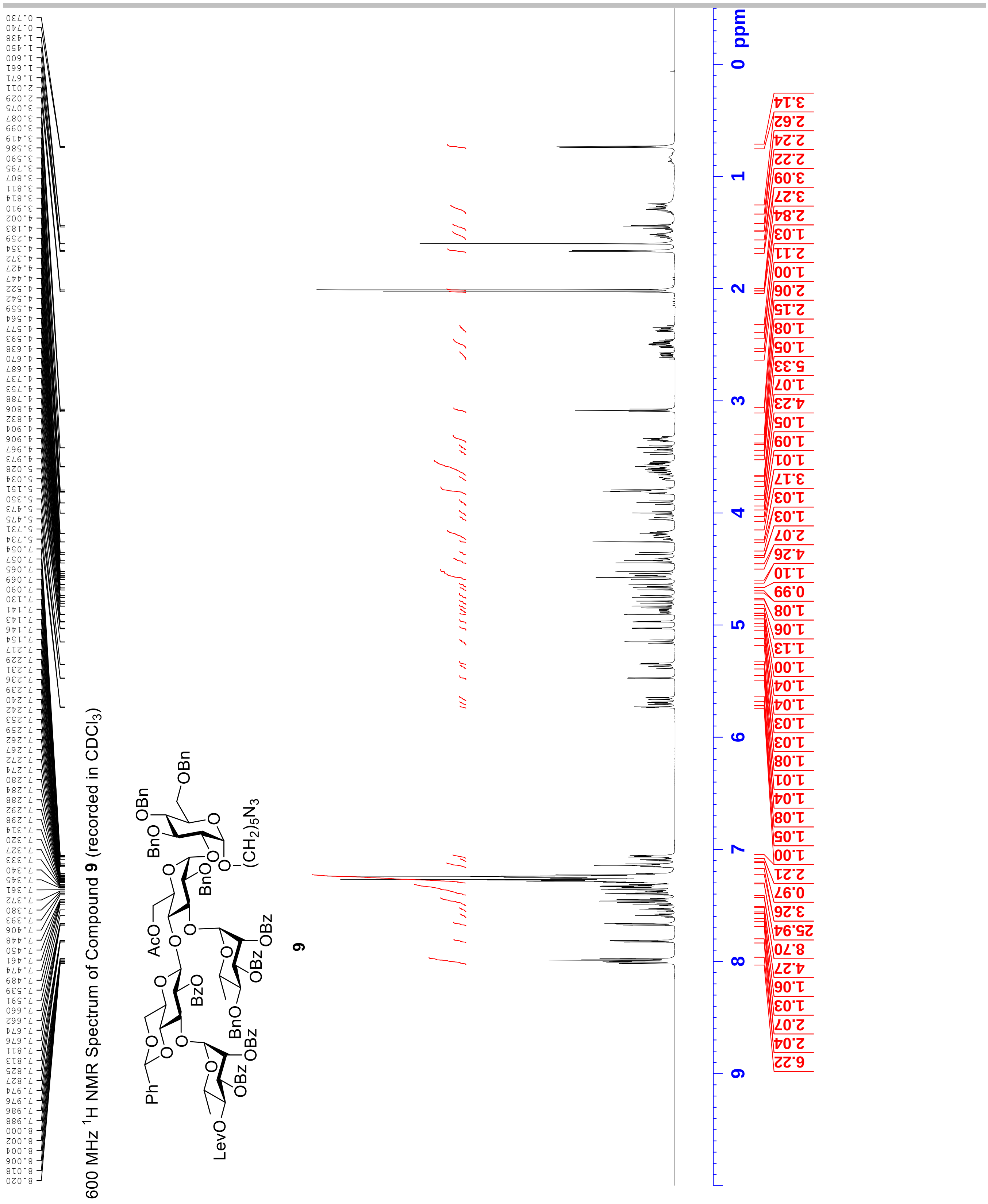

600 MHz ^{1}H NMR Spectrum of Compound **9** (recorded in $CDCl_3$)

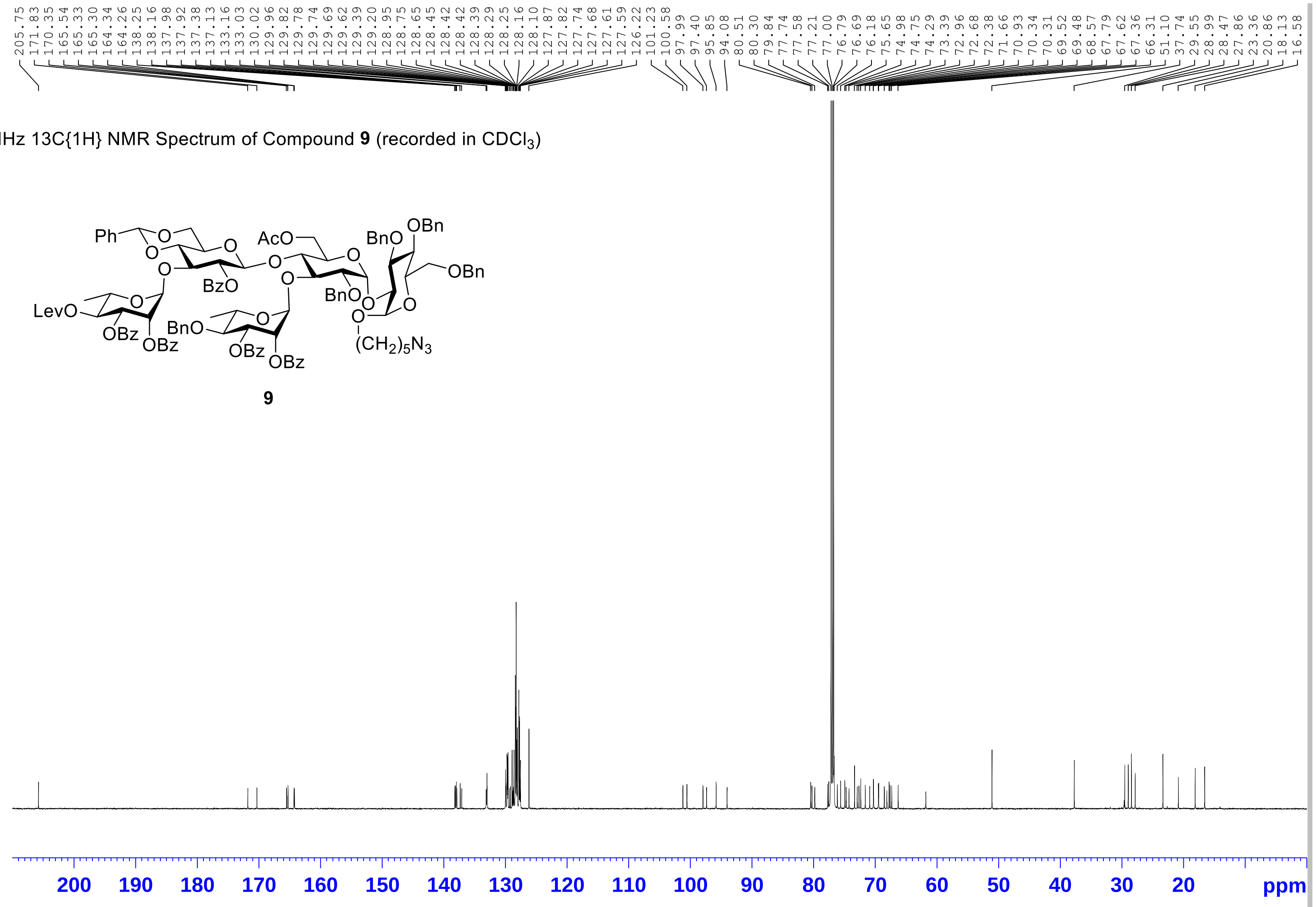

9

150 MHz 13C{1H} NMR Spectrum of Compound **9** (recorded in $CDCl_3$)

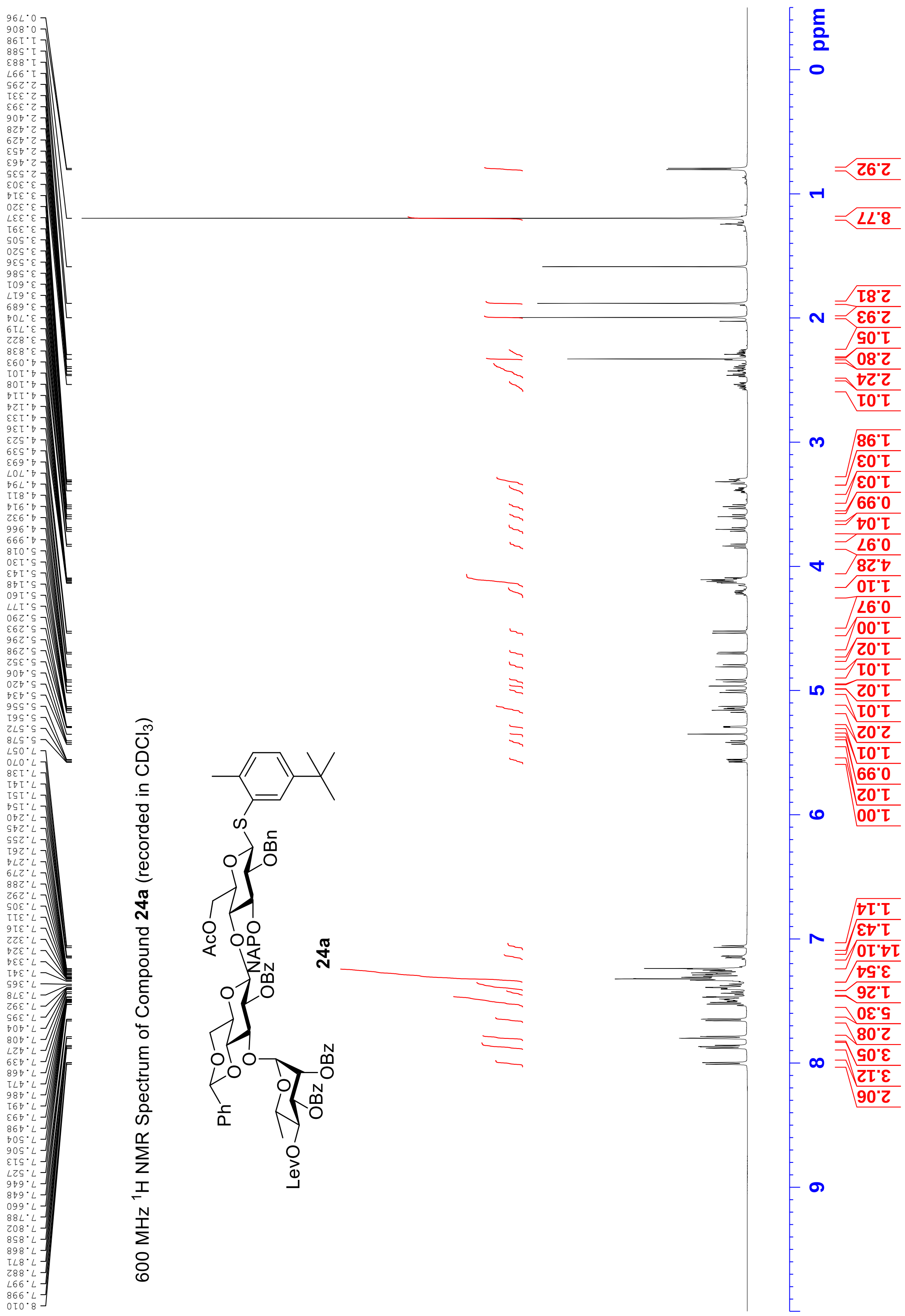

600 MHz ^{1}H NMR Spectrum of Compound **24a** (recorded in $CDCl_3$)

150 MHz 13C{1H} NMR Spectrum of Compound 24**a** (recorded in $CDCl_3$)

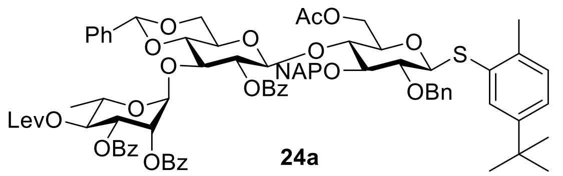

24a

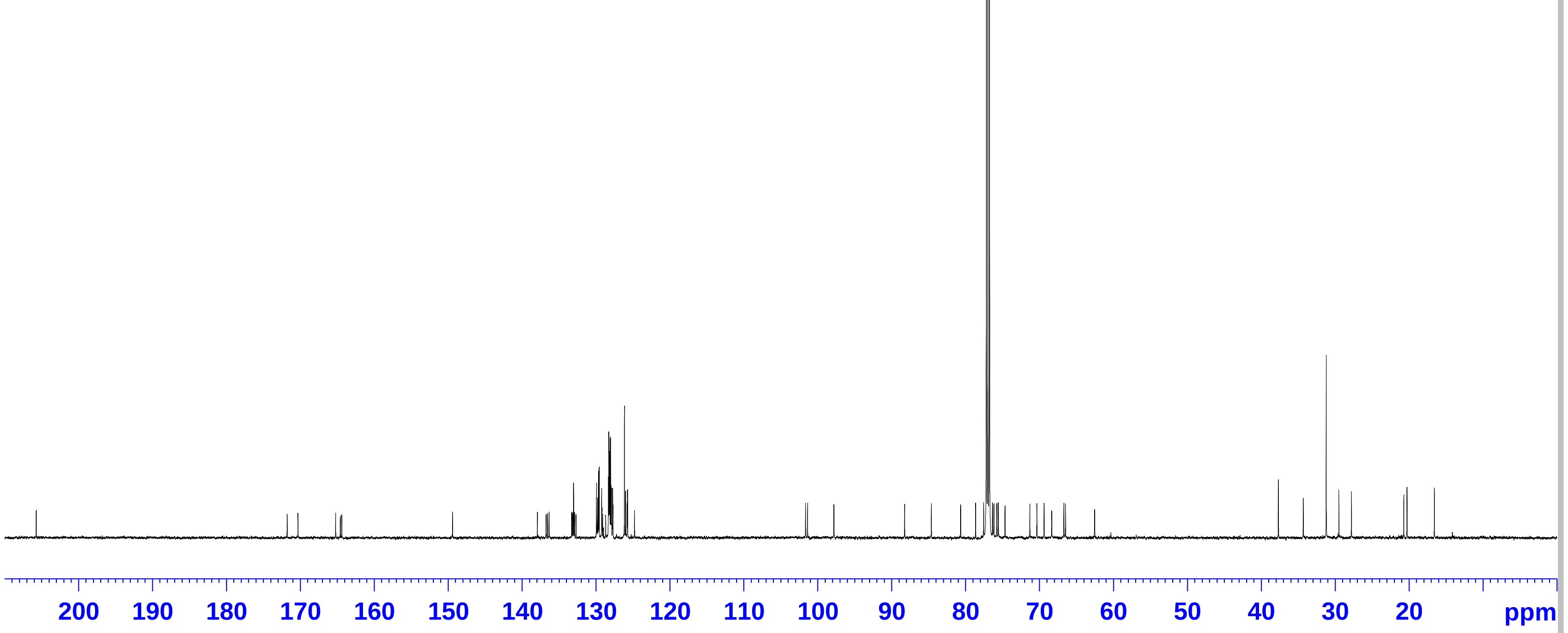

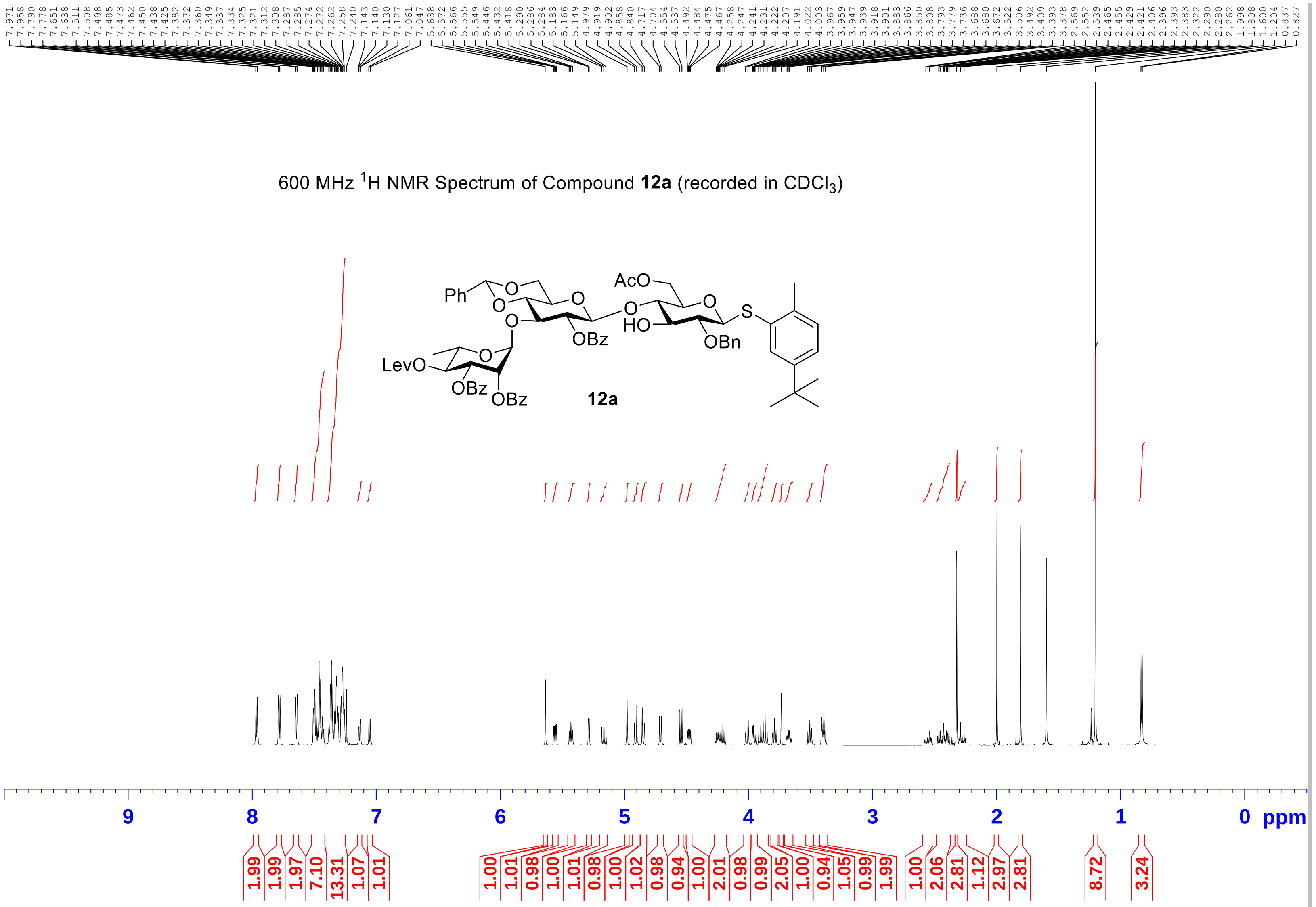
600 MHz ^{1}H NMR Spectrum of Compound **12a** (recorded in $CDCl_3$)
Ph
O
O
O
OBz
O
AcO
O
HO
O
S
OBn
LevO
O
OBz
OBz
12a
7.971 7.958 7.790 7.778 7.651 7.638 7.511 7.508 7.498 7.485 7.473 7.462 7.450 7.438 7.425 7.382 7.372 7.360 7.349 7.337 7.334 7.325 7.321 7.312 7.308 7.287 7.285 7.274 7.272 7.262 7.258 7.240 7.143 7.140 7.130 7.127 7.061 7.047 5.638 5.572 5.566 5.555 5.549 5.446 5.432 5.418 5.290 5.286 5.284 5.183 5.166 5.149 4.979 4.919 4.902 4.858 4.840 4.717 4.704 4.554 4.537 4.492 4.484 4.475 4.467 4.258 4.247 4.241 4.231 4.222 4.207 4.191 4.022 4.003 3.967 3.959 3.947 3.939 3.918 3.901 3.883 3.866 3.850 3.808 3.793 3.779 3.736 3.688 3.680 3.672 3.522 3.506 3.492 3.409 3.393 3.378 2.569 2.552 2.539 2.465 2.455 2.429 2.421 2.406 2.396 2.393 2.383 2.322 2.290 2.280 2.262 1.998 1.808 1.600 1.204 0.837 0.827
9
8
7
6
5
4
3
2
1
0 ppm
1.99 1.99 1.97 7.10 13.31 1.07 1.01
1.00 1.01 0.98 1.00 1.01 0.98 1.00 1.02 0.98 0.94 1.00 2.01 0.98 0.99 2.05 1.00 0.94 1.05 0.99 1.99
1.00 2.06 2.81 1.12 2.97 2.81
8.72
3.24

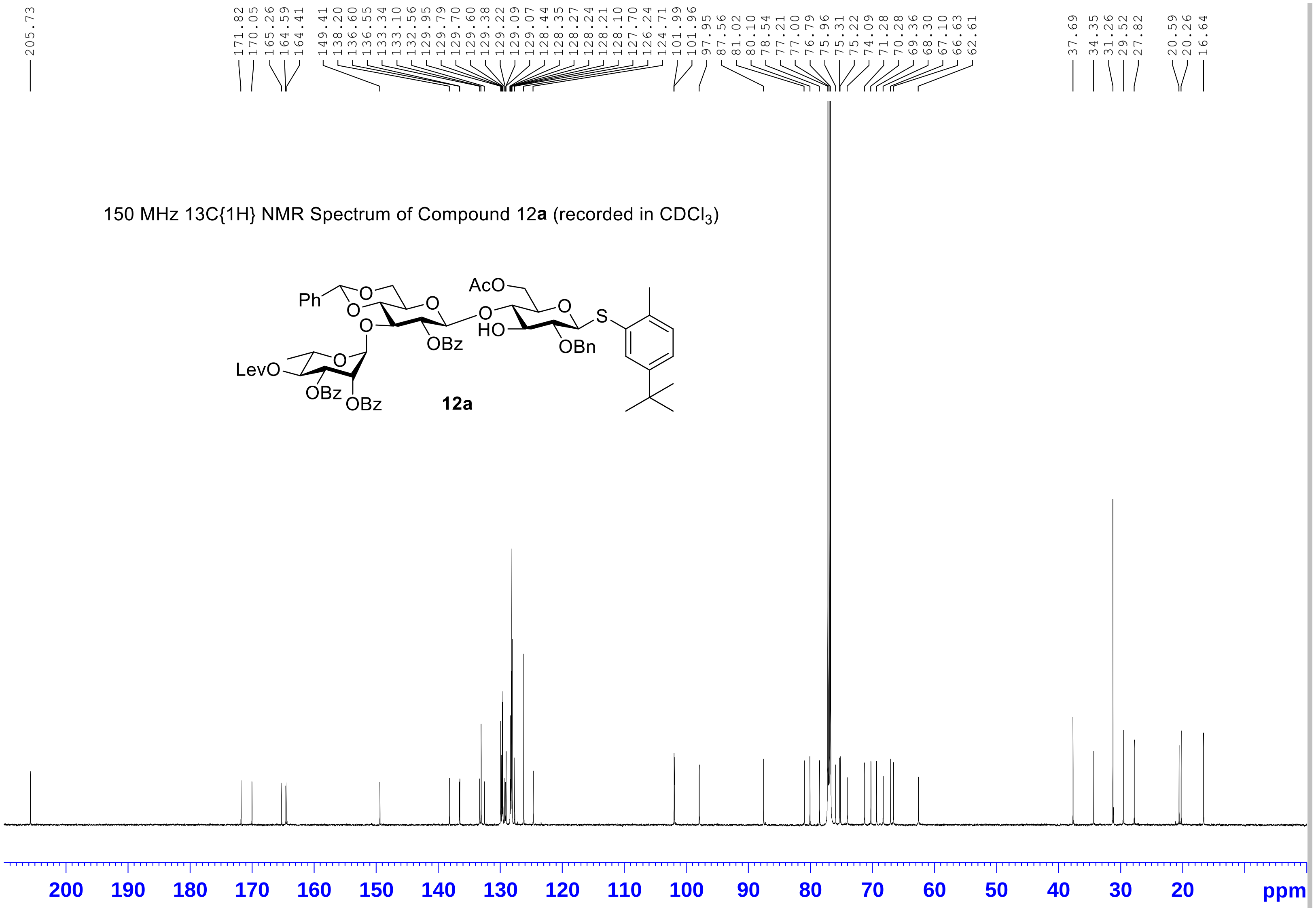

150 MHz 13C{1H} NMR Spectrum of Compound 12**a** (recorded in $CDCl_3$)

12a

600 MHz ^{1}H NMR Spectrum of Compound **25** (recorded in $CDCl_3$)

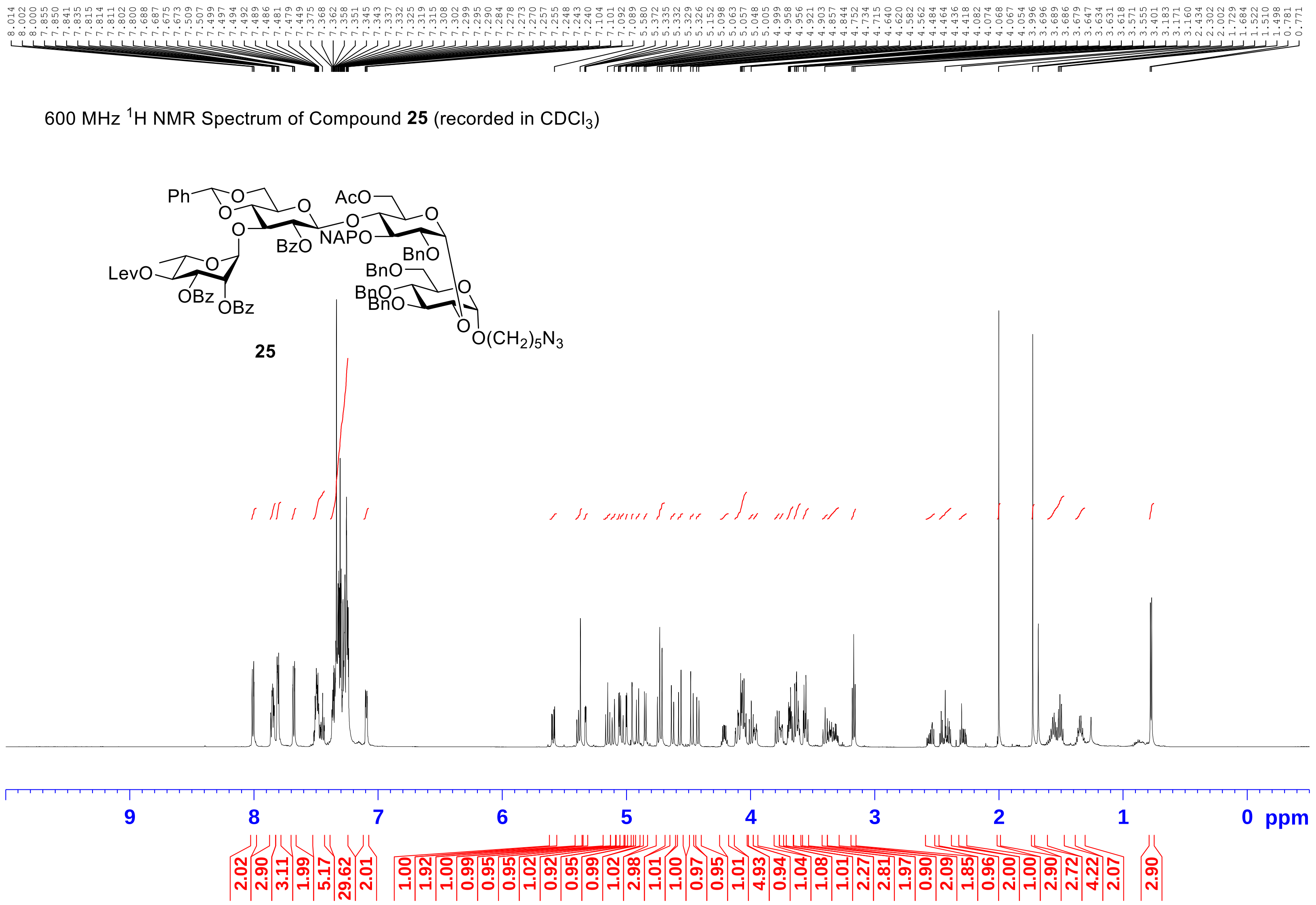

150 MHz 13C{1H} NMR Spectrum of Compound **25** (recorded in $CDCl_3$)

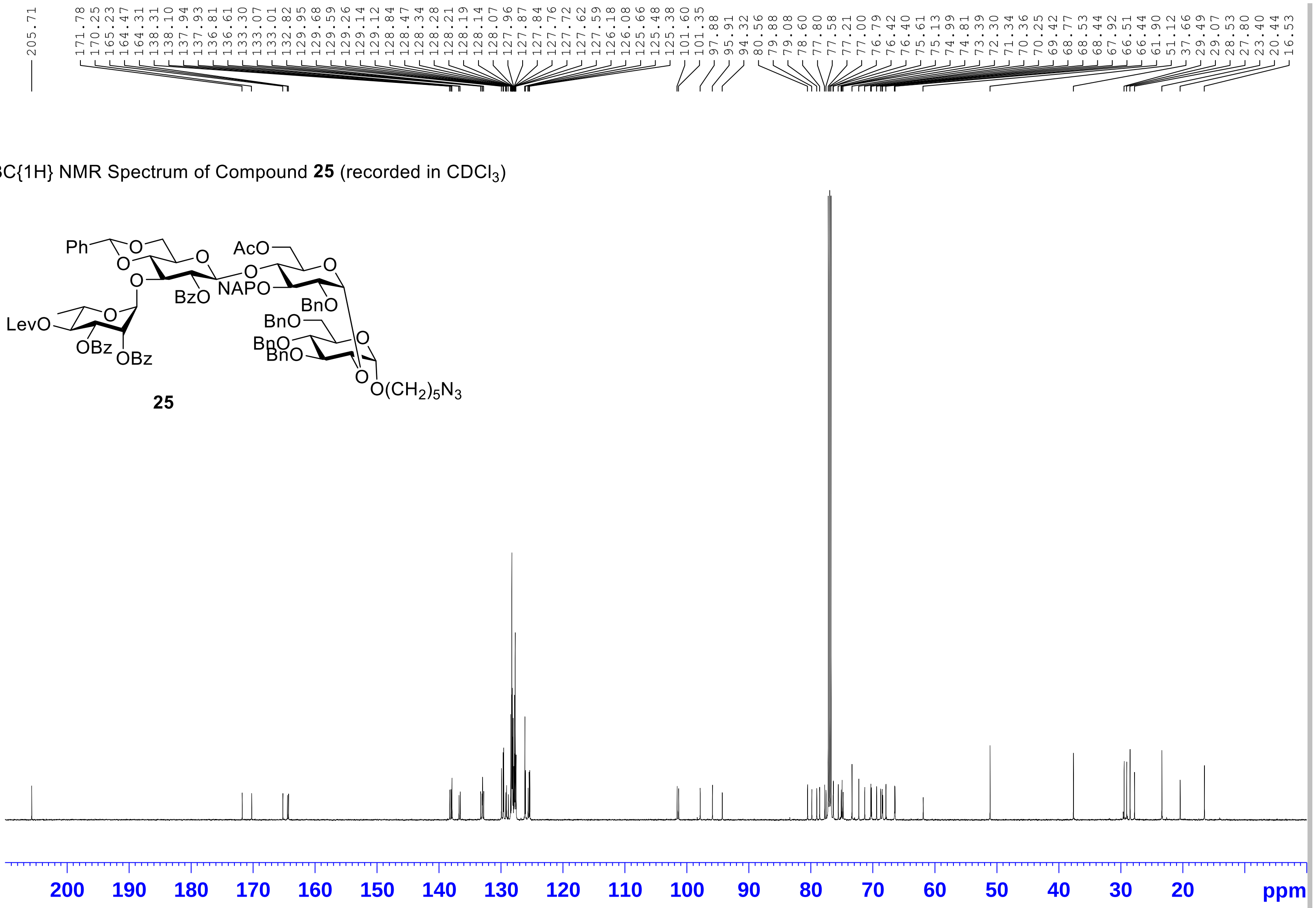

600 MHz ^{1}H NMR Spectrum of Compound **25 β-isomer** (recorded in $CDCl_3$)

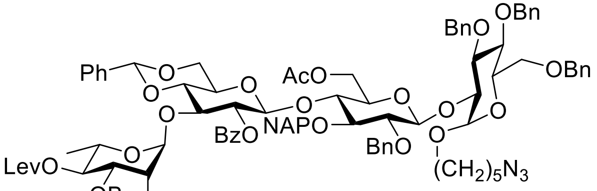

25 β-isomer

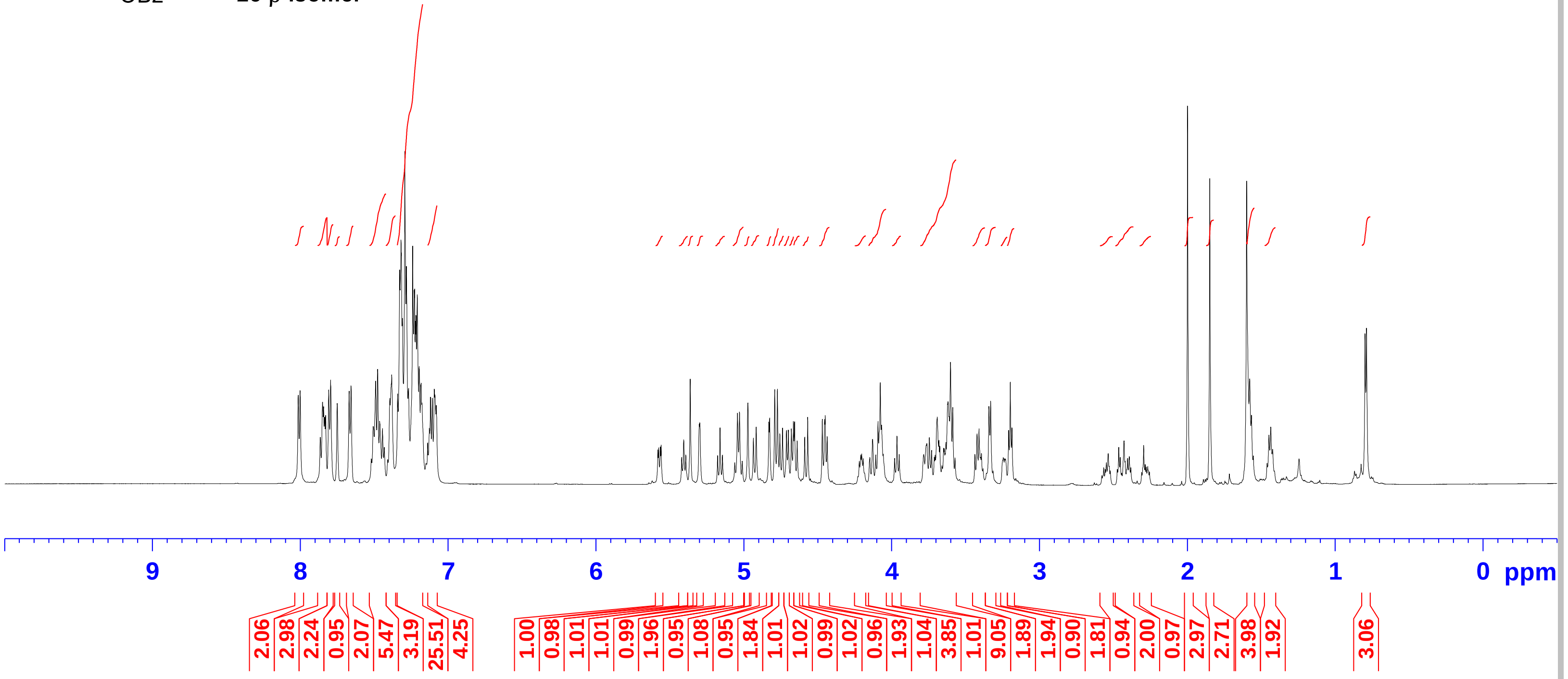

150 MHz 13C{1H} NMR Spectrum of Compound **25 β-isomer** (recorded in $CDCl_3$)

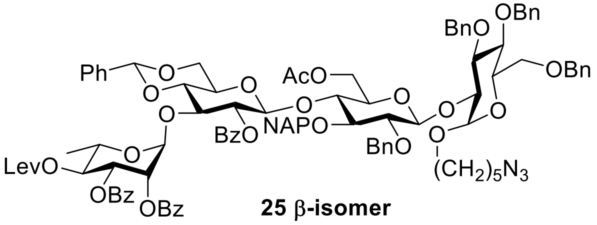

25 β-isomer

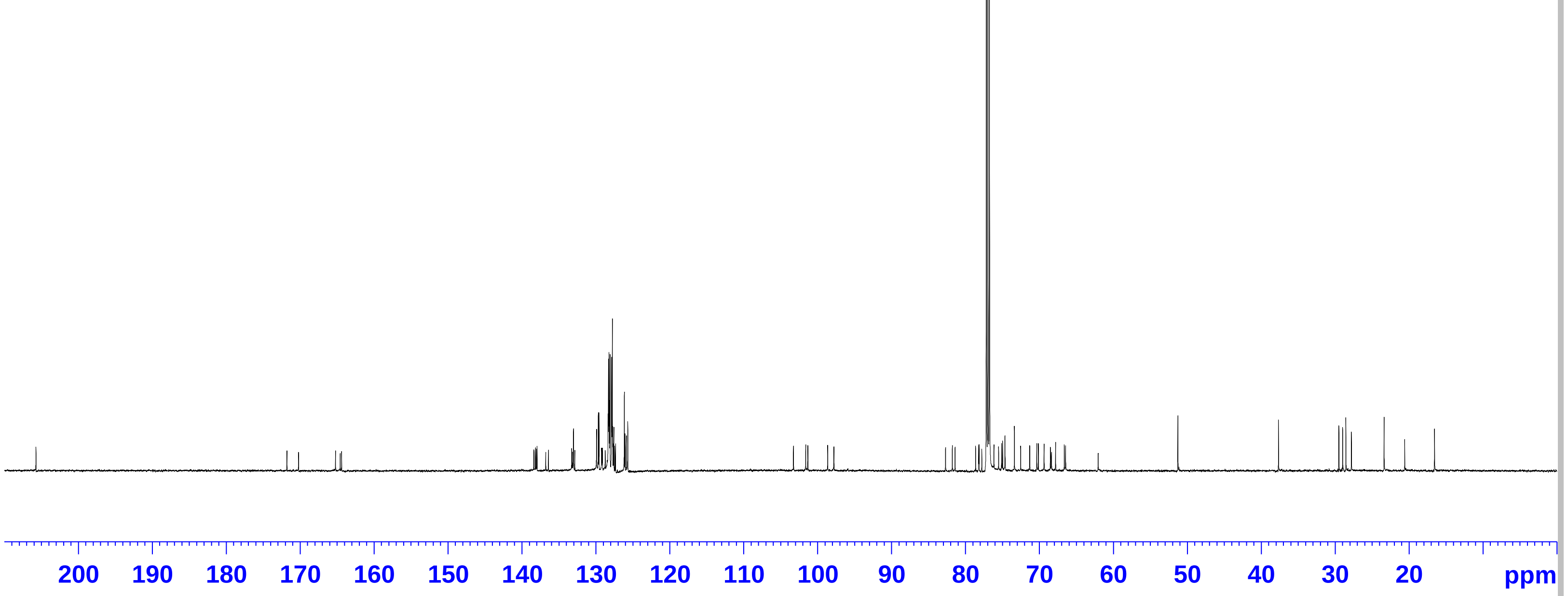

600 MHz ^{1}H NMR Spectrum of Compound **27a** (recorded in $CDCl_3$)

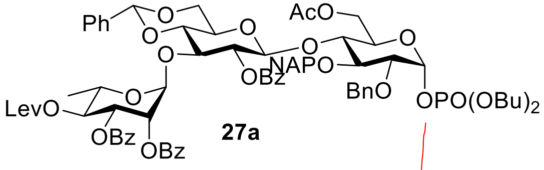

27a

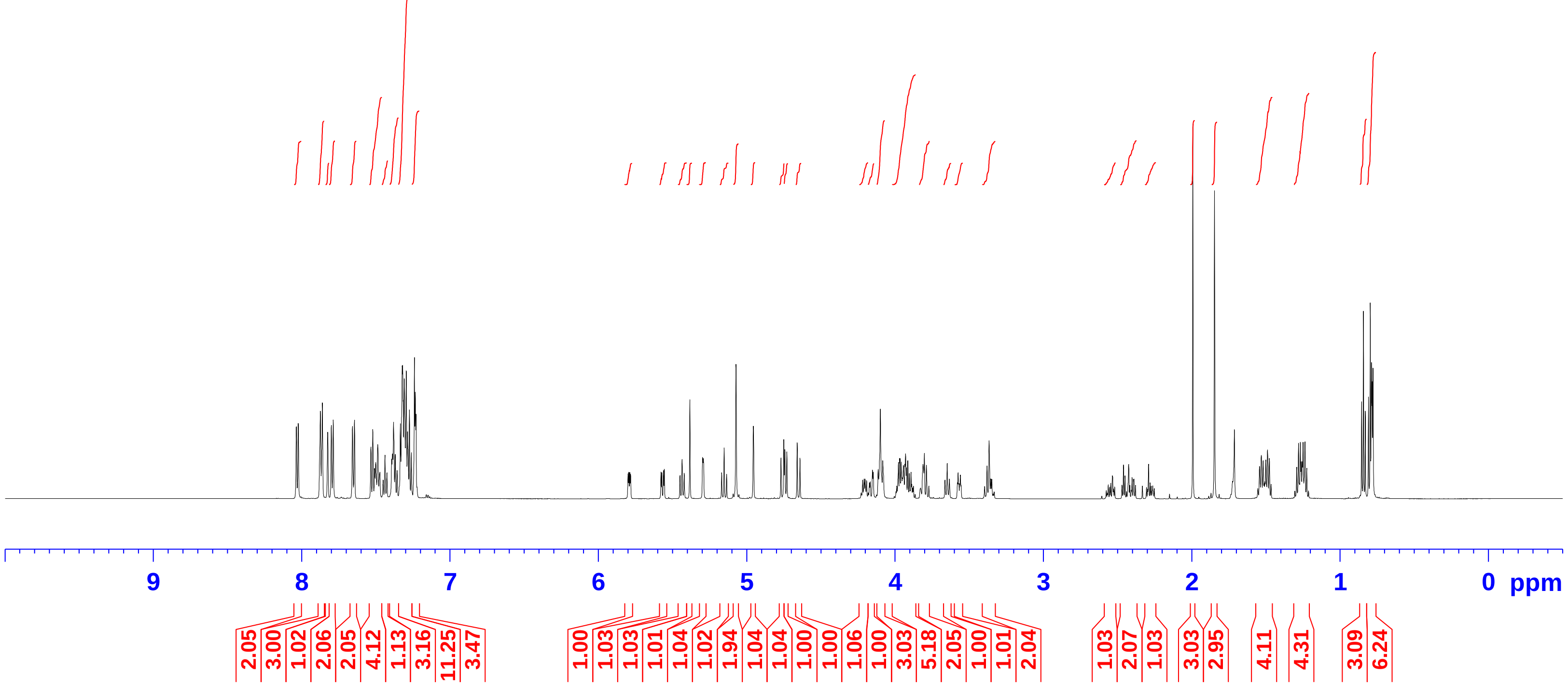

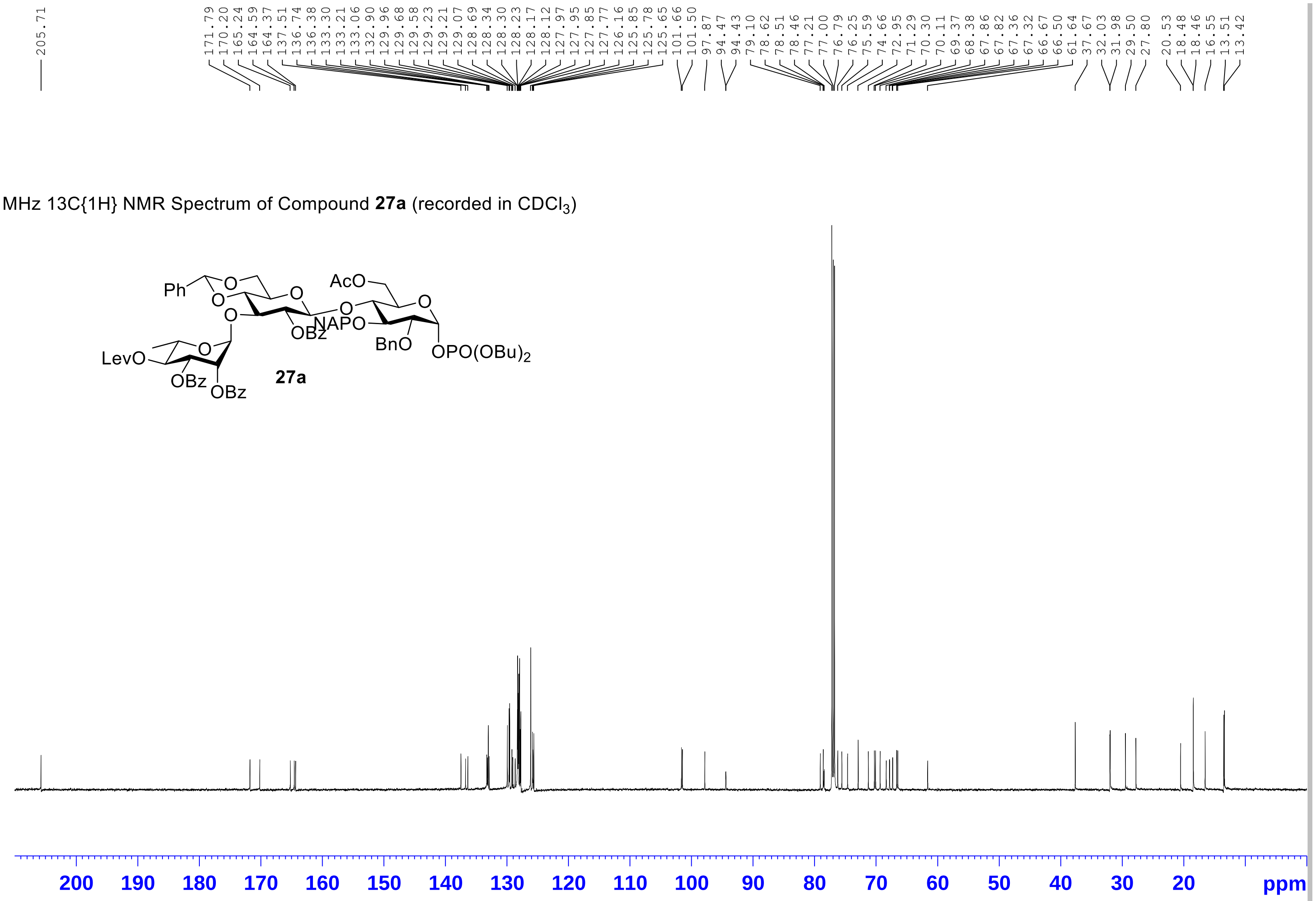

150 MHz 13C{1H} NMR Spectrum of Compound **27a** (recorded in $CDCl_3$)

600 MHz ^{1}H NMR Spectrum of Compound **26** (recorded in $CDCl_3$)

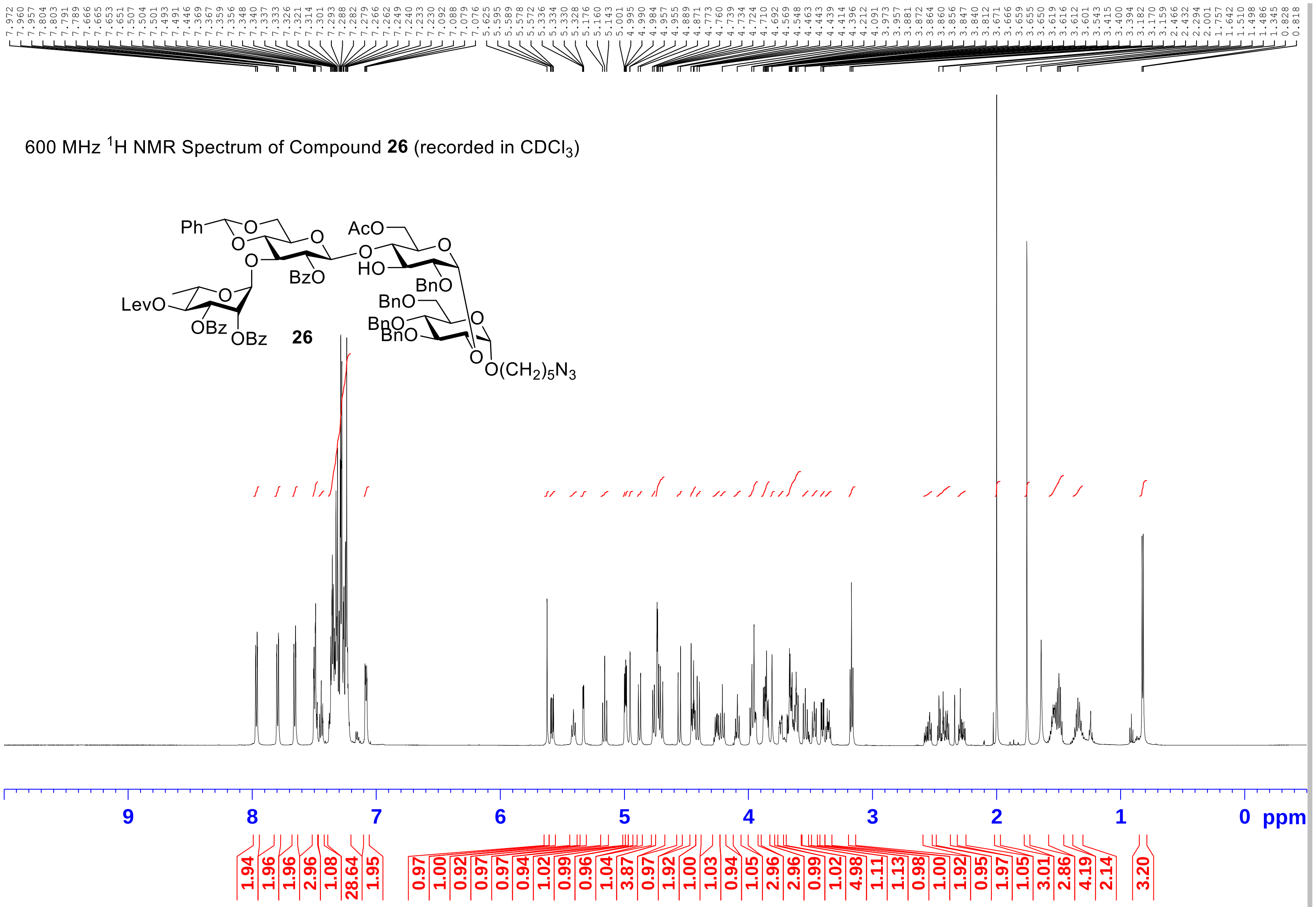

150 MHz 13C{1H} NMR Spectrum of Compound **26** (recorded in $CDCl_3$)

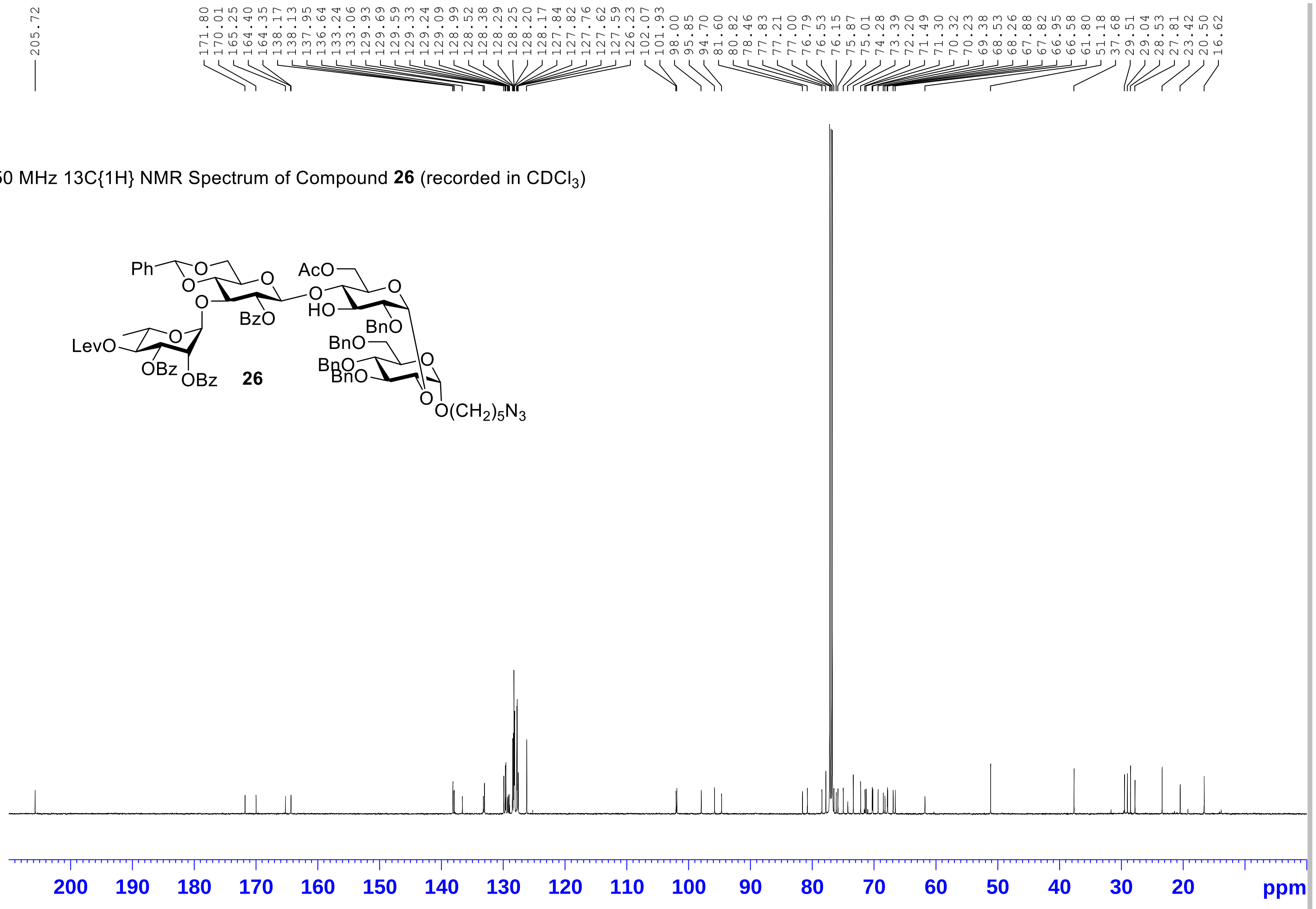

600 MHz ^1H NMR Spectrum of Compound **10** (recorded in $CDCl_3$)

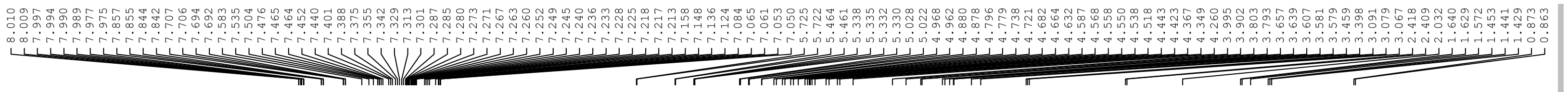

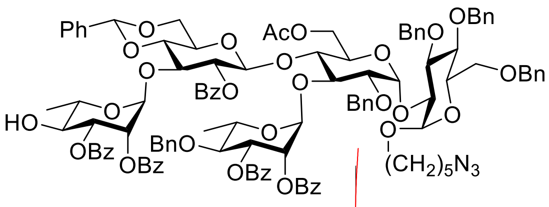

10

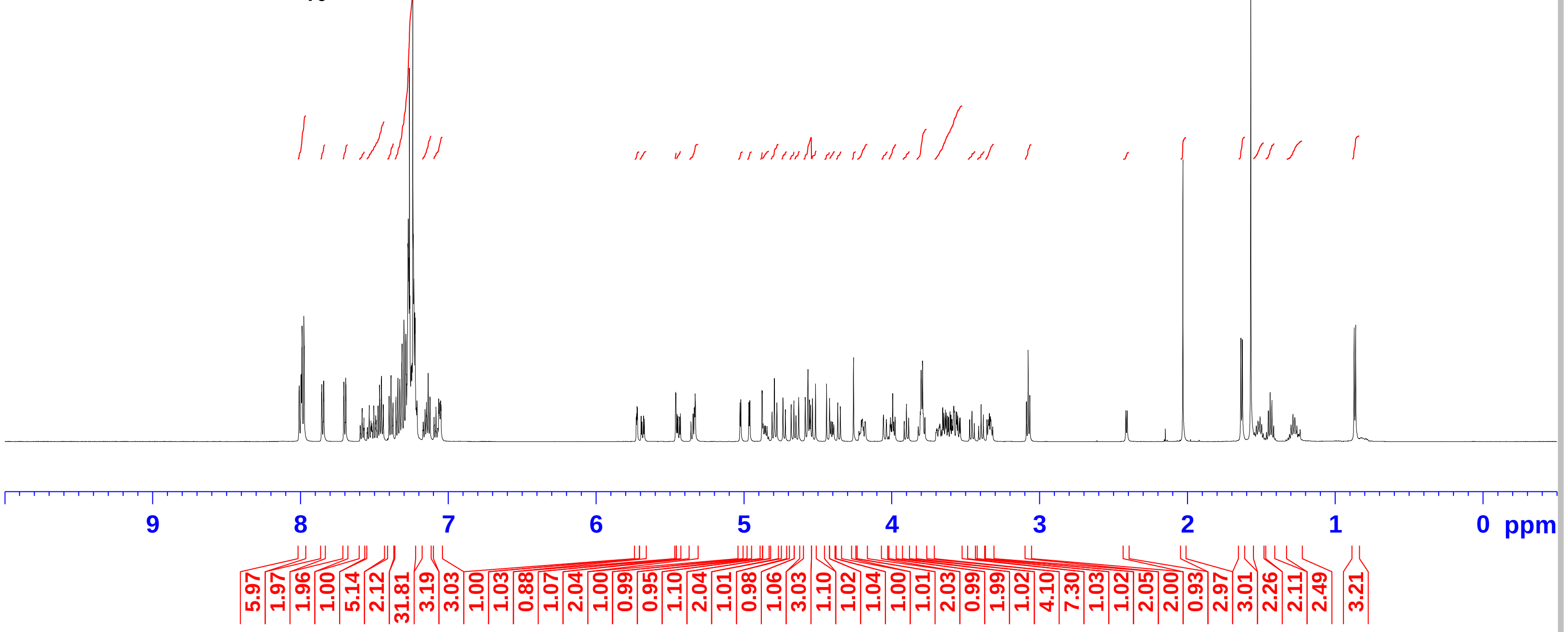

150 MHz 13C{1H} NMR Spectrum of Compound **10** (recorded in $CDCl_3$)

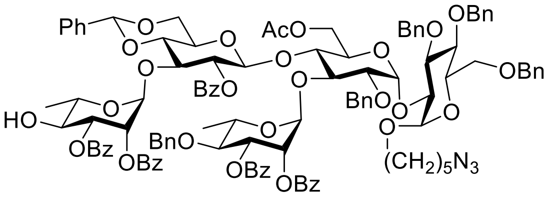

10

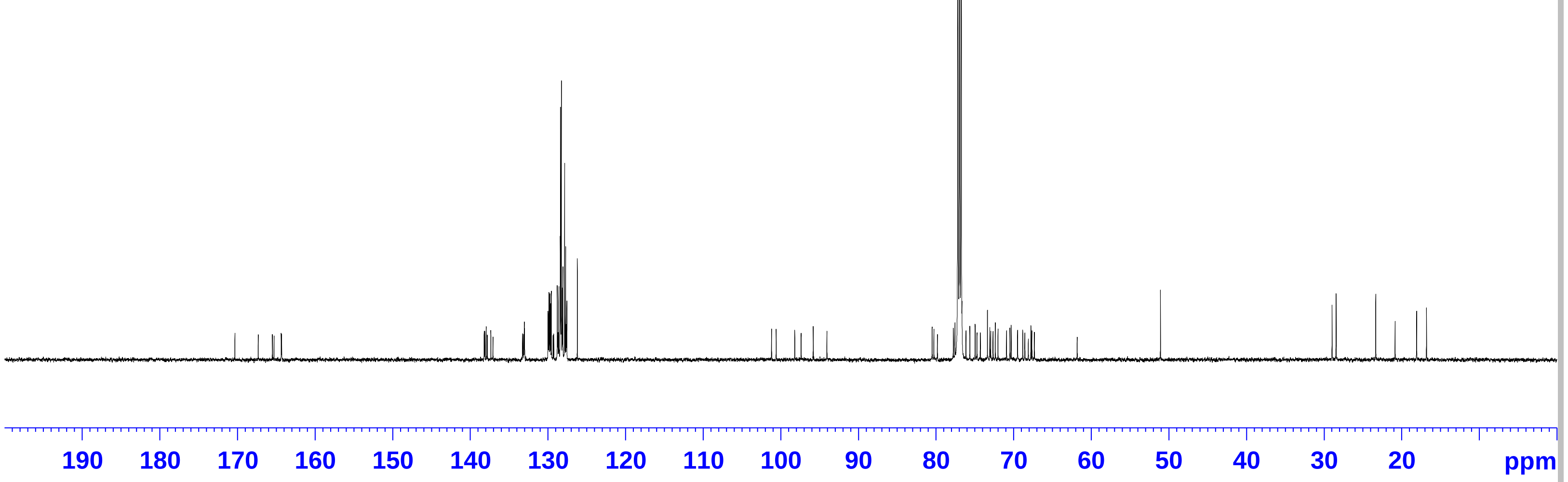

600 MHz ^{1}H NMR Spectrum of Compound **28 polar** (recorded in $CDCl_3$)

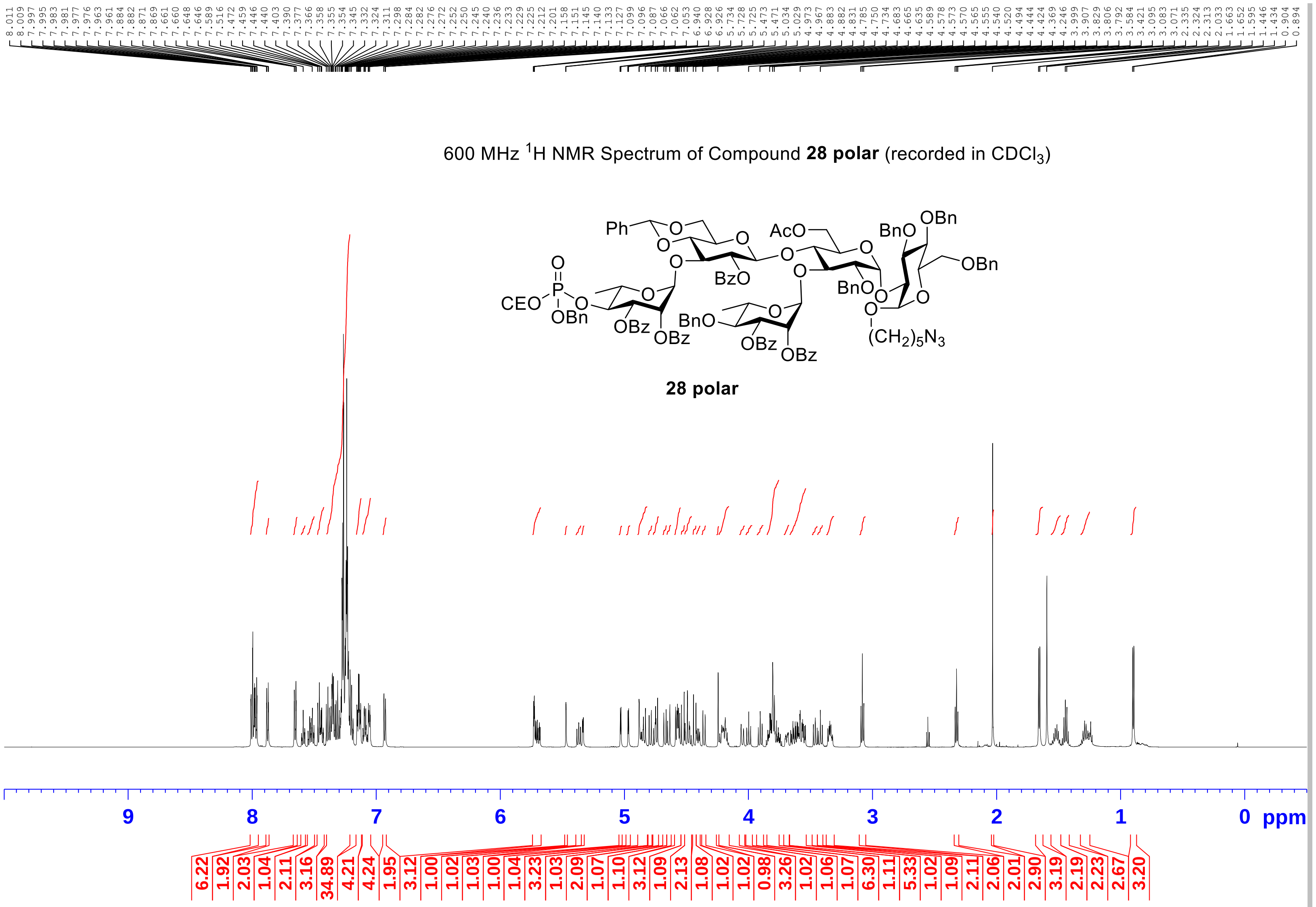

28 polar

150 MHz 13C{1H} NMR Spectrum of Compound **28 polar** (recorded in $CDCl_3$)

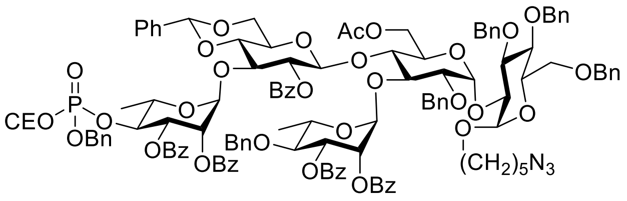

28 polar

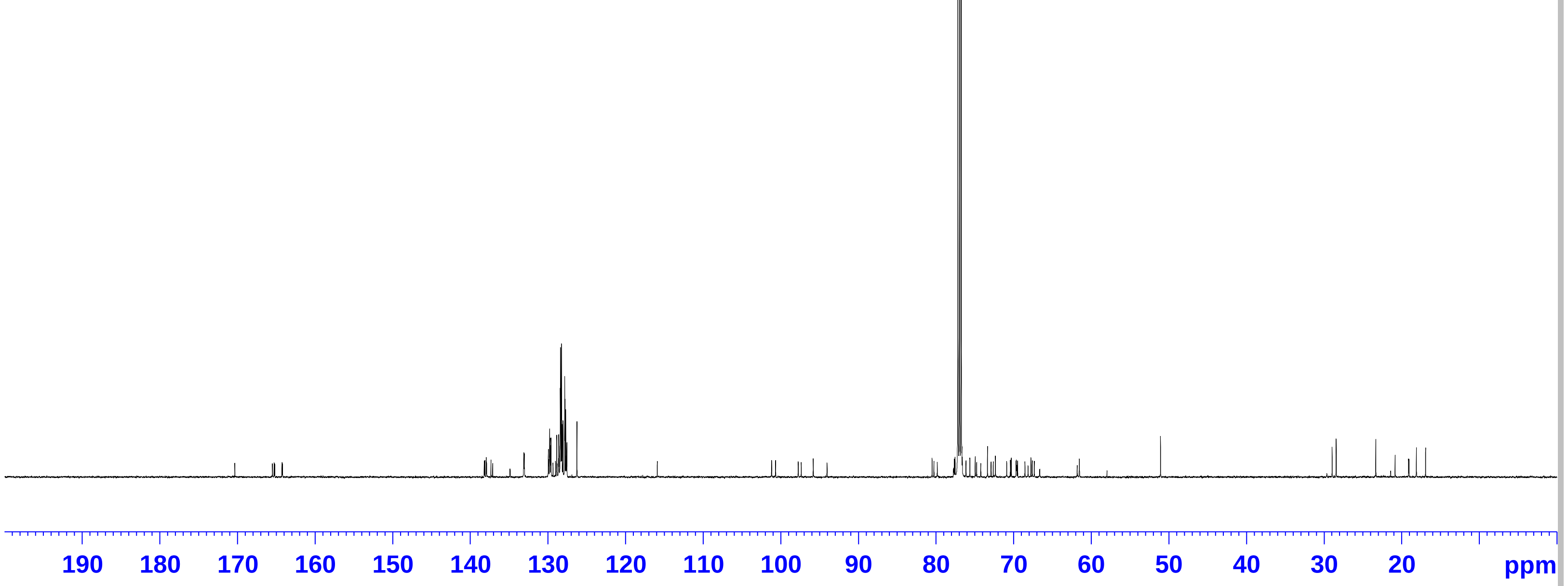

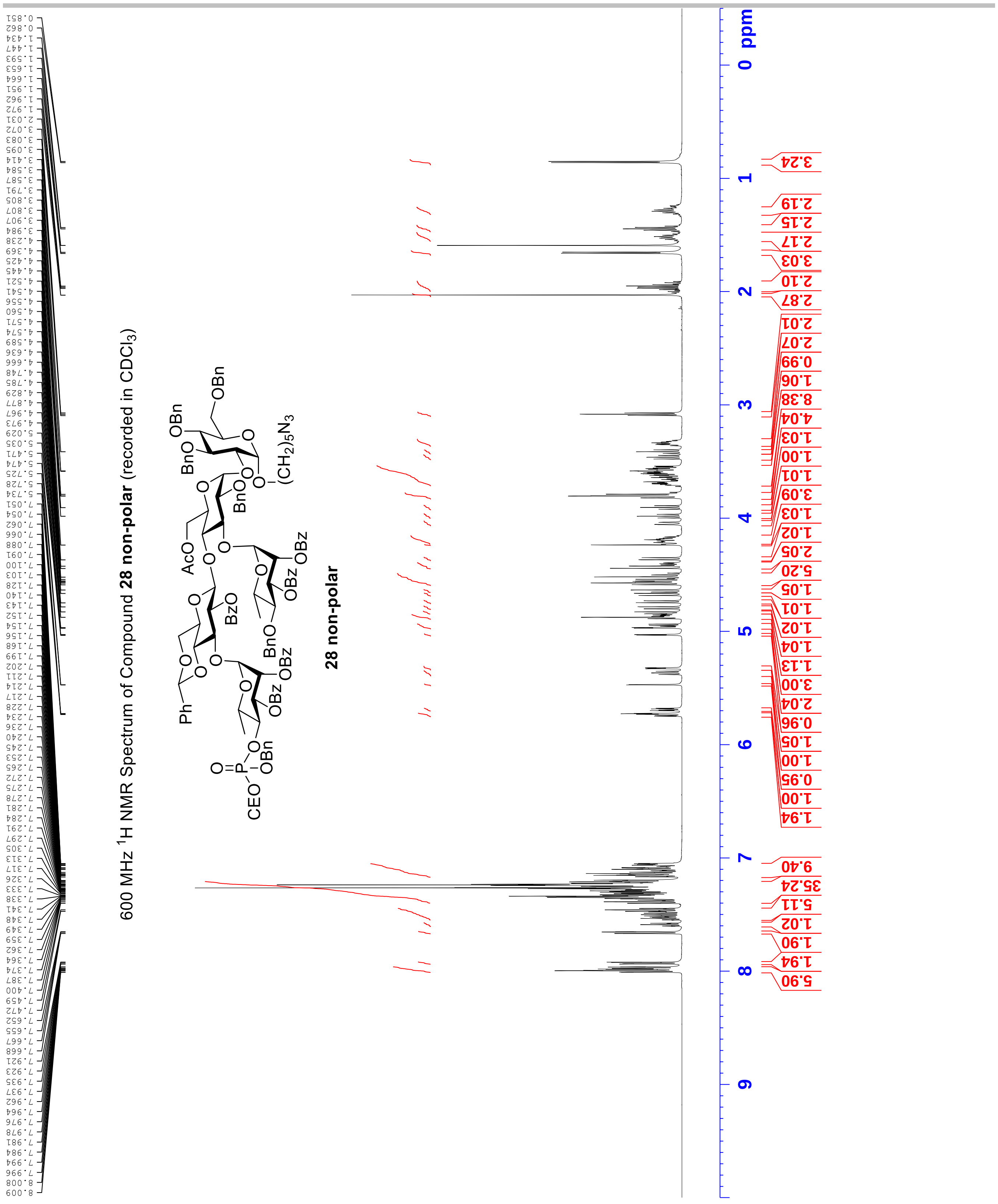
600 MHz ¹H NMR Spectrum of Compound 28 non-polar (recorded in CDCl3)
28 non-polar
ppm

150 MHz 13C{1H} NMR Spectrum of Compound **28 non-polar** (recorded in $CDCl_3$)

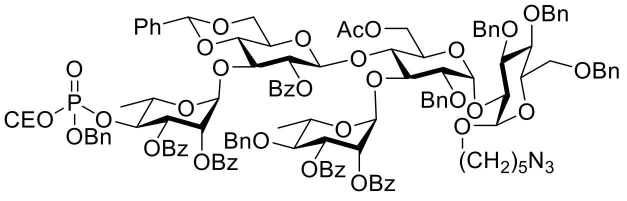

28 non-polar

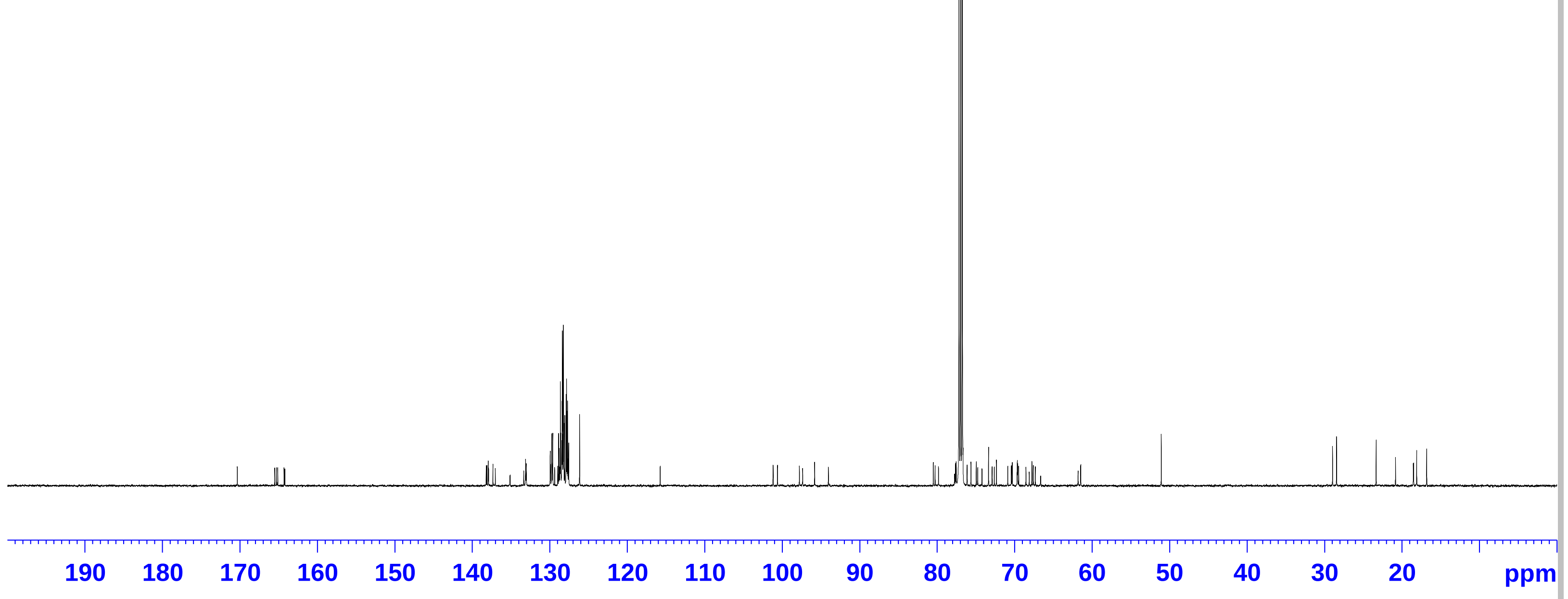

600 MHz ^{1}H NMR Spectrum of Compound **11** (recorded in $CDCl_3$)

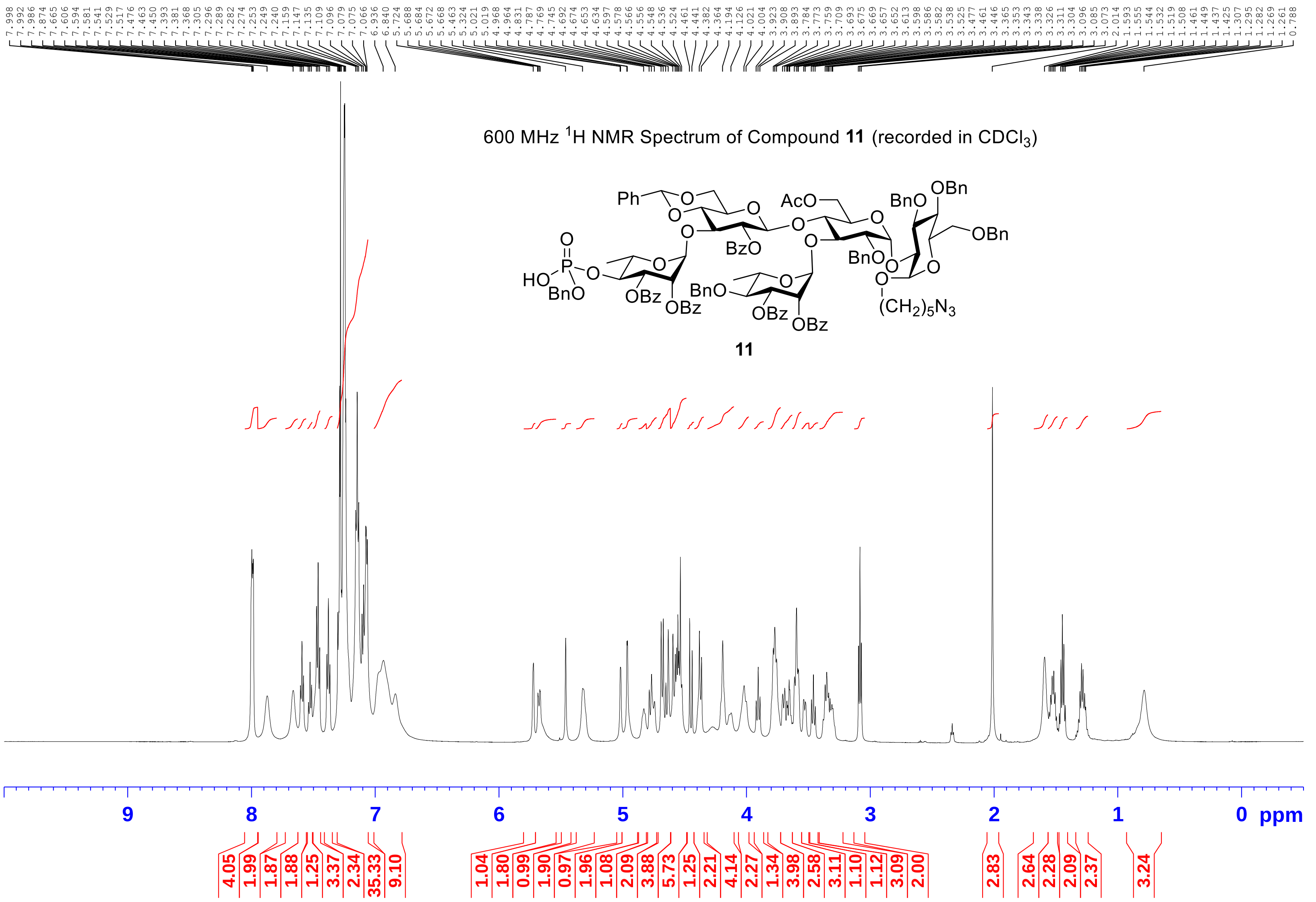

150 MHz 13C{1H} NMR Spectrum of Compound **11** (recorded in $CDCl_3$)

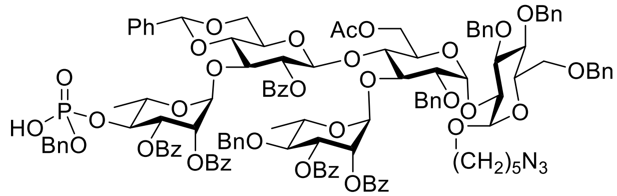

11

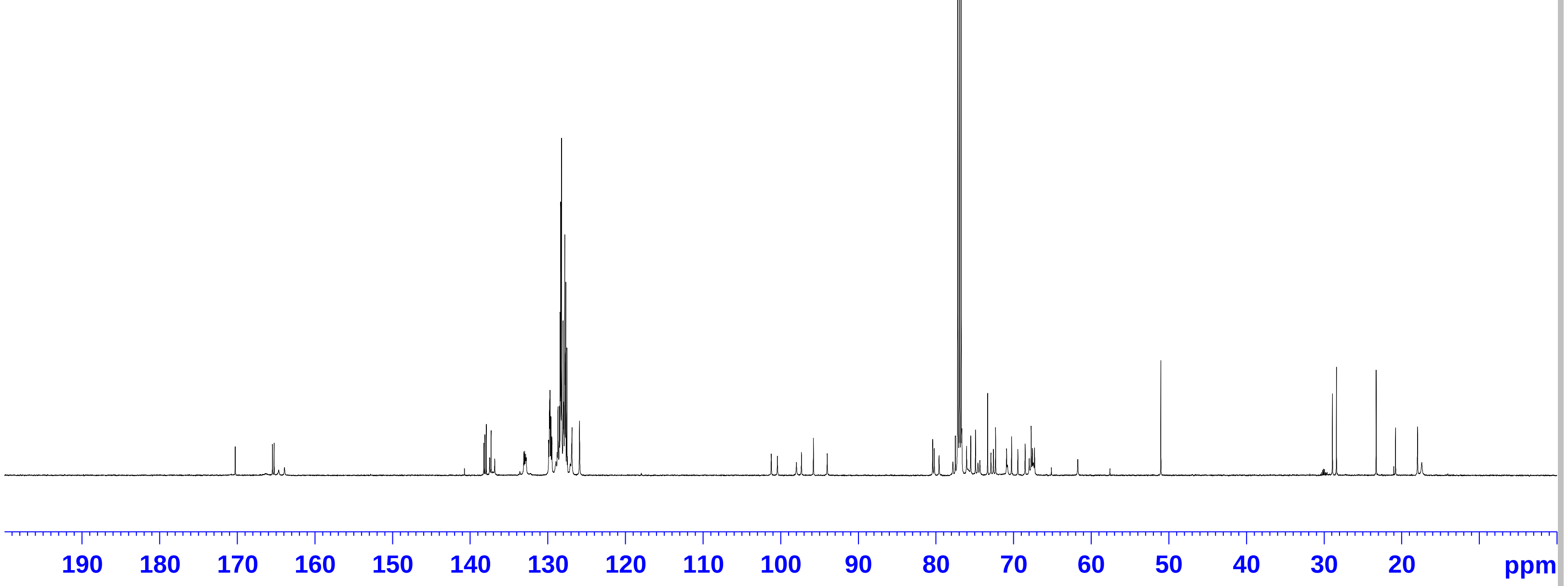

600 MHz ^{1}H NMR Spectrum of Compound **29 alpha polar** (recorded in $CDCl_3$)

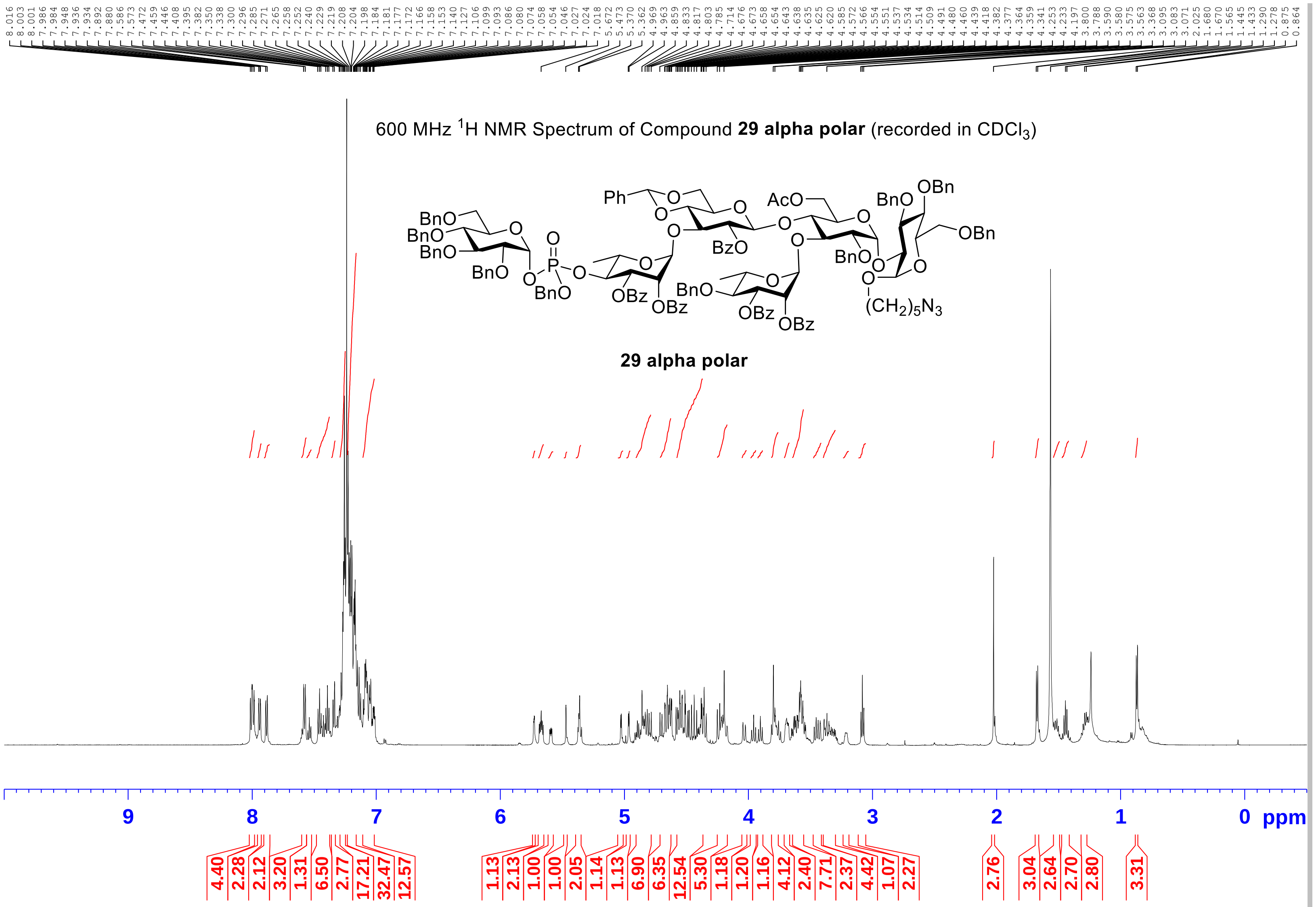

150 MHz 13C{1H} NMR Spectrum of Compound **29 alpha polar** (recorded in $CDCl_3$)

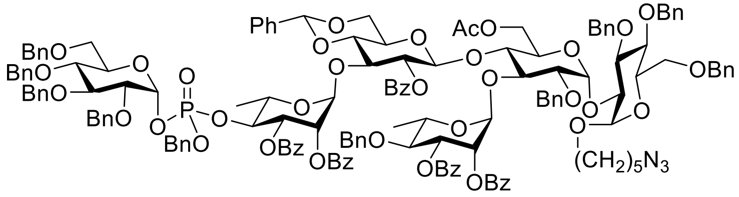

29 alpha polar

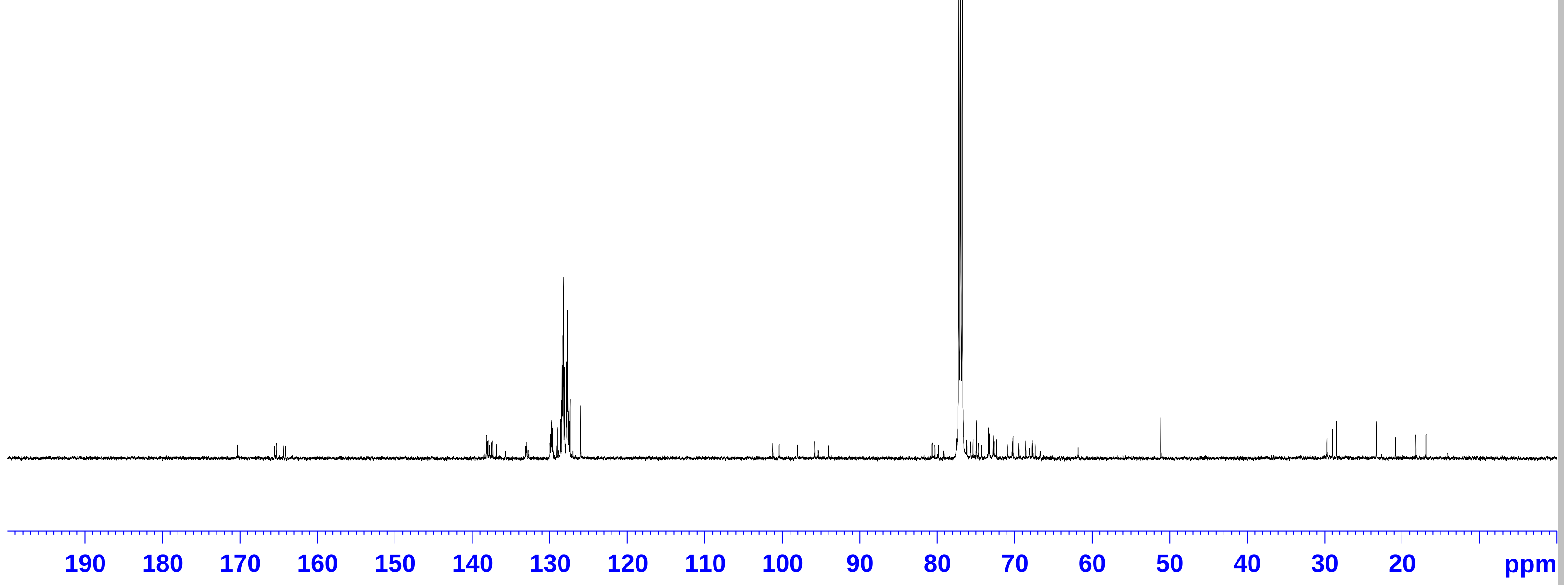

600 MHz ^{1}H NMR Spectrum of Compound **29 alpha non-polar** (recorded in $CDCl_3$)

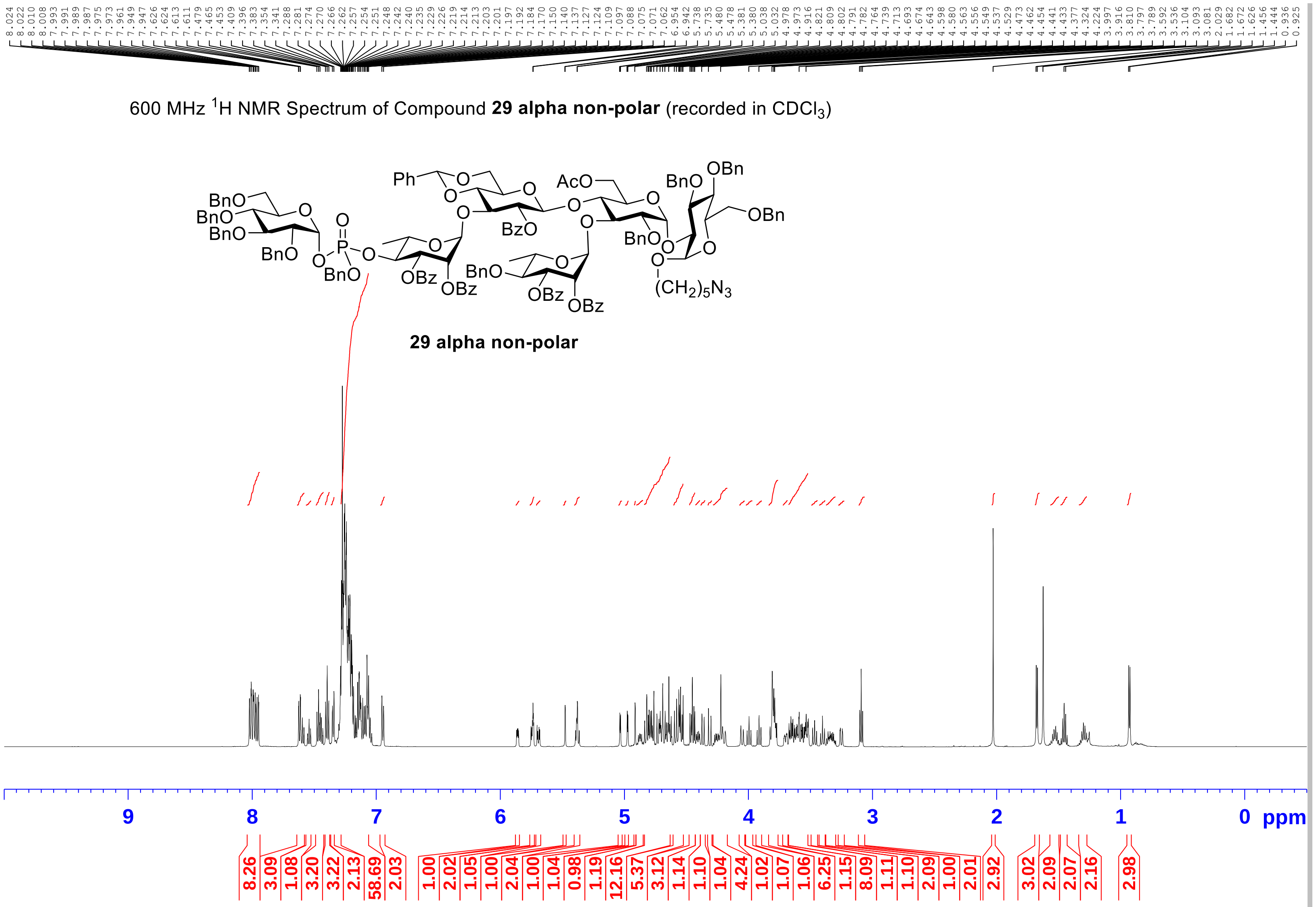

29 alpha non-polar

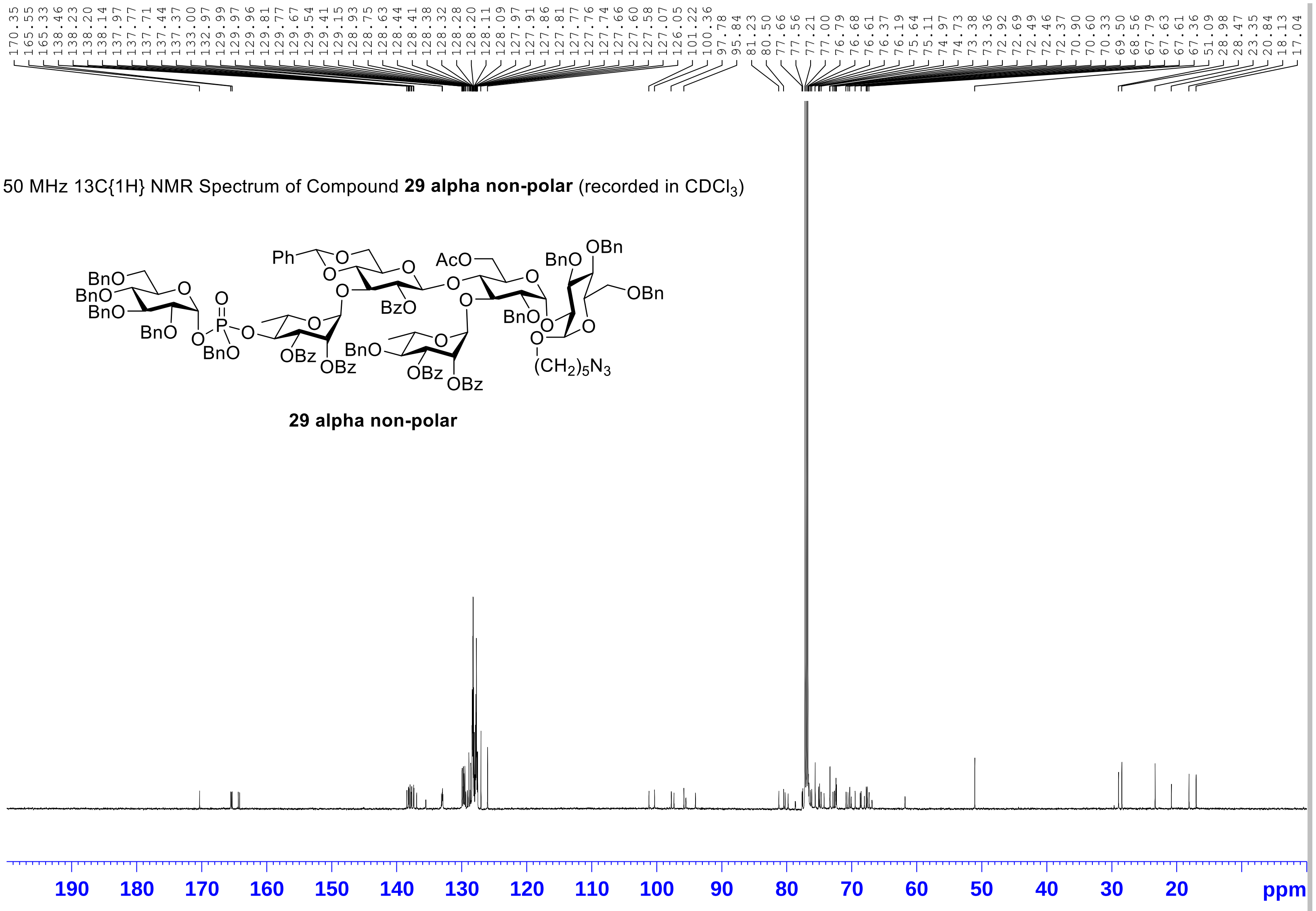

150 MHz 13C{1H} NMR Spectrum of Compound **29 alpha non-polar** (recorded in $CDCl_3$)

29 alpha non-polar

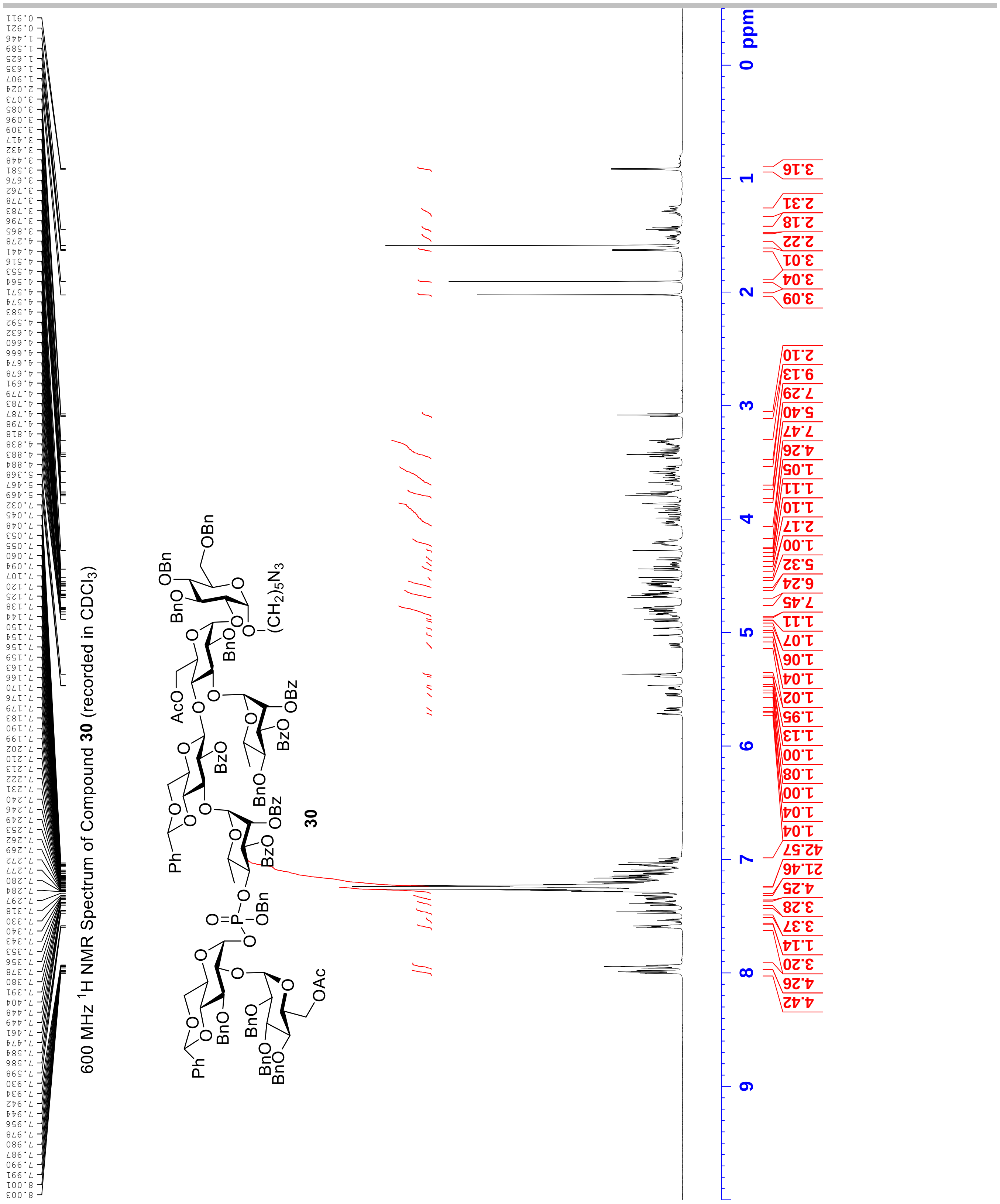

600 MHz ^{1}H NMR Spectrum of Compound **30** (recorded in $CDCl_3$)

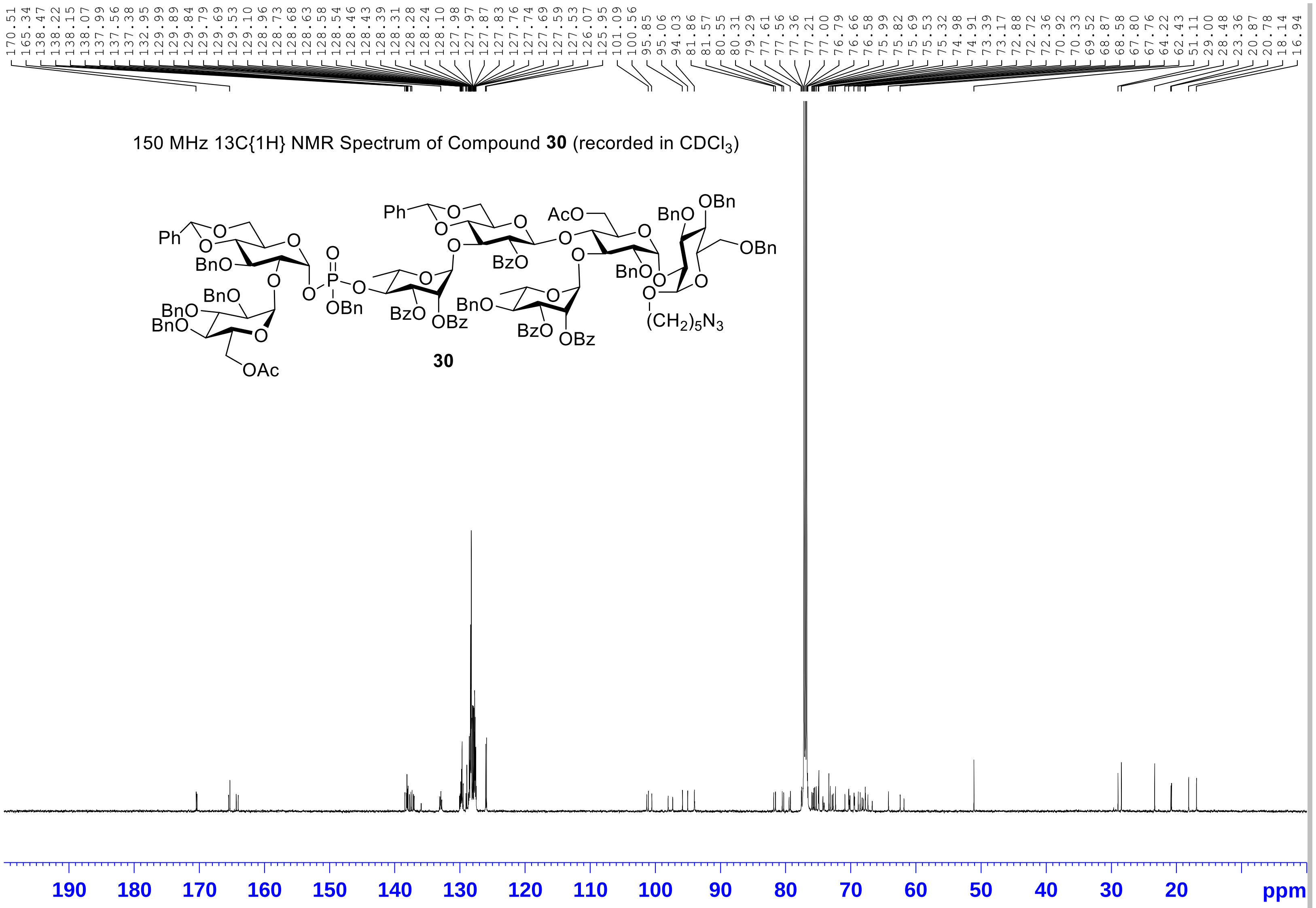

150 MHz 13C{1H} NMR Spectrum of Compound **30** (recorded in $CDCl_3$)

30

600 MHz ^{1}H NMR Spectrum of Compound **27b** (recorded in $CDCl_3$)

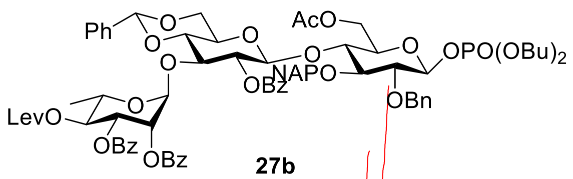

27b

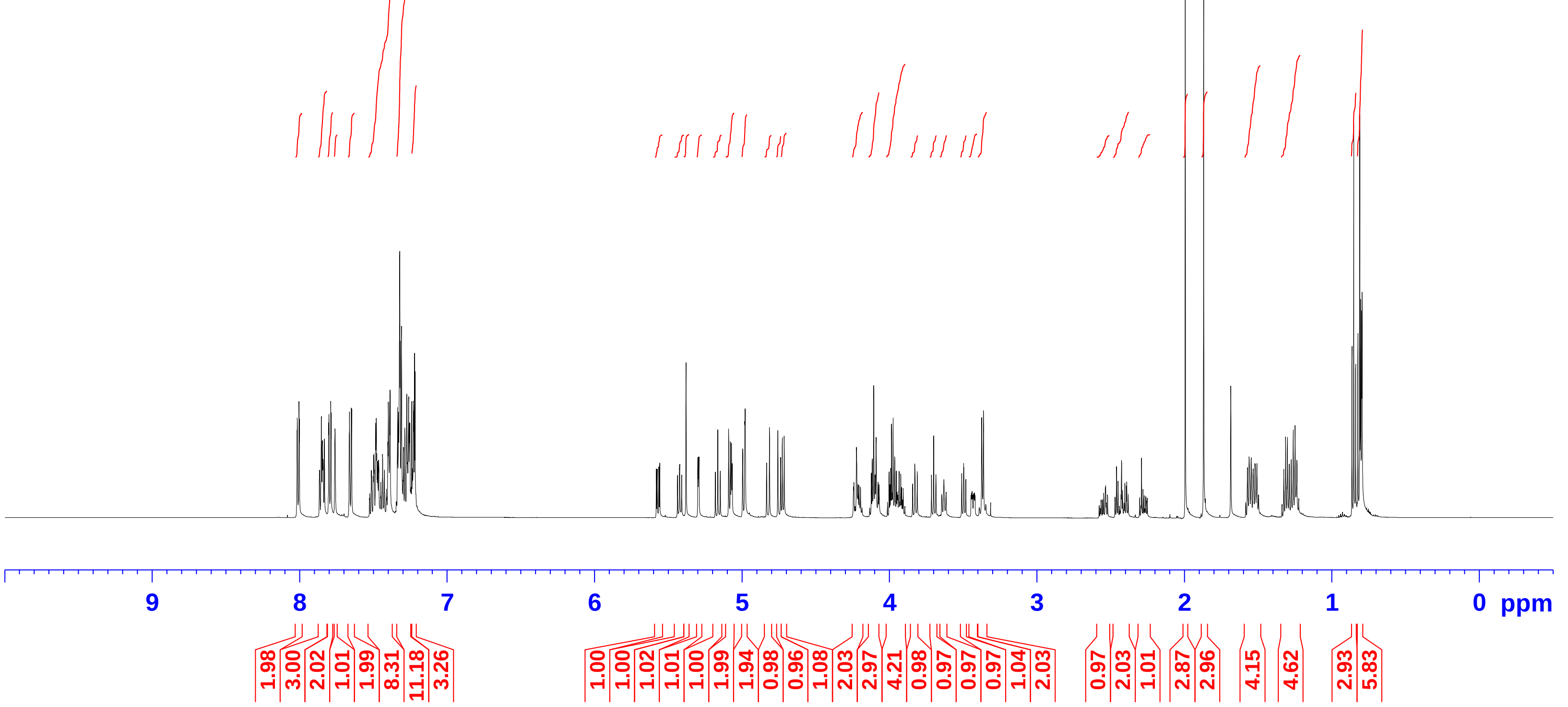

150 MHz 13C{1H} NMR Spectrum of Compound **27b** (recorded in $CDCl_3$)

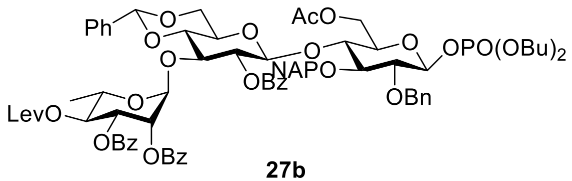

27b

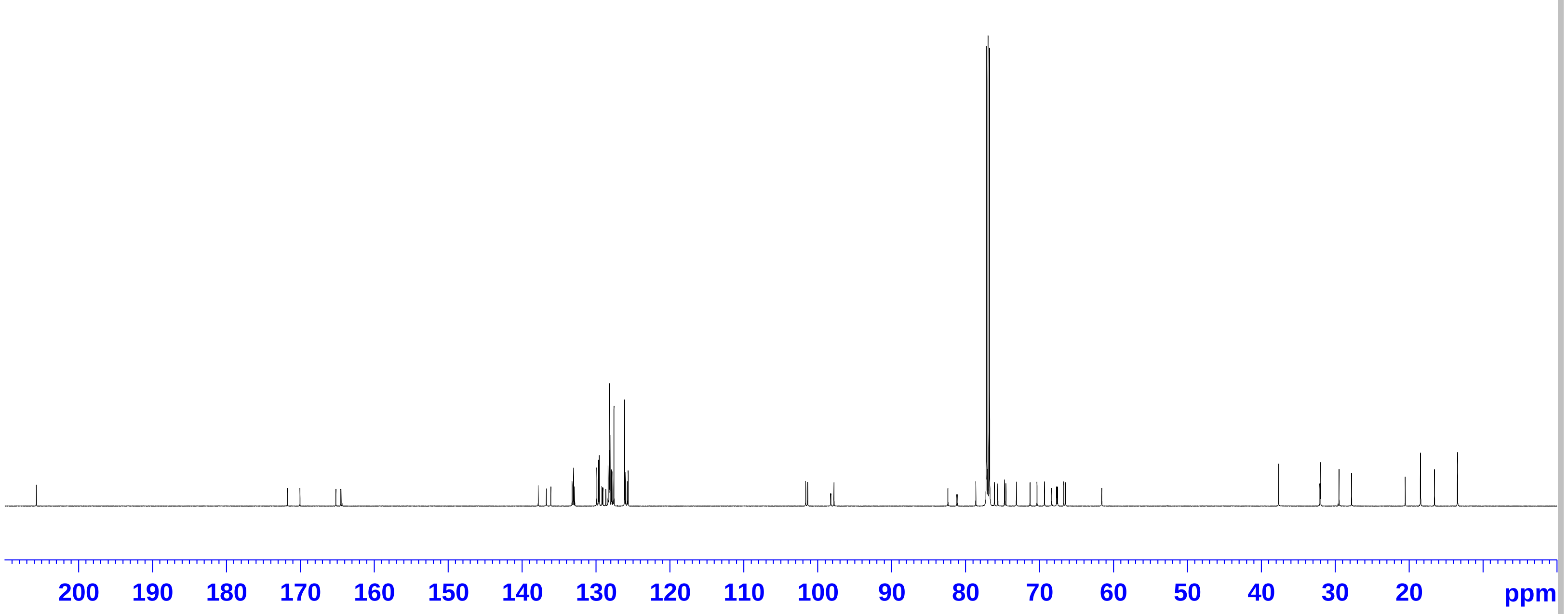

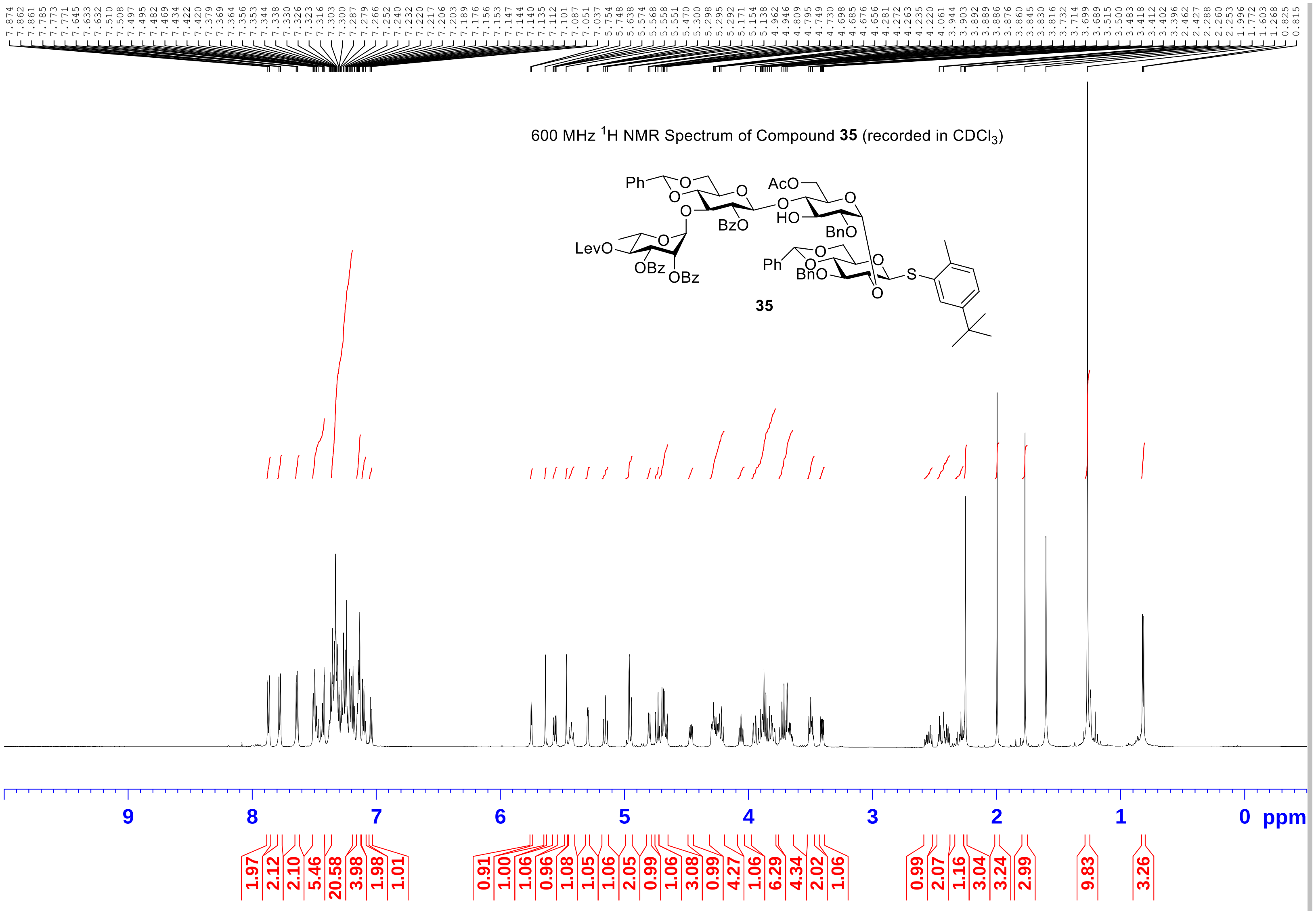

600 MHz ^{1}H NMR Spectrum of Compound **35** (recorded in $CDCl_3$)

35

150 MHz 13C{1H} NMR Spectrum of Compound **35** (recorded in $CDCl_3$)

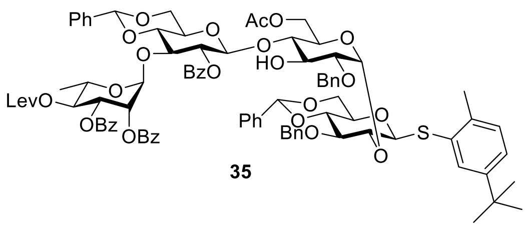

35

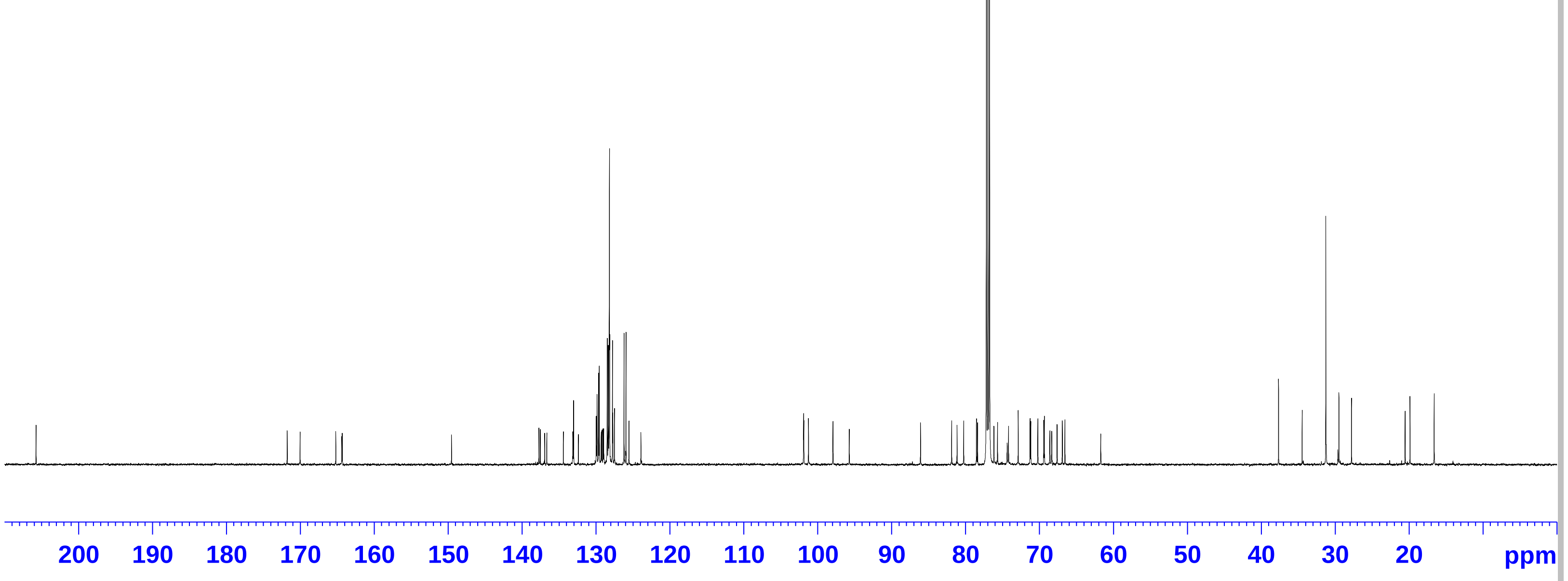

600 MHz ^{1}H NMR Spectrum of Compound **12b** (recorded in $CDCl_3$)

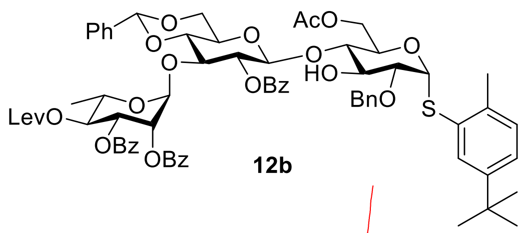

12b

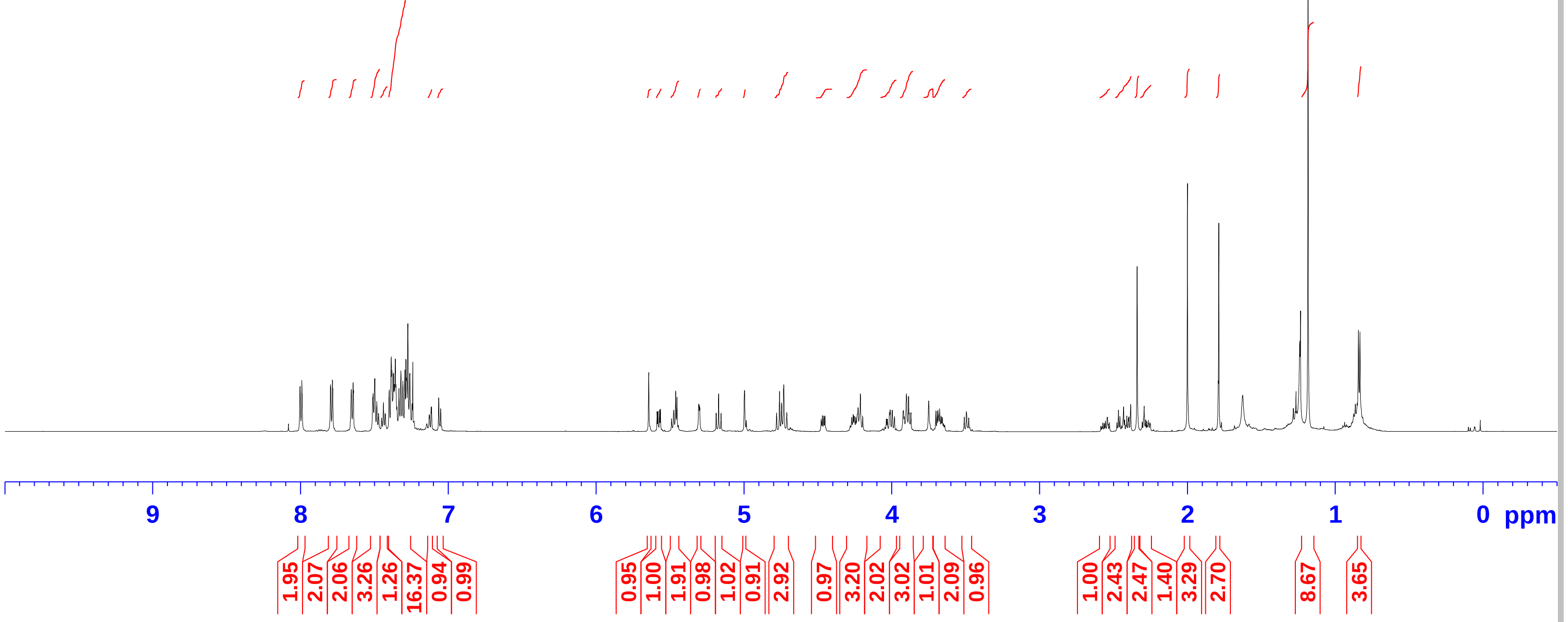

150 MHz 13C{1H} NMR Spectrum of Compound **12b** (recorded in $CDCl_3$)

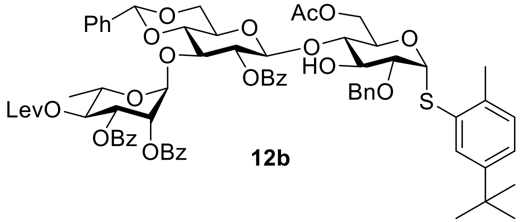

12b

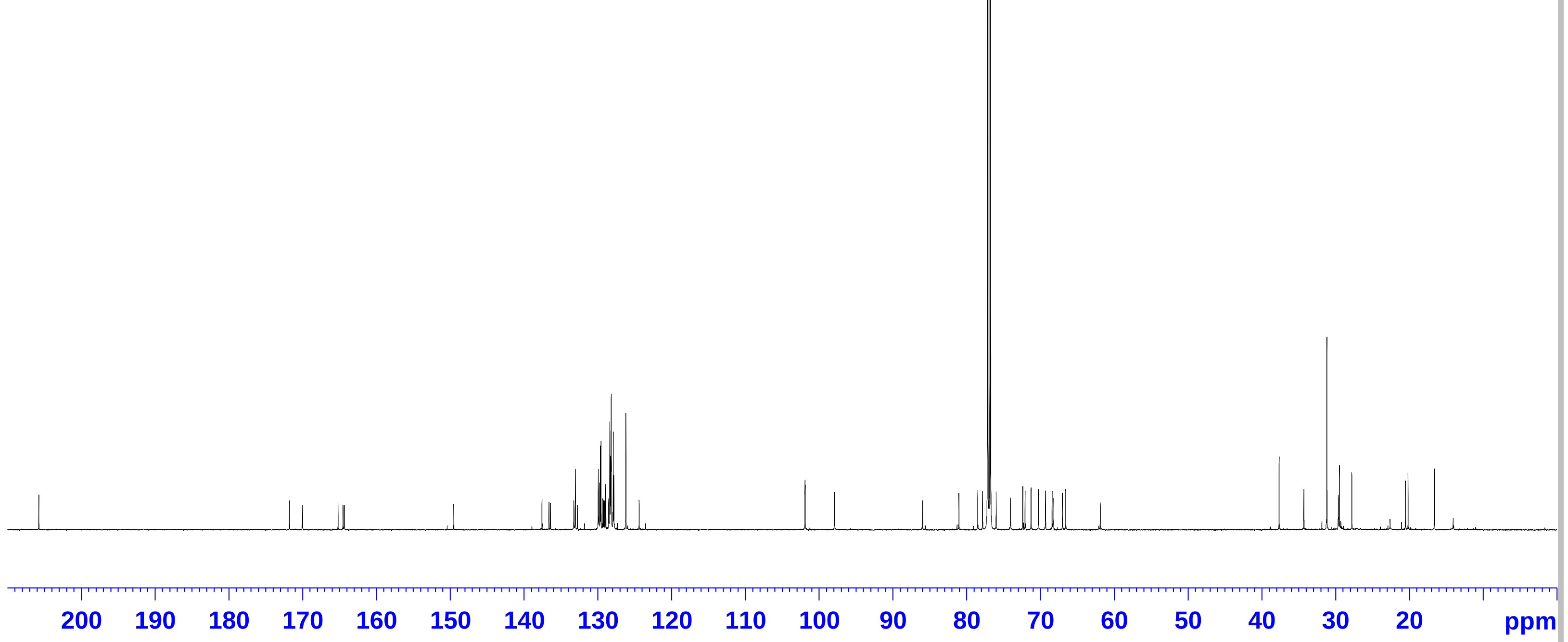

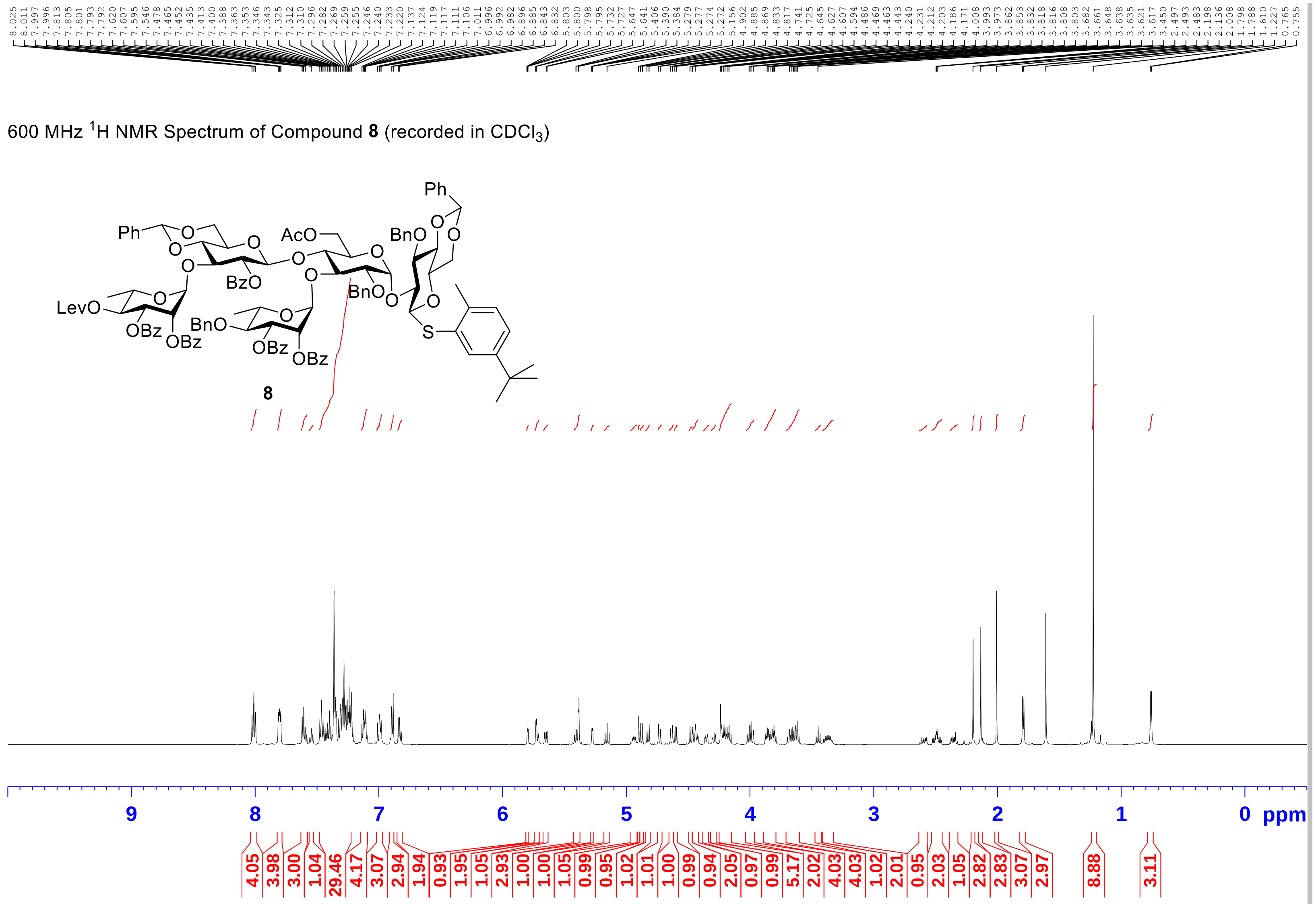

600 MHz ^{1}H NMR Spectrum of Compound **8** (recorded in $CDCl_3$)

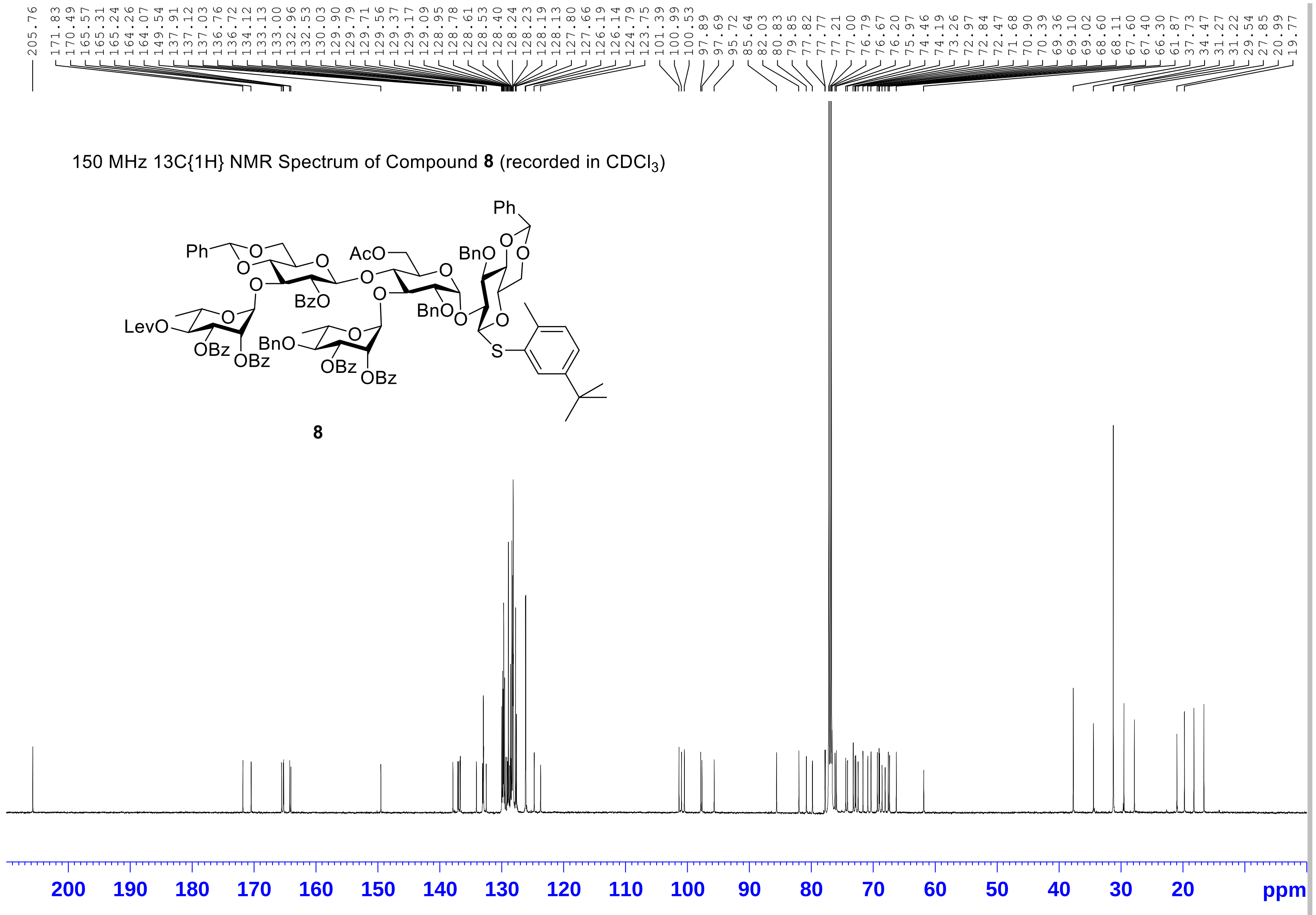

150 MHz 13C{1H} NMR Spectrum of Compound **8** (recorded in $CDCl_3$)

600 MHz ^{1}H NMR Spectrum of Compound **36** (recorded in $CDCl_3$)

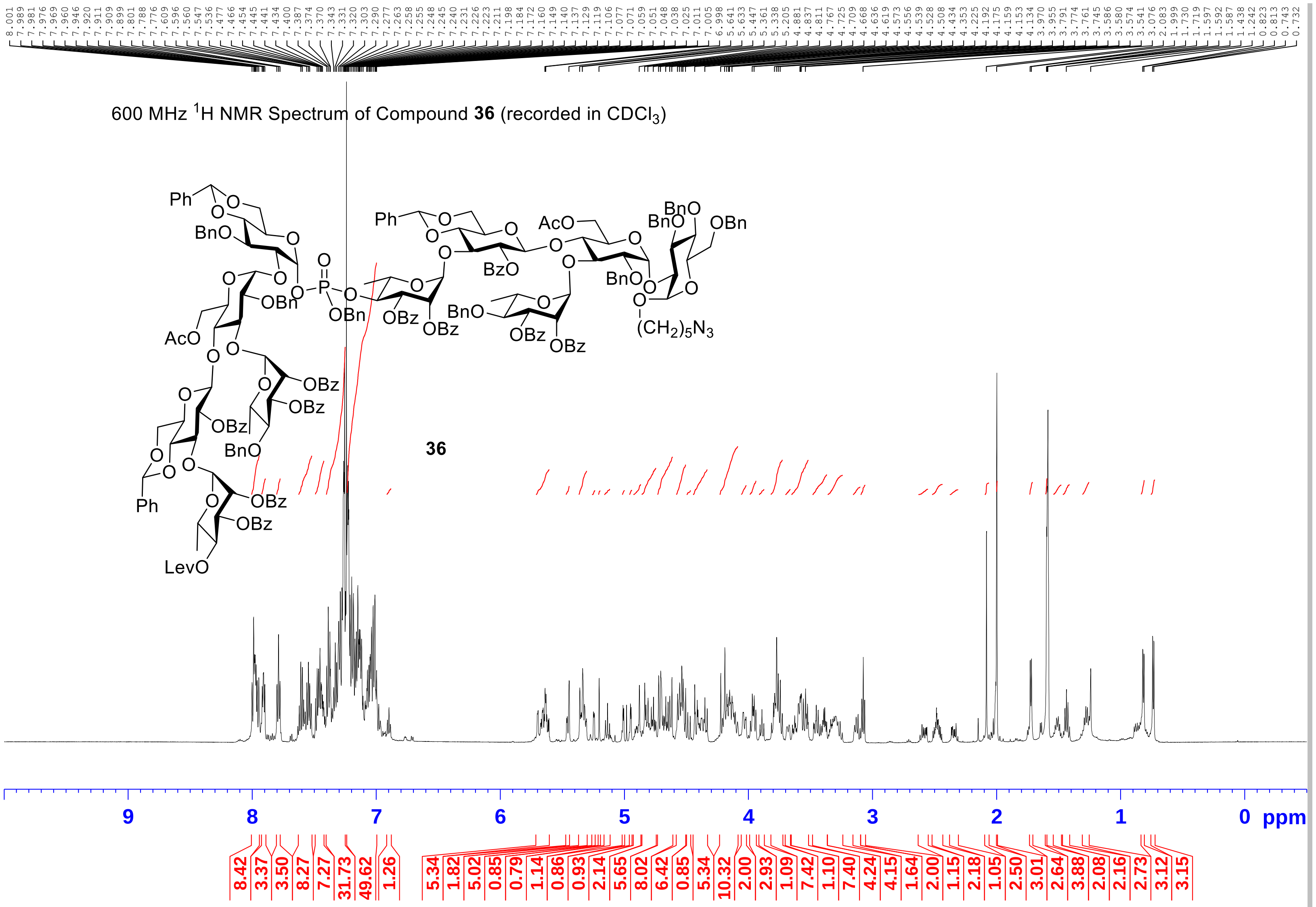

150 MHz 13C{1H} NMR Spectrum of Compound **36** (recorded in $CDCl_3$)

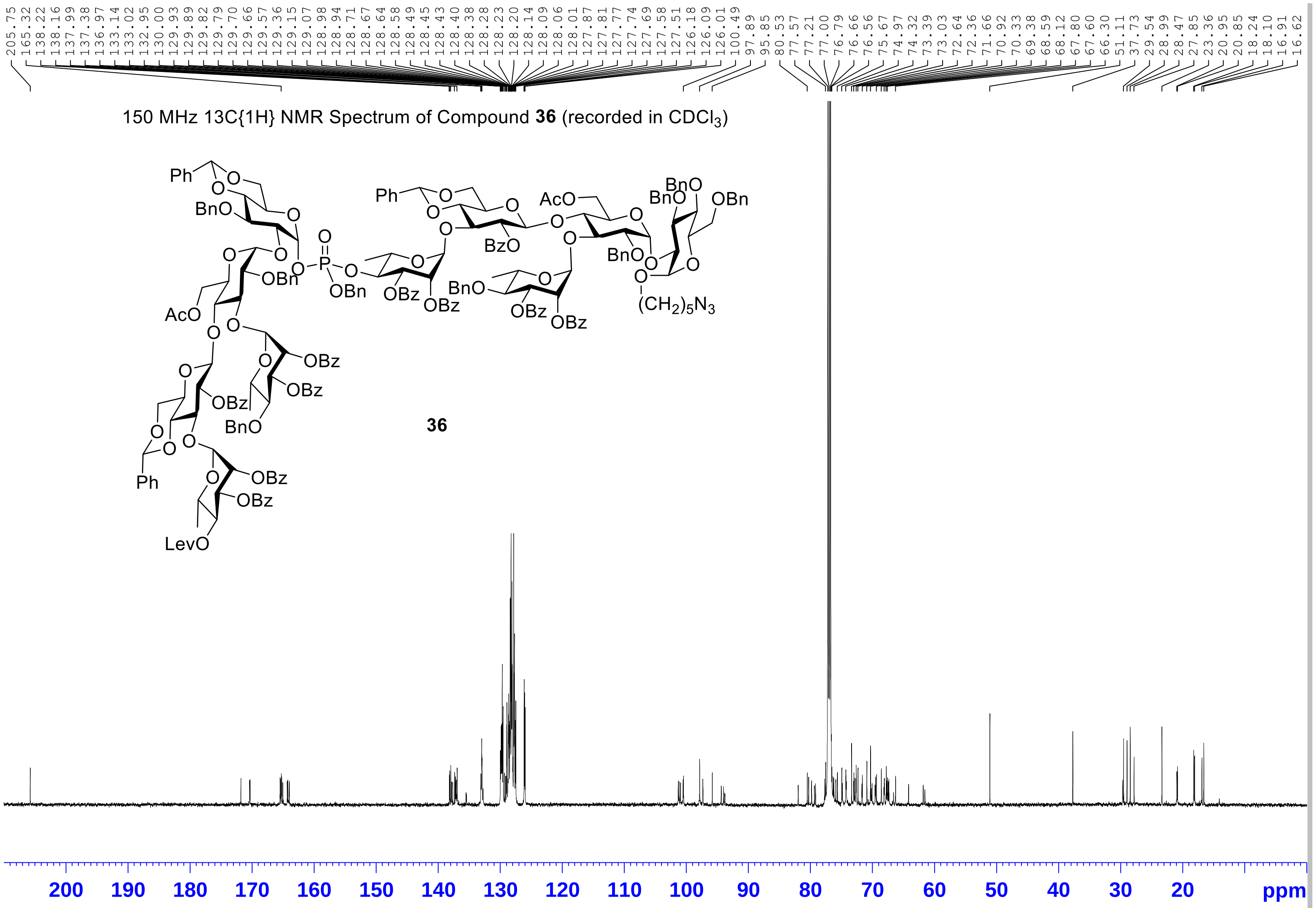

36

600 MHz ^{1}H NMR Spectrum of Compound **38** (recorded in $CDCl_3$)

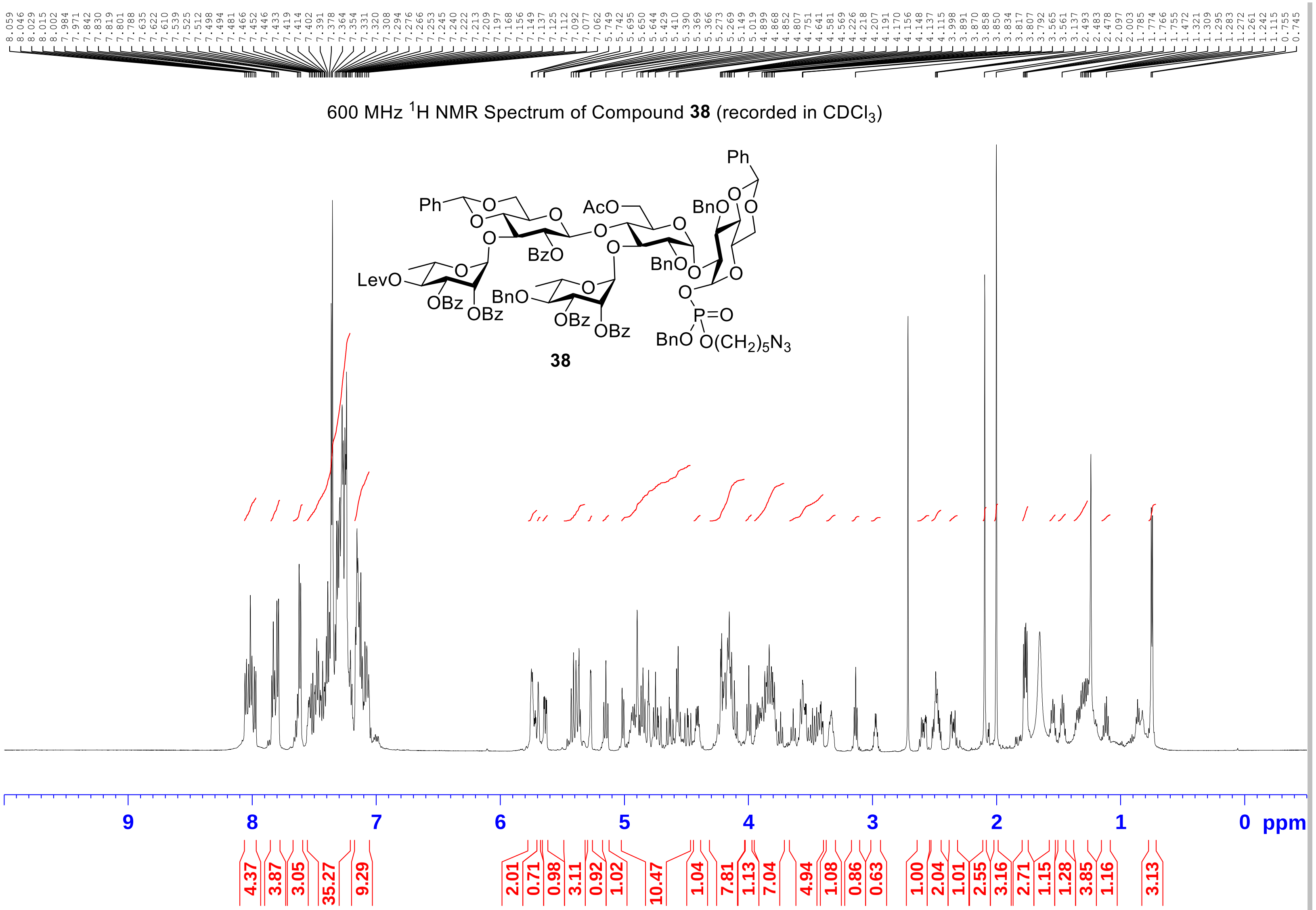

150 MHz 13C{1H} NMR Spectrum of Compound **38** (recorded in $CDCl_3$)

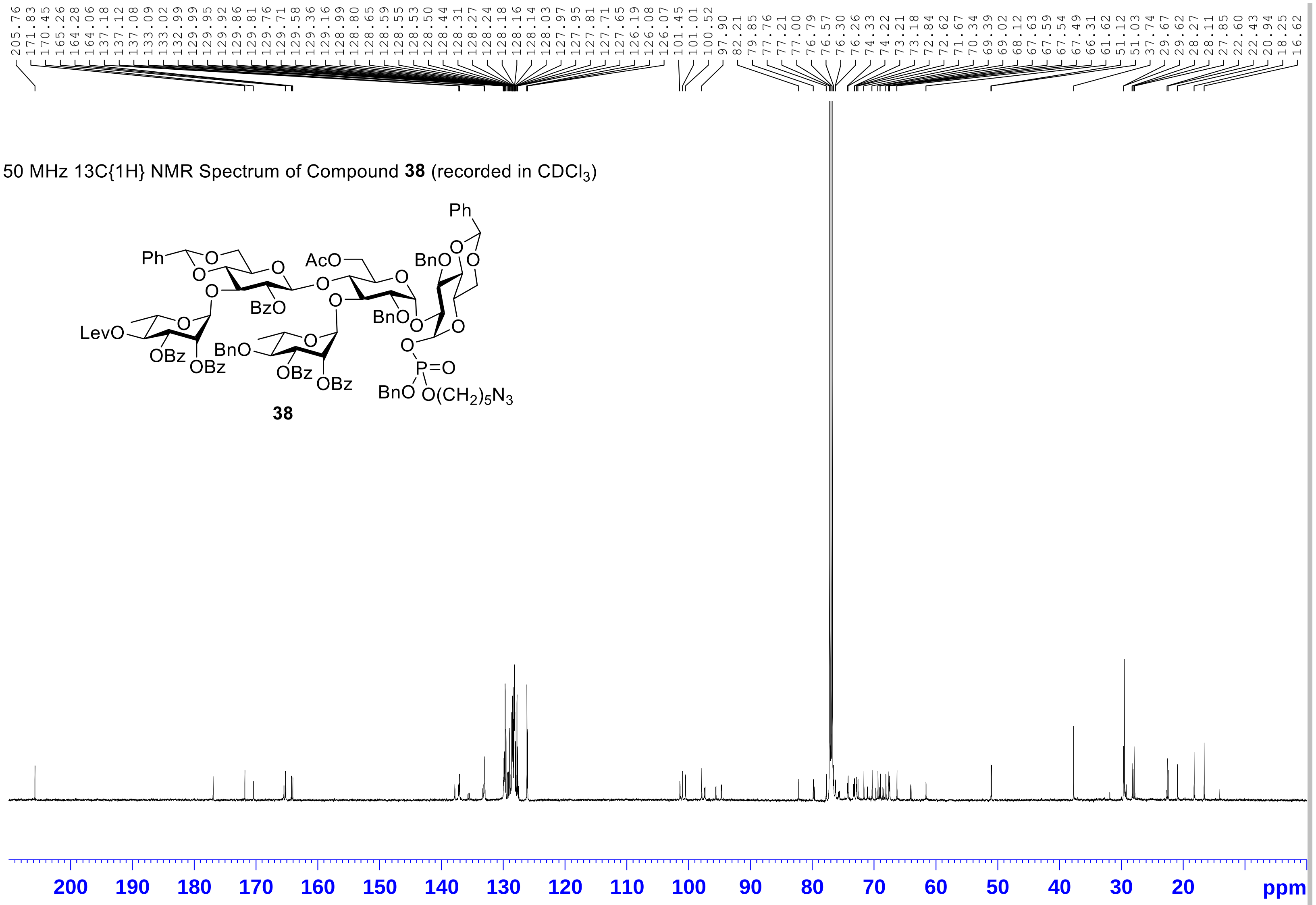

38

600 MHz ^{1}H NMR Spectrum of Compound **39** (recorded in $CDCl_3$)

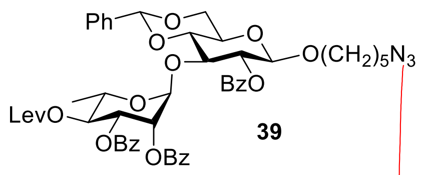

39

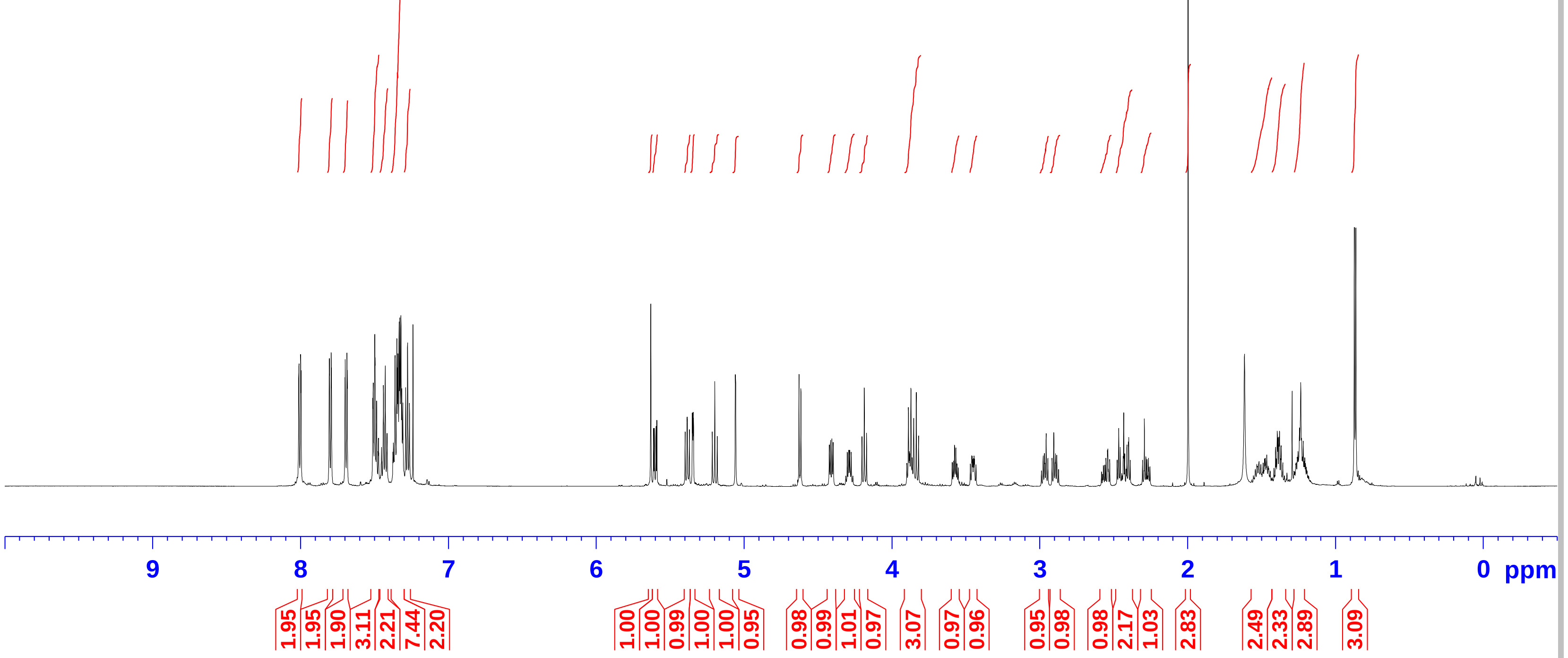

150 MHz 13C{1H} NMR Spectrum of Compound **39** (recorded in $CDCl_3$)

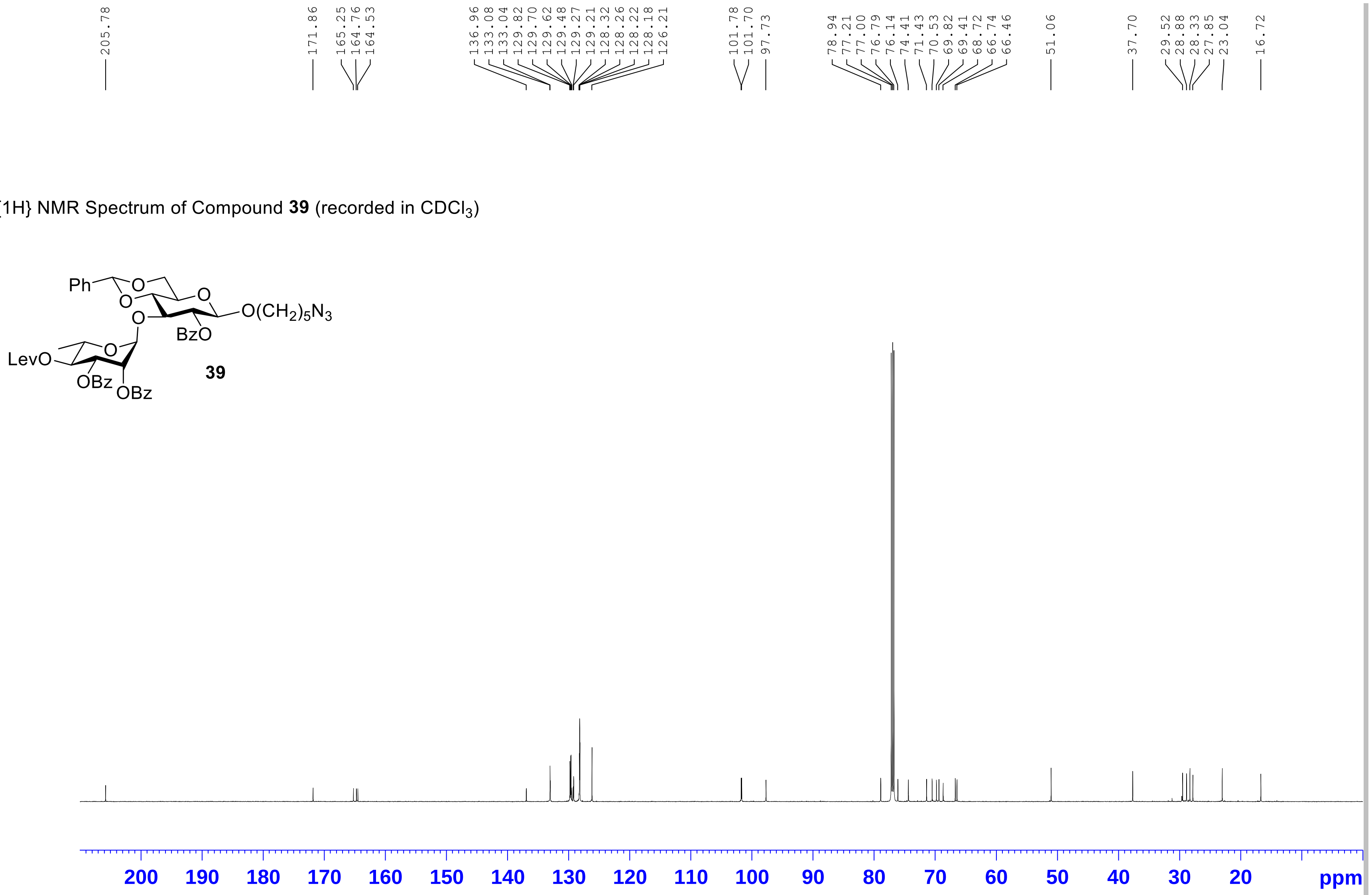

600 MHz ^1H NMR Spectrum of Compound **40** (recorded in $CDCl_3$)

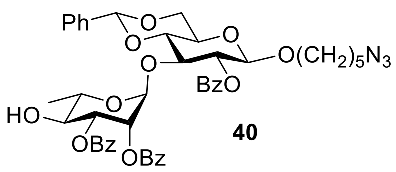

40

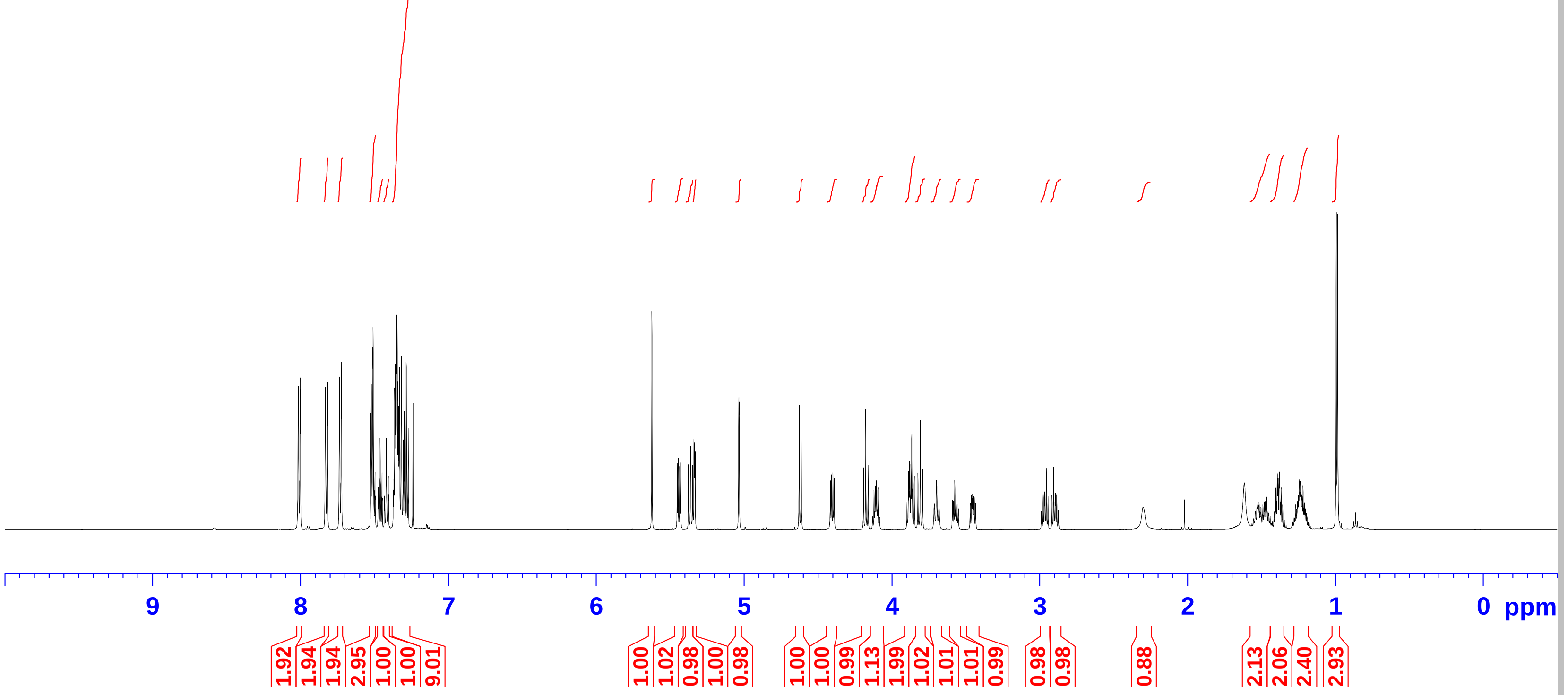

150 MHz 13C{1H} NMR Spectrum of Compound **40** (recorded in $CDCl_3$)

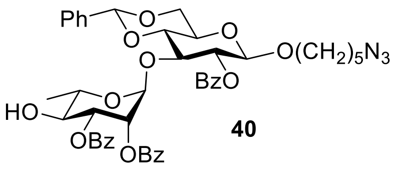

40

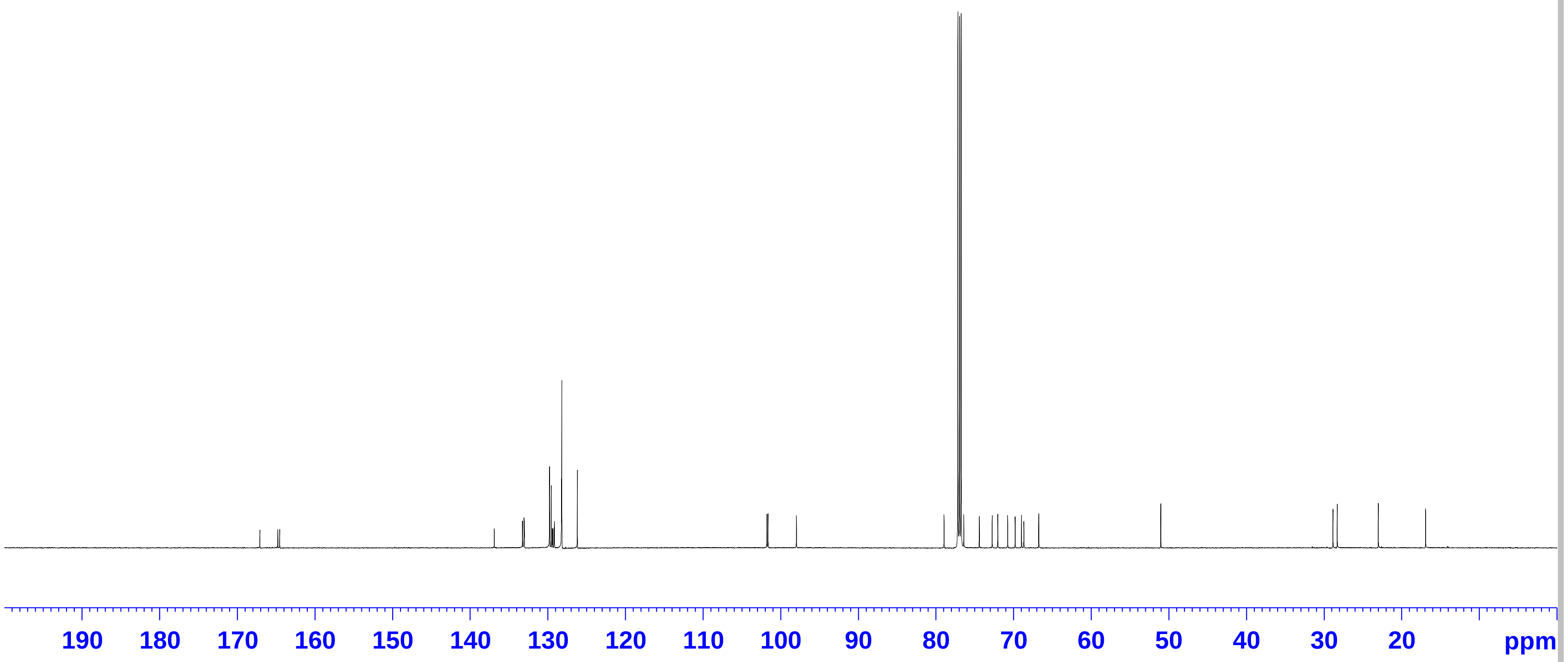

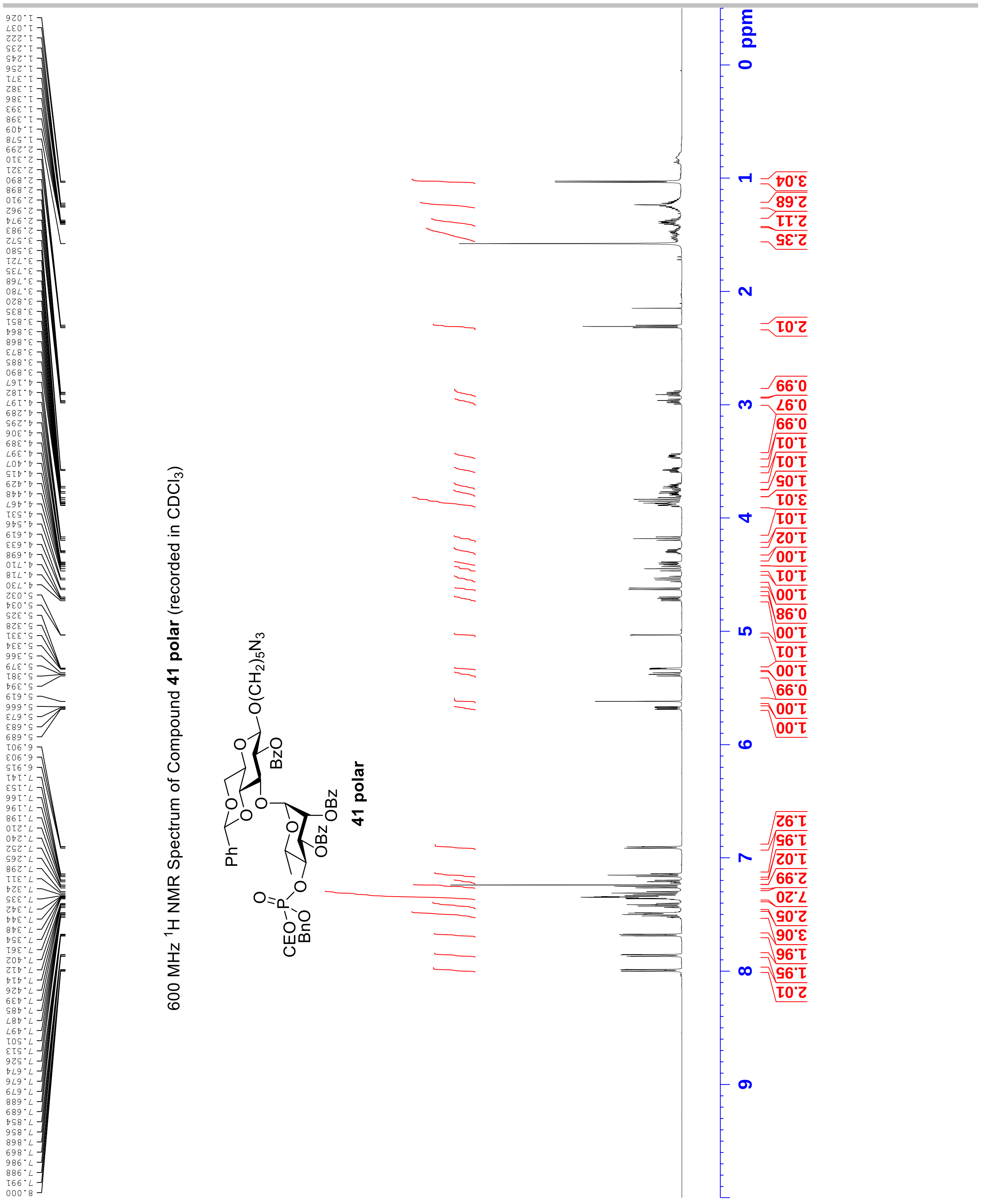

600 MHz ^{1}H NMR Spectrum of Compound **41 polar** (recorded in $CDCl_3$)

150 MHz 13C{1H} NMR Spectrum of Compound **41 polar** (recorded in $CDCl_3$)

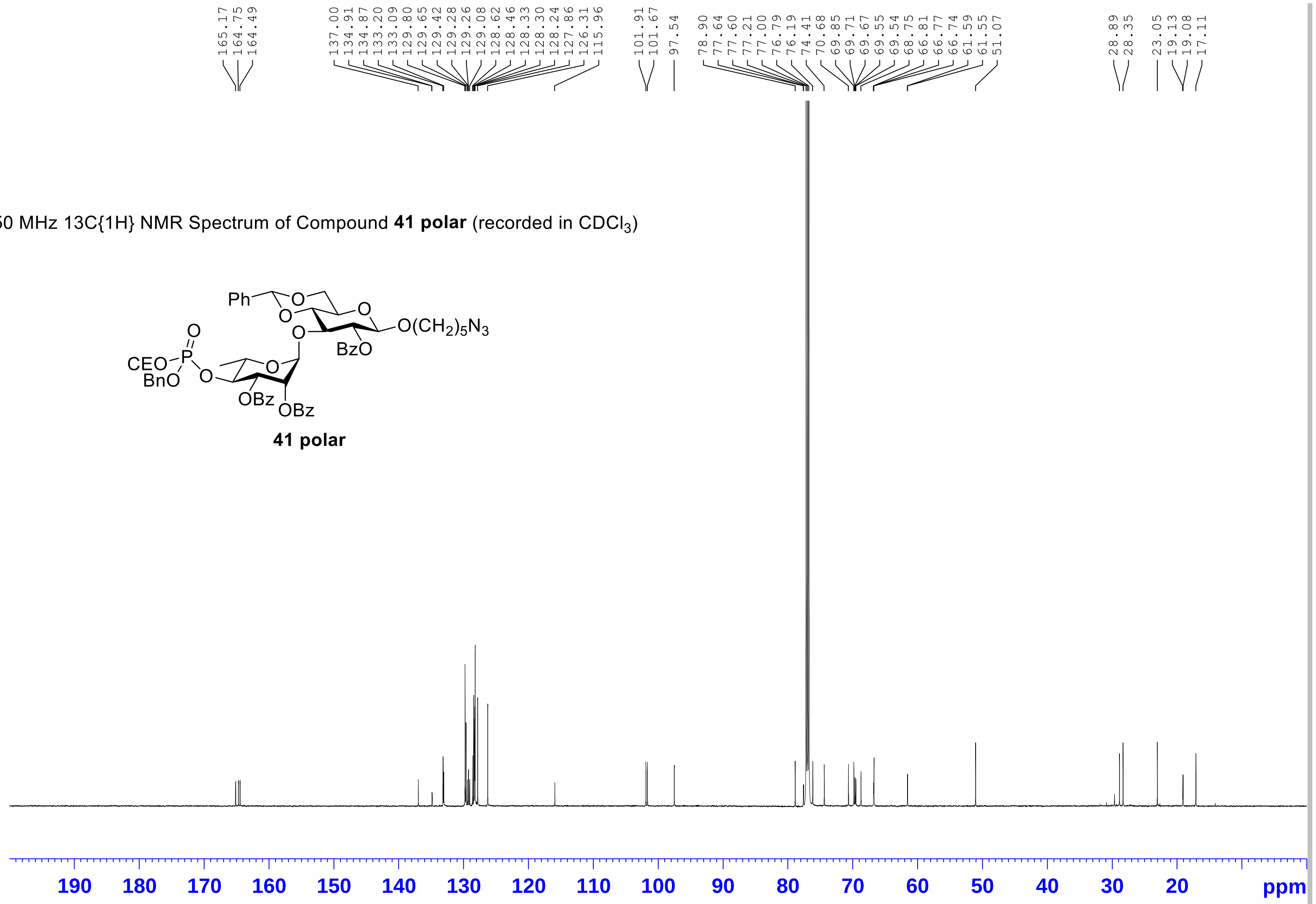

41 polar

600 MHz ^{1}H NMR Spectrum of Compound **41 non-polar** (recorded in $CDCl_3$)

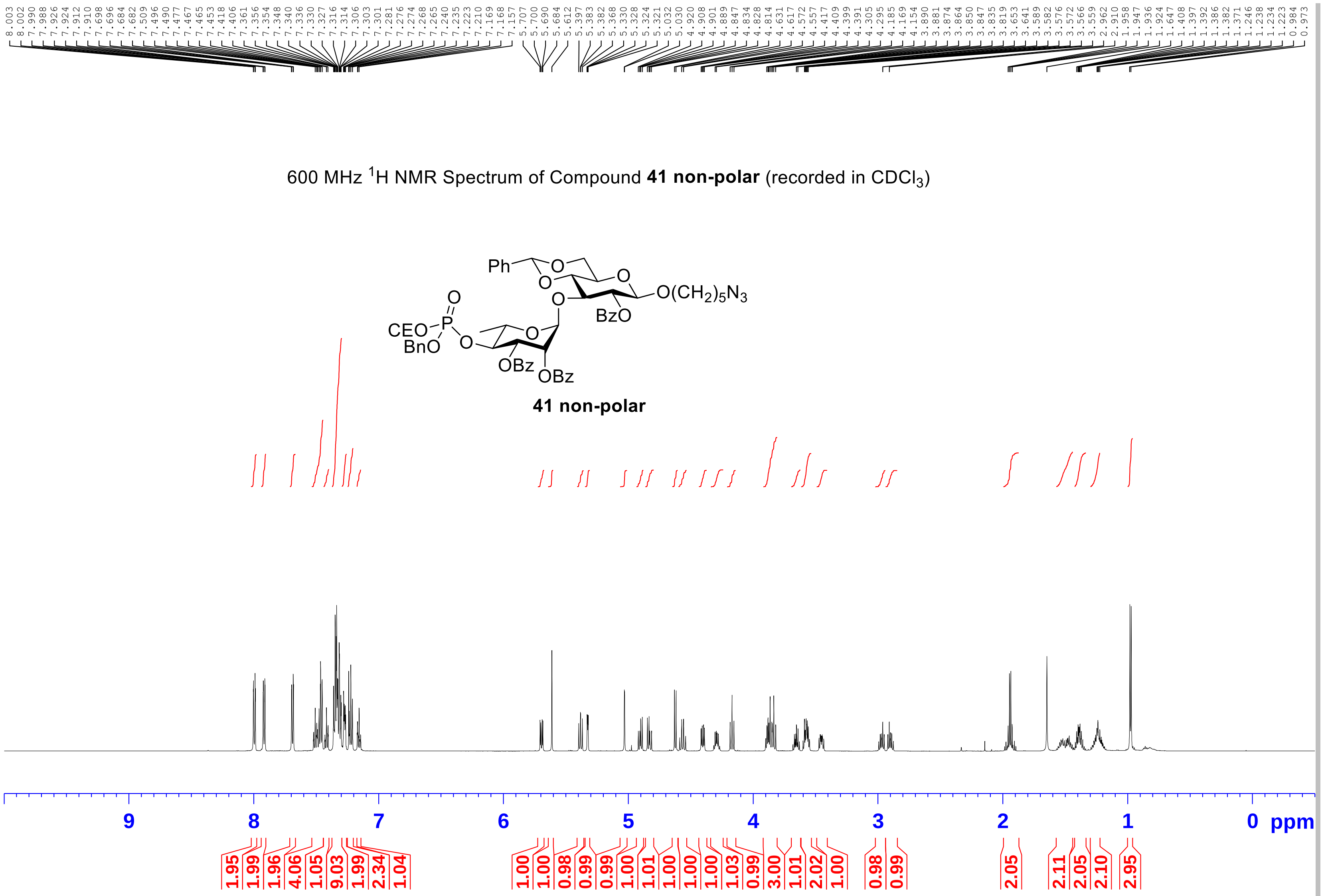

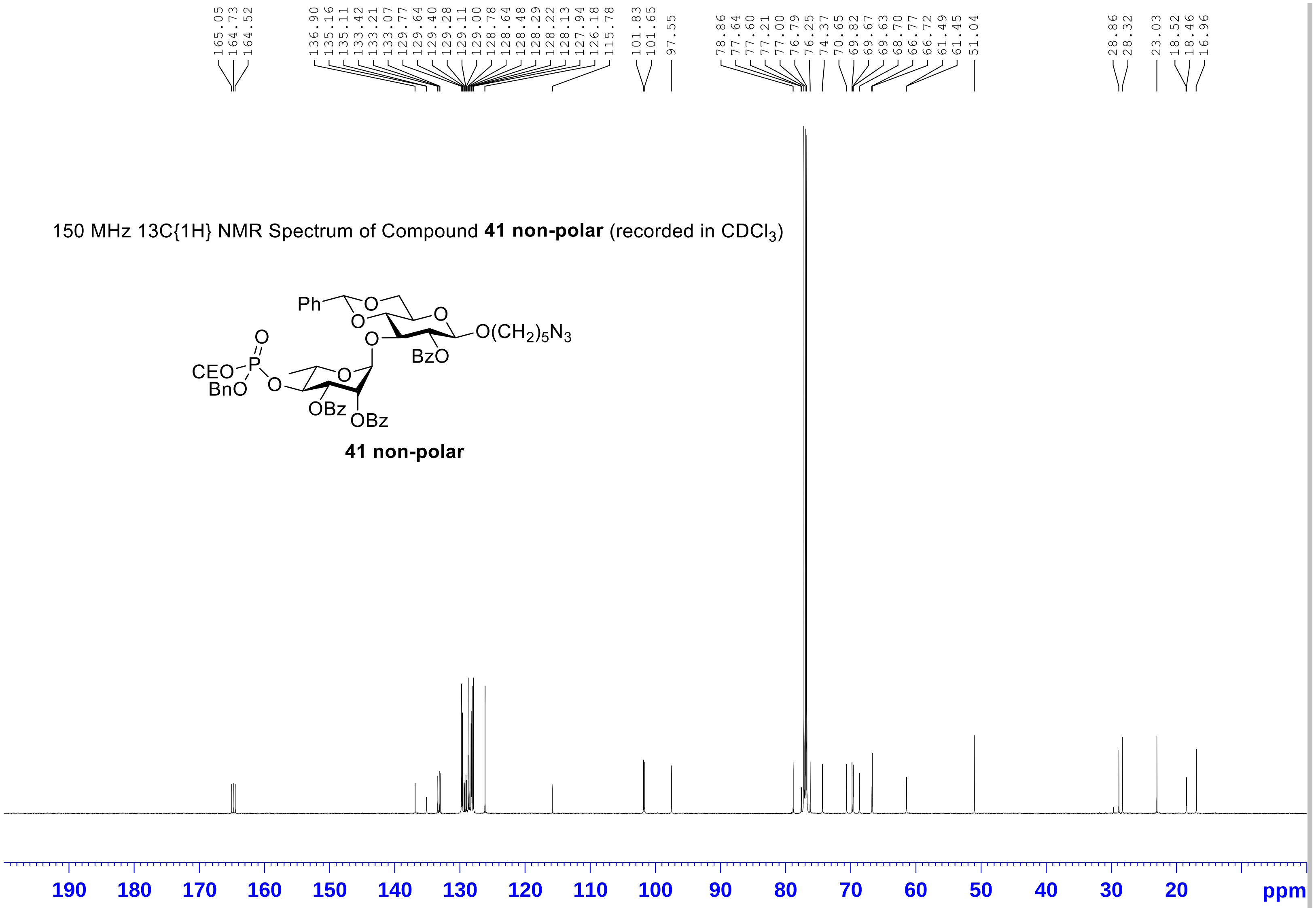

150 MHz 13C{1H} NMR Spectrum of Compound **41 non-polar** (recorded in $CDCl_3$)

41 non-polar

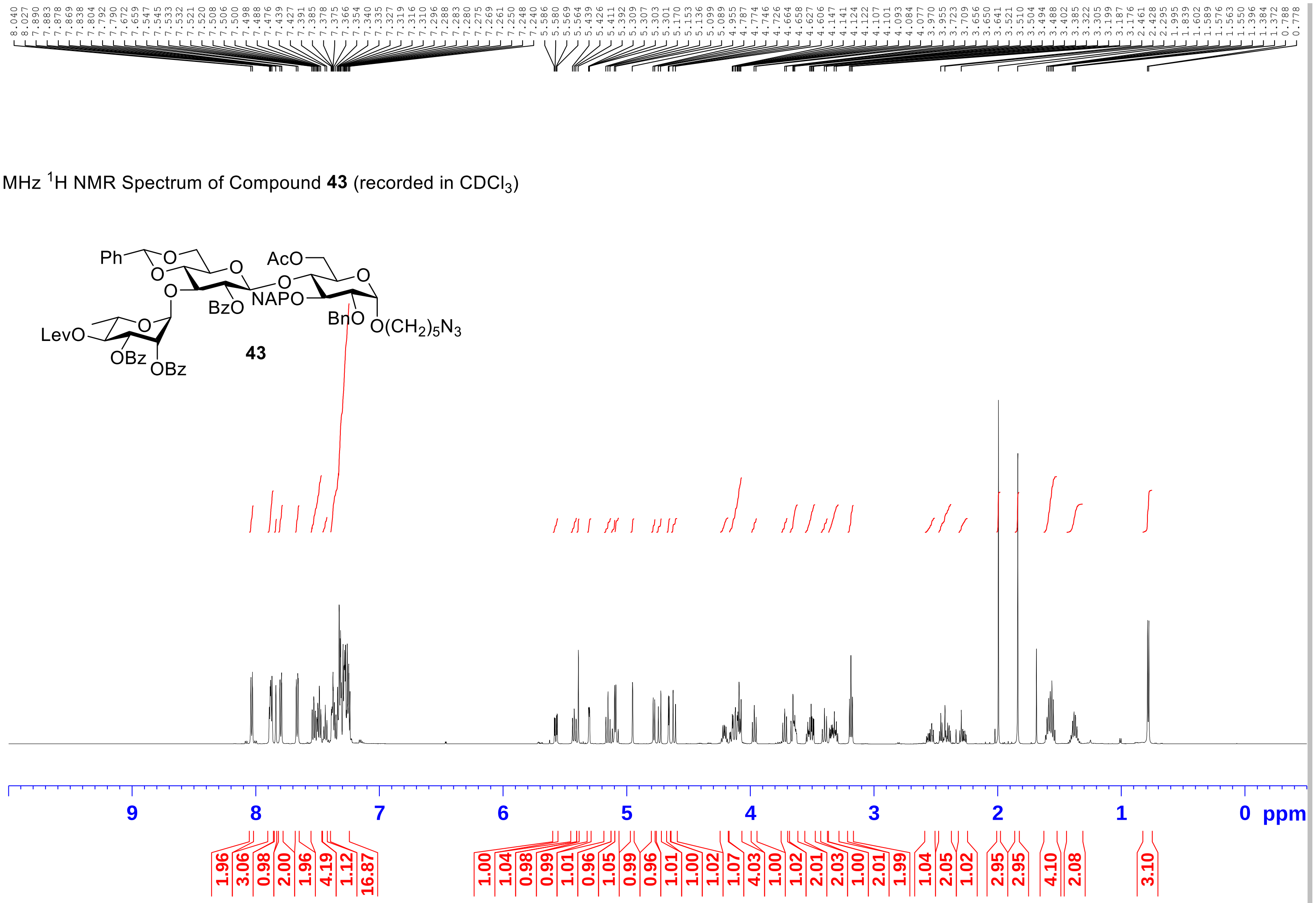

600 MHz ^{1}H NMR Spectrum of Compound **43** (recorded in $CDCl_3$)

150 MHz 13C{1H} NMR Spectrum of Compound **43** (recorded in $CDCl_3$)

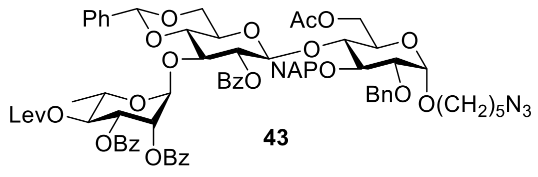

43

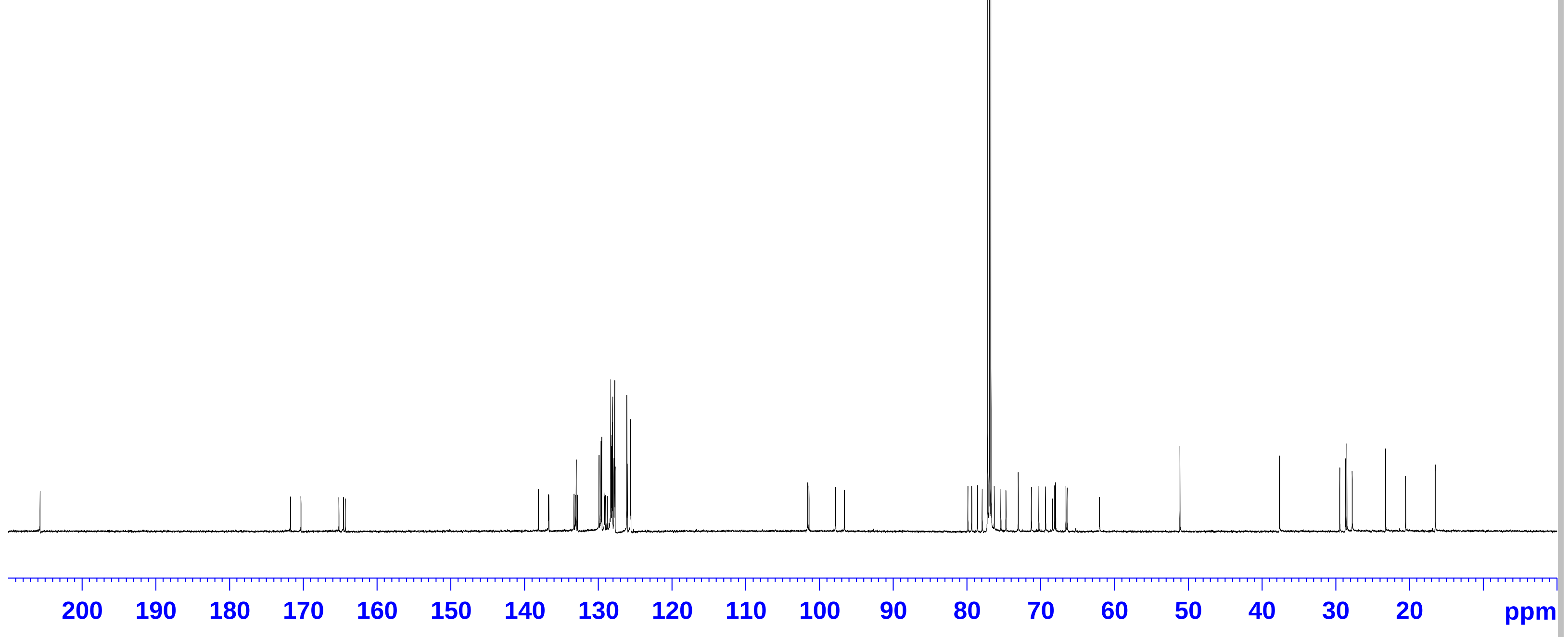

600 MHz ^{1}H NMR Spectrum of Compound **44** (recorded in $CDCl_3$)

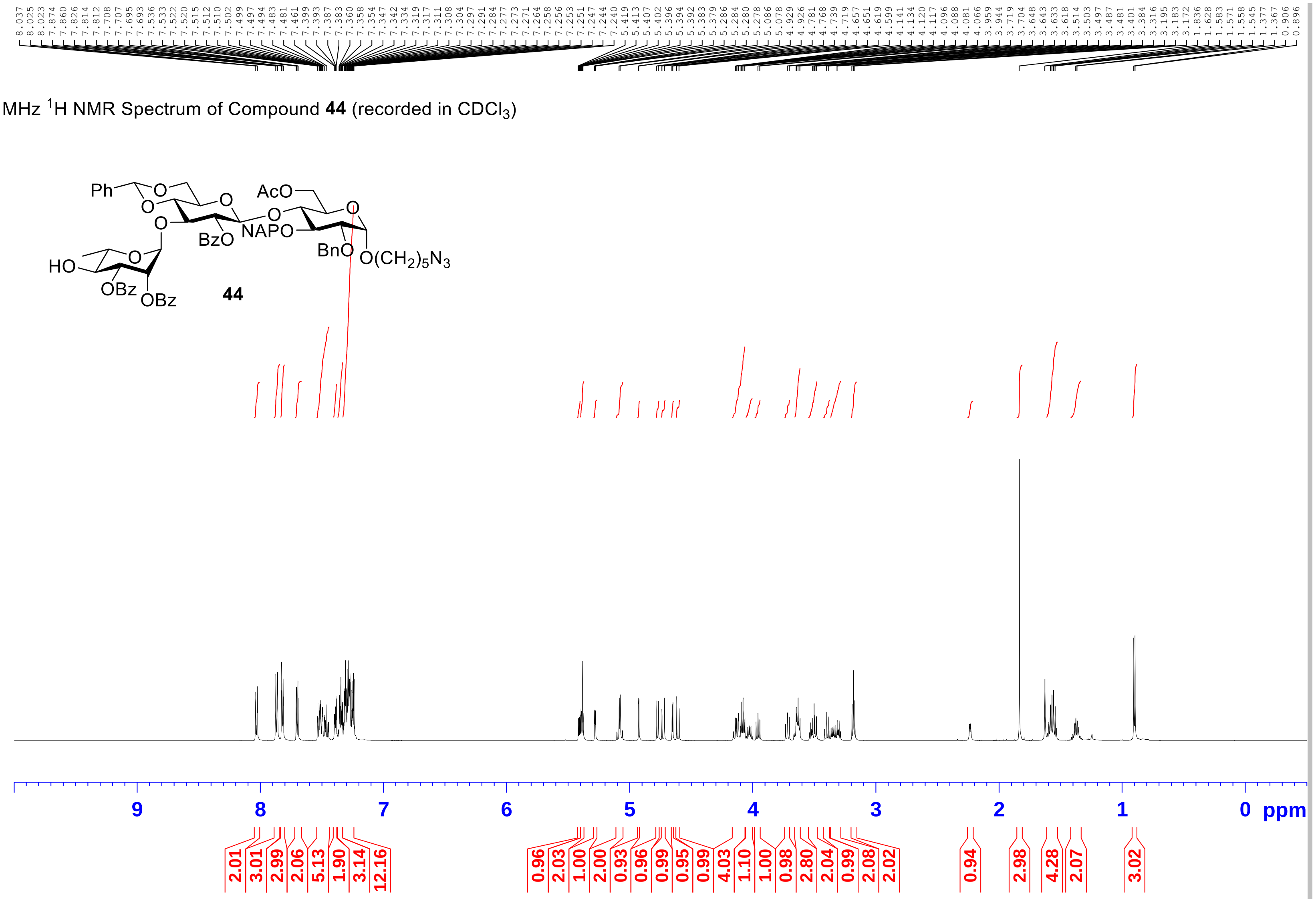

150 MHz 13C{1H} NMR Spectrum of Compound **44** (recorded in $CDCl_3$)

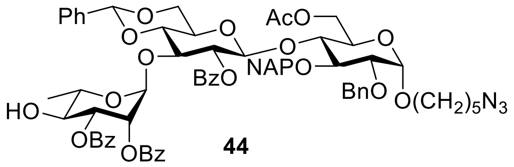

44

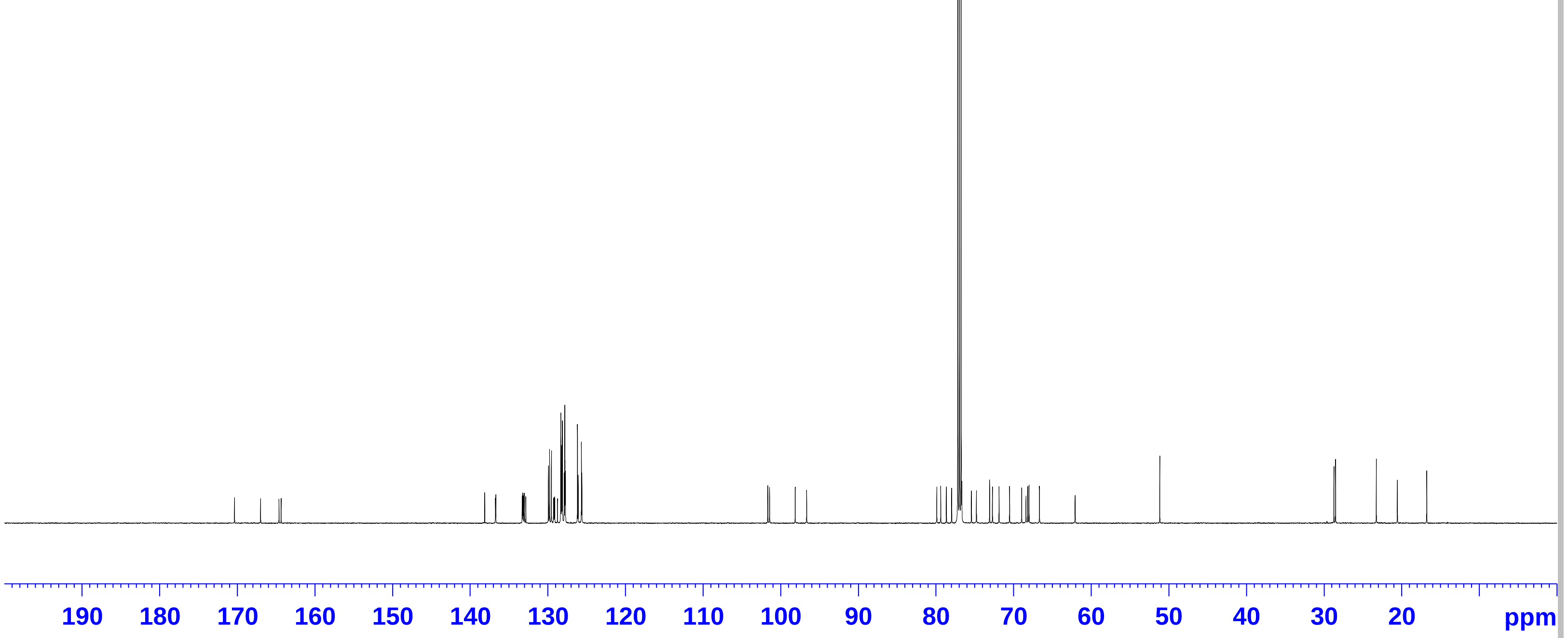

600 MHz ^{1}H NMR Spectrum of Compound **45 polar** (recorded in $CDCl_3$)

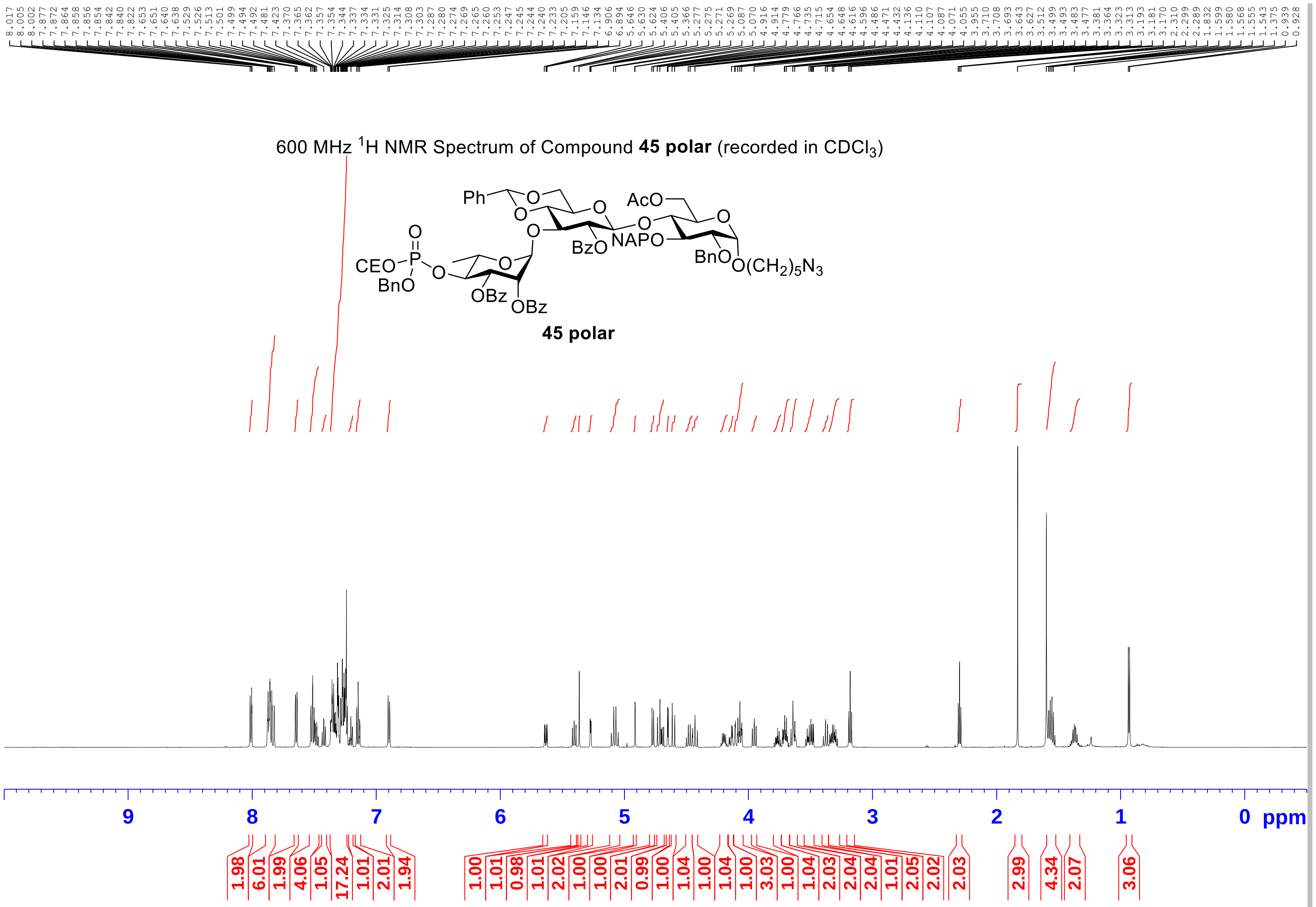

150 MHz 13C{1H} NMR Spectrum of Compound **45 polar** (recorded in $CDCl_3$)

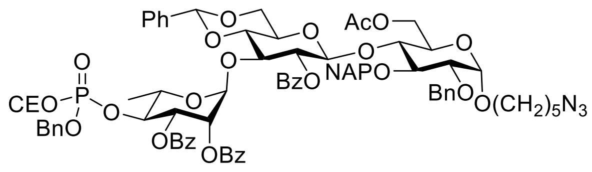

45 polar

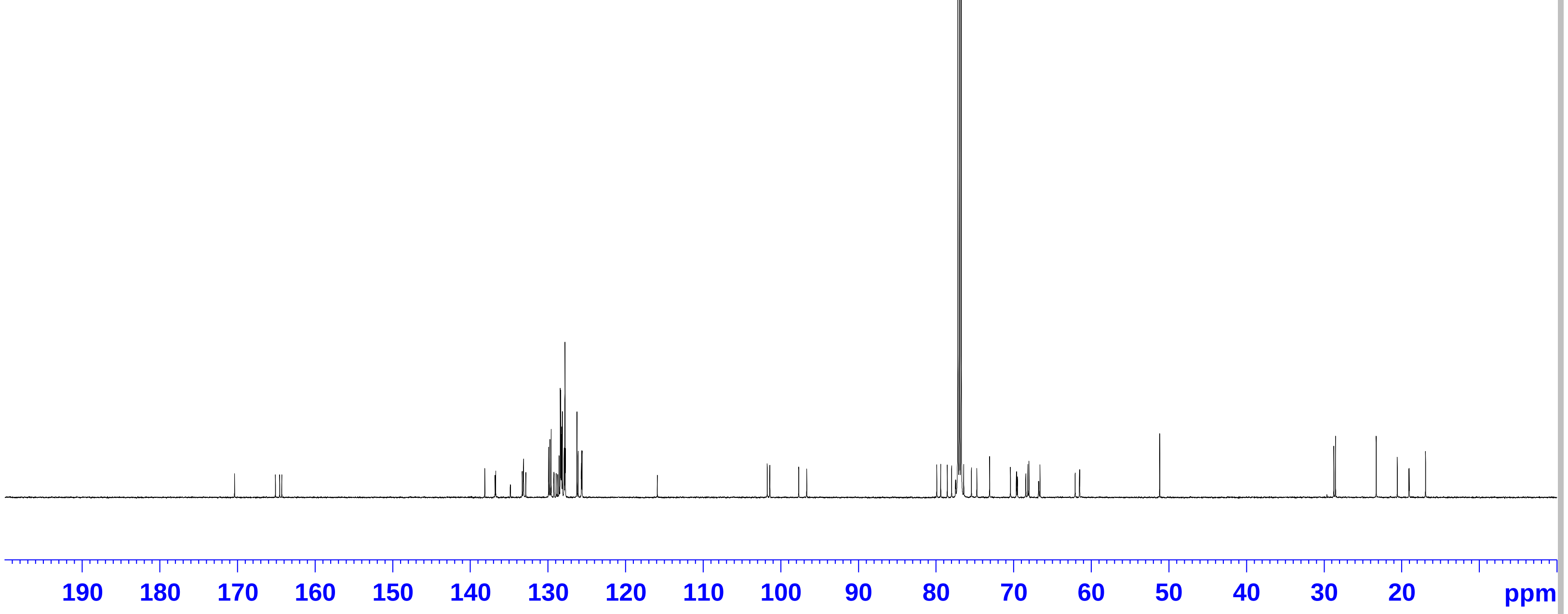

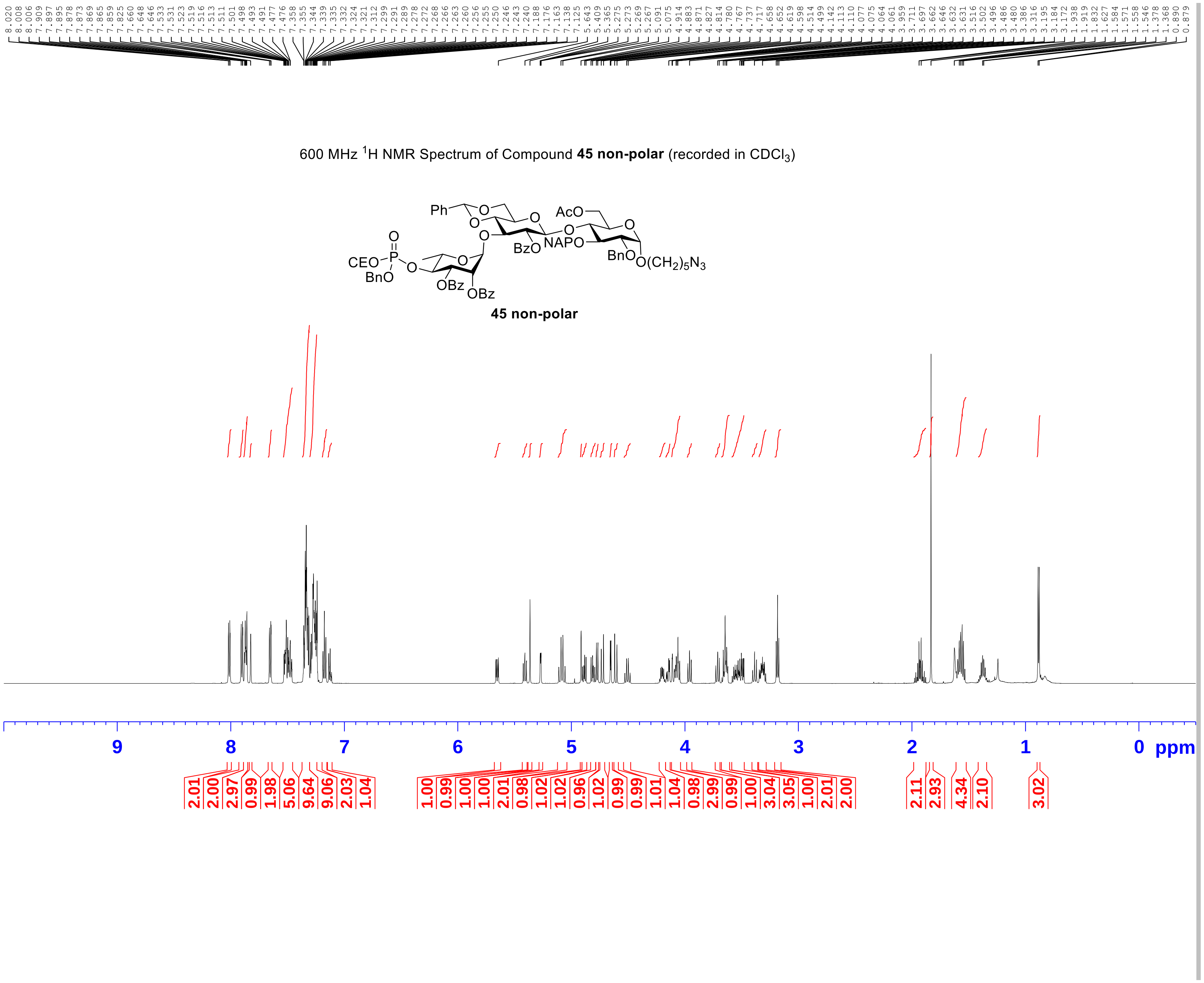
600 MHz ^{1}H NMR Spectrum of Compound **45 non-polar** (recorded in $CDCl_3$)
45 non-polar
ppm

150 MHz 13C{1H} NMR Spectrum of Compound **45 non-polar** (recorded in $CDCl_3$)

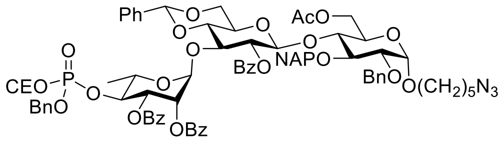

45 non-polar

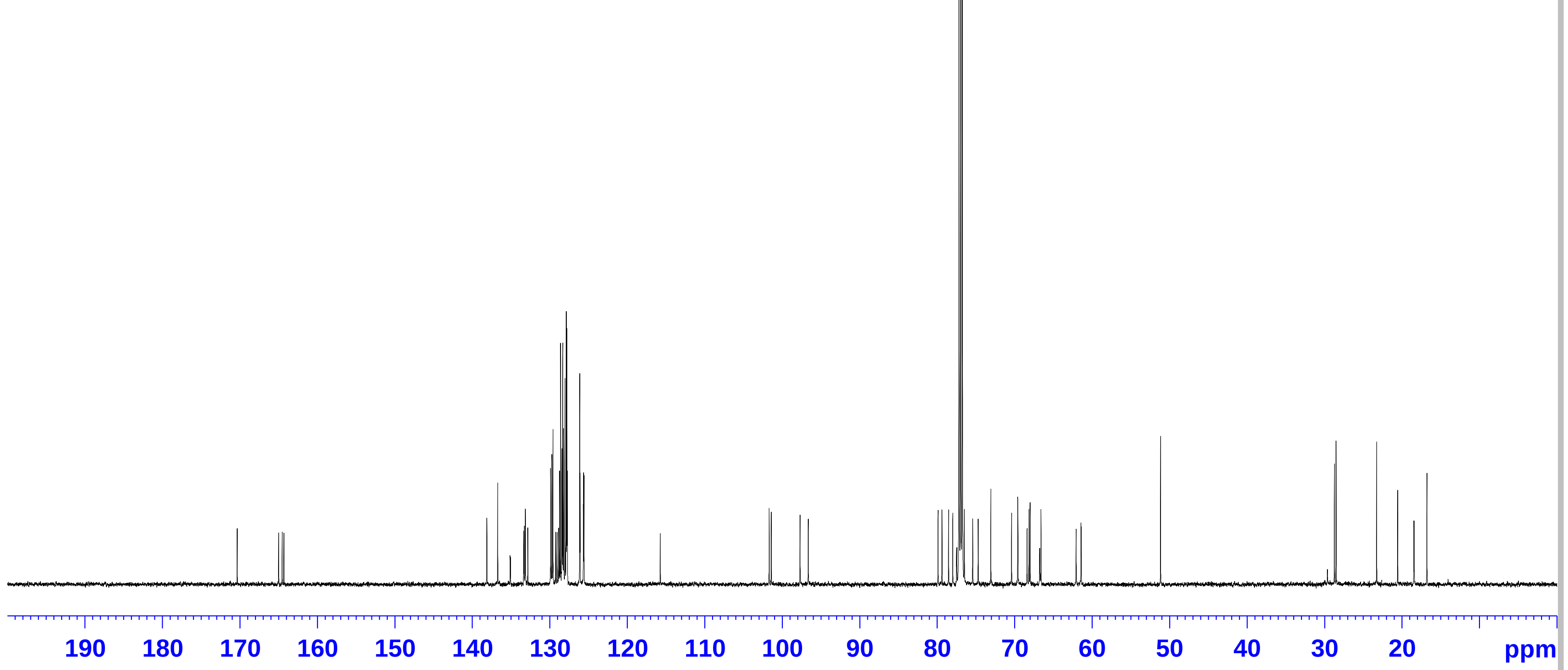

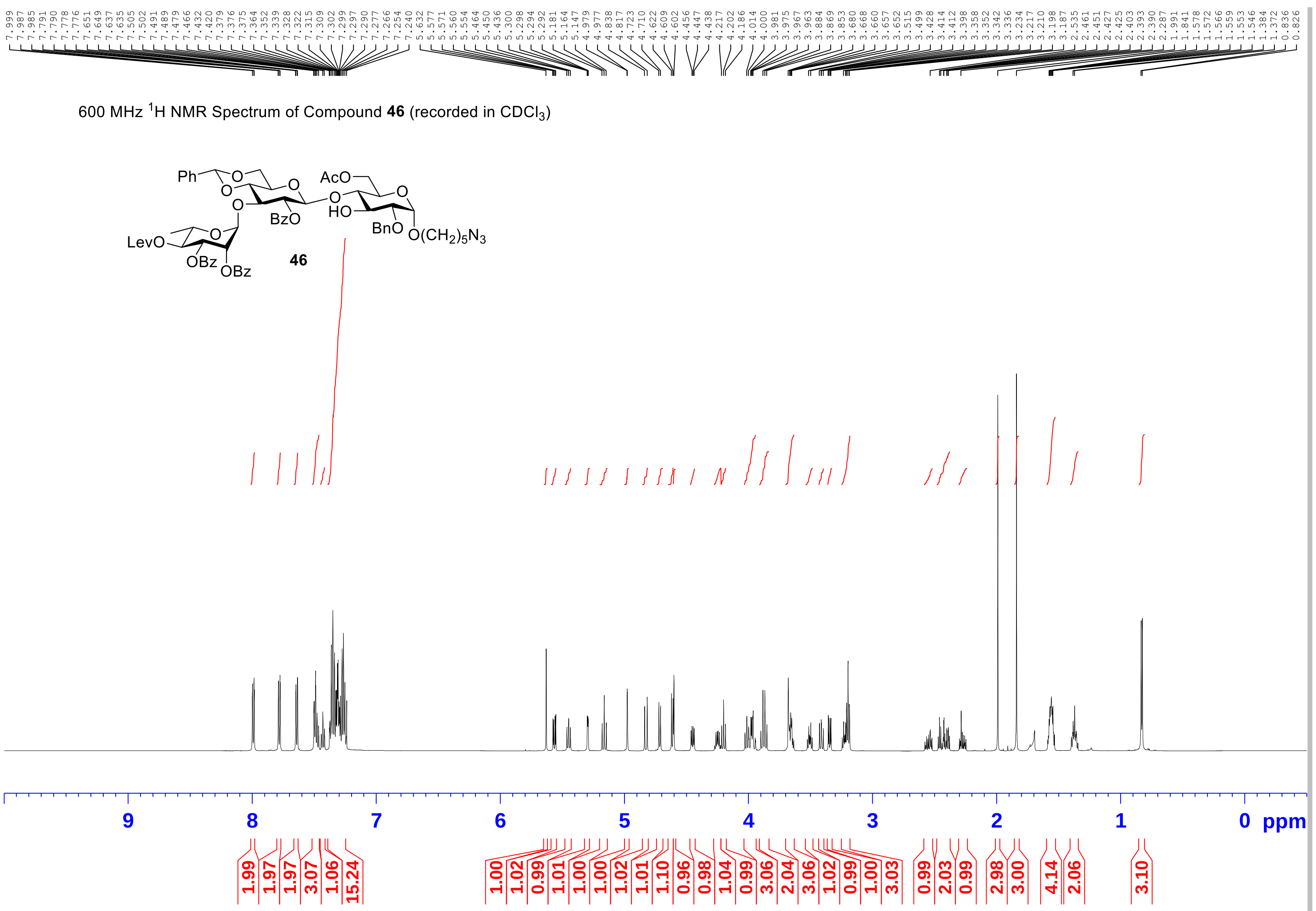

600 MHz ^{1}H NMR Spectrum of Compound **46** (recorded in $CDCl_3$)

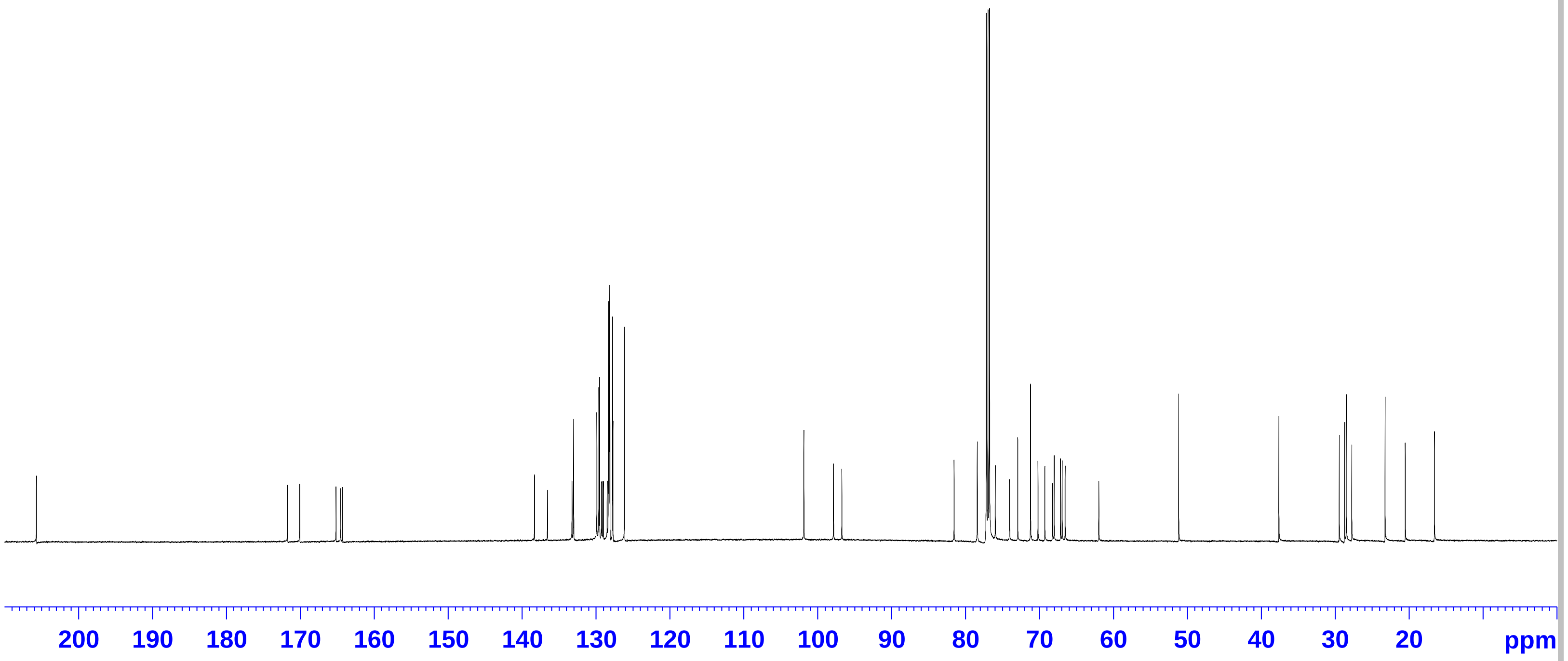

150 MHz 13C{1H} NMR Spectrum of Compound **46** (recorded in $CDCl_3$)

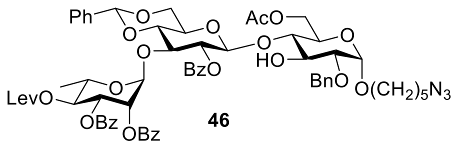

46

600 MHz ^{1}H NMR Spectrum of Compound **47** (recorded in $CDCl_3$)

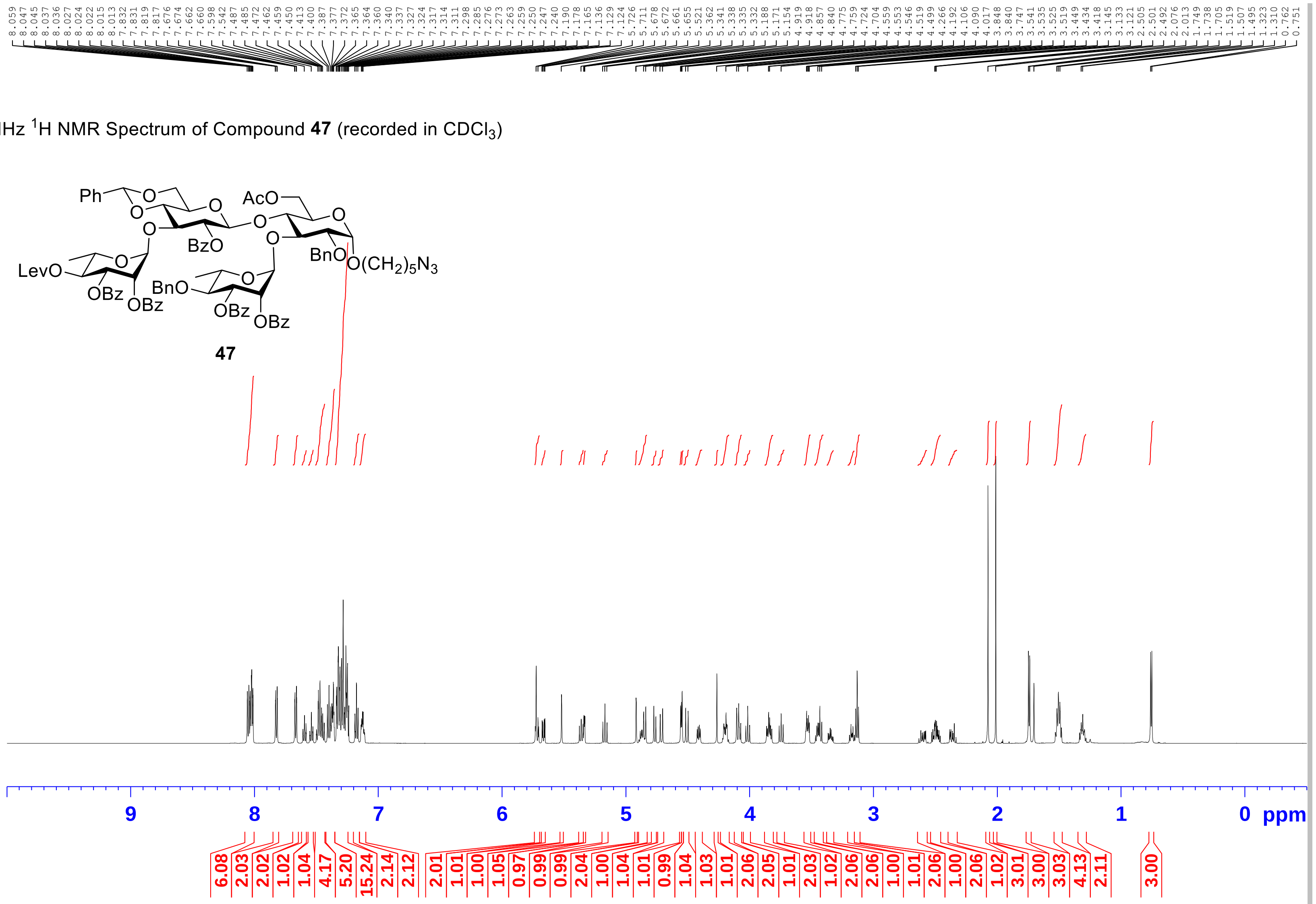

47

150 MHz 13C{1H} NMR Spectrum of Compound **47** (recorded in $CDCl_3$)

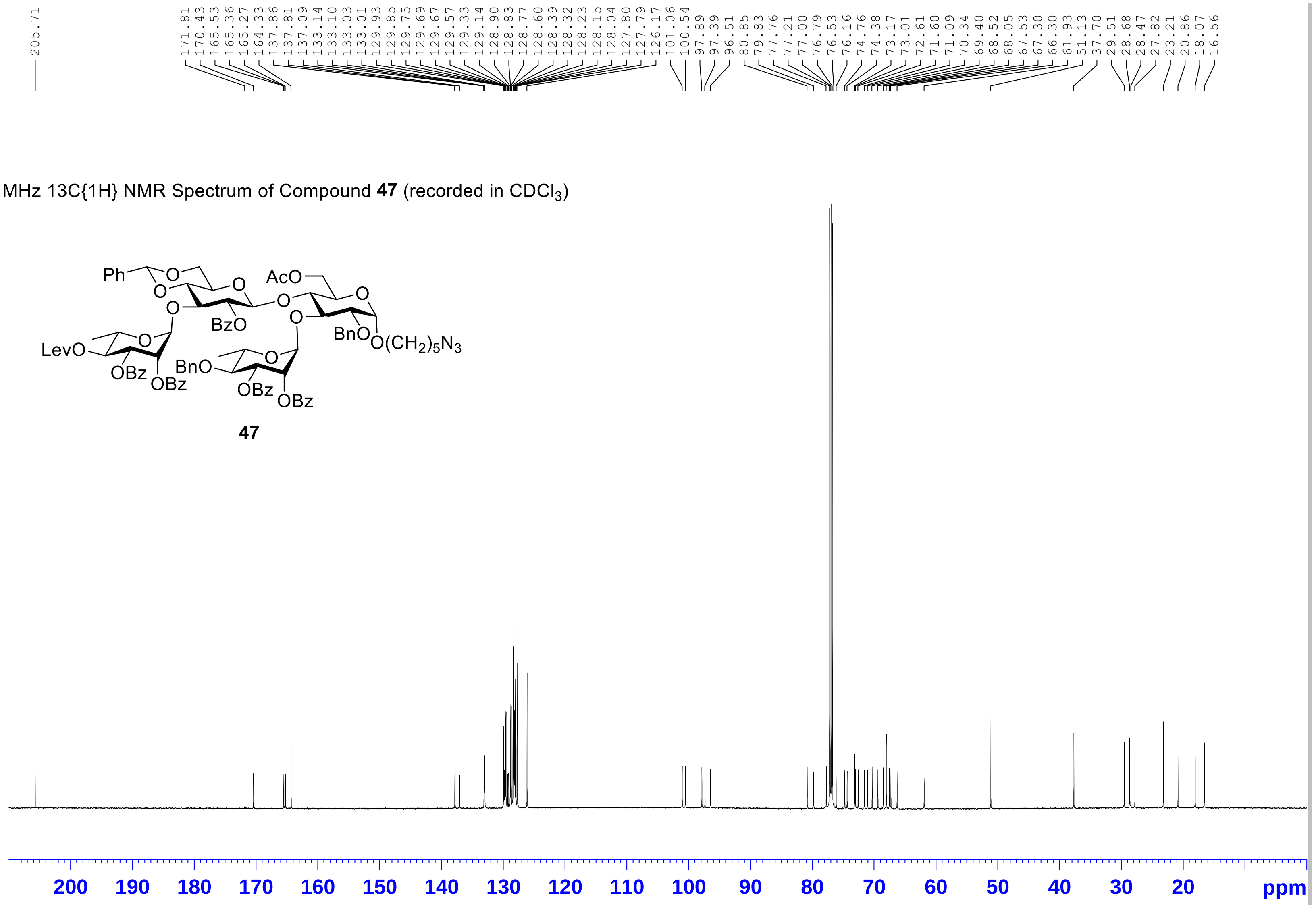

600 MHz ^{1}H NMR Spectrum of Compound **48** (recorded in $CDCl_3$)

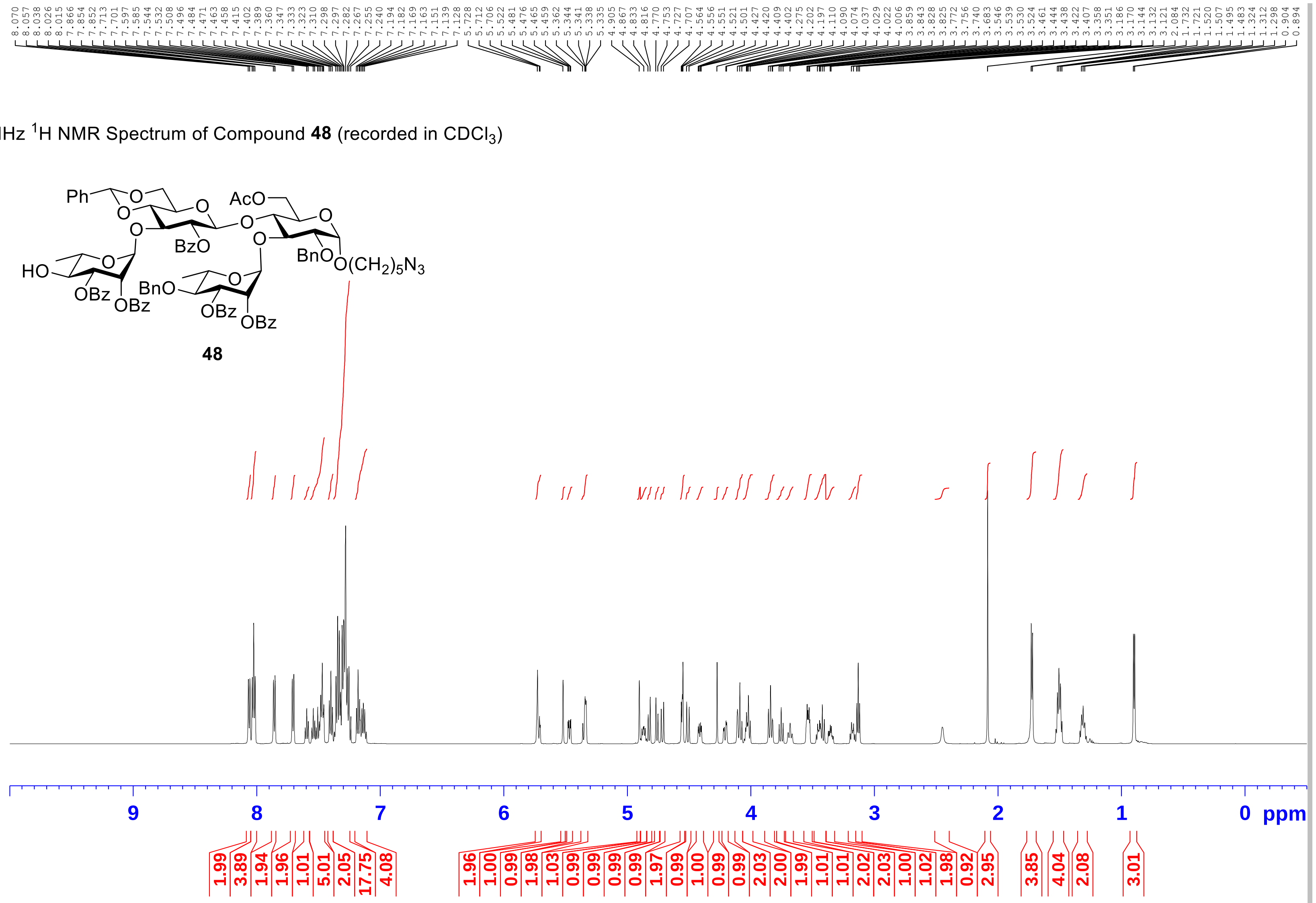

150 MHz 13C{1H} NMR Spectrum of Compound **48** (recorded in $CDCl_3$)

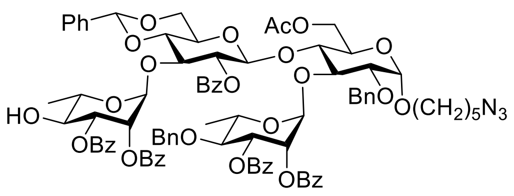

48

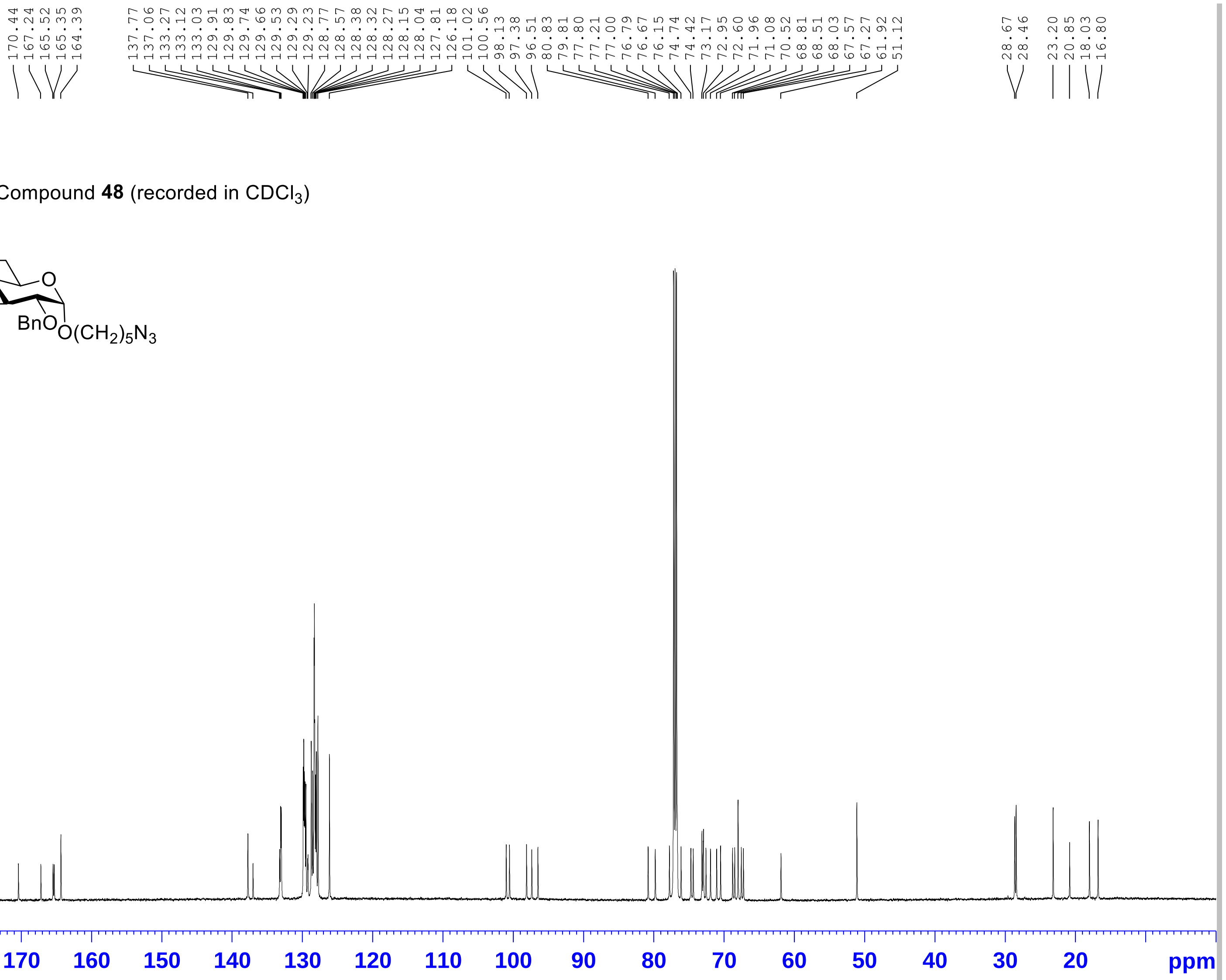

600 MHz ^{1}H NMR Spectrum of Compound **49 polar** (recorded in $CDCl_3$)

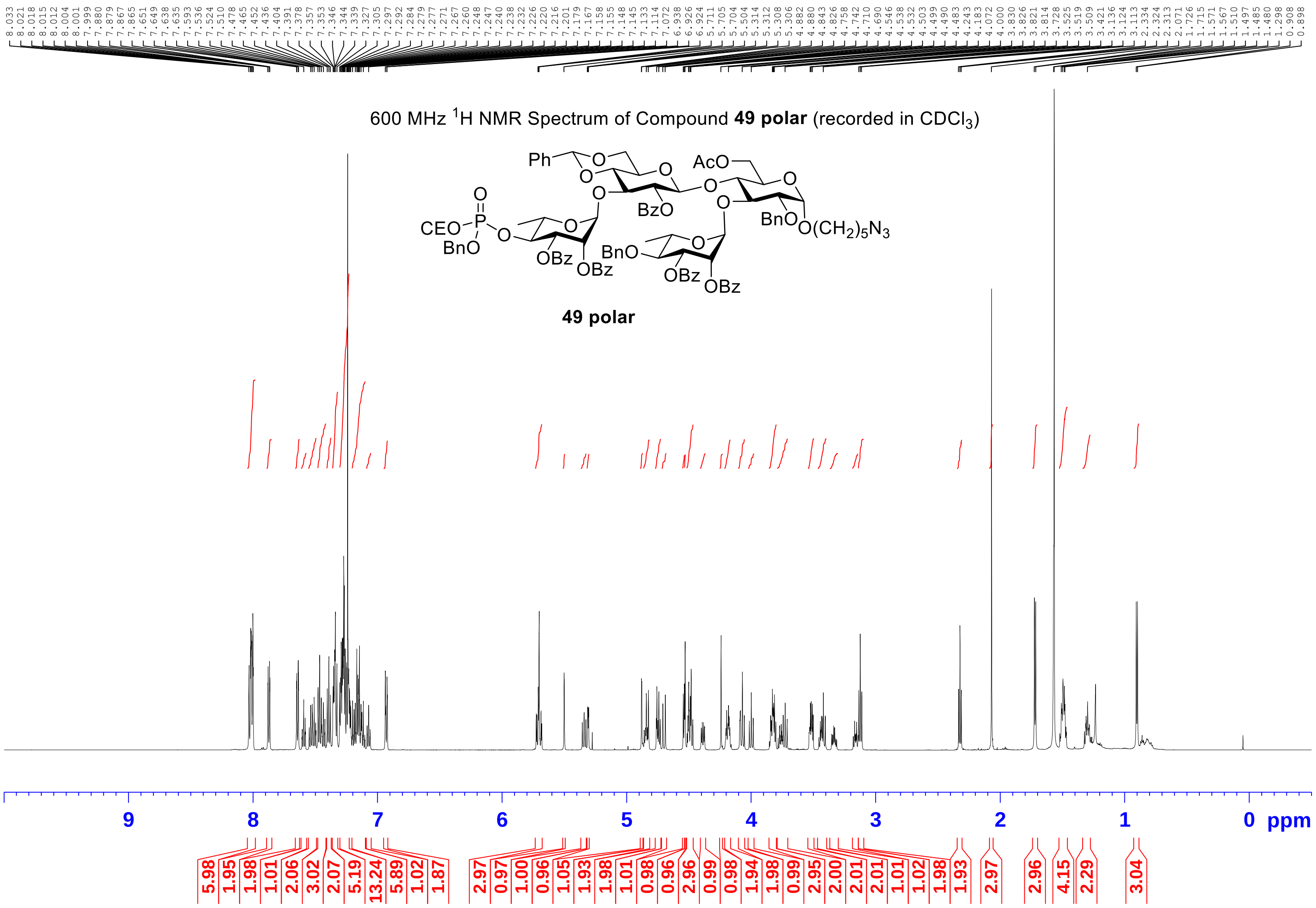

150 MHz 13C{1H} NMR Spectrum of Compound **49 polar** (recorded in $CDCl_3$)

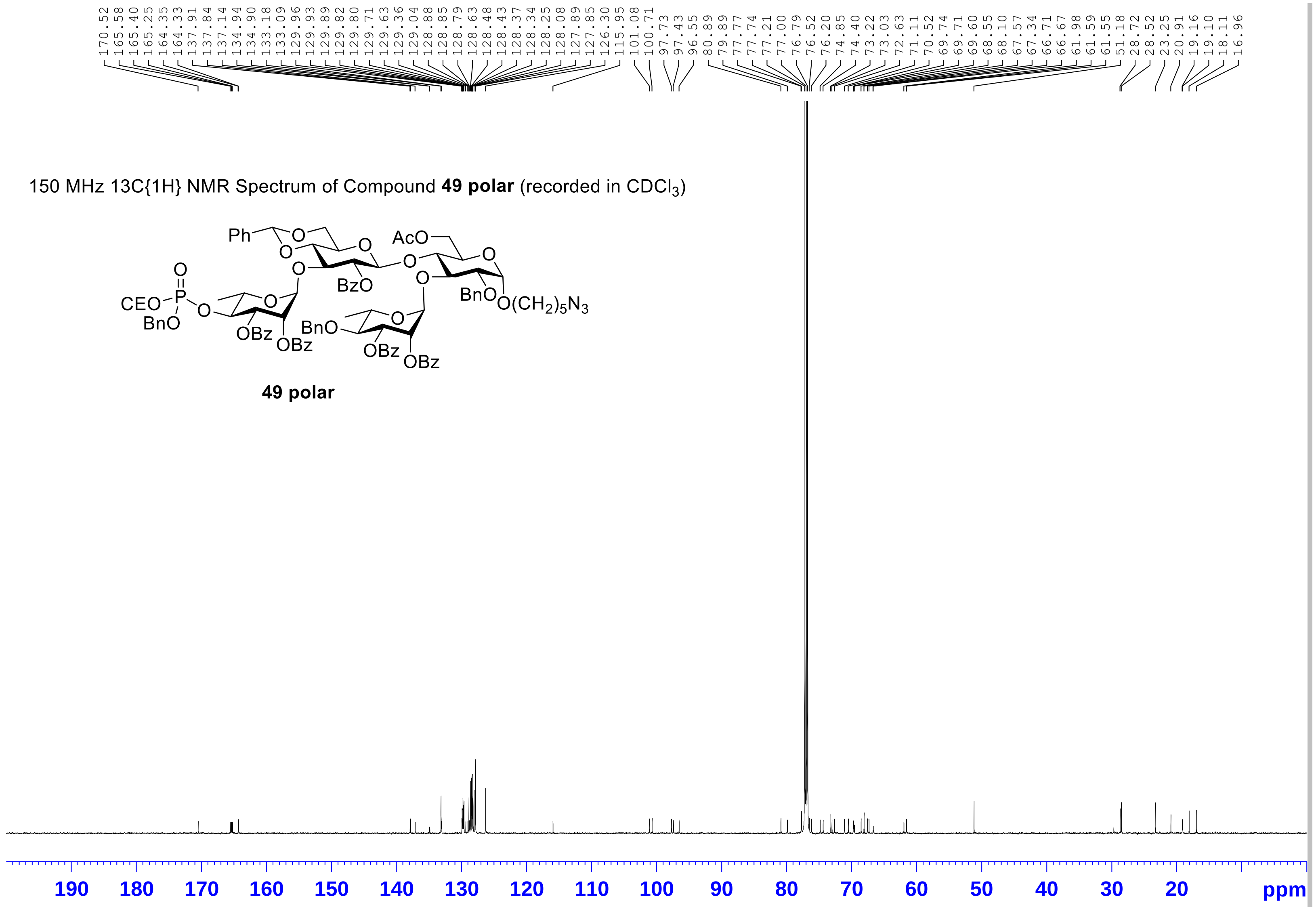

49 polar

600 MHz ^{1}H NMR Spectrum of Compound **49 polar** (recorded in $CDCl_3$)

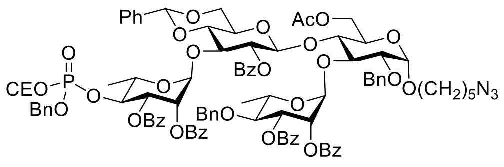

49 less polar

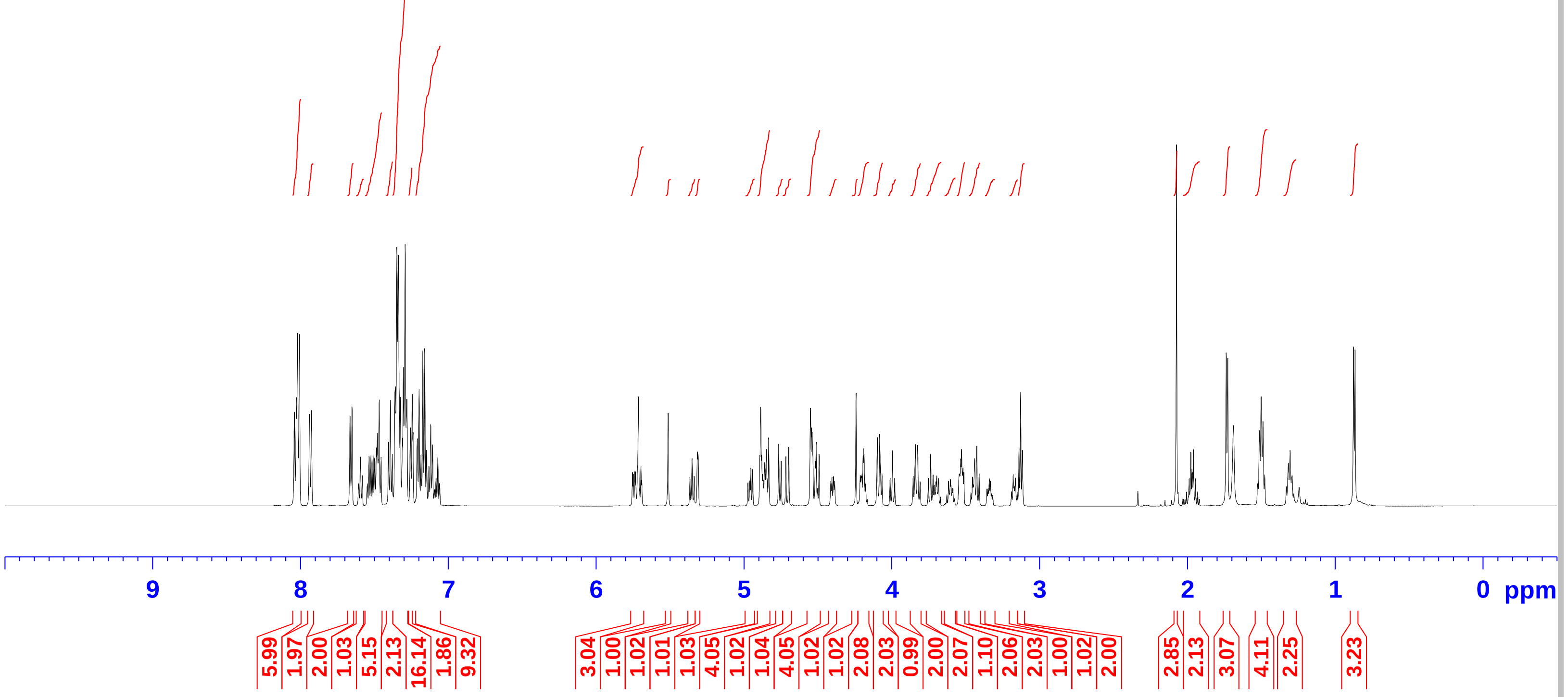

150 MHz 13C{1H} NMR Spectrum of Compound **49 non-polar** (recorded in $CDCl_3$)

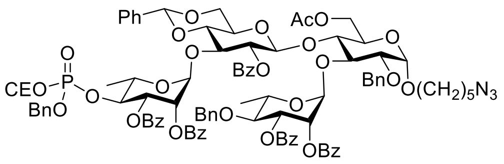

49 non-polar

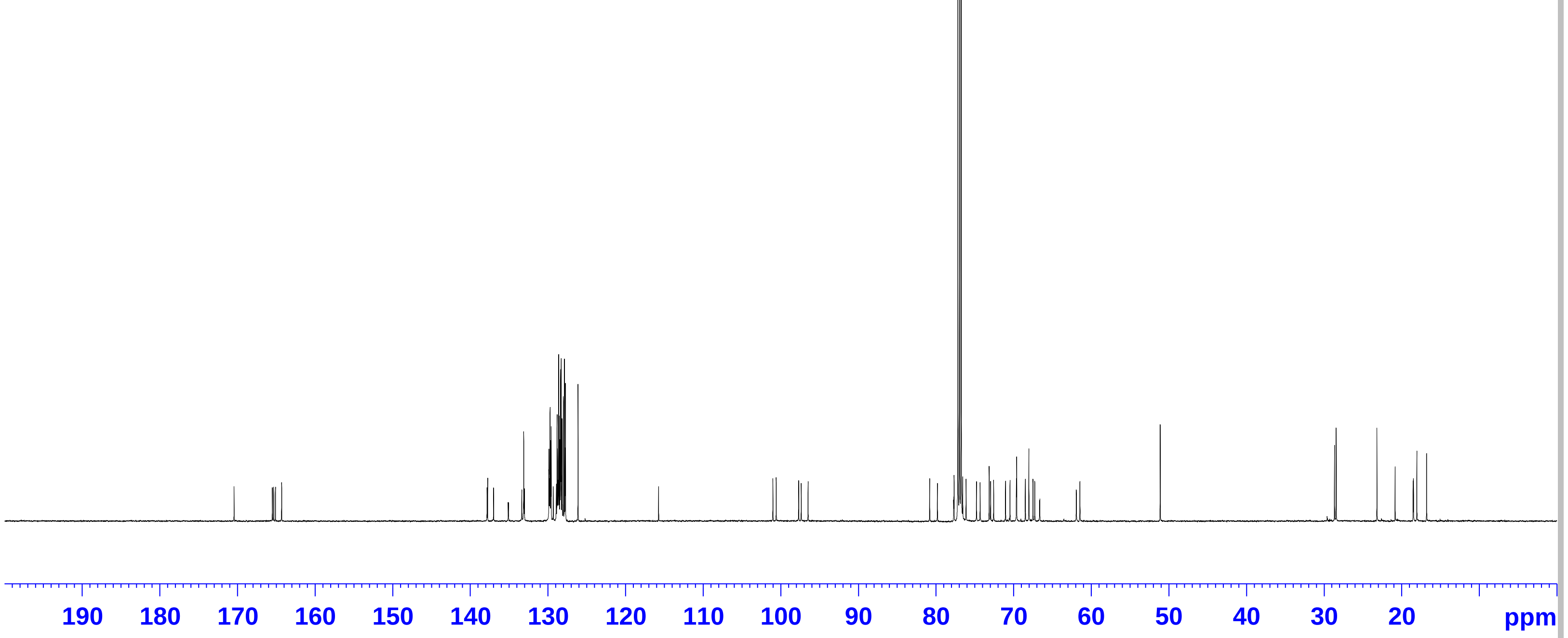

600 MHz ^{1}H NMR Spectrum of Compound **50** (recorded in $CDCl_3$)

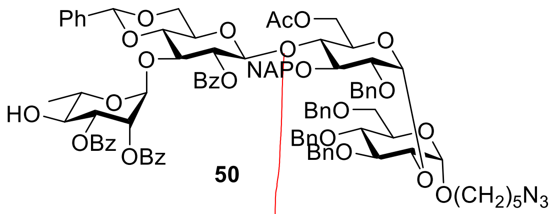

50

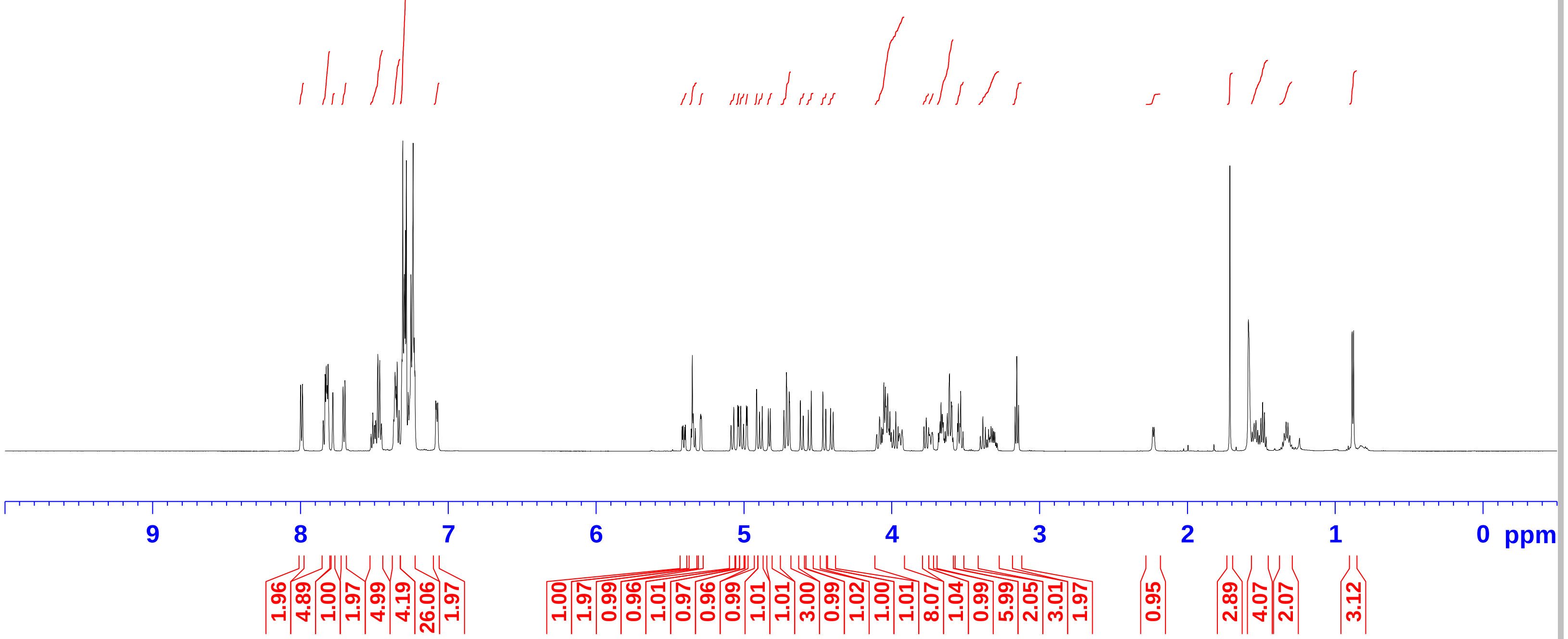

150 MHz 13C{1H} NMR Spectrum of Compound **50** (recorded in $CDCl_3$)

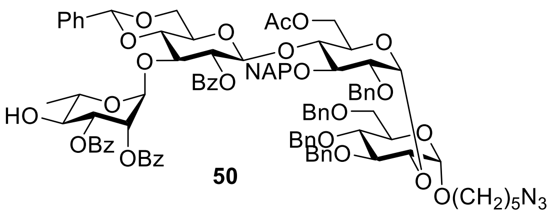

50

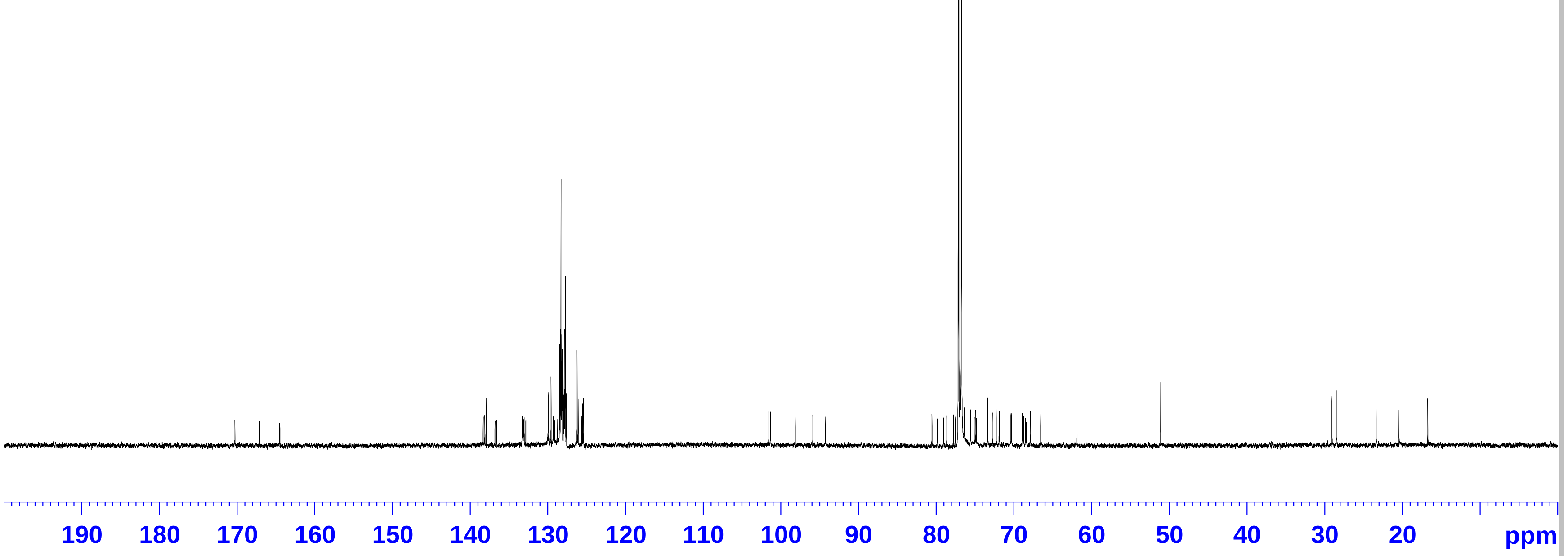

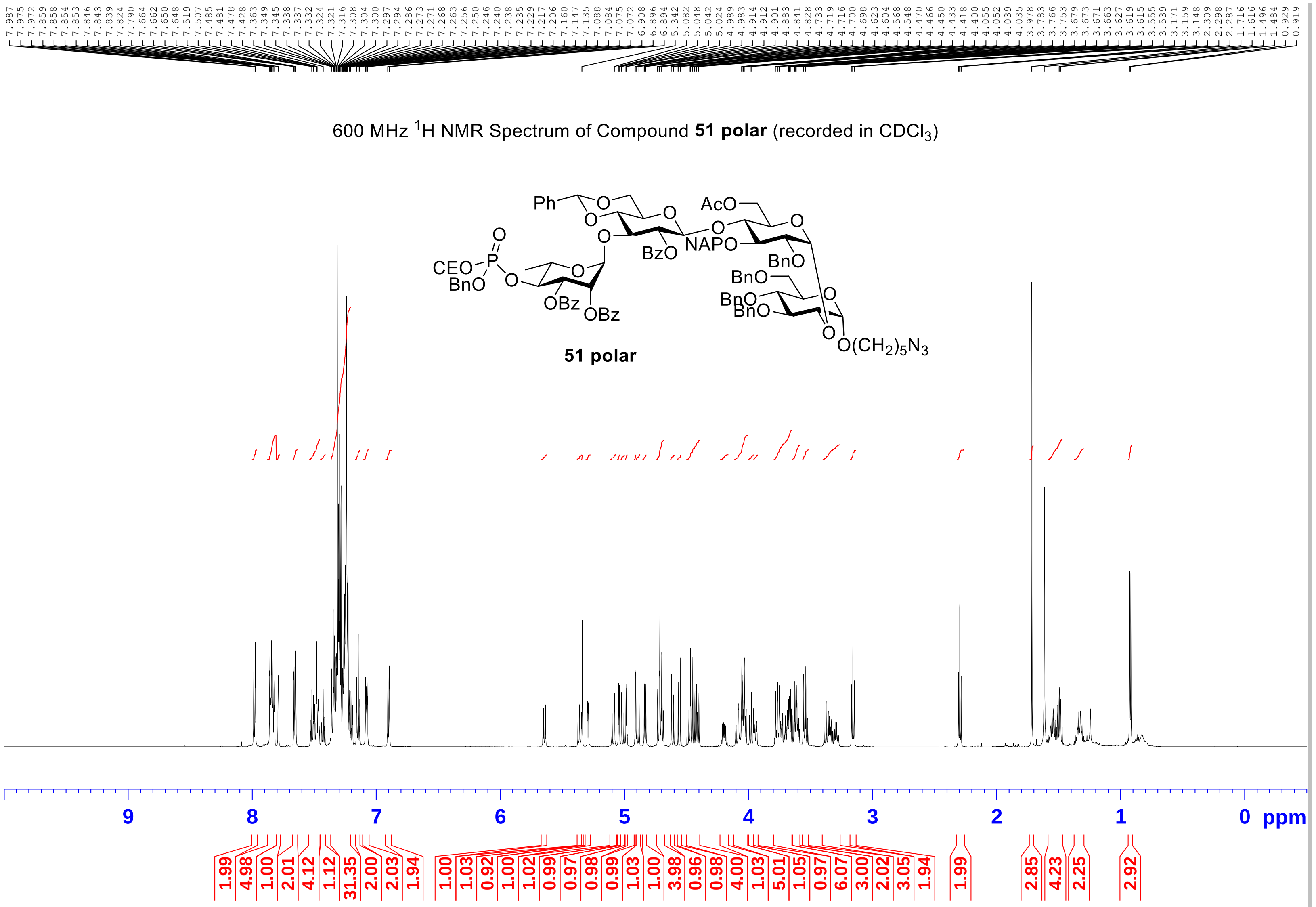

600 MHz ^{1}H NMR Spectrum of Compound **51 polar** (recorded in $CDCl_3$)

150 MHz 13C{1H} NMR Spectrum of Compound **51 polar** (recorded in $CDCl_3$)

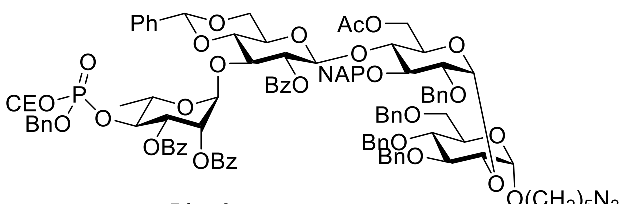

51polar

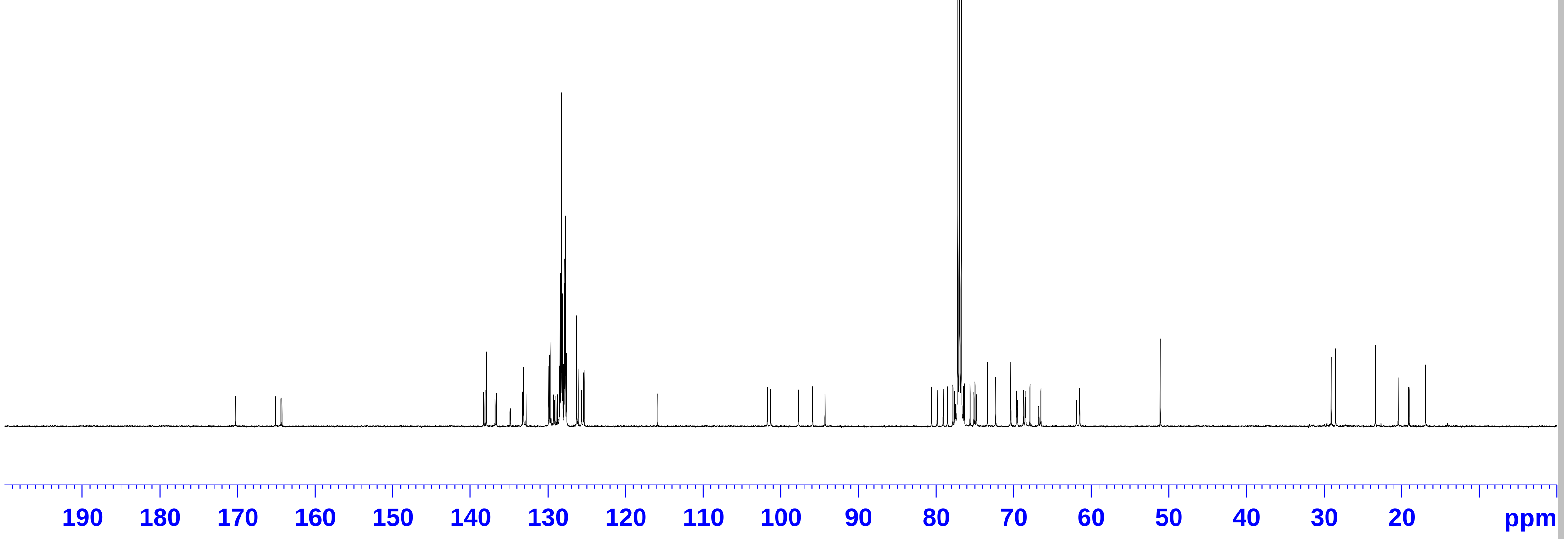

600 MHz ^{1}H NMR Spectrum of Compound **51 non-polar** (recorded in $CDCl_3$)

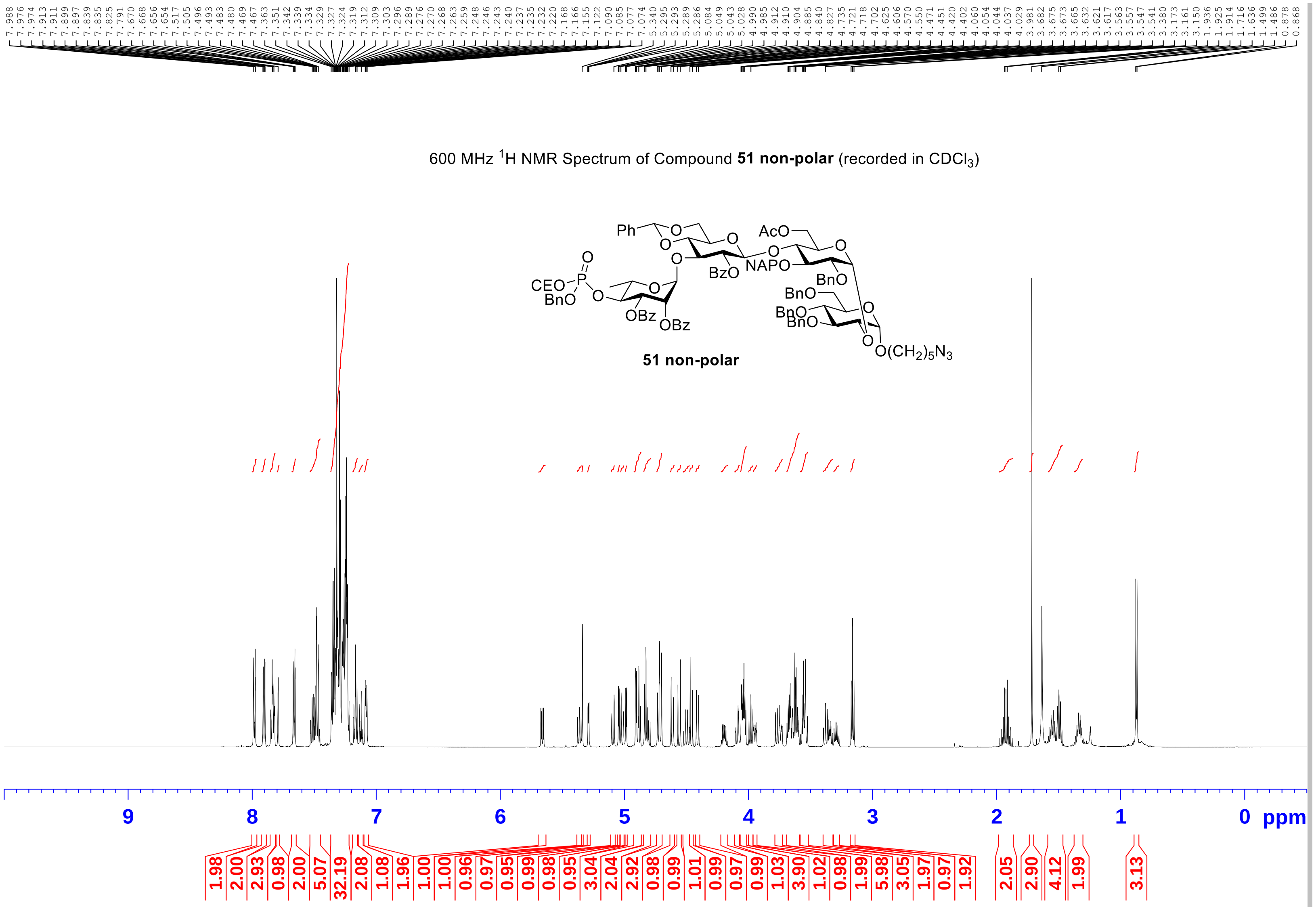

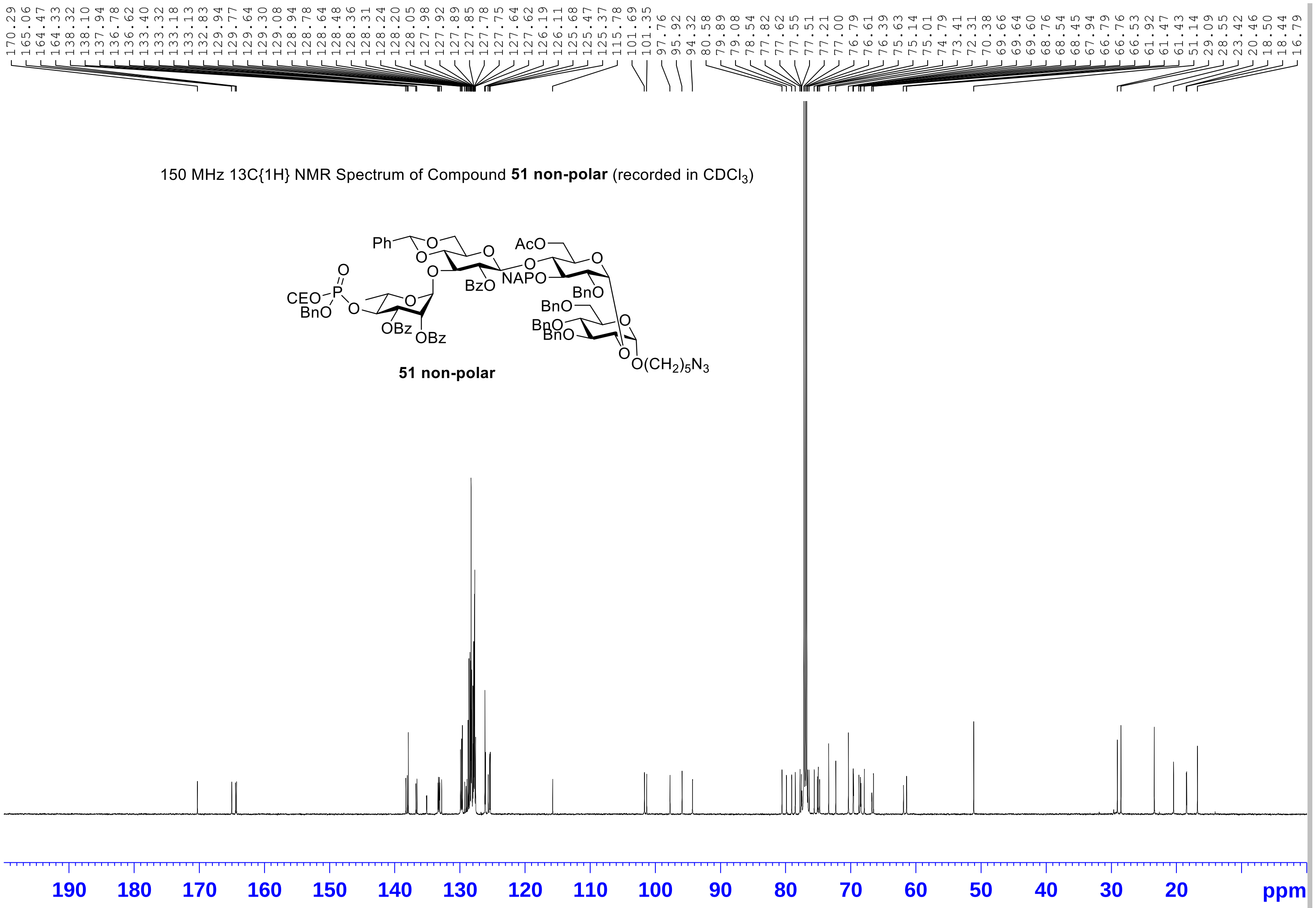

150 MHz 13C{1H} NMR Spectrum of Compound **51 non-polar** (recorded in $CDCl_3$)

600 MHz ^{1}H NMR Spectrum of Compound **52** (recorded in D_2O)

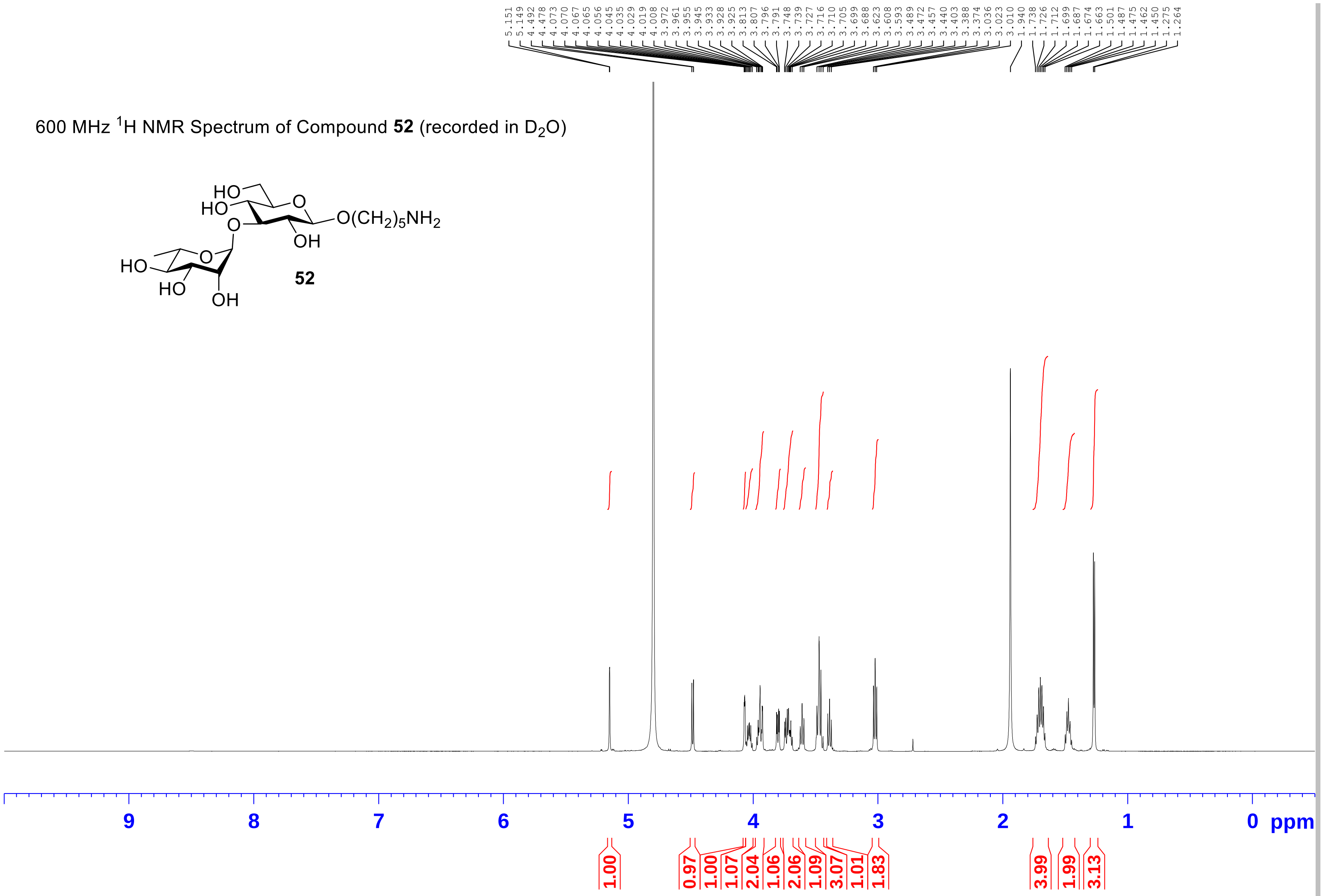

150 MHz 13C{1H} NMR Spectrum of Compound **52** (recorded in D_2O)

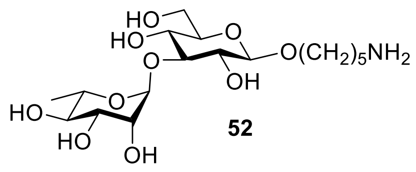

52

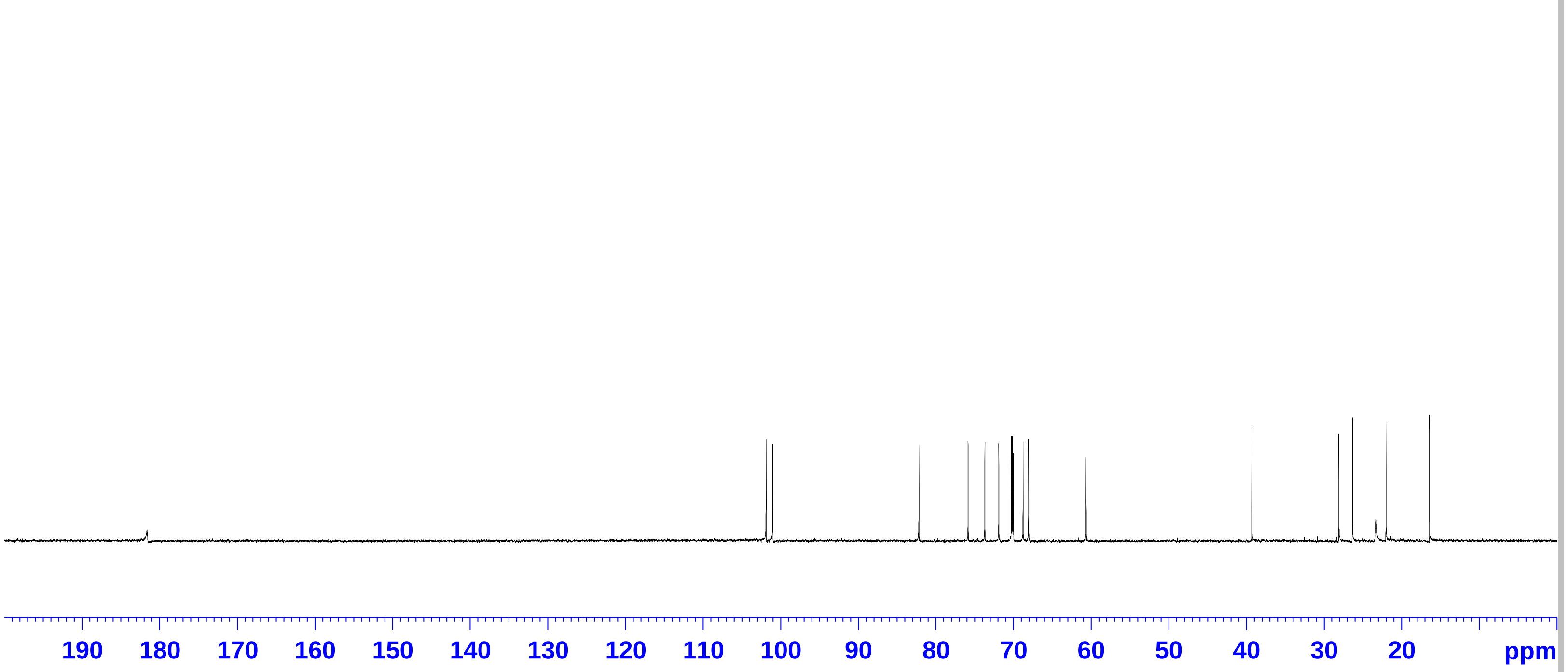

600 MHz ^{1}H NMR Spectrum of Compound **53** (recorded in D_2O)

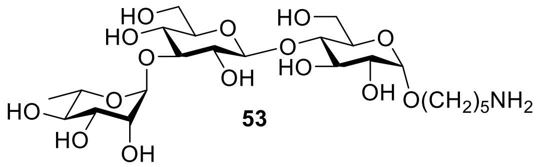

53

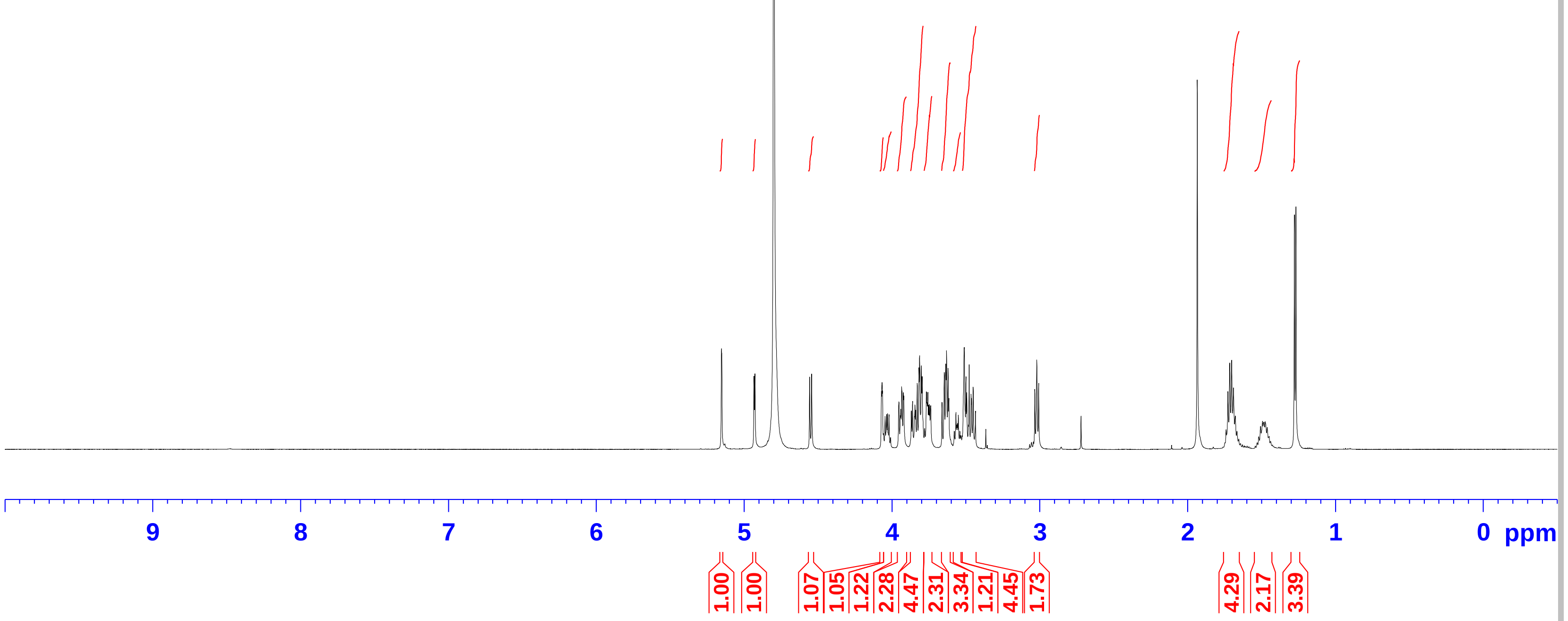

150 MHz 13C{1H} NMR Spectrum of Compound **53** (recorded in D_2O)

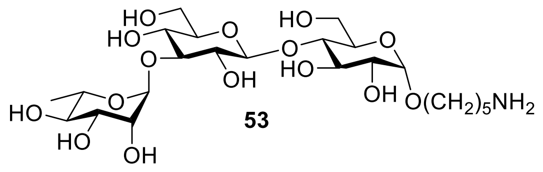

53

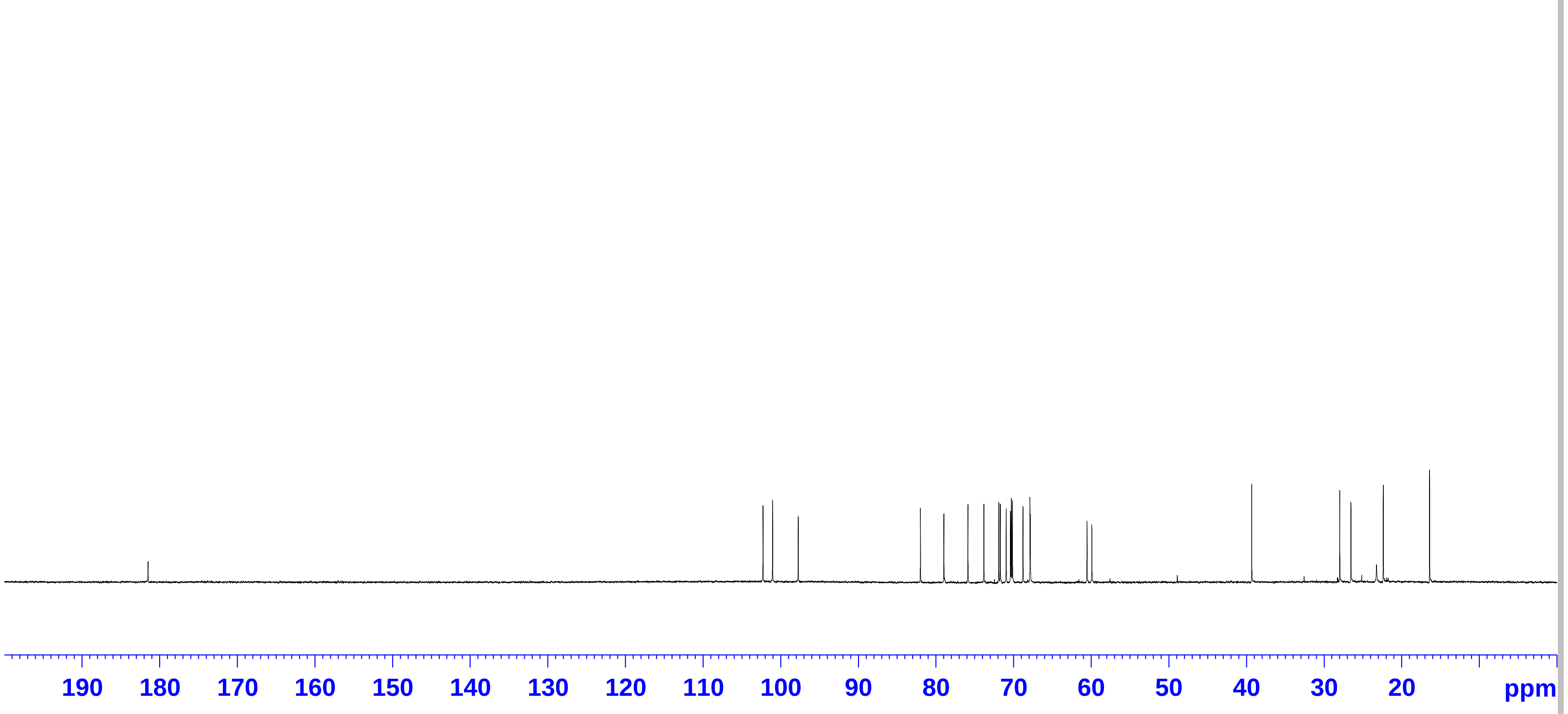

600 MHz ^{1}H NMR Spectrum of Compound **54** (recorded in D_2O)

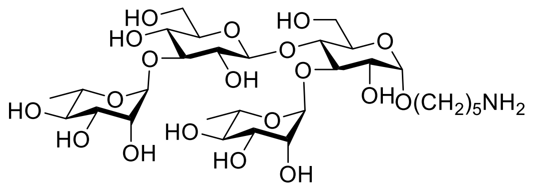

54

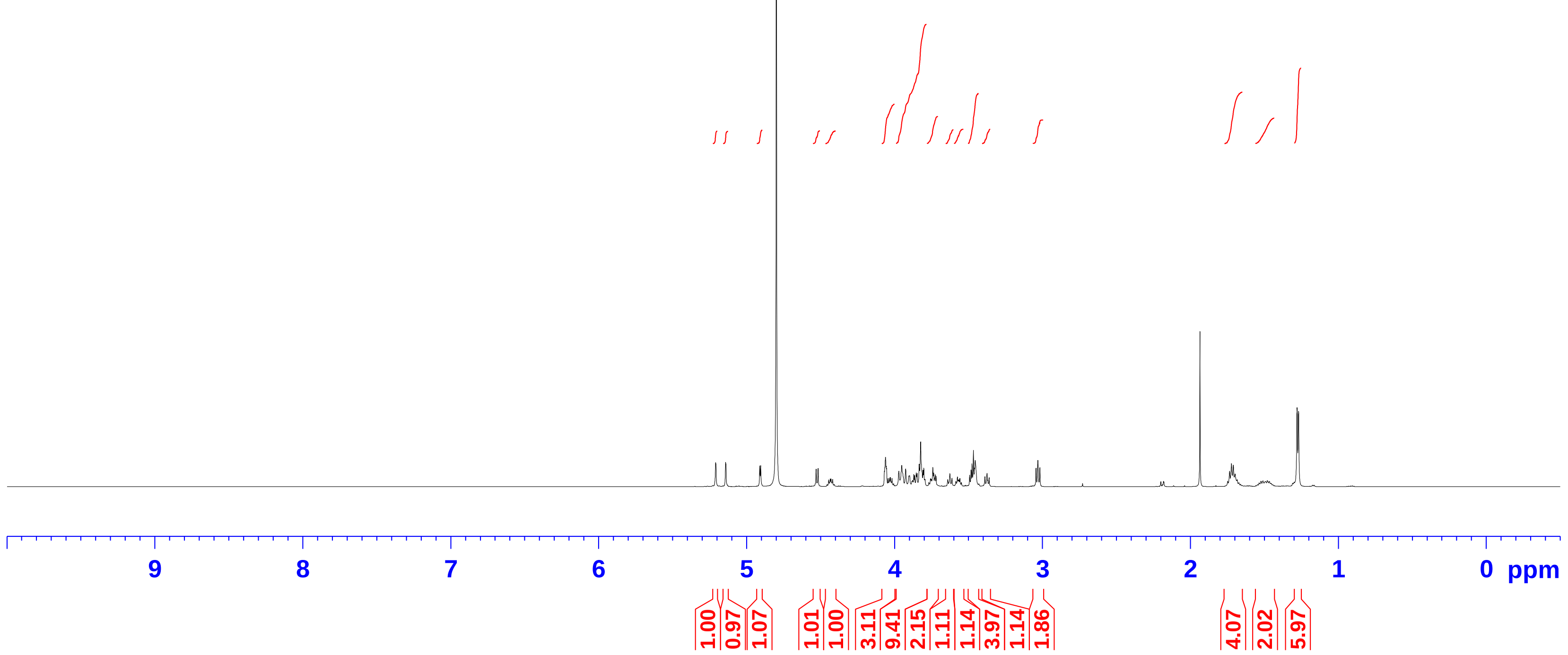

150 MHz 13C{1H} NMR Spectrum of Compound **54** (recorded in D_2O)

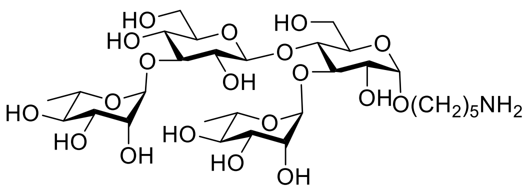

54

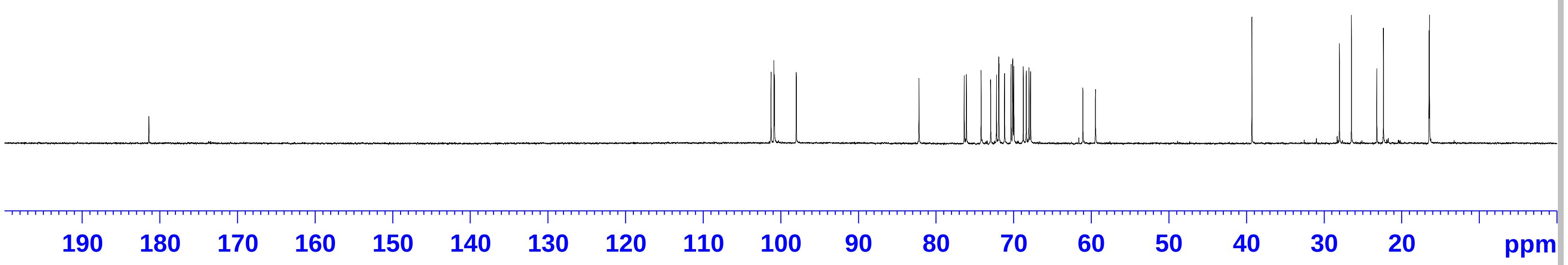

600 MHz ^{1}H NMR Spectrum of Compound **54** (recorded in D_2O)

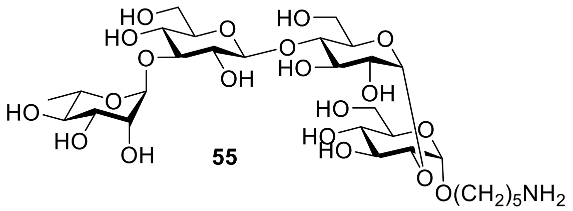

55

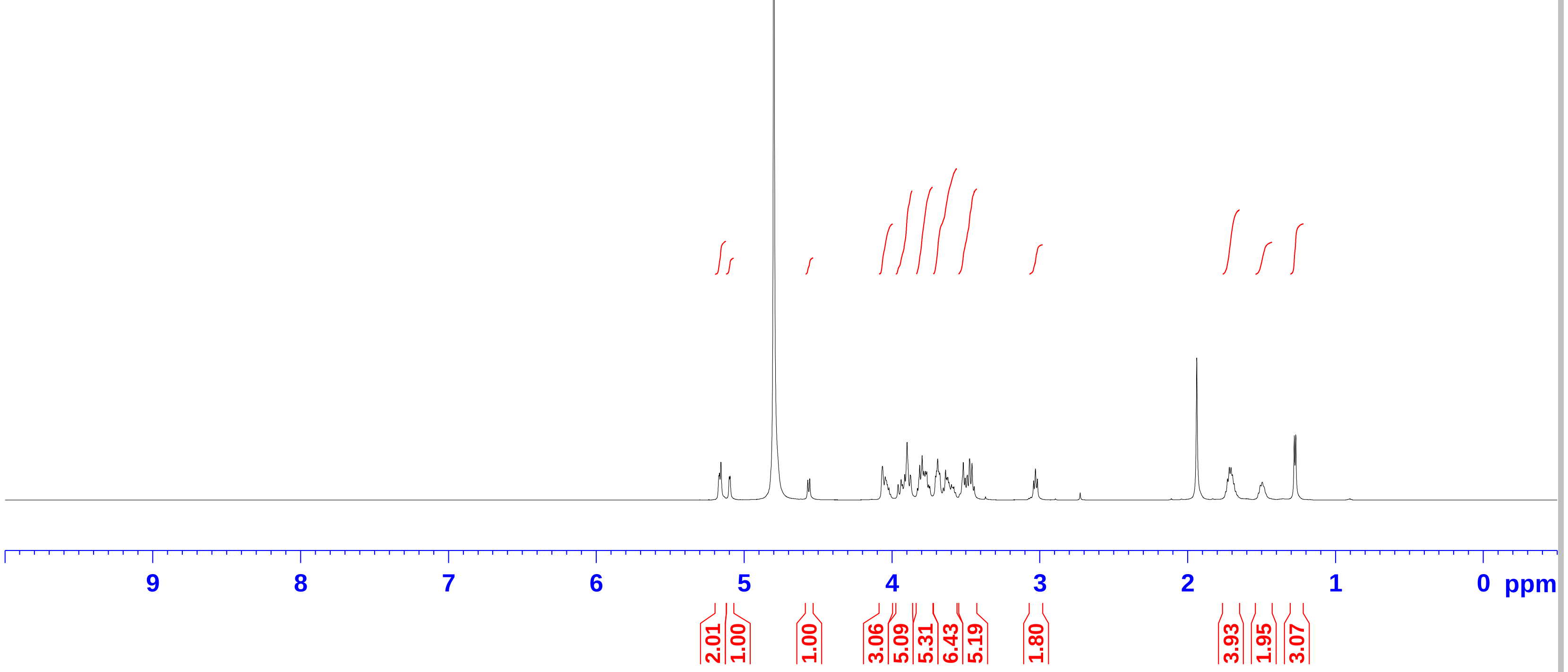

150 MHz 13C{1H} NMR Spectrum of Compound **55** (recorded in D_2O)

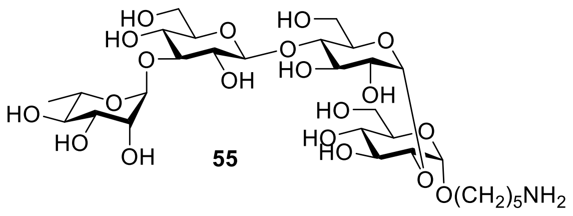

55

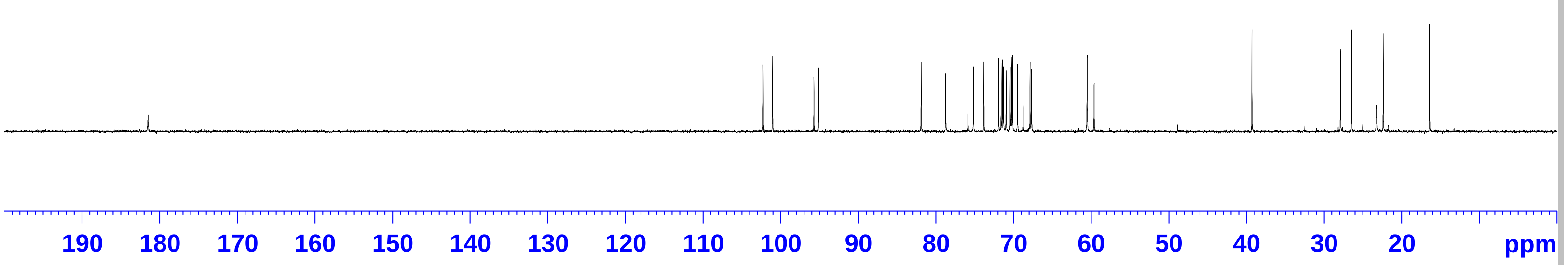

600 MHz ^1H NMR Spectrum of Compound **4** (recorded in D_2O)

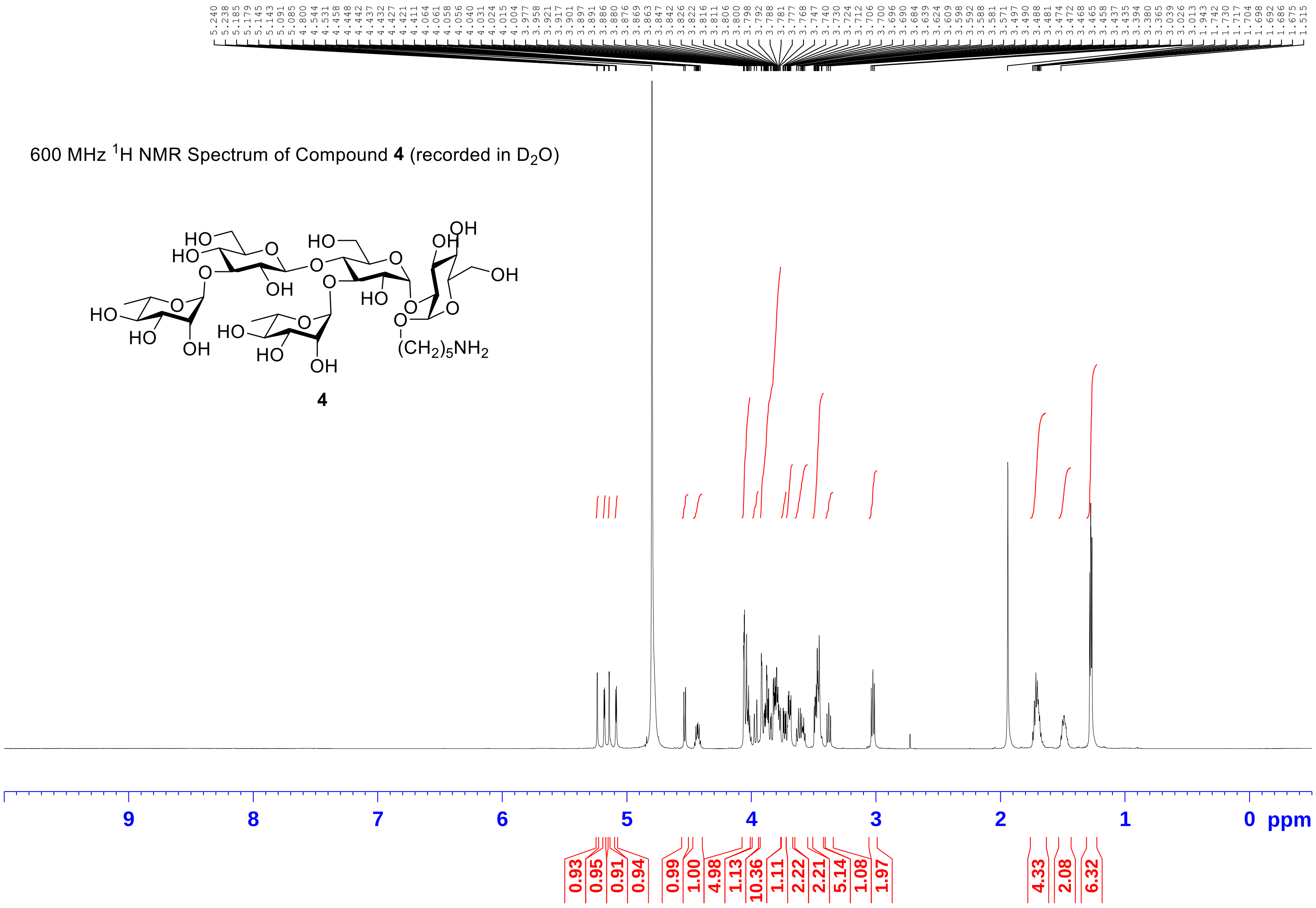

150 MHz 13C{1H} NMR Spectrum of Compound **4** (recorded in D_2O)

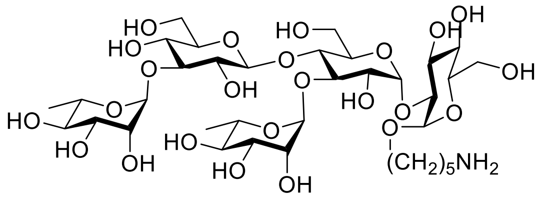

4

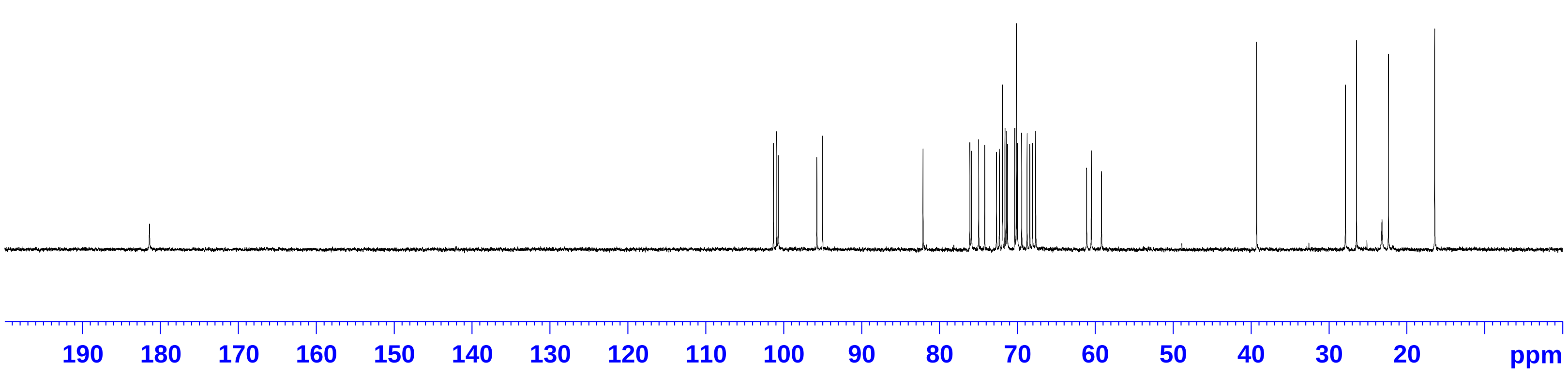

600 MHz ^{1}H NMR Spectrum of Compound **56** (recorded in D_2O)

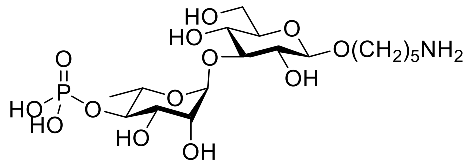

56

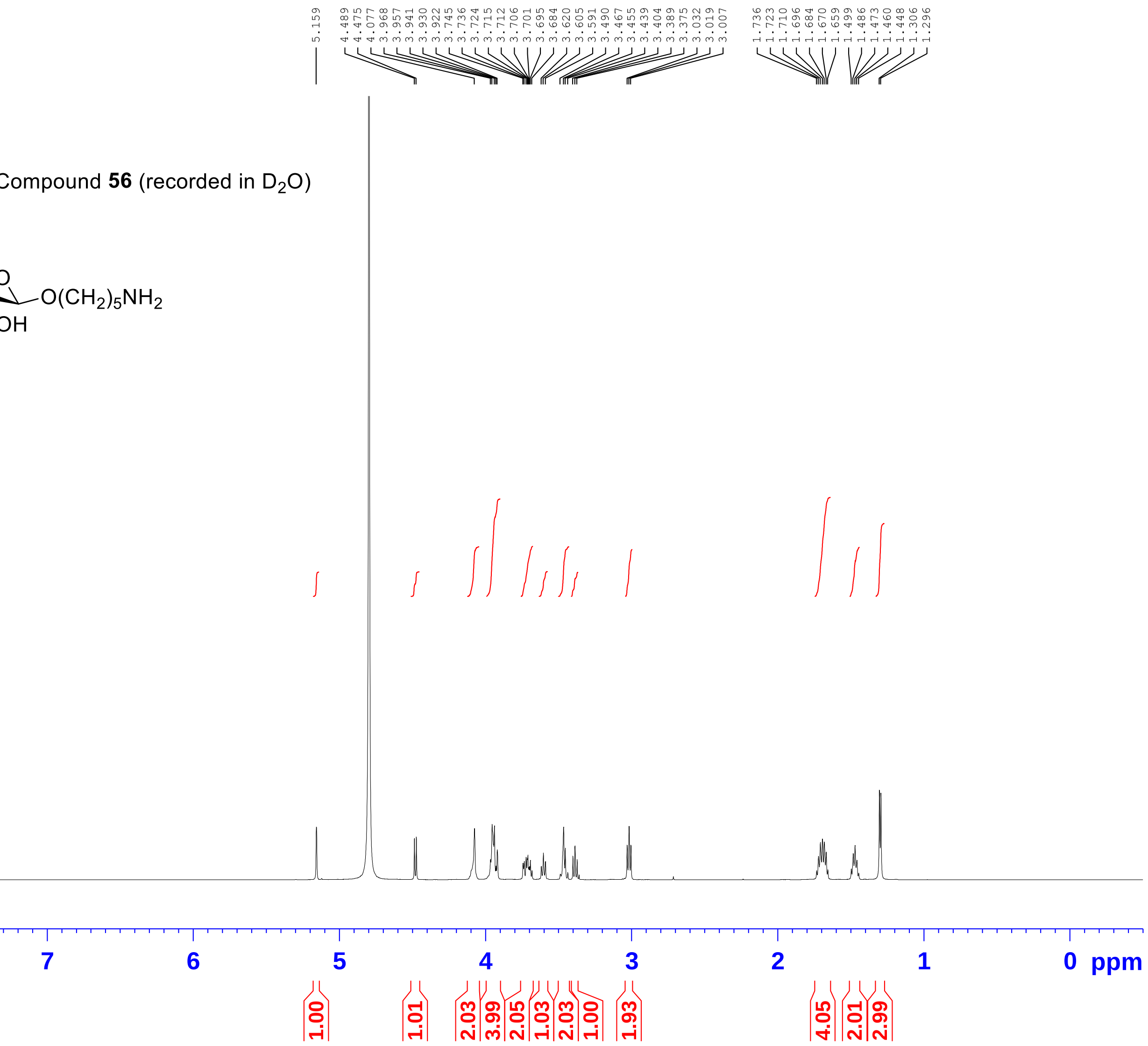

150 MHz 13C{1H} NMR Spectrum of Compound **56** (recorded in D_2O)

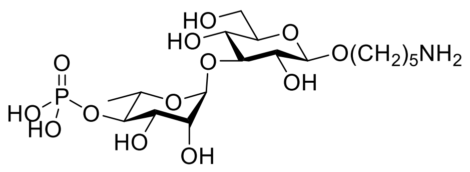

56

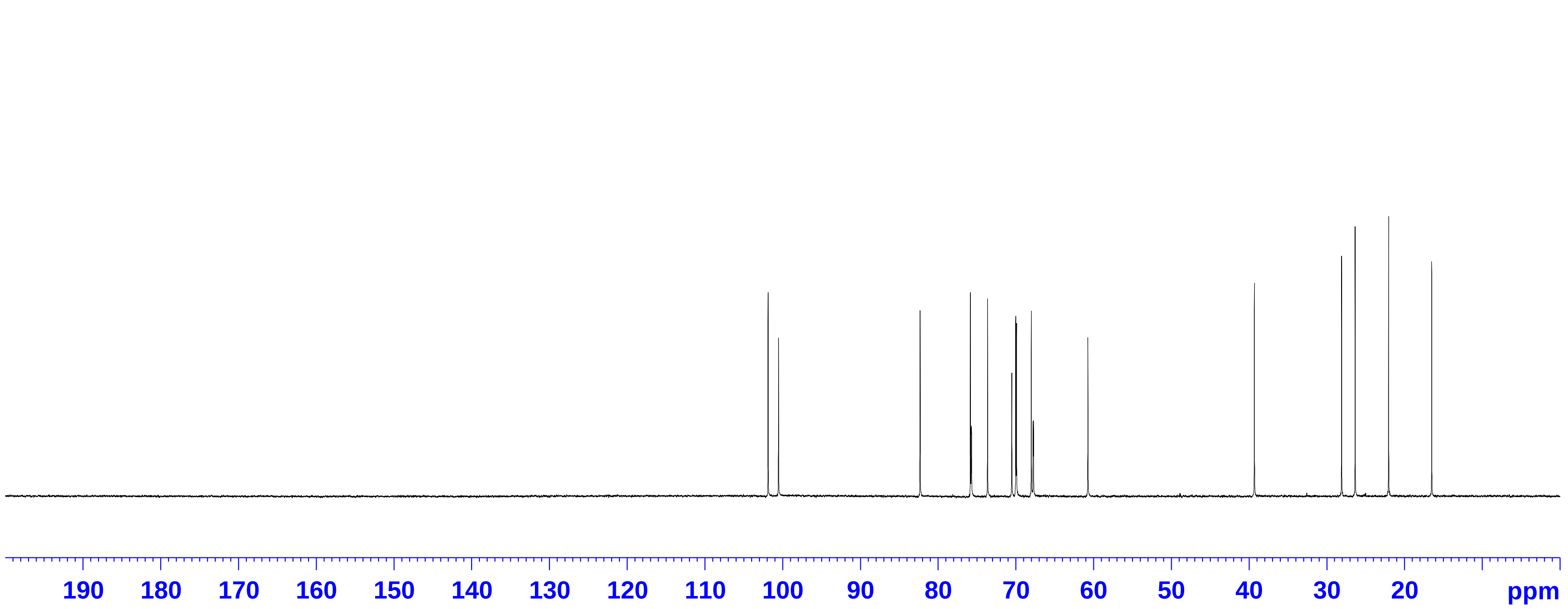

600 MHz ^{1}H NMR Spectrum of Compound **57** (recorded in D_2O)

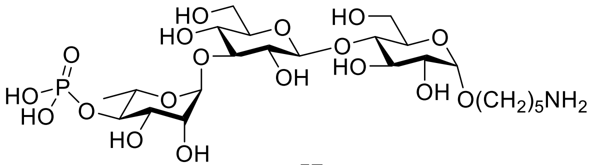

57

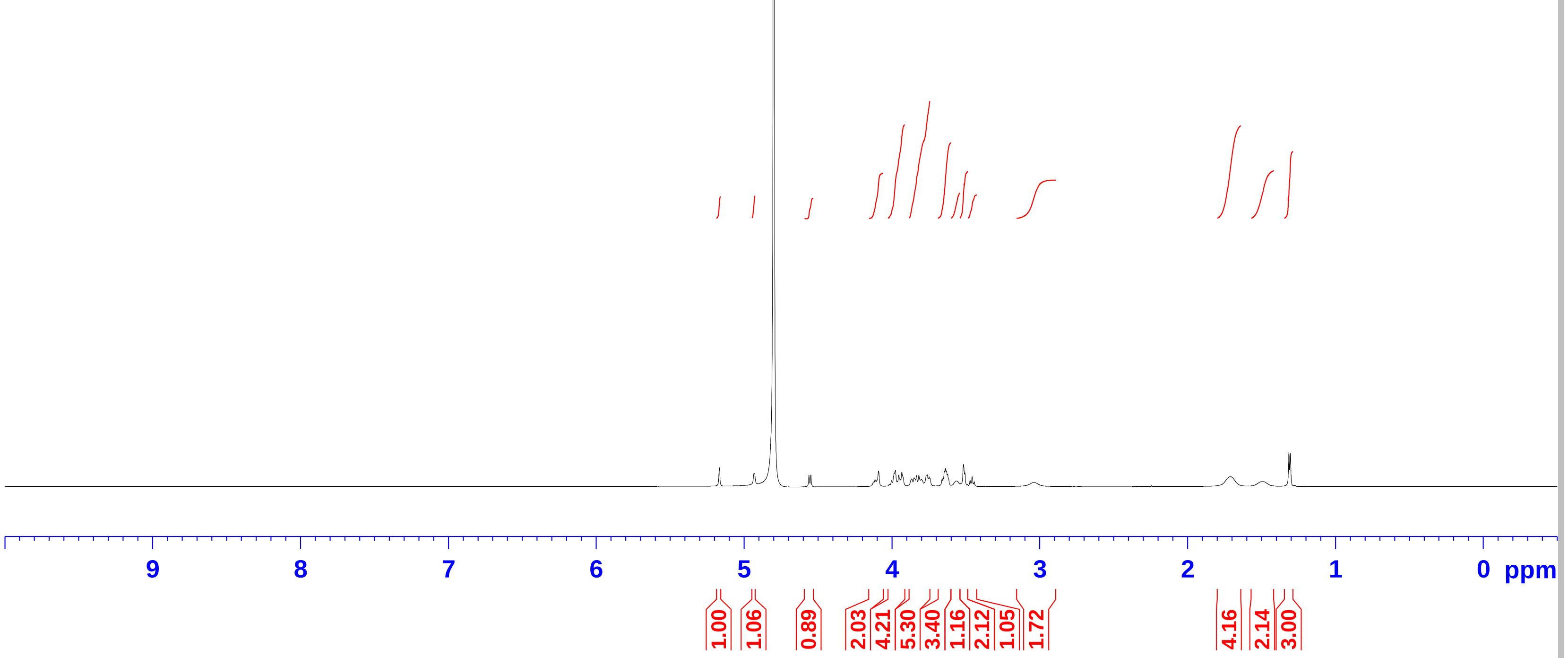

150 MHz 13C{1H} NMR Spectrum of Compound **57** (recorded in D_2O)

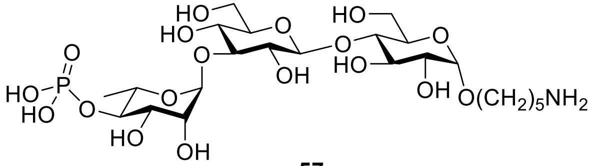

57

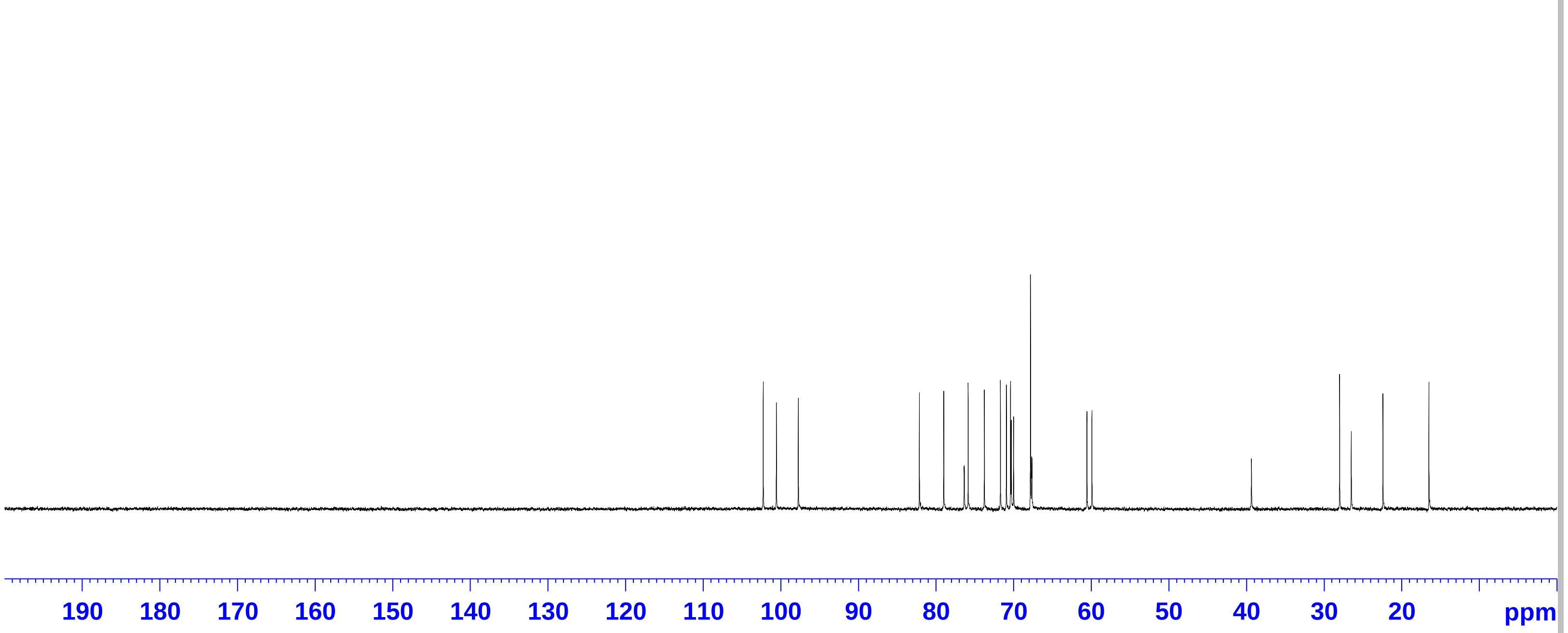

600 MHz ^{1}H NMR Spectrum of Compound **58** (recorded in D_2O)

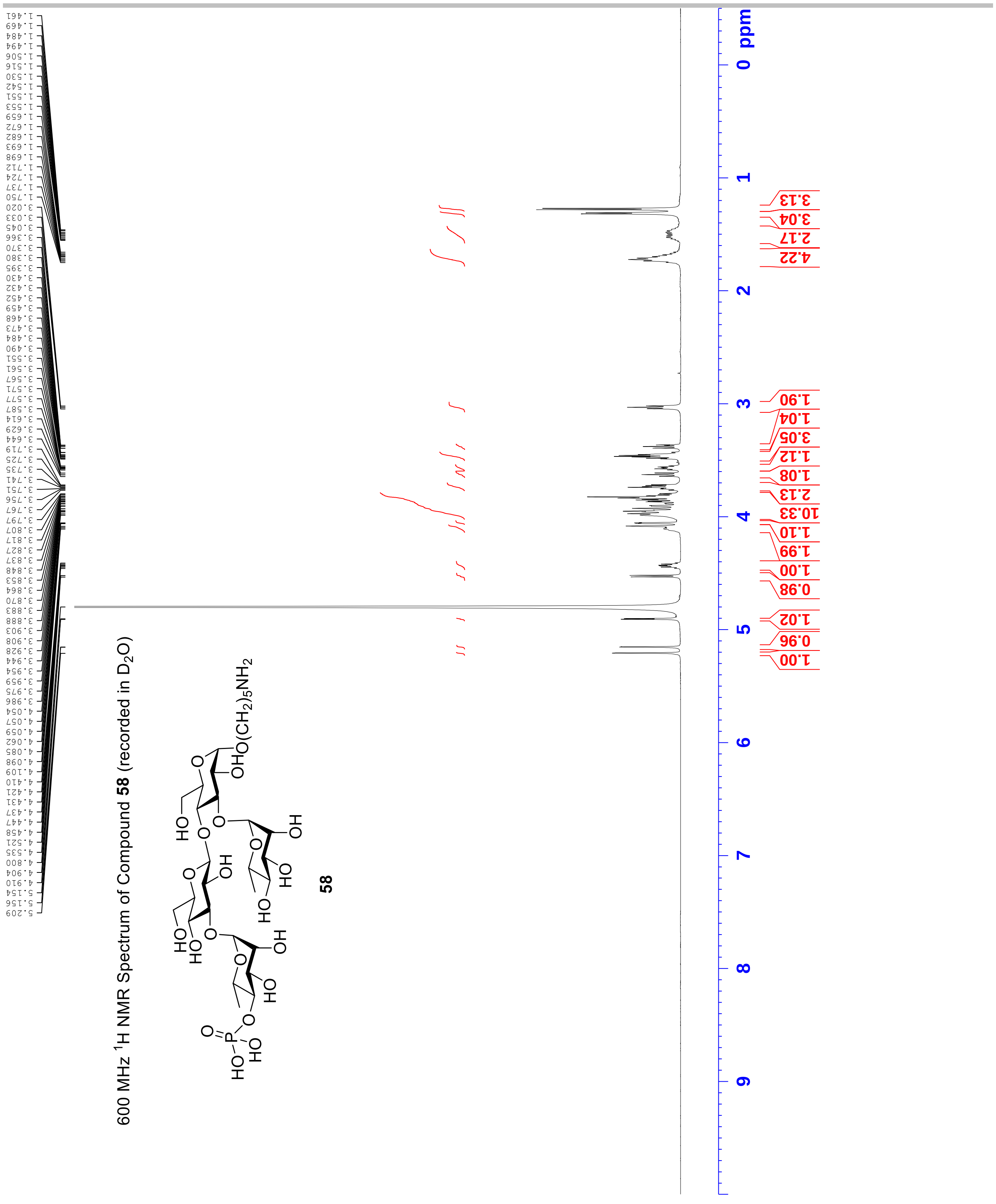

58

150 MHz 13C{1H} NMR Spectrum of Compound **58** (recorded in D_2O)

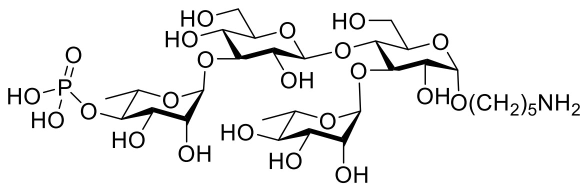

58

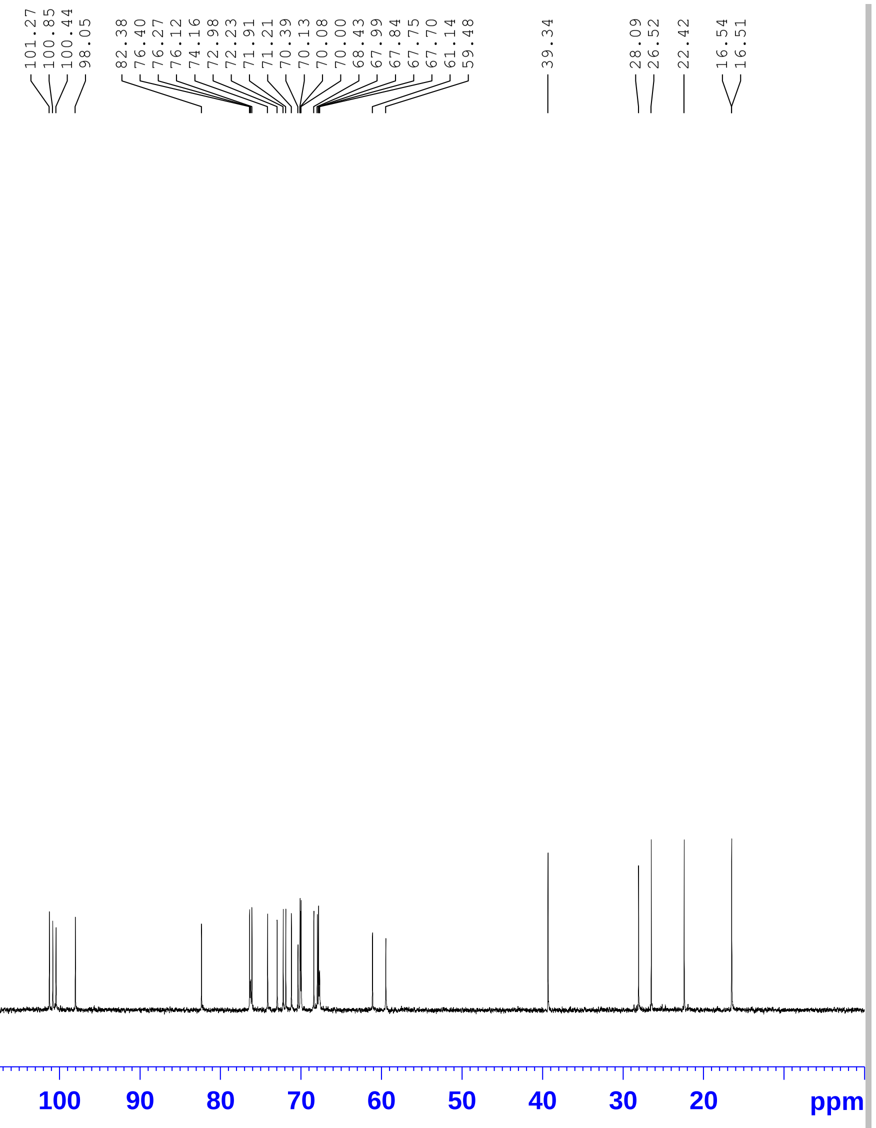

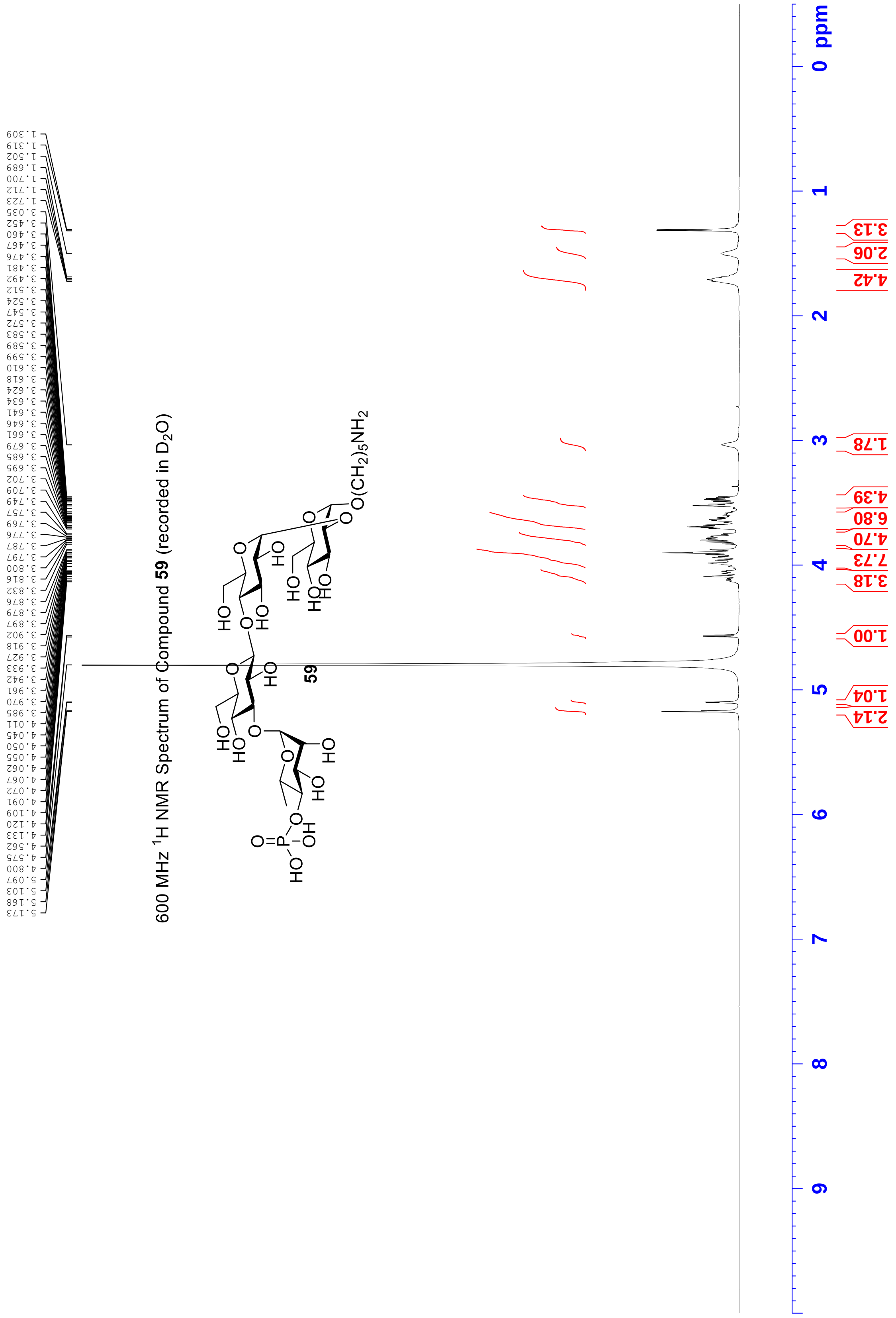

600 MHz ^{1}H NMR Spectrum of Compound 59 (recorded in D_2O)
59
$O(CH_2)_5NH_2$
ppm

150 MHz 13C{1H} NMR Spectrum of Compound **59** (recorded in D_2O)

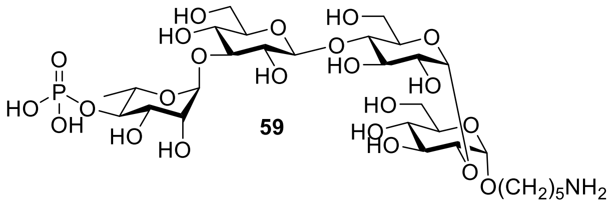

59

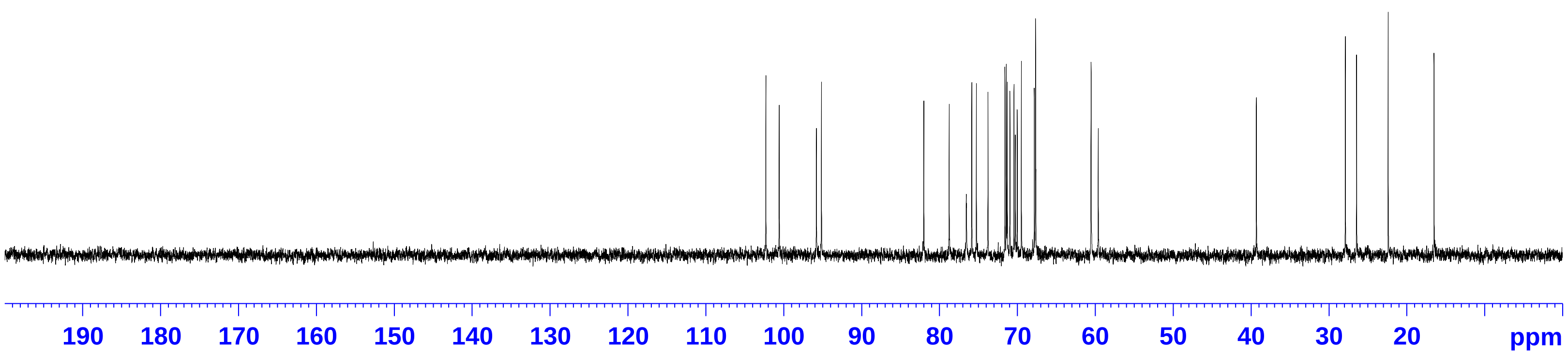

600 MHz ^{1}H NMR Spectrum of Compound **5** (recorded in D_2O)

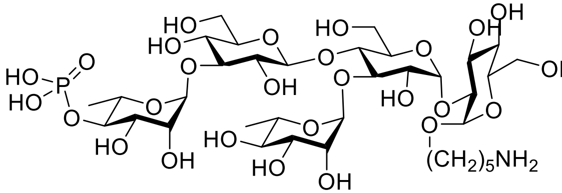

5

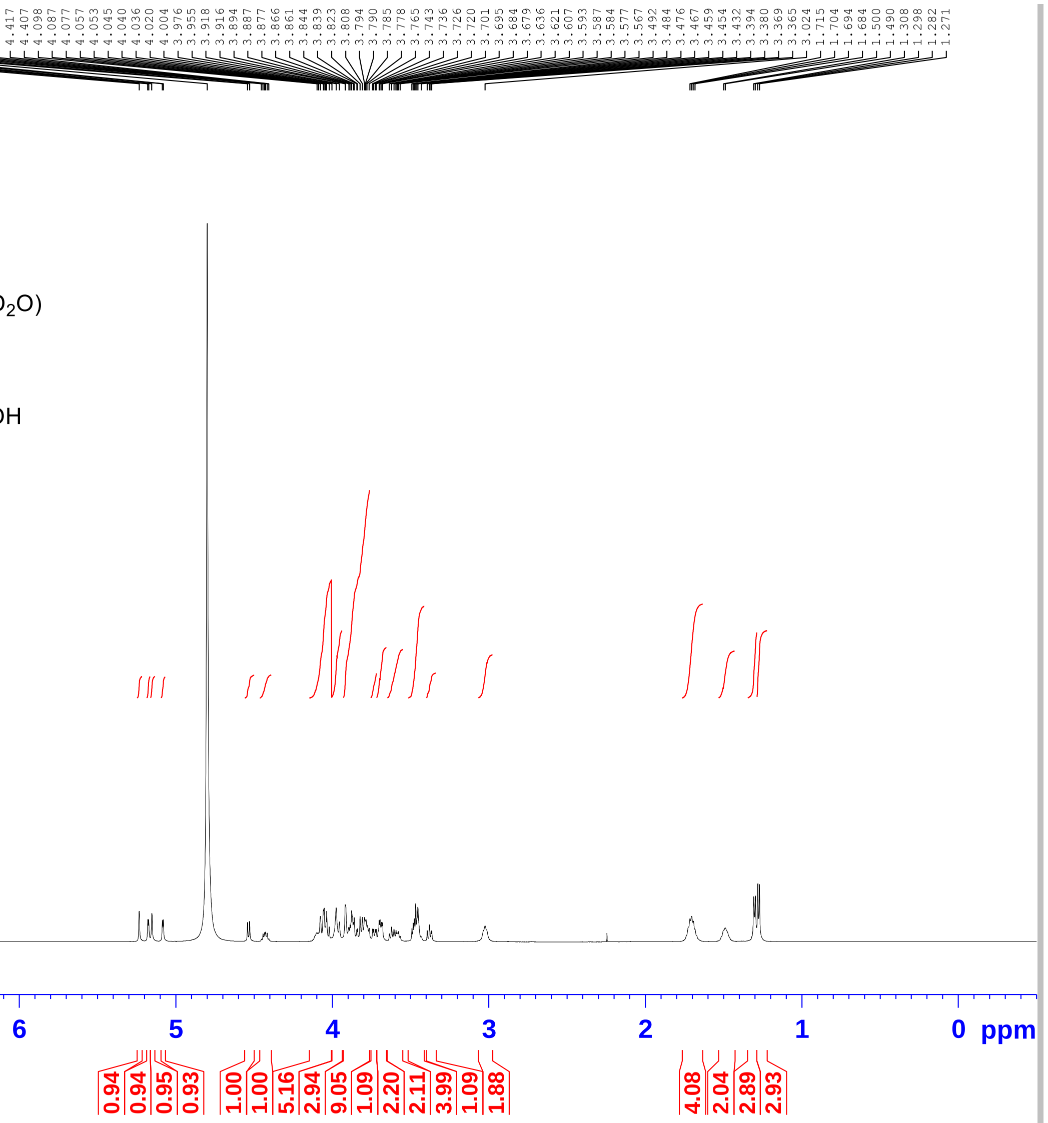

150 MHz 13C{1H} NMR Spectrum of Compound **5** (recorded in D_2O)

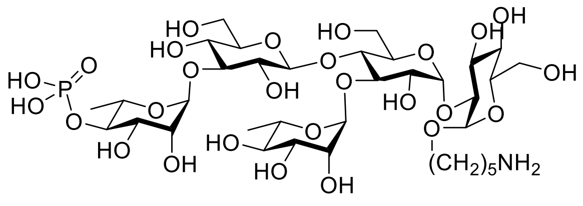

5

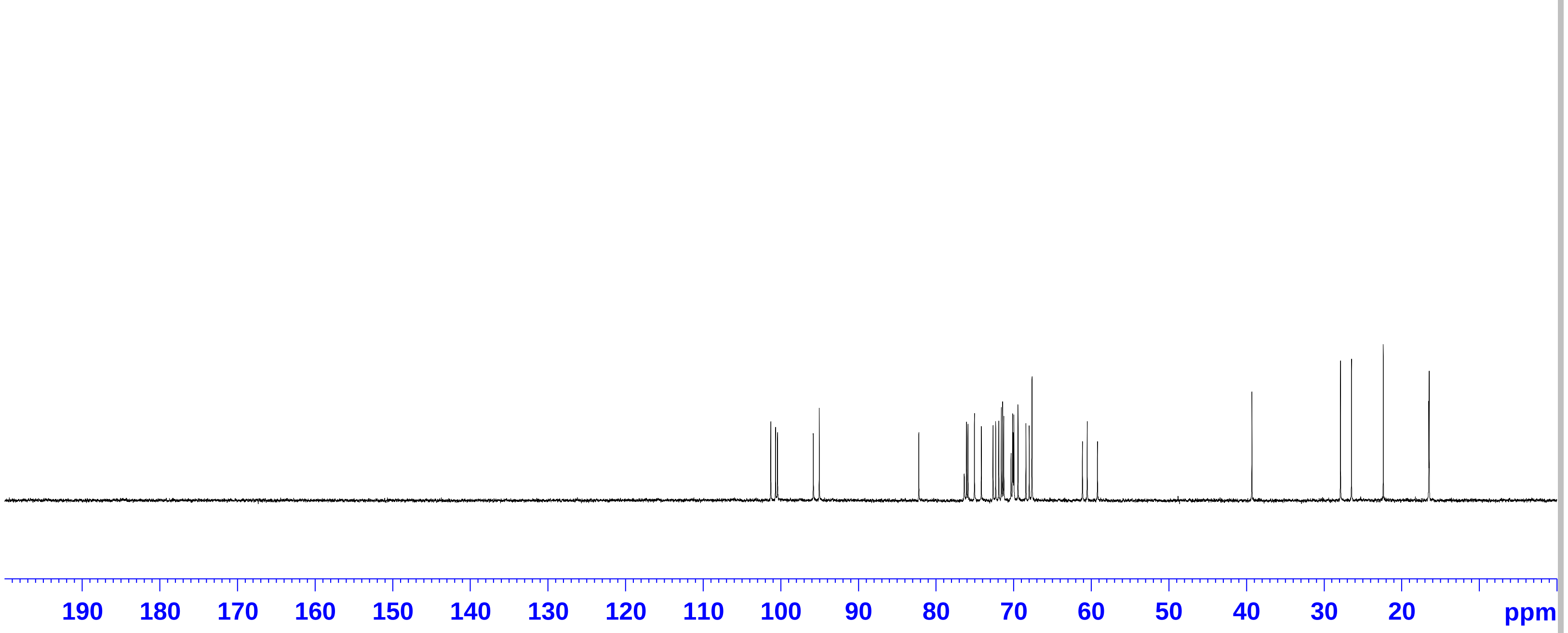

600 MHz ^1H NMR Spectrum of Compound **3** (recorded in D_2O)

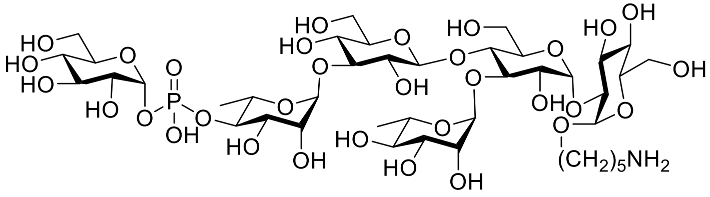

3

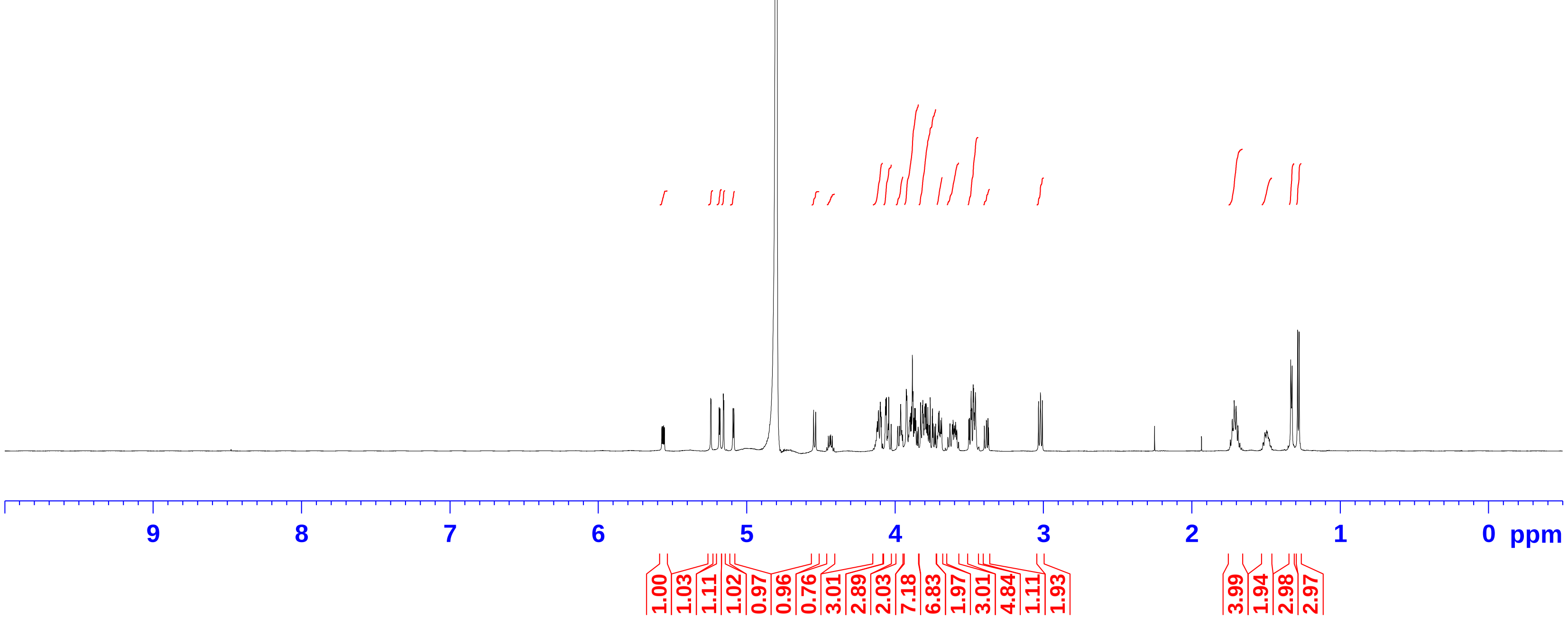

150 MHz 13C{1H} NMR Spectrum of Compound **3** (recorded in D_2O)

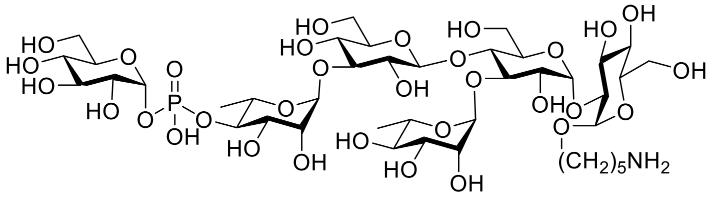

3

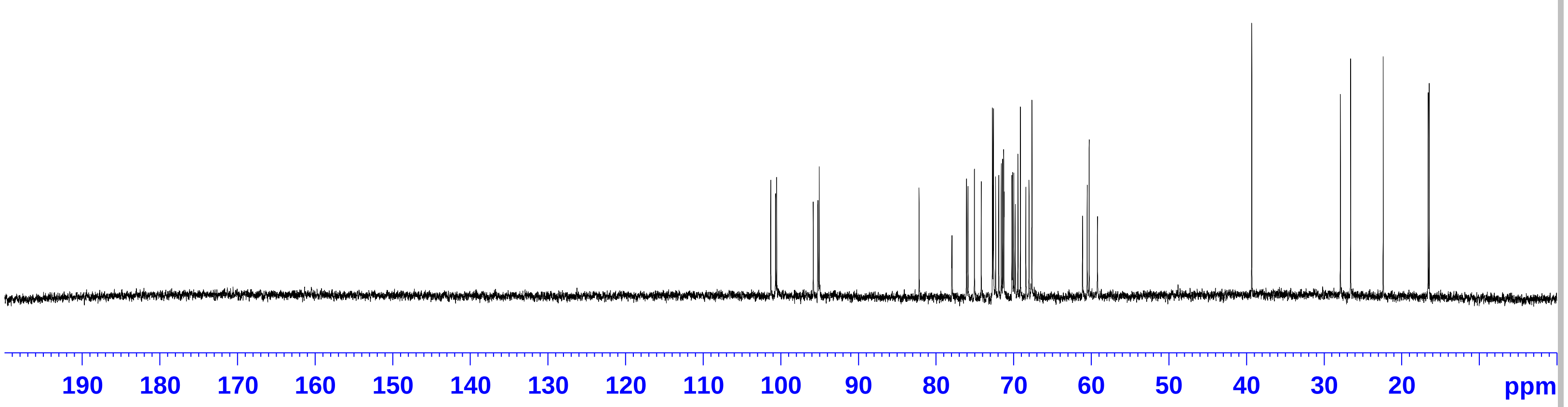

600 MHz ^1H NMR Spectrum of Compound **2** (recorded in D_2O)

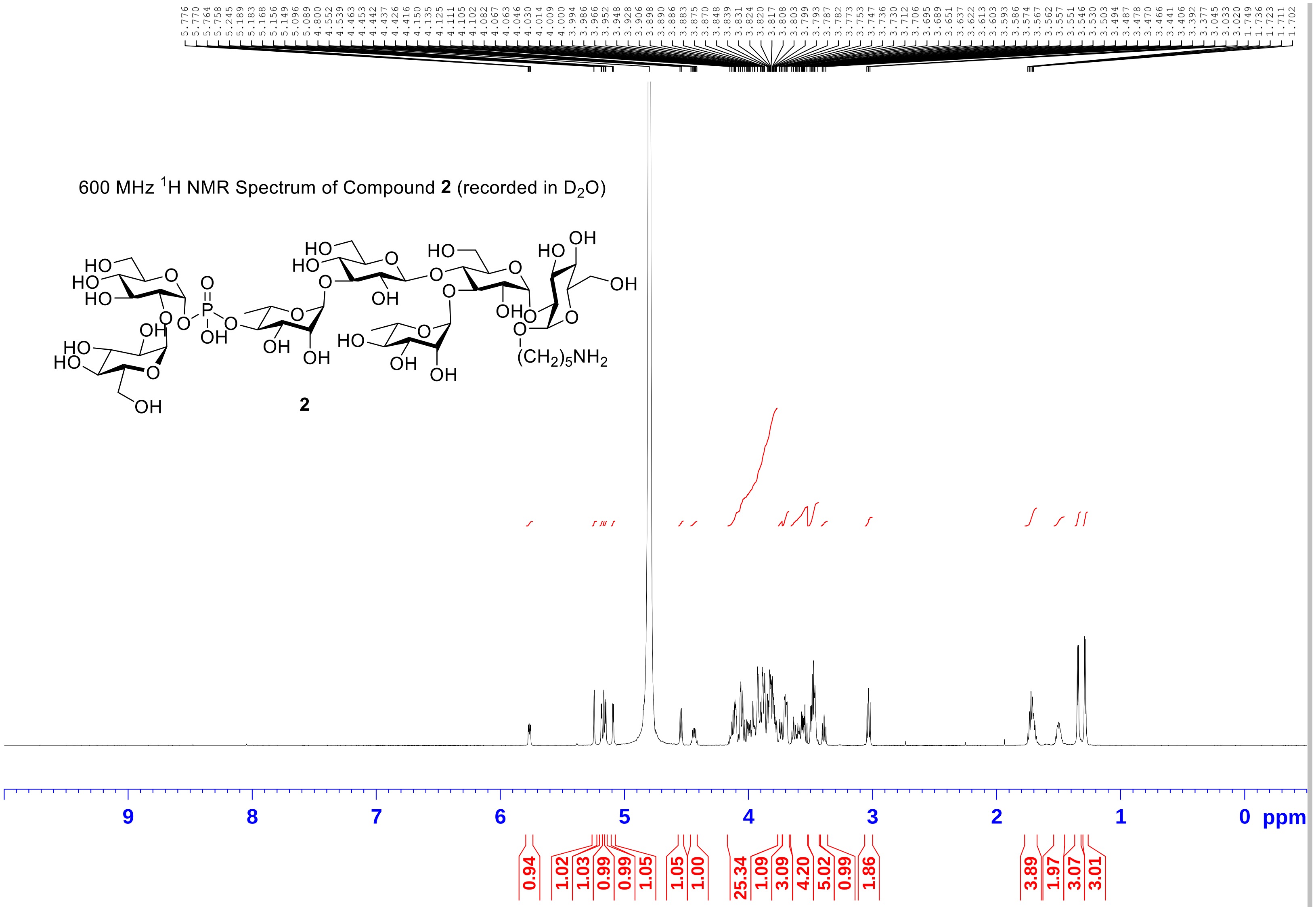

2

150 MHz 13C{1H} NMR Spectrum of Compound **2** (recorded in D_2O)

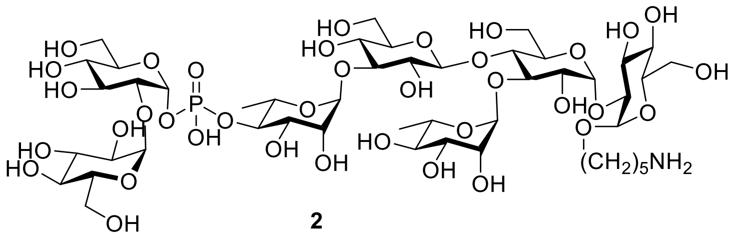

2

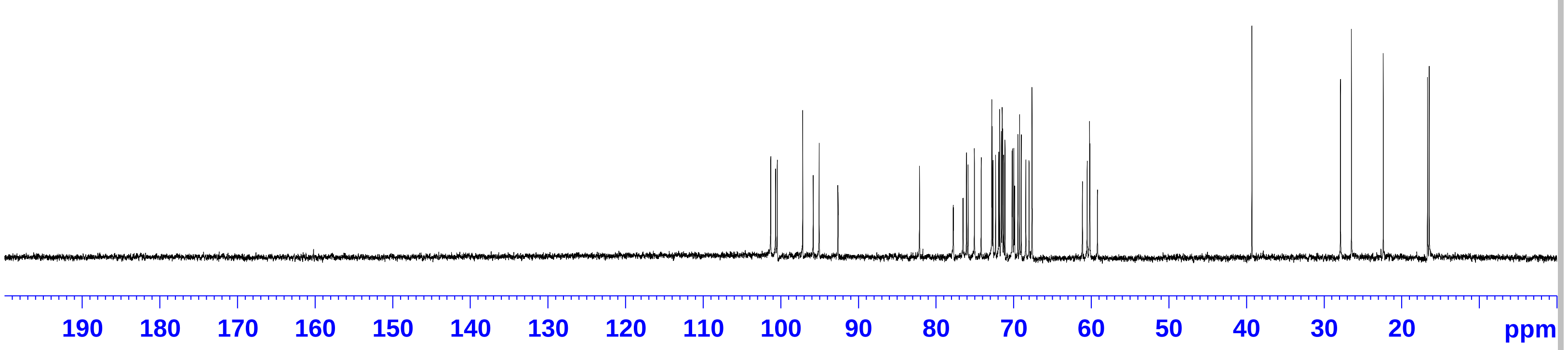

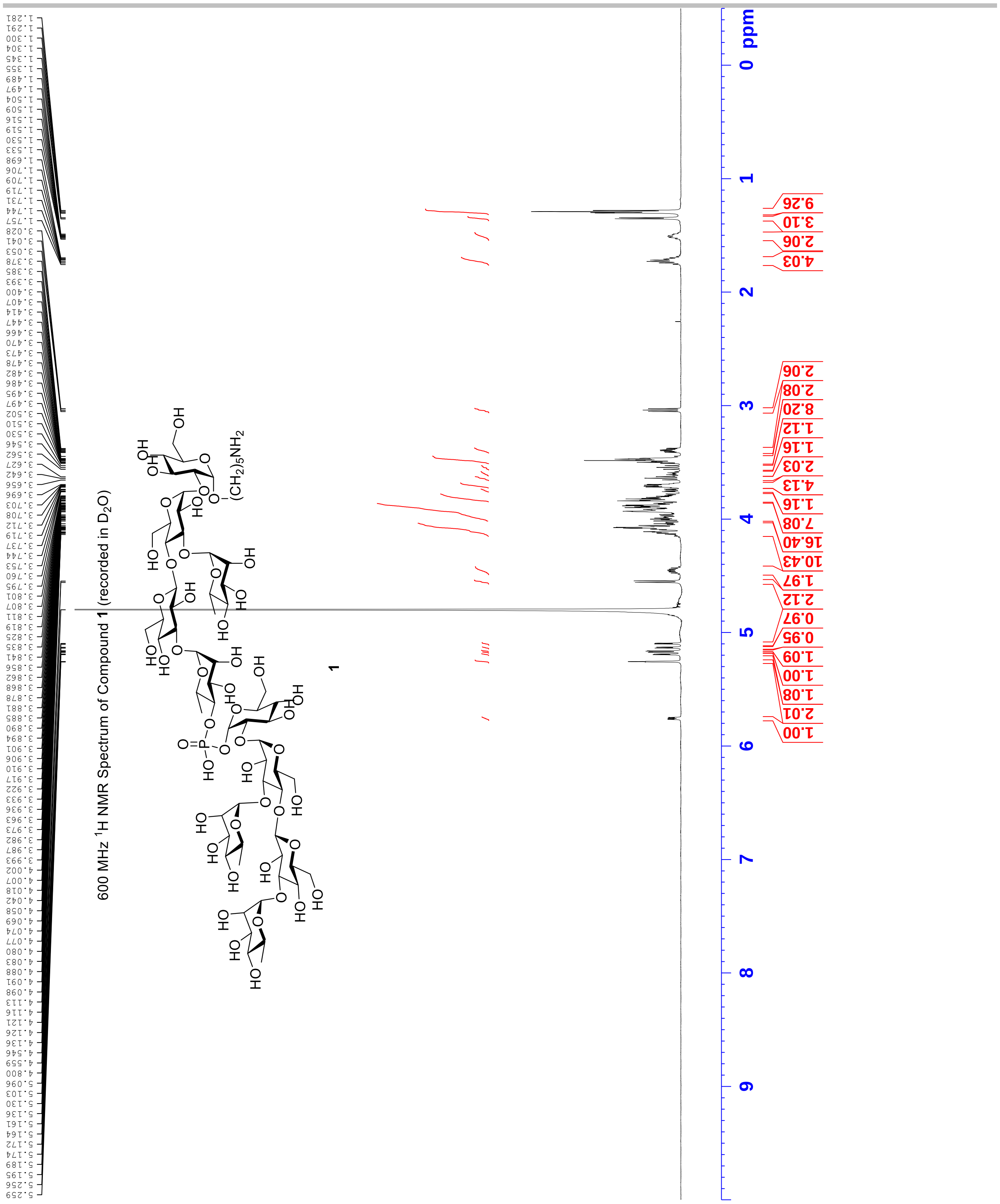
600 MHz ¹H NMR Spectrum of Compound 1 (recorded in D₂O)
1
ppm

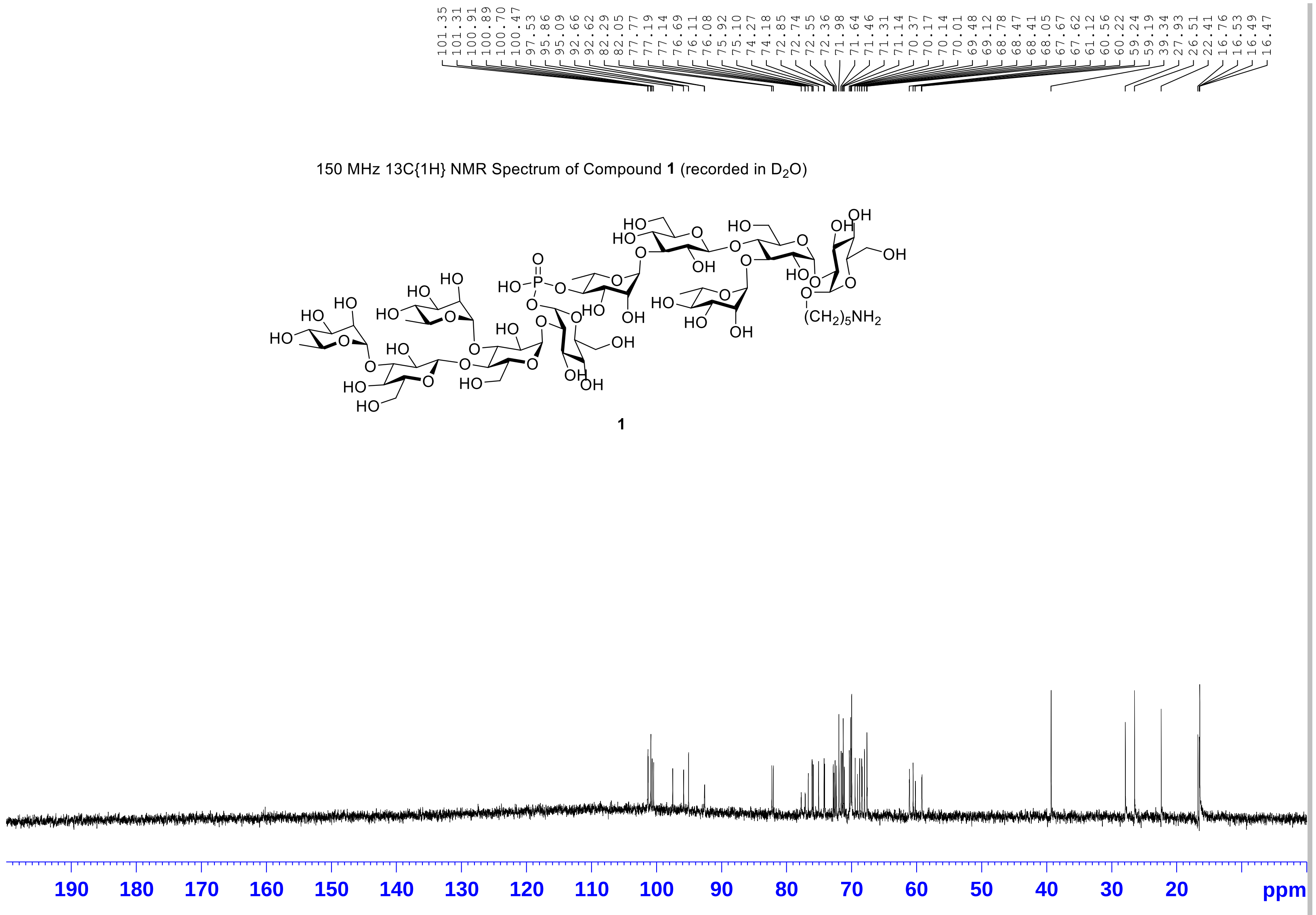

150 MHz 13C{1H} NMR Spectrum of Compound **1** (recorded in D_2O)

600 MHz ^{1}H NMR Spectrum of Compound **60** (recorded in D_2O)

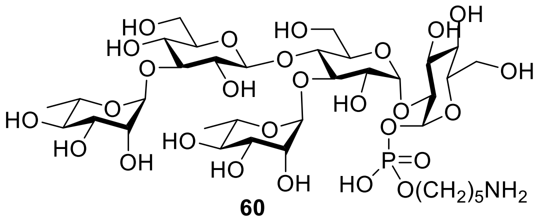

60

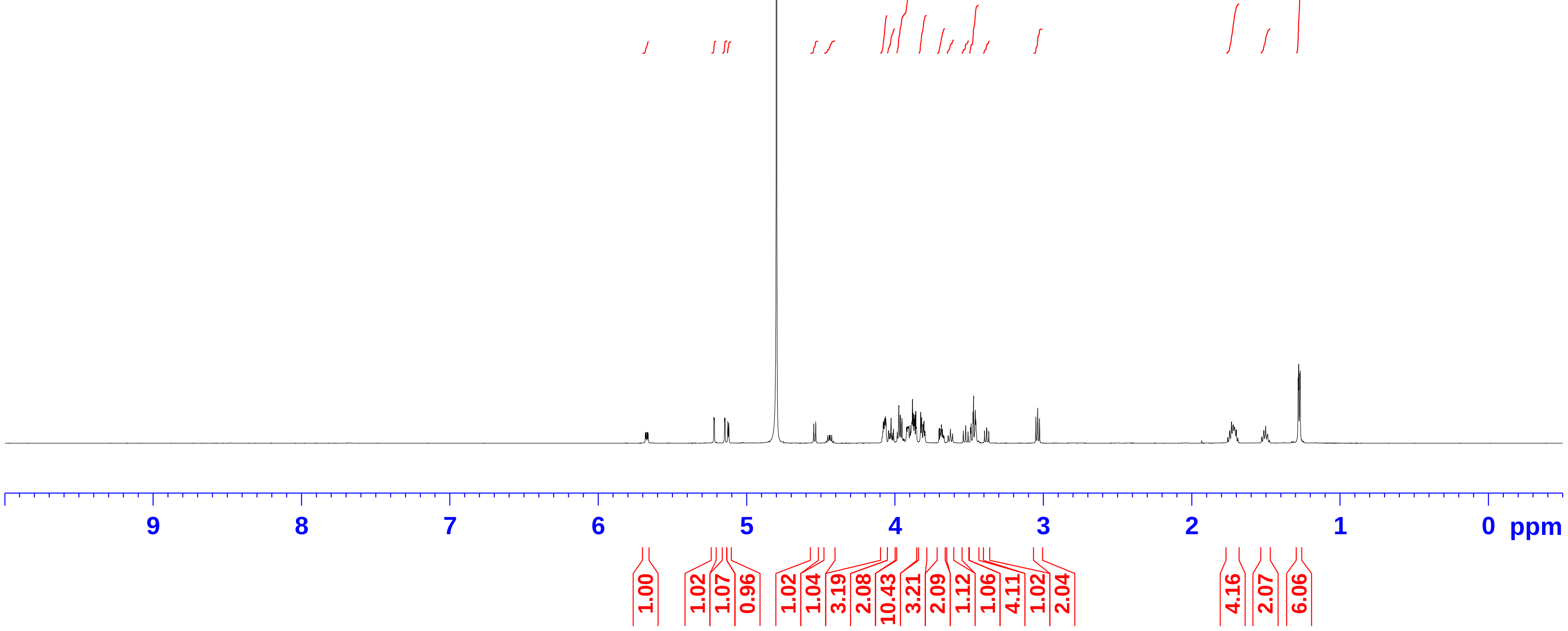

150 MHz 13C{1H} NMR Spectrum of Compound **60** (recorded in D_2O)

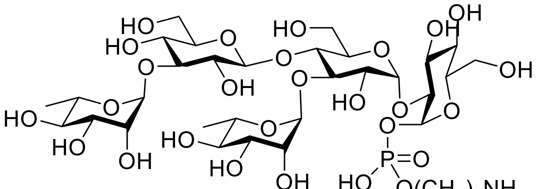

60

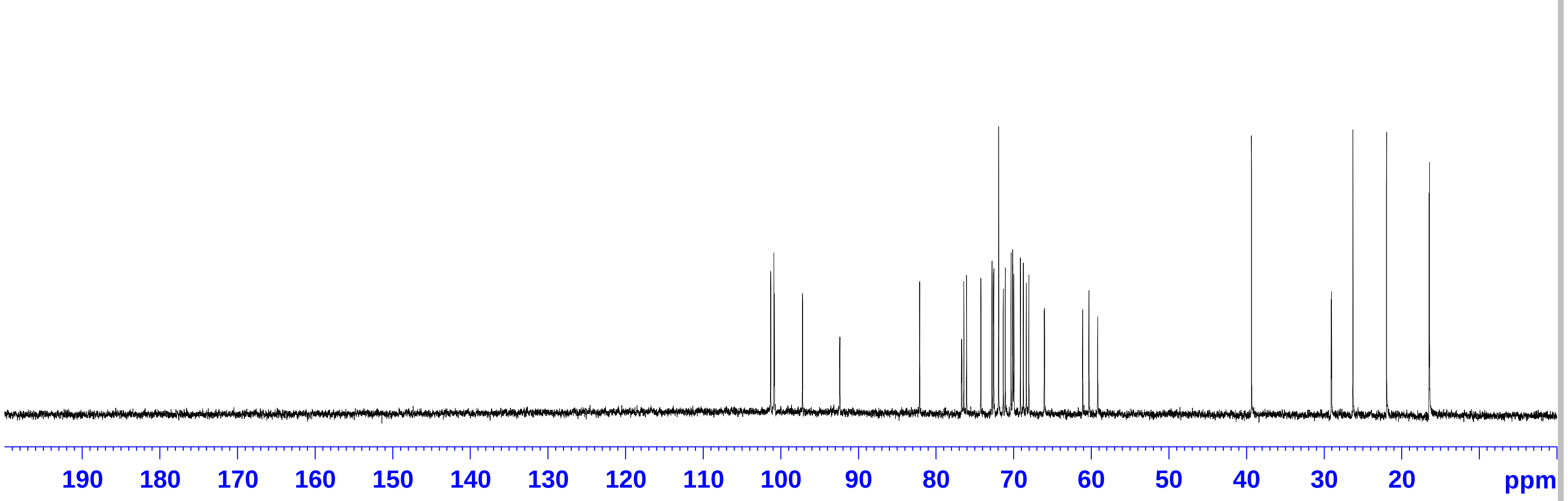

600 MHz ^{1}H NMR Spectrum of Compound **61** (recorded in $CDCl_3$)

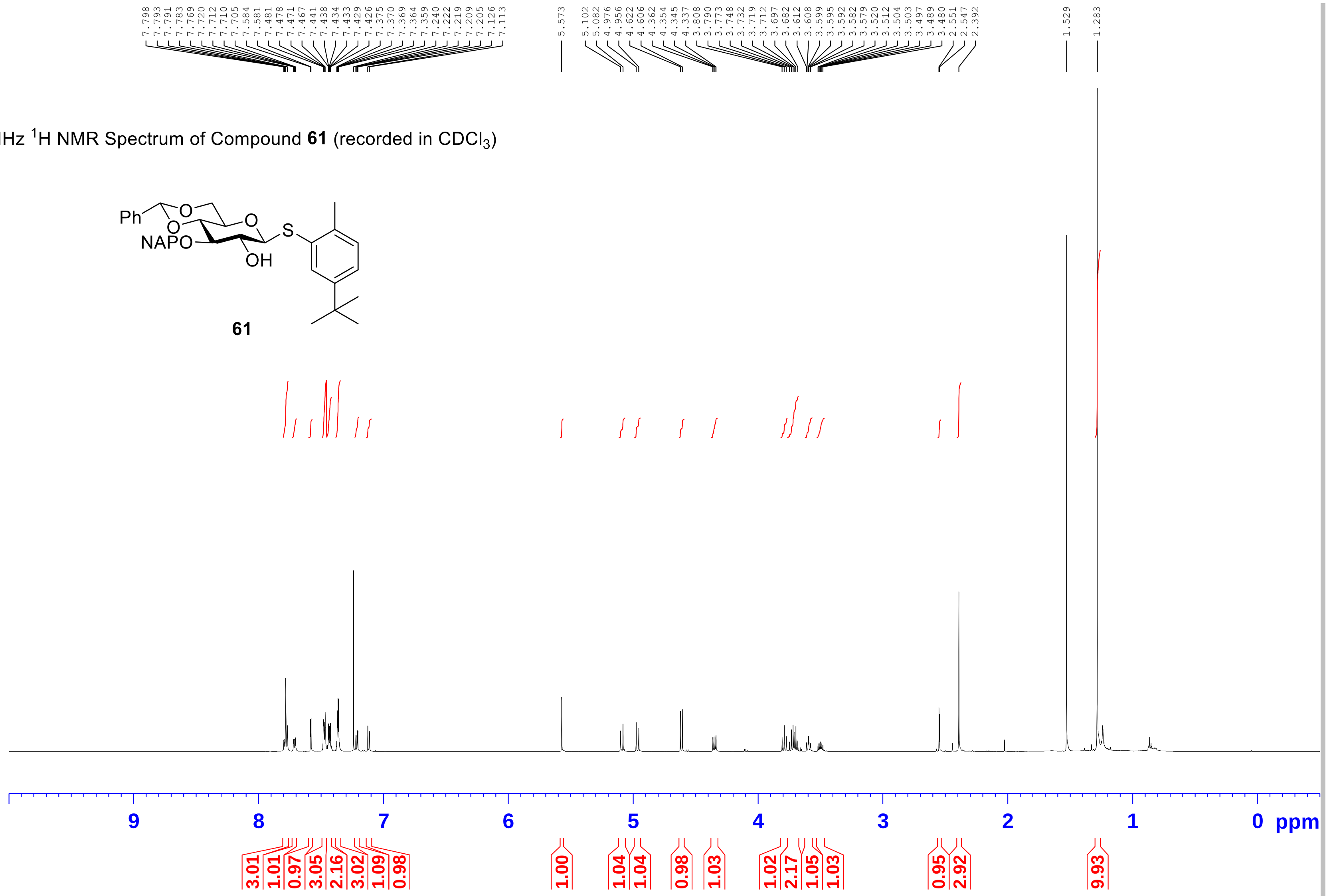

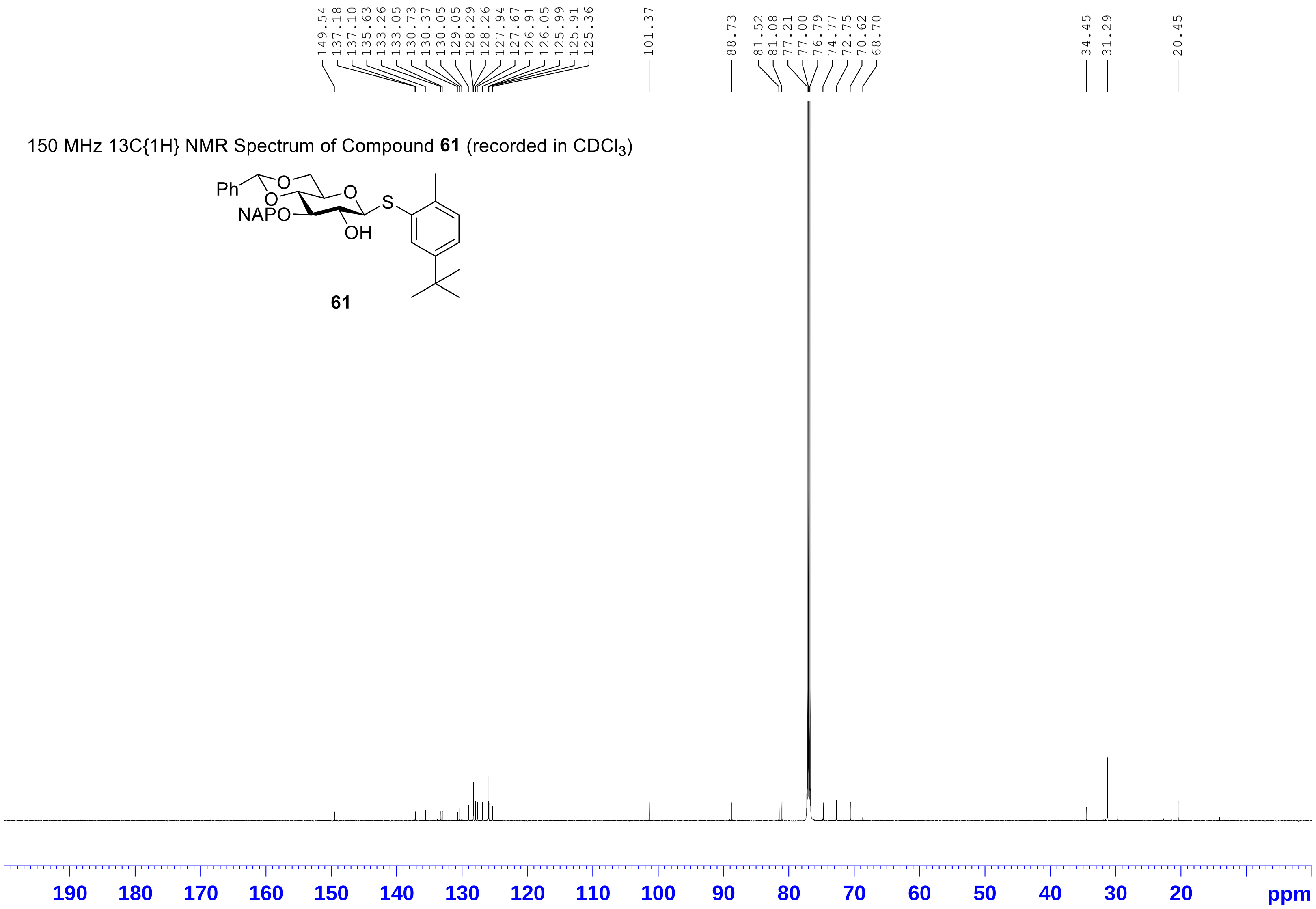

150 MHz 13C{1H} NMR Spectrum of Compound **61** (recorded in $CDCl_3$)

61

7.709 7.696 7.631 7.607 7.606 7.604 7.593 7.591 7.585 7.581 7.578 7.574 7.572 7.569 7.564 7.551 7.490 7.476 7.462 7.459 7.457 7.454 7.451 7.448 7.445 7.443 7.441 7.438 7.434 7.431 7.429 7.427 7.424 7.419 7.415 7.412 7.411 7.403 7.401 7.399 7.391 7.388 7.274 7.272 7.260 7.258 7.240 7.229 7.225 7.216 7.212 7.097 7.083 5.679 5.472 5.457 5.455 5.441 5.029 5.008 4.909 4.888 4.835 4.818 4.443 4.434 4.425 4.417 4.007 3.991 3.977 3.971 3.957 3.948 3.941 3.930 3.913 3.608 3.600 3.593 3.592 3.585 3.577 3.568 2.215 1.299

600 MHz ^{1}H NMR Spectrum of Compound **62** (recorded in $CDCl_3$)

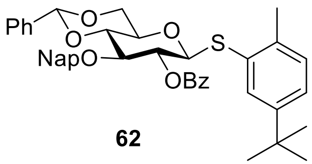

62

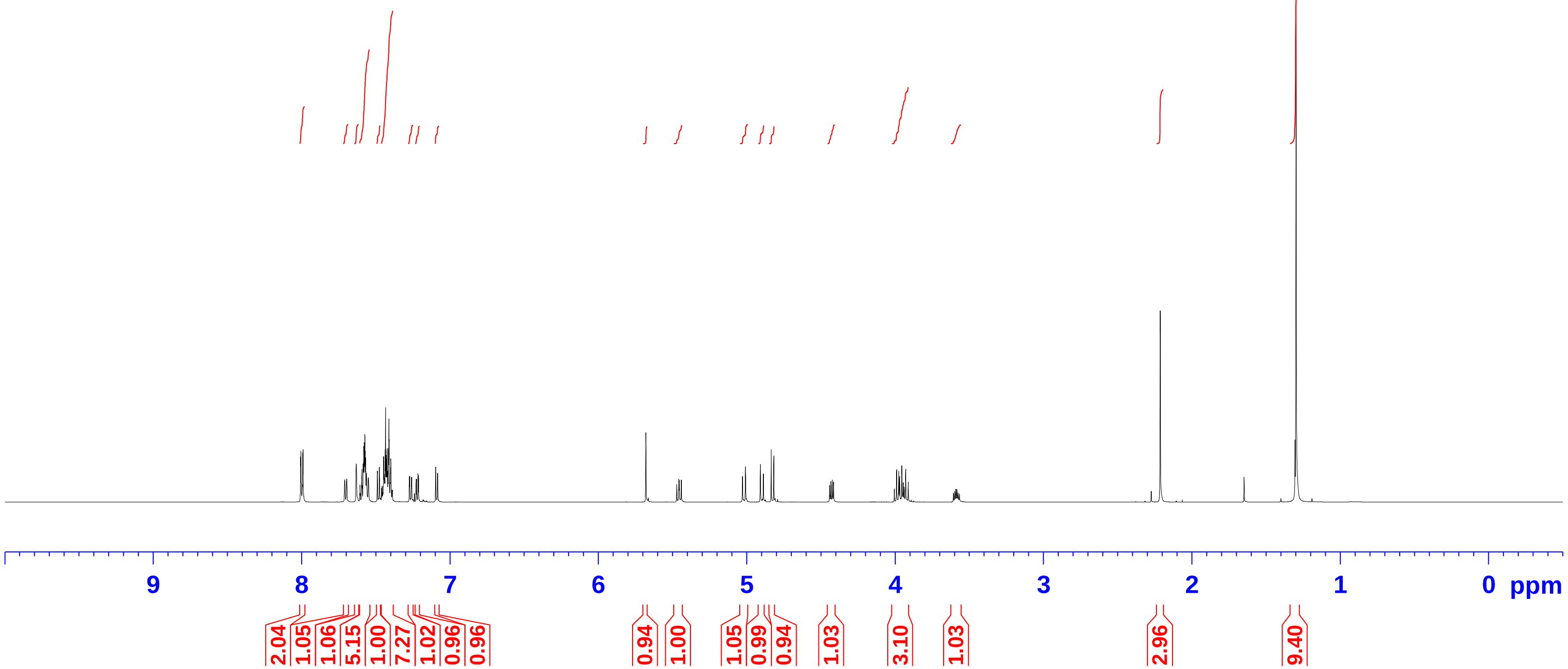

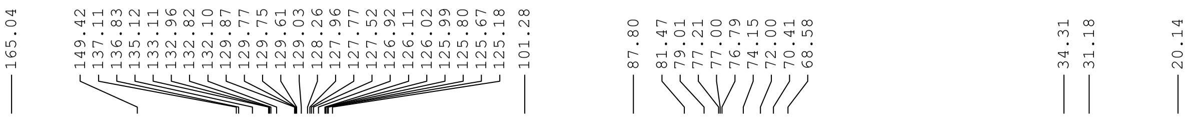

150 MHz 13C{1H} NMR Spectrum of Compound **62** (recorded in $CDCl_3$)

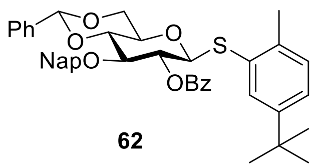

62

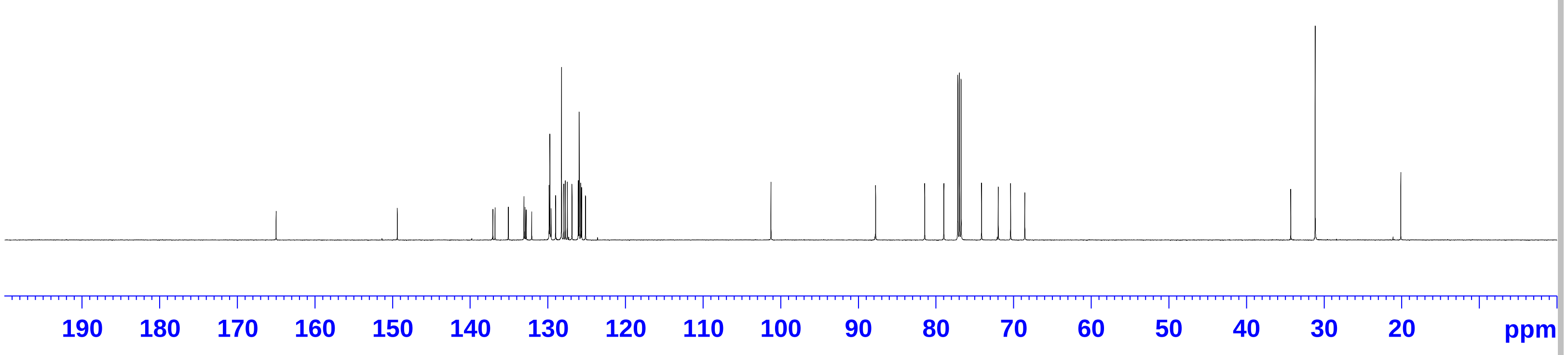

600 MHz ^{1}H NMR Spectrum of Compound **63** (recorded in $CDCl_3$)

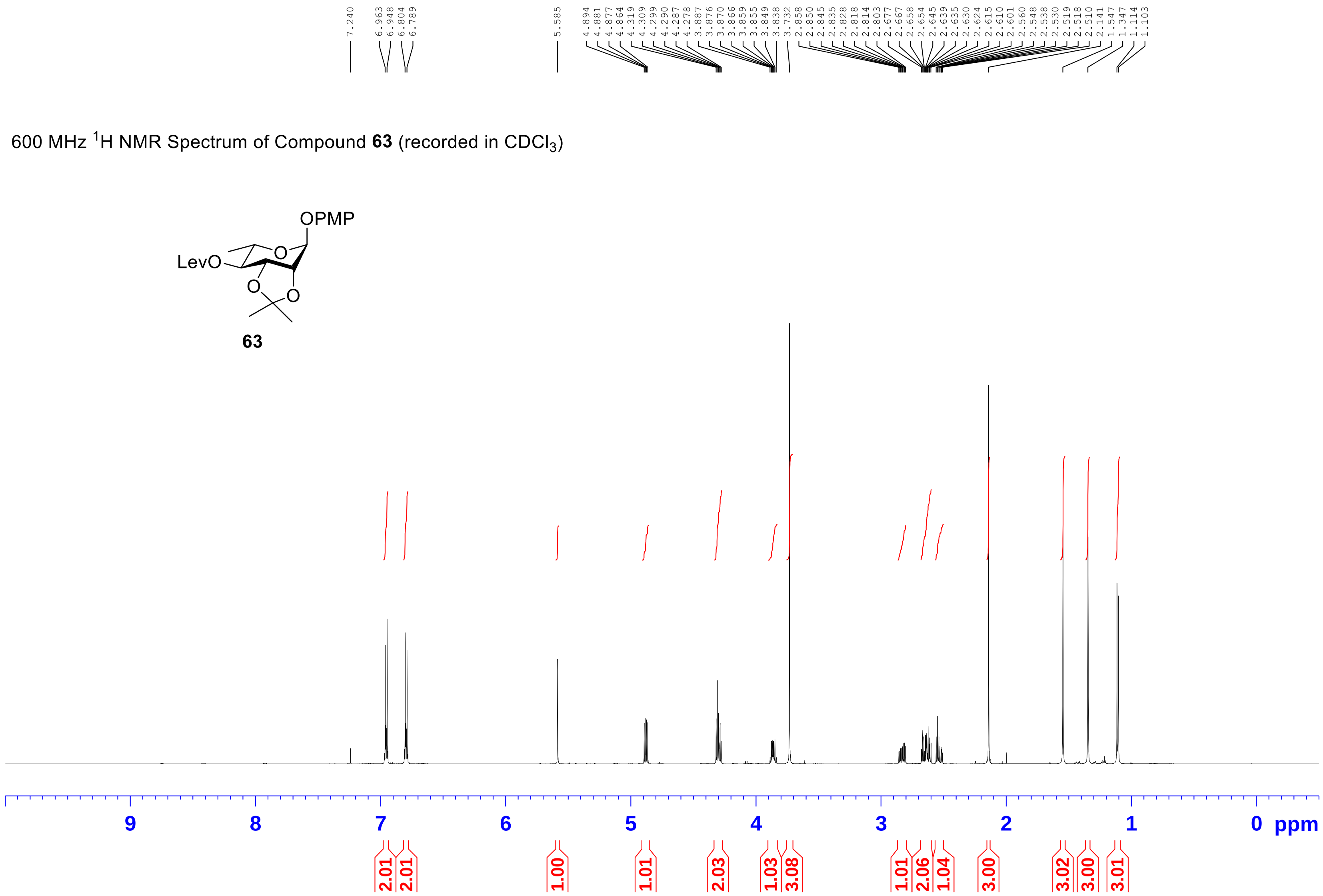

150 MHz 13C{1H} NMR Spectrum of Compound **63** (recorded in $CDCl_3$)

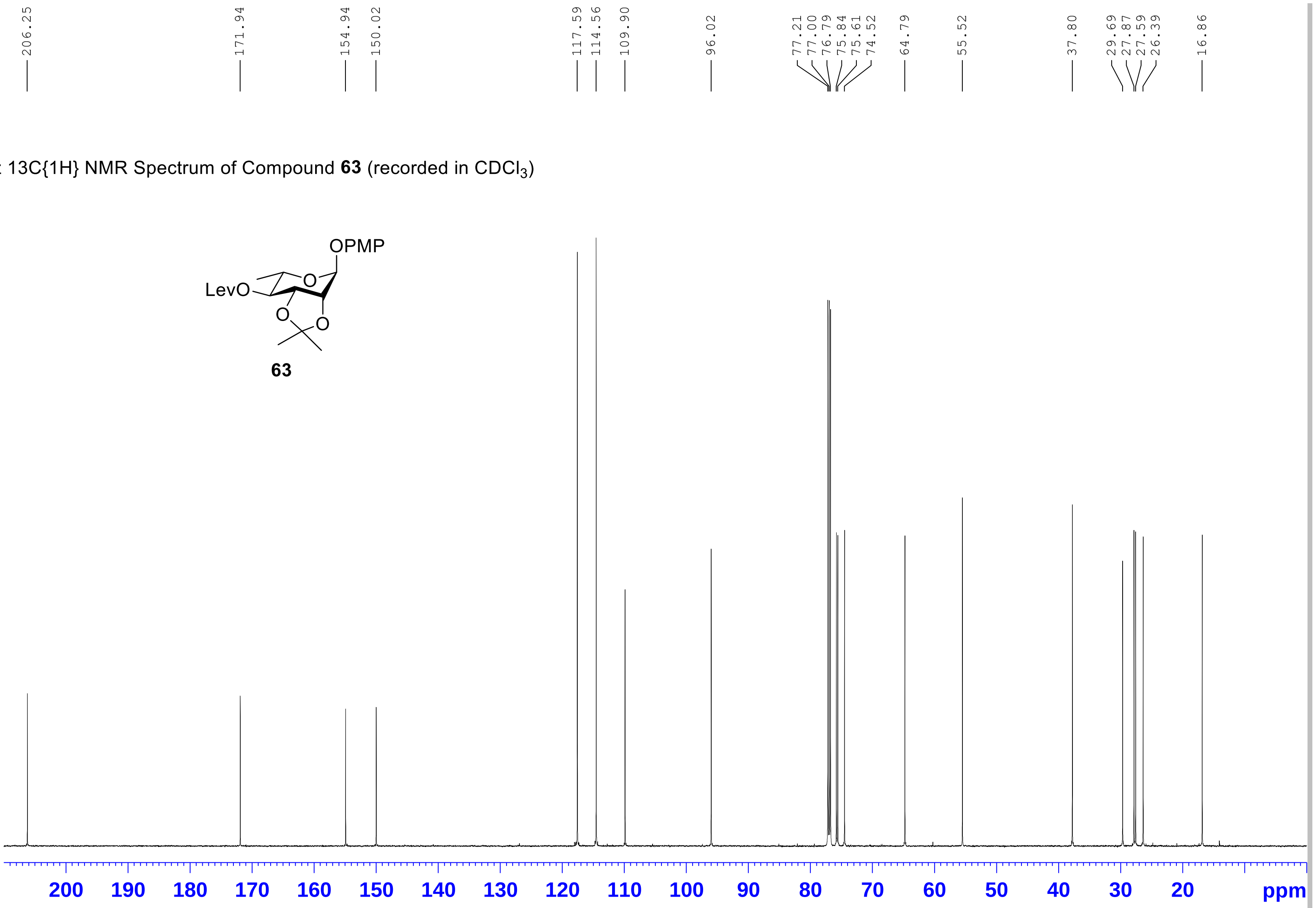

600 MHz ^{1}H NMR Spectrum of Compound **64** (recorded in $CDCl_3$)

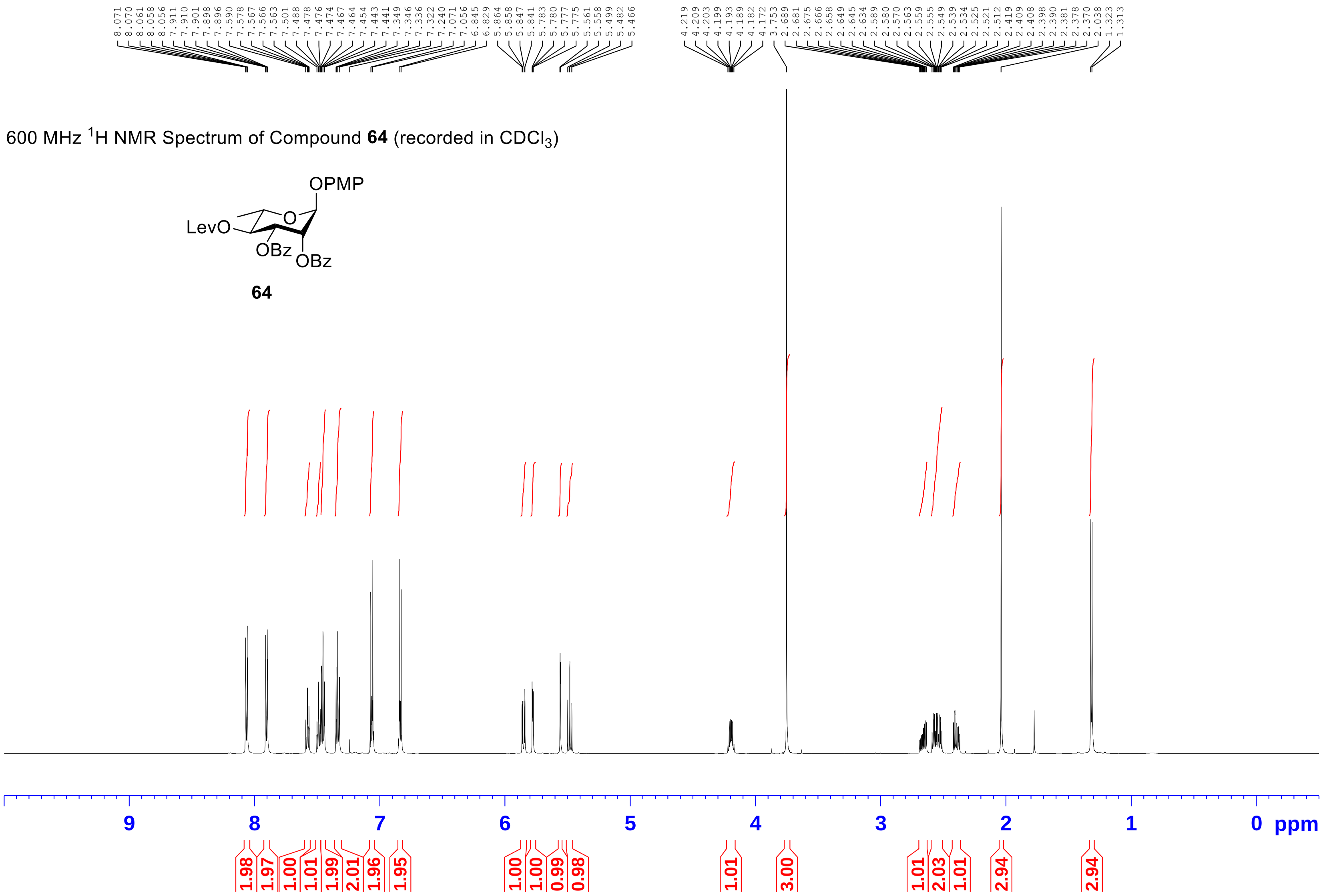

150 MHz 13C{1H} NMR Spectrum of Compound **64** (recorded in $CDCl_3$)

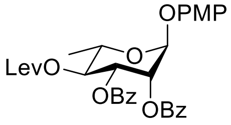

64

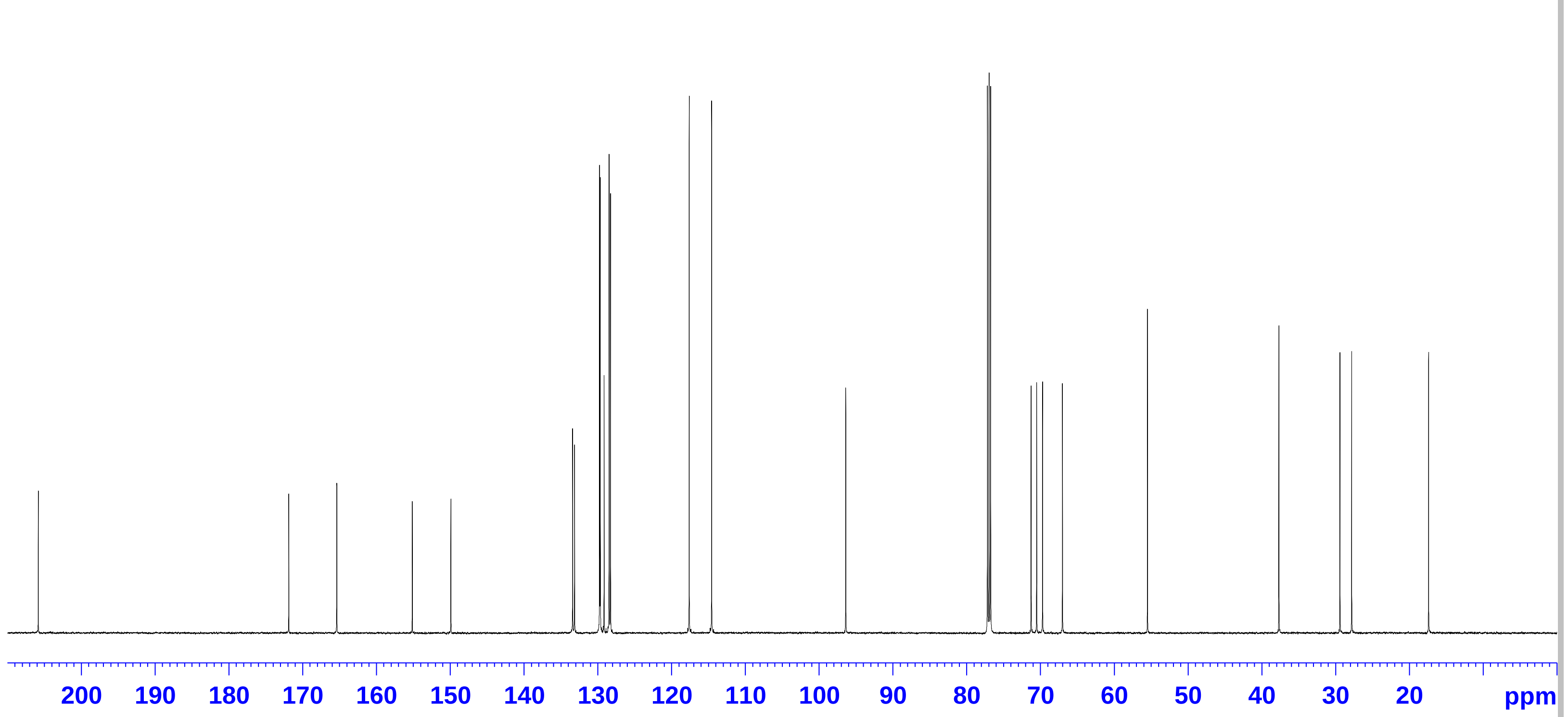

600 MHz ^1H NMR Spectrum of Compound **18** (recorded in $CDCl_3$)

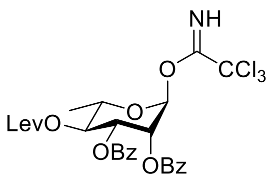

18

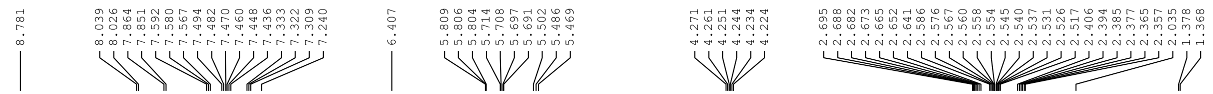

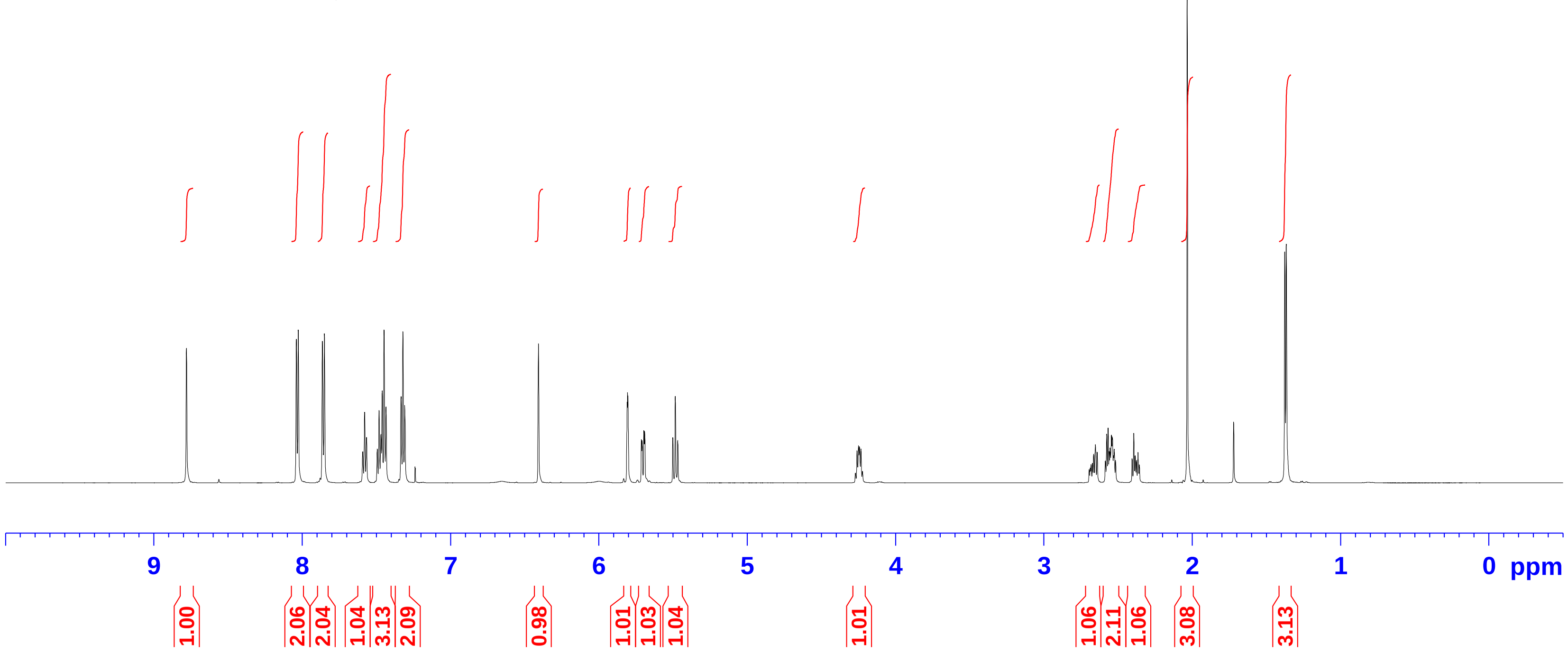

150 MHz 13C{1H} NMR Spectrum of Compound **18** (recorded in $CDCl_3$)

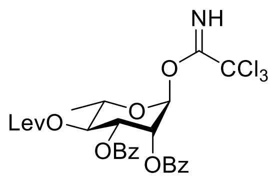

18

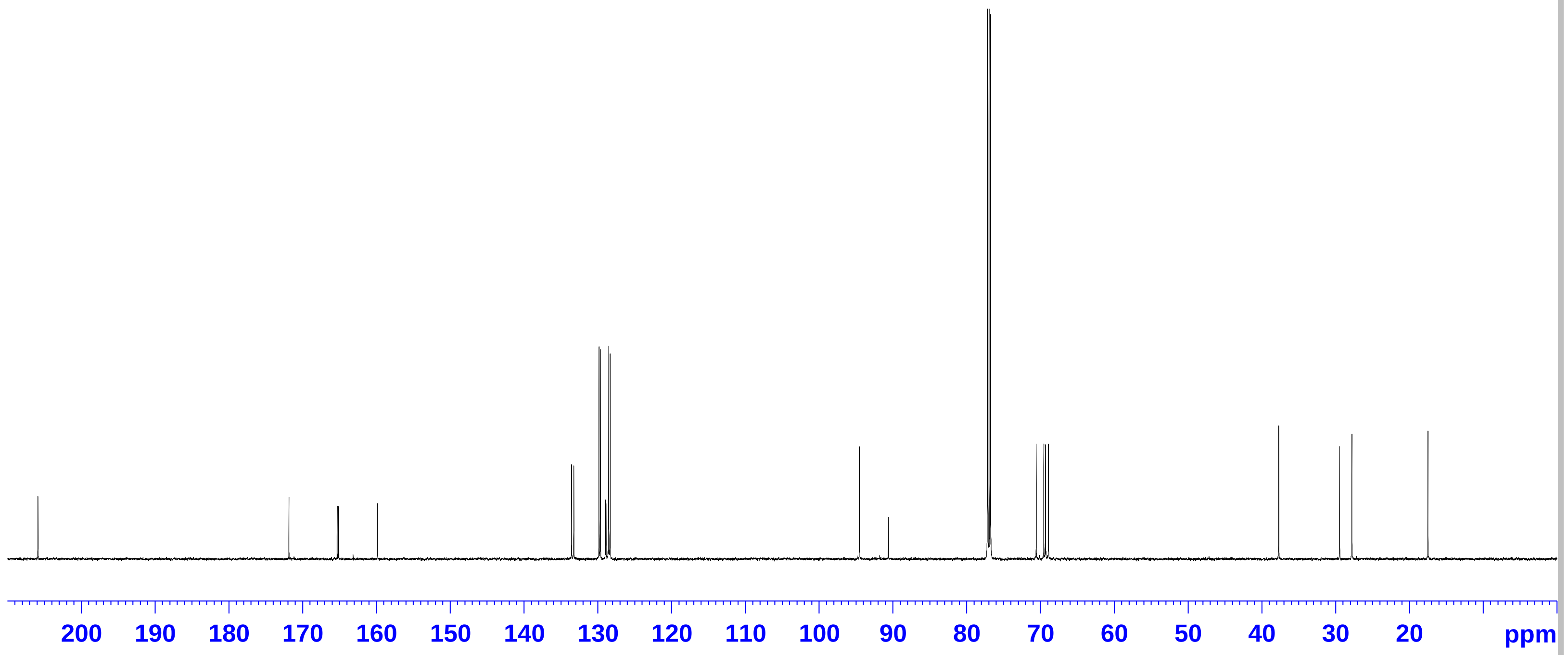

600 MHz ^{1}H NMR Spectrum of Compound **65** (recorded in $CDCl_3$)

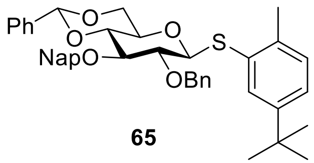

65

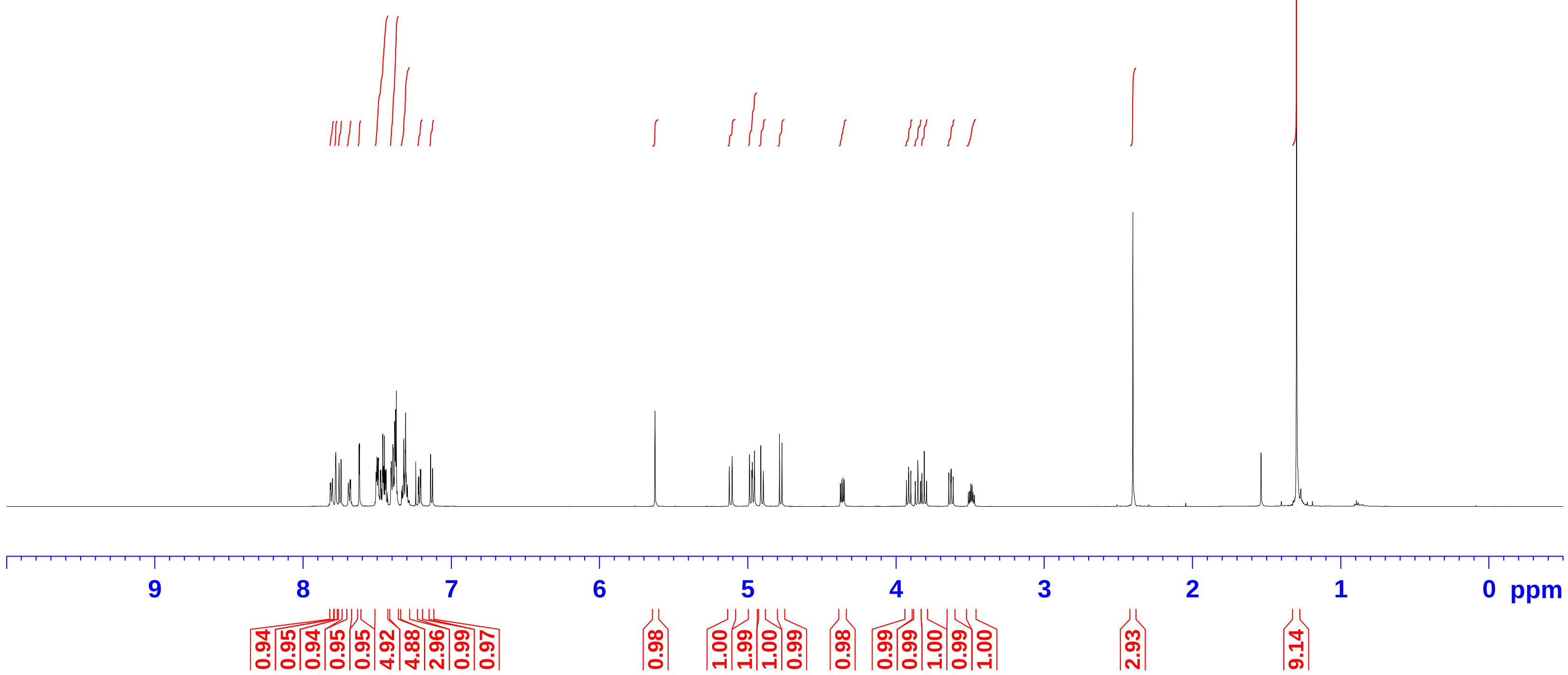

150 MHz 13C{1H} NMR Spectrum of Compound **65** (recorded in $CDCl_3$)

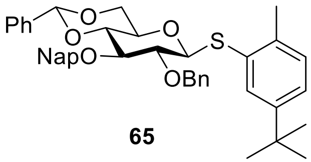

65

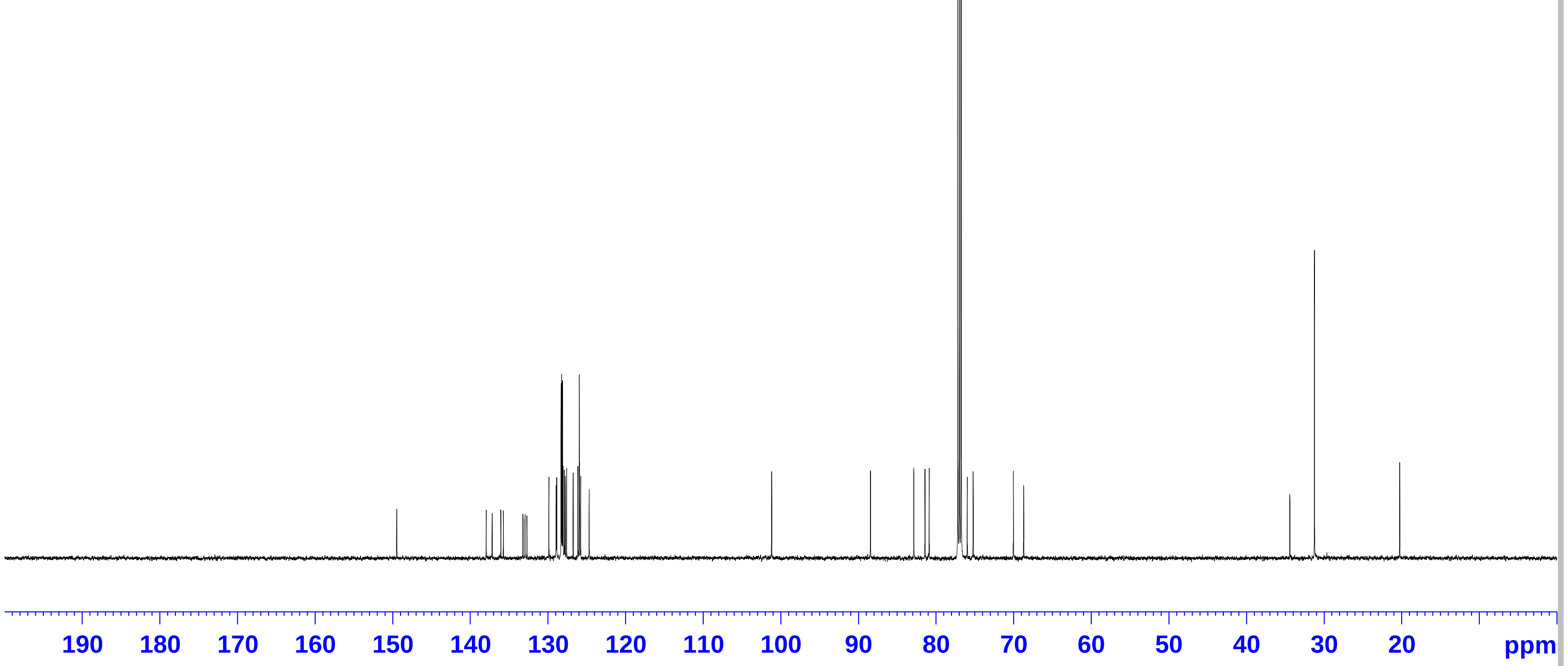

600 MHz ^{1}H NMR Spectrum of Compound **13** (recorded in $CDCl_3$)

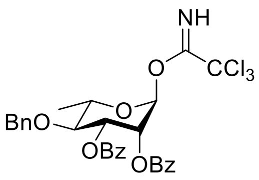

13

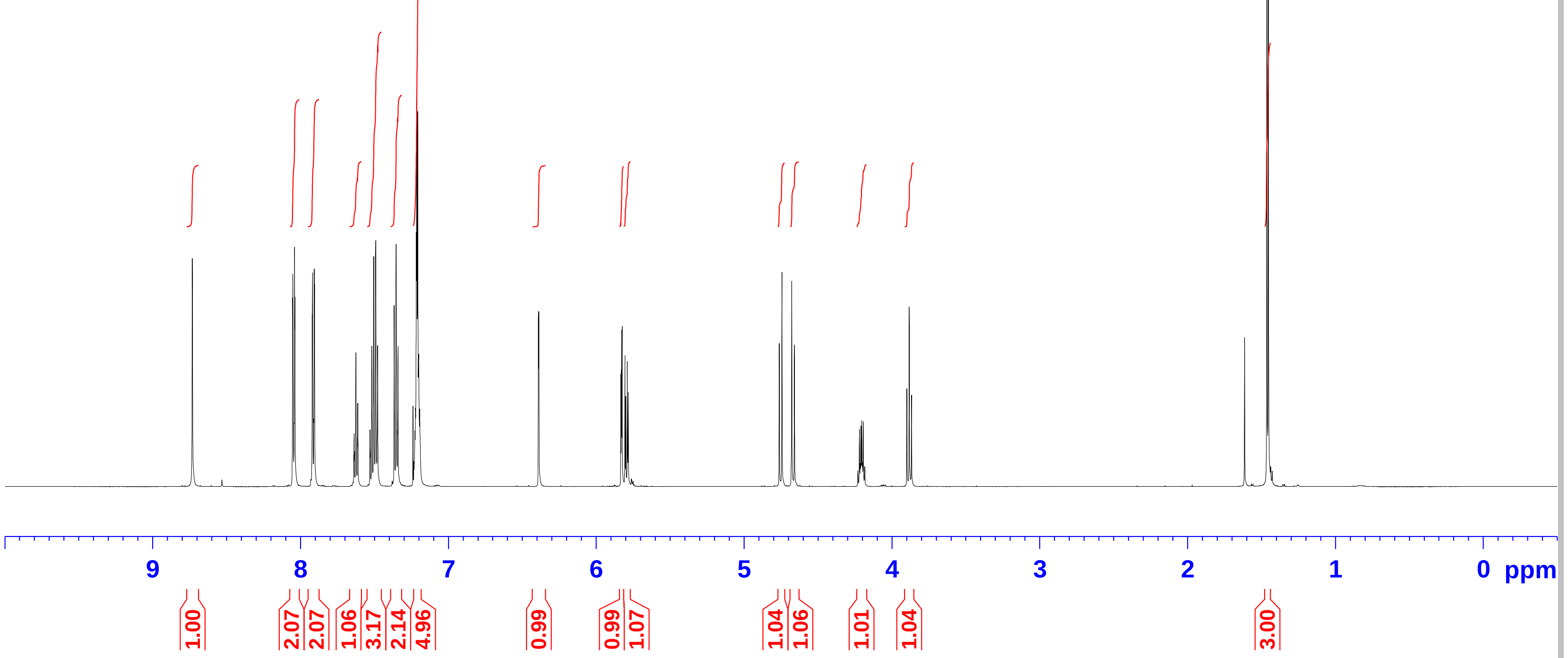

150 MHz 13C{1H} NMR Spectrum of Compound **13** (recorded in $CDCl_3$)

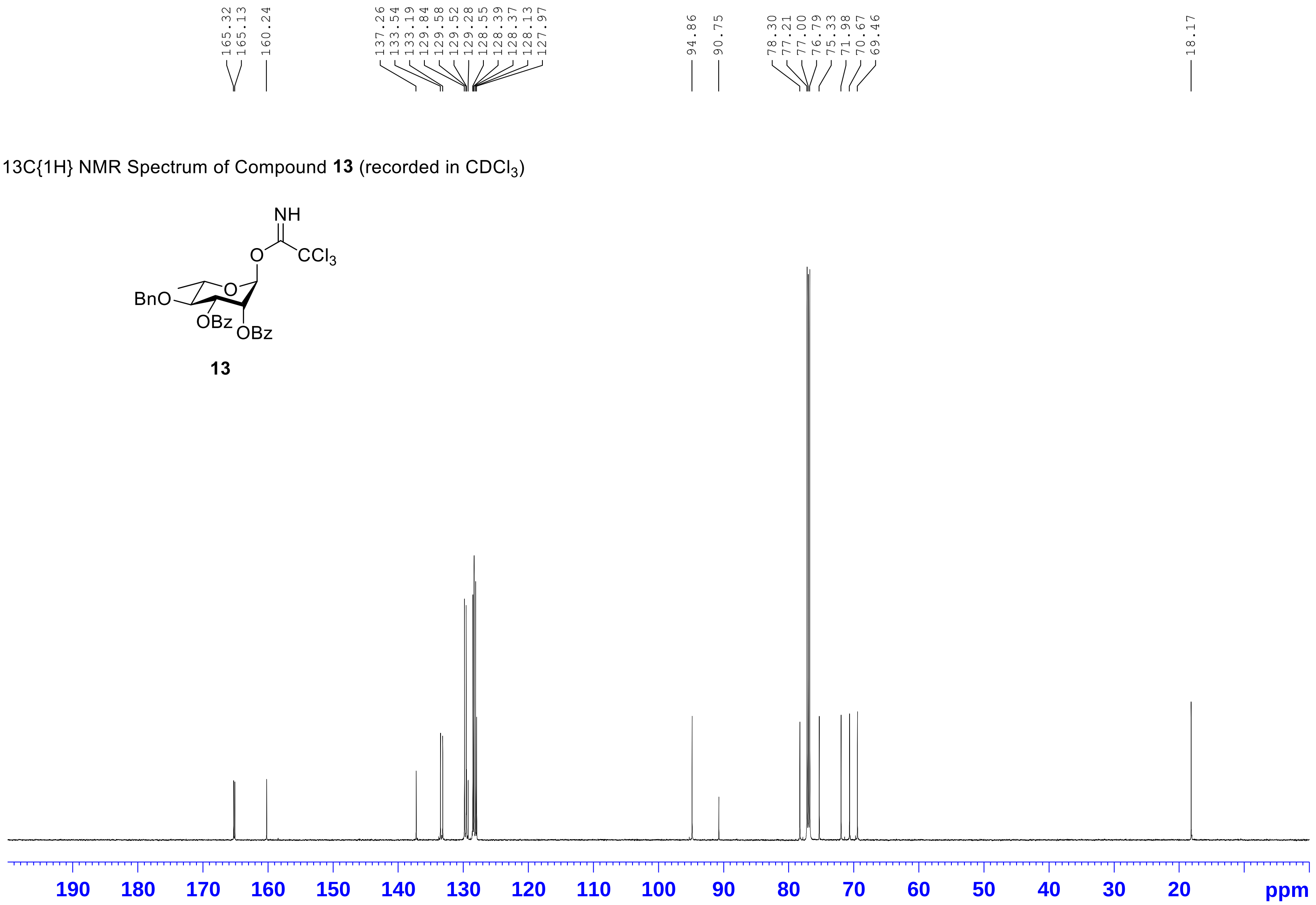

600 MHz ^{1}H NMR Spectrum of Compound **14** **α-isomer** (recorded in $CDCl_3$)

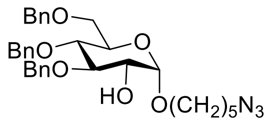

14 α-isomer

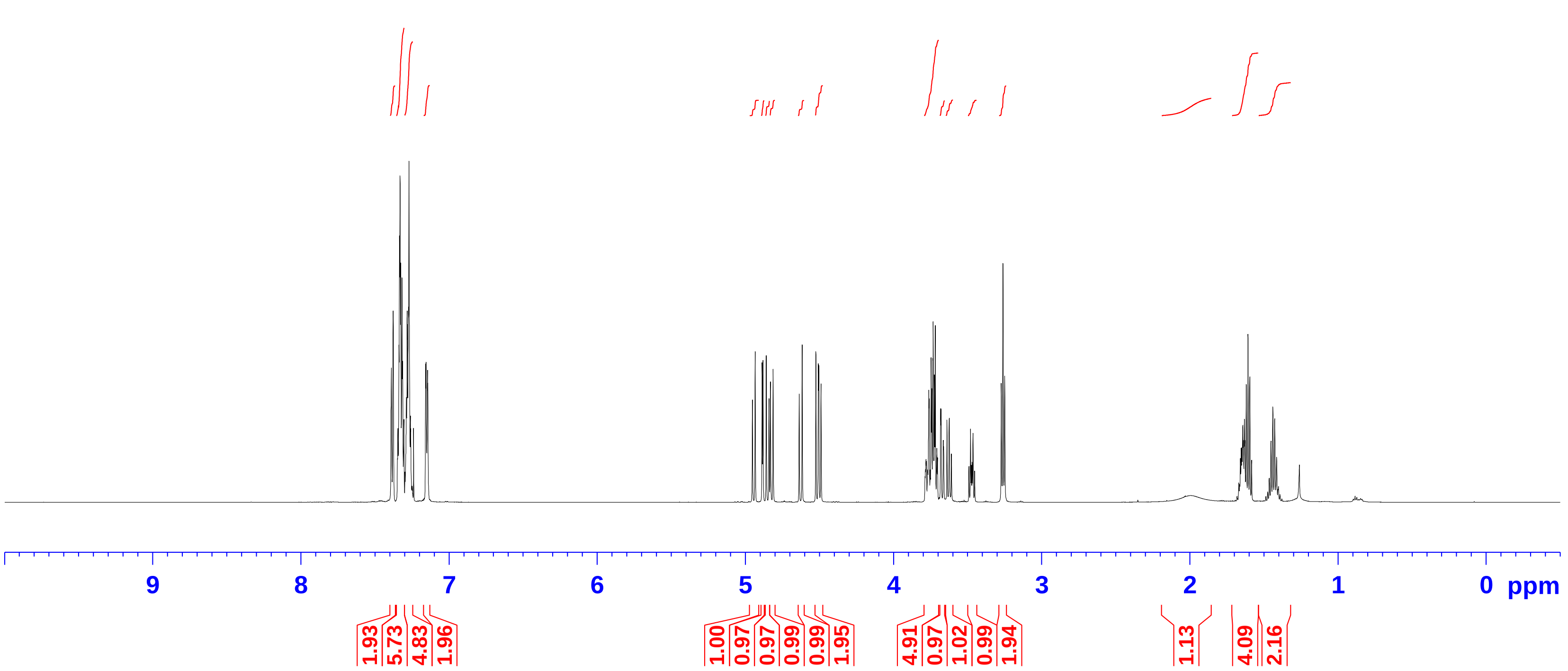

150 MHz 13C{1H} NMR Spectrum of Compound **14 α-isomer** (recorded in $CDCl_3$)

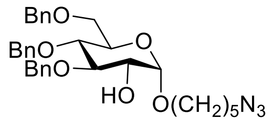

14 α-isomer

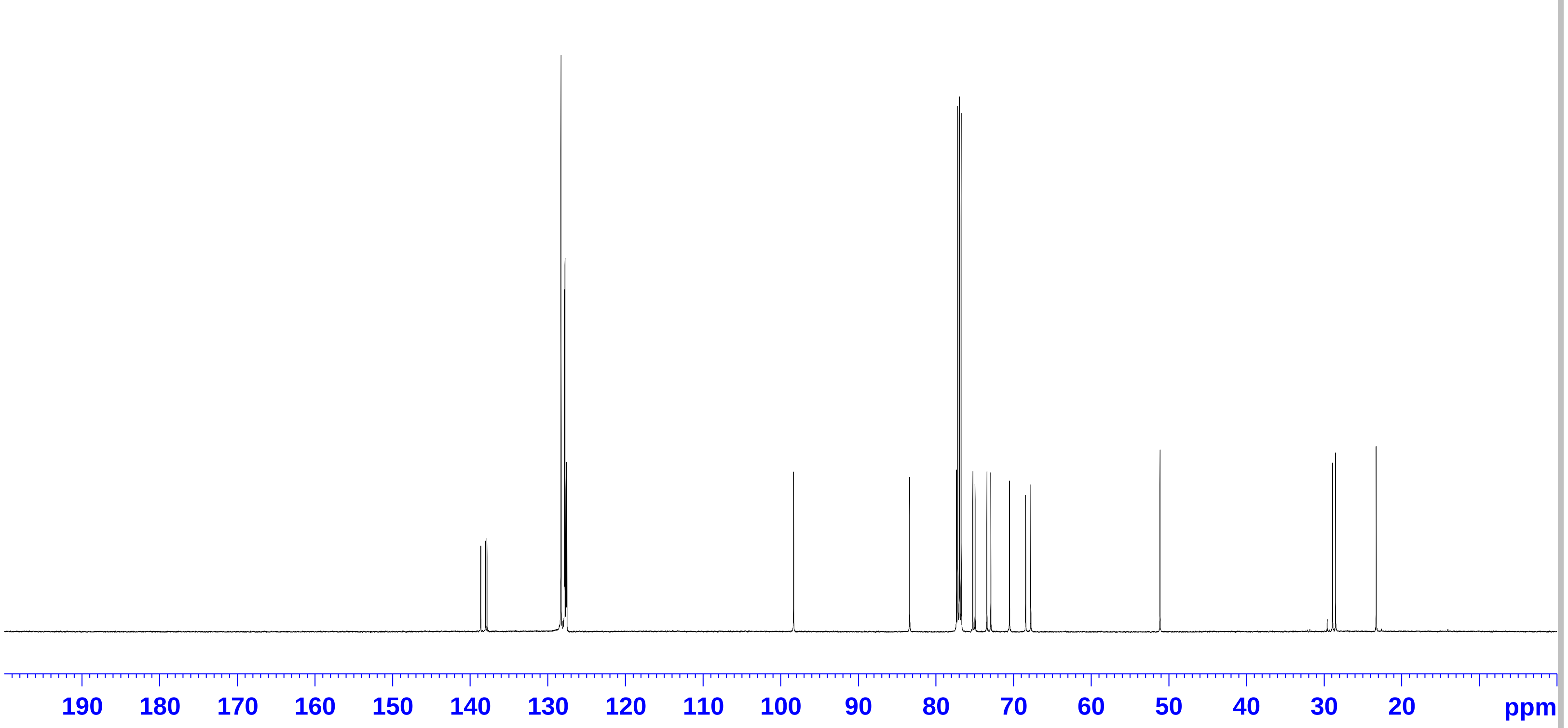

600 MHz ^{1}H NMR Spectrum of Compound **14 β-isomer** (recorded in $CDCl_3$)

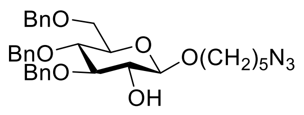

14 β-isomer

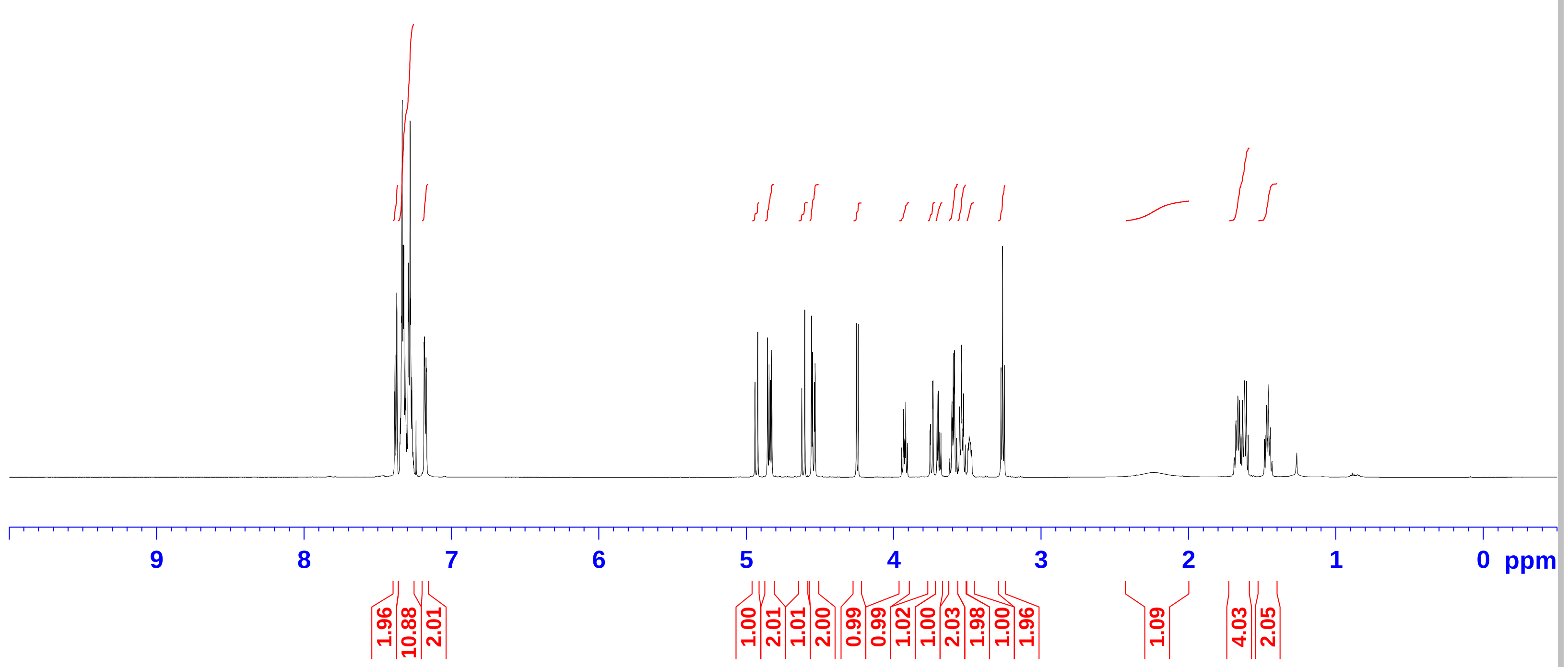

150 MHz 13C{1H} NMR Spectrum of Compound **14 β-isomer** (recorded in $CDCl_3$)

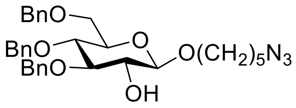

14 β-isomer

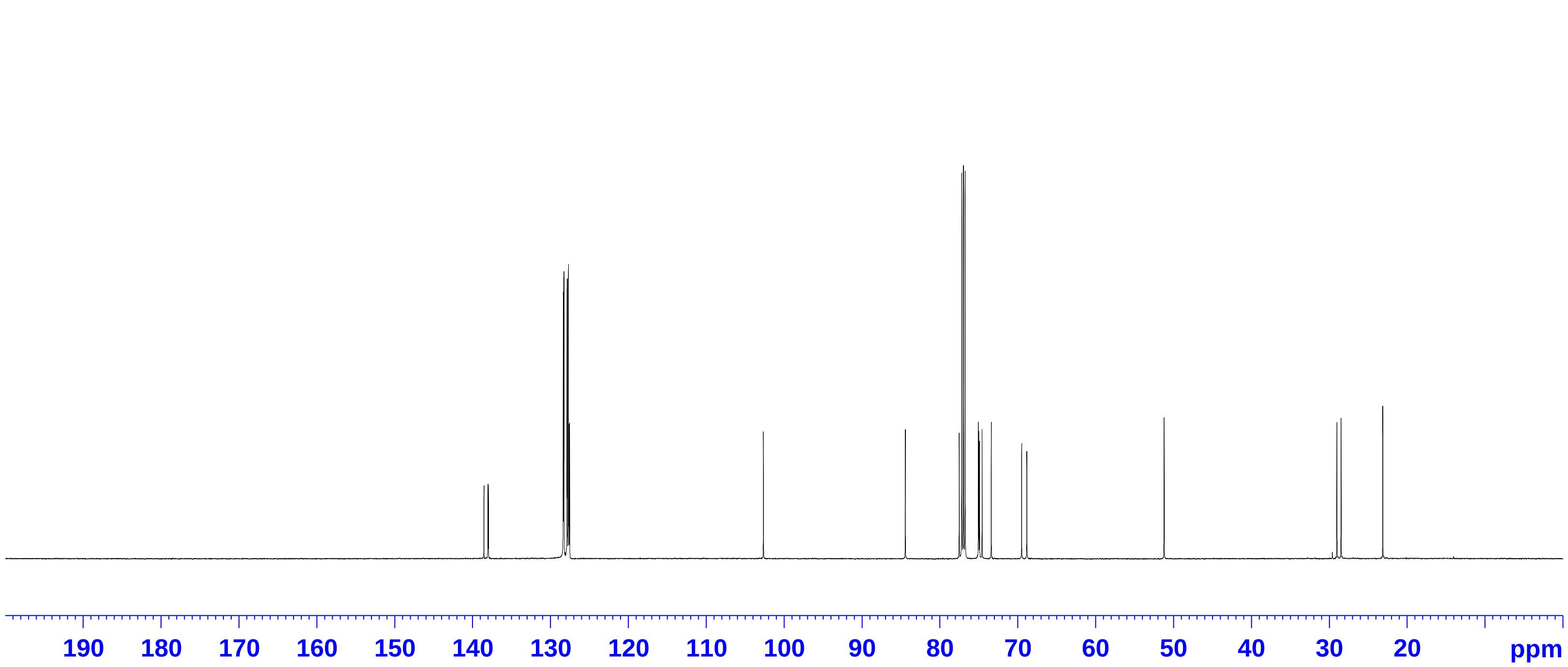

600 MHz ^{1}H NMR Spectrum of Compound **15** (recorded in $CDCl_3$)

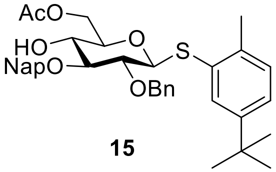

15

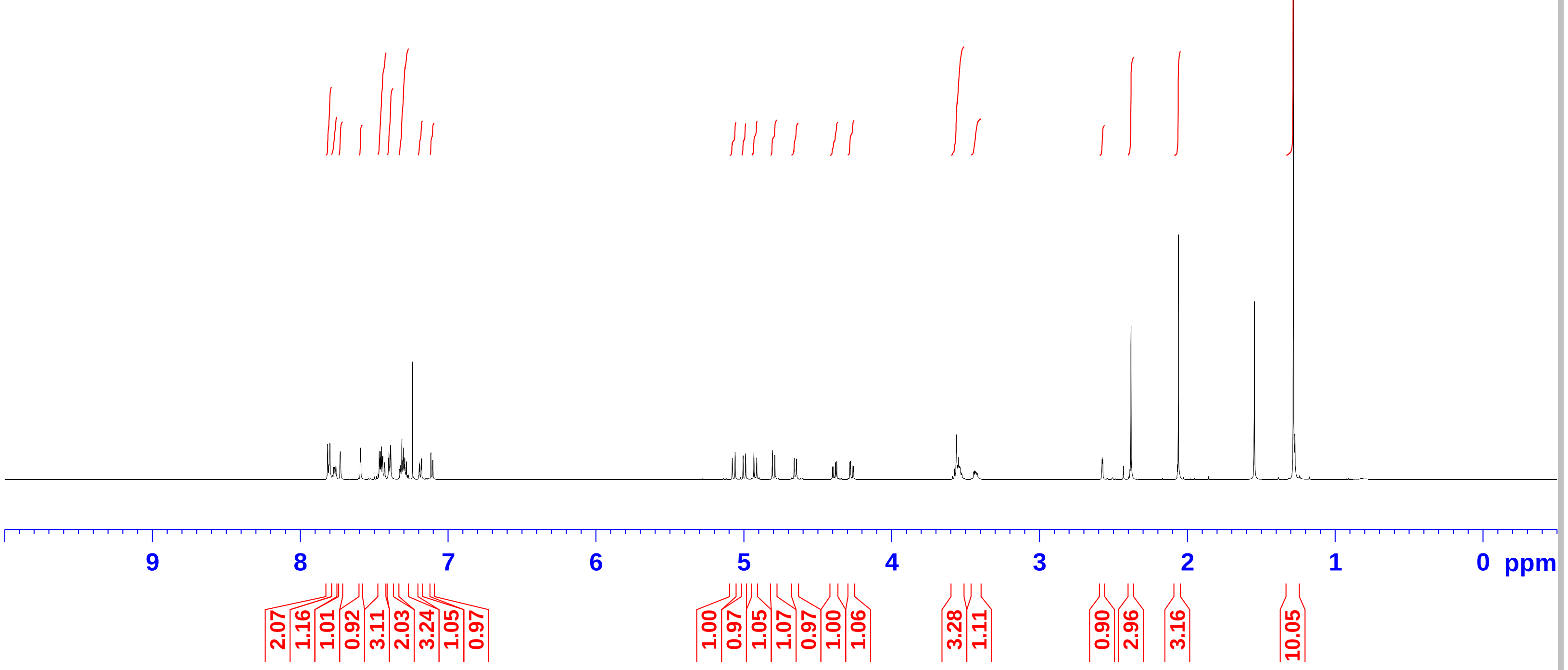

150 MHz 13C{1H} NMR Spectrum of Compound **15** (recorded in $CDCl_3$)

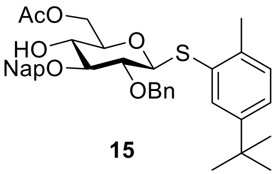

15

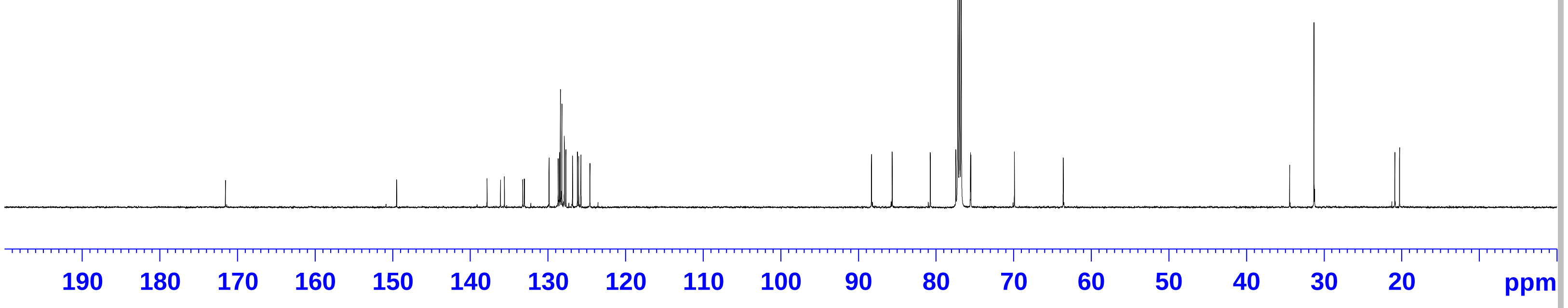

600 MHz ^{1}H NMR Spectrum of Compound **69** (recorded in $CDCl_3$)

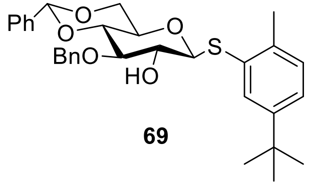

69

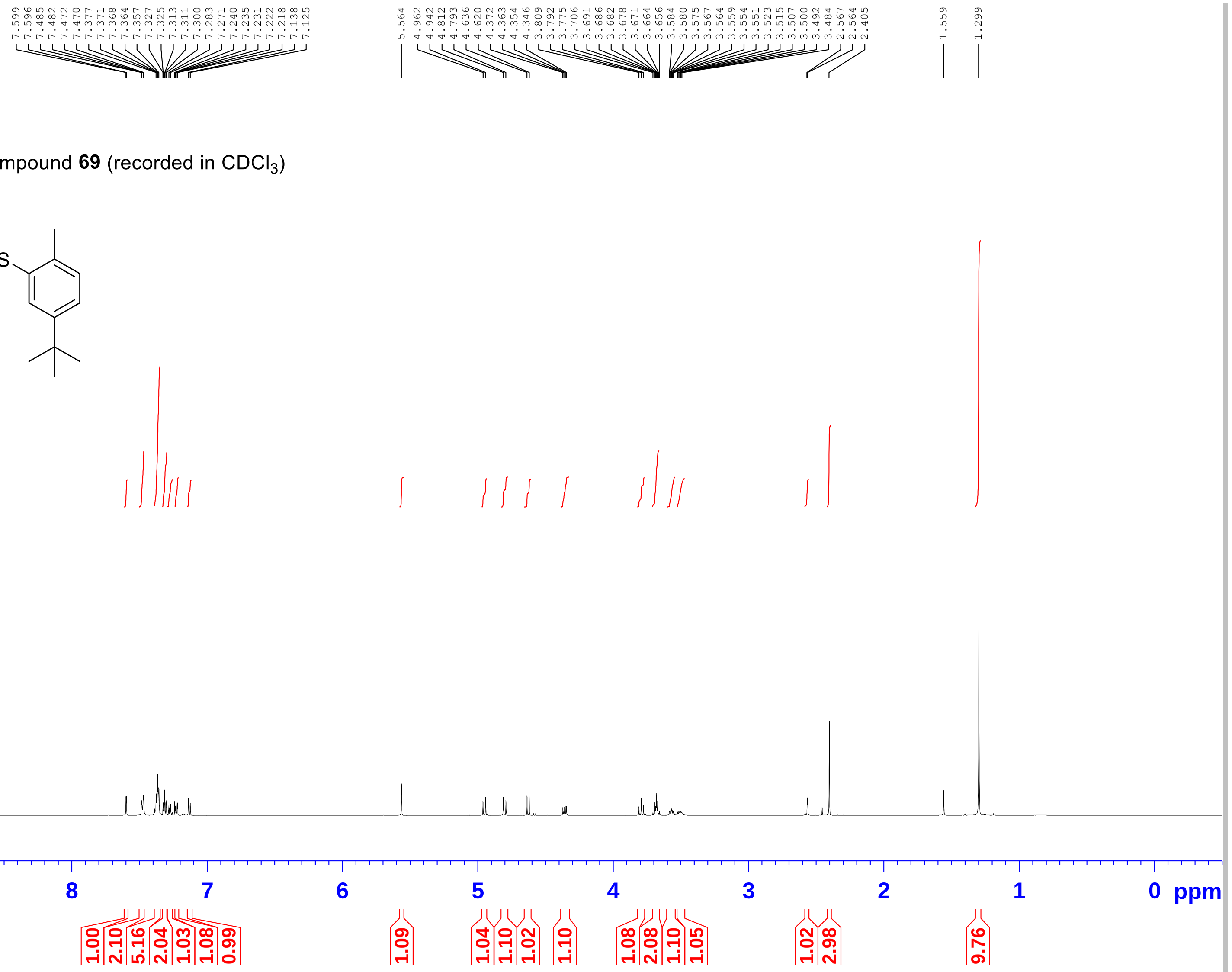

150 MHz 13C{1H} NMR Spectrum of Compound **69** (recorded in $CDCl_3$)

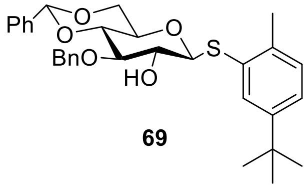

69

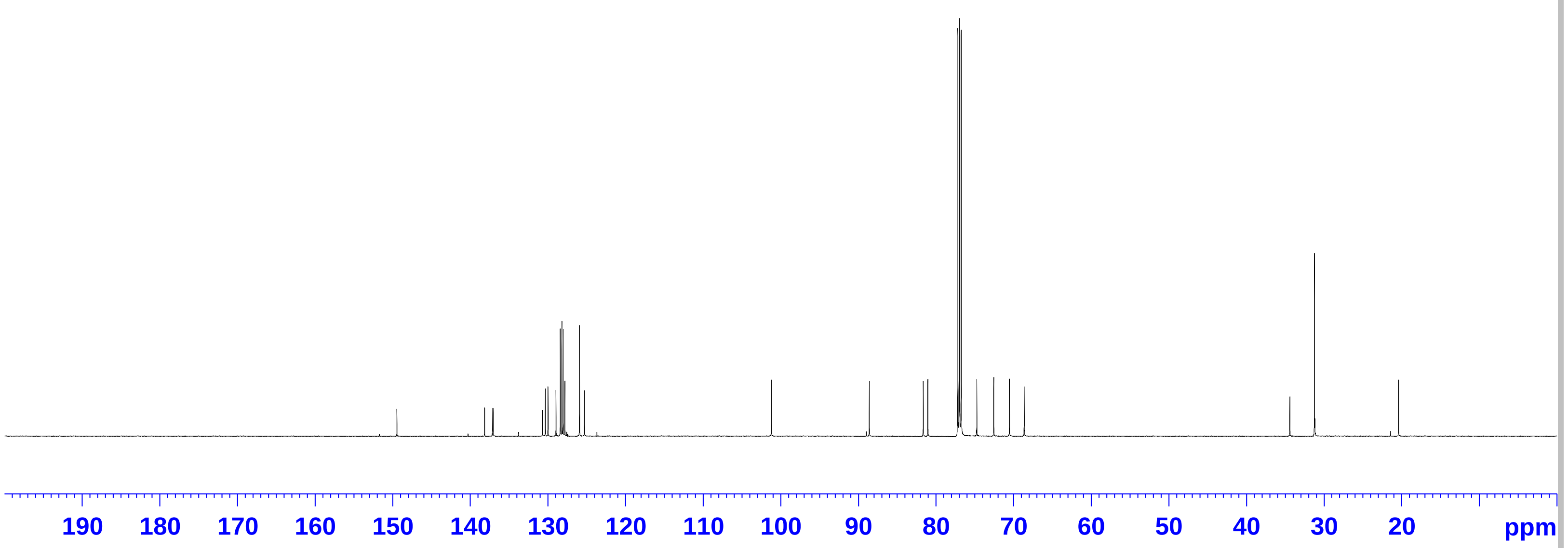

600 MHz ^{1}H NMR Spectrum of Compound **7** (recorded in $CDCl_3$)

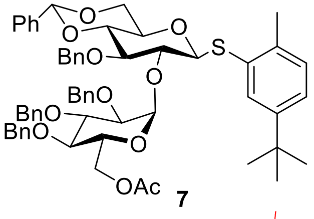

7

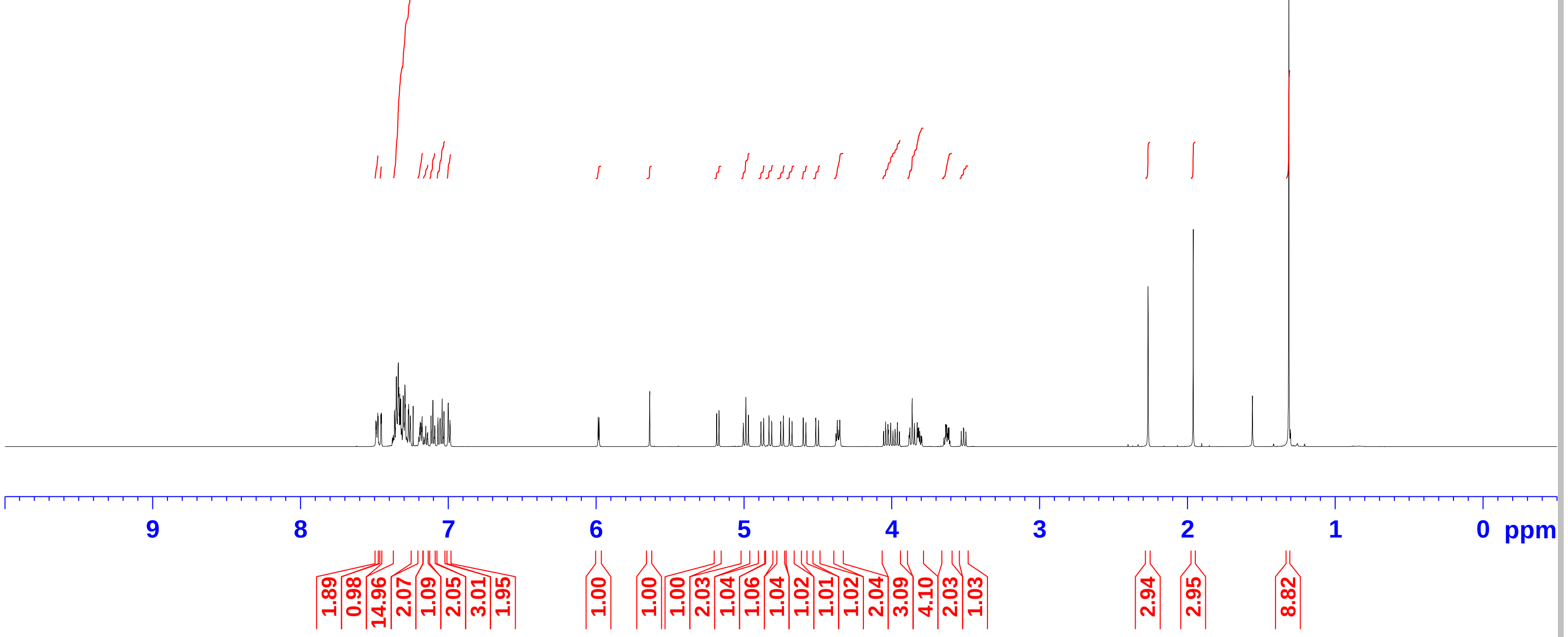

150 MHz 13C{1H} NMR Spectrum of Compound **7** (recorded in $CDCl_3$)

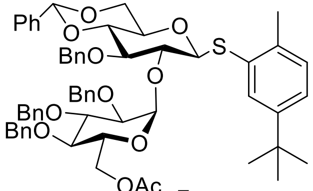

7

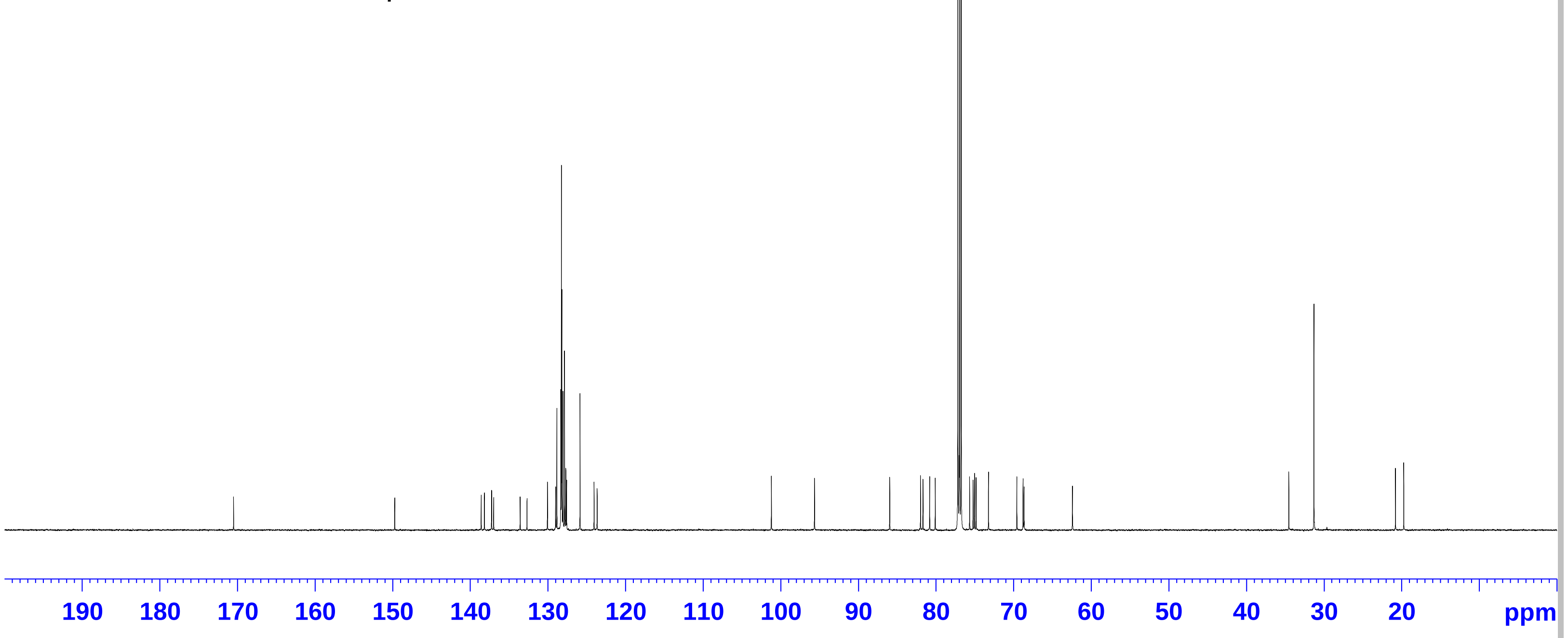

600 MHz ^{1}H NMR Spectrum of Compound 3**7** (recorded in $CDCl_3$)

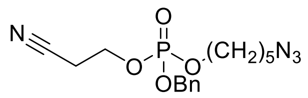

37

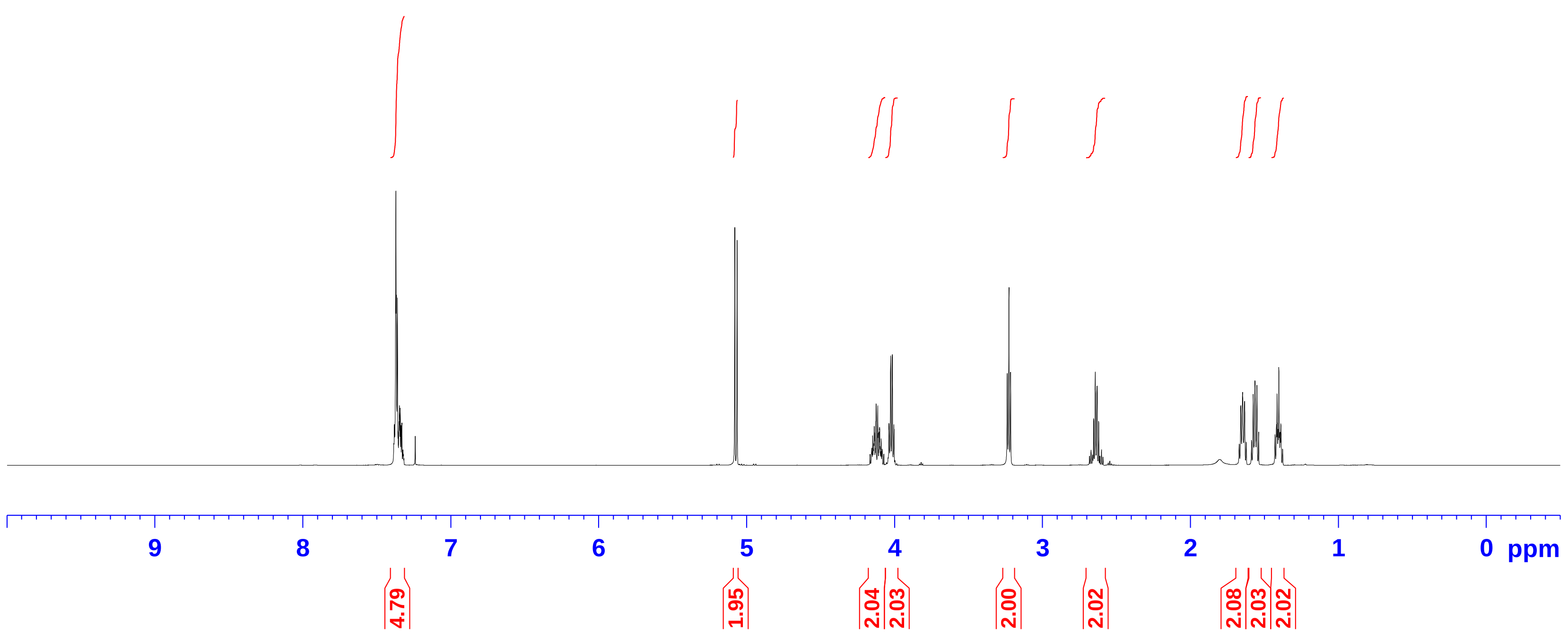

150 MHz 13C{1H} NMR Spectrum of Compound **37** (recorded in $CDCl_3$)

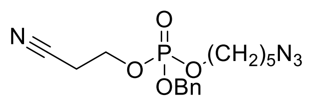

37

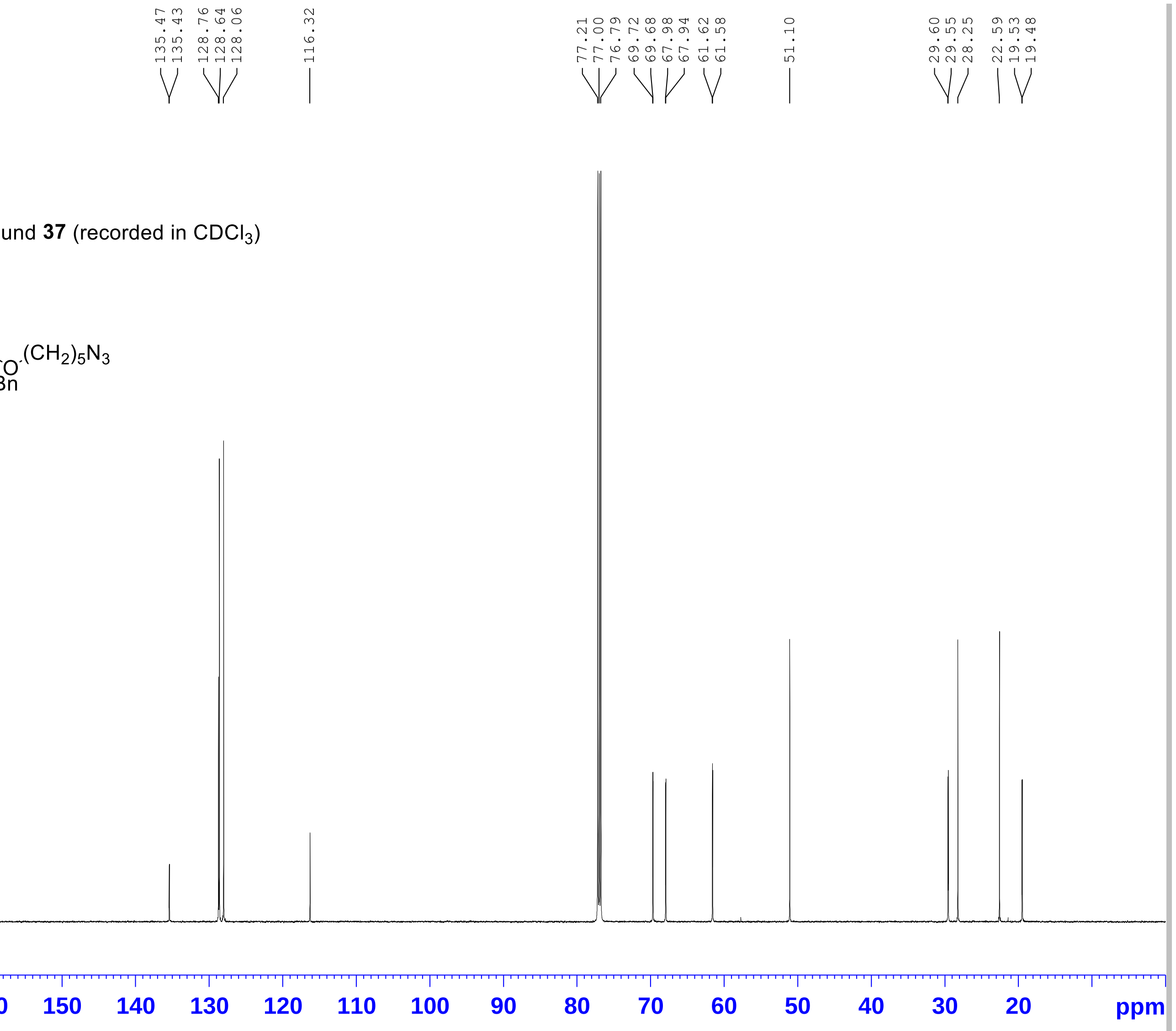

600 MHz ^{1}H NMR Spectrum of Compound **70a** (recorded in $CDCl_3$)

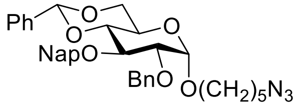

70a

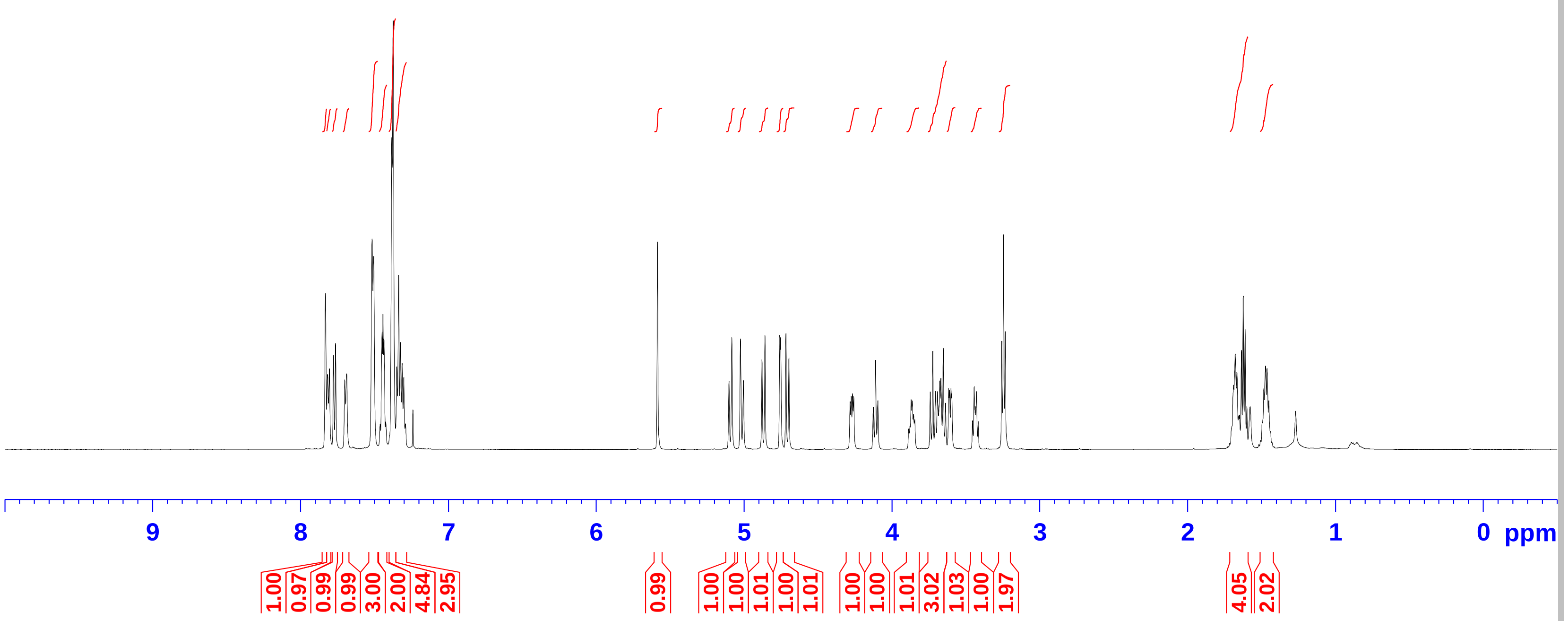

150 MHz 13C{1H} NMR Spectrum of Compound **70a** (recorded in $CDCl_3$)

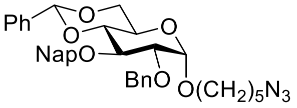

70a

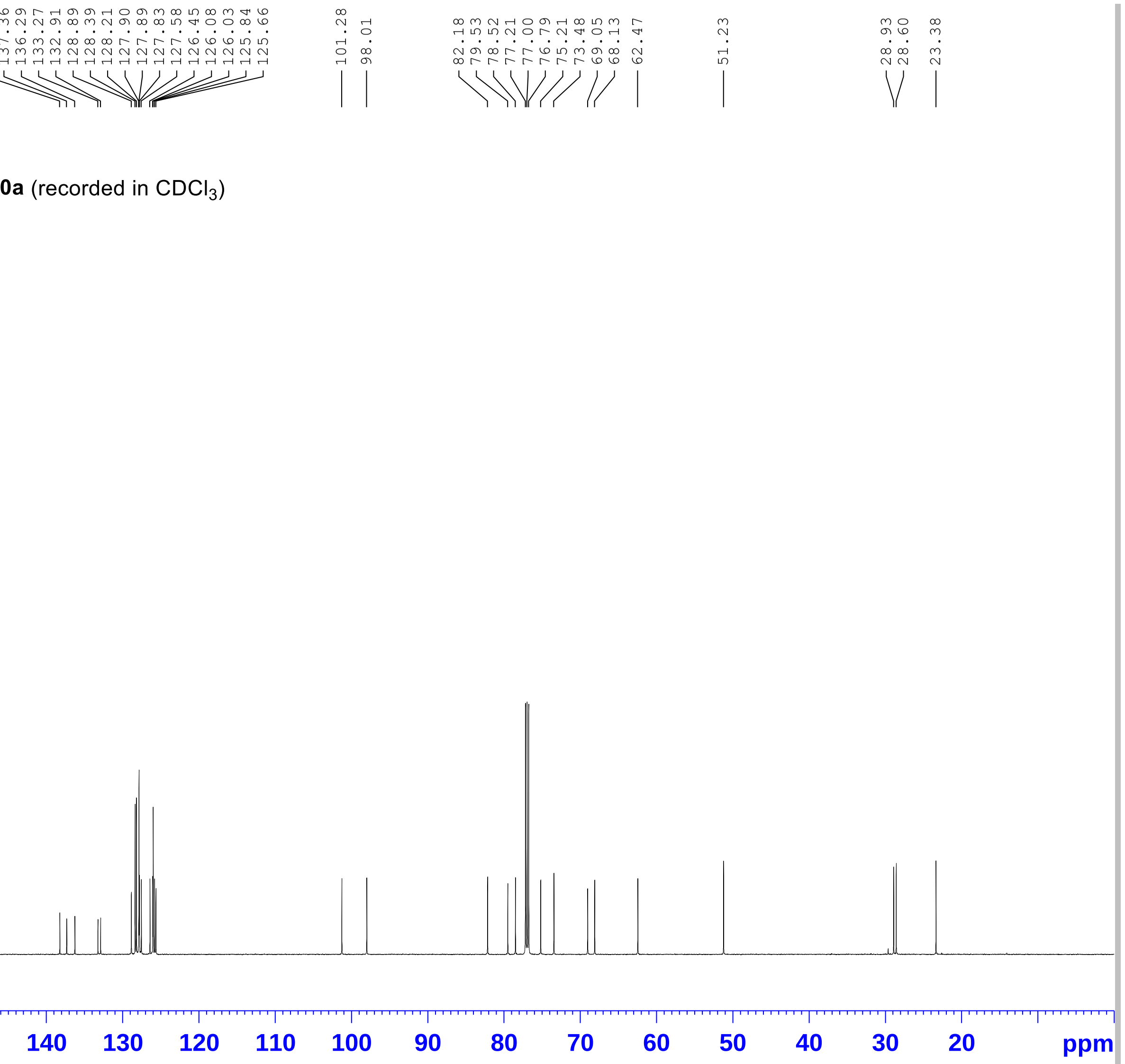

600 MHz ^{1}H NMR Spectrum of Compound **70b** (recorded in $CDCl_3$)

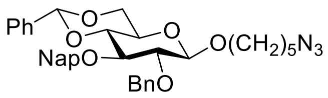

70b

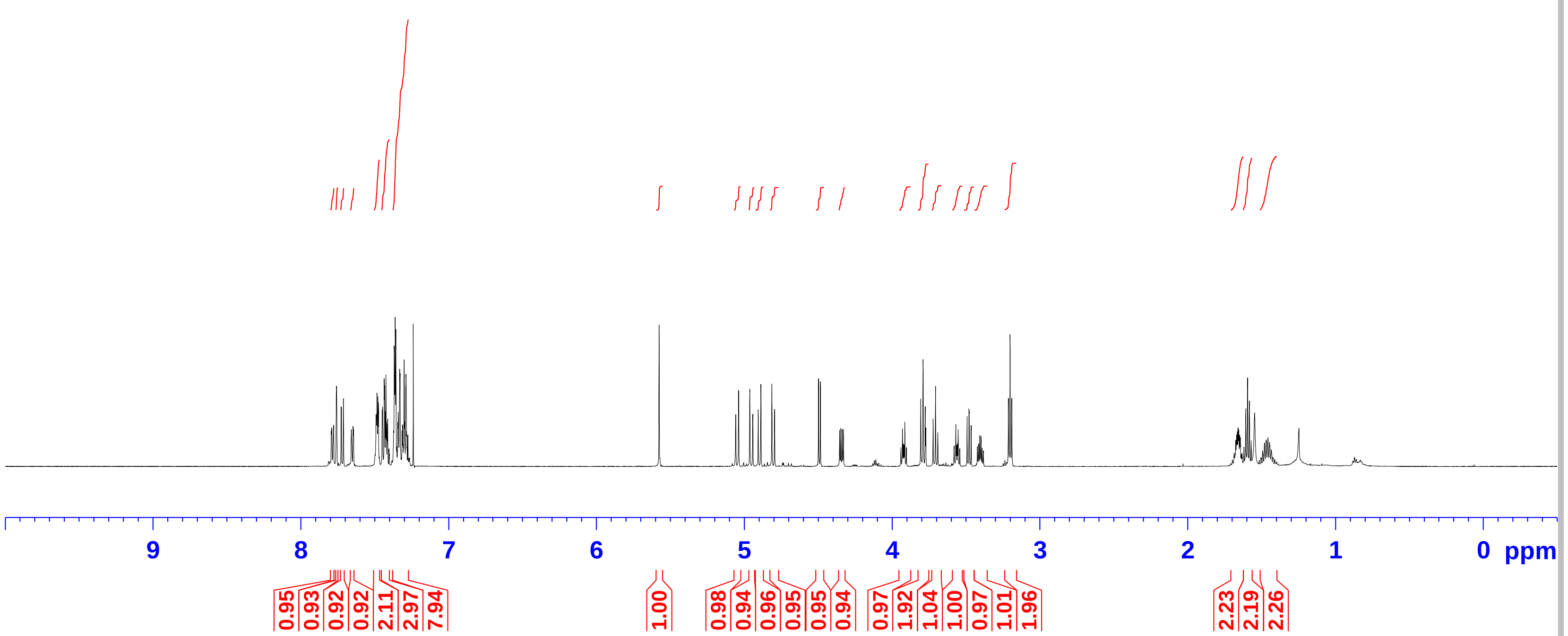

150 MHz 13C{1H} NMR Spectrum of Compound **70b** (recorded in $CDCl_3$)

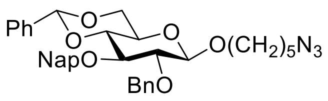

70b

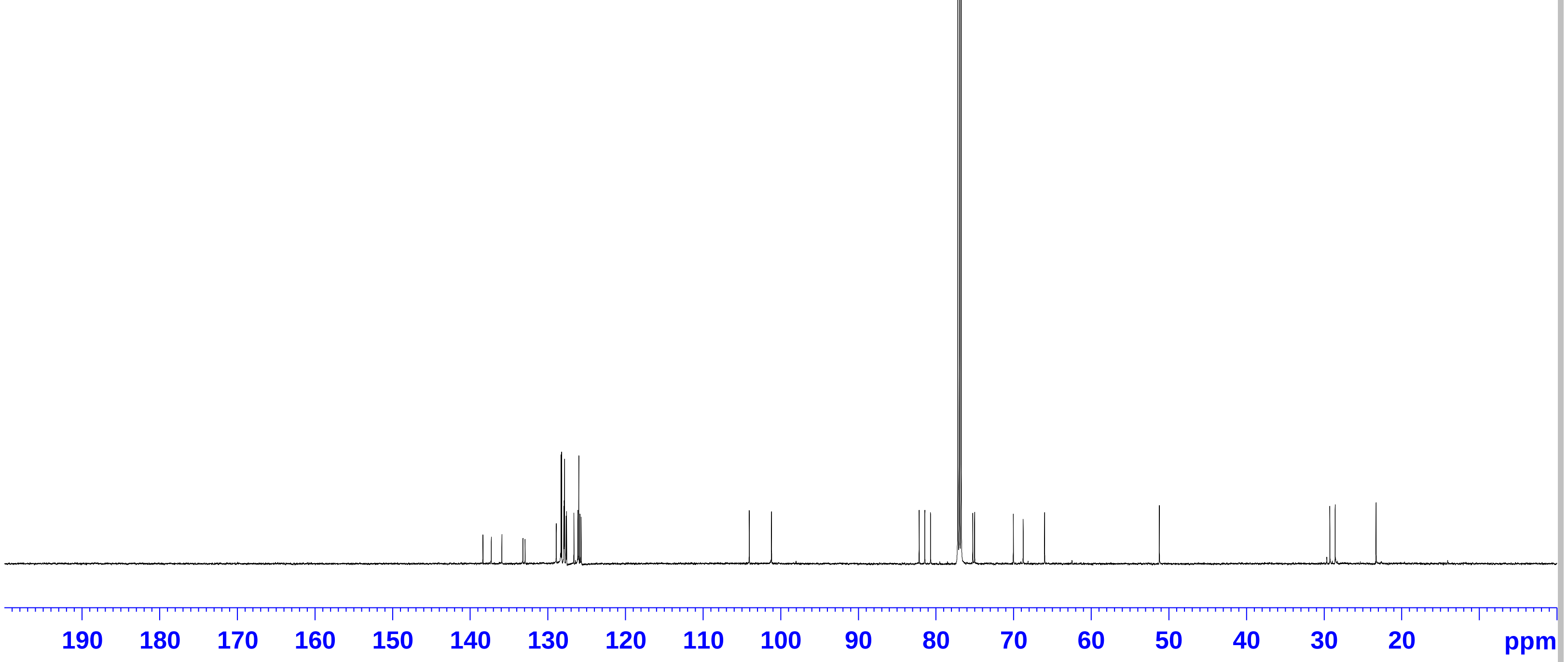

600 MHz ^{1}H NMR Spectrum of Compound **42** (recorded in $CDCl_3$)

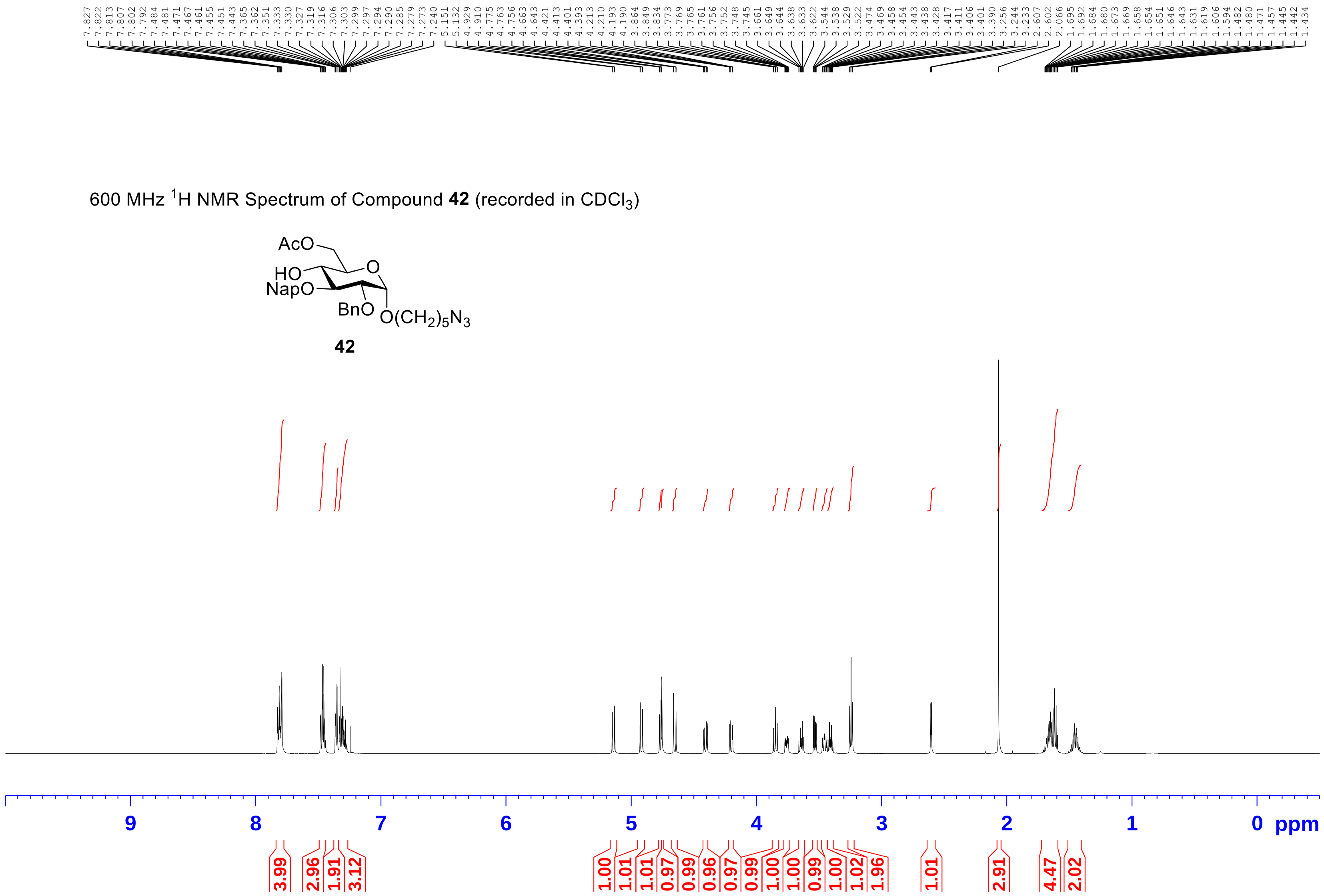

150 MHz 13C{1H} NMR Spectrum of Compound **42** (recorded in $CDCl_3$)

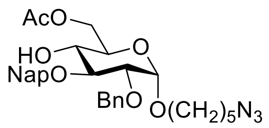

42

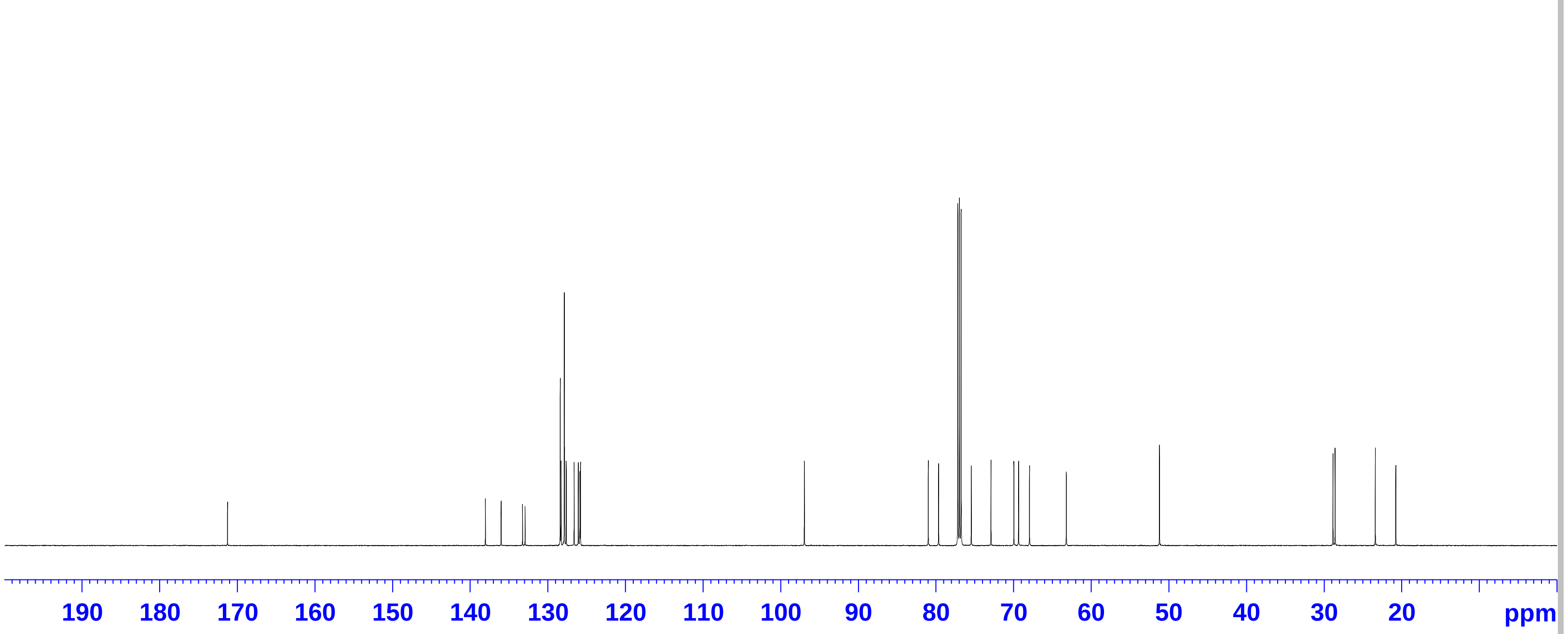

(2-Methyl-5-*tert*-butylphenyl) 2,3-*O*-dibenzoyl-4-*O*-oxopentanoate-α-L-rhamnopyranosyl-(1→3)-2-*O*-benzoyl-4,6-*O*-benzylidene-1-thio-β-D-glucopyranoside (19).

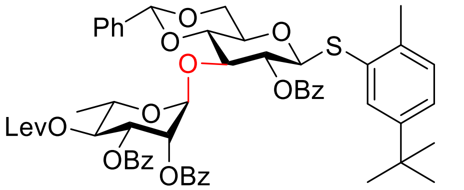

19

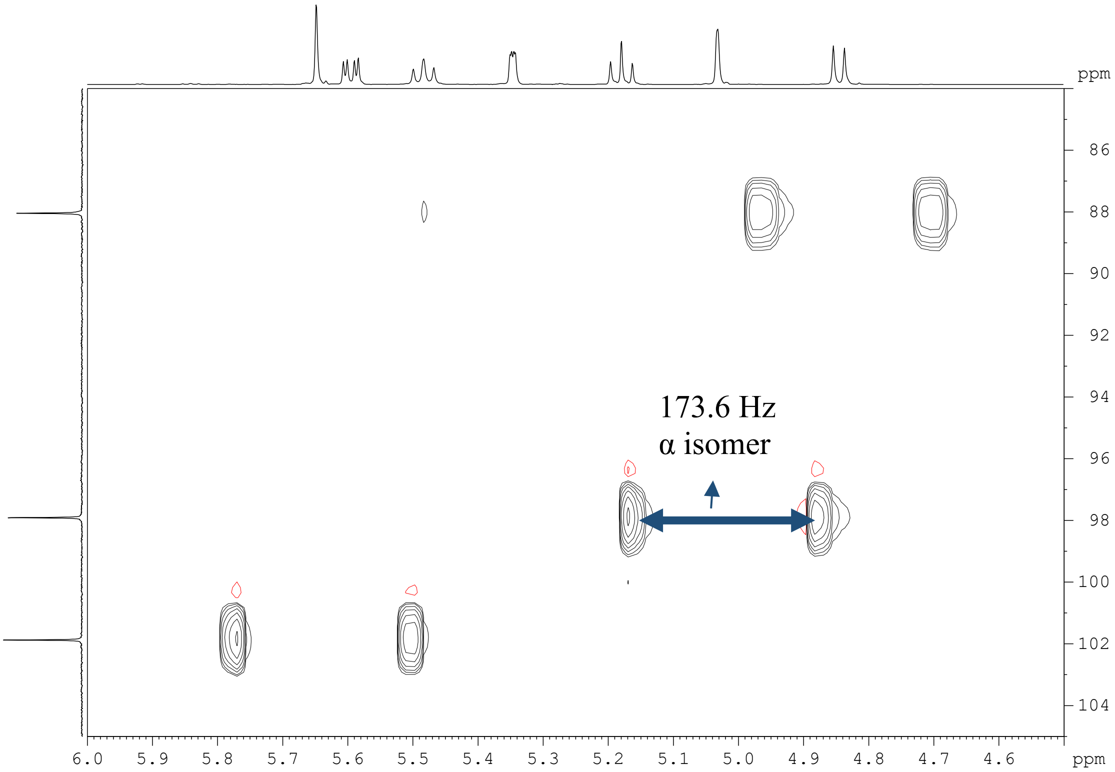

(2-Methyl-5-tert-butylphenyl) 2,3-di-*O*-benzoyl-4-*O*-benzyl-α-L-rhamnopyranosyl-(1→3)-4,6-*O*-benzylidene-2-*O*-benzyl-1-thio-β-D-glucopyranoside (21).

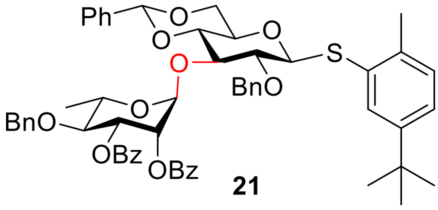

21

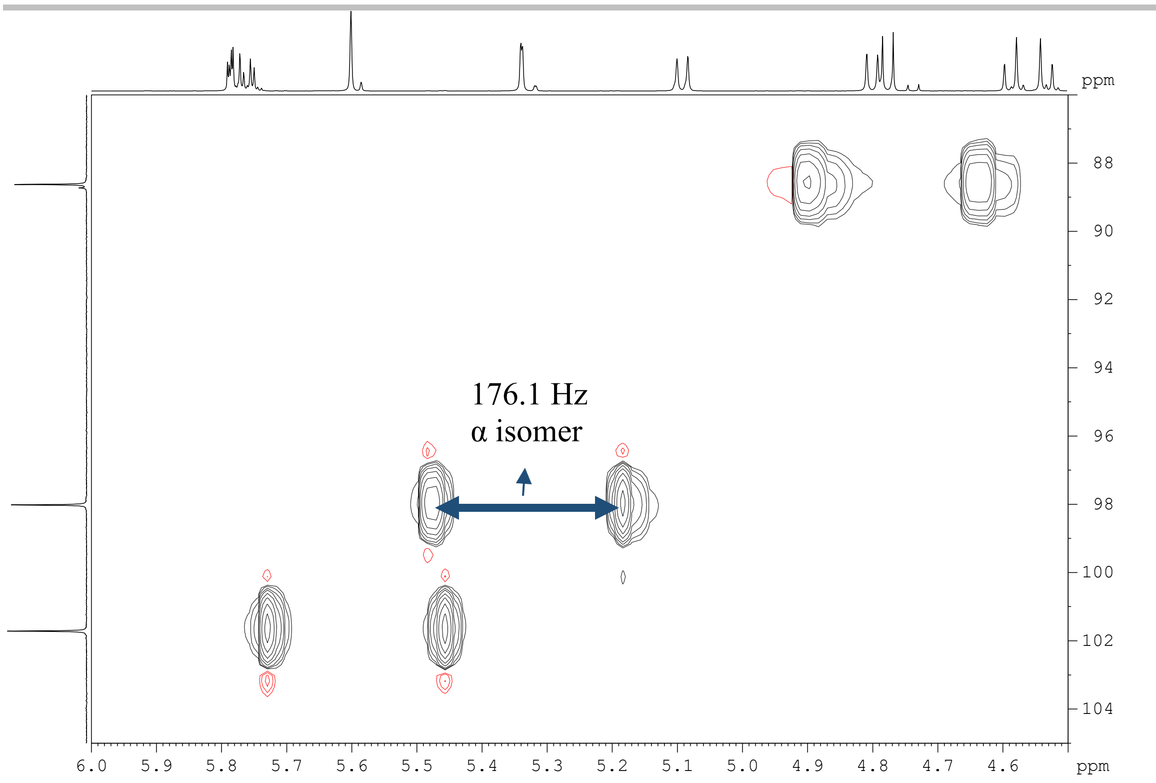

(2-Methyl-5-tert-butylphenyl) 2,3-*O*-dibenzoyl-4-*O*-oxopentanoate-α-L-rhamnopyranosyl-(1→3)-2-*O*-benzoyl-4,6-*O*-benzylidene-β-D-glucopyranosyl-(1→4)-[2,3-di-*O*-benzoyl-4-*O*-benzyl-α-L-rhamnopyranosyl-(1→3)]-6-*O*-acetyl-2-*O*-benzyl-1-thio-β-D-glucopyranoside (23a).

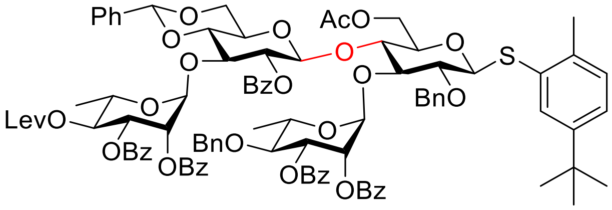

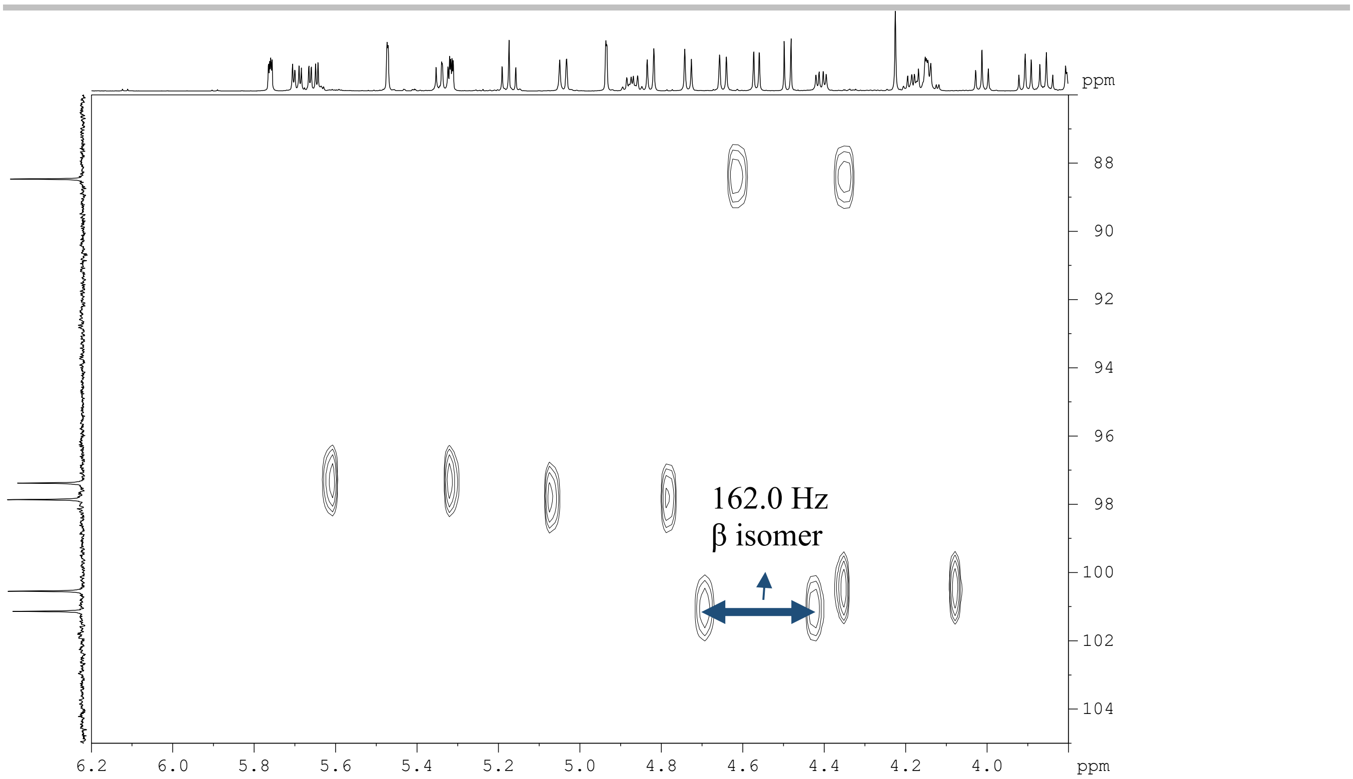

5-Azidopentyl 2,3-*O*-dibenzoyl-4-*O*-oxopentanoate-α-L-rhamnopyranosyl-(1→3)-2-*O*-benzoyl-4,6-*O*-benzylidene-β-D-glucopyranosyl-(1→4)-[2,3-di-*O*-benzoyl-4-*O*-benzyl-α-L-rhamnopyranosyl-(1→3)]-6-*O*-acetyl-2-*O*-benzyl-α-D-glucopyranosyl-(1→2)-3,4,6-*tri*-*O*-benzyl-α-D-glucopyranoside (9).

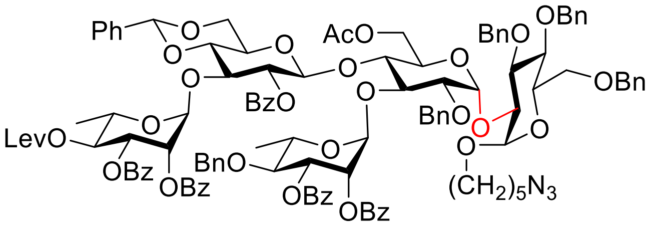

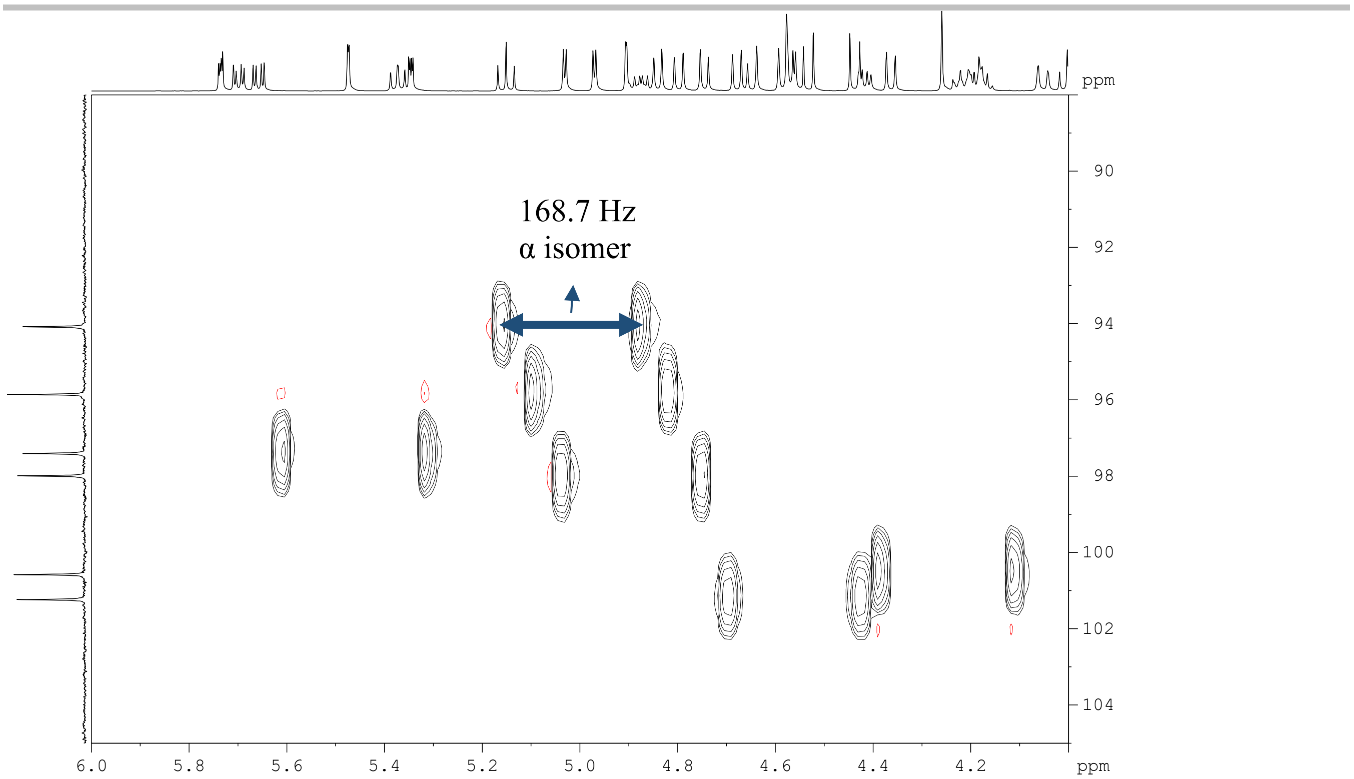

5-Aminopentyl α-D-glucopyranosyl-(1→dihydrogen phosporyl→4)-α-L-rhamnopyranosyl-(1→3)-β-D-glucopyranosyl-(1→4)-[α-L-rhamnopyranosyl-(1→3)]-α-D-glucopyranosyl-(1→2)-α-D-glucopyranoside (3). $^{1}J_{CH}$ for α, β isomer.

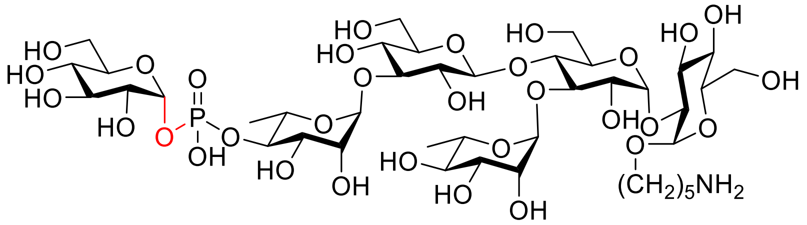

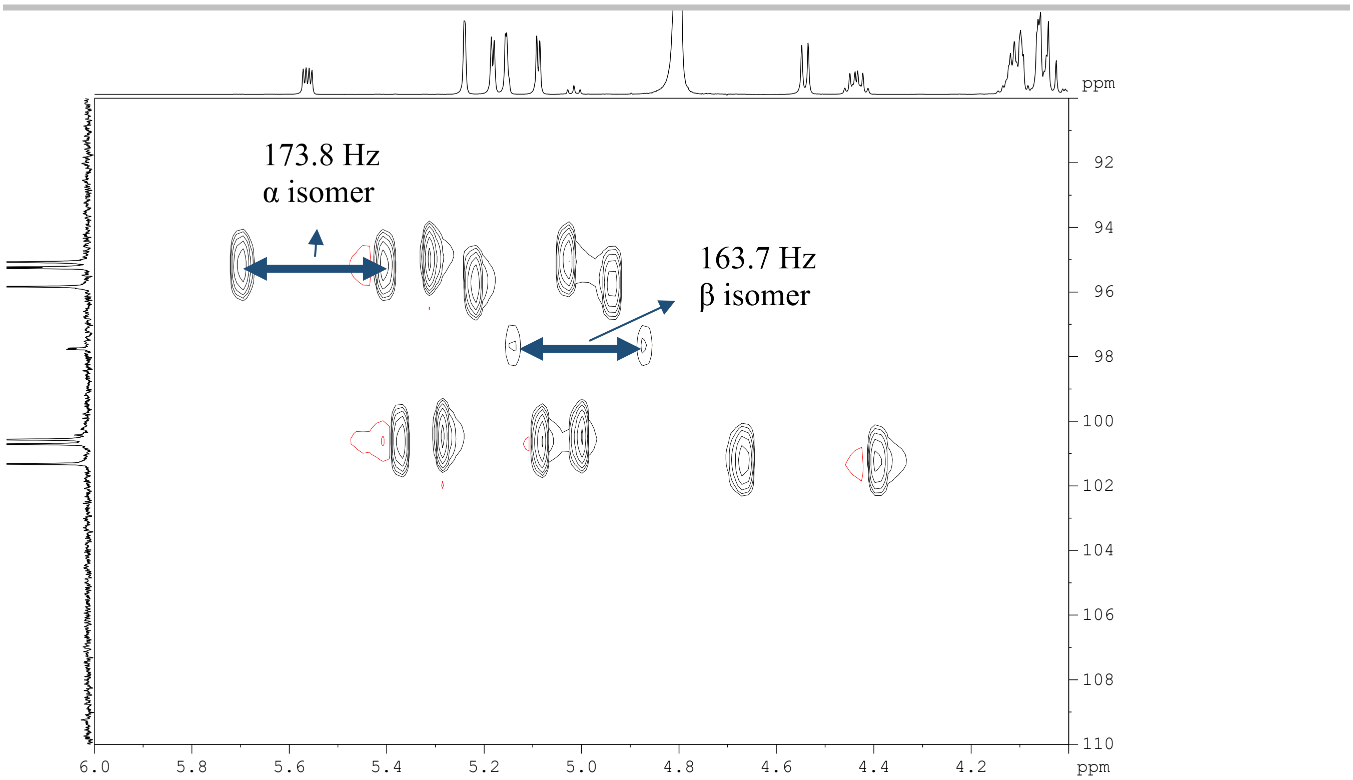

(2-Methyl-5-tert-butylphenyl) 6-*O*-acetyl-2,3,4-tri-*O*-benzyl-α-D-glucopyranosyl-(1→2)-4,6-*O*-benzylidene-3-*O*-benzyl-1-thio-β-D-glucopyranoside (7).

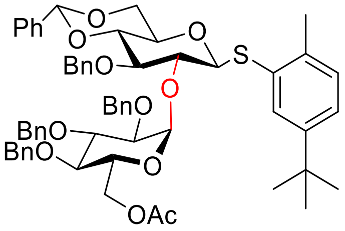

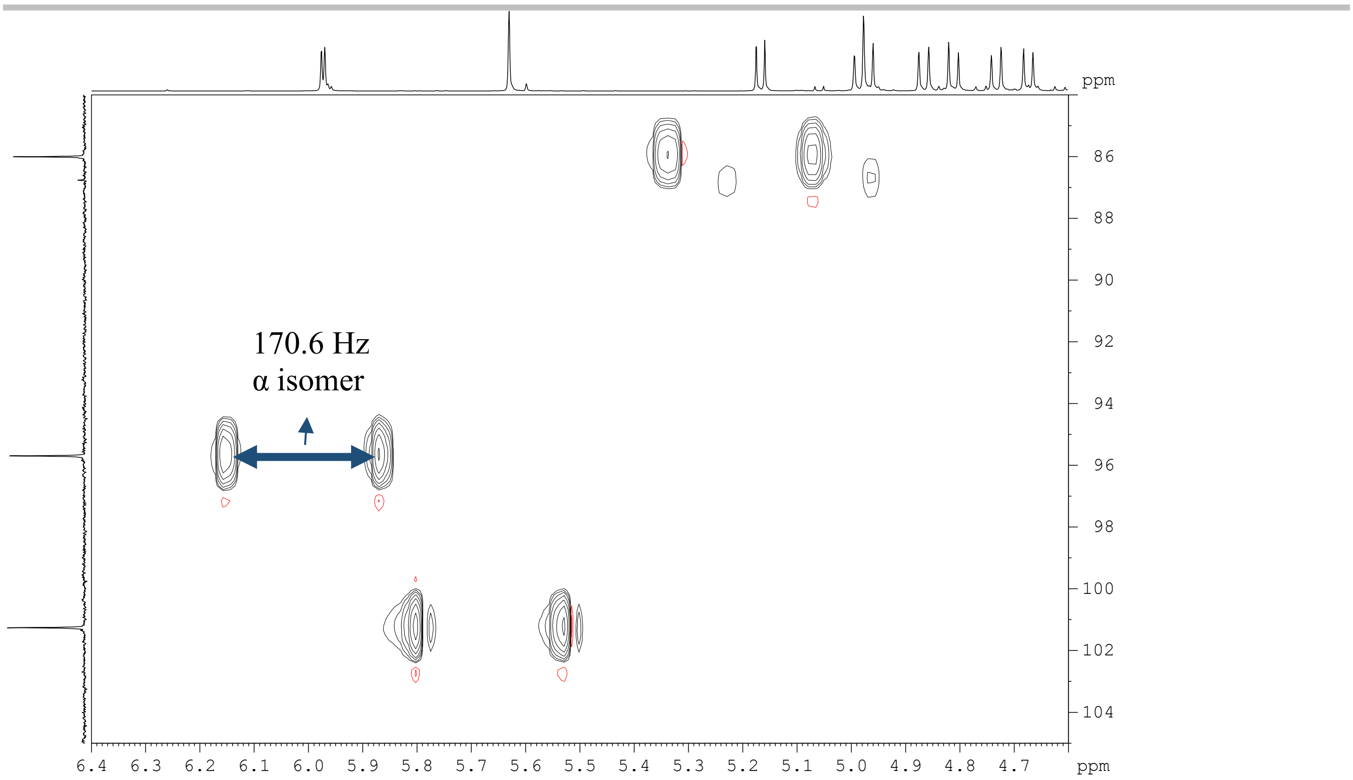

5-Aminopentyl α-D-glucopyranosyl-(1→2)-α-D-glucopyranosyl-(1→dihydrogen phosporyl→4)-α-L-rhamnopyranosyl-(1→3)-β-D-glucopyranosyl-(1→4)-[α-L-rhamnopyranosyl-(1→3)]-α-D-glucopyranosyl-(1→2)-α-D-glucopyranoside (2). $^{1}J_{CH}$ for α, β isomer.

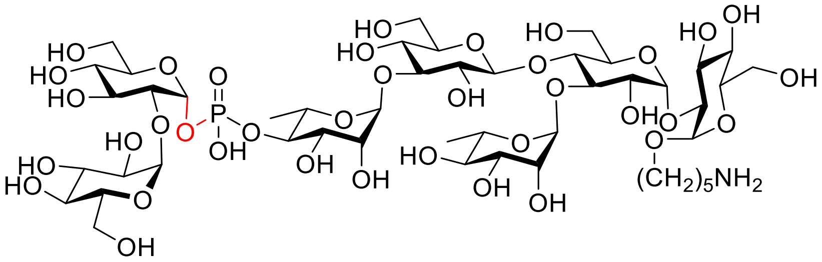

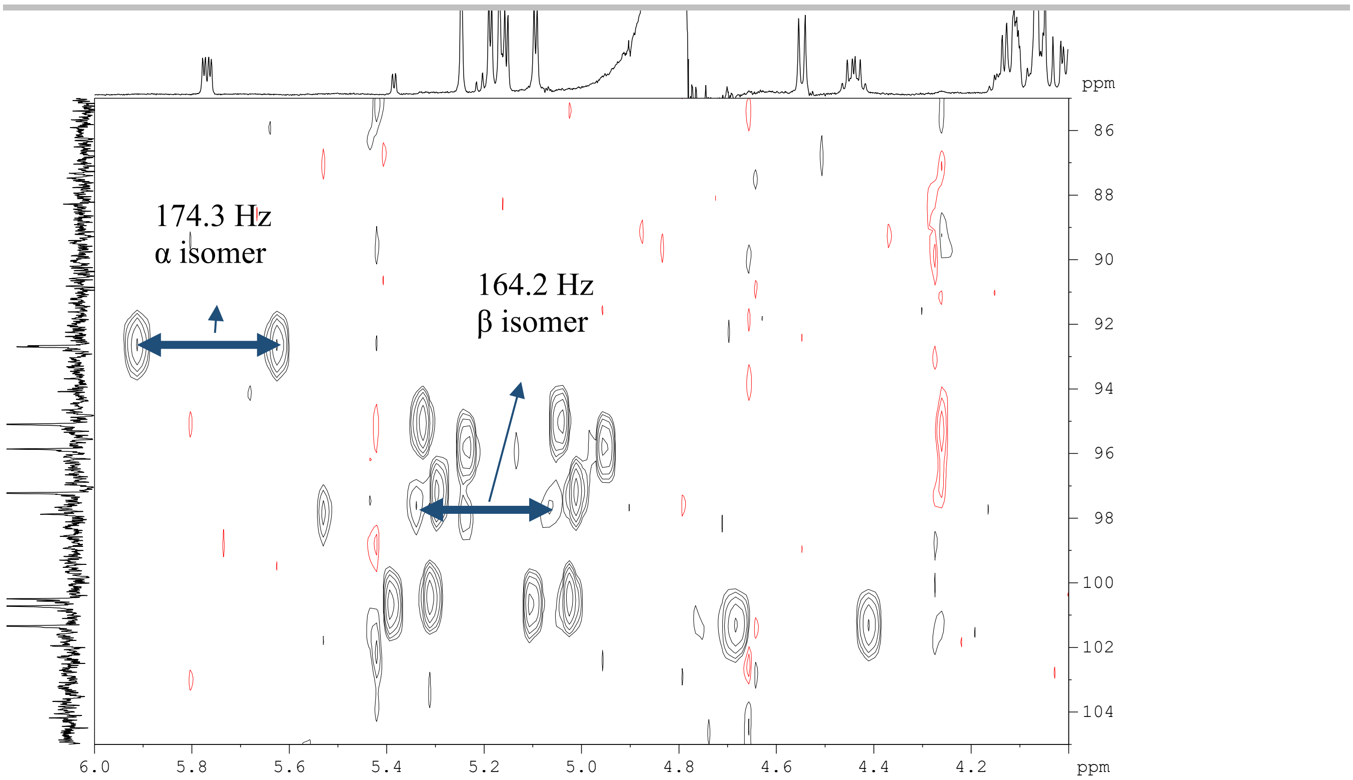

5-Azidopentyl 2,3-*O*-dibenzoyl-4-*O*-oxopentanoate-α-L-rhamnopyranosyl-(1→3)-2-*O*-benzoyl-4,6-*O*-benzylidene-β-D-glucopyranosyl-(1→4)-[2,3-di-*O*-benzoyl-4-*O*-benzyl-α-L-rhamnopyranosyl-(1→3)]-6-*O*-acetyl-2-*O*-benzyl-α-D-glucopyranosyl-(1→2)-3-*O*-benzyl-4,6-*O*-benzylidene-α-D-glucopyranosyl-(1→[phenylmethyl]-phosphate→4)-2,3-*O*-dibenzoyl-α-L-rhamnopyranosyl-(1→3)-2-*O*-benzoyl-4,6-*O*-benzylidene-β-D-glucopyranosyl-(1→4)-[2,3-di-*O*-benzoyl-4-*O*-benzyl-α-L-rhamnopyranosyl-(1→3)]-6-*O*-acetyl-2-*O*-benzyl-α-D-glucopyranosyl-(1→2)-3,4,6-*tri*-*O*-benzyl-α-D-glucopyranoside (36).

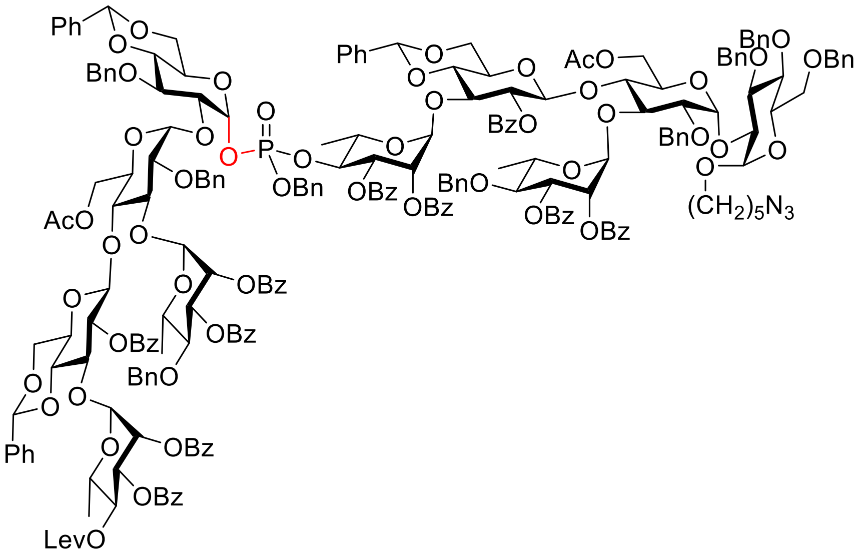

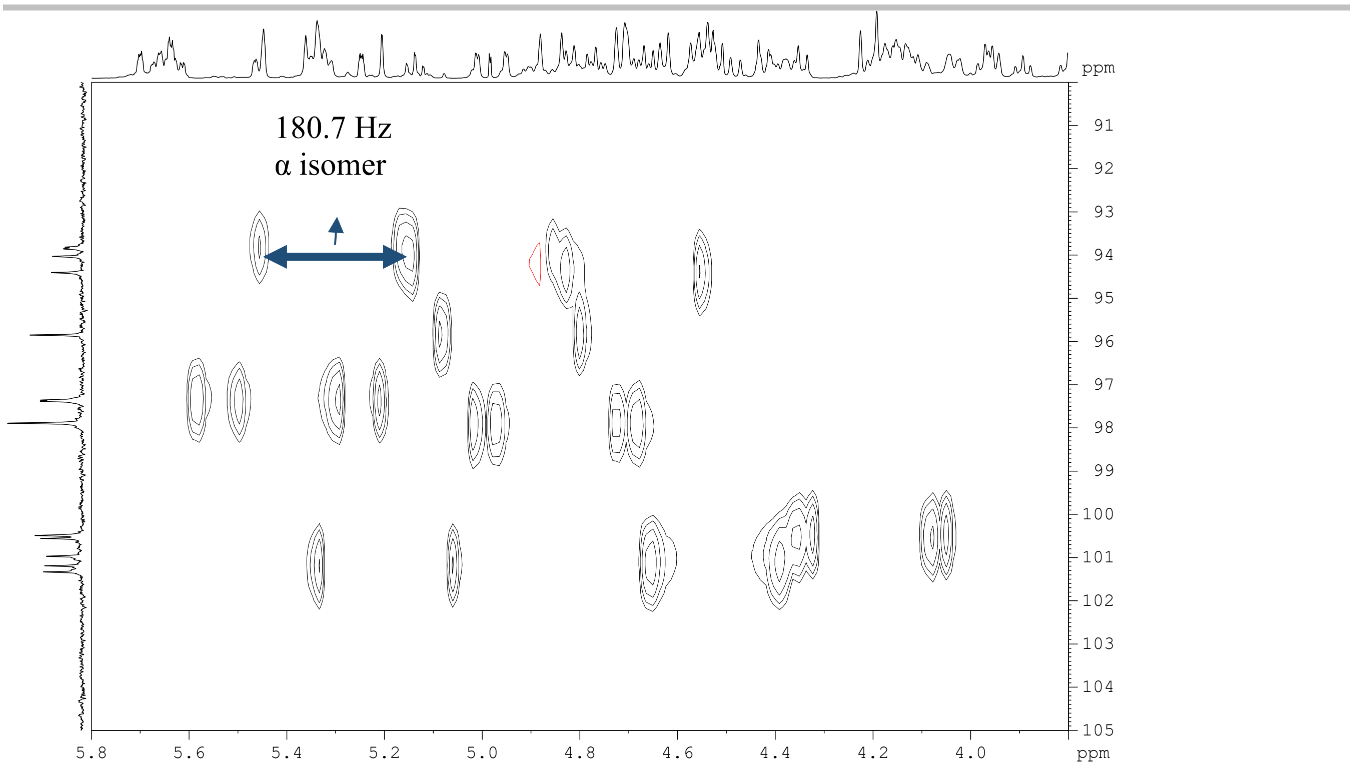

5-Aminopentyl α-L-rhamnopyranosyl-(1→3)-β-D-glucopyranosyl-(1→4)-[α-L-rhamnopyranosyl-(1→3)]-α-D-glucopyranosyl-(1→2)-α-D-glucopyranosyl-(1→dihydrogen phosporyl→4)-α-L-rhamnopyranosyl-(1→3)-β-D-glucopyranosyl-(1→4)-[α-L-rhamnopyranosyl-(1→3)]-α-D-glucopyranosyl-(1→2)-α-D-glucopyranoside (1). $^{1}J_{CH}$ for α, β isomer.

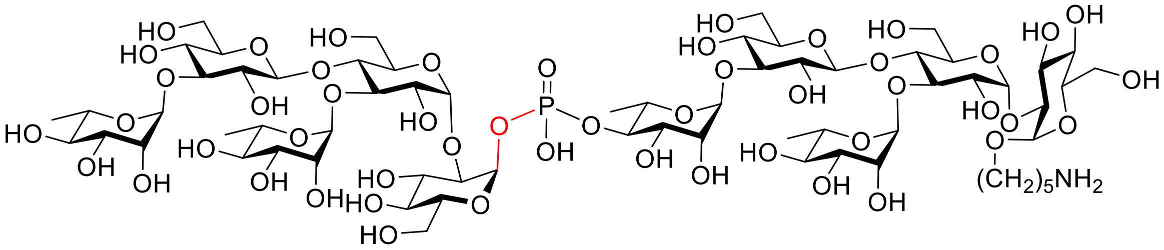

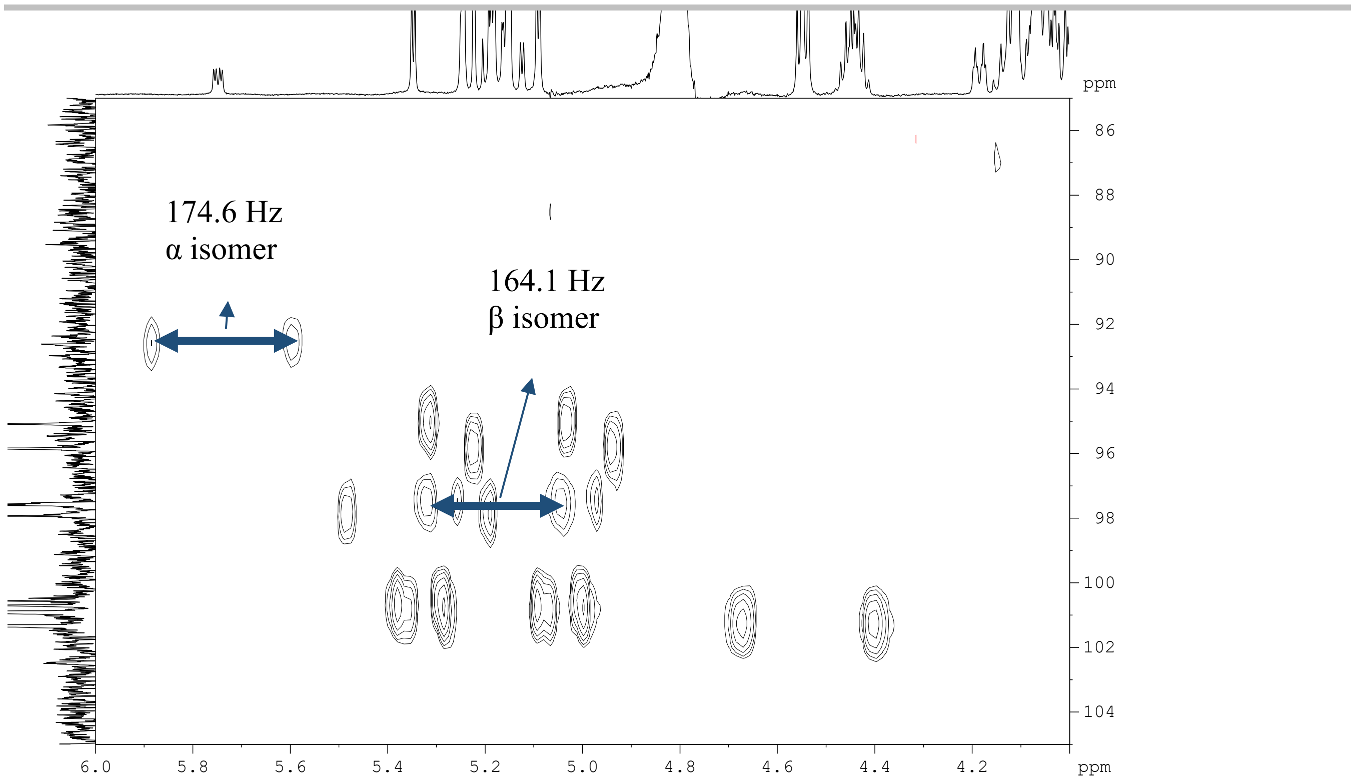
174.6 Hz
α isomer
164.1 Hz
β isomer
ppm
86
88
90
92
94
96
98
100
102
104
6.0
5.8
5.6
5.4
5.2
5.0
4.8
4.6
4.4
4.2
ppm
